# Oral and Poster abstracts

**DOI:** 10.1177/2397198319898367

**Published:** 2020-05-07

## WSF PROJECTS

## CO.01

## DEVELOPMENT OF THE OPTIMAL TOUCHSCREEN INTERFACE FOR PATIENTS WITH SCLERODERMA

G. Moroncini^1^, M. Germani^2^, A. Brunzini^2^, A. Papetti^2^, G. Cesaretti^1^, E. Filippini ^1^, V. Maurizi^1^, C. Fischetti^1^, L. Manfredi^1^, F. Grassetti^2^, R. Gesuita^3^, A. Gabrielli^1^

^1^*Department of Clinical and Molecular Sciences, Marche Polytechnic University, Ancona, Italy*, ^2^*Department of Industrial Engineering and Mathematical Sciences, Marche Polytechnic University, Ancona, Italy*, ^3^*Centre of Epidemiology and Biostatistics, Marche Polytechnic University, Ancona, Italy*

**Introduction:** Hand disability is a common consequence of scleroderma (SSc). Impaired hand function is a major contributor to overall disability and reduced health-related quality of life.

Since touchscreen technology is widely employed in personal and public devices (smartphones, tablets, ATM), this study investigated the performance of SSc patients with touchscreen interfaces.

**Material and Methods:** 1) a novel touchscreen application was developed to explore three different hand gestures: tapping, dragging and dropping, pinching to zoom. Each app session provided a set of mini-tasks in which the target object changed position on the screen, shape, color, size. The tablet device used in the study recorded task completion time and success rate.

2) after informed consent, this application was proposed to 80 SSc patients (63 with limited and 17 with diffuse cutaneous disease subset) consecutively followed by Clinica Medica, Ospedali Riuniti Ancona, Italy between November 1st 2018 and March 1st 2019, and to 80 sex- and age-matched healthy controls (HC). 21 subjects with Primary Raynaud’s Phenomenon (PRP) were also included.

3) performance parameters and corresponding task features were analyzed by Fisher exact test and Kruskal-Wallis test.

4) information gathered through data analysis was used to design a novel touchscreen adaptive user interface dedicated to facilitate the use by SSc patients.

**Results:** 1) SSc patients collectively showed statistically significant slower completion time and lower success rate compared to healthy controls in all of the three gestures explored. Unlike the tapping gesture, performance with the dragging/dropping and pinching to zoom gestures was affected by the different mini-tasks such as certain icon positions on the tablet and extent of icon zooming. No statistically significant difference was observed between the limited and the diffuse SSc disease subsets. Subjects with PRP showed a trend towards a worse performance than HC, without reaching statistical significance due to low number of patients.

2) An application containing three games based on the gestures tested in this study and adapted to facilitate the use by SSc patients was developed.

**Conclusions:** SSc patients are impaired in the use of touchscreen devices due to reduced hand function. A touchscreen adaptive interface dedicated to SSc patients was developed using the information collected in this study and may be exploited for a multicenter clinical study evaluating users satisfaction and hand function improvement after exercise.

## CO.02

## RANDOMIZED CONTROLLED TRIAL OF AN INTERNET-BASED EXERCISE PROGRAM TO IMPROVE HAND FUNCTION IN PATIENTS WITH SCLERODERMA: A SCLERODERMA PATIENT- CENTERED INTERVENTION NETWORK (SPIN) STUDY

L. Kwakkenbos^1^, M.-E. Carrier^2^, K. Turner^2^, L. Tao^2^, K. Aguila^2^, A. Carboni Jimenez^2^, M. Canedo Ayala^2^, S. Harb^2^, J. Welling^3^, M. Sauve^4,5^, C. van den Ende^6^, A. Schouffoer^7^, M. Hudson^2,8^, C. Nguyen^9^, I. Boutron^9,10^, F. Rannou^9^, B. Thombs^2,8,11^, L. Mouthon^9,10,12^, the SPIN Investigators^13^

^1^*Radboud University, Behavioral Science Institute, Clinical Psychology, Nijmegen, The Netherlands*, ^2^*Jewish General Hospital, Lady Davis Institute, Montreal, Canada*, ^3^*NVLE Dutch patient organization for systemic autoimmune diseases, Utrecht, The Netherlands*, ^4^*Scleroderma Society of Ontario, Hamilton, Canada*, ^5^*Scleroderma Canada, Ottawa, Canada*, ^6^*Sint Maartenskliniek, Department of Rheumatology, Nijmegen, The Netherlands*, ^7^*Leiden University Medical Center, Department of Rheumatology, Leiden, The Netherlands*, ^8^*McGill University, Department of Medicine, Montreal, Canada*, ^9^*Université Paris Descartes, Paris, France*, ^10^*Assistance Publique-Hôpitaux de Paris, Paris, France*, ^11^*McGill University, Department of Psychiatry, Montreal, Canada*, ^12^*Hôpital Cochin, Service de Médecine Interne, Paris, France*, ^13^*Scleroderma Patient-centered Intervention Network, Montreal, Canada*

**Introduction:** Significant functional impairment of the hands is nearly universal in systemic sclerosis (SSc, or scleroderma) and is a primary cause of disability that contributes to lower health-related quality of life (HRQL). Hand exercises may improve hand function, but developing, testing and disseminating rehabilitation interventions in SSc is challenging. The Scleroderma Patient-centered Intervention Network (SPIN) was established to address this issue, and has developed an online hand exercise program to improve hand function for SSc patients (SPIN-HAND). The SPIN-HAND trial is a pragmatic randomized control trial (RCT) embedded in the SPIN Cohort that evaluates the effect of SPIN’s online hand exercise program, in addition to usual care, on hand function and HRQL in SSc patients with at least mild hand function limitations.

**Material and Methods:** The SPIN-HAND program consists of 4 modules that address specific aspects of hand function, including (1) Thumb Flexibility and Strength; (2) Finger Bending; (3) Finger Extension; and (4) Wrist Flexibility and Strength. The program also integrates tools to support key components of successful self-management programs, including goal setting and feedback, social modeling, and mastery experiences. SPIN is in the process of randomizing 586 SPIN Cohort participants with at least mild hand function limitations (Cochin Hand Function Scale [CHFS] >= 3) and an indicated interest in using an online hand exercise program with a 3:2 ratio to be offered the hand exercise program or usual care only (Clinicaltrials.gov trial registration NCT03419208). The primary outcome analysis will compare CHFS scores 3-months post-randomization between patients offered versus not offered the intervention.

**Results:** Enrollment started on March 12, 2018 and at the time of abstract submission (September 2019), 443 SPIN Cohort participants were included in the SPIN-HAND RCT: 267 were allocated to the intervention arm, and 176 to the control arm. In total, 152 of 267 (57%) patients consented to use the SPIN-HAND intervention. Primary and secondary outcomes are forthcoming and will be presented at the World Scleroderma Congress.

**Conclusions:** The SPIN-HAND exercise program is a self-help tool that may improve hand function in patients with SSc. If supported by evidence, SPIN’s patient organization partners will disseminate the intervention free-of-charge to healthcare professionals and patients. The SPIN-HAND RCT is the first example of a trial conducted using the innovative cohort multiple RCT framework for adequately powered trials in a rare disease context and serves as a prototype for similar programs in other rare diseases.

## CO.03

## I’VE HANDED BACK MY MAN CARD: EXPERIENCES, COPING STYLES, AND SUPPORT PREFERENCES OF MEN WITH SYSTEMIC SCLEROSIS

C. Flurey^1^, J. Pauling^2^, L.A. Saketkoo^3^, C. Denton^4^, A. Herrick^5^, D. Khanna^6^, P. Galdas^7^, T. Frech^8^, A. Williams^2^, M. Sabbagh^6^, M. Hughes^9^

^1^*University of the West of England, Bristol, United Kingdom*, ^2^*Royal United Hospitals Bath NHS Foundation Trust, Bath, United Kingdom*, ^3^*Tulane University, New Orleans, USA*, ^4^*University College London, London, United Kingdom*, ^5^*University of Manchester, Manchester, United Kingdom*, ^6^*University of Michigan, Ann Arbor, USA*, ^7^*University of York, York, United Kingdom*, ^8^*The University of Utah, Salt Lake City, USA*, ^9^*Sheffield Teaching Hospitals NHS Foundation Trust, Sheffield, United Kingdom*

**Introduction:** Men with systemic sclerosis (SSc) have significantly reduced survival rates and report more severe disease than women. However, no previous psychosocial studies have focused solely on men with the condition. This study qualitatively explores experiences, coping strategies and support preferences of men with SSc.

**Material and Methods:** 6-8 focus groups (4-6/group) with male SSc patients purposively sampled to reflect a range of disease and demographic characteristics from the UK and USA. Data analysis is iterative using inductive thematic analysis.

**Results:** Data collection ongoing, full results will be presented. Results to date:

Three focus groups (n=13): mean disease duration 7years (SD 7.3); age 60years (SD: 9.3); 77% diffuse cutaneous SSc; 70% Caucasian.

Preliminary analysis suggests four broad themes:

“It’s a little embarrassing”: Erectile Dysfunction:

Participants reported erectile dysfunction (ED) as an important symptom impacting quality of life, that felt ignored by clinicians (“I had to figure it out”). They reported needing prompting to feel comfortable discussing ED (“It’s important they ask the question first”).

“You always think about how much this is going to shorten your life”: Mortality:

Participants discussed the life-limiting nature of SSc (“I worry a lot about what’s happening on the inside”). They grieved for future events they may not be around for, and planned ahead for death (“I figured [my wife] better know how to do this”).

“[My wife] makes more money than me”: Impact on masculinity:

Loss of the breadwinner role impacted participants’ sense of self-worth (“I ask myself what am I here for?”). They were resigned to needing practical help, but found it hard to accept (“You know how hard it is to have your wife…put your underwears on?”) and often used humour as a shield (“I say ‘here, I turned in my man-card, open this for me’”).

“I don’t harp on”: Social support:

Participants reported not discussing SSc with their friends (“that’s my personal business”). Whilst they will discuss the practical impact with family, they often protected them from the emotional impact (“I wanna tell people…but I’ve gotta try and stay positive and focused for as long as I can because I’ve got [mum and wife] depending on me”.

**Conclusions:** SSc impacts male patients’ masculine identity and roles. Some men withhold emotional impact from their family to maintain a protector role, which may limit their social support. Clinicians should be aware male patients report erectile dysfunction as an ignored symptom and need prompting to feel comfortable discussing this.

## WSF PRIZE for Professor Kazuhiko Takehara’s career

## CO.04

## UNRAVELLING THE AETIOPATHOGENESIS OF MORPHOEA BY NEXT GENERATION SEQUENCING OF PAIRED SKIN BIOPSIES

A.M. Saracino, C.P. Denton, D.J. Abraham

*Centre for Rheumatology and Connective Tissue Diseases, University College London Medical School, Royal Free Campus, London, United Kingdom*

**Introduction:** The precise aetiopathogenesis of morphoea is poorly understood. Importantly, the initiating genetic and molecular mechanisms remain unclear. Linear morphoea (LM) follows Blascho’s lines and hence may represent epidermal mosaicism. This, along with insights from related fibrotic processes, support the key role of the epidermis in disease initiation and propagation. The goal of this study was to investigate the genetic aetiologic and pathogenic basis of morphoea, focusing specifically on epidermal genomic variation and differential transcriptomic expression profiles in isolated epidermis and dermis. LM provides a unique template for this, by enabling intra-individual comparisons of clinically affected and contralateral site-matched unaffected skin, with background genetic homogeneity.

**Material and Methods:** Paired skin-biopsies were taken from 27 patients with LM. Epidermis and dermis was isolated using a 2-step chemical and physical separation protocol. WGS (n=4 epidermal; Illumina HiSeqX Ten-System, 70×) and RNA-seq (n=5 epidermal, n=5 dermal; BGISEQ-500, 20million-reads-per-sample) were performed on isolated tissue pairs (MREC Ref-6398, Royal Free Hospital). Somatic variant calling was performed using MuTect2, variants annotated with ANNOVAR and pathogenicity graded with SIFT, PolyPhen, POVEAN and CADD-scores. RNA-seq reads were examined for quality then aligned to human genome GRCh38. R v3.3.2 (edgeR) was utilised for gene expression analysis; GSEA (MSigDB v6.3) and PANTHER (v14.1) for pathway analyses. RTqPCR and whole-skin immunohistochemistry were used to validate key results.

**Results:** WGS of epidermis confirmed no single affected gene or developmental somatic mutation. However a number of potentially disease relevant protein-coding pathogenic and rare variants were seen, such as in ADAMTSL1, ADAMTS16; with known links to fibrosis and morphoea fibroblasts. Epidermal transcriptomic profiling demonstrated a highly proliferative, inflammatory and profibrotic signature, with overexpression of TNFa-via-NFkB, TGFb, IL6/JAKSTAT and IFN-signaling along with apoptosis, p53 and KRAS-responses. IFI27 was upregulated. Its induction of IFN-related-apoptosis could act as an epidermal initiating ‘damage’ signal. IFI27 also negatively regulates NR4A1, enabling persistent TGFb-signaling. Importantly, NR4A1 was correspondingly under-expressed in the dermis. Overall, morphoea dermis demonstrated a strong profibrotic, B-cell and IFN-signature, as well as upregulation of morphogenic pathways Wnt and Notch. WNT2 was upregulated in both epidermal and dermal isolates. Finally, downregulation of basement-membrane-zone LAMA4 was seen, providing a possible mechanism for epidermal-dermal communication. LAMA4 has known involvement in organ fibrosis.

**Conclusions:** This study supports possible complex polygenic epidermal mosaicism and the presence of key potential inciting and disease driving epidermal mechanisms in morphoea. We propose a possible genetic and molecular story underlying morphoea aetiopathogenesis.

## CO.05

## IMMUNIZATION OF MICE WITH HUMAN AT1R GENERATES AT1R-ACTIVATING ANTIBODIES AND INDUCES SSC-LIKE DISEASE

X. Yue^1^, X. Yu^1^, X. Wang^1^, J. Yin^1^, H. Heidecke^2^, G. Wallukat^3^, I. Schimke^3^, G. Riemekasten^4^, F. Petersen^1^

^1^*Research Center Borstel, Priority Area Asthma & Allergy, Borstel, Germany*, ^2^*CellTrend GmbH, Luckenwalde, Germany*, ^3^*Berlin Cures GmbH, Berlin, Germany*, ^4^*University of Lübeck, Department of Rheumatology, Lübeck, Germany*

**Introduction:** Systemic sclerosis (SSc) is an autoimmune disease targeting multiple organs with hallmarks of autoimmunity, vasculopathy and fibrosis. As is typical for many other systemic autoimmune diseases, the pathogenesis of SSc is only partially understood and the pathogenic autoantibodies and autoantigens are ill defined. Functional autoantibodies against angiotensin II receptor I (AT1R) might play a pathogenic role in vascular and fibrotic diseases and, particularly, in the development of systemic sclerosis (SSc). In this study we aimed to verify this hypothesis in vivo by a novel mouse model based on immunization with with human AT1R.

**Material and Methods:** We immunized C57BL/6J mice with human AT1R (hAT1R) by using a novel strategy, which retains the conformational epitopes of the antigen in their native state. At week 10 after immunization, mice were scarified and tissues were examined histologically for disease symptoms. Serum levels of anti-AT1R IgG and cytokines were analyzed. Moreover, the capacity of serum IgG to activate AT1R in vitro was analyzed. Finally, monoclonal antibodies directed to hAT1R were generated by this animal model and applied to naïve mice to induce disease symptoms.

**Results:** The hAT1R-immunized mice developed functional autoantibodies against AT1R, which can bind and activate the native receptor. Furthermore, hAT1R-immunization induced SSc-like disease symptoms including perivascular and interstitial inflammation in the lung as well as perivascular inflammation and fibrosis in the skin. Importantly, the SSc-like disease can be partially transferred by serum IgG of hAT1R-immunized mice as well as by functional monoclonal aab to AT1R raised from this animal model.

**Conclusions:** Our results prove directly the pathogenic role of functional anti-AT1R antibodies in skin and lung inflammation, in skin fibrosis, and their role in immune cell migration to selective tissues. They provide strong evidence that at least parts of the SSc-pathology is mediated by functional autoantibodies to AT1R. In addition, this novel disease concept based on the effects of functional autoantibodies could be of relevance for further systemic autoimmune diseases and may serve as a therapeutic target in the future.

## SESSION 1 - PULMONARY

## CO.06

## COMPUTED TOMOGRAPHY-BASED RADIOMICS FEATURES FOR DETECTION AND STAGING OF INTERSTITIAL LUNG DISEASE IN SYSTEMIC SCLEROSIS – TRANSFERABILITY FROM EXPERIMENTAL TO HUMAN LUNG FIBROSIS

J. Schniering^1^, H. Gabrys^2^, M. Brunner^1^, D. Kenkel^3^, O. Distler^1^, M. Guckenberger^2^, M. Bogowicz^2^, D. Vuong^2^, K. Karava^2^, T. Frauenfelder^3^, S. Tanadini-Lang^2^, B. Maurer^1^

^1^*Center of Experimental Rheumatology, Department of Rheumatology, University Hospital Zurich, Zurich, Switzerland*, ^2^*Department of Radiation Oncology, University Hospital Zurich, Zurich, Switzerland*, ^3^*Institute of Diagnostic and Interventional Radiology, University Hospital Zurich, Zurich, Switzerland*

**Introduction:** High resolution computed tomography (HRCT) is a cornerstone in the diagnosis and monitoring of interstitial lung disease (ILD) in systemic sclerosis (SSc).

Radiomics is a promising research field, which automatically extracts mineable quantitative data from medical images, aiming to identify imaging biomarkers.

In this study, we evaluated 1) the potential of CT-based radiomics features for diagnosis and staging of experimental ILD and 2) the transferability of radiomics signatures to human ILD.

**Material and Methods:** Radiomics analysis was performed on micro-CT scans from bleomycin (BLM)-treated mice and NaCl-treated controls at days 3, 7, 14, 21, 28 and 35 after intratracheal BLM instillation (n=6-9/group), as well as on human HRCT scans from 102 SSc patients. In total, 154 radiomics features were extracted from segmented lungs using the in-house developed software Z-Rad (Python 2.7). A univariate logistic model was built for each feature to discriminate between healthy and diseased subjects as well as limited (<20% disease extent on CT) and extensive (> 20% disease extent on CT) fibrosis, and the transferability between mice and humans was quantified.

**Results:** Radiomics features with good predictive performance (area under the receiver operating characteristic curve (AUC) >0.7 and p-value<0.05) were considered as candidate discriminators. Under this criterion, we identified 105/154 radiomics features, describing image intensity and texture that differentiated NaCl controls from BLM-treated mice and 56/154 features that discriminated limited (<20%) from extensive (>20%) BLM-induced ILD.

In human SSc-ILD, 124/154 features differentiated ILD from non-ILD and 121/154 features discriminated limited from extensive fibrosis. Notably, for diagnosis 89 and for staging 46 features overlapped between mice and humans, resulting in 85% or 82% transferability, respectively.

**Conclusions:** The results of this proof-of-concept study suggest that 1) the well-established mouse model of BLM-induced ILD is a valuable model system to test defined hypotheses in radiomics research and 2) that radiomics features show great potential as quantitative imaging biomarkers for diagnosis and staging of SSc-ILD.

## CO.07

## DISEASE COURSE OF PROGRESSIVE INTERSTITIAL LUNG DISEASE IN SYSTEMIC SCLEROSIS: A 5-YEAR FOLLOW-UP FROM THE EUSTAR DATABASE

A. Hoffmann-Vold^1^, Y. Allanore^2^, M. Alves^3^, P. Airò^4^, L. Czirják^5^, S. Guiducci^6^, E. Hachulla^7^, M. Li^8^, C. Mihai^9^, G. Riemekasten^10^, P. Sfikakis^11^, O. Kowal-Bielecka^12^, O. Distler^9^

^1^*Department of Rheumatology, Oslo University Hospital, Oslo, Norway*, ^2^*Department of Rheumatology A, Descartes University, APHP, Cochin Hospital, Paris, France*, ^3^*Boehringer Ingelheim International GmbH, Ingelheim am Rhein, Germany*, ^4^*UO Reumatologia e Immunologia Clinica, Spedali Civili di Brescia, Brescia, Italy*, ^5^*Department of Rheumatology & Immunology, Medical School, University of Pécs, Pécs, Hungary*, ^6^*Department of Clinical & Experimental Medicine, Section of Rheumatology, University of Florence, Florence, Italy*, ^7^*Department of Internal Medicine & Clinical Immunology, Hôpital Claude Huriez, University of Lille, Lille, France*, ^8^*Department of Rheumatology, Peking Union Medical College Hospital (West Campus), Beijing, China*, ^9^*Department of Rheumatology, University Hospital Zurich, Zurich, Switzerland*, ^10^*Department of Rheumatology & Clinical Immunology, University Medical Center Schleswig-Holstein, Lübeck, Germany*, ^11^*Joint Rheumatology Programme, National & Kapodistrian University of Athens Medical School, Athens, Greece*, ^12^*Department of Rheumatology & Internal Medicine, Medical University of Bialystok, Bialystok, Poland*

**Introduction:** Patients with systemic sclerosis-associated interstitial lung disease (SSc-ILD) may develop progressive ILD, but disease course and patterns are unknown. Here we report on the disease course of SSc-ILD over a 5-year observation period for patients in the European Scleroderma Trials and Research group (EUSTAR) database.

**Material and Methods:** Adult patients, registered in the EUSTAR database since 2010, who fulfilled SSc classification criteria and had ILD on high-resolution computed tomography, serial lung function and disease duration recordings, were eligible. Disease course over the 5-year follow-up period was assessed in patients with >=3 forced vital capacity (FVC) measurements available, and they were divided into subgroups based on the difference between the first and last available FVC measurement (% predicted). The FVC course for each year was evaluated by determining the magnitude of FVC changes in individual patients in each 12-month period during the 5-year follow-up. Changes in FVC (% predicted) were defined as follows: major decline (>20%); significant decline (>10–<=20%); moderate decline (5–10%); stable (decline/improvement <5%); and improvement (>=5%). Patients who experienced no decline, one episode of decline (significant or moderate), or multiple FVC declines (significant, moderate or both) over the 5-year observation period were assessed and patterns of ILD progression determined.

**Results:** Serial lung function data were available for 535/826 (65%) SSc-ILD patients. Based on the overall change from the first to the last FVC measurements, 200 patients experienced overall FVC decline: 49 (9%) had major decline; 75 (14%) had significant decline (>10–<=20%); 76 (14%) had moderate decline (5–10%). Of the remaining patients, 206 (39%) were stable (decline/improvement <5%), and 129 (24%) experienced improvement (>=5%). Based on FVC change during each 12-month period, 178 patients (33%) experienced no FVC decline of >=5% during any 12-month interval; 220 patients (41%) experienced one 12-month interval with significant or moderate FVC decline, and 137 (26%) experienced two or more 12-month intervals with significant and/or moderate FVC declines (see Table).

**Conclusions:** The disease course of SSc-ILD is heterogeneous, and variable FVC changes during 1-year periods did not consistently predict overall FVC changes. Regular patient monitoring is required, to identify progression early, enable early intervention and help improve patient outcomes.

**Funding:** Boehringer Ingelheim International GmbH, Germany

**Figure:**
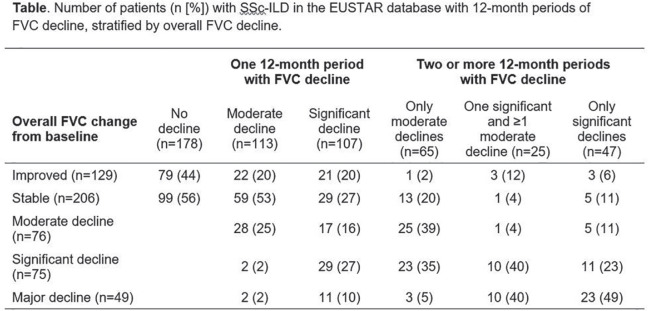


## CO.08

## NEW HEMODYNAMIC DEFINITIONS OF PULMONARY HYPERTENSION: IS IT USEFUL IN SYSTEMIC SCLEROSIS PATIENTS?

A. Volkov, N. Yunkina, E. Nikolaeva, I. Kurmukov

*Scientific Research Institute of Rheumatology named after V.A. Nasonova, Moscow, RUSSIA*

**Introduction:** Pulmonary arterial hypertension, associated with systemic sclerosis (PAH - SSc) - devastating disease with high mortality, but early treatment may considerably improve outcome.

Purpose: to assess the value of the new diagnostic criteria (mPAP < 20 mm Hg) in PAH verification in patients with SSc.

**Material and Methods:** The retrospective analysis of a cohort of the patients observed in our Center for the last 10 years was performed. In total, we analyzed the results of 303 right heart catheterization (RHC) in.156 SS patients (mean age 51±14 yrs). Among these patients there were 100 with PAH, 13 - with PH group II, 13 - with group III ant 30 SSc patients with mean pulmonary artery pressure (mPAP) less than 25 mm.Hg.

**Results:** The correlation between mPAP and pulmonary vascular resistant (PVR) was calculated. The regression equation is presented in the drawing (r = 0.86, p < 0,0001). According to the equation, the mPAP 20 mm.Hg measures up PVR 2 WU and PVR 3 WU will equal mPAP 24 mm.Hg. Also, mild correlation between pulmonary artery wedge pressure (PAWP) and age was found (r = 0.21, p = 0.006).

The group of SSc patients (mean age 54±11 y) with mPAP less than 25 mm.Hg., which was investigated for the algorithm DETECT validation is separately analyzed. 51 RHC were performed in this group (right atrial pressure was 3±2 mm.Hg., mPAP - 17±4 mm.Hg., PAWP - 8±2 mm.Hg., cardiac output (CO) 5.6±1.2 l/min., PVR 1.8±0.8 WU). 10 patients had mPAP more than 20 mm.Hg., but PAH, according with the new definition could be diagnosed only at one patient with mPAP 24 mm.Hg. and PVR 4 WU.

The mean observation period of this group is 61±7 month. During this time, PAH according the old definition developed in three patients with initial mPAP 22, 24 and 18 mm.Hg.

**Conclusions:** SSc patients with mPAP interval between 21 to 24 mm.Hg. seldom meets PVR more than 2 WU. Only 2 patients from 10 for the 5th year follow up developed PAH according the old definition.

## CO.09

## VEGF-C AND ITS RECEPTOR VEGFR-3 AS POTENTIAL SERUM BIOMARKERS FOR PULMONARY ARTERIAL HYPERTENSION IN SYSTEMIC SCLEROSIS

H. Didriksen^1^, Ø. Molberg^1^, H. Fretheim^1^, E. Gude^1^, V. Palchevskiy^2^, S. Jordan^3^, T. Garen^1^, Ø. Midtvedt^1^, A. Andreassen^1^, O. Distler^3^, J. Belperio^2^, A.-M. Hoffmann-Vold^1^

^1^*Oslo University Hospital, Oslo, Norway*, ^2^*University of California Los Angeles, Los Angeles, USA*, ^3^*Zurich University Hospital, Zurich, Switzerland*

**Introduction:** Pulmonary arterial hypertension (PAH) is a major complication in systemic sclerosis (SSc). Increased lymphangiogenesis in lung tissue from murine PAH-models has been shown by expression of vascular endothelial growth factor receptor 3 (VEGFR-3), a known lymphatic marker. We have previously shown that VEGF-C is downregulated in SSc patients and aim here to assess the predictive value of VEGF-C and VEGFR-3 in PAH development in SSc.

**Material and Methods:** Sera samples from the SSc cohorts at Oslo University Hospital (OUH) (n=457) and Zurich University Hospital (USZ) (n=95), and healthy controls (n=68) were analyzed for soluble VEGF-C and VEGFR-3 levels by Luminex analysis. Consecutive SSc patients showing clinically suspicion of PH were referred to right heart catheterization (RHC) and those with conducted RHC were included. Mean pulmonary arterial pressure (mPAP) >21 mmHg in the absence of significant interstitial lung disease, pulmonary artery wedge pressure (PAWP) <15 mmHg and pulmonary vascular resistance (PVR) >3 Wood Units (WU) was defined as PAH. For analysis of association of VEGF-C, VEGFR-3 and VEGF-C/VEGFR-3 ratio and PAH, data from serum samples that had been drawn 5 years before to 1 year after RHC and PAH diagnosis were included, and for the predictive value on PAH development, samples that had been drawn > 6 months before PAH diagnosis. Logistic and Cox regression analyses were performed.

**Results:** Demographic and clinical data of SSc patients were similar in the OUH and USZ cohorts, and were assembled for further analysis. Serum levels of VEGFR-3 were significantly higher in SSc compared to healthy controls (1.8±0.20 ng/ml vs. 0.5±0.06 ng/ml, p=0.001) while VEGF-C was significantly lower (2.1±0.04 ng/ml vs. 2.9±0.12 ng/ml, p<0.001) as previously shown. A total of 238 patients had conducted RHC. Of these, 59 patients had no PH and 68 had PAH. In multivariable logistic regression adjusted for age and gender, VEGF-C (OR 0.99, 95%CI 0.998-0.999, p<0.001) was associated with PAH compared to no PAH patients, but not VEGFR-3. In total, 43 patients developed PAH 2.4±1.6 years after sera was drawn. In multivariable analyses adjusted for age and gender, VEGF-C was predictive for PAH development (HR 0.99 95% CI 0.995-0.998, p=0.014), and a trend was found for VEGF-C/VEGFR-3 ratio (HR 1.00 95% CI 0.999-1.005, p=0.059), but not VEGFR-3.

**Conclusions:** We have shown in two well characterized SSc cohorts that VEGF-C and VEGFR-3 seem to be dysregulated in SSc patients and specifically in SSc-PAH patients. VEGF-C appears as a promising marker for SSc-PAH prediction in SSc.

## SESSION 2 - HEART

## CO.10

## SERUM CARDIAC BIOMARKERS AND CARDIAC MRI DIFFUSE FIBROSIS MAY PREDICT THE DEVELOPMENT OF CARDIOVASCULAR EVENTS IN SYSTEMIC SCLEROSIS PATIENTS

R.-B. Dumitru^1^, L.-A. Bissell^1^, B. Erhayiem^2^, G. Fent^2^, A. Kidambi^2^, A. Burska^2^, G. Abignano^1^, H. Donica^3^, J.P. Greeenwood^2^, J. Biglands^4^, F. Del Galdo^1^, S. Plein^2^, M.H. Buch^1,5^

^1^*Leeds Institute of Rheumatic and Musculoskeletal Medicine, University of Leeds, Leeds, United Kingdom*, ^2^*Department of Biomedical Imaging Science, Leeds Institute of Cardiovascular and Metabolic Medicine, University of Leeds, Leeds, United Kingdom*, ^3^*Department of Biochemical Diagnostics, Medical University of Lublin, Leeds, United Kingdom*, ^4^*National Institute for Health Research, Leeds Biomedical Research Centre, Leeds, United Kingdom*, ^5^*Division of Musculoskeletal & Dermatological Sciences, University of Manchester & NIHR Manchester Biomedical Research C, Manchester, United Kingdom*

**Introduction:** Primary systemic sclerosis heart involvement (pSSc-HI) is described in the majority of systemic sclerosis (SSc) patients when cardiovascular magnetic resonance (CMR) is employed however, the prognostic implication of subclinical pSSc-HI findings is unknown. This study aimed to evaluate whether CMR and serum cardiac biomarkers can predict cardiovascular (CV) outcomes in patients with pSSc-HI.

**Material and Methods:** SSc patients, fulfilling the 2013 ACR/EULAR criteria, with no cardiovascular (CV) disease, diabetes and no more than 2 CV risk factors had baseline 3T CMR, with late gadolinium enhancement (LGE), T1 mapping with extracellular volume (ECV) quantification for assessment of diffuse fibrosis, and stress perfusion, and high-sensitivity troponin I (hs-TnI) and N-terminal pro b-type natriuretic peptide (NT-proBNP) measured. Follow-up clinical and echocardiographic data to identify CV outcomes were recorded. CV outcomes were defined as episodes of myocarditis, heart failure, rhythm disturbances and/or any new echocardiographic abnormalities including systolic dysfunction, diastolic dysfunction > grade 1.

**Results:** 75 participants were included in this study, with a median (IQR) age of 57 (49, 64) years, 33% diffuse cutaneous SSc, 39% interstitial lung disease (ILD), 29% Scl70+. Patients were followed up for a median (IQR) of 22 (15,54) months. Ten patients developed CV outcomes, comprising one diagnosis of myocarditis and 9 arrhythmias: 3 non-sustained ventricular tachycardia and 6 supraventricular arrhythmias. Median (IQR) time to CV outcome was 14 (8,27) months. Patients with CV outcomes had higher left ventricular (LV) mass [mean (SD) 48 (10) vs 43 (11), p=0.127] and an ECV above the normal threshold (>29%) was found in 8/9 patients with CV outcomes versus 40/62 patients with no CV outcomes (p=0.144). The probability of CV outcomes was significantly higher in those with NT-proBNP >125 pg/ml compared to those with normal NT-proBNP levels (X2= 4.19, p=0.045) and trend for poorer survival course was noted in those with higher ECV compared to those with normal ECV values (X2= 2.659, p = 0.103) (Figure 1).

Hs-TnI and NT-proBNP>125pg/ml associated with the presence of CV outcomes at univariate analysis (OR=4.594, p=0.031; OR=1.009, p=0.044). In a predictive model using penalised likelihood estimation, NT-proBNP>125 pg/ml associated with the development of CV outcomes (OR=5.918, p=0.028), with ECV>29% almost reaching statistical significance (OR=5.838, p=0.075).

**Conclusions:** Serum cardiac biomarkers and CMR findings are associated with adverse cardiovascular outcomes in patients with pSSc-HI CV. Larger longitudinal studies are required to confirm the predictive potential and inform management pathways for early detection of pSSc-HI.

**Figure:**
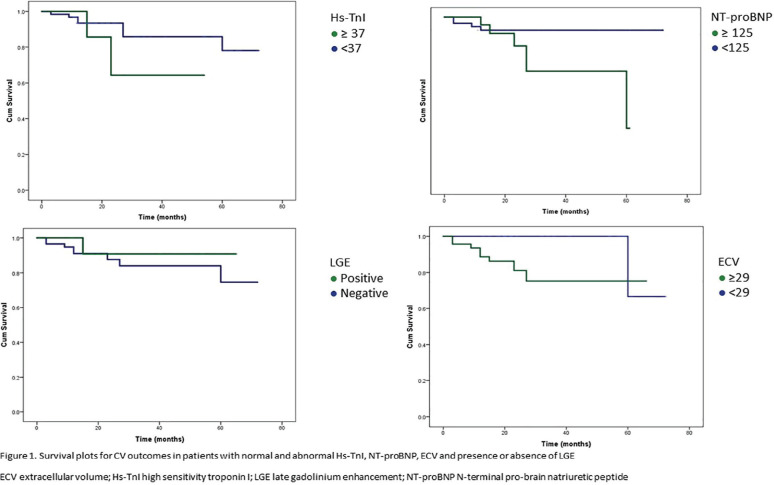


## CO.11

## SYSTEMIC SCLEROSIS MYOCARDITIS HAS UNIQUE CLINICAL, HISTOLOGICAL AND PROGNOSTIC FEATURES: COMPARATIVE ANALYSIS BETWEEN PATIENTS WITH ENDOMYOCARDIAL BIOPSY-PROVEN MYOCARDITIS

G. De Luca^1^, S.L. Bosello^2^, C. Campochiaro^1^, S. Sartorelli^1^, G. Peretto^3^, S. Sala^3^, G. Canestrari^2^, E. De Lorenzis^2^, S. Rizzo^4^, C. Basso^4^, G. Thiene^4^, A. Palmisano^5^, A. Esposito^5^, P. Della Bella^3^, E. Gremese^2^, L. Dagna^1^

^1^*Unit of Immunology, Rheumatology, Allergy and Rare Diseases, IRCCS San Raffaele Hospital, Vita-Salute University, Milan, Italy*, ^2^*Rheumathology Division, Fondazione Policlinico Universitario A. Gemelli IRCCS, Rome, Italy*, ^3^*Department of Cardiac Electrophysiology and Arrhythmology, IRCCS San Raffaele Hospital, Milan, Italy*, ^4^*Cardiovascular Pathology Unit, Department of Cardiac, Thoracic and Vascular Sciences, University and Hospital of Padua, Padua, Italy*, ^5^*Cardiac Magnetic Resonance Unit, Department of Radiology and Cardiovascular Imaging, IRCCS San Raffaele Hospital, Milan, Italy*

**Introduction:** Myocarditis is a life-threatening inflammatory disease increasingly reported in Systemic Sclerosis (SSc). We aimed to outline the clinical, histological and prognostic features of SSc endomyocardial biopsy (EMB)-proven myocarditis with respect to those of others EMB-proven virus-negative myocarditis (VNM).

**Material and Methods:** we retrospectively analysed data from 3 cohorts of EMB-proven myocarditis: SSc-related (SSc-VNM); isolated VNM(i-VNM); VNM related to other systemic autoimmune diseases(a-VNM), matched by age, gender and cardiovascular risk profile. On EMB, the degree of myocardial fibrosis was expressed as relative percentage and fibrotic score fro0 to 3. Clinical data, cardiac enzymes, echocardiogram, 24h-ECG-Holter and cardiac magnetic resonance (CMR), were obtained at baseline and at 3, 6 and 12 months, then during follow-up as clinically needed. Myocarditis-related complications (cardiac death, end-stage heart failure [HF], malignant arrhythmias or need for ICD-implantation) were recorded during follow-up(at least 1-year). Nonparametric tests were used.

**Results:** we enrolled 12 SSc-VNM(11 females, mean age 49.3±14.2years, 8 anti-Scl70+, 7 diffuse-SSc, 5 early-SSc, 4 with digital ulcers, 4 patients with interstitial lung disease), 12 i-VNM(12 females, mean age 47.7±10.8years) and 12 a-VNM(4 females, mean age 48.4±16.3years). On EMB, SSc-patients had higher degrees of myocardial fibrosis as assessed by both relative percentage(SSc-VNM:44.8±18.8%; a-VNM:28.6±16.5%; i-VNM:24.9±10.3%;p=0.019) and fibrotic score(SSc-VNM:2.3±0.8; a-VNM:1.4±1.1; i-VNM:1.2±0.7;p=0.002). Myocardial fibrosis directly correlated with skin score(r=0.625,p=0.03) and number of ventricular ectopic beats(VEBs) on 24h-ECG-Holter in SSc-patients(r=0.756,p=0.01). Dyspnoea class was higher at presentation in SSc-VNM patients(p=0.041) and we found heart failure (HF) only in SSc-patients(25%) (p=0.05). Levels of troponin T and NT-proBNP, left ventricular ejection fraction and number of VEBs on 24h-Holter did not differ between groups(p=ns). At CMR, myocardial oedema was nearly undetectable in SSc-VNM compared to others(p=0.02). All patients received immunosuppressive treatment. As about the clinical outcome, arrhythmic complications and need for ICD insertion were comparable between groups(p=ns). The number of patients who died during follow-up due to cardiac complications was significantly higher in SSc-VNM patients (50%), as compared to a-VNM(0%) and i-VNM(8.3%)(p=0.006). Patients who died during follow-up had higher degrees of myocardial fibrosis(52.2±11.6% vs 27.5±12.9%,p=0.024; fibrotic score:2.83±0.41 vs 1.4±0.9,p<0.001), higher number of VEBs at 24h-ECG Holter(p=0.04) and more frequently had a right-ventricular dysfunction at CMR(p=0.04).

**Conclusions:** SSc myocarditis has unique clinical and histological features, as it tends to present more frequently with HF and a higher dyspnoea class and to show higher degrees of myocardial fibrosis. These peculiar and distinctive features are paralleled by a worst cardiac prognosis.

**Figure:**
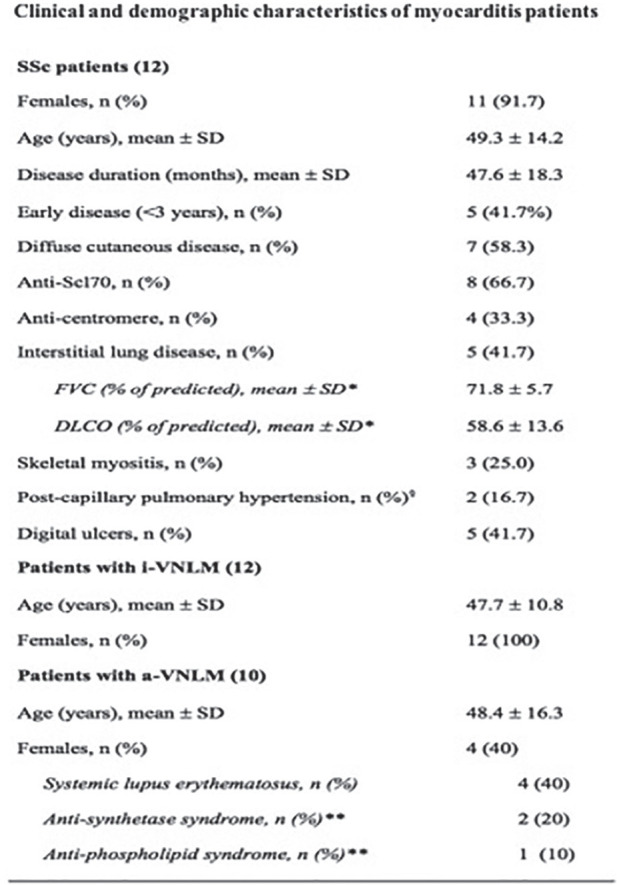


## CO.12

## PRIMARY SYSTEMIC SCLEROSIS HEART INVOLVEMENT (PSSCHI): A SYSTEMATIC LITERATURE REVIEW (SLR) AND PRELIMINARY CONSENSUS-BASED DEFINITION.

C. Bruni^1^, M.H. Buch^2^, A. Djokovic^3^, G. De Luca^3^, R.B. Dumitru^2^, A. Giollo^2,5^, M. Polovina^6^, Y.A. Suliman^7^, K. Bratis^8^, A. Steelandt^9^, I. Milinkovic^6^, A. Baritussio^10^, G. Hasan^11^, A. Xintarakou^12^, Y. Isomura^13^, G. Markousis Mavrogenis^14^, S. Bellando Randone^1^, S. Mavrogeni^14^, L. Gargani^15^, A.L. Caforio^10^, C. Tschoepe^16^, A. Ristic^6^, K. Klingel^17^, S. Plein^18^, E. Behr^19^, Y. Allanore^7^, M. Kuwana^13^, C. Denton^20^, D.E. Furst^1^–^21^, D. Khanna^22^, T. Krieg^23^, R. Marcolongo^24^, P. Seferovic^7–25^, M. Matucci-Cerinic^1^

^1^*Department of Experimental and Clinical Medicine, Division of Rheumatology, University of Florence, Florence, Italy*, ^2^*University of Leeds, Leeds Institute of Rheumatic and Musculoskelettal Medicine, Leeds, United Kingdom*, ^3^*Department of Cardiology, University Hospital Centre Bezanijska kosa and University of Belgrade, Faculty of Medicine, Belgrade, Serbia*, ^4^*Vita Salute San Raffaele University, IRCSS San Raffaele Hospital, Unit of Immunology, Rheumatology Allergology and Rare, Milan, Italy*, ^5^*University of Verona, Dept. Medicine, Rheumatology Unit, Verona, Italy, Verona, Italy*, ^6^*Department of Cardiology of the Clinical Centre of Serbia, Belgrade University School of Medicine, Belgrade, Serbia*, ^7^*Rheumatology Department, Paris University, Cochin Hospital, Paris, France*, ^8^*University of Manchester, Division of Cardiovascular Sciences, Manchester, United Kingdom*, ^9^*Assuit University Hospital, Department of Rheumatology and Rehabilitation, Faculty of Medicine, Assuit University Hospit, Assuit, Egypt*, ^10^*University of Padua, Department of Cardiac, Thoracic, Vascular Sciences and Public Health, Padua, Italy*, ^11^*Rutgers Robert Wood Johnson Medical Centre, Department of Medicine, New Brunswick, USA*, ^12^*Mitera General Hospital, Hygeia Group, Athens, Greece*, ^13^*Nippon Medical School Graduate School of Medicine, Department of Allergology and Rheumatology, Tokio, Japan*, ^14^*Onassis Cardiac Surgery Centre and Kapodistrian University of Athens, Athens, GREECE*, ^15^*National Research Council, Institute of Clinical Physiology, Pisa, Italy*, ^16^*Charité University Medicine Berlin, Department of Internal Medicine and Cardiology, Charité Campus Virchow Klinikum, Berlin, Germany*, ^17^*University of Tuebingen, Institute for Patology and Neuropathology, Cardiopathology, Tuebingen, Germany*, ^18^*University of Leeds, Leeds Institute of Cardiovascular and Metabolic Medicine, Dept. Biomedical Imaging Science, Leeds, United Kingdom*, ^19^*Saint George University of London, Institute of Molecular and Clinical Sciences, Cardiology Clinical Academic Group, London, United Kingdom*, ^20^*Royal Free Hospital, Dept. Rheumatology, London, United Kingdom*, ^21^*University of California in Los Angeles, Los Angeles, California, USA*, ^22^*University of Michigan, University of Michigan Scleroderma Program, Division of Rheumatology Department of Internal Medicine, Ann Arbor, MI, USA*, ^23^*Universitaetsklinikum Koeln, Department of Dermatology, Koeln, Germany*, ^24^*University Hospital of Padua, Haematology and Clinical Immunology Unit and Cardioimmunology outpatient Clinic, Padua, Italy*, ^25^*Serbian Academy of Sciences and Arts, Belgrade, Serbia*

**Introduction:** pSScHI may cause tissue, functional and conduction abnormalities with varied clinical manifestations. pSScHI is poorly defined, impairing the development of specific research projects, and often not distinguished from secondary causes (such as ischaemic heart disease and PAH. We aimed to establish an expert consensus definition for pSScHI to be used in clinical trials and clinical practice.

**Material and Methods:** A SLR for cardiac manifestations and alterations in SSc was conducted using PubMed, Web of Science and Embase. Articles published from inception to December 31st, 2018 were identified. Inclusion criteria included papers in English on adult SSc patients, with heart involvement as outcome. We excluded non-human studies, secondary heart involvement (eg PAH, drugs, infections), reviews and case reports<10 pts. PRISMA recommendations were followed where applicable. The extracted data were categorized into relevant domains (signs, symptoms, anatomical site involved, altered physiological function, pathological changes, prognostic outcomes), which informed the consensus definition. Sixteen senior experts (7 rheumatologists, 8 cardiologists, 1 pathologist) discussed the data and, using a nominal group technique, added expert opinion, provided statements to consider and ranked them. A consensus was attained when agreement was >70%. Seven clinical cases were then evaluated to test for definition’s face validity and feasibility and to allow further refinement.

**Results:** 2593 publications were identified and screened by title and abstract, 251 articles were fully evaluated and 172 met eligibility criteria.

Data from 7 above mentioned domains were extracted and used to develop the World Scleroderma Foundation – Heart Failure Association (WSF-HFA) consensus-derived definition of pSSc-HI, as follows:

“pSScHI comprises cardiac abnormalities that are predominantly attributable to SSc rather than other causes and/or complications*. pSScHI may be sub-clinical and must be confirmed through diagnostic investigation. The pathogenesis of pSScHI comprises one or more of inflammation, fibrosis and vasculopathy. *Non SSc-specific cardiac conditions (e.g. Ischaemic heart disease, arterial hypertension, drug toxicity, other cardiomyopathy, primary valvular disease) and/or SSc non cardiac conditions (e.g. PAH, Renal involvement, ILD).”

Face validity was determined by a 100% agreement on definition credibility, with an inter-observer Kappa (95% CI) of 0.94(0.86-0.99). Application of the definition was feasible, with a median 60 (5-600) seconds time taken per case. Content validity was reached based on the wide variety of patients in the SLR, although to be further validated.

**Conclusions:** Using a SLR and a modified nominal technique, we developed a preliminary pSScHI consensus-based definition and started a validation process. Further validation steps are ongoing, aiming to confirm reproducibility and discrimination.

**Figure:**
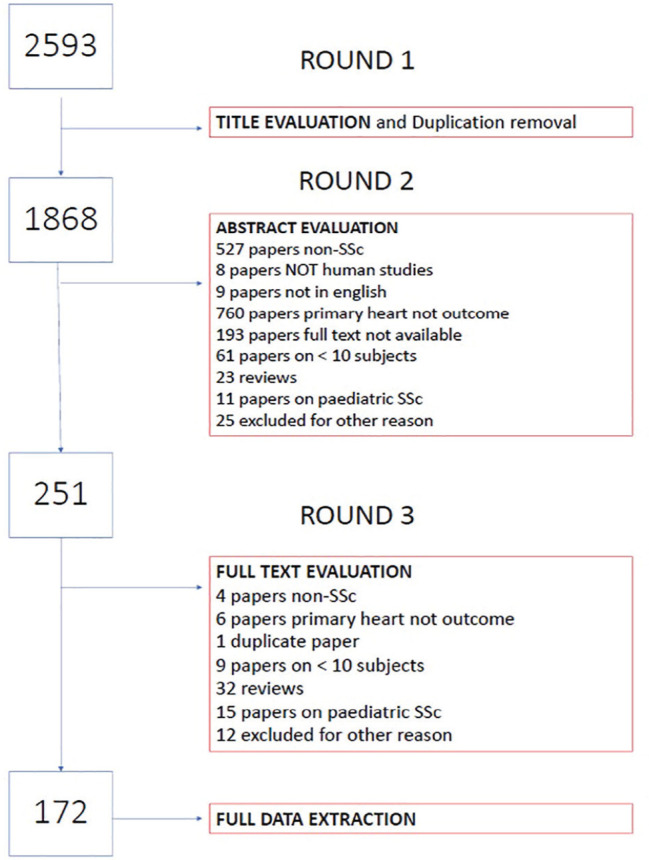


## PARALLEL SESSION 3 - BASIC

## CO.13

## MACHINE-LEARNING CLASSIFICATION IN THE ABATACEPT IN SYSTEMIC SCLEROSIS TRIAL (ASSET) IDENTIFIES A SUBSET OF PATIENTS THAT IMPROVE ON THERAPY VIA MODULATION OF A CD28-RELATED PATHWAY

B. Mehta^1^, J. Franks^1^, M. Espinoza^1^, Y. Yuan^1^, Y. Wang^1^, V. Berrocal^2^, T. Wood^1^, C. Spino^2^, D. Fox^2^, D. Khanna^2^, M. Whitfield^1^

^1^*Geisel School of Medicine at Dartmouth, Lebanon, USA*, ^2^*University of Michigan, Ann Arbor, USA*

**Introduction:** We analyzed a phase 2 study which assessed the efficacy of abatacept in diffuse Systemic Sclerosis (SSc) patients. We test the hypothesis that inflammatory subset patients on abatacept show a significant decline in modified Rodnan Skin Score (mRSS) which is correlated to modulation of pathways related to its mechanism of action.

**Material and Methods:** SSc patients who met 2013 ACR/EULAR criteria were randomized to receive abatacept or placebo for 12 months. RNA-seq was performed on 84 SSc patients at baseline, 3-month, and 6-month timepoints. Samples were assigned to an intrinsic gene expression subset using a Support Vector Machine (SVM) classifier. Linear mixed effect models assessed treatment differences in longitudinal outcomes. Improvement was defined as a 5 point or >20% change in mRSS between baseline and 12 months. A machine-learning approach trained on gene-gene relationships in the skin identified differentially regulated features. Gene Set Enrichment Analysis (GSEA) identified enriched pathways.

**Results:** Patients were assigned to intrinsic subset at baseline (33 inflammatory, 18 fibroproliferative, and 33 normal-like). Differences in trajectory for intrinsic subsets are evident (Figures 1a, 1b). In the abatacept arm, change in mRSS was most pronounced for the inflammatory (p<0.001) and normal-like (p=0.03) subsets when compared to placebo. Three of four patients that experienced disease exacerbation on Abatacept significantly clustered together via K-means clustering of only inflammatory patients, suggesting a unique subgroup (Figure 1c). The gene-gene network returned CD86 as a significant gene that decreased 3 months post-treatment in abatacept improvers despite not being differentially expressed. The pathway Costimulation by the CD28 family decreases (FDR=5.88x10-4) in patients that improve on abatacept, which is specific to the inflammatory subset (FDR=0%). Patients in the inflammatory subset have an elevation in CD28 Costimulation pathway relative to proliferative (p = 0.0026) or normal-like (p=0.0001) patients (Figure 1d). In inflammatory patients, we see a correlation (R=-0.62, p=0.02) between change in mRSS and baseline expression of the Costimulation by the CD28 family pathway (Figure 1e). δ

**Conclusions:** Abatacept shows the most benefit for inflammatory patients. Extent of improvement in inflammatory patients is directly correlated to their baseline expression of the Costimulation by the CD28 family pathway and can predict differential response. Furthermore, we can identify 3 of 4 patients that had severe adverse events by gene expression. These data suggest that stratifying patients by baseline signatures may clarify the effects observed and act as a step towards precision medicine for future clinical practice.

**Figure:**
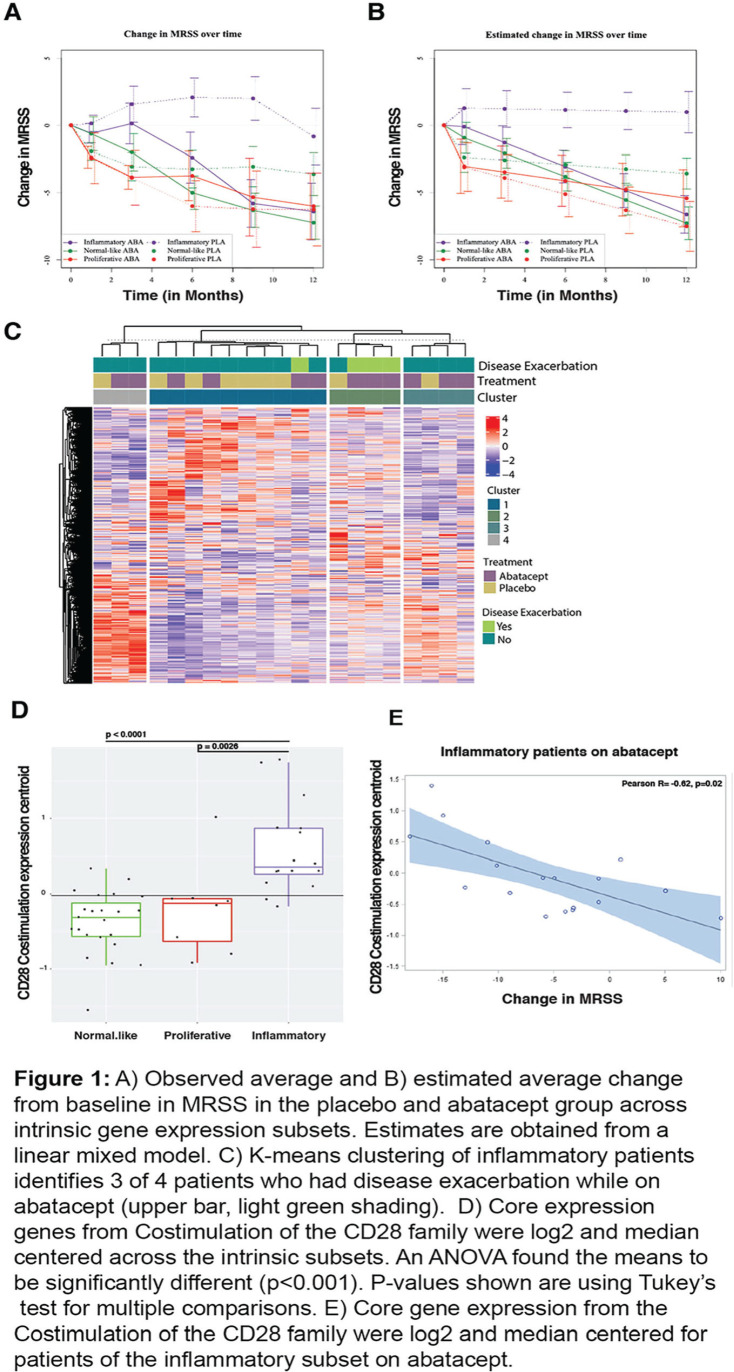


## CO.14

## LARGE-SCALE EXAMINATION OF LONGITUDINAL SKIN GENE EXPRESSION AND ITS ASSOCIATIONS WITH SKIN THICKNESS IN SYSTEMIC SCLEROSIS

B. Skaug^1^, M. Lyons^1^, W.R. Swindell^2^, G.A. Salazar^1^, J. Charles^1^, S. Theodore^1^, M.D. Mayes^1^, S. Assassi^1^

^1^*University of Texas Health Science Center Houston, Division of Rheumatology and Clinical Immunogenetics, Houston, TX, USA*, ^2^*The Jewish Hospital, Department of Internal Medicine, Cincinnati, OH, USA*

**Introduction:** Numerous studies have revealed dysregulated gene expression in the skin of systemic sclerosis (SSc) patients, with varying degrees of inflammatory/immune and fibroblast upregulation. However, the progression of skin gene expression over time and the relationships between skin gene expression and clinical manifestations are incompletely understood. We sought to address these questions through transcriptomic profiling of skin biopsies from a large, diverse group of well-characterized SSc patients, the majority of whom underwent longitudinally-collected biopsies.

**Material and Methods:** 341 total skin biopsies were obtained from the forearm of 113 SSc patients and 45 matched healthy controls. Disease duration was less than six years at initial biopsy (mean of 2.6 years), and 59.3% of patients had diffuse cutaneous involvement. 105 SSc patients underwent a second biopsy, and 77 underwent a third biopsy (mean of 0.8 years and 1.9 years after baseline biopsy, respectively). Biopsy samples were analyzed by microarray, and normalized transcript levels were analyzed for cell type-specific gene expression signatures comprising 15 cell types. Each SSc patient’s cell type signatures were compared to the average amongst healthy controls.

**Results:** 71% of SSc biopsies had upregulation of the fibroblast signature, 58% had upregulation of M1 and M2 macrophage signatures, and 24%, 29%, 36%, and 30% had upregulation of CD4 T cell, CD8 T cell, B cell, and NK cell signatures, respectively. Fibroblast and immune cell signatures correlated with local skin score, with the strongest correlations observed for fibroblast, M1, and M2 macrophage signatures. Fibroblast and immune cell signatures were higher in diffuse compared to limited cutaneous SSc patients, although much of these differences were driven by differences in local skin scores. At baseline biopsy, fibroblast and immune cell signatures correlated with shorter disease duration, particularly amongst diffuse cutaneous SSc patients (Table 1). Longitudinal biopsies revealed that immune cell signatures declined over time, particularly in diffuse cutaneous SSc. Immune cell signatures at baseline biopsy predicted subsequent skin fibrosis course, measured as longitudinal mRSS, based on linear mixed effects modeling. However, the predictive values were not statistically significant after adjustment for baseline mRSS, even when restricting the analysis to diffuse cutaneous SSc patients (Table 2).

**Conclusions:** In summary, immune cell and fibroblast signatures were associated with skin thickness, and immune cell signatures tended to decline over time. Baseline immune cell and fibroblast signatures did not provide predictive significance for the course of skin thickness beyond the information provided by the baseline mRSS.

**Figure:**
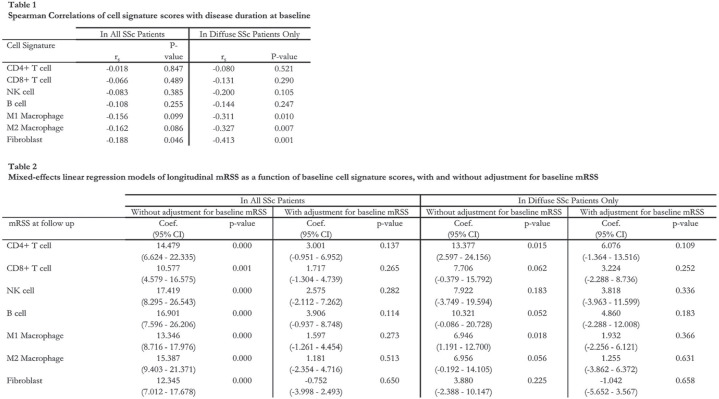


## CO.15

## SERUM INTERFERON SCORE PREDICTS WORSE CLINICAL OUTCOME AT 12 MONTHS IN DIFFUSE SYSTEMIC SCLEROSIS AS MEASURED BY GLOBAL RANKED COMPOSITE SCORE (GRCS) AND ACR COMPOSITE RESPONSE INDEX IN SSC (CRISS)

A. Carriero^1,2^, G. Abignano^1,2,3^, M. Hutchinson^1^, F. Del Galdo^1,3^

^1^*Leeds Institute of Rheumatic and Musculoskeletal Medicine, University of Leeds, Leeds, United Kingdom*, ^2^*Rheumatology Institute of Lucania (IReL), Rheumatology Department of Lucania, San Carlo Hospital, Potenza, Italy*, ^3^*NIHR Leeds Biomedical Research Centre, Leeds Teaching Hospitals NHS Trust, Leeds, United Kingdom*

**Introduction:** Several published studies have shown activation of interferon type 1 (IFN) pathway in Systemic Sclerosis (SSc). Specifically, levels of IFN activation correlate with severity of skin and lung fibrosis, cardiac involvement, quality of life, and measures of collagen turnover. Microarray and proteomic combined studies have indicated that the serum concentration of CCL2, CCL8, CCL19, CXCL9, CXCL10, and CXCL11 are the most relevant serum measures of IFN induced activation of PBMCs.

**Material and Methods:** We averaged the natural logarithm of the serum concentrations of CCL2, CCL8, CCL19, CXCL9, CXCL10, and CXCL11 to calculate IFN score in 143 SSc patients and 35 healthy controls (HC).

Chemokines serum concentration was measured by Luminex xMAP technology. As measure of outcome we calculated the global rank composite score (GRCS) and combined response index in SSc (CRISS) at 12 months, as previously described. GRCS were compared by Mann Whitney test and the effect size for the Mann Whitney test is reported as the percent of all possible pairs between the two groups that favor IFN LO or IFN HI. Fisher exact test was used to analyse patients with CRISS of 0 or >0.

**Results:** All chemokines had a higher serum concentration in SSc vs HC (p<0.0001 for all). Median IFN score was higher in SSc than HC (5.26 vs 4.70, p<0.0001), but within SSc group, there was no difference associated with disease subset or duration. We defined IFN LO or HI patients with a score within or above mean + 2STDV of IFN Score of HCs. Sixty-six 12-month outcome data of dcSSc patients were available for analysis. 37 were IFN HI and 29 IFN LO. GRCS ranged from -59 to 64. IFN HI patients had a worse outcome at 12 months with GRCS median score of -12 vs 15 in IFN LO (p=0.0271). Accordingly, GRCS favored IFN LO in 68.4% of 1073 (37*29) pairwise comparisons versus 31.6% of IFN HI (p=0.0001). 12 month CRISS was >0 in 24% of IFN HI vs 38% of IFN LO (P=0.0464).

**Conclusions:** Serum IFN Score predicts worse clinical outcome at 12 months in dcSSc. Stratification for IFN score could aid both in clinical trial design and clinical management. Moreover, here we show that GRCS and CRISS may be sufficiently sensitive to measure difference in composite outcome at 12 months in dcSSc in an observational setting.

## CO.16

## INTERFERON CHEMOKINE SCORE PREDICTS RESPONSE TO TREATMENT IN SYSTEMIC SCLEROSIS RELATED INTERSTITIAL LUNG DISEASE

S. Assassi^1^, L. Ning^2^, E. Volkmann^3^, M. Mayes^1^, J. Ying^1^, M. Roth^4^, M. Hinchcliff^5^, D. Khanna^6^, T. Frech^7^, P. Clements^3^, D. Furst^3^, J. Goldin^8^, E. Bernstein^9^, F. Castelino^10^, R. Domsic^11^, J. Gordon^12^, F. Hunt^13^, A. Shah^14^, V. Shanmugam^15^, V. Steen^16^, R. Elashoff^2^, D. Tashkin^4^

^1^*The University of Texas Health Science Center at Houston, Division of Rheumatology, Houston, USA*, ^2^*University of California Los Angeles, Department of Biomathematics, Los Angeles, USA*, ^3^*University of California Los Angeles, Division of Rheumatology, Los Angeles, USA*, ^4^*University of California Los Angeles, Division of Pulmonary and Critical Care, Los Angeles, USA*, ^5^*Yale University, Division of Rheumatology, New Haven, USA*, ^6^*University of Michigan Ann Arbor, Division of Rheumatology, Ann Arbor, USA*, ^7^*University of Utah, Division of Rheumatology, Salf Lake City, USA*, ^8^*University of California Los Angeles, Department of Radiological Sciences, Los Angeles, USA*, ^9^*Columbia University, Division of Rheumatology, New York City, USA*, ^10^*Massachusetts General Hospital, Harvard University, Division of Rheumatology, Boston, USA*, ^11^*Pittsburgh University, Division of Rheumatology, Pittsburgh, USA*, ^12^*Hospital for Special Surgery, Division of Rheumatology, New York City, USA*, ^13^*Medical University of South Carolina, Division of Rheumatology, Charleston, USA*, ^14^*Johns Hopkins, Division of Rheumatology, Baltimore, USA*, ^15^*George Washnigton University, Division of Rheumatology, Washington DC, USA*, ^16^*Georgetown University, Washington DC, USA*

**Introduction:** Response to immunosuppression is highly variable in systemic sclerosis (SSc)-related interstitial lung disease (ILD), and there are no widely accepted clinical or biological parameters that predict response to treatment. An up regulation of interferon inducible genes is the most prominent molecular signatures in the SSc peripheral blood cells. Herein, we investigated whether a composite serum Interferon Chemokine Score predicts response to immunosuppression in SSc-ILD.

**Material and Methods:** Serum samples from patients enrolled in the Scleroderma Lung Study II (SLS II) were investigated (n=133). An independent observational cohort of early SSc patients (PRESS cohort) served as the validation cohort. Levels of six interferon inducible chemokines (IP-10, MIG, MCP-2, B2M, MIP-3 beta, TNFR2), in addition to 36 other immune related proteins, were measured using validated multiplex assays. A composite score of the 6 interferon inducible proteins was calculated, and we examined whether the composite score predicted the course of the forced vital capacity (FVC)% predicted over time.

**Results:** In SLS II, the mean baseline FVC% was 66.3% and 77 patients (57.9%) had diffuse cutaneous involvement. Only two serum proteins, MIG and IP-10 (both interferon inducible chemokines) significantly predicted the course of the FVC% in both treatment arms in the same direction after correction for multiple comparisons. Specifically, higher baseline MIG and IP-10 levels predicted an improvement in the FVC% in both treatment arms over 3 to 12 month visits. Similarly, higher baseline Interferon Chemokine Score predicted better response during the 3 to 12 month study visits in the mycophenolate (estimate=0.41, p=0.001) and cyclophosphamide (estimate=0.91, p=0.009) arms. In contrast, higher baseline C-reactive protein levels predicted a decline in the FVC% over the 3 to 12 month in both treatment arms (mycophenolate / cyclophosphamide: estimate = -0.15/-0.56; p=0.038/<0.001).

During the second year, placebo treatment period of the cyclophosphamide arm, higher Interferon Chemokine Score at 12 month showed a trend for predicting worse ILD course from 15 to 24 months (estimate=-0.61, p=0.068), while it continued to predict better response to active immunosuppressive treatment in the mycophenolate arm (estimate=0.28, p=0.029).

The predictive significance of baseline Interferon Chemokine Score was replicated in the independent PRESS cohort in those patients who had ILD and were on active immunosuppressive treatment (n=26, rs=0.43; p=0.028).

**Conclusions:** Higher serum Interferon Chemokine Score in SSc-ILD predicts better response to immunosuppression with mycophenolate or cyclophosphamide and could help identify SSc-ILD patients who may derive the most benefit from these two treatments.

## PARALLEL SESSION 4 - VASCULAR

## CO.17

## PERFORMANCE OF AUTOMATED NAILFOLD CAPILLARY COUNTING SYSTEM (AUTOCAPI) IN SYSTEMIC SCLEROSIS PATIENTS

A. Sulli^1^, A. Vanhaecke^2,3^, C. Pizzorni^1^, V. Tomatis^1^, G. Ferrari^1^, M. Pendolino^1^, V. Smith^2,3,4^, M. Cutolo^1^

^1^*Research Laboratory and Academic Division of Clinical Rheumatology, Department Internal Medicine, University of Genova, Genova, ITALY*, ^2^*Department of Rheumatology, Ghent University Hospital, Ghent, Belgium*, ^3^*Department of Internal Medicine, Ghent University, Ghent, Belgium*, ^4^*Unit for Molecular Immunology and Inflammation, VIB Inflammation Research Center, Ghent, Belgium*

**Introduction:** Loss of nailfold capillaries has been associated with a high risk of developing disease complications in systemic sclerosis (SSc), and can be easily assessed by nailfold videocapillaroscopy (NVC) (1-2). An automated capillary counting system (AUTOCAPI) has recently been validated in SSc patients (3).

Aim of this study was to evaluate the performance of this automated software for absolute nailfold capillary number counting, in SSc patients with different NVC patterns of microangiopathy (Early, Active, and Late).

**Material and Methods:** 183 SSc patients were random collected at both Genova and Ghent Divisions of Rheumatology (LeRoy 2001 or ACR 2013 criteria, mean disease duration 5.5±6.8 years, mean age 55±13 years) and classified by NVC in one the following patterns: 28 Not specific, 37 Early, 89 Active, 29 Late) (4). Eight fingers for each patient were analysed, counting the number of nailfold capillaries manually and by the AUTOCAPI software (DS Medica, Italy) along a millimetre in each finger image (3). The mean capillary number value from the images of the eight fingers was calculated. The software reliability was assessed by calculation of intraclass correlation coefficient (ICC) between automatic and manual counting.

**Results:** The mean number of capillaries assessed by manual vs automatic counting was respectively as follows: 5.23±1.7 vs 5.47±1.3 in the total group of SSc patients, 5.91±1.2 vs 6.87±1.2 in the Not specific, 7.23±1.4 vs 5.67±1.1 in the Early, 4.67±1.1 vs 5.16±1.2 in the Active and 3.72±1.5 vs 4.85±1.1 in the Late pattern of microangiopathy. The highest standard deviation observed for automatic counting was 1.23 in the Not specific group. The following ICC’s were obtained respectively for Total patients, Not specific, Early, Active, and Late NVC patterns: 0.53, 0.51, 0.48, 0.50 and 0.66. The mean values for the manual versus automatic capillary counting assessed by the two centres in all SSc patients were respectively: 5.92±1.8 and 5.02±1.1 for Genova centre, and 4.71±1.5 and 5.83±1.4 for Ghent centre. The automatic counting confirmed that capillary number progressively reduces from Early to Active to Late NVC pattern of microangiopathy.

**Conclusions:** This study demonstrates the good reliability of AUTOCAPI software in nailfold capillary number counting in SSc patients with different patterns of microangiopathy. The use of automated counting software may allow to standardize nailfold capillary assessment among different Rheumatologic centres.

**References**

1. Cutolo M, et al. Arthritis Rheumatol. 2016;68:2527-39.

2. Smith V, et al. J Rheumatol. 2013;40:2023-8.

3. Cutolo M, et al. Microcirculation. 2018;25:e12447.

4. Sulli A, et al, Arthritis Rheum 2012;64:821-5.

## CO.18

## POTENTIAL BENEFICIAL EFFECTS FOR RESVERATROL IN SCLERODERMA VASCULOPATHY

B. Kahaleh, M. Alsehli, D. Gerbec, Y. Wang, S. Nada, N. Altorok

*University of Toledo, Toledo, USA*

**Introduction:** Scleroderma vasculopathy is characterized by enhanced vasospasm, adhesion molecules expression, platelet activation and enhanced vascular wall growth. Resveratrol (RES) is a polyphenol found in more than 70 plants including grapevine and berries. Recent data suggest that RES exerts multifaceted anti-oxidant and/or anti-inflammatory effects in various diseases. Particularly, RES has been reported to induce vasorelaxation and upregulation of NO synthetase, inhibition of platelet aggregation, inhibition of cell proliferation and adhesion molecule expression. In this study we tested the effects of RES on endothelial cells (MVECs), vascular smooth muscle cells (vSMCs) and platelets derived from SSc and control subjects in vitro.

**Material and Methods:** Four lines of MVECs and vSMCs were isolated from involved SSc skin and matched healthy subjects. RES was used at 10, 25, 50 and 100 mM in cell proliferation, migration, angiogenesis and apoptotic assay. Cell proliferation was measured by MTT assay and cellular migration by scratch test. Apoptosis examined by tunnel assay and angiogenesis by matrigel tube formation. Nitric oxide synthase (ENOS) expression level in MVECs was evaluated by western blot.

**Results:** SSc-MVECs displayed significantly higher proliferative capacity (30%) than control cells. RES reduced the proliferative potential by 34% in control cells and by 52% in SSc cells. Similarly, SSc-vSMCs showed significantly higher proliferative capacity than control cells (90%). RES reduced the proliferative rates in a dose dependent fashion in control and normalized the rates in SSc-cells. The migration potential of vSMCs was also significantly higher in SSc cells than control cells and RES significantly reduced the migration rates in both cell types. MVECs apoptosis induced by H2O2 and by growth factors withdrawal was inhibited by RES while vSMCs apoptosis was significantly enhanced by addition of RES, particularly in SSc–MVEC. VEGF induced angiogenesis was significantly inhabited in control-MCECs by RES while it was impaired at baseline in SSc-MVECs and remained so after addition of RES. Finally, the addition of RES enhanced ENOS expression levels in both SSc and control MVEC.

**Conclusions:** The data demonstrate potent anti-proliferative and migration potential for RES in SSc and control MVECs and SMCs. Furthermore, a protective effect on MVEC apoptosis was noted while a potent pro-apoptosis effect was noted in vSMCs. Finally, RES enhanced ENOS production but inhibited angiogenesis particularly in control MVECs. The data suggest potential benefit for RES in SSc vasculopathy. Further mechanistic studies are needed.

## CO.19

## ILOPROST USE AND MANAGEMENT OF SYSTEMIC SCLEROSIS-RELATED VASCULOPATHY IN ITALIAN TERTIARY REFERRAL CENTERS: RESULTS FROM THE PROSIT STUDY

S. Negrini^1^, O. Magnani^1^, M. Matucci Cerinic^2^, R. Carignola^3^, V. Data^3^, E. Montabone^3^, A. Santaniello^4^, G. Adorni^4^, G. Murdaca^1^, F. Puppo^1^, F. Indiveri^1^, A. Della Rossa^5^, A. D Ascanio^5^, S. Barsotti^5^, D. Giuggioli^6^, C. Ferri^6^, F. Lumetti^6^, S.L. Bosello^7^, G. Canestrari^7^, S. Bellando Randone^2^, C. Bruni^2^, S. Guiducci^2^, E. Battaglia^8^, M.I. De Andres^8^, A. Russo^8^, L. Beretta^4^

^1^*Department of Internal Medicine, University of Genoa - Policlinico San Martino, Genoa, Italy*, ^2^*Department of Experimental and Clinical Medicine, AOU Careggi - University of Florence, Florence, Italy*, ^3^*Internal Medicine, San Luigi Gonzaga Hospital Orbassano, Turin, Italy*, ^4^*Fondazione IRCCS Ca Granda, Ospedale Maggiore Policlinico di Milano, Milan, Italy*, ^5^*Rheumatology Unit, Department of Clinical and Experimental Medicine, University of Pisa, Pisa, Italy*, ^6^*Rheumatology Unit, University of Modena and Reggio Emilia, Azienda Ospedaliero Universitaria, Modena, Italy*, ^7^*Rheumatology Unit, Fondazione Policlinico Universitario A. Gemelli IRCCS, Rome, Rome, Italy*, ^8^*Rheumatology Unit, ARNAS Garibaldi, Catania, Italy*

**Introduction:** Vasculopathy has a deep impact on the quality of life of patients affected by systemic sclerosis (SSc) and its management is a challenging issue for the clinician due to the low tolerability of treatments and the lack of consensus on the best therapeutic approach. Iloprost, a synthetic analogue of prostacyclin, is used for the treatment of Raynaud’s phenomenon (RP) and ischemic ulcers secondary to SSc. However, no standardized protocol is currently available and the management of Iloprost treatment is largely based on each single center experience.

**Material and Methods:** The PROSIT project is an observational, multicenter study planned to investigate the current protocols utilized for Iloprost treatment in SSc patients and the perception of the treatment from a patient’s perspective. The study was conducted on a cohort of 346 SSc patients from eight Italian tertiary referral centers and included a structured survey addressed to physicians, data collected from patient’s medical records and two patient-administered questionnaires assessing the perception of the efficacy of Iloprost therapy in terms of level of satisfaction and tolerability.

**Results:** Data obtained confirm that Iloprost represents the first-line treatment for the management of SSc vasculopathy in Italian tertiary referral centers. Concerning infusion length, four centers utilize a 6–8 h infusion, three centers utilize a 4–6-h regimen and one center employs a 8–12 h schedule. The infusion rate ranges from 0.5 to 2.0 ng/kg/min. Iloprost schedule consisted in one to more consecutive days infusions and the intervals between administrations was generally 30 days. During the summer, the treatment was withdrawn in 3/8 centers. The reported number of RP attacks decreased, remained stable and increased in 52% 37% and 11% of the patients, respectively. The majority of patients reported no, or very low, impact on social life.

**Conclusions:** Although a standard protocol is lacking, PROSIT study confirmed that Iloprost is the first line treatment for SSc associated RP and identified several therapeutic approaches supported by tertiary Italian referral centers. Moreover, Iloprost is well-tolerated as reported by patients’ experience. Further studies are needed in order to optimize the best schedule for Iloprost treatment of SSc vasculopathy.

## CO.20

## NAILFOLD CAPILLARY MICROSCOPY HAS LIMITED PROGNOSTIC VALUE IN PREDICTING FUTURE DEVELOPMENT OF CONNECTIVE TISSUE DISEASE IN CHILDREN WITH RAYNAUD’S PHENOMENON

G. Van Der Kamp, A. Van Roon, A. Van Gessel, A. Van Roon, W. Armbrust, U. Mulder

*UMCG, Dept. Internal Medicine, Division Vascular Medicine, Groningen, The Netherlands Antilles*

**Introduction:** Nailfold capillary microscopy (NCM) is a cornerstone in the diagnosis of Systemic Sclerosis (SSc) in adulthood. Although Raynaud’s phenomenon (RP) is very common in childhood, studies on diagnostic methods to differentiate between primary RP (PRP) and secondary RP (SRP) at a young age are scarce. The general aim of this study was to determine the prognostic value of NCM in addition to antinuclear antibodies (ANAs) in children with RP.

**Material and Methods:** This was a case-control study, in which 83 patients diagnosed with RP and having undergone NCM in childhood were retrospectively included. Based on whether they were diagnosed with a connective tissue disease (CTD) during follow-up, they were classified as PRP or SRP. PRP and SRP patients were compared on demographics, NCM and ANA positivity. Variables associated with SRP were included in a multivariate logistic regression model. Predictive values were calculated for NCM, ANA positivity and the combination of NCM and ANA positivity.

**Results:** At the time of the baseline NCM, the mean age of the RP patients was 15.4±2.3 years. Averagely 6.4±3.2 years after the baseline NCM, 65 of the 83 patients were classified as PRP and 18 as SRP. The most common CTDs were MCTD and undifferentiated CTD. ANA positivity was associated with SRP (p<0.001). Of the NCM parameters, only capillary loss was associated with SRP (p=0.01). Abnormal numbers of dilated capillaries, giant capillaries and haemorrhages were not significantly associated with SRP. In a multivariate logistic regression model, only ANA positivity was predictive for SRP (OR 11.19, CI 3.07-40.79). ANA alone had a sensitivity of 66.7% and a specificity of 85.9% for SRP. ANA combined with capillary loss had a sensitivity of 33.3% and a specificity of 96.8%.

**Conclusions:** This study demonstrates that childhood RP is primary in most cases. Whereas RP in adulthood is most strongly associated with SSc, children with RP seem to be at risk of developing other CTDs with less apparent NCM abnormalities. Dilated capillaries, giant capillaries and haemorrhages on NCM are not associated with the spectrum of CTDs that children are at risk for, and do not differentiate between primary and secondary RP. Although capillary loss on NCM is associated with SRP, capillary loss may add little to the predictive value of serology. To clarify which NCM parameters are helpful for early detection of SSc-like CTDs, additional research is required.

## PARALLEL SESSION 5 – CLINICAL

## CO.21

## PROVISIONAL AMERICAN COLLEGE OF RHEUMATOLOGY (ACR) COMBINED RESPONSE INDEX IN DIFFUSE CUTANEOUS SYSTEMIC SCLEROSIS (CRISS) SCORE CORRELATES WITH CHANGES <DELTA> IN PATIENT-REPORTED OUTCOMES (PRO)

R. Spiera^1^, L. Hummers^2^, L. Chung^3^, T. Frech^4^, R. Domsic^5^, V. Hsu^6^, D. Furst^7^, J. Gordon^1^, M. Mayes^8^, R. Simms^9^, E. Lee^10^, S. Constantine^10^, B. Conley^10^, Q. Dinh^10^, B. Bloom^10^, B. White^10^

^1^*Weill Cornell Medical College, New York City, USA*, ^2^*Johns Hopkins University School of Medicine, Baltimore, USA*, ^3^*Stanford University School of Medicine and Palo Alto VA Health Care System, Palo Alto, USA*, ^4^*University of Utah and Salt Lake City VA Health Care System, Salt Lake City, USA*, ^5^*University of Pittsburgh School of Medicine, Pittsburgh, USA*, ^6^*Rutgers Robert Wood Johnson Medical School, New Brunswick, USA*, ^7^*Arthritis Association of Southern California, Los Angeles, USA*, ^8^*University of Texas Health Center at Houston, Houston, USA*, ^9^*Boston University School of Medicine, Boston, USA*, ^10^*Corbus Pharmaceuticals, Inc., Norwood, USA*

**Introduction:** ACR CRISS score and δ modified Rodnan Skin Score (mRSS) have been used as primary or secondary efficacy outcomes in trials assessing overall improvement in diffuse cutaneous systemic sclerosis (SSc). Efficacy outcomes should reflect changes in how the patient feels or functions. We hypothesized that ACR CRISS scores would have higher correlations with δ PRO than would δ mRSS.

**Material and Methods:** ACR CRISS score and change in (δ) mRSS were correlated (Spearman) with δ PRO at Months 3 and 4 in a double-blind placebo-controlled 4-month (Part A) study of lenabasum in SSc (N = 38) and Months 6, 12, 18, and 24 (N = 36, 31, 30, and 24, respectively) in an open-label extension (OLE, Part B). Patient-reported outcomes included Patient Global Assessment of Health related to SSc (PtGA), Health Assessment Questionnaire Disability Index (HAQ-DI), PROMIS-29 domain T-scores, and Systemic Sclerosis Skin Symptoms (SSPRO) score. Baseline was time of the first dose in Part A (Month 3 and 4 in Part A) or the first dose in Part B (OLE Months 6-24). For description, correlations coefficients (r) were categorized as having no (0 to 0.19), weak (0.20 to 0.34), moderate (0.35 to 0.59), or strong (0.60 to 0.79) correlations.

**Results:** All but 1 correlation were directionally correct and nearly all moderate or strong correlations were statistically significant, P <= 0.05. ACR CRISS score had moderate correlations with δ PtGA, δ HAQ-DI, and δ PROMIS-29 social role and pain interference scores at Months 3 or 4 in Part A. ACR CRISS score had moderate to strong correlations with changes in patient function (HAQ-DI, PROMIS-29 physical function and social role domains) throughout Part B. It had weak to moderate correlations with δ PtGA, δSSPRO, and δ PROMIS-29 pain interference in Part B. In contrast, correlation coefficients of δ mRSS with δPRO were >= 0.30 for all correlations at all times, except for δ PROMIS-29 physical function score at 6, 12, and 24 months in the OLE (r = 0.33 to 0.37).

**Conclusions:** ACR CRISS score consistently correlated with changes in measures of how the patient feels and functions including those measures not captured in ACR CRISS score calculation, whereas δ mRSS did not. Based on these findings, ACR CRISS score may be a more relevant choice as a primary efficacy outcome than δ mRSS in improvement trials in SSc.

**Figure:**
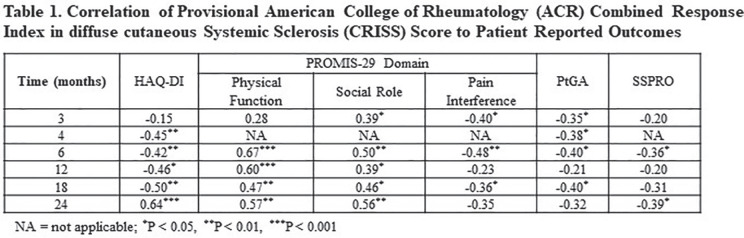


## CO.22

## THE EFFECT OF GENDER ON OUTCOME IN SYSTEMIC SCLEROSIS (SSC): DOES ANTI-TOPOISOMERASE I AUTOANTIBODY (ATA) MATTER?

M. Boonstra^1^, S. le Cessie^2^, A. Riccardi^3^, P. Airo^4^, O. Distler^5^, M. Matucci-Cerinic^6^, C. Caimmi^7^, Y. Allanore^8^, T. Huizinga^1^, R. Toes^1^, H. Scherer^1^, J. de Vries-Bouwstra^1^, A. EUSTAR co-authors^1^

^1^*Leiden University Medical Center, Department of Rheumatology, Leiden, The Netherlands*, ^2^*Leiden University Medical Center, Department of Clinical Epidemiology and Department of Biomedical Data Sciences, Leiden, The Netherlands*, ^3^*Dipartimento Medicina Clinica e Sperimentale, F-Magrassi, II Policlinico U.O. Reumatologia, Napoli, Italy*, ^4^*UOC Reumatologia e Immunologia Clinica, Spedali Civili, Brescia, Italy*, ^5^*University Hospital Zurich, Department of Rheumatology, Zurich, Switzerland*, ^6^*University of Florence, Department of Experimental and Clinical Medicine, Florence, Italy*, ^7^*University of Verona, Rheumatology Unit, Verona, Italy*, ^8^*Paris Descartes University, Cochin Hospital, Department of Rheumatology, Paris, France*

**Introduction:** Male gender and anti-topoisomerase I autoantibodies (ATA) have both been associated with unfavourable outcomes in Systemic Sclerosis (SSc). Prevalence of SSc autoantibodies also differs with sex, with males more often being ATA+. We present an in-depth study on the effect of sex on mortality, diffuse skin involvement (dcSSc), Interstitial lung disease (ILD) and Pulmonary Hypertension (PH) in SSc, not explained by auto-antibody status.

**Material and Methods:** Using Kaplan Meier curves and Cox proportional hazard models, with correction for left truncation due to various disease durations at cohort entrance, we evaluated the effect of sex (corrected for auto-antibody status, age and race) on all-cause mortality in SSc in two cohorts: 1. the Leiden Combined Care In SSc cohort (CCISS; n=242) and 2. the EULAR Scleroderma Trial and Research (EUSTAR) cohort (n=4263). In addition, we profited from the large sample size of the EUSTAR cohort to perform multivariate analysis and evaluate the effect of sex on the occurrence of diffuse skin involvement (dcSSc), interstitial lung disease (ILD) and pulmonary hypertension (PH), after adjustment for auto-antibody status, age and race.

**Results:** Both cohorts showed that SSc males more often express ATA than SSc females (CCISS: 40% of males vs 21% of females; EUSTAR: 49% of males vs 38% of females). In the multivariate analysis based on the EUSTAR cohort male sex was associated with mortality (HR 2.6 [95% CI 2.0-3.4]). This effect was stronger than the effect of ATA (HR 1.33 [95% CI 1.0-1.8]). Male sex was also independently associated with development of dcSSc (HR 1.4 [95%CI 1.1-1.8]) and PH (HR 1.5 [95%CI 1.2-2.0]). Only for ILD the effect of ATA (HR 1.9 [95%CI 1.5-2.5]) was stronger than the effect of sex (HR 1.1 [95%CI 0.9-1.3]). See Figure 1 for Kaplan Meier curves.

**Conclusions:** Male sex is strongly associated with mortality in SSc. This association cannot be explained by higher prevalence of ATA among males. Also dcSSc and PH, but not ILD develop more often in male patients independent of auto-antibody status. Hence, our data highlight an interesting effect of gender on disease outcome in SSc which may need to be taken into account for future risk stratification.

**Figure:**
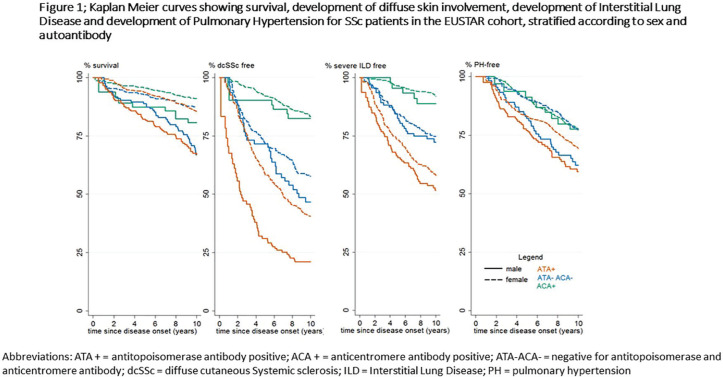


## CO.23

## THE PATIENT JOURNEY OF SSC-ILD IN EUROPE: AN INTEGRATED ANALYSIS OF HEALTHCARE PROFESSIONAL AND PATIENT PERSPECTIVES

A. Hoffmann-Vold^1^, E. Bendstrup^2^, M.C. Vonk^3^, W.A. Wuyts^4^

^1^*Oslo University Hospital, Department of Rheumatology, Oslo, Norway*, ^2^*Radboud University Medical Center, Department of Rheumatology, Nijmegen, The Netherlands*, ^3^*Aarhus University Hospital, Center for Rare Lung Diseases, Department of Respiratory Disease and Allergy, Aarhus, Denmark*, ^4^*University Hospitals Leuven, Department of Pulmonology, Leuven, Belgium*

**Introduction:** Interstitial Lung Disease is frequent in Systemic Sclerosis (SSc-ILD) and associates with high morbidity and mortality. Here we aimed to analyse patient pathways, experiences and needs of SSc-ILD patients and healthcare professionals (HCP) in order to identify gaps in the care and management of SSc-ILD patients.

**Material and Methods:** Semi-structured qualitative interviews were conducted in 8 European countries (Belgium, Denmark, Finland, Greece, Netherlands, Norway, Portugal and Sweden); 99 HCP from several disciplines (rheumatologists, pulmonologists, dermatologists) and nurses, and 49 patients, patient organisation representatives and caregivers participated. Two sets of 70 research questions were used to guide individual, in-depth face-to-face or telephone interviews. The following three phases of the patient pathway were explored: (1) pre-diagnosis (first symptoms, first line consultation, and referral); (2) diagnosis (tests, consultations); (3) post-diagnosis (consultations, treatment, quality of life, patient support).

**Results:** (1) The main gaps in the pre-diagnostic phase, identified by HCP and patients, were common to all participating countries: lack of disease awareness among primary care physicians and specialists, lack of accurate information for patients, and delayed and/or inappropriate referral.

(2) Regarding the diagnostic phase, most commonly, rheumatologists coordinate the diagnostic work-up and are the main point of care but in some countries other specialists (e.g. dermatologists, internists, pulmonologists) are also involved. Depending on the local health system, the organisation of multidisciplinary collaboration is varying. HCP issued the lack of national guidelines, except for the Dutch SSc pathway of care, while patients found it difficult to understand and remember the disease-related information that was given during this diagnostic phase.

(3) In the post-diagnostic phase, HCP and patients indicated lack of curative treatment, specialized nurses and paramedical and psychological support. Patients and caregivers additionally expressed the need for clear information on SSc-ILD.

**Conclusions:** Lack of disease awareness, gaps in national healthcare systems and insufficient support for patients and caregivers were identified as the main bottlenecks to overcome in order to improve quality of care and hopefully ensure timely diagnosis, provide better patient management and increase quality of life in SSc-ILD patients.

## CO.24

## THE TIMELINESS AND TYPES OF CANCER IN SYSTEMIC SCLEROSIS

K. Gunnarsson^1^, R. Hesselstrand^2^, M. Holmqvist^3^

^1^*Karolinska Institute, Department of Medicine Solna, Stockholm, Sweden*, ^2^*Skåne University Hospital, Department of Clinical Sciences Lund, Lund, Sweden*, ^3^*Karolinska Institute, Clinical Epidemiology Division, Department of Medicine Solna, Stockholm, Sweden*

**Introduction:** Systemic sclerosis (SSc) has the highest short-term mortality of all connective tissue diseases; the standardized mortality ratio has been estimated to around 3.5. The increased mortality is partly related to SSc-related organ manifestations, such as interstitial lung disease and pulmonary arterial hypertension. In recent years, cardiovascular disease and malignancies, diseases not typically associated with SSc, have been described as important contributing factors in the increased mortality. However, there are still conflicting data concerning the timing of cancer in SSc, and what types of cancers that are most common. Therefore, we wanted to investigate the association between cancer and SSc in a population-based study of Swedish patients with SSc and individuals from the general population.

**Material and Methods:** We identified all patients diagnosed with SSc (n=1091) between 2002 and 2016, using ICD codes in the outpatient register. Patients were matched to individuals from the general population (n=5352) on sex, age, and residential area. We linked our study population to the Swedish Cancer Register. The odds ratios (OR) of any first primary cancers before SSc diagnosis were estimated using logistic regression. The hazard ratios (HR) of any first primary cancers after SSc diagnosis were estimated by Cox proportional hazards model.

**Results:** In our study population, 78% of all individuals were women, mean age at diagnosis was 59. The overall OR for having any primary cancer before SSc diagnosis was 1.2 (95% CI 1.0-1.4) compared to the general population. The association was only seen in men, OR 2.3 (95% CI 1.5-3.4) vs 1.1 in women. The total follow up after diagnosis was 4746 person years in SSc, and 28393 in the general population. The HR for having any primary cancer after SSc diagnosis was 1.9 (95% CI 1.5-2.4). HR in women was 1.6 (95% CI 1.2-2.2) whereas in men 2.7 (95% CI 1.7-4.2). In cancer discovered before SSc diagnosis, GI cancers (excluding colorectal, buccal and larynx cancer), lung cancer, and non-melanoma skin cancer were more common in SSc than in the general population (see table for all estimates of site-specific cancers). In cancers detected after SSc diagnosis the risk for skin cancer was elevated, especially non-melanoma skin cancer.

**Conclusions:** These results confirm the increased occurrence of cancer in SSc patients both before and after diagnosis and adds information about what types of cancers are most prevalent.

**Figure:**
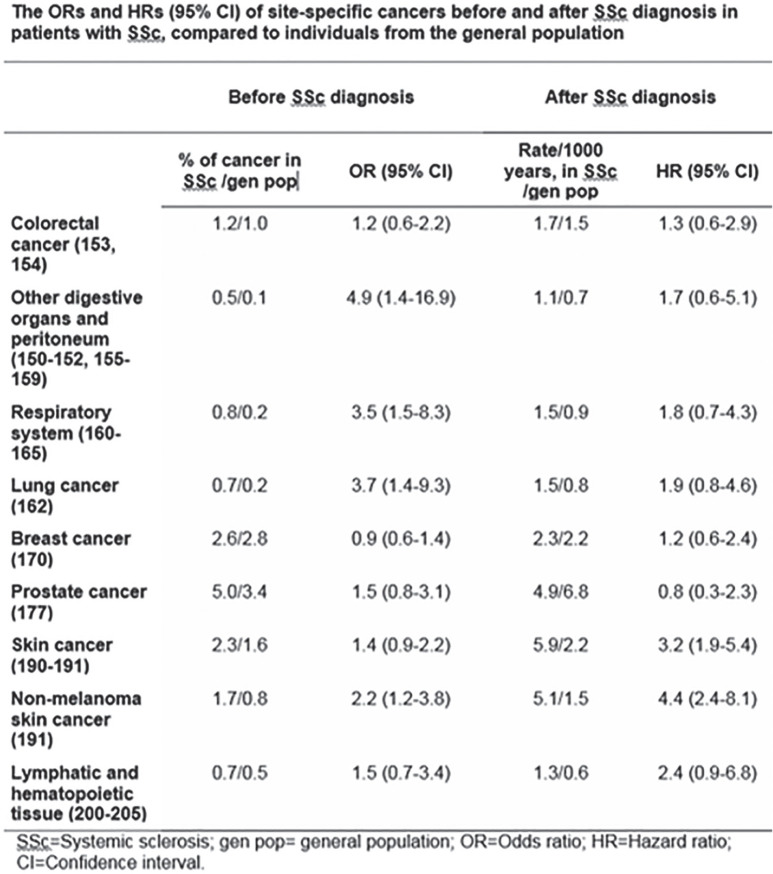


## CO.25

## THE RELATIONSHIP BETWEEN GASTROINTESTINAL SEVERITY AND SYMPTOMS AND WHOLE GUT TRANSIT IN PATIENTS WITH SYSTEMIC SCLEROSIS

Z. McMahan^1^, A. Tucker^2^, J. Perin^3^, E. Volkmann^4^, H. Zeissman^5^, A. Rosen^1^, J. Pasricha^6^, F. Wigley^1^

^1^*Johns Hopkins Department of Internal Medicine, Division of Rheumatology, Baltimore, USA*, ^2^*Johns Hopkins Department of Internal Medicine, Baltimore, USA*, ^3^*Johns Hopkins Department of Global Disease Epiemiology and Control, Baltimore, USA*, ^4^*University of California- Los Angeles, Division of Rheumatology, Los Angeles, USA*, ^5^*Johns Hopkins Department of Radiology, Baltimore, USA*, ^6^*Johns Hopkins Department of Internal Medicine, Division of Gastroenterology, Baltimore, USA*

**Introduction:** Scleroderma (SSc)-associated gastrointestinal (GI) disease is associated with various complications. A number of factors may contribute to GI symptoms and severity including factors such as diet, dysbiosis, or abnormalities in GI transit. We sought to examine whether abnormal GI transit contributes to GI severity and symptoms in SSc.

**Material and Methods:** Methods: Patients with SSc and GI symptoms were recruited consecutively from clinic in the Johns Hopkins Scleroderma Center. Patients underwent a nuclear medicine-based whole gut transit (WGT) study, which assesses transit from the esophagus to the colon. The Medsger GI severity score was used to examine the relationship between physician-determined GI severity and specific regions of dysfunction. The UCLA GIT 2.0 scale was used to determine whether patient-reported GI symptoms associate with GI transit severity.

**Results:** pending final

**Conclusions:** GI involvement in SSc often affects several regions of the GI tract simultaneously, suggesting a more comprehensive GI assessment is warranted. In SSc, the severity of GI transit is associated with more extensive GI involvement, while the presence of dysfunction in distinct anatomic regions in the gut is associated with specific GI complications. This suggests that physician-reported GI severity scales may play a role in determining regional involvement in SSc patients with severe disease, when WGT studies are not feasible.

## CO.26

## VALIDATION OF DISEASE ACTIVITY MEASURES IN PATIENTS WITH JSSC – RESULTS FROM THE JSSC INCEPTION COHORT

J. Klotsche^2^, I. Foeldvari^1^, O. Kasapcopur^3^, A. Adrovic^3^, K. Torok^3^, V. Stanevicha^3^, M.T. Terreri^3^, F. Sztajnbok^3^, E. Alexeeva^3^, J. Anton^3^, B. Feldman^3^, M. Katsikas^3^, V. Smith^3^, T. Avcin^3^, R. Cimaz^3^, M. Kostik^3^, T. Lehman^3^, W.-. Sifuentes-Giraldo^3^, N. Vasquez-Canizares^3^, N. Helmus^1^

^1^*Hamburg Centre for Pediatric and Adolescence Rheumatology, Hamburg, Germany*, ^2^*German Rheumatism Research Center, Berlin, Germany*, ^3^*jSSc Collaborative Group, Hamburg, Germany*

**Introduction:** Juvenile systemic sclerosis (jSSc) is a rare disease in childhood. Disease severity captures the impact of jSSc on organ function as a function of disease activity and damage. It does not exist a gold standard to describe disease activity in patients with jSSc by lack of a definition of flare and remission. A routinely used measure of disease activity is the physician’s global assessment (PGA) of activity based on a visual analogue scale (VAS) with remarkable interrater variability. Previously, La Torre proposed a composite score for disease activity (LaTorreDAi) in adult patients with systemic sclerosis (SSc). In addition, the Valentini disease activity index (VDAi) and revised EUSTAR index (rEUSTARi) provide a measure for adult SSc patients. The objective of this study was to validate the disease activity measures in a prospectively followed cohort of patients with jSSc.

**Material and Methods:** Data from the prospective international inceptions cohort for jSSc was used for the validation. The three composite measures for disease activity were calculated according to the scoring algorithms. The mRSS (modified Rodnan skin score) and type of jSSc were used to assess concurrent and discriminative reliability. Sensitivity to change was assessed in patients who improved in PGA during the 12-months follow-up.

**Results:** A total of 128 jSSc patients have been enrolled in the inception cohort. 70% of the patients had diffuse subtype, the mean age at enrollment was 12.9 (3.9) and the mean age at Raynaud onset 9.9 (4.2). The PGA (r=0.67), rEUSTARi (r=0.66), VDAi (r=0.59) and LaTorreDAi (r=0.58) showed a good concurrent validity with the modified Rodman skin score (MRSC). Discriminative validity was assessed between the jSSc subtypes. The mean PGA, rEUSTARi and VDAi significantly differed between patients with limited and diffuse jSSc subtype, however, the mean LaTorreDAi was comparable between both subtypes. The VDAi showed a good sensitivity to change between baseline and 12-month follow-up (d=0.64). A moderate sensitivity to change was observed for the rEUSTARi (d=0.37), whereas it was low for the LaTorreDAi (d=0.19).

**Conclusions:** The VDAi, rEUSTARi and PGA showed a good congruent validity with the MRSC and may be feasible as disease activity measures in pediatric SSc patients. The LaTorreDAi seems to present more damage rather than disease activity because of its low sensitivity to change.

## PARALLEL SESSION 6 – BASIC

## CO.27

## FGFR3/ FGF9 SIGNALING REGULATES A NETWORK OF PROFIBROTIC CYTOKINE AND GROWTH FACTOR PATHWAYS TO DRIVE FIBROBLAST ACTIVATION AND TISSUE FIBROSIS IN SYSTEMIC SCLEROSIS

D. Chakraborty^1^, H. Zhu^1^, A.-E. Matei^1^, T. Trinh-Minh^1^, C.-W. Chen^1^, A. Soare^1^, A. Ramming^1^, G. Schett^1^, O. Distler^2^, J. HW Distler^1^

^1^*Department of Internal Medicine 3- Rheumatology and Immunology, Friedrich-Alexander University (FAU) Erlangen-Nürnberg, Erlangen, Germany*, ^2^*Center of Experimental Rheumatology and Zurich Center of Integrative Human Physiology, University Hospital Zurich, Switz, Zurich, Switzerland*

**Introduction:** Fibroblast growth factor receptor 3 (FGFR3) is a member of the family of 4 different fibroblast growth factor receptors (FGFR1-4) with 22 identified fibroblast growth factors ligands (FGF1-22) in humans. Each FGFR has different isoforms resulting from alternative splice variants, which can bind specific FGF ligands and trigger various intracellular signaling pathways to regulate important biological processes. In this study, we elucidate specific upregulation of FGFR3 and its ligand FGF9 in SSc, which further induce skin fibrosis mediated by a network of profibrotic pathways.

**Material and Methods:** Differential expression profiling of dermal cells from SSc patients and healthy volunteers were performed employing microarrays. Further expression analyses were done in skin tissues and fibroblasts from SSc patients and in experimental models of SSc by real-time PCR, Western Blot, immunohistochemistry and immunofluorescence analysis. Selective inhibitors in conjunction with knockdown and knockout strategies were used to target FGF9/FGFR3 signaling and its downstream mediators in vitro and in vivo. Recombinant FGF9 was used to study the profibrotic effects of FGF9/FGFR3 upregulation in human dermal fibroblasts and in three-dimensional full thickness skin organoids. Genetic and pharmaceutical inactivation of FGF9/FGFR3 signaling was evaluated in different mouse models of SSc: skin fibrosis models induced by bleomycin and by constitutively active transforming growth factor receptor 1 (TBR) and dermal fibrosis in tight skin (TSK) mice.

**Results:** FGFR3, specifically the isoform FGFR3IIIb and its ligand FGF9 expression was significantly upregulated in the dermis and dermal fibroblasts in SSc. Furthermore, this increase of FGFR3IIIb/FGF9 expression was found to be upregulated by TGFB signaling in dermal fibroblasts in a SMAD3-dependent mechanism.

Microarray profiling, in silico analyses along with functional experiments demonstrate that FGFR3 induces the activation of ERK, AKT, CAMK2 and P38 kinases to induce CREB-dependent upregulation of multiple profibrotic pathways, including Endothelin-, Interleukin4-and CTGF-signaling. Several core meditors of fibrosis, including CTGF, EDN1 and its recptor EDNRB, IL4R and CCl2 have been identified as FGFR3 targets. FGF9 stimulated FGFR3 upregulation was sufficient to drive resting fibroblast-to-myofibroblast differentiation along with increased collagen secretion and aSMA production. Fibroblast-specific knockout of FGFR3 or FGF9 or pharmacological inhibition of FGFR3 abrogates myofibroblast differentiation in vitro and ameliorates experimental skin fibrosis in the animal models of SSc.

**Conclusions:** Considering that several FGFR inhibitors are in clinical trials, FGFR3 is a promising candidate for antifibrotic treatments as we have identified and characterized FGFR3 signaling as a novel, yet major upstream regulator of several profibrotic pathways in SSc.

## CO.28

## A NOVEL HUMANIZED MODEL FOR SYSTEMIC SCLEROSIS BY TRANSFERRING PBMC FROM PATIENTS INTO IMMUNODEFICIENT MICE

X. Yue^1^, Y. Shu^1^, F. Petersen^1^, G. Riemekasten^2^, X. Yu^1^

^1^*Research Center Borstel, Borstel, Germany*, ^2^*University of Lübeck, Lübeck, Germany*

**Introduction:** Systemic sclerosis (SSc) is a complex autoimmune connective tissue disease with an unclear pathogenesis. Although SSc is investigated in many experimental systems in vitro and in vivo, a humanized mouse model mimicking most closely the disease in humans is still lacking. In the current study, we aimed to close this gap by establishing a novel model for SSc based on transfer of patient cells to mice.

**Material and Methods:** To generate a humanized mouse model for SSc, PBMC from healthy subjects or patients were isolated and transferred into Rag2-/-/Il2R-/- mice. The recipient mice were sacrificed at week 12 after transfer, and blood, spleen, lung, kidney, heart, liver, muscle, esphogus, intestines as well as skin were collected for further evaluation. Serum levels of total human IgG, anti-angiotensin-II type1 receptor (AT1R) IgG and anti-endothelin-1 type A receptor (ETAR) IgG in recipient mice were determined by ELISA. Histopathology of murine organs was evaluated by hematoxylin and eosin (H&E) as well as immunohistochemstry (IHC) staining. To investigate the role of T and B cells in the pathogenesis of the humanized mouse model, human CD3+ T or CD19+ B cells were depleted from PBMC using a magnetic activated cell sorter (MACS) before the transfer.

**Results:** Twelve weeks after transfer of PBMC from SSc patients, the recipient mice generated human autoantibodies against AT1R and endothelin receptor type A (ETAR). Furthermore, these mice developed severe inflammatory infiltrates dominated by B cells in multiple organs such as lung, kidney and liver. By contrast, mice which received PBMC from healthy subjects produced significantly lower levels of anti-AT1R and anti-ETAR autoantibodies, than mice received PBMC from SSc patients although both groups of mice generated comparable levels of total human IgG. We also found that transfer of PBMC from healthy subjects did not induce obvious inflammation in any organs of recipient mice. Furthermore, neither autoantibodies nor tissue inflammation was detected in mice which received PBMC from SSc patients treated with rituximab, suggesting a role of B cells in the development of disease. Depletion of T or B cells from PBMC before the transfer demonstrated that both T and B cells were indispensable for the development of the disease.

**Conclusions:** Our results demonstrate that transfer of PBMC derived from SSc patient induces SSc-like symptoms in mice. Moreover, the identification of human lymphocytes as drivers of the disease in the humanized model used here argues strongly for the presence of a corresponding mechanism in SSc in humans.

## CO.29

## FLI1-DEFICIENT ADIPOCYTES PROMOTE SPONTANEOUS SKIN FIBROSIS AND VASCULOPATHY: POTENTIAL ROLES OF ADIPOCYTES IN SYSTEMIC SCLEROSIS

T. Miyagawa^1^, R. Saigusa^1^, T. Yamashita^1^, M. Hirabayashi^1^, K. Nakamura^1^, S. Miura^1^, T. Taniguchi^1^, A. Yoshizaki^1^, M. Trojanowska^2^, S. Sato^1^, Y. Asano^1^

^1^*The University of Tokyo Graduate School of Medicine, Tokyo, Japan*, ^2^*Boston University School of Medicine, Boston, USA*

**Introduction:** Systemic sclerosis (SSc) is a multisystem autoimmune disease characterized by vasculopathy and extensive organ fibrosis. Although its pathogenesis still remains unknown, adipokines have been suggested to be involved in the development of these pathological features of SSc. So far, our studies have demonstrated that the deficiency of transcription factor Fli1 induces SSc-like phenotypes in various cells like dermal fibroblasts, endothelial cells, macrophages and epithelial cells.

**Material and Methods:** We generated adipocyte-specific *Fli1* knockout (*Fli1* AdipoKO) mice (*Fli1*^flox/flox^;*Adipo*-Cre) and investigated whether these mice recapitulate the cardinal features of SSc.

**Results:** *Fli1* AdipoKO mice spontaneously developed dermal fibrosis at the age of 3 months. The skin of these mice demonstrated higher levels of total collagen content and myofibroblast counts. In double immunoflorescence for α-smooth muscle actin and perilipin, double positive fusiform cells were evident throughout the deep dermis in these mice, suggesting the promotion of adipocyte-to-myofibroblast transdifferentiation. As for vascular involvements, *Fli1* AdipoKO mice exhibited vascular structural and functional abnormalities, such as capillary dilation, arteriolar stenosis and increased vascular permeability, as early as 3 months of age. To assess genesis of vasculature, we examined bone marrow-derived mesenchymal stem cells (BM-MSCs). Strikingly, BM-MSCs exhibited de-differentiated phenotype characterized by α-smooth muscle action downregulation and Rgs5 up-regulation, suggesting the contribution of defective vasculogenesis to the development of vascular abnormalities. This phenotype was induced by co-culture of Fli1-deficient adipocytes and BM-MSCs of wild type mice.

**Conclusions:** These results indicate that Fli1-deficient adipocytes can be involved in the development of dermal fibrosis and vasculopathy recapitulating SSc, suggesting a potential contribution of phenotypically altered adipocytes to the development of SSc.

## CO.30

## ADENO-ASSOCIATED VIRUS-5 TARGETS PLATELET-DERIVED GROWTH FACTOR RECEPTOR ALPHA IN LUNG AND PERIPHERAL BLOOD OF PATIENTS WITH SYSTEMIC SCLEROSIS AND ELICITS STIMULATORY ANTIBODIES

G. Moroncini^1^, A. Grieco^1^, S. Svegliati^1^, T. Spadoni^1^, C. Paolini^1^, C. Tonnini^1^, S. Agarbati^1^, M. Cuccioloni^2^, M. Mozzicafreddo^2^, A. Funaro^3^, D. Benfaremo^1^, P. Dorfmuller^4^, D. Amico^5^, J. Kleinschmidt^6^, K. Nieto^6^, Q. Chen^6^, M. Muller^6^, E. Avvedimento^7^, A. Gabrielli^1^

^1^*Clinica Medica, Dipartimento di Scienze Cliniche e molecolari Università Politecnica delle Marche, Ancona, Italy*, ^2^*Scuola di BioScienze e Medicina veterinaria, Università di Camerino, Camerino, Italy*, ^3^*Dipartimento di Scienze mediche, Università di Torino, Torino, Italy*, ^4^*Department of Pathology, University Hospital of Giessen and Marburg, Giessen, Germany*, ^5^*Divisione di Pneumologia, Ospedali Riuniti Marche nord, Pesaro, Italy*, ^6^*German Cancer Research Center, Heidelberg, Germany*, ^7^*Dipartimento di medicina Molecolare e Biotecnologie Mediche, Università Federico II, Napoli, Italy*

**Introduction:** SSc patients contain stimulating antibodies targeting Platelet-derived growth factor receptor alpha (PDGFRalpha), which represents the main entry site for the adeno-associated virus type 5 (AAV5). We investigated the presence of AAV5 DNA in the lung and peripheral blood of SSc patients, and its relationship with anti-PDGFRalpha stimulating antibodies

**Material and Methods:** In silico molecular docking was performed to predict the binding between the three-dimensional structures of monomeric human PDGFRalpha and AAV5 capsid monomeric subunit. A surface plasmon resonance (SPR) assay was performed to validate in vitro the in silico prediction of the possible molecular complex between AAV5 and PDGFRalpha. The PDGFRalphaKO (by CRISP-CAS9 technology) human alveolar basal epithelial cell line A549, was used to assess the role of PDGFRalpha for AAV5 transduction. Bronchoalveolar lavage of 66 SSc patients and 77 controls affected by conditions other than SSc was analyzed by PCR for the presence of AAV5 DNA. The presence of the virus in the lung was also assessed by in situ hybridization, immunohistochemistry and confocal microscopy to demonstrate colocalization of PDGFRalpha and AAV5. Molecular docking, SPR, and immunoprecipitation studies, were performed to demonstrate the binding of anti-PDGFRalpha antibodies to AAV5.

**Results:** AAV5 in silico interacts with the extracellular domains of PDGFRalpha and SPR assay showed that the AVV5 capsid monomer binds PDGFRalpha with high affinity. Deletion of PDGFRalpha by CRISP-CAS9 in A549 cells inhibits significantly the transduction efficiency. AAV5 genomic sequences were found in 71.2% of SSc patients and in 28.5% of controls (p<0.0001). AAV5 was present in alveolar epithelial cells by immunohistochemistry and in situ hybridization and co-localized with PDGFRalpha by confocal microscopy. Both total SSc-IgG and human monoclonal anti-PDGFRalpha antibodies (Moroncini et al 2015) immunoprecipitated PDGFRalpha and AAV5 capsid from infected cells. Specific PDGFRalpha and AAV5 peptides recognize the monoclonal anti-PDGFRalpha antibodies.

**Conclusions:** The present study demonstrates that AAV5 is present in the lung and blood in a significant fraction of SSc patients, and recognizes stimulatory anti-PDGFRalpha antibodies. AAV5 contributes to the pathogenesis of systemic sclerosis by eliciting adaptive immunity targeting the PDGFRalpha complex.

## CO.31

## IMMUNE MECHANISMS OF DISORDER IN THE WORK OF THE BODY’S ANTIOXIDANT SYSTEM IN THE PATHOGENESIS OF LUNG DAMAGE IN SYSTEMIC SCLERODERMA

L. Shilova^1^, N. Aleksandrova^2^, I. Alekhina^3^, A. Aleksandrov^1,2^

^1^*Volgograd State Medical University, Department of Hospital Therapy, Volgograd, Russia*, ^2^*Research Institute for clinical and experimental rheumatology named after A.B. Zborovsky, Volgograd, Russia*, ^3^*Stavropol State Medical University, Department of Hospital Therapy, Stavropol, Russia*

**Introduction:** Oxidative stress plays an important role in the pathogenesis of lung injury and pulmonary fibrosis in patients with systemic scleroderma (SSc), which is considered to be potentially the most serious visceral lesions in this disease.

Objective: to study the clinical and laboratory features of lung disease and the role of antioxidant enzymes in the development of organ pathology in SSc.

**Material and Methods:** The study included 83 patients with a reliable diagnosis of SSc (97.6% of women, mean age 50.3±11.9, disease duration 8.3±7.1 years) and 30 healthy individuals. The initial stage of the disease was detected in 13.3% of cases, the developed one - in 75.9%, the terminal - in 10.8%.

The patients were assigned to clinical, laboratory, functional and instrumental research methods with the obligatory radiography and/or computer tomography of the chest organs and assessment of the function of external respiration.

Antibodies of the IgG class to antioxidant enzymes (glutathione peroxidase, glutathione reductase, superoxide dismutase) were determined in blood serum by the standard ELISA test using an immobilized antigenic form of the corresponding enzyme.

**Results:** Lung damage was observed in 66 (79.5%) patients with SSc and was mainly represented by interstitial pulmonary fibrosis (mainly the basal sections) - 60% of cases and pulmonary hypertension (according to echocardiography) - 6% of cases. Restrictive breathing disorders (reduced forced lung capacity below 80% of the proper values) were noted in 38 people (57.6%). Imbalance of the oxidative-antioxidant was observed in the early stages of the disease along with inflammation, disruption of the immune system (overproduction of various autoantibodies) and the development of vascular abnormalities with the subsequent spread of fibrosis.

The level of all antibodies studied in SSc was increased in comparison with healthy individuals (p<0.005). A significant decrease in the activity of glutathione peroxidase (p=0.008) and superoxide dismutase (p=0.042), an increase in the level of antibodies to glutathione reductase (p=0.04) and glutathione peroxidase (p=0.037) were observed in patients with respiratory failure with lung damage. Also in the group of patients positive for antibodies to superoxide dismutase the signs of lung damage were found statistically significantly more often (χ² with Yates correction = 3.47, p=0.048).

**Conclusions:** Glutathione transferase and superoxide dismutase are believed to play a key protective role in the pulmonary matrix. Antibodies to antioxidant enzymes can inhibit the extracellular activity of enzymes and reduce the possibility of antioxidant defense of the body, especially due to mechanisms that protect the lungs from injuries, inflammation and fibrosis.

## CO.32

## TGFBRII-FC REDUCES FIBROSIS AND IMPROVES LUNG FUNCTION IN FOS-RELATED ANTIGEN-2 TRANSGENIC MICE, A MODEL OF SYSTEMIC SCLEROSIS

G. Li^1^, A. Ucero^2^, T. Barr^1^, T. Bloom^1^, L. Bakiri^3^, E.F. Wagner^3^, P. Andre^1^, R. Kumar^1^

^1^*Acceleron Pharma, Cambridge, USA*, ^2^*Spanish National Cancer Research Centre - Genes, Development and Disease Group, Madrid, Spain*, ^3^*Medical University of Vienna - Department of Dermatology and Department of Laboratory Medicine, Vienna, Austria*

**Introduction:** Interstitial lung disease (ILD) is the leading cause of death for patients with systemic sclerosis (SSc). In SSc-ILD, interstitial lung fibroblasts undergo phenotypic conversion to αSMA-expressing myofibroblasts that deposit abnormal levels of extracellular matrix, leading to fibrosis and a decline in pulmonary function. TGF-β1 is a potent inducer of epithelial-to-mesenchymal transition and fibroblast-to-myofibroblast activation, two key cellular events leading to the uncontrolled deposition of fibrillar collagen, the hallmark of lung fibrosis. TGF-β1 is upregulated in lung and skin tissue of SSc patients and in animal models of lung fibrosis.

**Material and Methods:** We used TGFβRII-Fc, a selective ligand trap that neutralizes TGF-β1 and -β3, to investigate its protective effects in Fos-related antigen-2 transgenic (Fra2Tg) mice. Fra2 is upregulated in fibrotic lung tissue of SSc-ILD patients and in skin of SSc patients. Fra2Tg mice spontaneously develop severe, progressive pulmonary fibrosis, leading to death by respiratory failure at a median age of 17 weeks. In addition, Fra2Tg mice develop fibrosis and peripheral vasculopathy in skin. Hence, this model closely replicates the important pathological features of SSc in lung and skin.

**Results:** TGFβRII-Fc significantly inhibited TGF-β1-induced myofibroblast activation and epithelial-to-mesenchymal transition in vitro, as demonstrated by reduced expression of pro-fibrotic and pro-inflammatory genes (p < 0.001). Over the course of the in vivo study, Fra2Tg mice displayed a progressive increase in pulmonary collagen deposition, as determined by the fractional area of lung tissue stained by picrosirius red, and a significant decline in lung function as determined by plethysmography. Treatment of Fra2Tg mice with TGFβRII-Fc (10 mg/kg, s.c.) twice weekly for 8 weeks (starting at 8 weeks of age) completely inhibited pulmonary collagen deposition and significantly reduced (by 35%, p < 0.05) the decline in pulmonary function compared to vehicle. TGFβRII-Fc also significantly reduced dermal collagen content (p < 0.05) and dermal thickness in Fra2Tg mice and attenuated loss of body weight (p < 0.01). In addition, TGFβRII-Fc treatment inhibited pulmonary infiltration of inflammatory cells.

**Conclusions:** TGFβRII-Fc significantly reduces interstitial lung fibrosis, attenuates the decline in pulmonary function, and markedly downregulates expression of fibrosis-associated genes in a preclinical model of SSc-ILD. TGFβRII-Fc treatment may provide an effective therapeutic option for SSc patients with interstitial lung disease.

## SESSION 8 – SKIN

## CO.33

## BIOSAMPLES FROM AT RISK SSC PATIENTS SHOW CLASSIC PATHOLOGICAL SIGNS OF SCLERODERMA: OPPORTUNITY FOR A DIAGNOSIS OF PRE-CLINICAL SSC

R. Ross^1^, A. Carriero^1^, G. Abignano^1^, I. Georgiou^1^, C. Wasson^1^, C. Denton^1^, F. Del Galdo^3^, G. Migneco^2^, A. Herrick^1^

^1^*LIRMM, University of Leeds, Leeds, United Kingdom*, ^2^*Experimental Rheumatology, UCL, London, United Kingdom*, ^3^*Lydia Becker Institute of Immunology and Inflammation, University of Manchester, Manchester, United Kingdom*

**Introduction:** The VEDOSS study has recently indicated that more than 80% of patients affected by Raynaud’s phenomenon (RP) and specific SSc auto-antibodies + capillaroscopy changes satisfied ACR/EULAR 2013 criteria within 5 years. These data suggest that there is a window of opportunity for early detection of SSc in these patients. Here we aimed to determine whether sera, skin biopsies and skin fibroblasts cultured from these patients showed any biomarker sign of SSc.

**Material and Methods:** Fifty-nine at risk patients identified by having RP and SSc auto-antibodies or capillaroscopy pattern (or both) were enrolled in the national inception cohort (Kennedy Cohort). Sera were tested for IFN inducible chemokines (CXCL-9,10 and 11 and CCL2, 8 and 19) and biomarker of extracellular matrix turnover (ELF test), all previously shown to be increased in SSc. Further, two 3mm skin biopsies were taken from the forearms from 3 ACA+ve (anti-centromere antibodies), 3 SCL70+ve patients. One biopsy was subjected to histology analysis, including haematoxylin and eosin staining and immunohistological staining for Collagen Type 1, alpha-SMA, Caveolin 1 and CD31 as endothelial marker. The other biopsy was used to explant fibroblasts cultures. mRNA and protein were isolated from primary fibroblasts and processed for RT-qPCR and western blotting analyses.

**Results:** Sera from at risk patients showed overall higher IFN inducible chemokines and ELF test (P<0.05) with bimodal distribution among patients. Skin biopsies from both ACA or SCL70+ve patients showed decreased number of CD31+ cells, increased number of myofibroblasts and increased collagen bundles within the dermis, as usually seen in SSc, compared to healthy controls. In vitro, fibroblasts from both ACA or SCL70+ve patients showed average 10-fold higher collagen mRNA levels and 31-fold increased collagen protein levels compared to healthy control fibroblasts. Furthermore, fibroblasts from ACA or SCL70+ve patients showed limited TGF-beta induced increase in collagen and SMA expression, similar to SSc fibroblasts.

**Conclusions:** Although pilot in nature, this study suggests that patients “at risk” already show biomarker signs of SSc both in their sera and at skin biopsy and fibroblast level. Longitudinal studies on patients at this stage of pre-clinical disease may inform on the stratification strategies for imminent progression to clinical manifestations, and offer both insights on pathogenesis of clinical signs and a window of opportunity for delaying the onset clinical intervention trials.

## CO.34

## OPTICAL COHERENCE TOMOGRAPHY OF THE SKIN DETECTS SCLERODERMA CHANGES IN CLINICALLY UNAFFECTED SKIN: AN OPPORTUNITY FOR EARLY DETECTION OF SYSTEMIC SCLEROSIS

G. Abignano^1,2,3^, D. Temiz Karadag^1,4^, O. Gundogdu^5^, G. Lettieri^6^, M.C. Padula^1^, A.A. Padula^1^, P. Emery^2,3^, S. D’Angelo^1^, F. Del Galdo^2,3^

^1^*Rheumatology Institute of Lucania (IReL), Rheumatology Department of Lucania, San Carlo Hospital, Potenza, Italy*, ^2^*Leeds Institute of Rheumatic and Musculoskeletal Medicine, University of Leeds, Leeds, United Kingdom*, ^3^*NIHR Leeds Biomedical Research Centre, Leeds Teaching Hospitals NHS Trust, Leeds, United Kingdom*, ^4^*Department of Rheumatology, Kocaeli University, Kocaeli, Turkey*, ^5^*Department of Biomedical Engineering, Kocaeli University, Kocaeli, Turkey*, ^6^*Radiology Department, San Carlo Hospital, Potenza, Italy*

**Introduction:** The Very Early Diagnosis Of Systemic Sclerosis (VEDOSS) study has shown that 82% of patients with Raynaud’s Phenomenon, specific ANA positivity and scleroderma pattern at nail fold videocapillaroscopy will fulfil classification criteria within 5 years. This is suggesting that there is a subclinical window of opportunity to diagnose systemic sclerosis (SSc) before clinical manifestations occur. In this scenario, a non-invasive tool to diagnose SSc in clinically unaffected skin might improve the early detection of disease in at risk-patients. Optical coherence tomography (OCT) of the skin has been shown to be a sensitive and accurate biomarker of skin fibrosis in SSc. Here we aimed to assess the ability of skin OCT to “detect” SSc in clinically unaffected skin from a multicentre cohort.

**Material and Methods:** Dorsal forearm skin of SSc patients and matched-healthy controls (HC) was evaluated using VivoSight scanner (Michelson Diagnostics). Mean A-scans (mean OCT signal plotted against depth-in-tissue) were derived as previously described. Minimum Optical Density (MinOD), Maximum OD (MaxOD) and OD at 300 micron-depth (OD300) were calculated. Clinical involvement was assessed by an operator blinded to OCT findings using the mRSS. Receiver-operating characteristic (ROC) curve analysis was carried out for MinOD, MaxOD, and OD300 to evaluate their ability to discriminate between SSc and HC. Statistical analysis was performed using GraphPad Prism software V.7.0.

**Results:** One hundred seventy four OCT images were collected from 87 subjects [43 SSc (39 Female, mean age 49.7±9.1 years) and 44 gender/age-matched healthy controls (HC) (36 Female, mean age 50.2±8.3 years)] in two different SSc centres. All patients fulfilled classification criteria for SSc. OCT measures demonstrated discriminative ability in SSc skin detection with any clinical skin involvement (0-3 at site of analysis) with an AUC of 0.73 (MinOD, 95%CI 0.64-0.81), 0.77 (MaxOD, 95%CI 0.7-0.85) and 0.82 (OD300, 95%CI 0.76-0.89); p<0.0001 for all as previously indicated. Most importantly, all three measures showed comparable performance in detecting scleroderma also in clinically unaffected skin (mRss=0 at site of analysis), with an AUC of 0.7 (95%CI 0.6-0.81, p=0.001), 0.72 (95%CI 0.61-0.83, p=0.0003) and 0.72 (95%CI 0.61-0.83, p=0.0003) for MinOD, MaxOD and OD300 respectively.

**Conclusions:** Virtual biopsy by OCT recognises clinically unaffected skin of SSc patients from the HC skin. This is consistent with gene array data showing that scleroderma specific signatures are consistent in affected and clinically unaffected skin. These results inform future studies on at risk patients with clinically unaffected skin which may define a role for OCT in detecting subclinical SSc.

## CO.35

## EXPLORING ASSOCIATION BETWEEN SKIN SCORE TRAJECTORY, SURVIVAL AND PULMONARY OUTCOME IN DIFFUSE CUTANEOUS SYSTEMIC SCLEROSIS

S.I. Nihtyanova, E.C. Derrett-Smith, C. Fonseca, V.H. Ong, C.P. Denton

*UCL Medical School, Royal Free Campus, Centre for Rheumatology and Connective Tissue Diseases, London, United Kingdom*

**Introduction:** Skin thickness improves over time in most diffuse cutaneous systemic sclerosis (dcSSc) patients and the use of group level skin score (mRss) change as an endpoint is therefore challenging in clinical trials. We explore the association between individual mRss trajectories and outcome in a cohort of early dcSSc patients.

**Material and Methods:** DcSSc subjects with at least one mRss assessment within the first 5 years from onset were included. Random effect models were fitted to evaluate continuous changes in mRss over time and model-predicted individual patient intercepts and slopes were included in Cox regression to assess association between mRss and outcome.

**Results:** Of the 467 patients, 22.7% were male and mean age of disease onset was 45.5 years. Most frequent autoantibodies were anti-Scl70 in 30.2% and anti-RNA polymerase (ARA) in 30.0% of the subjects. Average mRss at 12 months from onset was 25 and there was consistent decline over subsequent years, which slowed down with longer disease duration (3.4, 2.7, 1.9 and 1.2 units at years 2, 3, 4 and 5) and higher initial mRss associated with greater subsequent decline (correlation coefficient -0.3).

Both higher baseline mRSS (intercept) and slower decline (higher slope) predicted increased risk of death with 8% increase in hazard for every unit higher baseline mRss (HR 1.08, p<0.001) and 4% increase for every unit higher slope (HR 1.04, p=0.017). Adjusting for autoantibodies did not change the estimates. ANA+ENA- subjects had the highest risk of death, followed by ATA+ (HR 0.91, p=0.677 v ANA+ENA-), while risk was lowest among ARA+ subjects (HR 0.47, p=0.002 v ANA+ENA-).

Risk of pulmonary complications was associated with rate of change in mRss but not with baseline absolute value. A unit slower yearly decline in mRss increased the hazard of pulmonary fibrosis (PF) by 3.5% and pulmonary hypertension by 7% (HR 1.035, p=0.045 and HR 1.069, p=0.042, respectively). Autoantibodies were much better predictors of PF development and the association between mRss and PF disappear after adjusting for antibody specificities. Conversely, the association between skin and PH did not change and remained after correcting for antibodies, which did not show significant association with PH development within this dcSSc cohort.

**Conclusions:** Although at a group level there is an improvement in skin over the initial 5 years, for individual patients, poor outcome for skin predicts increased risk of pulmonary complications and higher mortality.

## SESSION 10 – THERAPY

## CO.36

## DOES DOSE ADJUSTMENT AFFECT DECLINE IN FORCED VITAL CAPACITY IN PATIENTS TREATED WITH NINTEDANIB? DATA FROM THE SENSCIS TRIAL

M. Mayes^1^, O. Distler^2^, M. Kuwana^3^, V. Steen^4^, C. Stock^5^, M. Gahlemann^6^, V. Kohlbrenner^7^, S. Stowasser^8^, K.B. Highland^9^

^1^*Division of Rheumatology and Clinical Immunogenetics, University of Texas McGovern Medical School, Houston, Texas, USA*, ^2^*Department of Rheumatology, University Hospital Zurich, Zurich, Switzerland*, ^3^*Department of Allergy and Rheumatology, Nippon Medical School Graduate School of Medicine, Tokyo, Japan*, ^4^*Division of Rheumatology, Georgetown University, Washington, D.C., USA*, ^5^*Boehringer Ingelheim Pharma GmbH & Co. KG, Biberach, Germany*, ^6^*Boehringer Ingelheim (Schweiz) GmbH, Basel, Switzerland*, ^7^*Boehringer Ingelheim Pharmaceuticals, Inc., Ridgefield, Connecticut, USA*, ^8^*Boehringer Ingelheim International GmbH, Ingelheim am Rhein, Germany*, ^9^*Cleveland Clinic, Cleveland, Ohio, USA*

**Introduction:** In the SENSCIS trial in patients with systemic sclerosis-associated ILD (SSc-ILD), nintedanib reduced the annual rate of decline in forced vital capacity (FVC) versus placebo. Dose reductions from 150 mg bid to 100 mg bid and treatment interruptions were permitted to manage adverse events. We explored whether dose adjustments affected FVC decline in patients treated with nintedanib.

**Material and Methods:** The rate of decline in FVC (mL/year) over 52 weeks was assessed in all patients treated with nintedanib, and in patients treated with nintedanib who had >=1 dose reduction, >=1 treatment interruption, >=1 dose reduction and/or treatment interruption, and a dose intensity of <=90% over 52 weeks. Dose intensity was defined as the amount of drug administered divided by the amount of drug that would have been received if the 150 mg bid dose had been administered over the 52-week treatment period or until permanent treatment discontinuation. Analyses were descriptive.

**Results:** In total, 288 patients received >=1 dose of nintedanib and 288 patients received >=1 dose of placebo. Over 52 weeks, 117 (40.6%), 109 (37.8%) and 139 (48.3%) nintedanib-treated patients had >=1 dose reduction, >=1 treatment interruption, or >=1 dose reduction and/or treatment interruption, respectively. Dose intensity was <=90% in 105 (36.5%) nintedanib-treated patients. The adjusted mean (SE) rates of decline in FVC over 52 weeks were -52.4 (13.8) mL/year in all nintedanib-treated patients and -39.7 (21.4), -60.9 (22.0), -47.1 (19.7) and -44.3 (22.7) mL/year in nintedanib-treated patients who had >=1 dose reduction, >=1 treatment interruption, >=1 dose reduction and/or treatment interruption, and a dose intensity <=90%, respectively. The adjusted mean (SE) rate of decline in FVC over 52 weeks in the placebo group was -93.3 (13.5) mL/year. Statistical testing did not indicate heterogeneity in the treatment effect of nintedanib versus placebo on the annual rate of FVC decline between patients who did versus did not have each type of dose adjustment.

**Conclusions:** In the SENSCIS trial in patients with SSc-ILD, the estimated annual rate of decline in FVC was similar in nintedanib-treated patients irrespective of whether they had dose adjustments to manage adverse events.

## CO.37

## INTENSIFIED IMMUNOSUPPRESSION COMBINED WITH PLASMAPHERESIS HAS FAVORABLE LONG-TERM EFFECTS IN PATIENTS WITH EARLY-ONSET PROGRESSIVE SYSTEMIC SCLEROSIS-INTERSTITIAL LUNG DISEASE

J. Potjewijd, R. Tobal, S.J. Arends, L. Debrus-Palmans, J.G. Damoiseaux, P. Van Paassen

*Maastricht University Medical Center, Department of Clinical Immunology, Maastricht, The Netherlands*

**Introduction:** Systemic sclerosis, particularly the diffuse type with interstitial lung disease (SSc-ILD) has high morbidity and mortality. Early immunosuppression is most effective when inflammation and immune-activation and not fibrosis dominate the disease process. Autologous stem cell transplantation is promising, but toxic, and may not be necessary in all patients. Identification of the high risk patient is difficult. We evaluated long term intensified immunosuppression in combination with plasmapheresis in early-onset progressive SSc-ILD.

**Material and Methods:** Between 2000 and 2015 we included consecutive patients with <3 years progressive SSc-ILD in an uncontrolled prospective trial. Pulmonary involvement was defined as ground-glass opacities on HR-CT thorax and declined pulmonary function (FVC, DLCO/VA, measured yearly). Only patients with abnormal pulmonary DTPA–scan, indicating active alveolitis, were included. Inflammation and immune-activation was defined by elevated s-CD25 and/or the presence of IL6-inducing anti-endothelial cell antibodies (IgG-AECA). Treatment consisted of 7-10 plasmaphereses, 6 months cyclophosphamide (2-3 mg/kg) followed by long-term (mean 5,6 years) mycophenolate-mofetil. Prednisone was tapered from 40 mg/d, to 5 mg/d in 3 months. Primary endpoint was overall survival. Secondary endpoints were change in pulmonary function and safety.

**Results:** Nineteen patients, 52,6% women, aged 60±11,3 years, with SSc-ILD were included. Anti-topoisomerase, anti-centromere and other scleroderma-associated autoantibodies were present in 13, 3, and 3 patients, respectively. sCD25 was elevated in 10 patients. IgG-AECA-induced IL6 was elevated as compared to healthy control. 5-and 10-year survival rate was 89,5% and 84%. FVC increased from 83,4(SD±20,8) % predicted to +9,9%(94,1±SD5,0 p=0,12), +9,4% (93,3±SD5,7 p=0,13) and +12,4% (95,9±SD4,5 p=0,02) at 1,2 and 3 years, respectively. DLCO/VA was 70,2(SD±4,2) % predicted and increased non-significantly with +1,8%, +5,9%, and +3,6% over 3 years follow-up. DTPA scan normalized after 1 year and remained normal, indicating no residual active alveolitis. Four hospital admissions were registered (2 infection-related, 2 cardiovascular disease). Three patients died (1 lung cancer at age 70, 1 infection at age 81, 1 due to progressive SSc-ILD at age 67). Kidney function was stable in all patients. Clinical remission persisted after stopping immunosuppression.

**Conclusions:** Intensified immunosuppression combined with plasmapheresis in early onset, progressive SSc-ILD shows significant improvement in FVC after 3 years and good 10-year overall survival rate of 84%. DTPA-scan appears a valuable biomarker for active pulmonary involvement. Intensified treatment appears particularly effective in patients with proven immune-activation such as IL-6 producing IgG-AECAs, and seems to stop disease progression.

## CO.38

## RELATIONSHIP OF BASELINE MEASURES TO THE CHANGE IN THE FORCED VITAL CAPACITY IN A PHASE 3 RANDOMIZED CONTROLLED TRIAL OF TOCILIZUMAB FOR THE TREATMENT OF EARLY SYSTEMIC SCLEROSIS

D. Khanna^1^, C. Lin^2^, A. Jahreis^2^, D. Furst^3^, C. Denton^4^

^1^*University of Michigan, Ann Arbor, USA*, ^2^*Genentech, USA, San Francisco, USA*, ^3^*UCLA, Los Angeles, USA*, ^4^*University College London, London, United Kingdom*

**Introduction:** The anti–interleukin-6 (IL-6) receptor-alpha antibody tocilizumab demonstrated nonsignificant improvement in modified Rodnan skin score (mRSS, a measure of skin thickness) and clinically meaningful preservation of lung function (assessed by forced vital capacity [FVC]) in systemic sclerosis (SSc) patients in a phase 3 trial (NCT02453256 Khanna D et al. ACR 2018).

**Material and Methods:** Patients with SSc were randomly assigned 1:1 to receive weekly subcutaneous tocilizumab 162 mg or placebo for 48 weeks. The key secondary endpoint was change in baseline percent predicted FVC (ppFVC) and key exploratory endpoints included chest high-resolution computed tomography (HRCT). We performed post-hoc analyses to assess if baseline parameters influenced change in FVC at week 48 and the least square means treatment differences between tocilizumab and placebo.

**Results:** Among 210 treated patients (placebo, 106; tocilizumab, 104), 81% were women; baseline mean values were age 48 years, SSc duration 23 months, mRSS 20.4, ppFVC 82.1%, and ppDLCO 75.6%. The difference in mean change in baseline FVC at week 48 was 167 mL (95% CI: 83, 250) favoring tocilizumab. Table provides data from subgroup analyses for change in FVC at week 48 in the placebo and tocilizumab groups. Baseline variables that are associated with treatment differences of >= 200 ml at week 48 favoring tocilizumab are high platelet count, elevated CRP, male gender, disease duration < 2 years, +anti-topoisomerase I antibody, and total computer assessed quantification of lung fibrosis > 2% (Table).

**Conclusions:** In this post-hoc analyses, male gender, baseline acute phase reactants, baseline degree of fibrosis on HRCT, and autoantibodies predict a clinically meaningful difference in FVC between the tocilizumab and placebo groups at week 48. These findings will help with future trial design and select patients who will benefit with earlier treatment.

**Figure:**
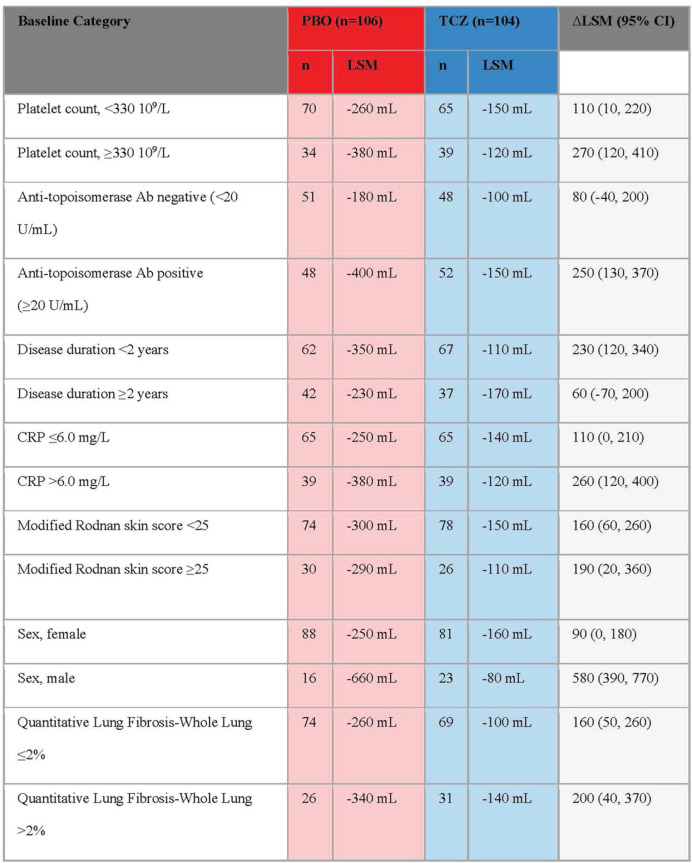


## CO.39

## SAFETY AND EFFICACY OF ABATACEPT IN EARLY DIFFUSE CUTANEOUS SYSTEMIC SCLEROSIS: RESULTS FROM THE OPEN-LABEL EXTENSION OF A PHASE II INVESTIGATOR-INITIATED RANDOMIZED CONTROLLED TRIAL

L. Chung^1^, C. Spino^2^, R. Mclain^2^, E. Bush^2^, S.R. Johnson^3^, C.P. Denton^4^, J. Molitor^5^, V.D. Steen^6^, R. Lafyatis^7^, R.W. Simms^8^, S. Kafaja^9^, T.M. Frech^10^, V. Hsu^11^, R.T. Domsic^7^, J.E. Pope^12^, J.K. Gordon^13^, M.D. Mayes^14^, N. Sandorfi^15^, F.N. Hant^16^, E.J. Bernstein^17^, S. Chatterjee^18^, F. Castelino^19^, A. Ajam^20^, D.A. Fox^2^, D.E. Furst^9^, D. Khanna^2^

^1^*Stanford University, Stanford, CA, USA*, ^2^*University of Michigan, Ann Arbor, MI, USA*, ^3^*University Health Network, Toronto, Toronto, ON, Canada*, ^4^*UCL Royal Free Hospital, London, United Kingdom*, ^5^*University of Minnesota, Minneapolis, MN, USA*, ^6^*Georgetown University, Washington DC, USA*, ^7^*University of Pittsburgh, Pittsburgh, PA, USA*, ^8^*Boston University, Boston, MA, USA*, ^9^*University of California Los Angeles, Los Angeles, CA, USA*, ^10^*University of Utah, Salt Lake City, UT, USA*, ^11^*Robert Wood Johnson University, New Brunswick, NJ, USA*, ^12^*University of Western Ontario, London, ON, Canada*, ^13^*Hospital for Special Surgery, New York, NY, USA*, ^14^*University of Texas Houston, Houston, TX, USA*, ^15^*University of Pennsylvania, Philadelphia, PA, USA*, ^16^*Medical University of South Carolina, Charleston, SC, USA*, ^17^*Columbia University, New York, NY, USA*, ^18^*Cleveland Clinic, Cleveland, OH, USA*, ^19^*Massachusetts General Hospital, Boston, MA, USA*, ^20^*Ohio State University, Columbus, OH, USA*

**Introduction:** We recently reported that abatacept (ABA) was well tolerated with potential efficacy for the treatment of early diffuse cutaneous systemic sclerosis (dcSSc) in a phase II placebo-controlled randomized trial. We report here the results of the 6-month open-label extension (OLE) period.

**Material and Methods:** Patients with dcSSc of <= 3 years duration from first non-Raynaud symptom were treated for 6 months with subcutaneous abatacept 125 mg weekly after completion of the double-blind period. Safety was assessed as well as exploratory efficacy endpoints including modified Rodnan skin score (mRSS), Health Assessment Questionnaire Disability Index (HAQ-DI), and percent predicted forced vital capacity (FVC). Descriptive statistics were performed.

**Results:** Eighty-eight patients (44 in each group) were randomized in the double-blind portion of the study. Thirty-four and 35 patients completed the 12-month trial in the ABA and PLB groups, respectively, and 32 in each group completed the 6-month OLE. Treatment emergent adverse events (TEAE) that led to study drug discontinuation included one patient in the PLB-ABA group (cardiac arrest), and two patients in the ABA-ABA group (tachycardia and URI/night sweats). Infections occurred in 9 (12 events) and 11 (14 events) patients in the PLB-ABA and ABA-ABA groups, respectively, and one event in each group was considered serious. There were no deaths during the OLE period. Mean (SD) change from baseline in mRSS was -3.56 (7.95) for PLB and -6.45 (6.43) for ABA at month 12. Mean change between months 12 and 18 in skin score was -2.23 (4.8) and -3.53 (4.75) in the PLB-ABA and ABA-ABA groups, respectively (Figure 1). HAQ-DI worsened by a mean of 0.09 (0.45) in the PLB group and improved by -0.11 (0.46) in the ABA group at month 12. The PLB-ABA group experienced subsequent improvement in HAQ-DI at month 18 by -0.12 (0.31), while the ABA-ABA group remained relatively stable (change month 18 vs. 12 -0.02 (0.37))(Figure 2). FVC %predicted declined by -3.13 (4.89) in the PLB group and -2.04 (7.64) in the ABA group at month 12, but subsequently improved by 2.49 (5.48) and 3.01 (5.26) in the PLB-ABA and ABA-ABA groups, respectively.

**Conclusions:** The 6-month OLE phase did not identify any new safety signals for ABA in the treatment of early dcSSc. Clinically meaningful improvements in skin score were observed in both the ABA and PLB groups when transitioned to open label treatment. These data support further studies of ABA in dcSSc.

**Figure:**
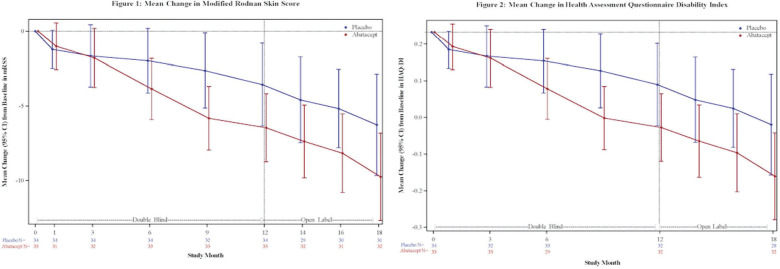


## CO.40

## RISK FACTORS AND LONG-TERM FOLLOW UP OF PATIENTS WITH SYSTEMIC SCLEROSIS TREATED WITH OR WITHOUT HEMATOPOIETIC STEM CELL TRANSPLANTATION IN THE GERMAN NETWORK FOR SYSTEMIC SCLEROSIS

N. Blank^1^, S. Hamacher^2^, K. Kuhr^2^, E. Siegert^3^, U. Müller-Ladner^4^, G. Riemekasten^5^, C. Günther^6^, I. Kötter^7^, G. Zeidler^8^, C. Pfeiffer^9^, A. Juche^10^, I. Jandova^11^, J. Ehrchen^12^, M. Schmalzing^13^, L. Susok^14^, T. Schmeisser^15^, C. Sunderkötter^16^, J.H. Distler^17^, M. Worm^18^, A. Kreuter^19^, T. Krieg^2^, P. Moinzadeh^2^, N. Hunzelmann^2^, J. Henes^20^

^1^*University of Heidelberg, Rheumatology, Heidelberg, Germany*, ^2^*University of Cologne, Dermatology and Medical Statistics, Cologne, Germany*, ^3^*University of Berlin, Charite, Rheumatology, Berlin, Germany*, ^4^*University of Giessen, Kerckhoff Clinic, Rheumatology, Bad Nauheim, Germany*, ^5^*University of Schleswig-Holstein, Rheumatology, Luebeck, Germany*, ^6^*University of Dresden, Dermatology, Dresden, Germany*, ^7^*University of Hamburg, Rheumatology, Hamburg, Germany*, ^8^*Johanniter Hospital, Rheumatology, Treuenbrietzen, Germany*, ^9^*University of Ulm, Dermatology, Ulm, Germany*, ^10^*Immanuel Hospital, Rheumatology, Berlin, Germany*, ^11^*University of Freiburg, Rheumatology, Freiburg, Germany*, ^12^*University of Muenster, Dermatology, Muenster, Germany*, ^13^*University of Wuerzburg, Rheumatology, Wuerzburg, Germany*, ^14^*University of Bochum, Dermatology, Bochum, Germany*, ^15^*St. Josef Hospital Wuppertal, Dermatology, Wuppertal, Germany*, ^16^*University of Halle, Dermatology, Halle, Germany*, ^17^*University of Erlangen, Rheumatology, Erlangen, Germany*, ^18^*University of Berlin, Charite, Dermatology, Berlin, Germany*, ^19^*Helios St. Elisabeth Hospital, Dermatology, Witten-Herdecke, Germany*, ^20^*University of Tuebingen, Rheumatology, Tuebingen, Germany*

**Introduction:** Despite several trials of hematopoietic stem cell transplantation (HSCT) for patients with an aggressive course there is still no general acceptance of HSCT for SSc. Because HSCT has a risk of transplant-related mortality (TRM), only selected patients were considered for HSCT.

**Material and Methods:** This is a retrospective multicenter analysis of patients in the DNSS registry. The DNSS data base with 4714 patients was screened for patients who received HSCT. The vast majority of patients had one or more follow-up visits. Patients with diffuse cutaneous (dc)-SSc were analyzed as SSc control group. Baseline was the time of HSCT or the first documented visit in the DNSS registry.

**Results:** Eighty patients with HSCT-treated SSc were identified in 12 DNSS centers. The clinical course was dc-SSc in 67 (84%), lc-SSC in 7 (9%) and overlap (ol)-SSc in 6 (8%) patients in the HSCT group. The SSc-control group comprised 1433 patients who did not receive HSCT. Risk factors for an aggressive course were male gender, dc-SSc, anti-Scl70, DLCOc-SB <75% and mRSS >20.

Six patients (7.5%) in the HSCT group and 113 (7.9%) SSc controls died during follow-up. The 6 patients were older at SSc onset (median 45.5 years (IQR 38-56)), had a shorter time to HSCT (1.7 (1.2-3.1) years) and were older at HSCT (age 49 (41-57) years) compared to SSc controls. SSc controls had an SSc onset at age 38.0 (29-45) years, time to baseline 3.9 (1.7–9.2) years and age at baseline of 42.0 (36-53) years. None of the demographic parameters, organ involvement and biomarkers were significantly different between HSCT patients who died and HSCT patients alive.

Follow up in the DNSS registry was documented up to 13 years. Patients in the HSCT group had an mRSS 17.0 (8.3-25.8) at baseline, 11.0 (5.0-18.0) after 1 year and 8.0 (5.0-16.0) after 5 years. The mRSS in SSc controls was 14.0 (8.0-22.0) at baseline, 11.0 (6.0-18.0) after 1 year and 11.0 (5.0-17.0) after 5 years. The diffusion capacity DLCO-SB was lower in the HSCT group (56.0% (48-68)) compared to SSc controls (63.4% (49.0-77.0)) and remained stable in both groups during the following 5 years.

**Conclusions:** SSc-Patients with risk factors should be considered for HSCT in centers with experience. We provide follow-up data for 5 years after HSCT. Deaths (TRM or progressive SSc) occurred in both groups at comparable rates. The mRSS in the HSCT group improved gradually during follow-up, whereas the mRSS in SSc controls remained stable.

## CO.41

## RIOCIGUAT IN PATIENTS WITH EARLY DIFFUSE CUTANEOUS SYSTEMIC SCLEROSIS AND INTERSTITIAL LUNG DISEASE: RESULTS FROM THE RANDOMIZED, DOUBLE-BLIND, PLACEBO-CONTROLLED PHASE IIB STUDY (RISE-SSC)

O. Distler^1^, Y. Allanore^2^, C.P. Denton^3^, M. Kuwana^4^, M. Matucci-Cerinic^5^, J.E. Pope^6^, K. Laapas^7^, F. Behmenburg^8^, M. Wosnitza^8^, D. Khanna^9^

^1^*Department of Rheumatology, University Hospital Zurich, Zurich, Switzerland*, ^2^*Rheumatology A Department, Cochin Hospital, Paris Descartes University, Sorbonne Paris Cite, Paris, France*, ^3^*UCL Division of Medicine, Royal Free Campus, London, United Kingdom*, ^4^*Department of Allergy and Rheumatology, Nippon Medical School Graduate School of Medicine, Tokyo, Japan*, ^5^*Department of Experimental and Clinical Medicine, University of Florence, Florence, Italy*, ^6^*Department of Medicine, Division of Rheumatology, University of Western Ontario, St. Josephs Health Care, London, On, Canada*, ^7^*StatFinn Oy, Espoo, Finland*, ^8^*Bayer AG, Wuppertal, Germany*, ^9^*Division of Rheumatology, University of Michigan Scleroderma Program, Ann Arbor, MI, USA*

**Introduction:** Diffuse cutaneous systemic sclerosis (dcSSc) is characterized by tissue fibrosis and, in some patients, interstitial lung disease (ILD). We present results from the Phase IIb RISE-SSc study (NCT02283762) of the soluble guanylate cyclase stimulator riociguat in early dcSSc.

**Material and Methods:** Inclusion criteria: dcSSc < / = 18 months; modified Rodnan skin score (mRSS) > / = 10 and < / = 22; forced vital capacity (FVC) > / = 45% of predicted; hemoglobin-corrected lung diffusion capacity for carbon monoxide (DLCO) > / = 40% of predicted. Patients were randomized double-blind to placebo or riociguat 0.5-2.5 mg three times daily. The primary endpoint was change in mRSS from baseline to Week 52. Exploratory analyses examined changes in lung function between baseline and Week 52 in patients with ILD defined as: baseline FVC% predicted 50-75% (n=18); medical history of ILD (n=25); and ILD confirmed by high-resolution computed tomography (HRCT) (n=22).

**Results:** In total, 60 patients were randomized to riociguat; 61 to placebo. Baseline mRSS was comparable in the two groups. The change in mRSS to Week 52 was -2.09±5.66 with riociguat and -0.77±8.24 with placebo (difference of least squares means -2.34; P=0.08). Mean±SD changes from baseline to Week 52 for riociguat vs placebo in FVC% predicted were -2.38±7.52 vs -2.95±9.73 with riociguat and placebo, respectively (nominal P=0.90), and in DLCO%, -2.31±10.08 vs -4.09±12.19, respectively (nominal P=0.35). Analysis of patients with SSc-ILD according to ILD definition showed differences between treatment arms in these subgroups. Changes in FVC% predicted for riociguat and placebo, respectively, were -2.66±3.81 (n=11) and -7.63±13.14 (n=11) in the ILD by medical history subgroup; +0.68±5.65 (n=8) and -8.70±13.95 (n=4) in the baseline FVC% predicted 50-75% subgroup; and -1.10±4.62 (n=12) and -8.97±13.92 (n=9) in the ILD by HRCT subgroup. In all three ILD subgroups, changes in DLCO% predicted showed trends in favor of riociguat vs placebo.

In the overall population, serious adverse events (SAEs) occurred in 9/60 (15.0%) riociguat patients and 15/61 (24.6%) placebo patients; no new safety signals were observed. The rates of SAEs for riociguat and placebo, respectively, were 1/12 (8.3%) and 3/13 (23.1%) in the ILD by medical history subgroup; 2/11 (18.2%) and 1/7 (14.3%) in the baseline FVC% predicted 50-75% subgroup; and 1/12 (8.3%) and 3/10 (30.0%) in the ILD by HRCT subgroup.

**Conclusions:** These exploratory data suggest that riociguat has good tolerability and showed trends toward preservation of lung function in patients with early dcSSc and evidence of ILD.

## Posters

## 1. Pathogenesis (Autoantibodies)

## P.001

## REGULATOR COMBINATIONS IDENTIFY SYSTEMIC SCLEROSIS PATIENTS WITH MORE SEVERE DISEASE

Y. Wang, J. Franks, D. Toledo, M. Whitfield

*Geisel School of Medicine at Dartmouth, Lebanon, USA*

**Introduction:** Gene expression associated with inflammation, fibrosis and innate immune system activation are dominant features of the systemic sclerosis (SSc) molecular landscape. The gene expression regulators that underlie the intrinsic subsets of SSc have not been systemically characterized. Here we present a computational framework to calculate the activity scores of gene expression regulators and identify their associations with clinical outcomes.

**Material and Methods:** Gene expression profiles were obtained four published, independent SSc datasets (431 samples). Targets of 836 regulators were obtained from the MSigDB. Regulator activity scores were calculated using the BASE algorithm. Correlations were calculated between activity scores and samples’ mRSS in each dataset. Clinically relevant regulators were identified with the top 50 highest mean correlations. A regulator-target network was constructed to visualize relationships.

**Results:** We found regulator activity scores can reproduce the intrinsic molecular subsets with distinct sets of regulators identified for inflammatory, fibroproliferative and normal-like samples. Examples include SMAD4 and NFAT for inflammatory, and MYC and FOX TFs for fibroproliferative samples. A regulator interaction network was created to provide novel insights for SSc, and captures their association with MRSS, allowing us to visualize the connectivity between different components of the regulator network (Fig. 1A). By using combinations of regulators, we identify a subgroup of fibroproliferative and inflammatory SSc samples that have more severe pathophenotypes. Inflammatory patients with higher SMAD4 and NFAT activities, demonstrating strong fibrotic and immune activation, had significant more severe mRSS than samples with low expression of both pathways (Fig. 1B). A subgroup of patient with higher RUNX1-related core binding factor and FOXO4 activities were identified that had higher incidence of pulmonary fibrosis (Fig. 1C). These individuals had significantly higher mRSS and a larger fraction of individuals with ILD.

**Conclusions:** Using the activities of specific gene expression regulators, we identified a subset of SSc patients within the intrinsic subsets that show increased expression of innate immune and fibrotic pathways. Patients with high activity of these two pathways had increased disease severity. This further highlights the role of innate immune and fibrotic transcriptional programs in the pathogenesis of SSc. The association between the activation of regulators and multiple clinical variables of SSc could be applied to personal diagnostic and treatment paradigms for patients with SSc.

**Figure:**
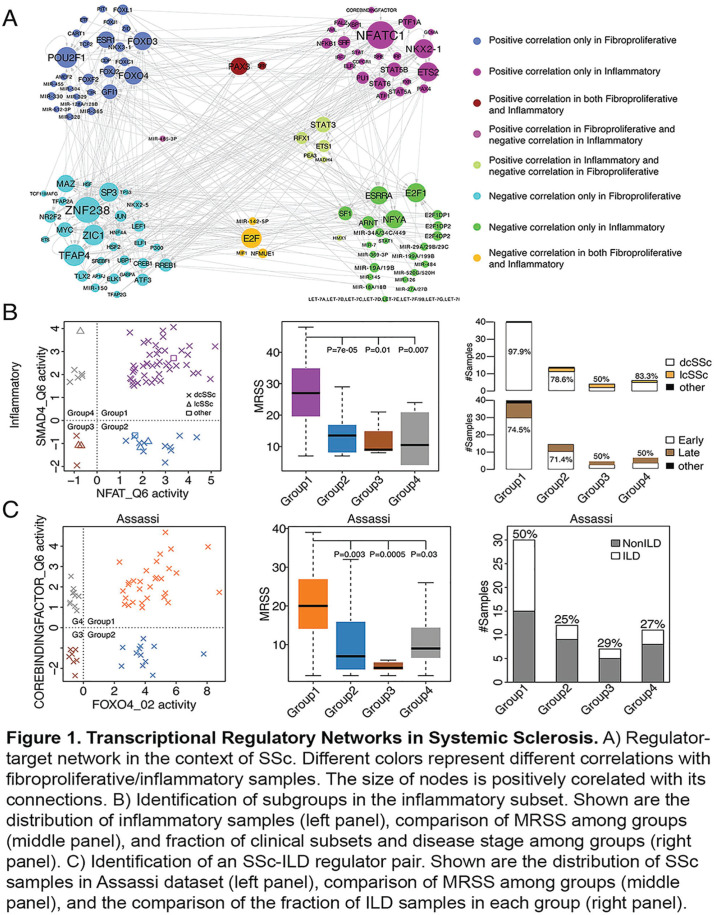


## P.002

## EPIGENETIC INSIGHTS FROM DIFFERENTIAL CHROMATIN ACCESSIBILITY PATTERNS IN DERMAL FIBROBLASTS AND SKIN-LIKE ORGANOIDS FROM PATIENTS WITH SYSTEMIC SCLEROSIS

T. Wheeler^1^, Y. Wang^2^, J.-Q. Chang^1^, D.M. Toledo^1^, B.K. Mehta^1^, M.S. Brown^1^, R. Bhandari^3^, M. Huang^1^, T. Wood^2^, P.A. Pioli^3^, M.L. Whitfield^1,3^

^1^*Department of Molecular and Systems Biology, Hanover, NH, USA*, ^2^*Department of Biomedical Data Science, Hanover, NH, USA*, ^3^*Department of Microbiology and Immunology, Hanover, NH, USA*

**Introduction:** Non-coding epigenetic changes likely play a key role in systemic sclerosis (SSc) biology. In this study we analyzed differentially accessible chromatin regions using Assay for Transposase Accessible Chromatin with sequencing (ATAC-seq) in both healthy control (HC) and SSc fibroblast cell lines in monolayer/2D and 3D skin-like tissue organoids.

**Material and Methods:** HC and SSc fibroblast lines were isolated from skin biopsies. Fibroblasts were seeded in transwell chambers and cultured for 5 weeks to obtain 3D tissues. ATAC-seq was performed on both 2D and 3D tissues following dissociation. Libraries were sequenced, paired end reads were aligned to the hg19 reference genome, and peaks were called in ZINBA/MACS2. Data underwent quality control analyses and unsupervised hierarchal clustering through Epinomics. Genomic location of peaks was determined using ChIPseeker. Transcription factor enrichment was performed by analyzing accessibility at TF motifs. The Integrative Genomics Viewer was used to analyze the genomic context of specific regions of interest.

**Results:** ATAC-seq libraries contained ~10-30 million reads and had comparable profiles to standard data sets (Fig. 1A-D). SSc fibroblasts contained significantly more open chromatin (Fig. 1E). Additionally, the percentage of peaks within distal/intergenic regions in SSc fibroblasts was significantly higher than in HC lines (Fig. 1F) and the percentage of peaks in 5’ untranslated regions (5’UTRs) was significantly lower (Fig. 1G). Transcription factor (TF) motif enrichment showed general clustering by disease state (Fig. 1H). Differentially accessible motifs in SSc cultures included SMAD4, STAT3, and FOX family proteins. A differentially accessible region of significant interest was identified in a combined 2D/3D heatmap in which it was the only genomic peak which distinguished between SSc and HC samples (Fig. 1G). This region falls within an intron of a gene on chromosome 8 and contains a putative enhancer predicted to contain a binding site for the transcription factor STAT3 (Fig. 1I).

**Conclusions:** We obtained high quality data from both 2D and 3D cultures. Further analyses demonstrate that SSc fibroblasts maintain a distinct chromatin accessibility profile as compared to HC fibroblasts. This is characterized by increased accessibility, particularly in distal/intergenic regions which are commonly associated with regulatory elements. SSc chromatin is also less accessible in 5’UTRs. The majority of the cell lines cluster by disease state with TF enrichment indicating dysregulation of key motifs including SMAD4, STAT3, and FOX family proteins. Additionally, we were able to identify a region of significant interest containing a putative enhancer and predicted binding site for STAT3.

## P.003

## EXOME-WIDE ASSOCIATION ANALYSIS IDENTIFIES LRP2BP AS A SUSCEPTIBILITY GENE FOR ENDOTHELIAL INJURY IN SYSTEMIC SCLEROSIS

J. Wang^1^, W. Pu^1^, W. Wu^2^, Q. Liu^2^, Y. Ma^3^, W. Tu^4^, X. Zuo^5^, G. Guo^6^, S. Jiang^7^, Y. Zhao^4^, J. Reveille^8^, M. Mayes^8^, J. Li^7^, E.B. Lee^9^, X. Zhang^5^, J. Xu^2^, Z. Zhang^2^, X. Zhou^8^, H. Zou^10,11^

^1^*State Key Laboratory of Genetic Engineering, School of Life Sciences, Fudan University, Shanghai, China*, ^2^*Department of Dermatology, Huashan Hospital, Fudan University, Shanghai, China*, ^3^*Six Industrial Research Institute, Fudan University, Shanghai, China*, ^4^*Division of Rheumatology, Shanghai TCM-integrated Hospital, Shanghai, China*, ^5^*Key Laboratory of Dermatology, Anhui Medical University, Ministry of Education, Hefei, China*, ^6^*Department of Rheumatology, Yiling Hospital, Shijiazhuang, China*, ^7^*Human Phenome Institute, Fudan University, Shanghai, China*, ^8^*Division of Rheumatology, Department of Internal Medicine, University of Texas Medical School, Houston, USA*, ^9^*Department of Internal Medicine, Seoul National University College of Medicine, Seoul, South Korea*, ^10^*Department of Rheumatology, Huashan Hospital, Fudan University, Shanghai, China*, ^11^*Institute of Rheumatology, Immunology and Allergy, Fudan University, Shanghai, China*

**Introduction:** Systemic Sclerosis (SSc) is a complex autoimmune disease characterized by immune dysregulation, fibrosis, and vasculopathy. Although the pathogenesis of SSc remains unclear, accumulating evidence has suggested that genetic factors play critical roles. In addition, genetic heterogeneity in different ethnic populations may significantly alter the clinical manifestations of SSc. To further characterize the genetic factors in SSc pathogenesis, we performed a two-stage exome-wide association study to identify SSc risk variants in Han Chinese population.

**Material and Methods:** We performed the first two-stage genome-wide association study of SSc in Han Chinese population including 1006 SSc patients and 5790 controls. In discovery stage, 527 SSc patients and 4694 controls were recruited and genotyped with Illumina Human Exome Asian Beadchip. In validation study, an independent sample set of 479 SSc patients and 1096 controls were examined with the candidate SNPs found in the discovery stage.

**Results:** After validation of the candidate SNPs identified in the discovery stage, we found six SNPs showed consistent associations that reached genome-wide significance. Among them, rs7574865 (Pcombined = 3.87 ×10-12) located within STAT4 was identified previously in studies using samples of European-ancestry. Additionally, three other SNPs are new susceptibility loci located in EDC (epidermis differentiation complex) region (FLG2, rs76285340, Pcombined = 2.30×10-11; FLG, rs146891517, Pcombined = 3.13×10-11; FLG3, rs75287745, Pcombined = 4.26×10-10). Furthermore, two SNPs located in the exon 3 of IGHM (rs45471499, Pcombined = 1.15×10-9) and the upstream of LRP2BP (rs4317244, Pcombined = 4.17×10-8) were also found as susceptible loci for SSc. Moreover, rs4317244 was identified as an eQTL for LRP2BP that regulates the tight junction, cell cycle, and apoptosis in microvascular endothelial cell lines. Furthermore, LRP2BP knockdown in HMEC-1 cell lines significantly inhibited microvascular morphogenesis, suggesting the vital role of LRP2BP in endothelial injury.

**Conclusions:** Collectively, we identified five novel SNPs to be associated with SSc in Han Chinese population and validated an SSc-associated SNP of STAT4, which has been reported in both Caucasians and Chinese. The rs4317244 [G] of LRP2BP gene may contribute to the endothelial injury in SSc patients through down-regulating LRP2BP in endothelial cells, suggested the importance of LRP2BP in the pathogenesis of SSc.

**Figure:**
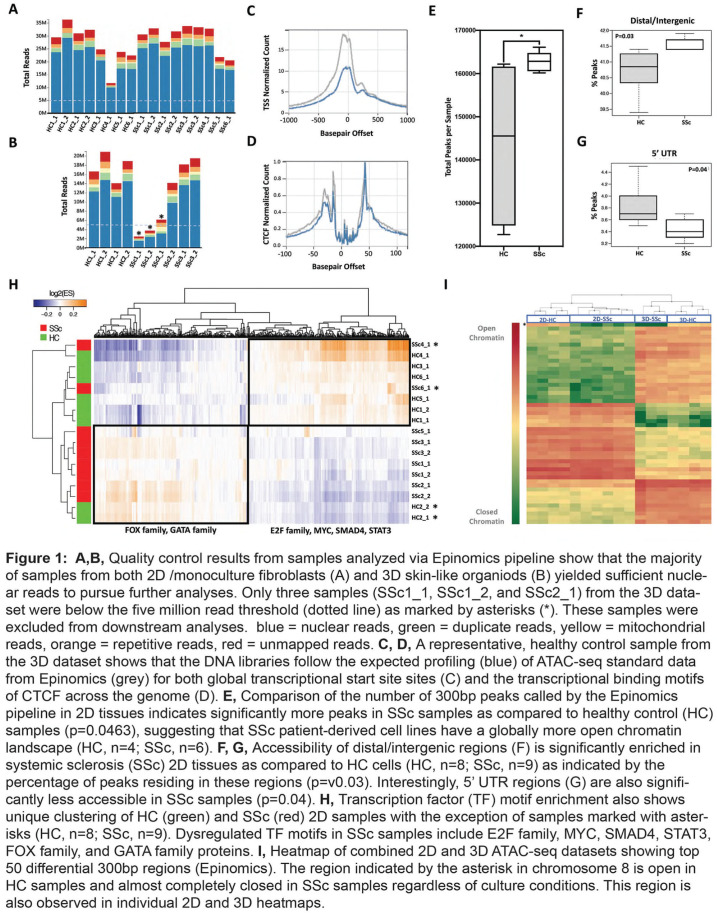


## P.004

## INCREASED PLATELET DEPOSITION UNDER SHEAR STRESS CONDITIONS IN PATIENTS WITH SCLERODERMA

I. Vigdorchick^1,2^, Y. Levy^1,2^

^1^*Department of Internal Medicine, Meir Medical Center, Kfar Saba, Israel*, ^2^*Sackler Faculty of Medicine, Tel Aviv University, Tel Aviv, Israel*

**Introduction:** Scleroderma is a rheumatic disease involving the skin, lung, gastro- intestinal tract, kidney, heart and the microvascular system. Vascular endothelial damage may contribute to the inflammatory process found in the vessel wall, which in turn contribute to the hypercoagulable state thathas already been reported in the disease. Nevertheless some studies fail to support evidence for platelet hyperactivity in scleroderma. In this study we used Cone and Plate(let) analyzer (CPA). The CPA technology is testing whole blood platelet adhesion and aggregation under arterial flow conditions. The adhered platelets are stained and the percentage of surface covered (SC) by the stained objects and the average size (AS) of the objects are determined by image analyzer.

**Material and Methods:** In the current study we employed the CPA technology for assessment of platelet deposition on Polystyrene surface (PS) under shear conditions (1800/second). This method allows for investigation platelet aggregation under conditions that mimic blood flow. The assays were applied from 41

patients with scleroderma and from 37 healthy controls. The degree of haemostasis was expressed in two terms: the percent of surface coverage (SC) and average size (AS) of the adherent aggregates.

**Results:** Our results demonstrated significant higher surface coverage (SC) and average size (AS)levels in scleroderma patients.

Cone and plate analyzer results: Percent of SC and AS of the adherent aggregates were high in majority of patients with scleroderma. Difference between patient and control groups is highly significant (p &lt;0.001).

**Conclusions:** Most of patients suffering of scleroderma exhibit an enhanced adhesion and aggregation of platelets on PS under shear stress conditions compared to healthy controls. In the future, CPA analysis of platelets may be applied to measure hypercoagulability level in scleroderma patients, thus direct us to select the right patients that can obtain benefit from antiaggregant treatment.

## P.005

## ANTICENTROMERE ANTIBODY LEVELS AND ISOTYPES ASSOCIATE WITH DISEASE SEVERITY IN SYSTEMIC SCLEROSIS

N. Van Leeuwen^1^, J. Bakker^2^, O. Distler^3^, A.-M. Hoffman-Vold^4^, A. Grummels^2^, C. Wortel^1^, H. Fretheim^4^, S. Jordan^3^, T. Huizinga^1^, R. Toes^1^, H. Scherer^1^, J. De Vries-Bouwstra^1^

^1^*Leiden University Medical Center, Department of Rheumatology, Leiden, The Netherlands*, ^2^*Leiden University Medical Center, Department of Clinical Chemistry, Leiden, The Netherlands*, ^3^*University Hospital Zurich, Zurich, Switzerland*, ^4^*Oslo University Hospital, Oslo, Norway*

**Introduction:** We evaluated whether anticentromere antibody (ACA) isotype expression and levels: 1. associate with disease severity, 2. differ between patients with very early SSc and SSc, and 3. can identify very early SSc patients that will progress to SSc.

**Material and Methods:** All ACA IgG+ patients fulfilling the American College of Rheumatology (ACR) 2013 criteria for SSc and ACA+ IgG patients with very early SSc (based on ACA,Raynaud and puffy fingers or abnormal nailfold capillaroscopy but not fulfilling ACR 2013 criteria), from the prospective SSc cohorts from the Leiden University Medical Centre, the University Hospital Zurich, and the Oslo University Hospital were included. Patients were categorized in three groups according to disease severity: very early SSc, SSc without organ involvement, and SSc with organ involvement. Organ involvement was defined as any of: digital ulcers, interstitial lung disease, pulmonary arterial hypertension, renal crisis and myocardial involvement. Associations between isotype presence and levels and disease severity were evaluated with logistic regression. ACA response characteristics were compared between very early SSc patients that progressed to ACR 2013 criteria and those who did not.

**Results:** Ninety patients(20%) had very early SSc and 355(80%) fulfilled the ACR criteria, in which 41% (n=144) were classified as SSc with organ involvement . Very early SSc patients show lowest titers of IgM and IgG. In contrast, SSc patients with organ involvement most often express ACA IgM and show highest titers of all isotypes, indicating a more active, specific B cell responses. With adjustment for disease duration and age, ACA IgG levels were significantly higher in SSc patients vs. very early SSc, and in SSc patients with organ involvement vs. SSc without organ involvement (Table 1). Of all very early SSc patients with follow-up (n=70; median FU 2.1 year) 30% (n=26) progressed to SSc, mostly due to skin progression (88%), and 23% developed lung involvement, after a median period of 4.3 year. As age and follow-up duration were significantly higher in the progressors we were underpowered to analyse isotype characteristics by means of regression. However, again, we observed a trend for higher ACA IgG levels in the very early SSc patients progressing to SSc fulfilling ACR 2013 criteria.

**Conclusions:** Here we show that ACA IgG levels increase with increasing disease severity, from very early SSc, to SSc without organ involvement and SSc with organ involvement. Our data indicate that ACA+ SSc specific B cell responses are potentially involved in disease-relevant pathogenic processes.

**Figure:**
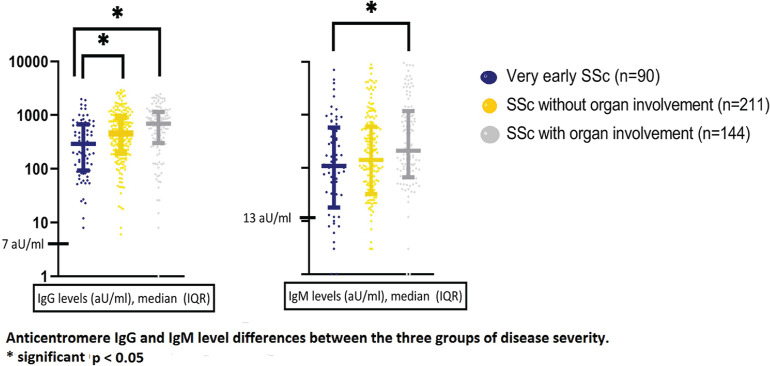


## P.006

## SPECIFIC IMMUNODOMINANT PDGFRALPHA AND NADPH OXIDASE HLA-I TRANS-SPLICED PEPTIDES ARE DEMONSTRATED IN SSC LYMPHOCYTES

S. Svegliati^1^, G. Moroncini^1^, T. Spadoni^1^, M. Mozzicafreddo^2^, M. Cuccioloni^2^, A. Grieco^1^, C. Paolini^1^, C. Tonnini^1^, S. Agarbati^1^, A. Funaro^3^, A. Gabrielli^1^, E. Avvedimento^4^

^1^*Clinica Medica Dipartimento di Scienze Cliniche e Molecolari, Università Politecnica delle Marche, Ancona, Italy*, ^2^*Università di Camerino, Scuola di BioScienze e medicina Veterinaria, Camerino, Italy*, ^3^*Dipartimento di Scienze Mediche, Università di Torino, Torino, Italy*, ^4^*Dipartimento di Medicina molecolare e Biotecnologie mediche, Università Federico II, Napoli, Italy*

**Introduction:** Scleroderma stimulatory IgG autoantibodies (SSc-IgG) recognize specific sequences of the Platelet-derived growth factor receptor alpha(PDGFRalpha) and activate collagen gene expression via reactive oxygen species (ROS) intracellular production by NADPH oxidase in the lipid rafts of the plasma membrane. We investigated whether: i. NADPH forms a molecular complex with PDGFRalpha: ii. NADPH sequences are targeted by SSc autoantibodies

**Material and Methods:** The interaction between PDGFRalpha and NADPH oxidase subunits was evaluated by immunoprecipitation of fibroblast total lysate with commercial anti-human PDGFRalpha, anti-human NADPH, SSc-IgG and human monoclonal anti-PDGFRalpha antibodies (Moroncini et al., A&R 2015). To predict the amino acid sequences of PDGFRalpha and NADPH oxidase involved in the formation of a molecular complex, in silico molecular docking between homology-modeled monomeric human PDGFRapha (Moroncini et al., A&R 2015) and NADPH was performed. A conformational peptide library comprising PDGFRalpha and NADPH motifs, designed upon molecular docking prediction, was synthesized and tested with serum samples of SSc patients and healthy donors (HD). HLA I-peptide complexes were immunoaffinity purified from peripheral blood mononuclear cells (PBMC) of SSc patients and HD and the bound peptides were obtained by chromatography and analysed by mass spectrometry. Specific peptide sequences and scrambled controls were used as markers

**Results:** PDGFRalpha forms a complex with NADPH as assayed by anti-PDGFRalpha, NADPH oxidase subunits specific antibodies and by SSc-IgG. Using as guide an in silico model that describe the interacting domains of PDGFRalpha and NADPH oxidase, we screened a conformational peptide library with SSc serum samples. We identified SSc-specific immunodominant epitopes belonging to both proteins. By MS spec analysis we demonstrated the presence in SSc lymphocytes of spliced peptides of both proteins. We were not able to identify the same peptides in cells derived from HD.

**Conclusions:** This is the first demonstration of the existence of spliced peptides in an autoimmune disease. Moreover, these data show the participation of T cells in SSc immunorecognition.

## P.007

## PATHOGENIC ACTIVATION OF MESENCHYMAL STEM CELLS IS INDUCED BY THE DISEASE MICROENVIRONMENT IN SYSTEMIC SCLEROSIS

Y. Suleman^1^, H. Liu^1^, Z. Taki^1^, E. Gostjeva^2^, W. Thilly^2^, H. Lopez^1,3^, S. Vigneswaran^1^, B. Ahmed Abdi^1^, C. Denton^1^, S. O Reilly^4^, S. Xu^1^, R. Stratton^1^

^1^*UCL Centre for Rheumatology and Connective Tissue Diseases, Royal Free Hospital Campus, London, United Kingdom*, ^2^*Department of Biological Engineering, Massachusetts Institute of Technology Cambridge, Massachusetts, USA*, ^3^*MuriGenics, Inc., 941Railroad Avenue, Vallejo, California, USA*, ^4^*Pathogen Genomics Unit, University College London, London, United Kingdom*

**Introduction:** In systemic sclerosis (SSc), persistent dysregulated tissue repair leads to progressive fibrosis of the skin and internal organs. The role of mesenchymal stem cells (MSCs), which characteristically initiate and regulate tissue repair, has not been fully evaluated. We sought to investigate whether actively dividing MSCs are present in the SSc involved tissue, and test whether exposure to the early disease microenvironment activates MSCs leading to pathogenic cells.

**Material and Methods:** Skin biopsy material from recent onset diffuse SSc patients were examined by collagenase spread of 1mm thick surface-parallel sections, in order to identify metakaryotic dividing stem cells in each tissue plain. Adipose-derived MSCs from healthy controls were treated with dermal blister fluid from recent onset diffuse SSc patients and profiled by next generation sequencing or evaluated for phenotypic changes relevant to the disease.

**Results:** MSC-like cells undergoing active metakaryotic divisions were identified in SSc but not control sections, most prominent in the deep dermis. Furthermore, exposure to the disease microenvironment caused selective MSC activation, inducing a resistant and invasive myofibroblast gene expression signature centered on ERBB2 (HER2) and CTGF, whilst reducing vascular repair and adipogenesis pathways. Factors present in the disease microenvironment implicated in inducing the transdifferentiation, include the pro-fibrotic growth factor TGF beta, extracellular lactate, as well as enhanced mechanosensing, whereas IL-31, an SSc-associated cytokine, induced mobilization and osteogenic differentiation (calcinosis) of the MSCs.

**Conclusions:** Dividing MSC-like cells are present in the SSc disease microenvironment. Factors derived from the disease microenvironment promote transdifferentation of MSCs to ERBB2 and CTGF expressing myofibroblast-like cells.

## P.008

## ALPHA- SMOOTH MUSCLE ACTIN EXPRESSION ON ENDOTHELIAL PROGENITORS CELLS OF SYSTEMIC SCLEROSIS (SSC) PATIENTS: POSSIBLE ROLE IN THE ENDOTHELIAL-TO-MESENCHIMAL TRANSITION PROCESS

K. Stefanantoni, C. Barbati, T. Colasanti, C. Angelelli, G. Pellegrino, C. Alessandri, G. Valesini, V. Riccieri

*UOC Reumatologia - Dipartimento di Medicina Interna e Specialità Mediche- Sapienza Università di Roma, Rome, ITALY*

**Introduction:** Endothelial-to-mesenchymal transition (EndoMT) seems to be involved in Systemic Sclerosis (SSc) pathogenesis. In this process endothelial cells lose their specific markers, and express mesenchymal markers such as alpha smooth muscle actin (α-SMA). Circulating endothelial progenitors cells (EPCs) derive from bone marrow stem cells and contribute to de novo vessels formation and in SSc seem to be impaired in their number and function. Our aim was to assess the expression of α-SMA, possibly associated with a pro-mesenchymal switch (EndoMT) of the circulating Early (CD34+KDR+CD 133+) and Late (CD34+KDR+) EPCs in the peripheral blood of SSc patients and of patients with Very Early Diagnosis of SSc (VEDOSS) compared with healthy controls (HC) using flow cytometry.

**Material and Methods:** We enrolled 11 patients (7 SSc and 4 VEDOSS), classified respectively according to the classification criteria for SSc and for VEDOSS not fulfilling SSc criteria, and 10 HC. Phenotypic characterization was performed as previously described by Vasa et al. using a FACS Calibur (BD Immunocytometry Systems). EPCs number was expressed as a percentage of cells within the lymphocyte gate.

**Results:** We found a significantly higher percentage of α-SMA positive Early EPCs (CD34+KDR+CD 133+α-SMA+) in all patients respect to HC (0,06% ±0,03 vs 0,03% ± 0,01; p=0,0149) particularly in VEDOSS patients (0,07%±0,01 vs 0,03±0,01 p=0,008). Moreover, in VEDOSS patients, also the percentage of Early EPCs (CD34+KDR+CD 133+), Late EPCs (CD34+KDR+)and α -SMA positive Late EPCs (CD34+KDR+α-SMA+) were significantly higher than in HC (0,05%±0,01 vs 0,03%±0,01 p=0,05; 0,07%±0,01 vs 0,04%±002 p=0,04; 0,06%±0,01 vs 0,04%±0,02 p=0,05). Besides Early EPCs and α -SMA positive Early EPCs percentages seem to be significantly reduced by Iloprost (p=0,05 and p=0,01 respectively), by Calcium Channel Blockers (CCB) (p=0,05 and p=0,03) and by DMARDs (p=0,017 and p=0,013).

**Conclusions:** In our study we found higher levels of EPCs, in particular α -SMA positive Early EPCs in both groups of patients (SSc and VEDOSS) respect to HC. Thus we can hypothesize a predominant pro-mesenchymal phenotype of this kind of EPCs. This could be considered the expression of the involvement of EPCs in EndoMT process and could better explain the controversial role of EPCs in SSc pathogenesis. Very interesting is the finding of alower percentage of Early EPCs, and in particular of α-SMA positive Early EPCs, in those patients taking Iloprost, CCB and DMARDs, suggesting a potential effect of these drugs on this subgroup of EPCs.

## P.009

## CLINICAL SIGNIFICANCE OF ISOLATED RO52/TRIM21 AUTOANTIBODIES IN THE WAIKATO HOSPITAL SYSTEMIC SCLEROSIS COHORT

K.K. Solanki^1^, P. Bardoul^2^, A. Soepnoel^3^, D. White^1^, M. Matucci-Cerinic^4^

^1^*Waikato Hospital, Hamilton, New Zealand*, ^2^*Dunedin School of Medicine, Dunedin, New Zealand*, ^3^*Immunology department laboratory services, Waikato District Health Board, Hamilton, New Zealand*, ^4^*Department of Experimental and Clinical Medicine, University of Florence & Division of Rheumatology AOUC, Florence, Italy*

**Introduction:** The serological hallmarks of SSc are autoantibodies targeted against a range of antigens, some of which are more specific to SSc than others, and commonly mutually exclusive to one another. However, until today a specific association among Ro52 autoantibodies and other clinical features remains controversial .

We aimed to identify and evaluate potential associations between the presence of isolated Ro52/TRIM21 antibody and other antibodies, as well as clinical manifestations in the Waikato hospital systemic sclerosis (SSc) cohort.

**Material and Methods:** A cohort of 152 patients that fulfilled the 2013 EULAR- ACR classification criteria for systemic sclerosis or systemic sclerosis overlap syndrome (SOS) have been prospectively followed for the period 2005-2018 by the Waikato Systemic Sclerosis clinic. Patient data were collaborated, and statistical analyses performed to determine associations present between anti-Ro52 and various clinical outcomes of interest.

**Results:** Ro52 antibodies were present in 22.4% of the Waikato SSc cohort. The presence of Ro52 was significantly associated with the presence of other SSc antibodies (p<0.001), in particular the anti-centromere antibodies. No association was found between Ro52 and any of clinical manifestations, including ILD and myositis. There was an association between Ro52 and SSA antibodies.

**Conclusions:** Our data show that Ro52/TRIM21 antibodies are a common finding in SSc patients. In our cohort, no significant associations between Ro52 positive patients and clinical manifestations were found. This included ILD and myositis which have previously reported having a significant relationship with R052 positivity in some studies only.

In our patients, Ro52 positivity was associated with multiple antibodies, and in particular with anticentromere antibodies. A significant relationship between Ro52 and Ro60 and immunoglobulin levels was detected.

Up-till today it remains an unsolved issue if Ro52 holds great potential as a marker of clinical outcome or disease evolution that need to be further investigated in a larger population cohort over a wider geographical distribution.

## P.010

## IS ANTI-DOUBLE-STRANDED, SINGLE-STRANDED DNA ANTI-C1Q ANTIBODY SEROPOSITIVITY A DISEASE MARKER IN PATIENTS WITH SYSTEMIC SCLEROSIS?

T. Simopoulou, E. Patrikiou, C. Liaskos, G. Efthymiou, L. Sakkas, D. Bogdanos

*Department of Rheumatology and clinical Immunology, University General Hospital of Larissa, Faculty of Medicine, School, Larissa, Greece*

**Introduction:** Co-occurrence of anti-C1q and anti-dsDNA appears to be associated with disease activity in patients with systemic lupus erythematosus (SLE). We have recently showed that anti-C1q autoAbs are frequently detected in patients with systemic sclerosis (SSc) and that their presence is associated with pulmonary fibrosis and pulmonary arterial hypertension.

Our study aim to assess whether anti-ssDNA and anti-dsDNA co-occurrence with anti-C1q in patients or autoantibody seropositivity in isolation is of clinical relevance in SSc.

**Material and Methods:** Serum samples from 100 consecutive patients with SSc (57 limited cutaneous SSc and 43 diffuse cutaneous SSc) were tested for anti-C1q, anti-ssDNA and anti-dsDNA antibodies by ELISA (Inova Inc).

**Results:** 17/99 (17%), 21/100 (21%), 8/100 (8%) were tested positive for the presence of anti-C1q, anti-ssDNA and anti-dsDNA respectively. Anti-ssDNA or anti-dsDNA positivity was not correlated with the type of the disease, sex, age, the presence of PF, PAH, serositis, gastrointestinal involvement, calcinosis, or arthritis with the exception of correlation of anti-ssDNA with the presence of telangiectasias (19/57; 33,3% vs 2/43; 4,6 %, p=000). Seven patients (7%) had double reactivity against ssDNA and C1q, while only 4 patients (4%) had double reactivity against dsDNA and C1q.

**Conclusions:** Anti-ssDNA and dsDNA are frequently found in patients with SSc, but not in conjunction with anti-C1q antibodies; their presence does not appear to be of particular clinical significance.

## P.011

## CD34 AND ALPHA-SMOOTH MUSCLE ACTIN DISTINGUISH SCLERODERMA GENE EXPRESSION SUBSETS USING A MACHINE LEARNING APPROACH

K. Showalter^1^, D. Orange^1,2^, C. Magro^3^, P. Agius^4^, V. Martyanov^5^, J. Franks^5^, R. Sharma^4^, H. Geiger^4^, T. Wood^5^, Y. Zhang^1^, R. Spiera^1^, M. Whitfield^5^, J. Gordon^1^

^1^*Hospital for Special Surgery, New York, USA*, ^2^*Rockefeller University, New York, USA*, ^3^*Weill Cornell Medicine, New York, USA*, ^4^*New York Genome Center, New York, USA*, ^5^*Geisel School of Medicine at Dartmouth, Hanover, USA*

**Introduction:** The purpose of this study is to identify whether histologic features distinguish gene expression subsets (normal-like, fibroproliferative, inflammatory) in diffuse systemic sclerosis (SSc; scleroderma).

**Material and Methods:** 58 skin biopsies from 26 patients with diffuse SSc (median disease duration 0.8y) were assessed by physical exam, microarray, and histology. Samples were assigned to gene expression subsets using multinomial elastic net supervised classifier (GLMnet). A dermatopathologist scored seven histology features (inflammatory infiltrates, collagen density, global severity, thickness (epidermis—subcutis), follicle count and two dermal fibroblast markers, semi-quantitatively (0—3): CD34 and alpha smooth muscle actin (aSMA). Kruskal-Wallis was used to determine differences in median histology scores across subsets. Support vector machine learning was performed using subset assignments as classifiers and histology scores as inputs. Area under the curve (AUC) of the receiver operating curves assessed algorithm performance. Mean absolute weight for each binary histology feature was determined using ‘w-vector’ to identify histologic features most predictive of subset assignment.

**Results:** 24, 16 and 18 samples were assigned to normal-like, fibroproliferative or inflammatory gene expression subsets, respectively. Skin biopsy sites from normal-like, fibroproliferative or inflammatory samples had median local modified Rodnan skin score of 1, 2, 3; respectively, (p=0.001). Erythrocyte sedimentation rate (mm/hr) was lowest in normal-like compared to fibroproliferative and inflammatory samples (5, 10, 12.5, respectively; p=0.02). Using binarized histology features as inputs, the initial machine learning algorithm AUC values for predicting inflammatory, normal-like and fibroproliferative gene expression subsets were 0.72, 0.66, and 0.52, respectively. The histology features with the strongest predictive values (highest mean weight) were CD34, followed by aSMA, thickness, and global severity.

CD34 staining was highest in normal-like compared to fibroproliferative and inflammatory samples (2, 1, 0.5, respectively; p<0.001). Conversely, aSMA staining was lowest in normal-like compared to fibroproliferative and inflammatory samples (0.5, 0.75, 2, respectively; p=0.02). There were also significant differences in global severity (1.5, 2.25, 2, respectively; p=0.01), and collagen density (1, 2, 2.5, respectively; p=0.01). In subsequent machine learning models, using only CD34 as input predicted fibroproliferative subsets (AUC 0.77), while using only aSMA predicted inflammatory subsets (AUC 0.73).

**Conclusions:** Our results extend upon previous studies that identified SSc gene expression subsets by identifying associated histology features. Using a machine learning approach, we identify CD34 as an informative biomarker of SSc heterogeneity and suggest that dermal fibroblast polarization between CD34 and aSMA expression distinguishes scleroderma skin gene expression subsets.

## P.012

## OCCURRENCE OF DIFFERENT ANTINUCLEAR ANTIBODIES IN PATIENTS WITH SYSTEMIC SCLEROSIS POSITIVE FOR ANTI-U1RNP

R. Shayashmetova, L. Ananieva, O. Koneva, M. Starovoitova, O. Desinova, O. Ovsyannikova, L. Garzanova

*V.A.Nasonova Research Institute of Rheumatology, Moscow, Russia*

**Introduction:** Patients with systemic sclerosis positive for anti-U1RNP have special clinical picture and disease progression. The autoimmune profile in this group is poorly understood. The purpose of our work was to study the level of major autoantibodies in patients with systemic sclerosis positive for anti-U1RNP.

**Material and Methods:** The study included 80 patients (71 women and 9 men, mean age 44,5±14 years) positive for antibodies to RNP and meeting the criteria of the systemic sclerosis (ACR/EULAR 2013). Patients were examined for autoantibodies: RF, ACCP, ACA, anti-Scl70, anti-RNAP-III, anti-Ro, anti-La, anti-dsDNA, anti-Sm, ACL, anti-Jo1. 44 patients were examined in dynamics in 24 months.

**Results:** In the study group the clinical picture was dominated by inflammatory musculoskeletal lesions (synovitis and myopathy), skin manifestations were poorly expressed. Interstitial lung disease was detected in 68% of cases. Overlaps (34%) with other rheumatic diseases (rheumatoid arthritis, systemic lupus erythematosus) and combination with Sjogren’s syndrome (32.5%) were frequently noted. Other antibodies were often detected: commonly - RF (31%), anti-Ro (38%), anti-dsDNA (42%), rarely - anti-Sm (11%), ACCP (8%), anti-La (8%), ACA (6%), anti-Scl70 (6%), AKL (2%). Anti-Jo1 and anti-RNAP-III were not detected at all. In patients with systemic sclerosis highly-positive for anti-U1RNP (more than 2 upper normal limits) RF, anti-Ro, anti-dsDNA were significantly more common in comparison with low-positive(p=0.00).

In dynamics 80% of patients maintained anti-U1RNP, while other autoantibodies were detected with the same frequency. In patients with initially low titer of anti-U1RNP, their disappearance was noted.

**Conclusions:** Patients with systemic sclerosis positive for anti-U1RNP differ in the predominance of inflammatory musculoskeletal manifestations and frequent combination with Sjogren’s syndrome and overlaps. Highly positivity for anti-U1RNP is accompanied by a persistent increase in RF, anti-Ro, anti-dsDNA.

## P.013

## NOTCH SIGNALING IN MICROVASCULAR ENDOTHELIAL CELLS IS MODULATED BY A SERUM FACTOR IN SYSTEMIC SCLEROSIS

F. Seguro Paula^1^, J. Delgado Alves^1,2^

^1^*Immune Response and Vascular Disease, CEDOC - Chronic Diseases Research Center, NOVA Medical School, Lisbon, Portugal*, ^2^*UDIMS - Systemic Immune-mediated Diseases Unit, Internal Medicine Department 4, Fernando Fonseca Hospital, Amadora, Portugal*

**Introduction:** Systemic sclerosis (SSc) is characterized by endothelial dysfunction, fibrosis and autoimmunity. Capillary rarefaction, dilatations, microhaemorrhages, and dysangiogenesis are observed since the early stages of the disease, with high but inefficient VEGF levels. The Notch pathway is increasingly recognized as a major player in neoangiogenesis and vascular maintenance, being closely linked with VEGF-signalling. Notch activation status in the endothelium of SSc is unknown. The aim of this study is to evaluate the role of SSc-specific humoral factors in modulating Notch signalling on endothelial cells, and its functional implications in neoangiogenesis and vascular maintenance.

**Material and Methods:** In vitro serum assays were performed with microvascular endothelial cells (MVECs) exposed to serum from 22 SSc patients and 10 age-matched healthy controls. Gene expression analysis for several Notch target-genes was undertaken, comparing the effects of SSc serum to control serum. Results were compared with known patterns of gene expression related to Notch pathway activation, and with internal positive controls for an activated Notch pathway. Protein analysis was performed by western blot to evaluate such effects at the protein level. Functional in vitro assays for angiogenesis were done to assess the functional repercussion of SSc-serum exposure to MVECs.

**Results:** MVECs exposure to SSc-derived serum resulted in an overall gene and protein expression patterns consistent with an activated Notch pathway, which results in a deranged in vitro neoangiogenesis. Notch pathway modulation can partially reverse such changes.

**Conclusions:** These results suggest that the Notch pathway could be abnormally activated in the microvascular endothelium of SSc by a serum factor. Such a pattern of modulation of the Notch pathway results in functional derangements of endothelial function, namely of neoangiogenesis. Overall, these findings might provide a new pathophysiologic mechanism in SSc. Pharmacological reversal of such an effect could prove beneficial, providing a new therapeutic target in treating SSc.

## P.014

## SPECIFIC AUTOANTIBODIES IN SYSTEMIC SCLEROSIS AND ITS CORRELATION WITH CLINICAL MANIFESTATION IN WEST JAVA INDONESIA

W.A.M. Saragih^1^, S. Dewi^2^, R.G. Wachjudi^2^, V. Logito^3^, A. Tjandrawati^3^

^1^*Department of Internal Medicine, Faculty of Medicine, Padjadjaran University University, Bandung, Indonesia*, ^2^*Immunology Study Center, Padjadjaran University, Bandung, Indonesia*, ^3^*Department of Clinical Pathology, Faculty of Medicine, Padjadjaran University University, Bandung, Indonesia*

**Introduction:** Systemic sclerosis (SSc) is a chronic inflammatory autoimmune disease, characterized by fibrosis of the skin and internal organ, whose main hint toward autoimmunity is given by the presence of high serum levels of autoantibodies in the majority of the patients. Some of the autoantibodies present an interesting association with clinical features of SSc. The identification of autoantibodies specific SSc help in diagnosis and predicting clinical phenotype. The purpose of this study is to give information regarding the pattern of autoantibodies specific SSc and its correlation with clinical manifestations.

**Material and Methods:** This is descriptive quantitative study, which autoantibodies specific test using Euroline immunoblot assay. Data was collected from medical records of outpatients visiting Rheumatology Clinic Dr. Hasan Sadikin General Hospital from 1 January 2018 until 30 August 2019.

**Results:** A total of 34 patients were enrolled in this study, 33 female and 1 male, median age 42 (21-65 years old). ANA by indirect immunofluorescence was present in 33 (97.8%) patients. Mean onset of disease until diagnose is 9 months. 29 (85.3%) patients with diffuse cutaneous SSc (85.3%) and 5 (14.7%) patients with limited cutaneous SSc. We found anti topo I in 30 (83.3%) patients, anti-Ro-52 in 10 (27.8%) patients, anti-RNA polymerase III(RP155) in 6 (16.7%) patients, anti-fibrillarin in 5 (13.8%) patients, anti-Th/To in 4 (11.1%) patients, anti centromere protein (anti-CENPA) in 3 (8.3%) patients, anti centromere protein (anti-CENPB) in 3 (8.3%) patients, anti-Ku in 2 (5.5%) patients, anti-NOR-90 in 2 (5.5%) patients and anti-PM-Scl-75 in 1 (2.2%) patients. Anti-RP-11, Anti-PM-Scl-100 and Anti-PDGFR was negative in testing. Stiffness of the skin and arthalgia found in all patients. Raynaud’s phenomenon in 30 (90.9%) patients, fingertip lesion in 28 (84.8%) patients. Gastrointestinal involvements in 10 (30.3%) patients. Renal involvement found in 15 (45.4%) patients. Pulmonary involvements found in 30 (90.9%) patients, acknowledged from clinical symptoms such as dyspnea and with functional lung test showing abnormality, with 27 (79.4%) confirmed lung fibrosis by HRCT scan.

**Conclusions:** Anti-Topo I, Anti-Ro-52 and Anti-fibrillarin are the most prevalent autoantibody in SSc. Most of systemic sclerosis have cutaneous and pulmonary involvement, diffuse type and women is more dominant.

## P.015

## DISSECTING THE ROLE OF IL-25 IN KERATINOCYTE-FIBROBLAST CROSSTALK IN SYSTEMIC SCLEROSIS

B. Russo, J. Borowczyk-Michalowska, P. Cacialli, N. Brembilla, C. Chizzolini

*Pathology & Immunology, School of Medicine, University of Geneva, Switzerland, Geneva, Switzerland*

**Introduction:** Skin fibrosis is a hallmark of systemic sclerosis (SSc). Recent evidence suggests that keratinocytes are altered in SSc and may influence the functional behavior of fibroblasts. Cytokines known to target keratinocytes, in particular those of the IL-17 family are increased in SSc skin. IL-25, a member of the IL-17 family, has been recognized to be a potential therapeutic target in models of fibrosis. It has been shown that the number of IL-25 positive cells is increased in scleroderma dermis.

Our aim is to investigate the expression of IL-25 in SSc epidermidis and its role in the regulation of keratinocyte-fibroblast crosstalk.

**Material and Methods:** Skin biopsies were obtained from 10 SSc patients and 8 healthy donors (HD). The skin expression of IL-25 was evaluated by chromogenic in situ hybridization and immunofluorescence. Primary keratinocytes were generated from 8 HD and primed in vitro with IL-25. Their conditioned medium was used to stimulate fibroblasts. IL-6, IL-8, MMP-1, type-I collagen (col-I), and fibronectin production was assessed by ELISA

**Results:** In situ hybridization revealed that keratinocytes were the only source of IL-25 in the skin. Compared to HD, IL-25-staining in SSc epidermis was fainter and showed a different pattern of distribution, with lower expression in the more differentiated keratinocytes. The supernatant of HD keratinocytes enhanced the production of MMP-1, IL-6, IL-8 (p<0.05), but not of col-I or fibronectin when applied to HD fibroblasts. The priming of HD keratinocytes by IL-25 further enhanced the production of these mediators. This effect was not due to IL-25 carryover, nor to direct production by keratinocyte. Neutralization experiments indicated that IL-1 was, at least in part, responsible for the IL-25 modulation of keratinocyte-dependent fibroblasts activation. When fibroblasts were directly exposed to IL-25 their production of col-I was reduced in a dose- dependent manner. Matrix turnover, as determined by the col-I to MMP-1 ratio was increased in the presence of IL-25

**Conclusions:** Our preliminary data demonstrate an altered expression of IL-25 in SSc epidermis. We further confirm that keratinocytes enhance dermal fibroblasts responses and show for the first time that IL-25 potently modulates this activity. Interestingly, we observed that IL- 25 is implicated in collagen turn-over either through direct effect on fibroblasts either through its effect on keratinocytes-fibroblast cross-talk. Investigations are planned to better elucidate the role of IL-25 using SSc keratinocytes in 3D culture conditions

## P.016

## CD25+ FOXP3+ REGULATORY B CELLS FUNCTION IN SYSTEMIC SCLEROSIS

D. Rimar^1^, G. Slobodin^1^, I. Rosner^1^, M. Rozenbaum^1^, E. Nasrin^2^, Z. Vadasz^2^

^1^*Rheumatology Unit, Bnai Zion Medical Center, Haifa, Israel*, ^2^*Division of clinical Immunology, Bnai Zion Medical Center, Haifa, Israel*

**Introduction:** Background: Regulatory B cells (Bregs) have been recently suggested to plays a regulatory role in Systemic sclerosis (SSc). CD 25+ Bregs are particularly similar to Tregs and have never been evaluated in SSc.

**Aim:** To investigate the percentage and function of CD25+ IL-10+ Bregs in SSc, and to correlate it with clinical and demographic characteristics of SSc patients in compare with healthy controls.

**Material and Methods:** Purified B cells collected from Ssc patients and from healthy controls were activated for 48H. RNA was extracted from each culture and was subjected for RQ-PCR with specific primers for CTLA-4, FOXP3 and CD3- in order to exclude T cells presence in the culture. These cells were analyzed for CD19, CD25, CTLA-4, sema3A, and IL-10 expression using Flow cytometry. SSc patients were evaluated for demographics, clinical manifestations, routine laboratory results, nailfold videocapillaroscopy, pulmonary function tests, echocardiograms, modified Rodnan skin score (mRSS) and disease activity and severity scores.

**Results:** 24 SSc women at the mean age of 56.5± 10.8 years were enrolled. Percent of C25+Bregs was similar between SSc patients and matched healthy controls, 3.89%±1.2 vs. 4.39%±0.86. CTLA4, IL-10 and sempahorin3A expression on Bregs, however, were significantly higher in SSC vs. healthy controls, 21.00 ± 1.204, 59.11 ± 2.241, 28.81 ± 1.324 vs. 7.930 ± 0.9930, 37.47 ± 2.384, 20.45 ± 1.252, p value<0.0001. RQ-PCR confirmed and validated that these cells were strictly Regulatory B cells. There was no correlation of mRSS, lung function tests, disease activity or severity, lung fibrosis, diffuse disease or type of antibodies with Bregs number or function.

**Conclusions:** This is the first study to show that CD 25+, IL-10+ FOXP3+ Bregs are highly active in SSc and express CTLA4 which was up to now considered to be an exclusive marker of regulatory T cells.

**Figure:**
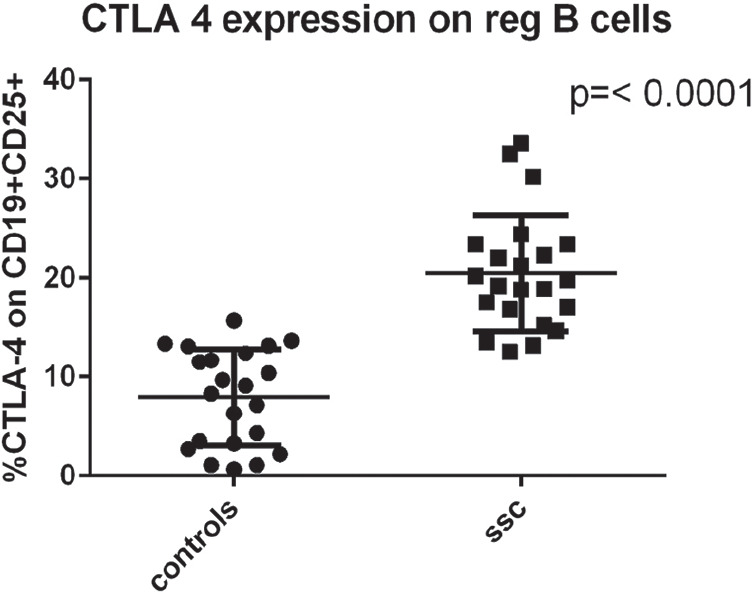


## P.017

## IS IGG4 AN IMMUNO-PROTECTIVE ANTIBODY? LESSONS FROM SYSTEMIC SCLEROSIS

N. Rabinowicz^1^, A. Ben Ari^2^, H. Paran^2,3^, Y. Levy^1,3^

^1^*Meir Medical Center, Department of Internal Medicine E, Kfar Saba, Israel*, ^2^*Meir Medical Center, Department of Surgery, Kfar Saba, Israel*, ^3^*Tel Aviv University, Sackler Faculty of Medicine, Tel Aviv, Israel*

**Introduction:** IgG4 is a unique immunoglobulin, different from other members of the immunoglobulin family by structure, affinity to its receptors and inability to activate the complement system. Its levels in the sera are low - 5% of all IgG, therefore it is considered of little significance in healthy people. However, there are studies showing its pathogenic role - for example, in IgG4-related disease where high IgG4 levels are present in most patients, and on the contrary- some studies show that IgG4 has some anti-inflammatory properties.

Systemic sclerosis (SSc) is an autoimmune disease of the connective tissue, characterized by thickening of the skin due to accumulation of collagen and by damage to small arteries.

Our objective was to determine whether IgG4 antibodies are related to immuno-protection, by comparing their levels in SSc patients to healthy subjects.

**Material and Methods:** IgG4 levels were obtained from medical records of 80 SSc patients between the years 2000-2019. These were compared to IgG4 levels from sera of 80 healthy donors (from Israel blood-bank - MDA). Correlations between IgG4 levels and the following were made: age, sex, disease type (limited or diffused SSc), organs involved, disease duration, medications.

**Results:** Compared to healthy subjects, IgG4 levels were significantly lower among SSc patients. Other findings will be described in the poster. Lower IgG4 levels were correlated with presence of Raynaud’s phenomenon, while higher IgG4 levels were measured among patients treated with Actemra. In both groups, there was a negative correlation between age and IgG4 levels.

**Conclusions:** IgG4 levels are lower in systemic sclerosis patients. Whether this finding is involved in the etiology of the disease, related to the biologic pathway of systemic sclerosis, or a general manifestation common among other autoimmune diseases, requires further research.

## P.018

## EVALUATION OF MEMBRANE-BOUND AND SOLUBLE FORMS OF HUMAN LEUCOCYTE ANTIGEN G (HLA-G) IN SYSTEMIC SCLEROSIS

P. Contini, S. Negrini, G. Murdaca, M. Greco, F. Puppo

*Department of Internal Medicine, University of Genova, Genova, Italy*

**Introduction:** Systemic sclerosis (SSc) is characterized by immune dysregulation, vascularopathy and fibrosis. Human leucocyte antigen G (HLA-G) is a non-classic class I major histocompatibility complex (MHC) molecule with immunomodulating properties expressed on the membrane of different cell lineages. HLA-G is also detectable in soluble form (sHLA-G) deriving from the shedding of surface isoforms (sHLA-G1) or the secretion of soluble isoforms (HLA-G5).

**Material and Methods:** Thirty-five patients (28 females and 7 males, aged 40–89 years) were enrolled. Forty healthy subjects, matched for sex and age, were recruited as controls. The expression of cell membrane antigens by peripheral blood mononuclear cells (PBMC) was analysed by flow cytometry. The plasma level of sHLA-G and TGF-beta was determined by sandwich immunoenzymatic assays.

**Results:** The percentage of HLA-G positive monocytes (0.98 ± 1.72), CD4+ (0.37 ± 0.68), CD8+ (2.05 ± 3.74) and CD4+CD8+ double-positive cells (14.53 ± 16.88) was higher in SSc patients than in controls (0.11 ± 0.08, 0.01 ± 0.01, 0.01 ± 0.01 and 0.39 ± 0.40, respectively) (P < 0.0001). The levels of sHLA-G and TGF-beta were higher in SSc patients (444.2 ± 304.8 U/ml and 18937 ± 15217 pg/ml) compared to controls (16.7 ± 20.5 U/ml and 11099 ± 6081 pg/ml) (P < 0.0001 and P < 0.003, respectively). In SSc patients, the levels of sHLA-G1 and HLA-G5 isoforms were comparable (264.6 ± 226.9 U/ ml and 181.4 ± 130.1 U/ml, respectively) A significant correlation was found between TGF-beta and the plasma levels of total sHLA-G (r = 0.65; P < 0.01), sHLA-G1 (r = 0.60; P = 0.003) and HLA-G5 (r = 0.47; P = 0.02).

**Conclusions:** These data indicate that in SSc the secretion and/or shedding of soluble HLA-G molecules and the membrane expression of HLA-G by peripheral blood mononuclear cells (PBMC) are clearly elevated, suggesting an involvement of HLA-G molecules in the immune dysregulation of SSc.

## P.019

## ANTI-IFI-16 AUTOANTIBODIES IN SYSTEMIC SCLEROSIS PATIENTS ARE ASSOCIATED WITH A POORER PROGNOSIS IN OVERALL SURVIVAL

J. Perurena-Prieto^1^, E.L. Callejas-Moraga^2^, A. Guillén-Del-Castillo^3^, L. Viñas-Gimenez^1^, A. Marín-Sánchez^1^, V. Fonollosa-Plá^3^, M.T. Sanz-Martinez^1^, C.P. Simeón-Aznar^3^

^1^*Department of Immunology, Vall D’Hebron University Hospital, Barcelona, Spain*, ^2^*Systemic Autoimmune Diseases Unit, Department of Internal Medicine, Parc Taulí University Hospital, Sabadell, Spain*, ^3^*Systemic Autoimmune Diseases Unit, Department of Internal Medicine, Vall D’Hebron University Hospital, Barcelona, Spain*

**Introduction:** It is widely recognized that the presence of some specific auto-antibodies defines the clinical presentation, cumulative manifestations and prognosis of Systemic Sclerosis (SSc) patients. Interferon gamma-inducible protein 16 (IFI16) is a member of the HIN200 gene family, which encodes innate pattern recognition receptors that sense double-stranded DNA. Auto-antibodies against IFI16 have been reported with a prevalence ranging from 21-33% in SSc. The objective of this study was to assess the prevalence of anti-IFI16 autoantibodies in the SSc cohort of the Vall d’Hebron University Hospital and to evaluate the clinical significance of these autoantibodies, isolated or in presence of anti-centromere antibodies (ACA).

**Material and Methods:** In total, 124 clinically established SSc serum samples from the Vall d’Hebron University Hospital were tested for anti-IFI-16 antibody by a direct “in-house” ELISA. An arbitrary serum was included as a reference in every plate and all absorbances were calibrated relative to this reference absorbance. The cut-off level was assigned at 2 standard deviations higher than the mean value of calibrated absorbances of 52 healthy controls. 66 Samples were positive for anti-centromere antibodies by indirect immunofluorescence using HEp-2 cells as substrates. 58 samples were negative for antibodies against Scl-70, CENP-A, CENP-B, RP11, RP155, fibrillarin, NOR-90, Th/To, Pm-Scl100, Pm-Sc75, Ku and PDGFR tested by LIA.

**Results:** Overall, 29.0% (n = 36) of the tested samples were positive for anti-IFI-16 antibodies. In the case of ACA positive patients, 39.4% (n = 26) of them were also positive for anti-IFI-16. In the other hand, 17.2% (n = 10) of the samples that were negative for all the tested auto-antibodies were positive for anti-IFI-16. Anti-IFI-16 antibodies were found only in patients within the limited cutaneous SSc subset. Higher titers of anti-IFI-16 antibodies correlated with a more severe peripheric vasculopathy (digital ulcers) (p=0.03). The relative risk of death for an anti-IFI-16 positive patient was 3.9 times that for an anti-IFI-16 negative patient. Kaplan-Meier with Mantel-Cox log-rank analysis showed that Anti-IFI-16 positive patients presented a poorer prognosis in overall survival (p = 0,032).

**Conclusions:** Anti-IFI-16 antibodies have a high prevalence in ACA positive SSc patients. In a high number of samples anti-IFI-16 antibody was the only SSc-associated antibody that could be identified. Anti-IFI-16 antibodies should be evaluated in these two groups of SSc patients since they were associated with a poorer prognosis in overall survival and also with a more severe peripheric vasculopathy.

## P.020

## INKT CELLS MODULATE REGULATORY AND EFFECTOR B CELLS OF SSC PATIENTS

E. Asteriti, H. Keppeler, S. Duerr-Stoerzer, H. Schmid, K.-A. Secker, A.-C. Pecher, J. Henes, R. Klein, C. Schneidawind, D. Schneidawind

*Universitätsklinikum Tübingen, Tuebingen, Germany*

**Introduction:** Systemic sclerosis (SSc) is an autoimmune disease characterized by a breach of immune tolerance towards endogenous antigens, leading to vasculopathy and progressive fibrosis of the skin and internal organs. The production of autoantibodies implicates a significant role of B cells in SSc pathogenesis. Invariant natural killer T (iNKT) cells are potent immunoregulatory T lymphocytes that are critical for maintaining immune tolerance by influencing other immune cells. Various studies suggest cognate interactions of iNKT cells with B cells shaping B-cell homeostasis. Our group has previously shown that SSc patients contain only very low iNKT-cell numbers in peripheral blood. Therefore, the purpose of this study was to examine the influence of culture-expanded human iNKT cells on B-cell phenotype and function of SSc patients.

**Material and Methods:** iNKT cells from healthy donors were expanded ex vivo following a two-week protocol using rhIL-2 and alpha-GalCer. Such culture-expanded iNKT cells were further enriched by magneticactivated cell sorting (MACS) to a purity >95%. B cells from healthy donors and SSc patients were also isolated using CD19 magnetic beads (purity >95%).

**Results:** To analyze effector and regulatory B cells in healthy volunteers and SSc patients, PBMCs were cultured in presence of LPS and CD40L. In SSc patients CD24+CD27+ regulatory B cells were significantly decreased compared to healthy controls. In addition, levels of IL-6 were increased while levels of IL-10 were decreased in SSc patients compared to healthy controls. This IL-6 bias could reflect the dysregulated immune homeostasis in SSc patients. We hypothesized that iNKT cells might restore a tolerogenic cytokine profile. Coculture of purified B cells from SSc patients with culture-expanded iNKT cells resulted in a significant decrease of IL-6 release measured by ELISA. Importantly, addition of iNKT cells did not change B-cell numbers.

**Conclusions:** In conclusion, culture-expanded iNKT cells are able to regulate B-cell homeostasis of SSc patients. Therefore, adoptive transfer of ex vivo expanded iNKT cells could restore immune tolerance in SSc patients with impaired iNKT-cell function.

## P.021

## DEFECTIVE DNA DAMAGE RESPONSE AND REPAIR NETWORK IN PATIENTS WITH SYSTEMIC SCLEROSIS

M. Pappa^1^, P.I. Ntouros^1^, N. Vlachogiannis^1^, S. Panopoulos^1^, V.-K. Bournia^1^, V. Souliotis^1,2^, P. Sfikakis^1^

^1^*Joint Rheumatology Program, Medical School, National Kapodistrian University of Athens, Athens, Greece*, ^2^*Institute of Chemical Biology, National Hellenic Research Foundation, Athens, Greece*

**Introduction:** The DNA Damage Response and Repair (DDR-R) network is a comprehensive signalling process that determines the cell’s ability to repair DNA damage or to undergo apoptosis. Previous studies have shown that increased DNA damage is present in patients with systemic autoimmune diseases, including Systemic Sclerosis (SSc). Herein, we tested the hypothesis that DDR-R network is defective in these patients.

**Material and Methods:** Peripheral blood mononuclear cells (PBMCs) were isolated from 27 SSc patients (aged 53.4±13.3, disease duration of 1-34 years), 7 patients with Very Early Diagnosis of Systemic Sclerosis (VEDOSS), 70 apparently healthy control subjects (HC; aged 37.5±15) and 30 SLE patients (aged 40.9±14.8), who served as disease controls. Endogenous DNA damage [Single-Strand breaks (SSBs) and Double-Strand Breaks (DSBs)] in PBMCs was measured by alkaline comet assay and specific markers for SSBs (RPA32) and DSBs (γH2Ax, 53BP1) were measured using western-blot. Formation of DNA damage was assessed by oxidative stress and abasic sites measurements. Transcription-Coupled Repair (TCR) and Global Genome Repair (GGR), subpathways of Nucleotide Excision Repair, as well as chromatin organisation, were assessed along the N-ras gene by Southern-blot analysis. DSBs repair was measured using immunofluorescence γH2AX staining and confocal laser scanning microscope analysis. Expression levels of 84 DDR-R-associated genes were also studied.

**Results:** Endogenous DNA damage levels in SSc was increased by almost 3-fold [Olive Tail Moment units: 12.4±5.9) versus HC (4.5±2.3); similar results were obtained when 19 patients were matched 1:1 for age/gender with HC, whereas there was no significant difference between SSc and SLE (8.4±8.3). Individual levels of DNA damage correlated significantly with Modified Rodnan Skin Score, presence of pulmonary arterial hypertension and of digital ulcers, being within normal limits in VEDOSS. Increased DNA damage was confirmed using RPA32, γH2Ax, and 53BP1 markers. Higher levels of oxidative stress and abasic sites were present in SSc than HC. A more condensed chromatin organisation, defective GGR, but not TCR, and defective DSB repair mechanisms were also present in SSc. Accordingly, 4 genes involved in DNA repair mechanisms (MRE11A, PARP1, PRKDC and XRCC1) were significantly underexpressed, whereas BAX and BBC3 genes implicated in apoptosis were significantly overexpressed.

**Conclusions:** Increased endogenous DNA damage that correlates with clinical disease burden in SSc is possibly due to augmented damage formation and defects in DNA repair mechanisms which are epigenetically regulated. Further studies to uncover a potentially crucial role of these defects in the SSc pathogenesis are warranted.

## P.022

## SKIN SINGLE-CELL RNA SEQUENCING AND TARGET VALIDATION IN SYSTEMIC SCLEROSIS AND SCLEROTIC GVHD

I. Odell, R. Flavell

*Yale University, New Haven, USA*

**Introduction:** Scleroderma (Systemic sclerosis) is a rare autoimmune disease that leads to excess production of collagen and other matrix proteins, which generates scar-like tissue (fibrosis) in the skin, lungs and other organs. The effector cells that produce fibrotic tissue are fibroblasts and pericytes. Recently, it has been increasingly appreciated that immune cells and their associated cytokines can drive the excess matrix production by effector cells. Graft-vs-host disease, a major complication of stem cell transplant, can also cause overproduction of matrix proteins and scarring leading to similar problems as in scleroderma. Therefore, we hypothesized that scleroderma and the sclerotic form of graft-vs-host disease have overlapping disease pathogenesis.

**Material and Methods:** To examine new pathways and cell-cell interactions that drive fibrosis, we performed single-cell RNA sequencing from skin biopsies from patients with diffuse scleroderma and sclerotic graft-vs-host disease compared to normal controls. We compared our data to previously published data from patients with scleroderma-associated interstitial lung disease. We validated the findings from patients using immunohistochemistry and qRT-PCR, performed mechanistic studies in vitro with patient-derived fibroblasts and pericytes, functional studies with knock-out mice strains and pre-clinical models with humanized mice.

**Results:** We discovered increased expression of both known and novel growth factors and their associated receptors by immune cells, fibroblasts and pericytes. One subset of ligands and their receptor were increased in our skin samples from patients with scleroderma or graft-vs-host disease as well as independently validated in sequencing data from patients with scleroderma interstitial lung disease. We confirmed that the RNA expression data corresponded to increased number of ligand-positive cells and increased receptor phosphorylation in biopsy specimens. We found that inhibition of the most upregulated ligand and its receptor in cultured fibroblasts decreased the expression of the extracellular matrix genes identified in the single-cell RNA sequencing. In mice, inhibition of the receptor with a small molecule inhibitor prevented the development of bleomycin-induced fibrosis. In knock-out mice, we found that there was significant functional redundancy by the family of ligands and that inhibition of multiple ligands was required to prevent fibrosis.

**Conclusions:** These findings show that inhibition of immune cell-derived growth factors or their cognate receptor are likely an effective treatment strategy in patients with scleroderma or graft-vs-host disease and warrants clinical trials with inhibitors of this ligand and receptor family.

## P.023

## CLINICAL CHARACTERISTICS OF SYSTEMIC SCLEROSIS (SSC) PATIENTS NEGATIVE FOR SSC-RELATED AUTOANTIBODIES

M. Miyake, Y. Hamaguchi, T. Matsushita, K. Takehara

*Department of Dermatology, Kanazawa University, Kanazawa, Japan*

**Introduction:** In systemic sclerosis (SSc), anti-nuclear antibody (ANA) is positive in more than 90% of patients and 11 SSc-related autoantibodies (autoAbs) have been identified. Identification of SSc-related autoAbs is clinically beneficial because each SSc-related autoAb is closely associated with specific clinical characteristics. However, a percentage of SSc patients are negative for SSc-related autoAbs and their clinical features have not been fully clarified. In this study, we examined the clinical characteristics of SSc patients negative for SSc-related autoAbs.

**Material and Methods:** Serum samples were collected from 546 SSc patients. The presence of ANA was screened by indirect immunofluorescence (IIF) staining using HEp-2 cells. Anti-centromere Ab (ACA) and anti-centriole Ab were identified by specific staining patterns on IIF staining. Enzyme-linked immunosorbent assay or immunoprecipitation assay was used to identify SSc-related autoAbs against topoisomerase I (topo I), RNA polymerase (RNAP), U3RNP, Th/To, hUBF, U1RNP, Ku, and RuvBL1/2. Clinical characteristics were analyzed among patients negative for ANA/SSc-related autoAbs, ACA, anti-topo I Ab, and anti-RNAP Ab.

**Results:** Of the 546 SSc patients, 26 (4.8%) were negative for ANA and 29 (5.3%) were ANA-positive, but negative for SSc-related autoAbs. Regarding clinical characteristics, patients negative for ANA/SSc-related autoAbs (n = 55) had a significantly shorter disease duration (2 [0.1-33] vs 8 [0.1-50], years), higher proportion of the diffuse form (56% vs 11%), contracture of phalanges (42% vs 17%), diffuse pigmentation (42% vs 16%), higher modified Rodnan total skin thickness score (mRSS) (10 [0-43] vs 2 [0-31]), and lower incidence of telangiectasia (14% vs 42%) than those with ACA (n = 224). On the other hand, younger disease onset (48 [8-76] vs 61 [20-81], years), lower mRSS (10 [0-43] vs 18 [0-49]), and lower incidence of scleroderma renal crisis (0% vs 13%) were observed in patients negative for ANA/SSc-related autoAbs than in those with anti-RNP Ab (n = 52). Although pitting scars were less common in patients negative for ANA/SSc-related autoAbs (15%) than those with anti-topo I Ab (48%) (n = 144), their clinical features were similar.

**Conclusions:** Therefore, patients negative for ANA/SSc-related autoAbs form a clinically distinct subset among SSc patients.

## P.024

## ROLE OF EFFECTOR AND REGULATORY B CELLS IN PATIENTS WITH SYSTEMIC SCLEROSIS: IL-6 PRODUCING EFFECTOR B CELLS ASSOCIATED WITH SKIN FIBROSIS

T. Matsushita^1^, T. Kobayashi^1^, M. Kano^1^, Y. Hamaguchi^1^, M. Hasegawa^2^, M. Fujimoto^3^, K. Takehara^1^

^1^*Kanazawa University, Kanazawa, Japan*, ^2^*University of Fukui, Fukui, Japan*, ^3^*Osaka University, Suita, Japan*

**Introduction:** Systemic sclerosis (SSc) is an autoimmune disease characterized by skin and lung fibrosis. Over 90% of the patients with SSc are positive for autoantibodies. In addition, the serum levels of BAFF, a potent B cell stimulator, are correlated with SSc severity and activity. Thus, B cells play an important role in SSc pathogenesis. However, two opposing B cell subsets exist: effector and regulatory B cells. IL-6 producing effector B cells promote scleroderma mouse model, whereas, IL-10 producing regulatory B cells inhibit it. In the present study, we have investigated the clinical association of effector and regulatory B cells in patients with SSc.

**Material and Methods:** The blood levels of IL-6 producing effector B cells and IL-10 producing regulatory B cells were measured in 29 patients with SSc and 16 healthy subjects by FACS.

**Results:** The frequency of IL-6 producing effector B cells in blood was significantly elevated in patients with SSc (55.3 ± 12.1%) than that in healthy controls (41.9 ± 10.6%, P<0.001). In contrast, the frequency of IL-10 producing regulatory B cells in blood was significantly decreased in patients with SSc (1.4 ± 0.7%) than in healthy controls (2.0 ± 0.8%, P<0.01). With respect to SSc subtypes, the frequency of IL-6 producing effector B cells was significantly higher in patients with diffuse cutaneous SSc (severe form of SSc) than that in patients with limited cutaneous SSc (mild form of SSc). Furthermore, the frequency of IL-6 producing effector B cells positively correlated with the extent of skin fibrosis in SSc patients.

**Conclusions:** The result suggested that the dysregulation of effector and regulatory B cell balance contributes to SSc pathogenesis.

## P.025

## CORRELATIONS BETWEEN NEUTROPHILS/LYMPHOCYTES RATIO AND PLATELETS/LYMPHOCYTES RATIO IN CLINICAL CHARACTERISTICS OF PATIENTS WITH SYSTEMIC SCLEROSIS

F. Masini^1^, F. Guarino^1^, K. Gjeloshi^1^, F. Danzo^1^, E. Pinotti^1^, R. Ferrara^1^, M. Tardugno^1^, L. Maresca^1^, C.P. Romano^1^, G. Cuomo^2^

^1^ *Dipartimento di Scienze Mediche e Chirurgiche Avanzate, Università degli studi della Campania L. Vanvitelli, Napoli, Italy, ^2^Dipartimento di Medicina di precisione, Università degli studi della Campania L. Vanvitelli, Napoli, Italy*

**Introduction:** Actually, neutrophils to lymphocytes ratio (NRL) and plateles/lymphocytes ratio (PLR) are considered novel inflammatory markers. Recently studies are ongoing to establish objectives and easy obtainable markers (1).

In this retrospective study, we aimed to assess the potential relationship between NRL and PLR with clinical characteristics of patients with systemic sclerosis (SSc).

**Material and Methods:** We consecutively enrolled fifty-two patients with SSc, mostly females (46; 10 subset diffuse) and collected all clinic and epidemiological characteristics.

We then assessed correlations with either NRL and PLR and skin core, ulcers, pitting scars, gastro-intestinal events, fibrosis on HTCR, respiratory parameters (FVC, DLCO), PAPs, diastolic abnormalities, capillaroscopy alterations and activity index (2). Clinical and sociodemographic characteristics of the study population were expressed as number and percentage (for categorical variables) and either mean ± SD or median and interquartile range (IQR) for continuous variables. Means were compared through the two-tailed Student’s t-test, whilst bivariate correlations among variables were assessed by the Spearman correlation coefficient.

Sociodemographic and clinical characteristic were reported in Table 1.

**Results:** Correlations emerged statistically significant for NLR and PLR are summarized in Tables 2 and 3 respectively: NLR vs skin score (R:0.28), DLCO (R:-0.30), PAPs (R:0.29), and activity index (R:0.28); PLR vs ulcers (R:0.31), fibrosis on HTRC (R:0.33). The correlation analysis between NLR and presence of ulcers and pitting scars was considered not statistically significant. There were no correlations between other parameters and NLR. The correlation analysis between PLR and diastolic abnormalities evaluated by echocardiography B-Mode is considered not statistically significant (p=0.06). There was no correlation between other parameters and PLR.

NLR values negatively correlated with DLCO value (R: -0.30) and positively with PAPs (R:0.29), skin score and activity index NLR level may serve as inflammatory marker in patients with SSc . Whilst PLR values correlated with presence of ulcers and fibrosis on HRCT.

**Conclusions:** In conclusion, our findings support the potential use of NLR and PLR as inflammatory markers in activity and/or severity in patients with SSc.

1.Absenger G. et al. Br J Cancer 2013; 109:395

2. Valentini G. et al. Ann Rheum Dids 2001; 60:592

**Figure:**
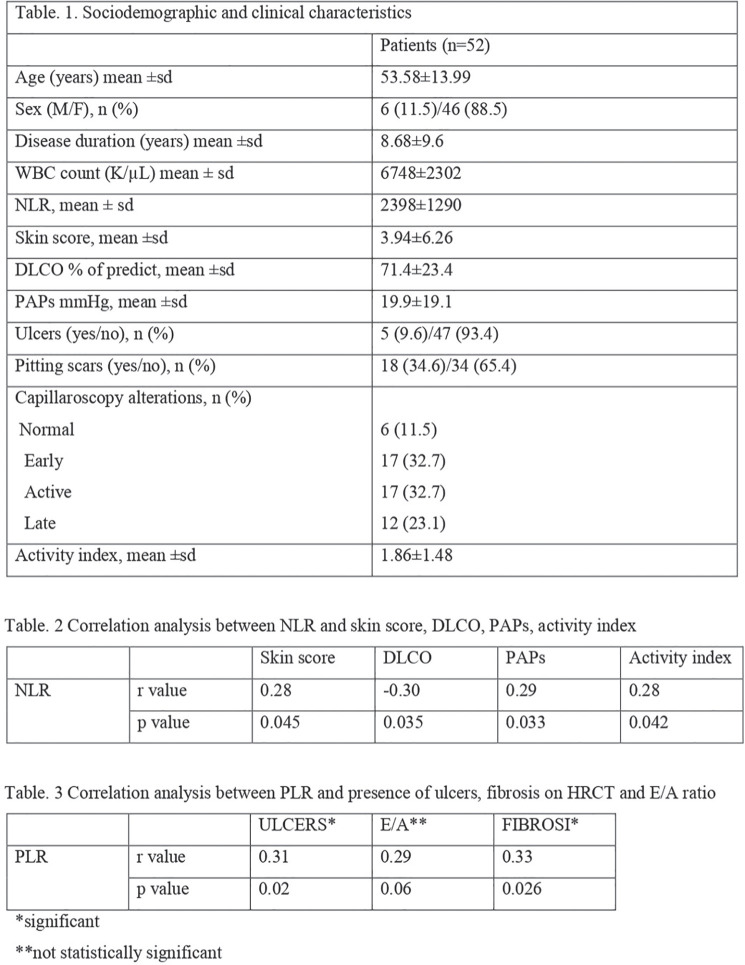


## P.026

## THE INTERCONNECTIONS BETWEEN ACTIVITIES OF THE OXIDATIVE-RELATED ENZYMES AND DISEASE ACTIVITY IN SYSTEMIC SCLEROSIS

E. Mozgovaya^1^, S. Bedina^1^, A. Trofimenko^1,2^, I. Zborovskaya^1,2^, M. Mamus^1^, E. Tikhomirova^1^, S. Spitsina^1,2^

^1^*Research Institute of Clinical and Experimental Rheumatology named after A.B. Zborovsky - Clinical biochemistry lab, Volgograd, Russia*, ^2^*Volgograd State Medical University - Hospital therapy department, Volgograd, Russia*

**Introduction:** Systemic sclerosis (SSc) is a chronic autoimmune disorder that is intimately associated with vascular damage and therefore with chronic perfusion/reperfusion and oxidative organ injury. Mesenchymal cell activation in SSc is now also considered to be mediated primarily through oxidative burst. Regulation of oxidative stress by specific enzymes including several purine metabolism enzymes is likely to play an important role in SSc progression. The objective is to characterize interrelationships among circulating xanthine oxidase (XO), xanthine dehydrogenase (XDH), superoxide dismutase (SOD) activities and SSc activity.

**Material and Methods:** 51 patients with verified SSc and 30 healthy controls were enrolled in the study. The diagnosis was verified using ACR/EULAR 2013 criteria. We assessed SSc activity in compliance with the original activity scale that is commonly used in Russia [Guseva, 1993] and by the 2001 European Scleroderma Study Group Activity Index. XO (EC 1.17.3.2), XDH (EC 1.17.1.4) and SOD (EC 1.15.1.1) plasma activities were measured by means of the spectrophotometric techniques [Dubinina, 1983; Karpova, 2006]. Results are expressed as mean±SD. The Mann-Whitney U test and Spearman’s correlation coefficient were used for statistical analysis.

**Results:** Mean age of patients was 42.8±1.3 years, mean SSc duration was 7.9±0.7 years. Mean enzymatic activities in normal controls were 3.43±0.56 nmol/ml×min (for XO), 5.19±0.71 nmol/ml×min (for XDH), and 5.40±1.03 U (for SOD). The respective enzymatic activities in SSc group were 3.91±0.62 nmol/ml×min, 7.10±0.71 nmol/ml×min, and 7.10±2.19 U. All these mean activities were significantly higher in SSc patients comparing to healthy individuals (p<0.001). XO and XDH activities positively correlated with SSc activity (r=0.499, p<0.001; r=0.741, p<0.001, respectively). The opposite but weaker trend was observed for SOD activity and SSc disease activity (r= -0.190, p=0.188).

**Conclusions:** A close relationship between prooxidant/antioxidant enzymes and some of the key SSc pathogenetic mechanisms, especially vascular disease and fibroblast activation, is widely accepted. Overall increase of oxidative stress in patients with higher disease activity, as well as depletion of antioxidant capacity can be also linked with disturbance of purine metabolism through XO and XDH modulation. Pathogenetic influence of this imbalance can also be mediated through initial phase of neutrophil extracellular traps (NETs) formation, an eventual source of nucleoprotein containing autoepitopes.

## P.027

## THE ROLES OF PLATELET-DERIVED GROWTH FACTOR RECEPTOR (PDGFR) INHIBITOR IN SKIN FIBROBLASTS AND IN MICE MODEL OF SYSTEMIC SCLEROSIS

K. Makino^1^, M. Trojanowska^2^, H. Ihn^1^

^1^*Department of Dermatology and Plastic Surgery, Kumamoto University, Kumamoto, Japan*, ^2^*Arthritis Center, Boston University School of Medicine, Boston, USA*

**Introduction:** Systemic sclerosis (SSc) is an acquired autoimmune disorder that typically results in fibrosis of the skin and internal organs. Activated fibroblasts are the key effector cells in SSc responsible for the production of collagen and the development of fibrosis. In this study, we examined the role of crenolanib, an inhibitor of PDGFR signaling, in cultured skin fibroblasts and evaluated its antifibrotic effect in the angiotensin II (Ang II)-induced mice skin fibrosis.

**Material and Methods:** Healthy control (HC) and SSc dermal fibroblasts were cultured in the presence of crenolanib, TGF-β, PDGF ligands or CCN2. Cell proliferation was measured using the Incucyte® system. Skin biopsy samples collected from 15 healthy controls and 33 dcSSc patients were included in the microarray analysis. Ang II was administered by subcutaneous osmotic pumps in mice.

**Results:** Crenolanib effectively inhibited proliferation of SSc and HC fibroblasts, and attenuated basal and TGF-β-induced expression of CCN2 and periostin. In contrast to HC fibroblasts, SSc fibroblasts proliferated in response to PDGFAA, while a combination of PDGFAA and CCN2 was required to produce a similar response in HC fibroblasts. PDGFRα mRNA correlated with CCN2 and other fibrotic markers in the skin of SSc. In mice challenged with Ang II, PDGFRα-positive cells were increased in the skin. These PDGFRα-positive cells co-localized with PDGFRβ, procollagen and periostin. Treatment with crenolanib by daily intraperitoneal injections attenuated the skin fibrosis in the Ang II model.

**Conclusions:** Our data suggest that inhibition of PDGF signaling presents an attractive therapeutic approach in SSc.

## P.028

## CORRELATION OF ANTINUCLEAR AUTOANTIBODIES, SOLUBLE TGF-<BETA> ISOFORMS, AND TGFB1 +869T>C AND +915G>C POLYMORPHISMS IN PATIENTS OF SOUTHERN MEXICO WITH SYSTEMIC SCLEROSIS

J.A. Lomeli Nieto^1^, J.F. Muñoz Valle^1^, C.J. Baños Hernández^2^, J.E. Navarro Zarza^3^, S. García Arellano^1^, B.U. Anaya Macias^1^, C.E. Fuentes Baez^1^, I. Parra Rojas^2^, J. Hernández Bello^1^

^1^*Instituto de Investigación en Ciencias Biomédicas, Guadalajara, Jalisco, Mexico*, ^2^*Facultad de Ciencias Químico-Biológicas, Chilpancingo, Guerrero, Mexico*, ^3^*Departamento de Reumatología, Hospital General de Chilpancingo, Chilpancingo, Guerrero, Mexico*

**Introduction:** Systemic Sclerosis (SSc) is an autoimmune disease characterized by chronic inflammation and fibrotic processes in the skin and internal organs. These two processes are associated with an increased level of transforming growth factorβ (TGF-β).

**Material and Methods:** 56 patients with SSc and 120 unrelated control subjects from Southern Mexico were included. The demographic and clinical data such as signs and symptoms, autoantibodies and treatment were collected from clinical history. The genotypes of the polymorphisms +869T>C and +915G>C of the TGFB1 gene were identified by the polymerase chain reaction-restriction fragment length polymorphism (PCR-RFLP) method. The levels of soluble isoforms of TGF-β (sTGF-β) were quantified by immunoassays with magnetic microspheres (MAGPIX).

**Results:** The laboratory tests of the clinical evaluation showed that antinuclear antibodies (ANAs) were present in 75% of the patients, in which the anticentromere antibodies (ACAs) were the most prevalent with 26%. The concentration of the three serum TGF-β isoforms was higher in control subjects than in patients with SSc (p < 0.0001). The analysis between each isoform and clinical data showed a positive correlation between TGF-β1, TGF-β2 and TGF-β3 isoforms with ACAs levels (r2 = 0.344, p = 0.014), (r2 = 0.291, p = 0.040), and (r2 = 0.344, p = 0.014) respectively. The CC genotype of the +869T>C variant was associated with SSc risk through a dominant inheritance model (p = 0.040, OR = 2.82, CI = 1.021-7.793) being the C allele, the risk allele for the disease susceptibility. On the other hand, the GC genotype of the +915G>C variant was associated through a dominant model with risk to SSc (p = 0.006, OR = 11.67, CI = 1.289-96.754) being the C allele, the risk allele for disease susceptibility.

**Conclusions:** There is a high prevalence of ACAs in SSc patients and this autoantibody have a positive correlation with the three TGF-β isoforms, which are present in low concentrations in SSc compared to control subjects. Besides, the C alleles of the +869T>C and +915G>C variants are associated with SSc susceptibility in the studied population.

## P.029

## THE LABORATORY STRATEGY FOR ANTIBODIES SCREENING AND SPECIFIC ANTIGEN DETECTION AS DIAGNOSTIC MARKER OF PULMONARY FIBROSIS IN SYSTEMIC SCLEROSIS: PRELIMINARY STUDY

V. Logito^1^, W. Agnestia Maranna Saragih^2^, A. Tjandrawati^1^, S. Dewi^3^

^1^*Department of Clinical Pathology, Faculty of Medicine, Padjadjaran University, Bandung, Indonesia*, ^2^*Department of Internal Medicine, Faculty of Medicine, Padjadjaran University, Bandung, Indonesia*, ^3^*Immunology Study Center, Padjadjaran University, Bandung, Indonesia*, ^4^*Bandung, Indonesia*

**Introduction:** Introduction: Systemic sclerosis (SSc) is a rare connective tissue disease with heterogenic cause. Interstitial pulmonary disease (ILD) is a common manifestation in organ involvement of SSc and leading cause of death especially pulmonary fibrosis. Pulmonary fibrosis is a serious manifestation of systemic sclerosis and lead as the cause of death in SSc. Specific antibodies and antigen detection plays important rules as diagnostic marker related to pulmonary fibrosis. The aim of this study was to evaluate two laboratory diagnostic strategies for pulmonary fibrosis detection in SSc.

**Material and Methods:** Material and Methods: This retrospective study was conducted in Rheumatology Outpatient Clinic Dr. Hasan Sadikin General Hospital Bandung, from 1 January 2018 until 30 August 2019. A total sample of 40 SSc patient were evaluated for pulmonary fibrosis, according to standard diagnostic using HRCT (High Resolution CT Scan). We defined two laboratory diagnostic strategy; the first strategy was used established algorithm from American College of Rheumatology / European League against Rheumatism (ACR/EULAR), which consist of antinuclear antibodies screening (ANA IIF, Euroimmun - Germany) and followed with specific antigen detection (Anti-topoisomerase I, Anti Th/To, Euroline, Euroimmun - Germany). The second strategy, we defined the vice versa strategy from the ACR/EULAR, of which started with specific antigen detection and followed by antibodies screening approach. Collected data were tabulated and presented as descriptive summaries (number and percentage).

**Results:** Results: The total of 40 patient consist of 34 patient with SSc and 6 patient with Mixed Connective Tissue Disease. Among of this study population, 19 subject was defined as pulmonary fibrosis (47.5%), with mean age of 42 years old and gender was dominated by female (94,73%). Using the first strategy approach, antibodies screening of homogenous nucleolar pattern was dominantly found (n=10, 52,6%) and combination with ATA antigen was achieved for 7 subject (70%). Meanwhile, using second strategy approach, ATA antigen was achieved among 8 subject (42,1%), but the positivity of homogenous nucleolar pattern was achieved as high as 37,5% (n=3). Other combination between antibodies screening and antigen detection using first strategy showed more subject being included as pulmonary fibrosis, than the second strategy. We found that first laboratory strategy was more likely to be used as pulmonary fibrosis diagnostic approach.

**Conclusions:** Established SSc algorithm from the ACR/EULAR is a useful diagnostic modalities for pulmonary fibrosis detection in SSc population.

## P.030

## CRYSTALLINE SILICA IMPAIRS EFFEROCYTOSIS ABILITIES OF HUMAN AND MOUSE MACROPHAGES

A. Lescoat^1^, A. Ballerie^1^, M. Lelong^2^, Y. Augagneur^2^, C. Morzadec^2^, S. Jouneau^3^, P. Jego^1^, O. Fardel^4^, L. Vernhet^5^, V. Lecureur^5^

^1^*CHU Rennes, Internal medicine and clinical immunology and IRSET UMR 1085, Rennes, France*, ^2^*IRSET UMR 1085, Rennes, France*, ^3^*CHU Rennes, Pneumology and respiratory disorders and IRSET UMR 1085, Rennes, France*, ^4^*CHU-Rennes, Biology and IRSET UMR 1085, Rennes, France*, ^5^*Rennes University and IRSET UMR 1085, Rennes, France*

**Introduction:** Inhalation of crystalline silica (SiO2) is considered as the main environmental risk factor of systemic sclerosis (SSc). The pathogenic links between silica inhalation and the onset of fibrotic autoimmune disorders such as SSc are still to be determined. A defect of apoptotic cell clearance (i.e. efferocytosis), is reported in macrophages from patients with SSc. However, the precise mechanisms associating SiO2 exposure and efferocytosis impairment remain to be determined in SSc. In the present study, we first aim to determine whether SiO2 might alter efferocytosis capacities of human and mouse macrophages. We secondly explore possible mechanisms that could explain an impaired efferocytosis, with a specific focus on macrophage polarization and on the RhoA/ROCK pathway, a key regulator of cell adhesion, cytoskeleton remodelling and phagocytosis, that has been recently advanced as a promising therapeutic target in SSc.

**Material and Methods:** Human monocyte-derived macrophages (MDM) and C57BL/6J mice exposed to SiO2 and to CFSE-positive apoptotic Jurkat cells were analyzed by flow cytometry to determine their efferocytosis index (EI). The effects of ROCK inhibitors on EI of SiO2-exposed MDM and MDM from SSc patients were evaluated in vitro.

**Results:** SiO2 significantly decreased EI of human MDM in vitro and mouse alveolar macrophages in vivo. In human MDM, this SiO2-associated impairment of efferocytosis required the expression of the membrane receptor SR-B1 and was associated with a decreased expression of M2 polarization markers (CD206, CD204, CD163). F-actin staining, RhoA activation and impairment of efferocytosis, all induced by SiO2, were reversed by the ROCK inhibitor Y27632. Moreover, the EI of MDM from SSc patients was similar to the EI of SiO2-exposed MDM and Y27632 significantly increased SSc MDM efferocytosis capacities, suggesting a likewise activation of the RhoA/ROCK pathway in SSc MDM.

**Conclusions:** Exposure to SiO2 impaired efferocytosis in mouse alveolar macrophages and human MDM. This altered efferocytosis in human MDM is associated with RhoA/ROCK activation both in SiO2 exposed MDM and in MDM from SSc patients. Interestingly, significant associations between ROCK1/2 and RhoA gene polymorphisms and SSc have been reported, which also strengthens the possible role of this pathway in this systemic autoimmune disorder. New Rho inhibitors have been recently designed for the treatment of SSc, with promising results on dermal fibrosis in bleomycin SSc mouse model. Beyond fibroblasts, identifying other biological targets of Rho inhibitors may be a new key step for scleroderma research and our results suggest that macrophages could constitute such relevant targets.

## P.031

## AN ITALIAN MULTICENTER STUDY ON THE ASSOCIATION OF ANTI-RNA POLYMERASE III ANTIBODY WITH BREAST CANCER AND SILICONE BREAST IMPLANTS RUPTURE IN PATIENTS WITH SYSTEMIC SCLEROSIS

M.G. Lazzaroni^1^, C. Caimmi^2^, E. Bertoldo^2^, C. Campochiaro^3^, G. De Luca^3^, F. Franceschini^1^, A. Tincani^1^, P. Airo’^1^

^1^*University and Spedali Civili of Brescia, Brescia, Italy*, ^2^*Azienda Ospedaliera Universitaria Integrata, Verona, Italy*, ^3^*IRCCS San Raffaele Hospital, Milano, Italy*

**Introduction:** Several epidemiological studies have investigated the link between silicone breast implants (SBI) and Systemic Sclerosis (SSc). Although discordant data were reported, a recent analysis of SBI followed by United States Food and Drug Administration post approval studies, including nearly 100,000 individuals, described an association of SBI with a higher rate of SSc (Standardized incidence ratio 7.00), compared with normative data (1). The analysis of clinical associations in patients with SSc is complicated by the heterogeneity of the disease, both on immunological and clinical terms. Interestingly, a specific association of anti-RNA polymerase III antibody (anti-RNAP3) and SBI in Japanese patients with SSc was described in a single-center cohort (2). It should be noted that an association of anti-RNAP3 with breast cancer, particularly when synchronous with SSc onset, was also demonstrated (3,4).

The objective of the study was to evaluate the association of SBI with SSc in Italian patients classified according to their SSc-related autoantibodies.

**Material and Methods:** 742 consecutive women with SSc classified according the 2013 criteria of SSc, that were evaluated for the presence of SSc-specific autoantibodies (anti-Topoisomerase-I (anti-Topo-I), anticentromere (ACA), and anti-RNAP3) in 3 Italian University centres, were included. For each patient, history of breast cancer and SBI were recorded. SBI rupture was confirmed by CT or MRI scan, that were performed when clinically indicated.

**Results:** Comparing anti-RNAP3+ vs anti-RNAP3- patients, a non-significantly higher frequency of breast cancer (10.5% vs 4.4%; p=0.09) was noticed. We collected 12/742 patients with an SBI; in 11/12 patients, SSc onset occurred after SBI implantation, while 1 patient that was diagnosed with SSc many years before SBI implantation was excluded from further analysis. Comparing anti-RNAP3+ vs anti-RNAP3- patients, a significant higher frequency of SBI (10.5% vs 1.0%; p=0.002) and SBI rupture (7.8% vs 0.3%; p=0.001) was noticed. Considering only the frequency of SBI rupture in the absence of a history of breast cancer, the comparison between anti-RNAP3+ vs anti-RNAP3- was significant (5.9% vs 0.1%; p=0.007).

**Conclusions:** In this large Italian cohort, a higher prevalence of SBI and SBI rupture in anti-RNAP3+ compared to anti-RNAP3- SSc patients was confirmed, regardless of the history of breast cancer. This preliminary observation should be confirmed in multicentre cohorts, particularly regarding the connection between SSc and SBI rupture.

**Figure:**
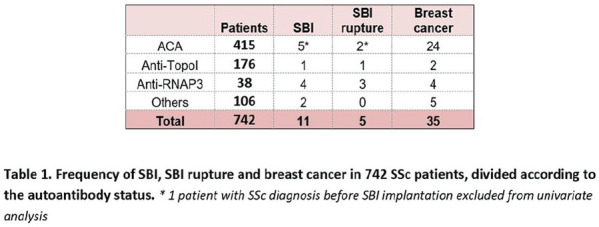


**References**

1. Coroneos CJ, et al. Ann Surg 2019;269:30-6.

2. Saigusa R, et al. J Dermatol 2016; 43:808-10.

3. Moinzadeh P, et al. Arthritis Res Ther. 2014;16(1):R53.

4. Lazzaroni MG, et al. J Rheumatol. 2017;44(5):639-47.

## P.032

## ANTI-PM/SCL ANTIBODY IN PATIENTS WITH SYSTEMIC SCLEROSIS. ANALYSIS OF THE MULTICENTER EUSTAR COHORT, WITH A CASE-CONTROL STUDY ON ASSOCIATION WITH MALIGNANCIES

M.G. Lazzaroni^1^, E. Marasco^2^, C. Campochiaro^3^, J. Devries-Bouwstra^4^, E. Hachulla^5^, E. Zanatta^6^, S. Barsotti^7^, G. Abignano^8^, M.-E. Truchetet^9^, G. De Luca^3^, E. De Langhe^10^, R. Hesselstrand^11^, F. Ingegnoli^12^, E. Bertoldo^13^, C. Caimmi^13^, V. Smith^14^, S. Bellando-Randone^15^, H. Poormoghim^16^, E. Colombo^17^, A. Ceribelli^18^, A. Furloni^1^, S. Zingarelli^1^, F. Franceschini^1^, F. Del Galdo^8^, C.P. Denton^19^, L. Cavagna^2^, Y. Allanore^20^, P. Airo’^1^

^1^*ASST Spedali Civili of Brescia, University of Brescia, Brescia, Italy*, ^2^*Hospital IRCCS Policlinico S. Matteo Foundation of Pavia, University of Pavia, Pavia, Italy*, ^3^*San Raffaele Scientific Institute, UniRAR, Milano, Italy*, ^4^*Leiden University Medical Centre (LUMC), Leiden, The Netherlands*, ^5^*University Lille Nord-de-France, Lille, France*, ^6^*University of Padova, Padova, Italy*, ^7^*University of Pisa, Pisa, Italy*, ^8^*University of Leeds, Leeds, United Kingdom*, ^9^*University Hospital of Bordeaux, Bordeaux, France*, ^10^*University Hospitals Leuven, Leuven, Belgium*, ^11^*Lund University, Lund, Sweden*, ^12^*ASST Pini-CTO, Università degli Studi di Milano, Milano, Italy*, ^13^*Azienda Ospedaliero Universitaria Integrata, Verona, Italy*, ^14^*Ghent University Hospital, Ghent University, Ghent, Belgium*, ^15^*University of Florence, Firenze, Italy*, ^16^*Firoozgar Hospital, Teheran, Iran*, ^17^*University of Sassari, Sassari, Italy*, ^18^*Humanitas Clinical and Research Center-Irccs, Milano, Italy*, ^19^*Royal Free Hospital and University College London Medical School, London, United Kingdom*, ^20^*University Paris Descartes and Cochin Hospital, Paris, France*

**Introduction:** The main clinical associations of anti-PM/Scl in Systemic Sclerosis (SSc) include calcinosis, joint and muscle involvement, interstitial lung disease (ILD) and possibly scleroderma renal crisis. A possible association of anti-PM/Scl with cancer was reported in single-centre SSc series, but has never been analysed in large multicentre studies.

The aim of the present study was to evaluate clinical associations of anti-PM/Scl in patients with Systemic Sclerosis (SSc) in the European scleroderma trials and research (EUSTAR) registry, with specific focus on scleroderma renal crisis and malignancies.

**Material and Methods:** 1) Analysis of the EUSTAR database: 7,353 SSc patients with information available on their anti-PM/Scl status were included; 2) Case/control study: additional retrospective data, including malignancy history, were queried to 21 participating EUSTAR centres; 165 anti-PM/Scl+ SSc cases were compared with 257 local anti-PM/Scl- SSc controls, matched for sex, cutaneous subset, disease duration, and age at SSc onset.

**Results:** 1) In the EUSTAR registry, renal crisis was more frequent among anti-PM/Scl+ than anti-PM/Scl-SSc patients, although not statistically significant (5.6% vs. 3.1%; p=0.14). In multivariable analysis, adjusted for age at disease onset, sex, and disease duration, in which anti-PM/Scl were associated with joint and muscle involvement, lung fibrosis, and intestinal symptoms. Moreover, anti-Pm/Scl+ patients with muscle involvement had significantly more frequent cardiac involvement (systolic and diastolic left ventricular dysfunction; conduction blocks), lung involvement at HRCT, intestinal symptoms, joint contractures and tendon friction rubs, as compared to those without muscle involvement.

2) In the case/control study, some of the clinical associations of anti-PM/Scl were confirmed (Table 1). No significant association with malignancies was found (OR 1.61 [0.84-3.09]; p=0.17); malignancies concomitant to SSc onset were rare both among anti-PM/Scl+ SSc patients and controls, without differences in the two groups (p=0.72 considering an interval of ±24 months). Patients with synchronous malignancies were older at SSc onset than other patients (p:0.003), irrespective of the anti-PM/Scl status.

**Conclusions:** In the largest series of anti-PM/Scl + SSc patients so far evaluated, well-known clinical associations were confirmed. Anti-Pm/Scl patients with muscle involvement should be considered at higher risk of a more severe disease phenotype, with cardiac and lung involvement. In the case/control study, a significant association of anti-PM/Scl with malignancies in SSc patients was not observed, but older age at SSc onset was observed in patients with a synchronous malignancy, irrespective of autoantibody status.

**Figure:**
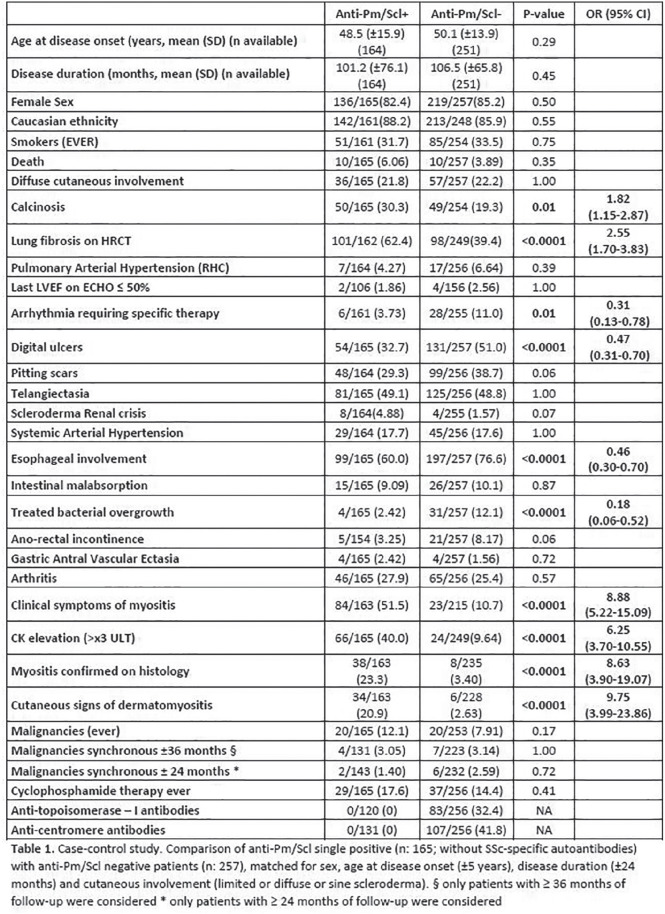


## P.033

## DISCOVERY OF T CELL EPITOPES FROM THE AUTOANTIGENS IMPLICATED IN SYSTEMIC SCLEROSIS: AN IN SILICO APPROACH

M. Larche, B. Duvvuri, R. Quinn, M. Larche

*McMaster University, Hamilton, Canada*

**Introduction:** Centromere and Topoisomerase proteins are the target of autoantibody responses in Systemic Sclerosis (SSc). Centromere Protein (CENP)- A and B are the major antigen targets for anti-centromere antibodies. Anti-topoisomerase antibodies (ATA) target DNA-topoisomerase I (Topo I), an enzyme present in all cells and crucial for DNA replication. A role for T cells in the generation of autoantibodies is supported by the correlation of autoantibodies with HLA-risk alleles. T cells, independently of autoantibodies, can also play a pathogenic role in autoimmunity. Thus, induction of tolerance in antigen-specific T cells may reduce levels of autoantibodies and modulate pathogenic autoreactive T cell responses. With the ultimate aim of tolerizing T cells in an antigen-specific manner, we sought to determine the specific epitope-allele combinations associated with the autoimmune response in SSc.

**Material and Methods:** Peptide sequences of human CENP-A, B and Topo I were retrieved from UniProt, a protein sequence database. Epitope prediction methods, Sturniolo and NetMHCII 2.2, were utilized to identify optimal CD4+ T cell epitopes (length 15 a.a.) based on peptide-MHC class II binding affinities. MHC class II alleles were selected based on their disease association and wide population coverage (~>95%). Predicted peptides were further filtered and redesigned based on the most commonly conserved core peptide sequences across MHC class II alleles.

**Results:** Each autoantigen had numerous strong core peptide sequences for each MHC class II allele. Multiple regions contained overlapping sequences capable of binding to different MHC alleles with different affinities. To focus, the core peptide sequences most commonly conserved across MHC alleles were explored. For Topo I, we found 4 core epitopes that were found to be conserved for >= 5 MHC alleles. For CENP-A, there were 5 core sequences that were commonly conserved (>= 10 MHC alleles). For CENP-B, we found 10 commonly conserved core peptide sequences (>= 10 MHC alleles). Altogether, 12 epitopes were selected for in-vitro testing based on strong binding affinity and maximum population coverage.

**Conclusions:** In silico T cell epitope prediction identified the MHC class II binding affinities of peptide sequences spanning the Topo I, CENP-A, and CENP-B autoantigens which may represent dominant T cell epitopes that trigger autoantibody formation in SSc. These T cell epitopes will be validated in future studies and used to develop a peptide-based therapeutic vaccine for the treatment of SSc.

## P.034

## THE ROLE OF DIFFERENT ANTINUCLEAR ANTIBODIES IN VASCULAR LESIONS IN PATIENTS WITH SYSTEMIC SCLEROSIS

N. Klyaus^1^, M. Simakova^2^, A. Maslyanskiy^1^, O. Moiseeva^2^

^1^*Almazov National Medical Research Centre - Department of Rheumatology, Saint-Petersburg, Russia*, ^2^*Almazov National Medical Research Centre - Department of Cardiology, Saint-Petersburg, Russia*

**Introduction:** Systemic sclerosis (SSc) is the disease that characterized by structural and functional changes in blood vessels. Different autoimmune reactions lead to the accumulation of collagen and the development of the obliterating vasculopathy, that leads to various clinical manifestations of the SSc. The role of various antinuclear antibodies in the pathogenesis of vasculopathy in SSc has not been enough studied.

**Material and Methods:** 143 patients (11 males, 132 females) with SSc were enrolled in the study. The presence of telangiectasias, finger tip pitting scars, digital tip ulcers, pulmonary hypertension were estimated. The naifold videocapillaroscopy (NVC) was performed, the density of the capillary loops and the pattern were assessed, a semi-quantitative evaluation of capillary density and alterations were carried out. Antinuclear antibodies nRNP/Sm, Sm, SS-A, Ro-52, SS-B, Scl-70, PM-Scl, Jo-1, CENP A, CENP B, PCNA, dsDNA, Nukleosomes, Histone, Rib. P-Prot, AMA-M2, Ku, Th/To, NOR 90, Fibrillarin, RP155, RP11 were evaluated. The presence of antinuclear antibodies no less than 2+ was interpreted as positive result.

**Results:** The median age was 53±12 years. The duration of symptoms of SSc was 10±8 years. The association between the higher percent of capillary alterations and PM-Scl-antibodies was found (p= 0,013). The association between the presence of pulmonary hypertension and CENT B-, AMA M2 and Ro-52-antibodies was established (p=0,018, p=0,002 and p=0,005, respectively). The association between the presence of digital tip ulcers and Scl-70 and RP11-antibodies was determined (p= 0,049 and p= 0,013, respectively). The association between the presence of telangiectasias and absence of Ku-antibodies was found (p= 0,006). The association between the presence of finger tip pitting scars and absence of SS-A-antibodies was determined (p= 0,018).

**Conclusions:** In our study the contribution of different autoantibodies in the pathogenesis of vascular involvement in patients with SSc is shown.

## P.035

## MALE MICROCHIMERISM IN SSC PATIENTS

J. Milas Ahic^1,2^, Z. Kardum^1^, S. Tokic^2^, V. Prus^1,2^

^1^*Department of Rheumatology, Clinical Immunology and allergology UHC Osijek, Osijek, Croatia*, ^2^*Faculty of Medicine Josip Juraj Strossmayer University of Osijek, Osijek, Croatia*

**Introduction:** Chimerism refers to the co-existence of genetically disparate populations of cells within a single host. This phenomenon is well demonstrated in several clinical settings, such as pregnancy, twinning, transplantation and blood transfusion. Given that chimeric populations typically account for less than 5% of the host cellular burden, the term microchimerism (MC) is used to describe this phenomenon. Male DNA or cells are often used to measure microchimerism in women. Male MC persisting from pregnancy has been found at greater frequencies in women with systemic sclerosis (SSc) compared with controls, suggesting a possible role in immunopathology of the disease.

**Material and Methods:** 10 healthy (mean age±SD; 56.4±2.54) and 18 SSc positive (59.4±8.44) adult women with at least one male offspring were studied for the presence of male DNA in peripheral blood mononuclear cells (PBMC). Following PBMC and DNA extraction (mean±SD; 18,04±4,56 ng/ul), selected subjects were probed for the expression of sex-determining region Y (SRY) gene using Quantifiler TM Y Human Male DNA Quantification kit and Quant Studio 5 real-time PCR system. In addition, AmpFlSTR Yfiler PCR Amplification Kit TM kit and ProFlex PCR instrument were selected for multiplex amplification of 16 Y STR loci including DYS456, DYS389 I, DYS390, DYS389 II, DYS458, DYS19, DYS385 a/b, DYS393, DYS391, DYS439, DYS635, DYS392, Y GATA H4, DYS437, DYS438 and DYS448. Allelic variants were identified by electrophoresis on an ABI 310. Genetic Analyser and GeneMapper ID v3.2.1 was used for raw data analysis.

**Results:** We found no evidence of Y DNA in PBMCs of healthy or SSc positive female examinees, within the tested concentration range (50-0.02 ng/ul). Positive results of both SRY and Y STR analysis, were expectedly however, obtained in two male SSc subjects used as positive controls.

**Conclusions:** In a small number of tested patients no evidence of male MC in PBMC of SSc patients or healthy examinees was found, which adds evidence that male MC hypothesis in the development of SSc is not supported. Other possible reasons for negative results could be that the tested concentration for Y DNA in PBMC was too small, but also, that the affected tissue (such as skin) in SSc patient would be more appropriate for testing then PBMC.

## P.036

## EPIDEMIOLOGY AND TREATMENT OF PERIPHERAL NEUROPATHY IN SYSTEMIC SCLEROSIS

B. Almehmadi^1-4^, F. To^1-3 ,5^, K. Devakandan^1,2^, M. Anderson^6^, S. Johnson^1,2,3^

^1^*Toronto Scleroderma Program, Toronto, Canada*, ^2^*Mount Sinai Hospital, Toronto Western Hospital, Department of Medicine, Division of Rheumatology, Toronto, Canada*, ^3^*University of Toronto, Toronto, Canada*, ^4^*College of Medicine, Majmaah University, Al Majma’ah, Saudi Arabia*, ^5^*University of British Columbia, Vancouver, Canada*, ^6^*University Health Network Library Services, Toronto General Hospital, Toronto, Canada*

**Introduction:** Peripheral neuropathy epidemiology and treatment in systemic sclerosis (SSc) is poorly understood. The objectives of this study were to evaluate the incidence, prevalence, risk factors, and treatments of peripheral neuropathy in SSc.

**Material and Methods:** A systematic review of the literature reporting peripheral neuropathy in SSc was performed. Evidence evaluating incidence, prevalence, risk factors, and treatments were synthesized.

**Results:** 111 studies reported 1788 subjects with at least one type of peripheral neuropathy. The mean age was 47.3 years. The mean time between onset of SSc and detection of peripheral neuropathy was 3.2 years. The mean incidence of peripheral neuropathy was 21.2% (range 4%-60%) and the mean prevalence 21.7% (range 4%-40.7%). Risk factors and neuropathic patterns are presented in Tables 1 and 2. There were 70 subjects with successful treatments (n=44 restoring sensation, n=26 restoring motor function). Treatments included decompression surgery, prednisone, cyclophosphamide, carbamazepine, transcutaneous electrical nerve stimulation, tricyclic antidepressants and IVIG. All studies were case reports and observational cohorts. There were no randomized controlled trials (RCT).

**Conclusions:** Peripheral neuropathy is not uncommon in SSc, with a variety of etiologies and presentations. Observational data suggests that compression neuropathies can be successfully treated with decompression surgery. The observational data supporting immunosuppressive and anticonvulsants to treat peripheral neuropathy in SSc is limited and conflicting. This data provides the signal of effect to justify RCT to evaluate the efficacy of these interventions.

## P.037

## AUTOANTIBODIES TARGETING CXCR3, CXCR4 AND AT1R AFFECT THE EXPRESSION OF THEIR COGNATE RECEPTORS IN THE APOE KO MOUSE MODEL

L. Johanson^1^, L. Brachaczek^1^, Z. Aherrahrou^2^, G. Marschner^1^, S. Pitann^1^, A. Kerstein-Stähle^1^, A. Müller^1^, H. Heidecke^3^, C. Herden^4^, G. Riemekasten^1^

^1^*Department of Rheumatology and Clinical Immunology, University of Lübeck, Lübeck, Germany*, ^2^*Institute for Cardiogenetics, University of Lübeck, Lübeck, Germany*, ^3^*CellTrend GmbH, Luckenwalde, Germany*, ^4^*Institute of Veterinary Pathology, Justus-Liebig-University, Gießen, Germany*

**Introduction:** It has been shown that autoantibodies recognising different G protein-coupled receptors (GPCR) are found in healthy donors and rheumatic diseases such as systemic sclerosis (SSc), forming specific regulatory networks. Our previous findings indicate that the chemokine receptors CXCR3 and CXCR4 as well as the Angiotensin II receptor type 1 (AT1R) along with their corresponding autoantibodies might be linked to vascular and pulmonary pathology in SSc. Herein, we investigated effects of autoantibodies recognising tree GPCR in a murine model of atherosclerosis with regard to SSc-related phenotypes.

**Material and Methods:** Two immunoglobulin G preparations containing autoantibodies directed against CXCR3, CXCR4 or AT1R from patients with SSc (SSc-IgG) and healthy donors (HD-IgG) were passively transferred into a murine model of atherosclerosis (C57BL/6, ApoE-/-, high fat diet). The SSc-IgG contained low amounts of anti-CXCR3 and anti-CXCR4 antibodies (SSc-IgG) and the HD-IgG contained high amounts of anti-CXCR3 and anti-CXCR4 antibodies. The controls consisted of mice fed with normal diet but without treatment (no/no) and mice fed with high fat diet and treated with sodium chloride (NaCl). Following sacrifice, the impact on gene and protein expression was examined using qPCR, flow cytometry of splenocytes and immunohistochemistry of lung tissue. ELISA of plasma samples was performed to determine autoantibody concentrations. As functional readout of atherosclerosis, the size of atherosclerotic plaques within the aorta was measured using red oil staining.

**Results:** Passive transfer of human IgG derived from healthy donors (HD-IgG) into ApoE KO mice induced elevated numbers of splenic CXCR3+ T-helper cells, CXCR3+ cytotoxic T cells and CXCR3+ B cells compared to the control group (no/no, p<0.05). Similarly, numbers of splenic CXCR4+ T-helper cells and CXCR4+ monocytes were increased in the HD-IgG group compared to the control group (no/no, p<0.05). In addition, passive transfer of SSc-IgG into ApoE KO mice induced a decreased CXCR3 mRNA expression in the lung compared to HD-IgG (p<0.05). Further, passive transfer of both, SSc-IgG and HD-IgG, into ApoE KO mice induced increased titers of murine autoantibodies (anti-CXCR3, anti-CXCR4, anti-AT1R IgG) in peripheral blood and an increased size of atherosclerotic plaques. Interestingly, in the NaCl-treated control group and in the HD-IgG-treated group high amounts of murine anti-CXCR4 autoantibodies were associated with a decreased size of atherosclerotic plaques in the aorta (rs=-0.8571 or -0.8929, respectively, p<0.05).

**Conclusions:** We assume that anti-CXCR3 and anti-CXCR4 antibodies modulate the expression of their cognate GPCR in the ApoE KO mouse model. This may also be of relevance in autoimmune diseases, such as SSc.

## P.038

## A PILOT STUDY ASSESSING THE EFFECT OF AIR POLLUTION ON EXTRACELLULAR VESICLES IN SYSTEMIC SCLEROSIS

F. Ingegnoli^1^, T. Schioppo^1^, M. Hoxha^2^, S. Iodice^2^, L. Pergoli^2^, A. Murgo^3^, L. Ferrari^2^, V. Bollati^2^, R. Caporali^1^

^1^*Università degli Studi di Milano, Dept of Clinical Sciences and Community Health, Div. Clinical Rheumatology, ASST Pini, Milano, Italy*, ^2^*EPIGET Lab, Università degli Studi di Milano, Department of Clinical Sciences and Community Health, Milano, Italy*, ^3^*Division of Clinical Rheumatology, ASST Pini, Milano, Italy*

**Introduction:** The identification of specific diagnostic and prognostic biomarkers remains an unmet need in Systemic Sclerosis (SSc). Over the past few years, it has been suggested that extracellular vescicles (EVs) and environmental toxicants, such as particulate matter (PM), may have an important role in the pathogenesis of autoimmune diseases. At present, no data are available on the impact of PM exposure on EVs from patients with SSc. Our aim was to evaluate the effects of PM with aerodynamic diameter less than 10 µm (PM10) and 2.5 µm (PM2.5) on EVs in SSc and osteoarthritis (OA).

**Material and Methods:** Plasma EVs were analyzed by Nanosight and flow cytometry after labeling with the following markers: CD14 (monocyte), CD61 (platelet), CD25 (T-reg), human endogenous retrovirus w (HERV-w) and human leukocyte antigen G (HLA-G). Demographic and clinical data were collected for each patient. Plasma EV concentrations were measured in SSc and OA patients and were analyzed by generalized linear regression models. Daily PM concentrations, estimated by the Regional Environmental Protection Agency at municipality resolution, were used to assign short-term exposure (mean of the 7 days preceding the evaluation) to each study subject.

**Results:** 12 consecutive limited cutaneous SSc (11 female, median age 66.8 yrs, median disease duration 12.3 yrs, median mRSS 3.5) and 12 patients with OA (8 female, median age 67.1 yrs, median disease duration 9.3 yrs) were enrolled. In the table below, EV count and MV subtypes are reported with respect to PM2.5 and PM10 exposure both in SSc and OA patients. The increase of PM2.5 led to a decrease of HERV-w+ microvesicles (MV) in both SSc (β =-0.10; p=0.01) and OA (β =-0.09; p=0.01) and of HLA-G+ (β =-0.11; p<0. 01) only in SSc. Similar results were observed analyzing PM10 exposure. Analysis of EV concentration according to their dimensions showed a negative association in the size range of exosomes (63-92nm) in SSc compared to OA (p<0. 05) and in the range of MV (230-510 nm). A positive association between HLA-G+ with ESR (β =0.34; p<0.01).

**Conclusions:** In our study, limited cutaneous SSc showed different EV concentrations from controls: SSc tends to have less exosomes and more MV than OA. Moreover, environmental stimuli are confirmed to be able to influence HERV-w+ MV release both in SSc and controls. Finally, in SSc patients PM exposure could significantly alter the release of HLA-G+ MV that has been correlated to the process of self-tolerance maintenance.

**Figure:**
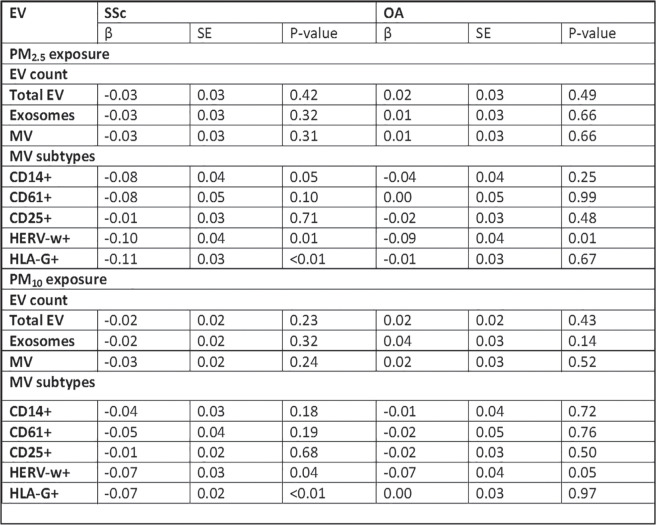


## P.039

## SYSTEMIC SCLEROSIS WITHOUT ANTINUCLEAR ANTIBODIES: A MULTI-CENTER STUDY OF EUSTAR COHORT IN CHINA

M. Hui^1^, J. Zhou^1^, X. Duan^2^, L. Zhang^3^, M. Li^1^, Q. Wang^1^, J. Zhao^1^, Y. Hou^1^, D. Xu^1^, X. Zeng^1^

^1^*Peking Union Medical College Hospital, Beijing, China*, ^2^*The Second Affiliated Hospital of Nanchang University, Nanchang, China*, ^3^*ShanXi Bethune Hospital, ShanXi Academy of Medical Sciences, Taiyuan, China*

**Introduction:** The presence of circulating antinuclear antibodies (ANAs) is a hallmark of immune dysregulation in patients with systemic sclerosis (SSc). A variety of ANAs, including anti-centromere, anti-topoisomerase I, and anti-RNA polymerase III antibodies, are associated with unique sets of disease manifestations and widely used in routine clinical practice for diagnosis, clinical subgrouping, and prediction of future organ involvements and prognosis in SSc patients. This study aimed to investigate the clinical features of SSc patients with negative ANAs in a European League Against Rheumatism Scleroderma Trials and Research Group (EUSTAR) and Chinese Rheumatism Data Center (CRDC) multi-center cohort in China.

**Material and Methods:** Patients were prospectively recruited between April 2008 and June 2019 based on the EUSTAR database and CRDC from 154 clinical centers nationwide, all of whom fulfilled the 2013 ACR/EULAR classification criteria for systemic sclerosis. Antinuclear antibody was intensively collected. Demographic, clinical, and laboratory data were compared between ANA-positive SSc patients and those with negative ANAs. T-test and chi-square analysis were performed in the comparison.

**Results:** Antinuclear antibodies were detected in 2129 out of 2809 patients enrolled in the cohort and 4.2% of them were negative. There was significant difference between patients with negative and positive ANAs based on gender (29/60 vs 294/1746, p<0.001). The presence of Raynaud’s phenomenon is less common (71.8% vs 99.8%, p<0.001) in the ANA-negative patients. In addition, compared with ANA-positive patients, the incidence of certain critical organ involvements, including gastroesophageal reflux (5.6% vs 18.5%, p=0.002), interstitial lung disease (65.2% vs 77.9%, p=0.015) and pulmonary hypertension (11.5% vs 29.0%, p=0.006) were significantly lower in ANA-negative patients than in the positive group. The proportion of IgG elevation, an indicator of disease activity, was significantly lower in the ANA-negative patients than that in the positive group ( 14.3% vs 41.2%, p<0.001), while no significant differences were found in other inflammatory indicators and skin scores.

**Conclusions:** This study describes the clinical features of SSc patients with negative ANAs, which have been rarely mentioned in existing studies. Antinuclear antibody is proved to be strongly associated with the clinical manifestations of systemic sclerosis patients and ANA-negative SSc patients tend to be in relatively milder conditions.

## P.040

## CD21LOW B CELLS IN SYSTEMIC SCLEROSIS: A POSSIBLE MARKER OF PULMONARY ARTERIAL HYPERTENSION

C. Pellicano^1^, R. Marrapodi^2^, G. Leodori^1^, G. Radicchio^2^, S. Colantuono^2^, A. Iacolare^1^, A. Gigante^1^, M. Visentini^2^, E. Rosato^1^

^1^*Department of Translational and Precision Medicine-Scleroderma Unit, Sapienza University of Rome, Rome, Italy*, ^2^*Department of Translational and Precision Medicine, Laboratory affiliated t, Sapienza University of Rome, Rome, Italy*

**Introduction:** Systemic Sclerosis (SSc) is characterized by an aberrant activation of the immune system, widespread functional and structural vascular damage and progressive fibrosis of skin and internal organs. One of the main complications of SSc is the development of pulmonary arterial hypertension (PAH). Some SSc patients present an increased percentage of B lymphocytes expressing low levels of CD21 (CD21low cells). Aims of the study were to evaluate whether CD21low B-cells could define a particular subset of patients with SSc and if their percentage could correlate with the development of PAH.

**Material and Methods:** Peripheral blood B-cells subpopulation, echocardiography and pulmonary function test has been assessed in 74 SSc patients (F=64, mean age 54.5 ± 10.9 years) and 20 healthy donors (HD).

**Results:** SSc patients with high percentage of CD21low B-cells have a significantly higher sPAP and forced vital capacity/diffusing capacity of the lung for monoxide carbon (FVC/DLCO) than patient with normal percentage of CD21low; DLCO is lower in SSc-CD21low group. We found a positive linear correlation between CD21low and sPAP (r=0.443; p<0.0001) and FVC/DLCO ratio (r=0.24, p=0.042); conversely, a negative correlation was observed between CD21low and DLCO (r=-0.312; p=0.007).

**Conclusions:** Increased CD21low B-cells in SSc patients define a particular subset of patients with severe visceral vascular manifestations.

## P.041

## TOXIC EXPOSURE SURVEY IN A SYSTEMIC SCLEROSIS COHORT

M. Freire^1^, B. Sopeña^1^, A. Gonzalez-Quintela^1^, C. Tolosa Vilella^2^, A. Guillén Del Castillo^3^, B. Marí Alfonso^2^, J.A. Vargas Hitos^4^, X. Pla Salas^5^, C. González-Echávarri^6^, A. Chamorro^7^, I. Perales Fraile^8^, A. González García^9^, G. de la Red Bellvis^10^, D. Bernal Bello^11^, A. Castro Salomó^12^, I. Jiménez Pérez de Heredia^13^, E. Callejas Moraga^2^, V. Fonollosa Pla^3^, C.P. Simeon Aznar^3^, On Behalf of RESCLE Investigators^14^

^1^*Unit of Autoimmune Diseases, Department of Internal Medicine, Hospital Clínico Universitario de Santiago, Santiago de Compostela, A Coruña, Spain*, ^2^*Department of Internal Medicine. Corporación Sanitaria Universitaria Parc Taulí, Sabadell, Barcelona, Spain*, ^3^*Unit of Autoimmune Diseases, Department of Internal Medicine. Hospital Universitario Vall d’Hebron, Barcelona, Spain*, ^4^*Department of Internal Medicine. Hospital Universitario Virgen de las Nieves, Granada, Spain*, ^5^*Unit of Systemic Autoimmune Diseases, Department of Internal Medicine. Consorci Hospitalari de Vic, Vic, Barcelona, Spain*, ^6^*Autoimmune Diseases Research Unit, Department of Internal Medicine. Biocruces Bizkaia Health Research Institute, Barakaldo, Spain*, ^7^*Department of Internal Medicine. Complejo Asistencial Universitario de Salamanca, Salamanca, Spain*, ^8^*Department of Internal Medicine. Hospital Universitario Rey Juan Carlos, Móstoles, Madrid, Spain*, ^9^*Department of Internal Medicine. Hospital Universitario Ramón y Cajal, Madrid, Spain*, ^10^*Unit of Systemic Autoimmune Diseases, Department of Internal Medicine. Fundacio Hospital de lEsperit Sant, Santa Coloma de Gramenet, Barcelona, Spain*, ^11^*Department of Internal Medicine. Hospital Universitario de Fuenlabrada, Fuenlabrada, Madrid, Spain*, ^12^*Department of Internal Medicine. Hospital Universitario Sant Joan, Reus, Tarragona, Spain*, ^13^*Department of Internal Medicine. Hospital de Sagunto, Sagunto, Valencia, Spain*, ^14^*Autoimmune Diseases Study Group (GEAS), Spain*

**Introduction:** Systemic sclerosis (SSc) is a systemic autoimmune disease with extremely heterogeneous clinical features and unknown etiology, although numerous studies suggest a relationship with environmental and occupational factors. So far there is little information on whether toxic substances can play a relevant role in its phenotypic expression (1).

**Material and Methods:** A survey was conducted aimed at the knowledge of the working life of patients from six centers belonging to the Spanish Scleroderma Registry (RESCLE), categorizing them in six groups: no potential exposure to toxic substances, potential exposure to silica, to hydrocarbons, to organic solvents, to mixed toxics (silica and / or hydrocarbons and / or organic solvents) and to another toxics. In all patients 87 epidemiological, clinical and analytical variables included in the registry were analyzed, carrying out a comparative study.

**Results:** 225 SSc patients were selected. 81 patients (36%) had worked in professions with potential risk of toxic exposure, 64 women out of the 227 included (28%) and 17 men out of the 28 included (60%). The toxic agent most frequently involved was silica in 29 patients (35.8%), followed by hydrocarbons in 21 (25.9%), mixture of toxic substances in 21 other patients ( 25.9%), organic solvents in 4 patients (4.9%) and other toxic in 6 cases (7.4%). Toxic exposure was associated with a lower risk of being female (OR 0.15, p <0.001), having been exposed to tobacco (OR 0.4, p 0.037) and digital ulcers (OR 0.43, p 0.016) and with a greater likelihood of hepatic involvement (OR 3.63, p 0.021), musculoskeletal involvement (OR 2.13, p 0.017) and the slow capillary pattern of Maricq (OR 1.8, p 0.063). Analyzing the exposure groups separately, patients exposed to silica had a lower probability of Raynaud’s phenomenon (OR 0.25, p 0.005) and a higher probability of diagnosis at older ages (OR: 1.04, p 0.005), presence of Topoisomerase I antibody (OR 3.71, p 0.023) and slow capillary pattern of Maricq (OR 4.15, p 0.008). Patients exposed to hydrocarbons had an increased risk of liver involvement (OR 5.34, p 0.029).

**Conclusions:** In our cohort of 225 patients with SSc, 60% of male patients and 28% of women worked in a profession with a potential risk of toxic exposure. In this preliminary study, important differences were observed in the probability of a different phenotypic expression of the SSc according to the history of occupational exposure to toxins.

## P.042

## IMPROVING CLASSIFICATION CRITERIA FOR SYSTEMIC SCLEROSIS, A EUSTAR STUDY

M. Elhai^1^, M. Boubaya^2^, E. Siegert^3^, V. Riccieri^4^, P. Airò^5^, O. Distler^6^, Y. Allanore^1^

^1^*Paris Descartes University, INSERM U1016, Sorbonne Paris Cité, Rheumatology A department, Cochin Hospital, Paris, France*, ^2^*Unit of Clinical Research, Paris Seine Saint Denis University, Bobigny, France*, ^3^*Department of Rheumatology, Charité University Hospital, Berlin, Germany*, ^4^*Dipartimento di Medicina Interna e Specialità Mediche, Sapienza Università di Roma, Rome, Italy*, ^5^*Spedali Civili di Brescia Servizio di Reumatologia Allergologia e Immunologia Clinica, Brescia, Italy*, ^6^*Department of Rheumatology, University Hospital Zürich, Zürich, Switzerland*

**Introduction:** Systemic sclerosis (SSc) patients are stratified according to the extent of skin involvement into diffuse (dcSSc), limited cutaneous (lcSSc) and sine scleroderma subtypes. This classification remains inaccurate to capture disease heterogeneity and is not stable with time, particularly in the early stages, when skin disease has not reached its peak. Autoantibodies are detected in more than 90% of the patients, can be identified even before the disease is overt, with three predominant and specific ones: anticentromere (ACA), anti-Scl70 and anti-RNA polymerases III antibodies. We aimed to compare performances of classification into cutaneous subtypes versus auto-antibody status according to (i) organ involvements and (ii) survival.

**Material and Methods:** We extracted data from EUSTAR database in July 2019. Patients were classified either as (i) lcSSc, dcSSc or sine scleroderma (by their physician) or (ii) according to autoantibodies. The respective performance of each model was assessed using multivariable logistic regressions and the two models were compared by the area under the curve (AUC) and the net reclassification improvement (NRI). To account for missing data, analyses were conducted using multiple imputations.

**Results:** 10711 patients were included: 84.6% of males, mean age: 56.3±13.9 years and mean disease duration: 9.9 ± 8.7 years. 64.2% were classified as lcSSc, 28.4% as dcSSc and 7.4% sine scleroderma, whereas 31.7% had isolated positive and unspecific ANA, 34.5% ACA, 26.1% Scl70 antibodies, 2.9% RNA pol III antibodies and 4.7% no antibody.

At inclusion, 23% and 13.8% had previous and current digital ulcer (DU) respectively, 38.2% lung fibrosis on HRCT, 1.9% renal crisis, 3.1% LVEF<50% and 0.9% PAH on right heart catheterization. In cross-sectional analysis, the two models had similar performance to diagnose DU, gastrointestinal, musculoskeletal, renal involvements and LVEF<50%. However, the “antibody model” was more performant to diagnose lung fibrosis (AUC: 0.771 vs 0.727, p<0.0001) with NRI showing improvement in classification of 21% of patients (vs 20% of misclassified patients in the “cutaneous form model”). The “antibody model” also trended to better diagnose PAH (AUC: 0.831 vs 0.821, p between 0.01 and 0.08 in the different imputation models), with a worse identification of patients without PAH using the “cutaneous form model” (29% of misclassified patients). The NRI showed improvement in classification for survivors using the “antibody model” (37% vs 19%).

**Conclusions:** Classifying SSc patients according to autoantibodies has a higher performance to diagnose lung fibrosis and improves classification of patients for PAH and survival compared to skin involvement’s classification. Additional longitudinal analyses are pending.

## P.043

## SYSTEMIC SCLERODERMA: CLINICO-IMMUNOLOGICAL CORRELATION

K. Echchilali^1^, K. Ouazahrou^2^, H. Elkabli^1^, A.A. Bousfiha^2^

^1^*Internal Medicine Department, Casablanca, Morocco*, ^2^*Laboratory of Clinical Immunology, Inflammation and Allergy, Casablanca, Morocco*

**Introduction:** Systemic sclerosis (SSc) is a multisystem connective tissue disorder characterized by excessive fibrosis of skin and internal organs and microvascular damage. Autoimmunity is considered to be involved in the pathophysiology. Autoantibodies do not seem to be simply epiphenomena, but are involved in disease pathogenesis. It is believed that the SSc-specific autoantibodies are responsible both for amplifying immune response and targeting cell types that are relevant in the pathophysiology of SSc. The aim of this study is to correlate the profile of the following specific autoantibodies with clinical and laboratory manifestations of SSc in Morroco.

**Material and Methods:** The occurrence of specific autoantibodies in 30 patients with SSc was investigated by indirect immunofluorescence (IIF) and Immunodot, correlating the type of autoantibody with clinical and laboratory manifestations.

**Results:** Among all patients evaluated, we found a predominance of females (90%), meanage 50 years old, Caucasian (100%), time of diagnosis between 1 and 10 years (50%), and disease duration of 9,5 years. According to the specific autoantibody profile, 4 patients were ACA-positive (10,25%), 12 were positive for anti-topoI (30,7%), 5 were positive for anti-RNP (12,8%), 5 were positive for anti PM-Scl 100 (12,8%), 4 were positive sor anti Ro-52 (10,25%), 2 showed positive for anti SSA (5,12%), 1 patient was positif for anti fibrillarine (2,5%), 1 patient for anti Jo1(2,5) and 1 patient for anti PM-Scl 75 (2,5) . The anti-topo I autoantibody correlated with diffuse scleroderma, with greater disease severity and activity, with worse quality of life measured by the SHAQ index, with a higher prevalence of objective Raynaud’s phenomenon and digital pitting scars of fingertips. The ACA correlated with limited scleroderma, with earlier onset of disease .The anti-RNP correlated with diffuse scleroderma.

**Conclusions:** The clinical subtype of the disease and some clinical manifestations in SSc may correlate positively with the presence of specific autoantibodies.

## P.044

## LONG NON-CODING RNA HOTAIR INDUCES MYOFIBROBLAST ACTIVATION IN SYSTEMIC SCLEROSIS THROUGH EZH2 DEPENDENT DE-REPRESSION OF NOTCH SIGNALLING PATHWAY ACTIVATION

F. Del Galdo^1^, C. Wasson^1^, G. Abignano^2^, R. Ross^1^, H. Hermes^3^, S. Jimenez^3^

^1^*Institute of Rheumatic and Musculoskeletal Medicine and Biomedical Research Centre, University of Leeds, Leeds, United Kingdom*, ^2^*Rheumatology Institute of Lucania (IReL), Rheumatology Department of Lucania, San Carlo Hospital, Potenza, Italy*, ^3^*Thomas Jefferson University, Philadelphia, USA*

**Introduction:** Fibroblasts explanted from affected tissues in Systemic Sclerosis (SSc), conserve their pro-fibrotic phenotype in vitro. Long non-coding RNAs (lncRNAs) within the HOX loci have been described as master epigenetic regulators within the connective tissue. Specifically, HOTAIR has been shown to have increased expression at the hands and feet which are also the first body regions affected by SSc. Here we aimed to unravel the mechanisms responsible for the epigenetically stable activation of SSc fibroblasts.

**Material and Methods:** Dermal fibroblasts were isolated from forearm skin biopsies of twelve adult patients with SSc of recent onset. Laser capture was performed to isolate cells expressing or not α-SMA as a marker of myofibroblast differentiation. HOTAIR was expressed in healthy dermal fibroblasts by lentiviral induction employing a vector containing the specific sequence. Gamma secretase inhibitors RO4929097 and DAPT were employed to block Notch signalling in SSc fibroblasts. EZH2 was blocked in SSc fibroblasts with the specific inhibitor GSK126.

**Results:** HOTAIR expression was increased in SSc patients’ skin (100 fold) and in SSc explanted fibroblasts (5 fold; p<0.001 for both). Further, laser captured α-SMA expressing myofibroblasts expressed in average 2.5 fold greater HOTAIR RNA levels compared to α -SMA negative cells from the same donors (P<0.05). In vitro, we demonstrated that overexpression of HOTAIR in healthy dermal fibroblasts led to increased expression of Col1A1 and α-SMA both at mRNA and protein levels (2.8 and 1.8 fold respectively, p<0.05). We further showed that HOTAIR-induced profibrotic activation was due to EZH2 dependent spread of H3k27me3 methylation marker, as demonstrated by complete inhibition by treatment with GSK126. Additionally, we showed that EZH2 led to profibrotic activation by decreasing the expression of miRNA34a. The reduced miRNA 34a levels in turn led to NOTCH increased expression and signalling pathway activation. Consistent with these findings, treatment of HOTAIR expressing cells with two different types of gamma secretase inhibitors known to block NOTCH activation, completely suppressed the profibrotic effects induced by HOTAIR overexpression. Importantly, treatment with either GSK126 or gamma secretase inhibitors suppressed collagen production in the HOTAIR expressing cells as well SSc fibroblasts by 30% and 50% respectively (p<0.05).

**Conclusions:** Here we show that the epigenetically stable activation of SSc dermal fibroblasts is due to HOTAIR led EZH2 dependent de-repression of NOTCH signalling. The results of these studies identify a new venue to modulate fibroblasts biology which could inform clinical research to resolve chronic fibrosis and re-establish tissue homeostasis in SSc.

## P.045

## TGFBETA PROMOTES DNA METHYLATION OF THE SOCS3 PROMOTER TO INDUCE FIBROBLAST ACTIVATION AND TISSUE FIBROSIS IN SSC

C. Dees^1^, S. Pötter^1^, Y. Zhang^1^, C. Bergmann^1^, M. Luber^1^, T. Wohlfahrt^1^, A. Ramming^1^, O. Distler^2^, G. Schett^1^, J. Distler^1^

^1^*Friedrich-Alexander-University (FAU) Erlangen-Nürnberg, Department of Internal Medicine 3, Universitätsklinikum Erlangen, Erlangen, Germany*, ^2^*Rheumaklinik, University Hospital Zurich, Zurich, Switzerland*

**Introduction:** Pathological activation of fibroblasts releasing large amounts of extracellular matrix proteins is a key feature of systemic sclerosis (SSc). Epigenetic gene silencing of anti-fibrotic genes is thought to play a central role to establish the persistently activated phenotype of fibroblasts. In the present study, we evaluated whether the aberrant activation of JAK2-STAT3 signaling in fibrosis might be caused by epigenetic silencing of SOCS expression.

**Material and Methods:** 5-aza-2’-deoxycytidine (5-aza) was used to inhibit DNA methyltransferases (DNMTs) in vitro and in vivo. DNA methylation was evaluated by methylation-specific PCR and MeDIP assays. Knockdown analyses were done by transfection of siRNA and fibroblast-specific knockout mice.

**Results:** The expression of SOCS3 was severely downregulated in skin of SSc patients compared to healthy individuals with only minor differences between limited and diffuse cutaneous SSc. The SSc-like phenotype could be mimicked in normal fibroblasts by chronically increased levels of TGFβ with reduced levels of SOCS3. Evaluation of DNA methylation demonstrated prominent promoter hypermethylation of SOCS3 in SSc fibroblasts and in normal fibroblasts exposed to persistently high levels of TGFβ. Mechanistically, chronic exposure to TGFβ induced an increase in DNMT activity and a time-dependent induction of DNMT3A and DNMT1 expression resulting in promoter hypermethylation of SOCS3. Inhibition of the DNMTs genetically by siRNA or pharmacologically by 5-aza restored the expression of SOCS3 and reduced fibroblast activation and collagen release. Knockdown of SOCS3 induced an SSc-like phenotype in normal dermal fibroblasts and aggravated experimental tissue fibrosis with increased activation of JAK2-STAT3 signaling. Vice versa, forced overexpression of SOCS3 inhibited TGFβ-mediated fibroblast activation, ameliorated the endogenous activation of SSc fibroblasts, and reduced TGFβ-induced activation of STAT3. Moreover, treatment with DNMT inhibitors or fibroblast-specific knockout of DNMT3A re-activated the expression of SOCS3, reduced JAK2-STAT3 signaling and exerted potent antifibrotic effects in bleomycin- and TBRIact-induced dermal fibrosis. In addition, treatment with 5-aza or knockdown of either DNMT1 or DNMT3A induced regression of established fibrosis.

**Conclusions:** We demonstrate that chronic activation of TGFβ signaling causes DNMT-induced silencing of SOCS3 expression in SSc resulting in perturbation of the epigenetic control of STAT signaling. Re-activation of the endogenous regulation of STAT signaling prevents aberrant STAT3 signaling, inhibits TGFβ-induced fibroblast activation and collagen release, ameliorates experimental fibrosis and induced regression of established fibrosis.

## P.046

## IL-1 BETA-STIMULATED MICROVASCULAR ENDOTHELIAL CELLS PROMOTE M2-LIKE MACROPHAGES: CONSEQUENCES ON FIBROBLASTS ACTIVATION DURING SCLERODERMA

C. Contin-Bordes^1^, P. Laurent^1^, J. Lapoirie^3^, B. Jurado-Mestre^1^, E. Levionnois^1^, M.-E. Truchetet^1,4^

^1^*CNRS-UMR5164- ImmunoConcEpT, Bordeaux, France*, ^2^*Bordeaux University Hospital - Department of Immunology and Immunogenetics, Bordeaux, France*, ^3^*Bordeaux University Hospital - Department of Internal Medicine, Bordeaux, France*, ^4^*Bordeaux University Hospital - Department of Rheumatology, Bordeaux, France*

**Introduction:** Interaction between platelets and microvascular endothelial cells (MEC) has emerged as a new physiopathological loop in scleroderma fibrosis. We previously show that during early scleroderma, platelets-derived IL-1β induces the production of the pro-fibrotic factor Thymic Stromal Lymphopoïetin (TSLP) by MEC. Type-2 macrophages are important players in the pathophysiology of scleroderma. However, the precise mechanisms involved in their recruitment and/or polarization are not yet elucidated. We therefore aimed at analysing the contribution of IL-1β-activated MEC on the polarization of monocytes/macrophages and its consequences in fibroblast activation during scleroderma.

**Material and Methods:** Patients were included in the context of the “Vasculopathy and Inflammation in Systemic Sclerosis” biomedical research project founded in 2012 (institutional ethical committee CPP, 2012-A00081-42, Aquitaine) after they gave their informed consent. Punch biopsy specimens of affected mid-forearm skin were obtained for some patients. Skins from plastic surgery (brachioplasty or abdominoplasty) were taken as control. Quantification of mRNA transcript in paraffine-embedded skin biopsies from 24 SSc patients and 10 HD was performed by nanostring technology using the panCancer Immune profiling kit. MEC and fibroblasts were purified from skin of SSc patients and/or HD for cell culture experiments (monocyte-derived macrophage polarization test, fibroblasts activation test). Age- and sex-matched healthy donors (HD) were recruited at the local Blood Transfusion Centre (Etablissement Français du sang, Bordeaux) for monocytes immunomagnetic separation using CD4+ beads and serum cytokine measurements.

**Results:** Transcriptomic analysis of skin biopsies revealed a strong type-2 macrophages signature in SSc patients, encompassing CD68, cMAF, CCL18 and DC-SIGN expression. These data were confirmed in skin biopsies, where CD68+cMAF+ and CD68+DC-SIGN+ cells are increased in SSc patients compared to HD. In vitro, IL-1β-activated MEC from SSc patients induced a M2-like profile in macrophages reminiscent of intermediate macrophages, as they express pSTAT3, CD163 and DC-SIGN and produce IL-10, CCL18 and IL6. IL-6 and endothelin-1 (ET-1) derived from IL-1β-activated MEC were key players in this process. In turn, M2-like induced cells promote the expression of MMP-1 and CCL-2 transcripts in fibroblasts.

**Conclusions:** This work revealed a new pathway in which platelet-derived factors promote the activation of endothelial cells that shapes type-2 macrophage polarization. This process contributes to fibroblasts activation and production of pro-remodelling and monocytes-chemoattractant factors. Those results reinforced the concept of limiting platelets activation as new therapeutic perspectives notably in early form of the disease.

## P.047

## EXOSOMES IN SYSTEMIC SCLEROSIS (SSC): HOW TO SPREAD THE PRO-FIBROTIC SIGNALLING IN THE DISEASE

C. Corallo^1^, M. Cutolo^2^, S. Soldano^2^, N. Giordano^1^

^1^*Scleroderma Unit, Department of Medicine, Surgery and Neurosciences, University of Siena, Siena, Italy*, ^2^*Research Laboratory and Academic Division of Clinical Rheumatology, Department of Internal Medicine, University of Genoa, Genoa, Italy*

**Introduction:** Exosomes are lipid-like microvesicles containing a cargo of biomarkers, such as proteins, metabolites and nucleic acids, including microRNA (miRNA). They are implicated in intercellular communication by fusing and releasing their cargo into the target cells. In the present study, we evaluated the potential of exosomes deriving from plasma of SSc patients or generating from cultured SSc fibroblasts to drive the fibrotic signalling in the disease.

**Material and Methods:** Exosomes were isolated from plasma of n=10 SSc patients and from n=10 control subjects. Exosomes were also purified from cell culture supernatants of SSc fibroblasts and of control fibroblasts. Exosome size and concentration were assessed by Nanosight-Particle-Tracking-Analysis (NTA) and by transmission-electron-microscopy (TEM). The content of anti-fibrotic (let-7a, 146a, 200a, 223a) and pro-fibrotic (150, 155) miRNAs was analyzed in all plasma-derived and cell culture-derived exosome populations by semiquantitative real-time-PCR. Finally, the isolated exosomes were used to stimulate control dermal fibroblasts in culture. Gene expressions (COL1A1, ACTA2 and TAGLN) were assessed by quantitative real time PCR (qRT-PCR) and the respective protein levels (type-I-collagen, α-SMA and SM22) by immunofluorescence (IF).

**Results:** Exosomes isolated from SSc plasma samples showed higher concentration (3.3x10^10±1.1x10^10 particles/mL) with respect to control plasma ones (1.5x10^10±0.4x10^10 particles/mL) (p<0.01). Size did not differ between SSc and control plasma samples ranging from 50nm to 150nm. Similar results were obtained with exosomes generated from fibroblasts: the concentration was higher in SSc fibroblasts (1.1x10^10±0.2x10^10 particles/mL) than in control ones (0.4x10^10±0.1x10^10 particles/mL) (p<0.05) with no significant differences in size distribution. The content of anti-fibrotic (let-7a, 146a, 200a, 223a) miRNAs was decreased in exosomes from both SSc plasma samples and from SSc fibroblasts with respect to control plasma samples (p<0.05) and to control fibroblasts (p<0.05). The pro-fibrotic (150, 155) miRNAs were significantly upregulated in exosomes deriving from SSc plasma samples and from SSc fibroblasts, with respect to control plasma samples (p<0.05) and to control fibroblasts (p<0.05). Finally, only exosomes coming from SSc plasma samples or SSc fibroblast cultures were able to induce pro-fibrotic gene (COL1A1, ACTA2 and TAGLN) and protein (type-I-collagen, α-SMA and SM22) expression in control fibroblasts.

**Conclusions:** This study demonstrates that plasma from SSc patients contains higher concentration of exosomes compared to plasma from control subjects and that SSc-derived exosomes contain specific pro-fibrotic miRNA signatures. These results suggest that exosomes could be fibrotic drivers towards non-affected areas in vivo, and therefore they might represent novel targets for the development of anti-fibrotic treatments in SSc.

## P.048

## SERUM MARKER ANALYSIS SUGGESTS SUBSET AND STAGE SPECIFIC PROFILES OF FIBROGENESIS ACROSS THE SCLERODERMA SPECTRUM

K.E.N. Clark^1^, C. Campochiaro^1^, K. Nevin^2^, E. Csomor^2^, N. Galwey^2^, M. Morse^2^, N. Wisniacki^2^, S. Flint^2^, V.H. Ong^1^, E. Derrett-Smith^1^, C.P. Denton^1^

^1^*Department of Rheumatology and Connective Tissue Diseases, UCL, London, United Kingdom*, ^2^*Immuno-Inflammation, GlaxoSmithKline, Stevenage, United Kingdom*

**Introduction:** Systemic sclerosis is characterised by autoimmunity, fibrosis and vasculopathy. There is striking heterogeneity in skin fibrosis that is likely to reflect the balance between pro- and anti-fibrotic pathways underlying spontaneous regression of skin fibrosis in late stage diffuse SSc. We have studied potential serum markers of profibrotic activity in SSc, with a view to understanding their relationship with progression of fibrosis as measured by the modified Rodnan skin score (MRSS) to better define cases likely to respond to fibrosis-targeted therapies.

**Material and Methods:** We prospectively recruited a cohort of well characterised patients (the BIOPSY cohort) from across the scleroderma spectrum. In total 67 patients had adequate serum and plasma samples to be included in the analysis (21 early dcSSc (<5 years disease duration), 14 established dcSSc, 16 lcSSc, 16 healthy controls (HC)). MRSS was recorded at the time of sample collection. Standard and novel measures of serum or plasma markers were undertaken by immunoassay (20 in total) reflecting extracellular matrix (ECM) turnover or cytokine drivers of fibrosis.

**Results:** Our results confirmed that 13 analytes showed significant differences in concentration by subgroup using one-way ANOVA. Markers of collagen synthesis were significantly different between the subgroups (Pro-C6, Pro-C3, PIIINP), while markers of collagen degradation were not significantly altered (C3M, C6M, C4M2, C7M). The C3 fibrotic index (Pro-C3:C3M) showed more significant differentiation between subgroups compared to the C6 fibrotic index. Across all tests, this difference was most significant between the early dcSSc subgroup compared with the other subgroups. There was significant upregulation of IL-6, MCP-1, and oncostatin M in SSc compared to HC. Consistent with other reports the ELF score, was significantly higher in the SSc patient cohort. There was significant correlation between several candidate profibrotic serum markers and MRSS: Pro-C3, Pro C6, PIIINP, and IL6 (all p<0.01).

**Conclusions:** Our results show the utility of extended patient cohorts to delineate fundamental biology in SSc. We identify key pro-fibrotic molecular markers upregulated in SSc and correlated these to extent of skin fibrosis. Markers of collagen III and collagen VI synthesis are particularly raised, especially in the early stages of the disease. We did not find any significant difference between SSc subgroups and healthy controls for markers of collagen degradation. These promising cross-sectional data suggest that therapies targeting drivers of fibrosis are most likely to show benefit for skin in early dcSSc. This will be further explored longitudinally in the BIOPSY cohort.

## P.049

## ANTIBODIES VERSUS SKIN FIBROSIS EXTENSION IN SYSTEMIC SCLEROSIS: A CASE-CONTROL STUDY

A. Tieu, B. Chaigne, B. Dunogue, J. Dion, A. Regent, P. Legendre, C. Le Jeunne, L. Mouthon

*Department of Internal Medicine, APHP, COCHIN, Paris, France*

**Introduction:** Little is known of anti-centromere antibodies (ACA) diffuse cutaneous systemic sclerosis (dSSc) and of anti-Scl70 antibodies (Scl70) limited cutaneous systemic sclerosis (lSSc).

**Material and Methods:** A retrospective monocentric case control study of patients with SSc fulfilling ACR/EULAR classification criteria was performed. Patients with dSSc and ACA or patients with lSSc and Scl70 were included in the study and compared to patients with ACA lSSc and Scl70 dSSc.

**Results:** Of 1040 patients followed in Cochin University Hospital Internal Medicine department, 12 (1.1%) patients had ACA dSSc and 93 (8.9%) patients had Scl70 lSSc.

Patients with ACA dSSc had a more severe disease than patients with ACA lSSc including a larger skin extension (p<0.01) and more frequent organ involvement (p<0.05) than patients with ACA lSSc. Oppositely, these patients had less frequently interstitial lung disease (ILD) (p<0.01) than patients with Scl70 dSSc. After a median follow-up of 5 years, patients with ACA dSSc remained with a more important skin extension (p<0.01) than patients with ACA lSSc and with less frequent (p=0.05) ILD than patients with Scl70 dSSc.

Patients with Scl70 lSSc had a more severe disease than patients with ACA lSSc including a more important skin extension (p< 0,0001) and more frequent ILD (p< 0,0001) than patients with ACA lSSc. Oppositely, these patients had a more limited skin extension (p< 0,0001) and less organ involvement (p<0.01) than patients with Scl70 dSSc. After a median follow-up of 5 years, patients with Scl70 lSSc remained with more frequent (p< 0,0001) and more severe (p<0.0001) ILD than patients with ACA lSSc but less severe (p<0.01) than Scl70 dSSc patients.

Patients’ survival rate was different between the four groups, Scl70 dSSc patients having the worst prognosis (p< 0.01). Interestingly, ACA SSc patients’ survival did not differ from Scl70 SSc patients’ survival (p=0.122) whereas dSSc patients’ survival differed from lSSc patients’ survival (p< 0.001).

**Conclusions:** Scl70 lSSc and ACA dSSc are rare subgroups of SSc and show intermediate patterns of SSc. Scl70 antibodies are associated to ILD regardless of skin extension which determines the severity and the prognosis of the disease.

## P.050

## CLINICAL AND IMMUNOLOGICAL CHARACTERISTICS OF PATIENTS WITH SYSTEMIC SCLEROSIS AND CANCER: RESULTS OF THE SPANISH REGISTRY OF SCLERODERMIA (RESCLE)

C. Carbonell^1^, A.-J. Chamorro^1^, P. Segovia Alonso^1^, C. Tolosa Vilella^2^, L. Trapiella Martínez^3^, D. Colunga Argüelles^4^, L. Sáez Comet^5^, M. Rubio Rivas^6^, A. Argibay^7^, A.B. Madroñero Vuelta^8^, M.E. Sánchez García^9^, M. Ruiz Muñoz^10^, F.J. García Hernández^11^, G. Espinosa^12^, J.J. Ríos Blanco^13^, E. Callejas Moraga^2^, A. Guillén Del Castillo^14^, V. Fonollosa Pla^14^, C.P. Simeón Aznar^14^, On Behalf of RESCLE Investigators^15^

^1^*Department of Internal Medicine. Complejo Asistencial Universitario de Salamanca, Salamanca, Spain*, ^2^*Department of Internal Medicine. Corporación Sanitaria Universitaria Parc Taulí, Sabadell, Barcelona, Spain*, ^3^*Unit of Systemic Autoimmune Diseases, Department of Internal Medicine. Hospital de Cabueñes, Gijón, Asturias, Spain*, ^4^*Department of Internal Medicine. Hospital Universitario Central de Asturias, Oviedo, Asturias, Spain*, ^5^*Department of Internal Medicine. Hospital Universitario Miguel Servet, Zaragoza, Spain*, ^6^*Unit of Autoimmune Diseases, Department of Internal Medicine. Hospital Universitario de Bellvitge-IDIBELL, L’Hospitalet de Llobregat, Barcelona, Spain*, ^7^*Unit of Systemic Autoimmune Diseases and Thrombosis. Department of Internal Medicine. Complejo Hospitalario Universitari, Vigo, Pontevedra, Spain*, ^8^*Department of Internal Medicine. Hospital General San Jorge, Huesca, Spain*, ^9^*Department of Internal Medicine. Hospital Universitario Virgen de Valme, Sevilla, Spain*, ^10^*Department of Internal Medicine. Hospital Universitario Fundación Alcorcón, Alcorcón, Madrid, Spain*, ^11^*Department of Internal Medicine. Hospital Universitario Virgen del Rocío, Sevilla, Spain*, ^12^*Department of Autoimmune Diseases. Hospital Clinic, Barcelona, Spain*, ^13^*Department of Internal Medicine. Hospital Universitario La Paz, Madrid, Spain*, ^14^*Unit of Autoimmune Diseases, Department of Internal Medicine. Hospital Universitario Vall d’Hebron, Barcelona, Spain*, ^15^*Autoimmune Diseases Study Group (GEAS), Spain*

**Introduction:** Prior literature shows an increased risk of cancer among patients with systemic sclerosis (SSc) and cancer is a growing cause of non-SSc related death among these patients. Therefore, the aim of this work was to describe the frequency, characteristics and risk factors for cancer among Spanish patients with SSc.

**Material and Methods:** Ambispective, multicenter and analytical study of patients with a SSc according to ACR / EULAR criteria and/or LeRoy classification with and without neoplasia from Spanish Scleroderma Registry (RESCLE) (from 2006 to 2018).

**Results:** Among 1930 patients with SSc, 206 of them had cancer. The most frequent malignancies were breast (47/206 [22.8%]), lung (29/206 [14.1%]), hematological (20/206 [9.7%]) and colorectal (18/206 [8.7%]). Synchronous neoplasms were found in 72/206 cases (4.0%). One hundred thirty-three (70%) cases were diagnosed after the diagnosis of SSc. From the clinical point of view, patients with and without neoplasia were compared and patients with cancer had a later onset of SSc (45.9 [SD 16.1] years) compared with patients without cancer (49.3 [SD 17.0], P <0.001). Cancer patients had a significantly higher diagnosis of primary biliary cirrhosis (PBC) (72/1709 [4.2%] vs. 18/202 [8.9%], P =0.007), interstitial lung disease (694/1715 [40.5%] vs. 108/204 [52.9%], P <0.001), pulmonary hypertension (262/917 [28.6%] vs. 44/107 [41.1%], P =0.010), and a higher proportion of cases with forced vital capacity (FVC) <70% (316 / 1482 [21.3%] vs. 57/173 [32.9%], P <0.001).

Regarding autoimmunity profile, patients with SSc and cancer had a significantly higher presence of anti-RNA polymerase III antibodies (39/376 [10,4%] vs. 10/42 [23.8%], P =0.019) and anti PM-Scl (65/919 [7.0%] vs. 15/115 [13.0%], P =0.024) and lower frequency of ACA (73/178 [41.0%] vs. 761/1546 [49.2%], P =0.033). Multivariable regression analysis showed an independent and significant association between the presence of PBC (OR 2.35; 95% CI 1.18-4.68; P = 0.015), CVF <70% (OR = 1.83; 95% CI 1.24-2.70; P = 0.002) and the absence of the ACA (OR 0.66, 95% CI 0.45-0.97; P = 0.036) with the overall development of cancer.

**Conclusions:** Breast, lung, blood and colorectal were the most frequent cancer locations in SSc. A late onset of disease, greater pulmonary involvement, a history of CBP, the presence of RNA polymerase III and PM-Scl antibodies, as well as the absence of ACAs were associated with the development of cancer in our multicenter registry.

## P.051

## CO-EXISTENCE OF SSC HALLMARK AUTOANTIBODIES ASSOCIATES WITH DISTINCT CLINICAL PHENOTYPE

C. Campochiaro, K. Clark, L. Host, S. Alper, S. Nihtyanova, C. Denton, V. Ong

*Centre for Rheumatology and Connective Tissue Diseases, UCL Medical School, Royal Free Campus, London, United Kingdom*

**Introduction:** Systemic sclerosis(SSc) is characterized by the mutually exclusivity of the SSc-specific(SSc-Abs): anti-topoisomeraseI(ATA),anti-centromere(ACA),anti-RNApolymeraseIII(ARA),anti-U3RNP(U3RNP),anti-U1RNP(U1RNP),anti-PmScl(PmScl),anti-Ku(Ku) and anti-Th/T0(Th/T0) with distinct clinical features and prognosis. The presence of a >1 SSc-Abs positivity is considered rare and few data are available about the clinical phenotype of these patients.

**Material and Methods:** The autoantibody profiles of 2799 SSc patients from February 2001 to June 2017 were retrospectively reviewed. Patients with >1 positivity at any time for SSc-Abs were identified. Clinical features were collected and compared to historical cohorts of SSc patients with single SSc-Ab positivity. We excluded patients who had been treated with rituximab, iv immunoglobulins or stem cell transplantation prior to the immunology tests. Two-sided Fisher’s exact test and Mann-Whitney U test were used to perform comparisons. A p-value <0.05 was considered statistically significant.

**Results:** We identified 72 patients(2.6%) with >1 SSc-Ab positivity. Full clinical data were available for 63 patients. 60 patients(2.1%) had double Ab positivity and 3 patients had triple Ab positivity(0.1%). We found a total of 13 possible Ab combinations (Table 1). U1RNP and ATA was the most common cohort(35%), patients were significantly younger (51.38±11.56years) than both U1RNP (58.64 ±13.10years, p=0.050) and ATA(62.03±15.04 years, p=0.002) patients and more commonly of diffuse cutaneous subset(76%v 21%, p=0.001 and 75% vs 52%, p=0.041 respectively). Compared to ATA patients they had significantly more frequently overlap features(43%vs15%, p=0.004) including inflammatory arthritis(29%vs10%, p=0.025) and myositis(19%vs4%, p=0.013). U1RNP and ACA(8 patients, 13%) had a significantly higher prevalence of pulmonary arterial hypertension(50%) compared to both U1RNP(16%, p=0.039) and ACA(15%, p=0.022) patients. Compared to ACA patients they were also significantly younger(57.88±10.87vs68.75±12.61,p=0.015) and more frequently affected by myositis(37%vs1%, p=0.001). U1RNP and ARA(4 patients,7%) patients had no overlap features compared to U1RNP patients (0vs53%, p=0.050) and were more frequently of diffuse cutaneous subtype compared to U1RNP patients(75%vs21%, p=0.040). U1RNP and PmScl patients had a higher prevalence of skeletal myositis compared to U1RNP patients (100%vs16%, p=0.006). ATA and ACA(n=4, 7%) patients behaved more similarly to ATA than ACA patients as they had a significantly higher prevalence of lung fibrosis compared to ACA(75%vs11%,p=0.006) and of skeletal myositis (25%vs1%,p=0.041). ACA and PmScl(4 patients, 7%) had higher prevalence of myositis compared to ACA patients(25%vs1%,p=0.04).

**Conclusions:** Coexistence of hallmark autoantibodies is exceedingly rare in SSc patients. When combined, both SSc-Abs have the potential to synergistically interact and modify the clinical phenotype.

**Figure:**
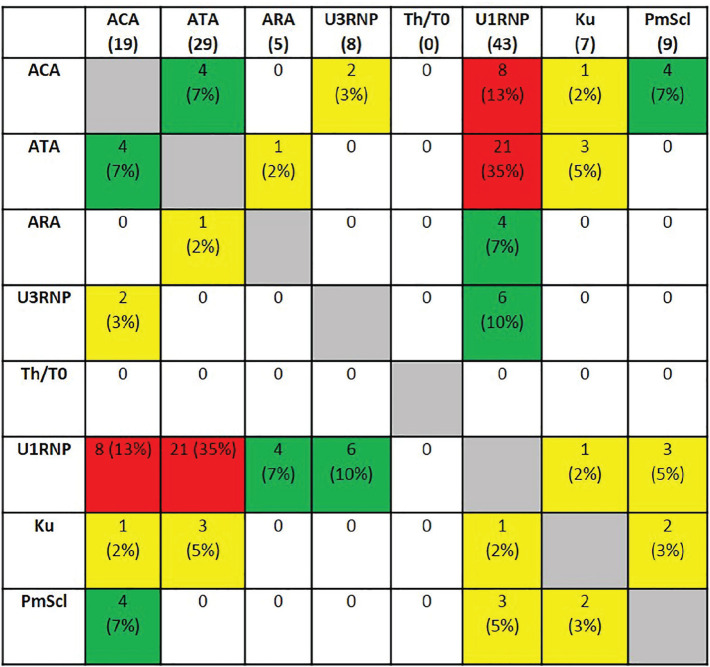


## P.052

## EVALUATION OF TWO DISTINCT ASSAYS FOR DETECTION OF ANTI-TH/TO ANTIBODIES IN SYSTEMIC SCLEROSIS: A CROSS-SECTIONAL STUDY

E.L. Callejas-Moraga^1^, J. Perurena-Prieto^2^, A. Guillén-Del-Castillo^3^, L. Viñas-Gimenez^2^, A. Marín-Sánchez^2^, E. Balada-Prades^5^, V. Fonollosa-Plá^3^, M. Sanz-Martinez^2^, M. Mahler^4^, C. Simeón-Aznar^3^

^1^*Unidad de Enfermedades Autoinmunes Sistémicas. Servicio de Medicina Interna. Hospital Universitari Parc Tauli, Sabdell, Barcelona, Spain*, ^2^*Servicio de Inmunología. Hospital Universitario Vall d’Hebron, Barcelona, Spain*, ^3^*Unidad de Enfermedades Autoinmunes Sistémicas. Servicio de Medicina Interna. Hospital Universitario Vall dHebron, Barcelona, Spain*, ^4^*Research and Development, Inova Diagnostics, San Diego, USA*, ^5^*Inmunomediadas y Terapias Innovadoras. Enfermedades Sistémicas. Vall dHebron Institut de Recerca, Barcelona, Spain*

**Introduction:** Antibodies (Abs) directed against a macromolecular complex consisting of ribonucleoproteins as well as catalytic RNA commonly referred to as anti-Th/To have been described in Systemic Sclerosis (SSc).

Objectives: Comparative evaluation of a commercial line immunoassay (LIA) and a novel particle-based multi-analyte technology (PMAT) system for presence of anti-Th/To Abs and correlation with the clinical manifestations.

**Material and Methods:** In total, 205 clinically established SSc serum samples from the Vall d’Hebron University Hospital were tested for anti-Th/To Abs using LIA and PMAT systems. LIA utilizes the hPop1 as Th/To antigen whereas the PMAT system incorporates Rpp25 and Rpp38 components. Other SSc associated Abs were detected by LIA and indirect immunofluorescence using HEp-2 cells.

**Results:** Eight and 13 patients were positive by LIA and PMAT, respectively. Globally, 21 (10.2%) patients were anti-Th/To positive. Poor agreement between both assays was found (Cohen’s kappa < 0.2). Other SSc associated Abs were found in 15 (71.4%) anti-Th/To positive individuals. Patients that were only positive for anti-Th/To Abs showed a negative association with arthralgia (0% Vs. 54%, p=0.011) and Raynaud’s Phenomenon (66.7% Vs. 98.5%, p=0.007). Moreover, they presented a significantly higher prevalence of myositis (33% Vs. 1%, p=0.013) and interstitial lung disease (ILD) (67% Vs. 11%, p=0.005) when compared to anti-centromere (ACA) positive individuals (n=75).

**Conclusions:** Low concordance between both techniques could be explained by the use of different antigens of the same complex as well as pointing out the complementarity of the assays. When considering all the anti-Th/To positive individuals scarce significantly clinical and epidemiological differences were found. However, patients that were only positive for anti-Th/To antibodies showed interesting clinical associations compared to the anti-Th/To negative group and ACA positive group. Further studies are needed to confirm these results.

## P.053

## COEXISTENCE OF AUTOANTIBODIES IN SYSTEMIC SCLEROSIS: FREQUENCY AND ASSOCIATION WITH CLINICAL CHARACTERISTICS

J. Caetano^1^, F. Paula^1,2^, M. Amaral^1,2^, S. Oliveira^1^, J. Delgado Alves^1,2^

^1^*Systemic Immune mediated Diseases Unit, Medicine IV, Fernando Fonseca Hospital, Amadora, Portugal*, ^2^*CEDOC, Nova Medical School, Lisboa, Portugal*

**Introduction:** The presence of autoantibodies (Ab) in systemic sclerosis (SSc) is one of the hallmarks of the disease, being strong predictors of organ complications and of disease outcome. There are a number of Ab highly associated with SSc and with SSc overlap features, which are generaly mutually exclusive. Rarely some patients can be positive for more than 1Ab (1.6-4%). The aim of our study is to describe the frequency of patients presentig more than 1Ab and the clinical characteristics associated with that profile.

**Material and Methods:** Retrospective review of 84 consecutive SSc patients from our cohort (fullfiling 2013 EULAR/ACR classification criteria). Profile of Ab analyzed: anticentromere antibodies (ACA), anti-Scl70 (ATA), anti-RNA polymerase III (ARA), anti-U1RNP, anti-pm-scl, anti-Th/To, anti-Ku and anti-U3RNP. Indirect immunofluorescence was performed as screening method for the detection of antinuclear antibodies on HEp-2 cells, followed by immunoblotting. 2 subgroups were studied: patients positive for more than 1Ab (group 1) and patients positive for only 1Ab (group 2). Demographic variables analyzed: age, gender, ethnicity and time since disease-onset. Clinical characteristics analyzed: cutaneous subtype, frequency of Raynaud’s phenomenon, digital ulcers, calcinosis, arthritis, myositis, gastrointestinal and cardiac involvement, interstitial lung disease (ILD), scleroderma renal crisis, pulmonary hypertension and mortality. Comparison of clinical characteristics was made between the 2 groups for each Ab identified.

**Results:** 10 patients (11.9%) were positive for more than 1Ab: ACA+ARA (n=4), ACA+anti-U1RNP (n=1), ACA+anti-pm-scl (n=1), ATA+anti-Ku (n=1), ATA+ ARA+anti-pm-scl (n=1), ARA+anti-Th/To+anti-U1RNP (n=1), anti-U1RNP+anti-Ku (n=1). Demographic characteristics were not different between the two groups. Comparison of clinical characteristics didn’t show any difference for each Ab studied. In group 1, the pattern and frequency of organ involvement was similar to the that described in the literature, regardless of the Ab combination: patients ATA+ had more diffuse cutaneous SSc and ILD, and patients ACA+ had a limited cutaneous SSc and less ILD; patients with anti-U1RNP and anti-pm-scl had more overlap features with muscle involvement and arthritis.

**Conclusions:** In our cohort the frequency of patients with coexisting Ab is higher than that reported in the literature. The clinical profile of this subgroup of patients does not seem to behave differently from the expected for each isolated Ab.

## P.054

## THE AP1-TRANSCRIPTION FACTOR CJUN AND THE HEDGEHOG MEDIATOR GLI2 COOPERATE TO AMPLIFY FIBROBLAST ACTIVATION AND TISSUE FIBROSIS

C. Bergmann, B. Merlevede, L. Hallenberger, C. Dees, C.-W. Chen, T. Trinh-Minh, A. Bozec, G. Schett, J. Distler

*Department of Internal Medicine 3, Rheumatology and Immunology, Friedrich Alexander University Erlangen Nürnberg (FAU), Erlangen, Germany*

**Introduction:** Pathologic activation of fibroblasts is a central feature of fibrotic disorders such as Systemic Sclerosis (SSc). Dysregulation of TGFβ- and Hedgehog signaling is critical for the persistent activation of fibroblasts in SSc. However, the consequences of their concomitant upregulation is unknown and cross-talk between individual mediators of TGFβ and Hedgehog signaling in fibrotic diseases is poorly characterized. Mutual activation and amplification of both pathways might be central for the persistent activation of fibroblasts. Thus, the identification of feedback loops between both pathways might offer a way to recognize novel anti-fibrotic therapies.

**Material and Methods:** Mutual interaction of cJUN and GLI2 was analyzed by confocal microscopy and by Co-IP. cJUN/AP1 signaling and GLI2 were inhibited using T5224 and GANT61. Hedgehog signaling was activated in mice by fibroblast-specific overexpression of constitutively active Smoothend (SmoACT mice).

**Results:** Expression profiling of all AP1 family members revealed most pronounced differences for cJUN in SmoACT mice. The expression of cJUN colocalized with the hedgehog transcription factor GLI2. Overexpression and colocalization of cJUN and GLI2 were also observed in fibroblasts in the skin of SSc patients. The number of GLI2- and cJUN double-positive fibroblasts was particularly high in involved SSc skin. We demonstrated by co-immunoprecipitation that GLI2 and cJUN interact in the nucleus and that TGFβ stimulates their interaction. The central role of the crosstalk between cJUN and GLI2 for tissue fibrosis was further highlighted by the finding that hedgehog-induced fibrosis was strongly reduced by AP1 inhibition. SmoACT mice developed extensive skin fibrosis. Treatment with the AP1 inhibitor T5224 strongly ameliorated hedgehog-induced fibrosis in SmoACT mice. Moreover, combined inhibition of AP1- and Hedgehog signaling exerted additive anti-fibrotic effects in a mouse model of TGFβ driven fibrosis (TBRact mice).

**Conclusions:** We demonstrate that the concomitant activation of AP1- and Hedgehog signaling amplifies fibroblast activation and tissue fibrosis. These profibrotic effects are mediated by direct interaction of GLI2 and cJUN in the nucleus. These findings may open venues for combined inhibition of AP1- and hedgehog signaling for the treatment of fibrosis.

## P.055

## CLINICAL PREVALENCE OF ANTI-BICD2 ANTIBODIES USING A NOVEL MULTI-ANALYTE ASSAY FOR THE DIAGNOSIS OF SYSTEMIC SCLEROSIS IN A SPANISH COHORT

E. Ruiz-Ortiz^1^, S. Prieto^2^, F. Hernández-González^3^, A. Pérez-Isidro^1^, M. Mahler^4^, S. Casas^4^, C. Bentow^4^, A. Seaman^4^, K. Norvell^4^, M. Tiongson^4^, M.A. Aure^4^, G. Kim^4^, R. Cervera^2^, O. Viñas^2^, G. Espinosa^2^

^1^*Immunology Department, Centre Diagnòstic Biomèdic CDB, Hospital Clínic Barcelona, Barcelona, Spain*, ^2^*Autoimmune Diseases Department, Hospital Clínic Barcelona, Barcelona, Spain*, ^3^*Pneumology Department, Respiratory Institute, Hospital Clínic, IDIBAPS, Universitat de Barcelona, Barcelona, Spain*, ^4^*Research and Development, Inova Diagnostics, San Diego, USA*

**Introduction:** Anti-nuclear antibodies (ANA), which are present in approximately 90% of systemic sclerosis (SSc) patient’s sera, play an important role in establishing the diagnosis of disease. In the majority of ANA positive SSc patients, SSc-specific and SSc-associated antibodies (Abs) can be detected, including those directed against centromere (ACA), topoisomerase I (ATA), RNA Pol III, PM/Scl, Fibrillarin, Ro52/TRIM21, RNP, and Th/To. Recently, a novel Ab has been described in SSc patients targeting Cytoskeleton-Like Bicaudal D Protein Homolog 2 (BICD2). Abs directed toward the human BICD2 (anti-BICD2) can be found in about 10-25% of SSc sera, mostly in association with ACA or ATA, but also in isolation. The aim of this study was to analyze the prevalence and titers of anti-BICD2 Abs using a particle-based multi-analyte technology (PMAT) system in a Spanish cohort of SSc patients.

**Material and Methods:** In this study, 239 patient samples collected from Hospital Clínic Barcelona were tested using a novel fully automated PMAT system (research use only, Inova Diagnostics) for detecting Abs to BlCD2 along with criteria SSc biomarkers (ACA, ATA, RNA Pol III). The patient population included 133 SSc samples, together with other non-SSc groups (n=16 autoimmune liver disease (ALD), n=38 idiopathic inflammatory myopathy (IIM), n=28 samples from pneumology department consisting of idiopathic pulmonary fibrosis (IPF, n=14)/interstitial pneumonia with autoimmune features (IPAF, n=14), n=5 other connective tissue diseases (CTD), n=6 overlap syndrome, n=13 suspected/early CTD).

**Results:** The sensitivity of BICD2 in SSc was 10.5% (95% CI 6.4-16.9%, 14/133) while only one positive was found in the other conditions (IPAF/IPF group 3.6%, 1/28). Anti-BICD2 Ab levels were detected significantly higher in SSc patients versus all other patient groups (p<0.0001). The prevalence of ACA, ATA, and RNA Pol III in SSc was 65.4% (87/133), 22.6% (30/133), and 5.3% (7/133), respectively, which is in line with published literature. In the SSc group, 85.7% (12/14) of anti-BICD2 positives were positive for ACA indicating a strong overlap. Interestingly, when analyzing antibody levels among the patient groups, anti-BICD2 had significantly higher antibody levels in the IPAF/IPF group versus all other non-SSc patients (p<0.0001).

**Conclusions:** Our data confirm the presence of anti-BICD2 Ab in SSc patients that may help to differentiate SSc from other systemic autoimmune diseases. The new BICD2 assay measured on the PMAT system shows good clinical performance for SSc in this cohort. Further studies are warranted to investigate clinical associations in the Spanish SSc populations.

## P.056

## THE HMGB1/AGE-RAGE AXIS IN PATIENTS WITH SYSTEMIC SCLEROSIS: A POTENTIAL ROLE IN ITS VASCULOPATHY?

I.M. Atzeni^1^, A. Eman Abdulle^1^, A. van Roon^1^, A.J. Smit^1^, G.F.H. Diercks^2^, H. van Goor^2^, J. Westra^3^, D.J. Mulder^1^

^1^*Department of Internal Medicine, Division of Vascular Medicine, University of Groningen, University Medical Center, Groningen, The Netherlands*, ^2^*Department of Pathology and Medical Biology, University of Groningen, University Medical Center, Groningen, The Netherlands*, ^3^*Department of Rheumatology and Clinical Immunology, University of Groningen, University Medical Center, Groningen, The Netherlands*

**Introduction:** Systemic sclerosis (SSc) is a progressive fibro-inflammatory autoimmune disease of which the pathogenetic pathways are incompletely understood. Advanced glycation endproducts (AGEs) are oxidative stress derived compounds with potential proinflammatory effects. Their exact role in fibrosis remains unknown. The receptor for AGEs is RAGE, which is also the receptor for high mobility group box 1 (HMGB1), a nuclear protein, which is proinflammatory when released from activated or apoptotic cells. We hypothesize that AGEs and HMGB1 may promote inflammation, oxidative stress and profibrotic processes, presumably mediated by RAGE. The aim is to study the role of the HMGB1/AGE-RAGE axis in the pathogenesis of SSc.

**Material and Methods:** For this study, we included 20 SSc patients [median 51 years (IQR 44-58)] and 20 age- and sex-matched healthy controls [(HC) 52 years (45-62)]. Sera were obtained to determine CRP, ESR, HMGB1, free thiols (markers of oxidative stress) and soluble RAGE (sRAGE) levels. AGE accumulation in skin was assessed as skin autofluorescence (SAF) by the AGE Reader. In vitro experiments were performed on healthy human dermal fibroblasts, which were stimulated by HMGB1, AGE-BSA or TGF-β1 as control. Inflammatory and profibrotic markers were measured by ELISA and rt-PCR. Differentiation to myofibroblasts was assessed by staining of α-smooth muscle actin (α-SMA).

**Results:** In SSc patients levels of CRP, ESR, HMGB1, sRAGE and SAF were all significantly increased and free thiols were decreased, compared to HC. In vitro stimulation of fibroblasts with AGE-BSA and HMGB1 resulted in increased mRNA expression of IL-6 and IFI44L (Interferon type 1 induced gene) and a small increase in connective tissue growth factor mRNA expression. Stimulation with AGE-BSA and HMGB1 also induced myofibroblast differentiation and formation of SMA fibers in normal dermal fibroblasts (Figure 1).

**Conclusions:** This translational study confirms that patients with SSc are at increased risk for systemic oxidative stress and inflammation, accompanied by enhanced accumulation of AGEs. RAGE ligands such as AGE-BSA and HMGB1 can induce differentiation to myofibroblasts, possibly by induction of inflammation or interferon upregulation, being a potential new target for treatment. Further studies investigating these pathways are ongoing.

**Figure 1.**
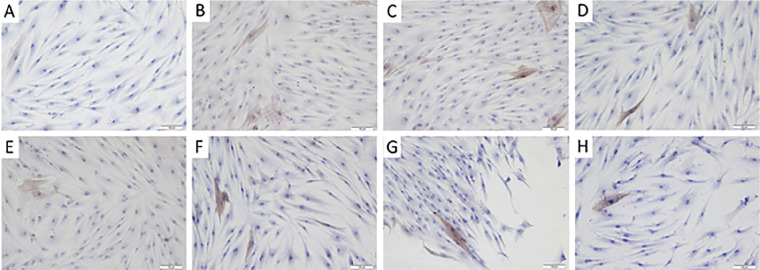
Smooth muscle actin (α-SMA) expression by immunohistochemistry in stimulated and unstimulated human skin fibroblasts. Fibroblasts were treated with 0 (A), 1 (B), 10 (C) or 50 (D) ng/ml TGF-β1, 10 (E) or 100 (F) µg/ml AGE-BSA or 1 (G) or 10 (H) µg/ml HMGB1. All cells were treated for 24 hours. Detection was performed with 2 µg/ml anti α-SMA. Bar 100 µm.

## P.057

## ABERRANT EXPRESSION LEVELS OF SOLUBLE CO-INHIBITORY RECEPTORS AS MARKERS OF T CELL EXHAUSTION AND DISEASE ACTIVITY IN SYSTEMIC SCLEROSIS

M. Aspari^1^, V. Ong^2^, B. Deleuran^1^, D. Abraham^2^

^1^*Aarhus University-Department of Biomedicine, Aarhus, Denmark*, ^2^*Centre for Rheumatology and Connective Tissue Diseases-University College London, London, United Kingdom*

**Introduction:** Recent studies suggest dysregulation in T cell activation in systemic sclerosis (SSc). Co-inhibitory-receptors (Co-IRs) such as TIM-3, PD-1 and LAG-3 play a crucial role in controlling inappropriate T cell activation and in maintaining immune homeostasis. Engagement of these receptors by their ligands limits cytokine production in response to TCR or activating NK receptor stimulation and hence limiting tissue damage from excessive immune activation. However, chronically increased expression of multiple Co-IRs is a hallmark of immune exhaustion. We evaluated the role of these soluble Co-IRs in diffuse SSc (dcSSc) and their impact on disease outcomes.

**Material and Methods:** sPD-1, sLAG-3 and sTIM-3 were measured in sera from 35 patients with dcSSc and 26 healthy controls. All patients had disease duration of less than 5 years. Modified Rodnan skin sore (mRss) varied between 8-39, mean 22. These results were correlated with clinical parameters and possible effect of disease modifying agents (DMARDs: methotrexate[MTX], mycophenolate mofetil [MMF] and prednisolone).

**Results:** The mean of sPD-1 level was increased (136pg/ml) among dcSSc patients in comparison to healthy controls. Comparison of sPD-1 levels in patients on DMARDs with those without treatment demonstrated significant effect of immunosuppressive therapies, with mean sPD-1 95.1 pg/ml among patients on DMARD compared to 216.7 pg/ml among those on no treatment (p=0.0178). sPD-1 demonstrated association with ESR (p=0.034), but not CRP. There was association between sPD-1 and mRss (p=0.04) and FVC (p=0.04).

Mean sLAG-3 levels were significantly lower among dcSSc patients (394.6 pg/ml) vs healthy controls (740.8 pg/ml, p=0.0001). sLAG-3 was inversely associated with disease duration (p=0.04). There was a trend for association between sLAG-3 and mRss, with higher levels of sLAG-3 seen in patients with higher skin score (p=0.06),and between sLAG-3 levels and presence of tendon friction rubs (TFR) (mean sLAG-3 366.3 ng/ml among patient without TFR and 531.5 ng/ml among those with TFR, p=0.08). There was highly significant difference in the sTIM-3 levels between healthy controls (mean 4721.9 ng/ml) and dcSSc patients (8728.0 ng/ml, p<0.0001). There was a trend for association between anti-Scl70 (ATA) positivity and sTIM-3 levels (p=0.0944). Hb levels showed significant association with sTIM-3, with higher Hb levels associated with lower sTIM-3 levels (p=0.02).

**Conclusions:** Soluble co-inhibitors are differentially expressed in early dcSSc and correlate with key clinical features. Our experiments show that the Co-IRs are selectively affected by certain immunosuppressive therapies, which may reflect pathway dysregulation and act as biomarkers for evaluation of disease severity and activity.

**Figure:**
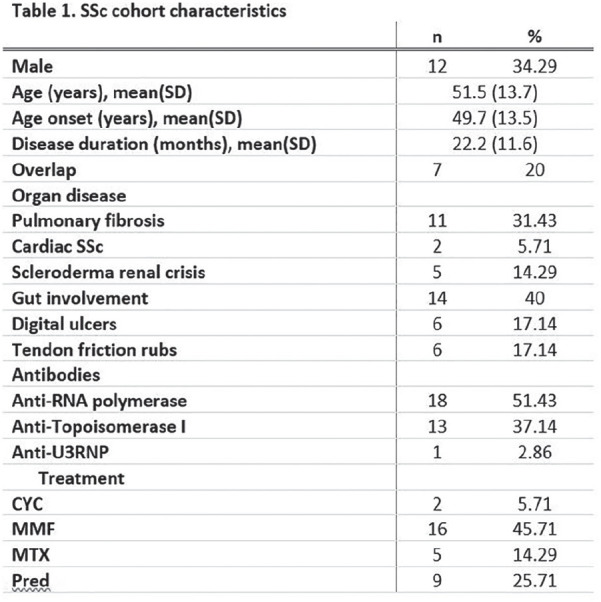


## P.058

## FEATURES OF THE DEBUT OF SYSTEMIC SCLERODERMA ARE ABLE TO DETERMINE THE DISEASE PATTERN

A. Aleksandrov^1,2^, L. Shilova^1^, V. Aleksandrov^1,2^

^1^*Volgograd State Medical University, Department of Hospital Therapy, Volgograd, Russia*, ^2^*Research Institute for clinical and experimental rheumatology named after A.B. Zborovsky, Volgograd, Russia*

**Introduction:** The importance of early detection of systemic scleroderma (SSc) is due to the complexity of the diagnosis at the early stages of the disease.

Objective: to study the effect of various options for the onset of SSc on the dynamics of the clinical picture of the disease, taking into account the clinical, laboratory and instrumental features of the early SSc.

**Material and Methods:** The study included 83 patients with a reliable diagnosis of SSc (97.6% of women, mean age 50.3±11.9), 30 patients with localized scleroderma, 30 patients with primary Raynaud’s syndrome (PRS). A complete clinical, laboratory and instrumental examination was carried out, including capillaroscopy of the vessels of the nail bed. A limited form of the disease was determined in 67.5% of patients with SSc, a diffuse form in 32.5%, and in 11 cases an overlap syndrome was noted. Early scleroderma was diagnosed in 33.7% of patients.

**Results:** The study of the SSc debut showed that in 34.9% of cases the disease started with skin syndrome, in 48.2% with Raynaud’s syndrome, in 16.9% with articular syndrome. The average diagnosis term for SSc was 3.1±1.01 years; later diagnosis was observed in patients with Raynaud’s syndrome and plaque form of skin manifestations.

The number of capillaries of the nail bed in the group of patients with early SSc averaged 6.5±2.0, avascular zones - 2.0±1.17, bush-shaped capillaries - 1.53±1.23, dilated capillaries - 1.9±1.54, extravasates - 0.12±0.33. When comparing these indicators with early SSc, differences were obtained with patients with localized scleroderma (p<0.001 for all indicators); with patients with PRS, significant differences were revealed in the number of capillaries (M-W U-test=35.5, p=0.0003), the presence of avascular zones (M-W U-test=35.0, p=0.0003) and bush-shaped deformity (MW U-test=49.5, p=0.001).

Changes in laboratory parameters (increase in ESR in 57.1% of cases, CRP - in 21.4%, decrease in hemoglobin - in 25%) in patients with early SSc indicated the inflammatory processes in the early stages of the disease. Immunological changes were characterized by an increase in the titer of antinuclear antibodies in 89.3% of patients; these changes were detected in the first 3 years of the disease in 90% of cases.

**Conclusions:** The early form of SSc has its own clinical, laboratory and instrumental features that can determine different dynamics of the clinical picture of the disease. Careful attention is required to all patients with Raynaud’s syndrome and localized forms of skin manifestations, as well as screening for the presence of antinuclear antibodies.

## P.059

## A NOVEL HUMANIZED MOUSE MODEL OF SKIN FIBROSIS

G. Moroncini^1^, C. Paolini^1^, F. Orlando^2^, A. Grieco^1^, S. Agarbati^1^, C. Tonnini^1^, S. Svegliati Baroni^1^, T. Spadoni^1^, A. Funaro^3^, E. Avvedimento^4^, M. Provinciali^2^, A. Gabrielli^1^

^1^*Università Politecnica delle Marche, Dipartimento di Scienze Cliniche e Molecolari, Ancona, Italy*, ^2^*IRCCS - INRCA, Servizio di Allevamento e Sperimentazione Animale, Polo Scientifico Tecnologico, Ancona, Italy*, ^3^*Università di Torino, Dipartimento di Scienze Mediche, Torino, Italy*, ^4^*Università Federico II, Dipartimento di Medicina Molecolare e Biotecnologie Mediche, Napoli, Italy*

**Introduction:** Platelet Derived Growth Factor (PDGF) Receptor alpha (PDGFR alpha) is a target of the autoimmune response in scleroderma (SSc). Both total serum IgG (SSc-IgG) and anti-PDGFR alpha antibodies cloned from memory B cells of SSc patients (SSc-Mabs) demonstrated the ability to increase collagen gene transcription in healthy donor skin fibroblasts and to induce fibrosis ex vivo, in skin grafts in SCID mice. In order to replicate these findings in vivo, we generated human PDGFR alpha-transgenic mice.

**Material and Methods:** Full length human PDGFR alpha cDNA was knocked-in into the ubiquitously expressed Rosa26 locus on mouse chromosome 6. Correctly targeted C57BL/6 ES cell clones were selected for blastocyst microinjection, followed by chimera production. F2 heterozygous C57BL/6-hPDGFR alpha transgenic mice were used to establish the colony. Twelve weeks-old male mice were injected into the back skin at days 0, 3, 6 and 9, either with 0.02 mg/ml of SSc-Mabs (VHPAM-VK16F4 or VHPAM-VK13B8), or with 2 mg/ml of SSc-IgG or IgG purified from serum of healthy donors (HD-IgG). Vehicle only injection control was included. Age- and sex- matched C57BL/6 wild type mice were used as controls. Animals were sacrificed at day 14. Human PDGFR alpha transgene expression and collagen amount were assessed in explanted skin tissue.

**Results:** Transgenic mice were phenotypically normal, fertile, and did not display any apparent pathological features. Human PDGFR alpha mRNA and protein were detectable in the skin of all examined transgenic mice. Intradermal injection of stimulatory human SSc-Mab VHPAM-VK16F4 or SSc-IgG resulted in dermal thickening and increased collagen deposition, whereas non-stimulatory human SSc-Mab VHPAM-VK13B8 or HD-IgG did not induce any significant skin tissue alterations compared to vehicle control. C57BL/6 wild type mice did not show any significant skin tissue changes with any antibodies.

**Conclusions:** We generated a novel humanized mouse model of skin fibrosis based on the concomitant expression of human PDGFR alpha and injection of stimulatory anti-PDGFR alpha antibodies. This model may be useful to identify new therapeutic strategies for SSc and for their preclinical validation.

## P.060

## THE ROLE OF CXCL4, CXCL8 AND GDF-15 IN SYSTEMIC SCLEROSIS

JE. Oller Rodríguez^1^, M. De La Rubia Navarro^1^, E. Grau García^1^, I. Martínez Cordellat^1^, FM. Ortiz Sanjuán^1^, E. Vicens Bernabéu^1^, R. Negueroles Albuixech^1^, I. Chalmeta Verdejo^1^, C. Alcañiz Escandell^1^, C. Perales Pávez^1^, C. González Mazarío^1^, JJ. Fragío Gil^1^, I. Cánovas Olmos^1^, C. Nájera Herranz^1^, L. González Puig^1^, J. Ivorra Cortés^1^, JA. Román Ivorra^1^

*Rheumatology Department. HUP La Fe. Valencia. Spain*

**Introduction:** Systemic Sclerosis (SSc) is an autoinmune disease that can affect several organs and its mortality is fundamentally related to its pulmonary involvement.

It is mandatory to seek for biomarkers that help us with early diagnosis and that are also useful for predicting organic involvement, so that we can adjust the diagnostic and therapeutic approach. Our aim was to check if the presence of CXCL4, CXCL8 and GDF-15 is greater in the disease than in healthy population, ald also their involvement in organic damage.

**Material and Methods:** Observational and cross-sectional study, with a prospectively performed protocol, of patients diagnosed of SSc according to ACR/EULAR 2013 criteria. Demographic, clinical, analytical, activity (EUSTAR index), severity (Medsger scale and modified Rodnan index), health perception (SF36) and disability (HAQ and Cochin test) variables were collected. Moreover, Videocapillaroscopy (VCL) and Respiratory Function Test were made, as well TCMD and echocardiography in order to describe pulmonary features. Serum levels of CXCL4, CXCL8 and GDF-15 were measured in SSc patients and in healthy controls.

**Results:** A total of 42 patients were included (95.4% women), with an average age of 59.2 years. The median of years from diagnosis was 4, and 6 from the first not Raynaud symptom. Out of them, 20 were limited, 20 patients diffuse form; and 2 patients were sine scleroderma. We also included 42 healthy controls.

We found significantly higher levels of GDF-15 in SSc patients, with no significant differences in CXCL4 and CXCL8 levels between SSc patients and healthy controls.

The presence of GDF-15 was associated with diffuse SSc, pulmonary arterial hypertension (PAH), interstitial lung disease (ILD), a decrease in forced vital capacity (FVC), high serum titles of antiScl70, higher disease activity (EUSTAR index) as dilations and decreased capillary density in capillaroscopy.

Moreover, we only found association between CXCL4 levels and skin involvement showed as a higher Rodnan score punctuation, not being associated to lung involvement or another features (spirometric or analytical changes, capyllaroscopy and functional tests).

Attending to CXCL8, it was associated to consumption of the C4 fraction of complement and the presence of tortuosities in capillaroscopy, with no other significant findings.

**Conclusion:** We can conclude that presence of GDF-15 is associated with diffuse SSc, lung impairment, disease activity and changes capillaroscopy. Moreover, CXCL4 was only associated with skin impairment, while CXCL8 was not related to any organic damage in our patients.

## 2. Pulmonary including Interstitial Lung Disease and Pulmonary

## P.061

## SYSTEMIC SCLEROSIS AUTO-ANTIBODY PROFILES PREDICT INTERSTITIAL LUNG DISEASE ONSET BUT NOT PROGRESSION

B. Zheng^1^, M. Wang^1^, M.J. Fritzler^2^, M.Y. Choi^2^, M. Baron^1^, M. Hudson^1^, On behalf of the CSRG

^1^*Jewish General Hospital, McGill University, Montreal, Canada*, ^2^*University of Calgary, Calgary, Canada*

**Introduction:** Interstitial lung disease (ILD) is a leading cause of mortality in systemic sclerosis (SSc) where it is associated with the presence of anti-topoisomerase I autoantibody (aAb) (ATA) while anti-centromere aAb (ACA) may be protective. The association with other aAbs is unclear and it is unknown whether aAb profiles can predict the rate of ILD progression. Our goal was to examine the incidence of SSc-ILD and its subsequent progression between different aAb profiles.

**Material and Methods:** SSc subjects without pre-existing ILD in a multi-center cohort from 2004-2018 were included. ILD was defined by high-resolution computed tomography (HRCT) findings, or if not was available, either a chest x-ray showed fibrosis, and/or “velcro like crackles” was present on exam. Clinically meaningful ILD progression was defined as a decline of: FVC >=10%, or FVC >=5% and DLCO >=15%. Subjects were grouped based on single specificity aAbs: ATA, ACA, anti-RNA polymerase III (ARA), anti-PM/Scl, anti-Ku, anti-Th/To (hPOP1), anti-fibrillarin (AFA), anti-U1RNP (U1RNP) or no detectable aAbs. The time to ILD onset and the time to meaningful progression and mortality after incident ILD were compared between groups using Kaplan Meier and multivariate Cox analyses adjusted for age, gender, diffuse disease, physician global assessment of severity, immunosuppression exposure at baseline and anti-Ro52/TRIM21 aAb positivity. ATA served as the reference for all comparisons.

**Results:** Of 931 patients, 190 (20%) developed ILD: 60/429 (14%) ACA, 31/94 (33%) ATA, 32/114 (28%) ARA, 6/24 (25%) anti-PM/Scl, 0/2 anti-Ku, 2/5 (40%) anti-Th/To, 4/5 (80%) AFA, 6/26 (23%) U1RNP, and 55/258 (21%) negative aAb. Subjects with ATA developed ILD at shorter disease durations than ACA, anti-PM/Scl, U1RNP, and negative aAb groups (log rank p<0.01, p=0.02, p=0.01, p<0.01 respectively). The AFA group had even earlier onset (p<0.01). In multivariate analyses, ACA and negative aAb groups were less likely (HR (95% CI) 0.36 (0.22, 0.57) and 0.53 (0.34, 0.84) respectively), whereas AFA was more likely (HR (95% CI) 4.12 (1.22, 13.86)) to develop ILD compared to ATA. Despite this, there were no differences in the time to clinically meaningful ILD progression or survival in patients with incident ILD.

**Conclusions:** Although we had few AFA patients, this aAb conferred a higher risk of developing ILD compared to patients with ATA. ACA, anti-PM/Scl, U1RNP and the absence of SSc specific aAbs were associated with a lower risk. Nevertheless, ILD developed in all aAb sub-groups and, once present, the aAb profile was not associated with ILD progression or subsequent mortality.

## P.062

## TREATMENT WITH MYCOPHENOLATE MOFETIL AND CYCLOPHOSPHAMIDE LEADS TO CLINICALLY MEANINGFUL IMPROVEMENTS IN PATIENT-REPORTED OUTCOMES IN PATIENTS WITH SYSTEMIC SCLEROSIS-RELATED INTERSTITIAL LUNG DISEASE

E. Volkmann^1^, D. Tashkin^1^, H. Leclair^1^, M. Roth^1^, P. Clements^1^, G. Kim^1^, J. Goldin^1^, D. Furst^1^, D. Khanna^2^

^1^*University of California, Los Angeles, USA*, ^2^*University of Michigan, Ann Arbor, USA*

**Introduction:** Patient reported outcomes (PROs) are increasingly utilized in clinical trials to assess how a patient feels and functions in response to an intervention. In systemic sclerosis-related interstitial lung disease (SSc-ILD), measures of dyspnea, cough and disability can provide important and comprehensive insights into therapeutic effectiveness. Furthermore, these outcomes may have more meaning to patients than outcomes, such as the forced vital capacity (FVC). The objective for this study was to determine whether cyclophosphamide (CYC) and mycophenolate mofetil (MMF) improve PROs in SSc-ILD patients who participated in the Scleroderma Lung Study (SLS) II.

**Material and Methods:** SLS II was a randomized controlled trial comparing MMF for 2 years versus oral CYC for 1 year followed by 1 year of placebo. The following PROs were assessed every 3-6 months, and each one aimed to address a unique aspect of the SSc-ILD disease experience: Short Form 36 (SF-36), Health Assessment Questionnaire (HAQ) disability index (DI), Baseline and Transitional Dyspnea Index (BDI, TDI), the Leicester Cough Questionnaire (LCQ), St. George’s Respiratory Questionnaire (SGRQ). Joint models were created to evaluate the course of PROs over 2 years. The difference in PRO scores from baseline to 24 months were measured, and the percentage of patients meeting the minimum clinically important difference (MCID) were calculated. Correlations between PRO and surrogate measures of SSc-ILD disease severity were also examined.

**Results:** Treatment with CYC and MMF led to improvements in several PRO outcomes, with no between-treatment arm differences. Scores for the TDI (Figure 1a) and SGRQ (Figure 1b) improved significantly over 2 years, and 29%/24% and 28%/25% of participants in the CYC/MMF groups met or exceeded the MCID estimates for TDI and SGRQ, respectively. The course of the HAQ-DI also improved over the course of the study, although the change was not statistically significant; 17% and 14% of participants randomized to CYC and MMF, respectively met or exceeded the MCID for this outcome at 24 months. At baseline, the FVC%-predicted did not correlate with the BDI or SGRQ. However, improvements in the FVC%-predicted were weakly associated with improvements in the TDI and SGRQ scores.

**Conclusions:** Treatment with CYC and MMF improved overall health-related quality of life in patients with SSc-ILD, including breathlessness. The relationship between individual PRO measures and the FVC was relatively weak, suggesting that PROs provide complementary information about treatment efficacy not captured by changes in the FVC alone in this patient population.

**Figure:**
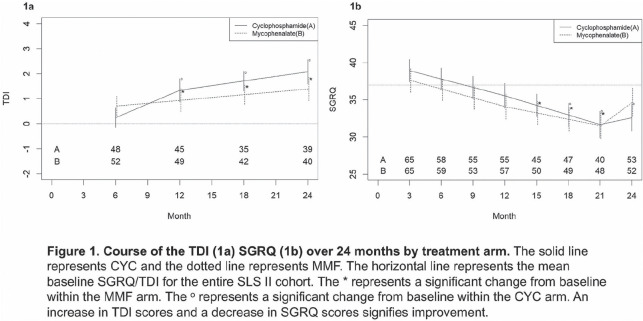


## P.063

## THE MUC5B PROMOTER VARIANT DOES NOT PREDICT RESPONSE TO IMMUNOSUPPRESSION IN PATIENTS WITH SYSTEMIC SCLEROSIS-RELATED INTERSTITIAL LUNG DISEASE

E. Volkmann^1^, D. Tashkin^1^, N. Li^1^, J. Charles^2^, M. Mayes^2^, M. Roth^1^, G. Kim^1^, J. Goldin^1^, P. Clements^1^, D. Furst^1^, D. Khanna^3^, L. Pourzand^1^, R. Elashoff^1^, S. Assassi^2^

^1^*University of California, Los Angeles, USA*, ^2^*University of Texas, Houston, USA*, ^3^*University of Michigan, Ann Arbor, USA*

**Introduction:** The natural history of interstitial lung disease (ILD) in patients with systemic sclerosis (SSc) is highly variable. While certain factors may increase the risk of SSc-ILD progression, no factors consistently predict response to therapy in these patients. Prior studies have demonstrated that the presence of a specific genetic polymorphism (MUC5B) is associated with ILD development in rheumatoid arthritis and idiopathic pulmonary fibrosis. The present study investigated whether this gain-of-function promoter polymorphism exists among patients with SSc-ILD and whether its presence predicts response to immunosuppression with cyclophosphamide (CYC) and mycophenolate (MMF).

**Material and Methods:** Patients who participated in Scleroderma Lung Study (SLS) II were included in this study (N=142). SLS II randomized SSc-ILD patients to receive MMF for 2 years or oral CYC for 1 year followed by 1 year of placebo. Genotyping of the MUC5B rs35705950 single nucleotide polymorphism was performed using TaqMan Genotyping Assays in all patients with an available DNA sample. The forced vital capacity (FVC) and the diffusing capacity for carbon monoxide (DLCO) were measured every 3 months. Quantitative Lung fibrosis (QLF) and Quantitative ILD (QILD) scores for whole lung (WL) and zone of maximum involvement (ZM) were measured at baseline and 24 months. Joint models were generated to examine how the presence of this variant affected the course of the FVC and DLCO. Linear regression models were created to investigate the relationship between the presence of this variant and the change in radiographic fibrosis at 24 months.

**Results:** Among 128 participants who were tested for this variant, 23 (18%) possessed at least one copy of the MUC5B rs35705950 minor allele. Patients with at least one copy of this allele were similar to those without the allele with respect to age, sex, %diffuse disease, disease duration, ILD disease severity. Similar to available data from the general population, this variant was rare among African Americans (3.7%) in our cohort. The presence of the MUC5B variant did not affect the course of the FVC (Figure 1), DLCO, nor the change in the QLF-ZM, QLF-WL, QILD-ZM, QILD-WL scores over 24 months in either treatment arm.

**Conclusions:** In the context of a rigorously-conducted clinical trial for SSc-ILD, the presence of MUC5B rs35705950 minor allele did not alter treatment response with MMF or CYC. Future studies are needed to determine whether the presence of this variant affects ILD progression in other SSc cohorts and in patients receiving anti-fibrotic therapy for SSc-ILD.

**Figure:**
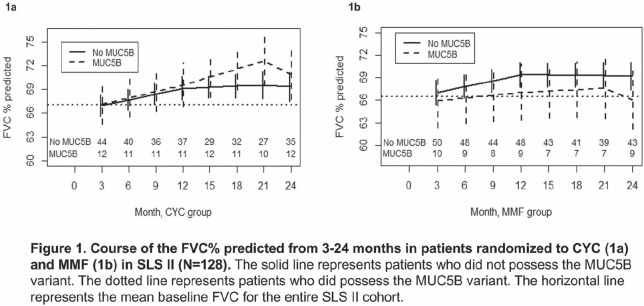


## P.064

## FATAL CONSEQUENCES OF THERAPEUTIC THORACENTESIS IN PATIENTS WITH SYSTEMIC SCLEROSIS

I. Vigdorchick^1^, A. Natour^1^, O. Wand^2^, Y. Levy^3^

^1^*Department of Internal Medicine, Meir Medical Center, Kfar Saba, Israel*, ^2^*Department of Pulmonology, Meir Medical Center, Kfar Saba, Israel*, ^3^*Sackler Faculty of Medicine, Tel-Aviv University, Tel-Aviv, Israel*

**Introduction:** Systemic sclerosis (SSc) is a systemic autoimmune disease, characterized by systemic fibrosis and involvement of visceral organs. Pulmonary complications are common and a leading cause of death. Pleural effusions, however, are rare. Thoracentesis is a common procedure, performed to reveal the cause of pleural effusion or to drain it and relieve dyspnea. Although generally considered a low-risk intervention, complications of thoracentesis can lead to increased morbidity and mortality.

**Material and Methods:** We describe three patients with SSc and symptomatic pleural effusion who required thoracentesis.

**Results:** All patients deteriorated shortly after the procedure and died.

**Conclusions:** We assume that patients with SSc are at high-risk to develop complications after thoracentesis, most likely due to the low compliant lungs and the low elastance of the pleura. In this population, thoracentesis should be done with high caution, while measuring the pleural pressure – invasively, or with noninvasive surrogates. Further studies are required to determine mechanisms of the complication.

## P.065

## QUANTITATIVE CHEST COMPUTED TOMOGRAPHY (QCT) ANALYSIS OF SYSTEMIC SCLEROSIS INTERSTITIAL LUNG DISEASE (SSC-ILD) TREATED WITH RITUXIMAB: PRELIMINARY FINDINGS

F. Cacciapaglia^1^, S. Parisi^2^, F. Montini^1^, V. Fiore^1^, L. Urso^1^, E. Fusaro^2^, A. Ariani^3^, F. Iannone^1^

^1^*Rheumatology Unit, Department of Emergency and Organs Transplantation, University of Bari, Bari, Italy*, ^2^*Rheumatology Unit, University Hospital Città della Salute e della Scienza di Torino, Torino, Italy*, ^3^*Department of Medicine, Unit of Internal Medicine and Rheumatology, AOU di Parma, Parma, Italy*

**Introduction:** Several evidence show that rituximab (RTX) may be effective in interstitial lung disease associated with systemic sclerosis (SSc-ILD) treatment [1, 2]. Quantitative Computed Tomography (QCT) is an automated and operator independent method assessing SSc-ILD severity and extent [3]. The main aim of this study is to evaluate the capacity of QCT method to identify improvement in SSc-ILD patients treated with RTX.

**Material and Methods:** All SSc patients, fulfilling the 2013 ACR/EULAR classification criteria [4], with ILD unresponsive to standard treatment that underwent to at least 2 RTX cycles (1000 mg IV two weeks apart, every 24 weeks) since 2013 in Bari’ Rheumatology Unit, were screened. In order to assess the response, pulmonary function tests (PFTs) and chest high-resolution computed tomography (HRCT) performed at baseline (T0) and after 12 months (T1) treatment, were considered. Two radiologists assessed all HRCTs according to the standard qualitative ILD. QCT analysis of all HRCTs was performed with a free medical image viewer (Horos; https://horosproject.org). Categorical data were assessed using the Fischer exact test; the non-parametric Wilcoxon test for paired samples was used to evaluate differences between T0 and T1. A p-value <0.05 was considered statistically significant.

**Results:** Of 20 SSc-ILD patients that received RTX, 10 (50%) patients (8 females; age 50±8 years; median (IQR) disease duration 5.5 (3.2-6.7) years) with a total of 20 HRCTs were available for QCT analysis. In these patients, forced vital capacity significantly improved (T0: 88 (75.3-104.4)%; T1: 95 (78.2-110.6) % - p=0.04), while the diffusion capacity of the lung for CO change didn’t reach the statistically significance (T0: 71.2 (63.6-81)%; T1: 79 (64,9-82,9)% - p=0.42). According to standard radiologist evaluation, there were no changes in SSc-ILD evolution. The majority of QCT parameters improved between T0 and T1. Particularly one of them, Kurtosis referred to pulmonary parenchyma, statistically increased from 2.3 (1.2-4.3) to 3.3 (1.6-4.1) (p=0.027).

**Conclusions:** Our preliminary data demonstrate no worsening in SSc-ILD treated with RTX. The QCT evaluation of SSc-ILD appears to be able to discriminate minor changes and to better highlight the outcome after a potential effective treatment such as RTX, but it is necessary to widen the sample by more significant data.

## P.066

## LUNG FIBROSIS DOES NOT IMPACT PLATELET COUNT IN SCLERODERMA PATIENTS WITH LUNG INVOLVEMENT

Y. Suliman^1^, S. Kafaja^2^, M. Alemam^3^, D. E Furst^2^

^1^*Rheumatology and Rehabilitation, Assiut university hospital, Assiut, Egypt*, ^2^*Rheumatology Division, Dept of Medicine, UCLA, California, USA*, ^3^*Laboratory Medicine, South Valley University, Qena, USA*

**Introduction:** Lung involvement in SSc is a major cause of morbidity and mortality. HRCT and pulmonary function tests are the useful tools for diagnosis and follow-up of Interstitial lung disease (ILD) in SSc pts. These are expensive and subject to variability. Platelet counts(plt), on the other hand, are feasible tests ordered routinely in SSc patients.

Recent research on Megakaryocyte physiology reported that the lungs have a substantial role in platelet biogenesis and that they are responsible for 50% of total platelet production*. Since lung fibrosis is a common, important feature of SSc, we hypothesized that platelet counts might function as an inexpensive, biomarker to reflect lung fibrosis. We therefore examined correlations of platelet counts with ILD patients (diagnosed by HRCT and pulmonary function tests [PFT])

Objective: 1) To evaluate platelet counts in SSc pts with and without interstitial lung disease, 2) to evaluate differences in platelet count among SSc pts with normal and abnormal PFT (FVC)

**Material and Methods:** SSc patients meeting the ACR/EULAR 2013 SSc criteria were recruited from two SSc centers (University of California, Los Angeles [UCLA], USA and Pacific Arthritis Scleroderma Clinic.) Laboratory and clinical data were obtained from charts for statistical associations.

Statistical analysis included descriptive analysis and Wilcoxon rank correlation analysis as well as regression to account for other potential causes of that affect plt count (ie,anemia, use of corticosteroids, immunosuppressive drugs )

**Results:** In 95 SSc pts, 45(47% )diffuse SSc, mean age55.6( ±13.7) interstitial lung disease (ILD) 54(57%), Immunosuppressive 62(65%) : Corticosteroids: 12(12%); PH: 26(27%)(table 1).

Platelet counts were not significantly lower in SSc patients with ILD compared to non-ILD patients (mean 250 [SD±87] versus 280 [SD ±98) p=0.09). There were also no differences for FVC (> 80%, < 80 %): 270 [ ±92] vs256 [ ±93] p=0.2) or Hgb-corrected-DLCO (>75%, <75 %) : 266 ( 111) vs 255 ( ±99) p=0.7).

Regression models showed that Hgb and use of Immunosuppressive use were the only significant predictors of Plt count (p=0.04 and p=0.005 respectively).

**Conclusions:** Platelet counts are not influenced by pulmonary fibrosis or abnormal pulmonary function (FVC)in this cohort of 95 SSc patients. We speculate that compensatory platelet formation by the bone marrow may confound this correlation.

**Figure:**
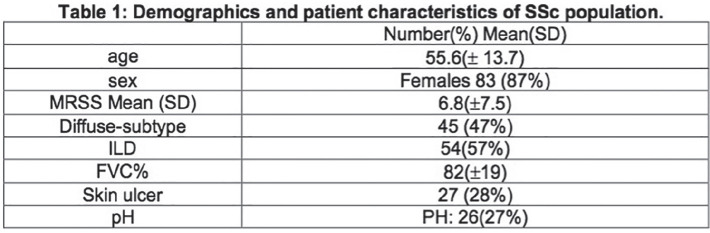


**References**

*E Lefrançais et al., The lung is a site of platelet biogenesis and a reservoir for hematopoietic progenitors Nature. 2017 April 06; 544(7648)

## P.067

## SYSTEMIC LEVELS OF S100A4 CORRELATE WITH DISEASE ACTIVITY, SKIN FIBROSIS AND INTERSTITIAL LUNG DISEASE IN PATIENTS WITH SYSTEMIC SCLEROSIS

H. Storkanova^1,2^, L. Andres Cerezo^1^, S. Oreska^1,2^, M. Spiritovic^3^, B. Hermankova^3^, M. Komarc^4^, K. Pavelka^1,2^, J. Vencovsky^1,2^, J. Distler^5^, R. Becvar^1,2^, L. Senolt^1,2^, M. Tomcik^1,2^

^1^*Institute of Rheumatology, Prague, Czech Republic*, ^2^*Department of Rheumatology, 1st Faculty of Medicine, Charles University, Prague, Czech Republic*, ^3^*Faculty of Physical Education and Sport, Department of Physiotherapy, Charles University, Prague, Czech Republic*, ^4^*Department of Methodology, Faculty of Physical Education and Sport, Charles University, Prague, Czech Republic*, ^5^*Department of Internal Medicine III and Institute for Clinical Immunology, University of Erlangen-Nuremberg, Erlangen, Germany*

**Introduction:** In our previous study we demonstrated that S100A4 is overexpressed in scleroderma (SSc) skin, SSc fibroblasts and preclinical models of SSc in a TGF-b dependent manner. We showed that S100A4 is a new regulator of TGF-b signalling and its inhibition prevents the pro-fibrotic effects of TGF-b. Inactivation of S100A4 prevented dermal fibrosis induced by bleomycin and in Tsk-1 mice.

The aim of this study was to evaluate S100A4 in the peripheral blood of SSc patients and characterize its potential association with SSc-related features.

**Material and Methods:** A total of 33 patients (29 females; mean age 52.8; disease duration 4.2 years; dcSSc/lcSSc = 8/25) who met the 2013 EULAR/ACR classification criteria for SSc and 20 healthy age- and sex-matched individuals were included in this study. Plasma levels of S100A4 were measured using ELISA (CUSABIO, Houston, USA). Data are presented as median (IQR).

**Results:** S100A4 plasma levels were significantly increased in SSc patients compared to healthy controls (78.6(32.3-146.5) vs. 43.4(32.3-53.4)ng/mL,p=0.011). Patients with diffuse cutaneous (dc)SSc had significantly higher levels of S100A4 than patients with limited cutaneous (lc)SSc or healthy controls (168.5(81.5-347.5) vs. 63.4(30.9-130.6),p=0.017 and vs. 43.4(32.3-53.4)ng/mL,p=0.001, respectively). Plasma levels of S100A4 positively correlated with mRSS (r=0.556,p=0.001). Furthermore, S100A4 negatively correlated with forced vital capacity (FVC) and saturation of peripheral oxygen (SpO2) (r=- 0.362,p=0.038;r=-0.414,p=0.029, respectively). S100A4 levels positively correlated with ESSG activity score (r=0.750,p<0.001). However, only correlations between S100A4 and mRSS, and ESSG activity score were approved at corrected level of statistical significance after Bonferroni’s correction (p<0.01). In a prospective analysis of patients (n=24) with progressive SSc-ILD treated with 6 (n=15) or 12 (n=9) monthly i.v. pulses of cyclophosphamide (CPA,10 mg/kg) we observed a significant decrease in plasma S100A4 levels between the baseline samples (month 0) and blood drawn after 6 months of CPA treatment (74.7(42.9–89.2) vs. 59.8(36.5–73.2)ng/mL, p=0.028).Furthermore, baseline S100A4 levels predicted the change (m0-m6) in CRP and ESR levels after 6 months of CPA therapy (r=0.554,p=0.011;r=0.764,p<0.001, respectively).

**Conclusions:** We demonstrate that plasma S100A4 levels are significantly increased in SSc patients compared with healthy controls. Increased S100A4 is associated with dcSSc subset, skin involvement, deteriorated parameters of interstitial lung disease and higher disease activity, and in patients with progressive SSc-ILD declines after 6 months of cyclophosphamide therapy and predicts the systemic inflammatory response. These data further support our previous findings on the role of S100A4 as a regulator of TGF-b induced fibrosis in SSc.

**Acknowledgements:** Supported by MHCR023728, SVV–260373.

## P.068

## EXTRACELLULAR HSP90 AS A POTENTIAL BIOMARKER OF SKIN AND LUNG INVOLVEMENT IN SYSTEMIC SCLEROSIS

H. Storkanova^1,2^, S. Oreska^1,2^, M. Spiritovic^3^, B. Hermankova^3^, K. Bubova^1,2^, M. Komarc^4^, K. Pavelka^1,2^, J. Vencovsky^1,2^, J. Distler^5^, L. Senolt^1,2^, R. Becvar^1,2^, M. Tomcik^1,2^

^1^*Institute of Rheumatology, Prague, Czech Republic*, ^2^*Department of Rheumatology, 1st Faculty of Medicine, Charles University, Prague, Czech Republic*, ^3^*Faculty of Physical Education and Sport, Department of Physiotherapy, Charles University, Prague, Czech Republic*, ^4^*Department of Methodology, Faculty of Physical Education and Sport, Charles University, Prague, Czech Republic*, ^5^*Department of Internal Medicine III and Institute for Clinical Immunology, University of Erlangen-Nuremberg, Erlangen, Germany*

**Introduction:** Our previous study demonstrated that Hsp90 is overexpressed in the skin of patients with SSc, in cultured SSc fibroblasts and preclinical models of SSc in a TGF-b dependent manner. The aim of this study was to evaluate plasma Hsp90 of SSc patients and characterize its potential association with skin changes and SSc-related features.

**Material and Methods:** A total of 92 patients (79 females; mean age 52.7; disease duration 6.0 years; diffuse cutaneous (dc)SSc / limited cutaneous (lc)SSc=38/54) and 92 age- and sex- matched healthy individuals were included. Plasma Hsp90 levels were measured by ELISA (eBioscience, Vienna, Austria). Data are presented as median (IQR).

**Results:** Plasma Hsp90 levels were increased in SSc patients compared to healthy controls [12.5(9.6–17.9) vs. 9.8(7.7–12.4)ng/mL, p=0.0001]. Hsp90 levels in all patients positively correlated with CRP (r=0.271, p=0.015). Furthermore, Hsp90 concentrations were negatively associated with functional parameters of interstitial lung disease (ILD): FVC (r=-0.291, p=0.013), FEV1 (r=-0.248, p=0.036), DLCO (r=-0.290, p=0.012) and SpO2 (r=-0.317, p=0.038). When adjusted for CRP, these correlations still remained significant in multivariate analysis. Higher Hsp90 concentrations were associated with presence of synovitis [17.6(15.4–24.0) vs. 12.2(9.3–17.3), p=0.039]. In addition, only in patients with dcSSc, Hsp90 levels positively correlated with the mRSS (r=0.437, p=0.006). In a prospective analysis of patients with progressive SSc-ILD treated with 6 (n=21 patients) or 12 (n=14 patients) monthly i.v. pulses of cyclophosphamide (CPA, 10 mg/kg) we did not observe any significant differences between the baseline sample (month 0) and blood drawn after 1, 6 and 12 months of CPA therapy. Nevertheless, baseline Hsp90 was able to predict long-term response after one year of CPA treatment (DLCOm12-m0: r=-0.494, p=0.037). Moreover, change in Hsp90 after one month of CPA treatment (Hsp90m1-m0) was able to predict the short-term inflammatory response (CRPm3-m0: r=-0.495, p=0.019; ESRm3-m0: r=-0.496, p=0.031). Concentrations of extracellular Hsp90 were not significantly affected by other main clinical parameters of SSc.

**Conclusions:** We demonstrated higher plasma levels of Hsp90 in SSc patients compared to healthy controls. Concentrations of extracellular Hsp90 increase with higher inflammatory activity, with deteriorated lung functions in ILD and also with the extent and severity of the skin involvement in patients with diffuse cutaneous SSc. These data further highlight the role of Hsp90 as a significant regulator of fibroblast activation and tissue fibrosis in SSc. Moreover, Hsp90 could become a predictor of treatment response in SSc-ILD.

**Acknowledgement:** Supported by AZV-16-33542A, MHCR023728 and SVV–260373.

## P.069

## PULMONARY HYPERTENSION IN PATIENTS WITH SYSTEMIC SCLEROSIS – AN AUDIT OF SCREENING PRACTICES AND COST OVER 10 YEARS

L. Spray, J. Vila, B. Griffiths

*Rheumatology Department, Freeman Hospital, Newcastle upon Tyne, United Kingdom*

**Introduction:** Patients with systemic sclerosis (SSc) are at risk of developing pulmonary arterial hypertension (PAH), a subtype of pulmonary hypertension (PH) which is not due to left-heart disease, chronic hypoxia or pulmonary arterial thrombus. The European Society of Cardiology recommends annual screening of patients with SSc due to the insidious presentation of PAH, poor outcomes, and the availability of effective treatments. Transthoracic echocardiography is the standard screening investigation, but NT pro-BNP, a biochemical marker of ventricular stretch, may be a cost-effective initial test with echocardiography reserved for patients with new or worsening symptoms or increasing NT pro-BNP. Our centre introduced NT pro-BNP as an adjunct to echocardiography in 2014.

**Material and Methods:** We audited our SSc-PAH screening programme from 2009-2018 against the standard that every SSc patient should be screened annually with echocardiography or NT pro-BNP. Patients seen by the regional PH service prior to the first rheumatology clinic were excluded. We used our centre’s database of SSc patients and electronic patient records to determine if a patient had undergone PH screening. We calculated cost estimates from our hospital’s biochemistry and echocardiography departments.

**Results:** From 2009 to 2018, the number of SSc patients requiring annual screening rose from 81 to 215. In 2009, 65% of patients were screened – all with echocardiography. In 2018, 88% of patients were screened – 25% had an echocardiogram and 83% had an NT pro-BNP. 63% of patients were screened only through NT pro-BNP. Across the 1476 patient-years studied, only 6 new cases of PH were identified. PH was secondary to ILD in two cases, and true PAH in three cases (one patient refused diagnostic right-heart catheterisation). All three PAH diagnoses came from echocardiograms requested for worsening dyspnoea, so are not attributable to the screening programme. Many of our patients now take phosphodiesterase 5 inhibitors for severe Raynaud’s phenomenon, which may explain our centre’s low incidence of PAH. The annual cost of screening per patient has dropped from £82 in 2014 to £59 in 2018, and the total annual cost has plateaued since 2014, despite rising patient numbers and improved screening rates.

**Conclusions:** Since introducing NT pro-BNP alongside echocardiography as a screening tool for PAH in SSc patients, we spend less on our screening programme per patient and achieve higher screening rates. However, in 10 years, our screening programme has not detected any asymptomatic cases of PAH, raising questions about the necessity of screening asymptomatic SSc patients.

## P.070

## MANAGEMENT AND BURDEN OF DISEASE OF SSC-ILD IN EIGHT EUROPEAN COUNTRIES: RESULTS OF THE BUILDUP PROJECT

W. Wuyts^1^, J. Rømhild Davidsen^2^, M. Kilpeläinen^3^, M.T. Durheim^4^, S. Papiris^5^, E. Manali^5^, J. Miedema^6^, C. Robalo Cordeiro^7^, A. Morais^8^, M. Perez^9^, G. Asijee^10^, S. Soulard^10^

^1^*Unit for Interstitial Lung Diseases, Department of Respiratory Medicine, University Hospital of Leuven, Leuven, Belgium*, ^2^*South Danish Center of Interstitial Lung Diseases, Department of Respiratory Medicine, Odense University Hospital, Odense, Denmark*, ^3^*Division of Medicine, Department of Pulmonary Diseases and Clinical Allergology, Turku University Hospital and Universit, Turku, Finland*, ^4^*Department of Respiratory Medicine, Oslo University Hospital - Rikshospitalet, Oslo, Norway*, ^5^*2nd Pulmonary Medicine Department, General University Hospital Attikon, Medical School, National and Kapodistrian Uni, Athens, Greece*, ^6^*Department of Respiratory Medicine, Erasmus University Medical Center, Rotterdam, The Netherlands*, ^7^*Department of Pulmonology and Allergy, University Hospital of Coimbra, Coimbra, Portugal*, ^8^*Department of Pneumology of São João Hospital Center, Diffuse Lung Diseases Unit, Oporto, Portugal*, ^9^*Adelphi Spain, Barcelona, Spain*, ^10^*Boehringer Ingelheim, Amsterdam, The Netherlands*

**Introduction:** Interstitial Lung Disease (ILD) encompasses a large group of over 200 pulmonary disorders; most of those are rare, often fibrotic and are associated with a high morbidity and mortality. ILD in Systemic Sclerosis (SSc) is common and is a leading cause of death.

The BUILDup project has investigated the management, costs and burden of disease of fibrosing ILD in 8 European countries (Belgium, Denmark, Finland, Greece, Netherlands, Norway, Portugal and Sweden). Here we report the results for SSc-ILD.

**Material and Methods:** The DELPHI methodology was used to explore the management and burden of disease for limited and extensive SSc-ILD (classification according to Goh et al. - a combination of HRCT and pulmonary function test data, which provides prognostic outcome (mortality) information on ILD due to SSc). An online questionnaire was sent in two waves to pulmonologists and rheumatologists in Q1 and Q2 2019. Questions that did not reach consensus in the first wave of questions were repeated in the second – shorter- wave of questions.

**Results:** 40 healthcare providers (HCP’s), including 32 pulmonologists and 8 rheumatologists, participated in the study and treated on average 20 SSc-ILD patients yearly; of these, 39.1% were classified as having limited disease and 60.9% as extensive disease.

The diagnosis was mainly made by rheumatologists and the mean time from ILD symptom development to definite diagnosis was ~2.1 years. Compared to patients with limited SSc-ILD, healthcare resource use for those with extensive SSc-ILD was ~1.5 times higher with respect to medication and diagnostic tests, ~11 times higher for pulmonary rehabilitation and ~3.5 times higher for hospital admissions. 32.8% of the HCP’s initiated treatment at diagnosis, 42.3% after the first signs of deterioration/progression and 24.7% when the disease becomes extensive. Overall, 84.2% of patients with limited disease were pharmacologically treated compared to 98% of patients with extensive disease. The mentioned most prominent co-morbidities for SSc-ILD patients were gastroesophageal reflux (53.8%), fatigue (40.3%) and depression (22.6%). Finally, over 80 % of the panelists considered the quality of life of SSc-ILD patients to be impaired, with profound impact on sleep, general health, emotion, social life and daily activities. Quality of life of caregivers is also negatively affected.

**Conclusions:** SSc-ILD represents a high burden for patients, caregivers and society. Estimated management costs are substantially higher for extensive disease compared to limited disease.

## P.071

## NINTEDANIB INHIBITS GROWTH FACTOR INDUCED FIBROSIS IN AN IN VITRO FIBROBLAST MODEL OF SYSTEMIC SCLEROSIS

A.S. Siebuhr^1^, M.A. Karsdal^2^, A.-C. Bay-Jensen^1^, P. Juhl^1,3^

^1^*ImmunoScience, Nordic Bioscience Biomarkers and Research, Herlev, Denmark*, ^2^*Nordic Bioscience, Herlev, Denmark*, ^3^*University of Copenhagen, Copenhagen, Denmark*

**Introduction:** Dermal fibroblasts are responsible for the excessive extracellular matrix (ECM) formation observed in the skin of systemic sclerosis (SSc) patients. Fibroblasts are an obvious target for anti-fibrotic treatments. Nintedanib, a tyrosine-kinase inhibitor approved for treatment of idiopathic pulmonary fibrosis, did not show effect on skin fibrosis, only on pulmonary fibrosis, in SSc patients with interstitial lung disease (ILD). We examined nintedanib’s effect on ECM production from human dermal fibroblast using translational biomarkers of type I, III and VI collagens and fibronectin.

**Material and Methods:** Primary healthy human dermal fibroblasts were grown in DMEM media containing 0.4% fetal calf serum, Ficoll (to produce a crowded environment) and ascorbic acid for 17 days. The cells were activated with either PDGF [3 nM] or TGF-β [1 nM]. Nintedanib [1 nM-10 μM] treatment was initiated at day 0 or 7 together with PDGF or TGF-β. Media and treatments were changed twice a week. Non-activated cells (w/o) were used as control. Type I, III and VI collagen formation (PRO-C1, PRO-C3 and PRO-C6, respectively) and fibronectin (FBN-C) were evaluated by validated ELISAs (Nordic Bioscience). Statistical analysis included 2-way ANOVA and Mann-Whitney T-test. μ

**Results:** PDGF significantly increased PRO-C3 and PRO-C6 (peaking at day 14, 21.8 and 44.7-fold, p=0.0001, respectively) and PRO-C1 minimally (1.7-fold, p<0.034). PDGF did not induce FBN-C. TGF-β increased PRO-C1 from day 3 peaking at day 14 (4.6-fold, P<0.0001) and PRO-C6 peaking at day 7 (3.5-fold, P<0.0001). TGF-β increased FBN-C at all days with a peak at day 7 (3.5-fold, p=0.0002), but did not induce PRO-C3.

Nintedanib (>=100 nM) added either from day 0 or 7 reduced PDGF induced PRO-C3 and PRO-C6 to the levels of w/o throughout the remainder of the study. In TGF-β treated fibroblasts, nintedanib added either from day 0 or 7 reduced PRO-C1, PRO-C6 and FBN-C dose-dependently and the biomarker levels were at study end at the level of w/o. Nintedanib at a concentration of 1 μM and higher significantly decreased the biomarker levels.

**Conclusions:** TGF-β and PDGF induced formation of different ECM profiles; PDGF type III and VI collagen and TGF-β type I and VI collagen and fibronectin. Nintedanib inhibited ECM production differently in PDGF and TGF-β induced dermal fibroblast. In PDGF induced fibrosis, it acted as an on-off switch, whereas the inhibition was dose-dependent in TGF-β induced fibrosis. This cell study indicates that nintedanib may inhibit skin fibrosis contrary to the resent clinical trial of nintedanib in SSc-ILD.

## P.072

## PLEURO-PARENCHYMAL FIBROELASTOSIS IN SYSTEMIC SCLEROSIS ASSOCIATED INTERSTITIAL LUNG DISEASE

A. Sari^1^, O. Onder^2^, B. Armagan^1^, E. Bolek^1^, B. Farisogullari^1^, E. Bilgin^1^, G. Yardimci^1^, M. Ariyurek^2^, A. Akdogan^1^

^1^*Hacettepe University Medical School, Department of Rheumatology, Ankara, Turkey*, ^2^*Hacettepe University Medical School, Department of Radiology, Ankara, Turkey*

**Introduction:** Pleuroparenchymal fibroelastosis (PPFE)-like lesions are exaggerated fibrotic changes located in bilateral upper lung zones and accompanied by subpleural opacities and pleural thickening. Data about frequency and clinical significance of these lesions in interstitial lung diseases (ILD) is limited. The aim of this study was to determine the frequency and clinical significance of PPFE-like lesions in ILD associated with systemic sclerosis (SSc).

**Material and Methods:** SSc-ILD patients admitted between 2015 and 2018 were included. High resolution computed tomography (HRCT) images of the patients were examined by two radiologists for the presence of PPFE-like lesions. Demographic, clinical, laboratory and pulmonary function test (PFT) data of patients with and without PPFE-like lesions were compared.

**Results:** Among 152 patients with SSc-ILD, 105 had available HRCT scans. PPFE-like lesions were detected in 13 (12.4%) patients. Disease duration at first available HRCT scan was higher in patients with PPFE-like lesions (16 vs. 10 years, p<0.001). Gender, age at disease onset, cutaneous subtype, autoantibody specifity and the frequency of overlapping myositis did not differ between groups. Patients with PPFE-like lesions had lower vital capacities (FVC), and the frequency of patients with FVC below 70% during follow-up was higher this group, but the differences were not statistically significant 70% vs 82% p=0.08 and 71% vs 36%, p=0.18). Although the follow-up period was shorter, mortality was more frequent in the PPFE (+) group (38 vs. 12, p=0.03). Spontaneous pneumothorax developed in 1 patient with PPFE-like lesions during follow-up.

**Conclusions:** PPFE-like lesions are not uncommon in the course of SSc, and may be associated with more severe ILD and complications such as pneumothorax. Prospective studies are needed on this subject.

## P.073

## COMPARISON BETWEEN DIFFERENT THERAPEUTICAL APPROACH TO SYSTEMIC SCLEROSIS (SSC) RELATED INTERSTITIAL LUNG DISEASE (ILD)

M. Saracco, T. Oddenino, C. Lomater

*Rheumatology Unit - Ospedale Mauriziano, Turin, Italy*

**Introduction:** ILD is one of the leading causes of morbidity and mortality in SSc.

In years different therapeutical approach have been tried to improve SSc-ILD outcome.

The aim of this study is to compare different therapeutic scheme in meaning of efficacy in reduce fibrosis, improving pulmonary functional test (PFT) and side effects.

**Material and Methods:** We used the historical cohort of our Rheumatology center and collected HRCT and PFT before and after 6 months treatment of ILD.

We collected long term side effects and new episode of worsening in ILD evolution.

The treatment we used in our center are so divided: oral cyclophosphamide, iv pulse cyclophosphamide, mycophenolate/ azathioprine and rituximab (Table 1).

We enrolled in the study 52 patient with a diagnosis of SSc according to ACR/EULAR criteria between 1990 and 2019, with an ILD established by HRTC and PFT. The fibrosis extent were evaluated by OsiriX open-source software.

Statistical analysis were performed using the Spearman test because the non-normal distribution of study population.

Treatment efficacy were defined as an improvement >/= 10% in FVC and DLCO values; then PFT were correlated with fibrosis index (FI) in HRTC with a rho cut-off of correlation between 0,35 and 0,6.

PFT and HRCT were performed before, after and 6 month after iv treatment; before and after 6 months of treatment for oral therapy.

**Results:** Only the Rituximab treatment were correlated with a positive variation in PFT while in our cohort cyclophosphamide seems to not influence functional test (Table 2-3); results are not statistically significant

The correlation between PFT and HRCT is observed just for DLCO (r > 0.46; p < 0.05).

Regarding long term side effects we observed neoplasm in 6 patients treated with oral or iv CTX and none in RTX or MMF group.

In the RTX group we observed a greater incidence of infective disease with, in two patients, discontinuation in treatment.

The number of patients who needed a second line treatment is too little for a significant difference.

**Conclusions:** This is the first study comparing different therapeutical approach in SSc-ILD in real life. The limit of this study is the poor number of study population and the lack of all HRCT because exams performed in 1990 were not suitable for OsiriX study.

Further investigation with a larger cohort can be useful to define which is the best first line treatment for this kind of patients most of all regarding long term efficacy and side effects.

**Figure:**
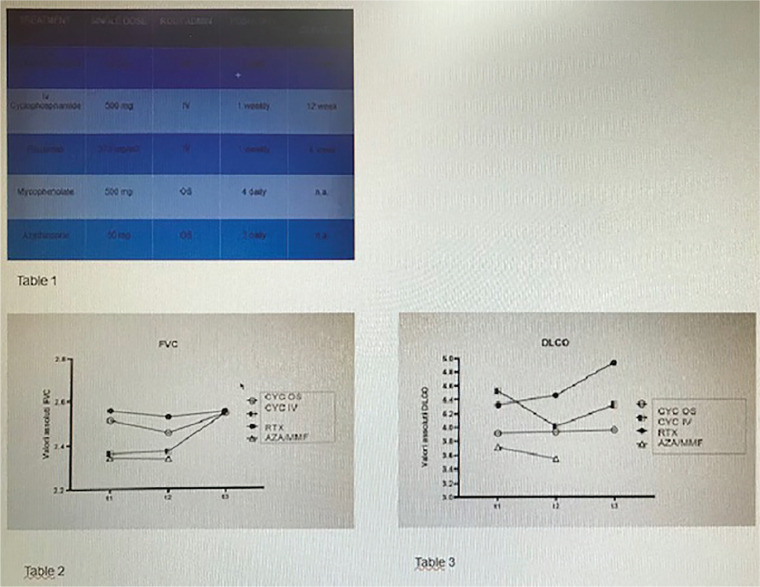


## P.074

## WHICH TREATMENT ASPECTS MATTER TO PATIENTS WITH SYSTEMIC SCLEROSIS ASSOCIATED INTERSTITIAL LUNG DISEASE? THE DEVELOPMENT OF A PATIENT PREFERENCE INSTRUMENT

L.A. Saketkoo^1^, C. Bruni^2^, N. Schoof^3^, S. Heidenreich^4^, A. Duenas^4^, M. Alves^3^, A. Hoffmann-Vold^5^, A. Gabrielli^6^, O. Distler^7^

^1^*New Orleans Scleroderma & Sarcoidosis EUSTAR Patient Care & Research Ctr, Louisiana State Uni/Tulane Uni School of Med, New Orleans, USA*, ^2^*Dept. of Rheumatology/Scleroderma Unit, University of Florence, Florence, ITALY*, ^3^*Boehringer Ingelheim International GmbH, Ingelheim Am Rhein, Germany*, ^4^*Evidera, London, United Kingdom*, ^5^*Dept. of Rheumatology, Oslo University Hospital, Oslo, Norway*, ^6^*Dept. of Clinical and Molecular Sciences, Polytechnic University of Marche, Ancona, Italy*, ^7^*Dept. of Rheumatology, University Hospital Zurich, Zurich, Switzerland*

**Introduction:** Systemic sclerosis (SSc) is an autoimmune disease characterized by progressive skin and internal organ fibrosis. Interstitial lung disease (ILD) is common in patients and associated with high morbidity and mortality. Immunosuppression is the standard of care, with treatment choice being influenced by the disease complexity and treatment profiles. Patients’ preferences are essential in identification of optimal therapy and treatment development. We present a conceptual framework for a patient preference instrument to elicit patients’ treatment priorities.

**Material and Methods:** A literature review identified relevant treatment attributes, which were discussed in interviews with nine SSc-ILD patients from New Orleans (US), to assess importance and trade-offs. The interviews had two parts: Part 1 discussed symptoms, quality of life and treatment experiences. Part 2 was a hypothetical choice between two treatments with different attribute profiles. Following the interviews, a discrete choice experiment (DCE) was developed to quantify patients’ preferences. An online version of the DCE was pretested by three patients from the US, the UK and Italy. One patient completed the DCE together with an interviewer, and two patients completed a debriefing questionnaire. SSc-ILD patients were recruited through the European Scleroderma Trials and Research Group (EUSTAR) from the EUSTAR registry or based on medical records.

**Results:** The nine SSc-ILD patients interviewed (Table 1) suffered from symptoms such as coughing (78%), shortness of breath (56%), and fatigue/dizziness (56%), which affected their social life (100%), physical activity (67%) or work productivity (67%). Symptom improvement was considered as an important treatment attribute. When presented with the hypothetical treatment choice (Table 2), patients voiced a willingness to make trade-offs between benefits and treatment risks, such as gastrointestinal side effects or increased likelihood of infections. Based on patients’ feedback in the interviews, a DCE with 12 choices between two SSc-ILD treatments was generated. Treatments were described by seven attributes: 1) mode of administration; 2) shortness of breath; 3) skin tightness; 4) coughing; 5) tiredness; 6) risk of diarrhoea, nausea and/or vomiting; 7) risk of infections. The three patients who pretested the DCE expressed the choice tasks were clear and easy to understand. Minor changes were made after the pretest.

**Conclusions:** The interviews demonstrated the relevance of identified treatment attributes and patients’ willingness to make trade-offs. The DCE pretesting suggested that the preference elicitation survey was accessible to patients with next steps being fielding.

**Funding:** Boehringer Ingelheim International GmbH, Germany

**Figure:**
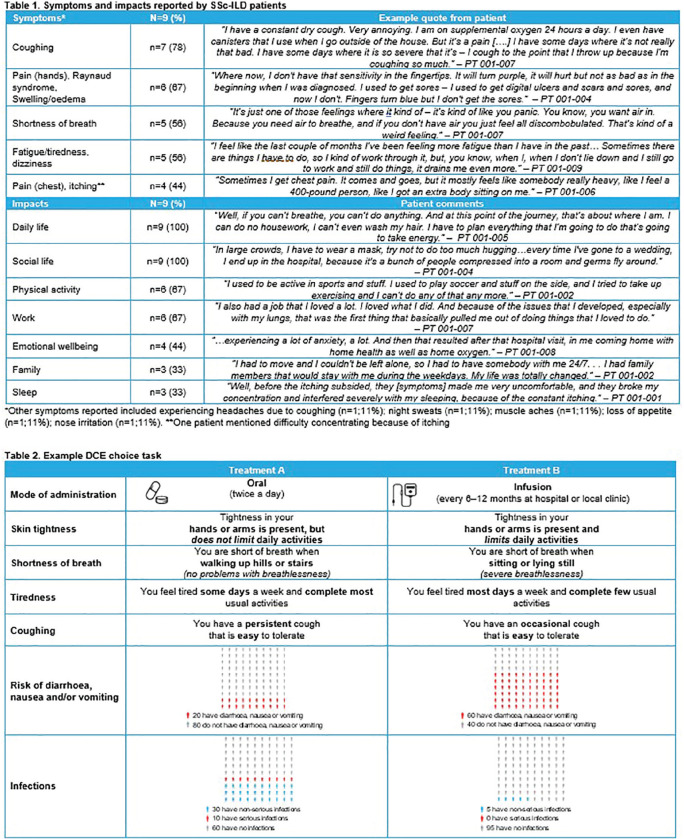


## P.075

## PREVALENCE AND SURVIVAL OF SYSTEMIC SCLEROSIS (SSC) AND ASSOCIATED INTERSTITIAL LUNG DISEASE IN ONTARIO, CANADA OVER 10 YEARS

J.E. Pope^1^, K. Quansah^2^, M. Kolb^3^, J. Flavin^2^, S. Hassan^4^, S.J. Seung^4^, K. Garlick^2^

^1^*Schulich School of Medicine and Dentistry, Western University, London, Canada*, ^2^*Boehringer Ingelheim (Canada) Limited, Burlington, Canada*, ^3^*Firestone Institute for Resipiratory Health, McMaster University, Hamilton, Canada*, ^4^*HOPE Research Centre, Sunnybrook Research Institute, Toronto, Canada*

**Objective:** Systemic sclerosis (SSc) is an autoimmune disease characterized by skin thickening, vascular lesions and fibrotic changes in various organs, mainly the lungs, heart, intestinal tract, kidneys, muscles and joints. Pulmonary complications of SSc are one of the leading causes of morbidity and mortality. Interstitial lung disease (ILD) is among the most common forms of lung disease associated with SSc. To date, no published study has generated population-based estimates of the prevalence of SSc-ILD in Canada. The objective of this study was to develop prevalence and survival estimates of SSc and SSc-ILD in Ontario, Canada using administrative data over 10 years.

**Methods:** Adult patients diagnosed with SSc between April 1, 2008 and March 31, 2018 were identified from the National Ambulatory Care Reporting System (NACRS) and Discharge Abstract Database (DAD) administrative databases, using ICD 10 CA codes. Patients with SSc were identified first, using M34 ICD-10 CA codes (M34.0, M34.1, M34.2, M34.8, and M34.9). SSc-ILD patients were identified if an additional one of J84.1, J84.8, J84.9 or J99.1 codes for lung disease was used after the SSc diagnosis. Prevalence estimates were determined for both SSc and SSc-ILD, based on the population of all eligible Ontario adults (~11.0 million as of fiscal 2017). Descriptive statistics and Kaplan Meier survival curves were generated.

**Results:** There were 3,111 unique patients identified as having SSc over 10 years. Of those, 559 (18%) were further identified as having SSc-ILD. At the start of fiscal year 2017/18 (final year of the cohort), there were 2,114 prevalent SSc cases for a cumulative prevalence of 19.12 per 100,000 persons from 2008/9 to 2017/18. Over the same time frame, there were 257 prevalent cases of SSc-ILD, generating a prevalence of 2.32 cases per 100,000 persons. At index date, mean age was approximately 57 and 58 years of age for SSc and SSc-ILD patients with 84% and 80% females in the groups. The survival rates at one, five and ten years after diagnosis for the SSc group were 84.96%, 64.45% and 44.88%, respectively. The SSc-ILD group survival rates at one, five and ten years were lower, at 77.12%, 44.41% and 22.02%, respectively.

**Conclusions:** This study provides the first population based estimates of SSc-ILD in Canada. Results confirm that the prevalence of SSc-ILD may fall within a Canadian threshold for drugs for ‘other’ rare disease. It also demonstrates the poor survival in SSc and especially when SSc-ILD is present.

## P.076

## CORRELATION BETWEEN PARAMETERS OF PULMONARY FUNCTION AND ANTI-SCL-70 ANTIBODIES IN THE PATIENTS WITH SSC AND ILD OVER 5 YEAR FOLLOW UP

O. Ovsyannikova, O. Koneva, L. Ananieva, L. Garzanova, O. Desinova, M. Starovoytova

*V.A. Nasonova Research Institute of Rheumatology, Moscow, Russia*

**Introduction:** Taking into account that anti-Scl-70 antibody is an unfavorable predictor of ILD, we have assessed the time course of FVC and DLco in anti-Scl-70-positive and anti-Scl-70-negative patients.

**Material and Methods:** it was a longitudinal study involving 77 pts with SSc-ILD (mean age was 46.2 ± 13,4; 69% have limited subset of the disease; 93% were female). The mean duration of follow up was 58.9 ± 11,4 months. Pts were investigated with HRCT twice ( at first visit and at the end of the study) and according the CT –changes were divided into 3 groups: the 1st group (16 pts) with improvement; 2nd group (39 pts) without any changes and 3rd group (22 pts) with worsening of fibrosis. We evaluated the forced vital capacity (FVC), diffusing capacity of carbon monoxide (DLco) in one year and in 5 year and anti-Scl-70 antibodies at the end of the study.

**Results:** In anti-Scl-70-negative patients the average FVC remained unchanged within a year (91.4 ± 17.4% and 91.6 ± 17%, respectively), however, within 5 years FVC significantly increased to 99.1 ± 20% (p < 0.001), while in anti-Scl-70-positive patients the average FVC was lower at the baseline and remained virtually unchanged both 1 year and 5 years later (85.4 ± 17.3%. 85.5 ± 17 and 86 ± 21%, respectively), as a result in 5 years’ time the average FVC in anti-Scl-70-negative patients was significantly higher than in anti-Scl-70-positive patients (p = 0.009).

At the baseline average DLco were below normal and were similar in anti-Scl-70-positive and anti-Scl-70-negative patients: 60 ± 19% and 61.2 ± 14.3% respectively (p>0,05). Within 1 year DLco significantly decreased in all patients, however anti-Scl-70-positive patients demonstrated more pronounced DLco decrease to 53.4 ± 17.2% (p<0.0001), while in anti-Scl-70-negative patients it was less pronounced to 57.2 ± 12.8% (p<0.05), respectively. Within 5 years significant DLco decrease was found only in anti-Scl-70-positive patients: 53.9 ± 16.4% (p<0.001).

Significant DLco decrease over the 5-year period was observed in Group 2 in anti-Scl-70-positive pts from 62 ± 19 to 56 ± 14 and Group 3 in anti-Scl-70-positive pts from 52 ± 17 to 45 ± 16 (p<0.05) respectively, and the lowest DLco (<50%) were observed in Group 3.

**Conclusions:** Therefore, the presence of anti-Scl-70 antibodies was associated with significantly lower pulmonary function parameters specifically in pts with progressive ILD.

## P.077

## ASSOCIATION PROGRESSION PULMONARY ALTERATIONS BY HRCT OF WITH EUROPEAN SCLERODERMA STUDY GROUP ACTIVITY INDEX (ESCSG-AI) IN PATIENTS WITH SYSTEMIC SCLEROSIS OVER A FIVE YEAR PERIOD

O. Ovsyannikova, O. Koneva, L. Ananieva, L. Garzanova, O. Desinova, M. Starovoytova

*V.A. Nasonova Research Institute of Rheumatology, Moscow, Russia*

**Introduction:** Systemic sclerosis (SSc) is a rare connective tissue disease with a heterogeneous clinical course. Interstitial lung disease (ILD) is a common manifestation of SSc and a leading cause of death. Patients with early active SSc are at great risk for progressive ILD.

Objectives: To assess the EScSG-AI in patients with systemic sclerosis (SSc) and interstitial lung disease over a five year period.

**Material and Methods:** it was a longitudinal study involving 77 pts with SSc-ILD (mean age was 46,2±13,4; 69% have limited subset of the disease; 93% were female). The mean duration of follow up was 58,9±11,4 months. Pts. were investigated with HRCT twice (at first visit and at the end of the study) and according the CT-changes were divided into 3 groups: the 1st group (16 pts) with improvement; 2nd group (39 pts) without any changes and 3rd group (22 pts) with worsening of fibrosis. Disease activity was assessed by the 2001 European Scleroderma Study Group Activity Index (EScSG- AI).

**Results:** the most pts in this study exhibited stabilization in HRCT features of ILD. There were no significant differences between groups related to sex, frequency of diffuse form and duration disease. Mean values of EScSG-AI score of all pts and in the each groups in first visit and the end of follow up are present in table 1.

**Figure:**
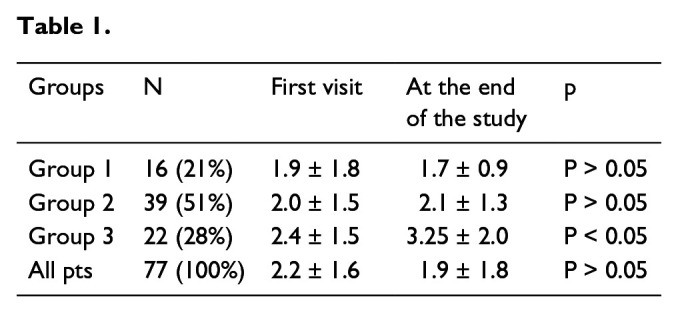


After 5 years of follow up mean values of EScSG-AI score increased significantly in group 3 and was more than 3, this actually means the activity of the disease. The mean values of EScSG-AI score in group 3 was significantly higher than in groups 1,2 (p < 0,01 and p < 0,04 accordingly) at the end of the study.

**Conclusions:** Conclusions: Mean values of EScSG-AI score associated with and were accompanied by progression pulmonary alterations in pts SSc so EScSG-AI score can be used as valuable tool for long-term follow-up studies.

## P.078

## PULMONARY FUNCTION IN PATIENTS DIAGNOSED OF EARLY SYSTEMIC SCLEROSIS: A NEW TOOL FOR SYSTEMIC SCLEROSIS CLASIFFICATION?

F. Ortiz-Sanjuán, C. Alcañiz, I. Cánovas, I. Chalmeta, M. De La Rubia, J. Fragio, R. González, L. González, E. Grau, S. Leal, I. Martínez, C. Nájera, R. Negueroles, C. Pávez, J. Oller, E. Vicens, J. Ivorra, J.A. Román-Ivorra

*Hospital Universitario y Politécnico La Fe-Rheumatology Department, Valencia, Spain*

**Introduction:** Interstitial lung disease (ILD) is a frequent complication of systemic sclerosis (SSc) and is often progressive and has a poor prognosis. A restrictive ventilatory defect could suggest ILD either alone or in combination with pulmonary arterial hypertension.

Nowadays, Early-SSc is well defined as preliminary stage of SSc. Patients who meet criteria for Early-SSc could benefit from an early diagnosis of pulmonary involvement.

Our aim was to assess the pulmonary function in patients diagnosed of Early SSc.

**Material and Methods:** Retrospective observational study of a wide and unselected series of patients diagnosed as Early-SSc from a single university hospital from 2012 to 2019. Patients were classified as Early-SSc following Le Roy criteria. Despite this, patients already did not meet 2013 ACR/EULAR classification criteria for SSc. We reviewed pulmonary function through conventional spirometry and diffusing capacity of lung for carbon monoxide (DLCO).

**Results:** We included 56 patients with a mean age of 52.3±12.1 years (96.4% women; 3.6% men).

At the diagnosis of Early-SSc, no one of our patients evidenced a restrictive ventilatory pattern. DLCO was below normal limits in 18 patients (32.1%). Small airway obstruction expressed according decreased maximal (mid-) expiratory flow (MMEF) 25-75 was present in 24 patients (42.8%).

After a mean follow-up period of 38.3±2.4 months, 29 (51.8%) patients fulfilled 2013 ACR/EULAR criteria. The average time between diagnosis of Early-SSc and achieve SSc classification was 24.4±1.8 months. The remaining 27 patients continued classified as Early-SSc.

An analysis of the subgroup of patients which progressed to SSc showed that DLCO was decreased in 15 of those 29 patients (51.7%) and 18 of 29 patients (62.1%) presented decreased MMEF 25-75. Comparing with the subgroup of patients which not progressed to SSc were significant differences (Decreased DLCO: 51.7% vs 11.1%; p=0.02 and decreased MMEF 25-75: 42.8% vs 22.2%; p=0.05).

The analysis of pulmonary function of the subgroup of patients continued classified as Early-SSc after follow-up period did not show significative changes after follow-up.

**Conclusions:** In our study, a third of the patients classified as Early-SSc presented at diagnosis abnormal values of DLCO and/or signs of small airway obstruction without the presence of a restrictive ventilatory pattern. Moreover, this pulmonary disfunction was significantly more frequent in patients who progressed to definitive SSc. Patients which remains classified as Early-SSc did not experience significative changes.

Our results support the concept that pulmonary function was impaired in Early-SSc and that I should probably be considered for future Early-SSc classification criteria.

## P.079

## FORCED VITAL CAPACITY TRAJECTORIES IN PATIENTS WITH SYSTEMIC SCLEROSIS ASSOCIATED PULMONARY FIBROSIS

S.I. Nihtyanova, E.C. Derrett-Smith, C. Fonseca, V.H. Ong, C.P. Denton

*UCL Medical School, Royal Free Campus, Centre for Rheumatology and Connective Tissue Diseases, London, United Kingdom*

**Introduction:** Pulmonary fibrosis (PF) is common in systemic sclerosis (SSc) and serial pulmonary function tests (PFTs) are used for routine PF monitoring. Forced vital capacity (FVC) decline reflects progression in PF and FVC is frequently used as an endpoint in clinical trials. We explore the changes in FVC over time in patients with SSc-PF, receiving standard management, including immunosuppression.

**Material and Methods:** Only SSc patients with CT-confirmed PF were included. FVC changes over the first 10 years from onset and the effects of age, sex, cutaneous subset and autoantibodies were assessed using linear mixed effects models.

**Results:** We identified 505 SSc-PF subjects, 21.6% male, average age at onset 47 years and 49.3% had diffuse cutaneous subset (dcSSc). The most common autoantibody was anti-Scl70 in 40.4%, followed by anti-RNA polymerase (ARA) in 11.7% and anti-centromere (ACA) in 7.1%. In 16.4% of the patients ANA was positive, but no ENAs were identified (ANA+ENA-). FVC measurements on at least three occasions were available for 72.1% of the patients. Mean period between PFTs was 13 months.

For the whole cohort average FVC at 12 months from onset was 80.1% (SD 19.3) and there was a small but significant absolute decline of approximately 0.32% per year (p=0.007) at a group level. There was no evidence for a significant correlation between baseline FVC and subsequent change (correlation coefficient -0.13; 95%CI -0.26, 0.01).

Multivariable analysis demonstrated significant associations between FVC and age at onset, sex, cutaneous subset and antibodies. For every year older age at onset, average FVC increased by 0.32%, p<0.001. Male patients had 3.3% lower FVC at 1 year of disease compared to females (p<0.001) and demonstrated much faster FVC drop over time (additional 0.6% per year, p=0.034). DcSSc subjects had 5.6% lower FVC compared to those with limited disease (p=0.003). Autoantibodies showed strong association with baseline FVC and changes in FVC over time. Average FVC at 1 year from onset in ARA+ subjects was significantly higher than any other antibody group (15.1% v. ANA+ENA-, p<0.001; 14.6% v. ATA, p<0.001 and 12.5% v. ACA, p=0.010). Nevertheless, ARA+ subjects had significant drop in FVC over time, which was comparable to ATA+ and ANA+ENA-subjects, while FVC in ACA+ PF patients demonstrated a small but significant increase over time.

**Conclusions:** This study provides insight into long-term patterns of FVC change and develops a model that may help predict those most at risk of significant decline.

## P.080

## ABNORMAL ESOPHAGOGRAM PREDICTS PULMONARY FUNCTION DETERIORATION IN PATIENTS WITH SYSTEMIC SCLEROSIS

L. Gigante^1^, E. De Lorenzis^1^, G. Natalello^1^, L. Berardini^3^, G.B. Canestrari^2^, L. Verardi^1^, E. Gremese^1,2^, L. Richeldi^3^, S.L. Bosello^2^

^1^*Institute of Rheumatology, Catholic University of the Sacred Heart, Rome, Italy*, ^2^*Department of Rheumatology, Fondazione Policlinico Universitario A. Gemelli - IRCCS, Rome, Italy*, ^3^*Department of Pulmonology, Fondazione Policlinico Universitario A. Gemelli - IRCCS, Rome, Italy*

**Introduction:** Interstitial lung disease (ILD) is the leading cause of death in systemic sclerosis (SSc) but its pathogenesis and the risk factors of pulmonary function deterioration are not fully understood. Esophageal disease is high frequent in SSc and motor activity abnormalities with occult micro-aspiration of both acid and non-acid gastro oesophageal reflux has been implicated in the pathogenesis of ILD. DLCO reduction is considered the earliest sign of microaspiration-induced lung damage. Cross-sectional studies have demonstrated an association of SSc-ILD and esophageal abnormalities on intraesophageal pH-monitoring and esophageal manometry, but prospective evaluation of lung deterioration is lacking. Esophagogram was proposed as a useful tool to evaluate disease severity of upper gastrointestinal tract involvement in SSc. We evaluated the role of esophagogram in predicting pulmonary function test deterioration in SSc-patients.

**Material and Methods:** We retrospectively evaluated 160 consecutive SSc patients who underwent esophagogram because of suspected upper gastro-intestinal involvement. All patients underwent baseline pulmonary function tests and global clinical evaluation. Eighty-five patients underwent a High Resolution CT within 3 months from esophagogram because of suspected lung involvement. One hundred twenty three patients underwent pulmonary function test every 6 months up to 24 months.

**Results:** Seventy five patients (46.9%) presented abnormalities of peristaltic waves, 50 patients (31.2%) showed structural changes (hypotonic oesophagus or dilatation) while indirect signs of cardial incontinence (patent cardia or gastro-esophageal reflux) were present in 36 patients (22.5%). A reduced peristaltic activity with a prolongation of transit time was associated to reduced DLCO (50.2±19.8%vs60.4±22.7%, p=0.002) and TLC (87.1±20.4%vs95.1±20.6%, p=0.017). An hypotonic esophagus was reported in 25.2% of patients and it was associated to ILD on CT (72.0% vs 28.0%, p=0.033). Patients with hypotonic esophagus presented a reduced FVC (84.6±22.9%vs102.9±21.4%, p<0.001), TLC (79.8±19.6% vs 95.3±19.8%, p<0.001) and DLCO (42.9±20.0% vs 59.9±20.8%, p<0.001) at baseline and to a faster deterioration of DLCO median values (5.1±20.6%vs-4.8±14.2%, p=0.012) at follow-up. Patients with hypotonic esophagus have an higher prevalence of diffuse skin disease and ongoing immunosuppressive treatment, but were comparable in term of age, sex, BMI, smoking habits, disease duration and prevalence of autoantibodies to the patients without this alteration.

**Conclusions:** The esophagogram is a widely available, well tolerated and inexpensive tool to assess due to upper gastro-intestinal tract involvement, and its abnormalities are associated to SSc-ILD severity. Since a faster deterioration of lung function appears to be linked to esophagogram alterations, a complete gastro-intestinal assessment in patients with ILD-SSc is mandatory.

## P.081

## ESOPHAGEAL EROSIONS PREDICT PROGRESSION OF LUNG DISEASE IN PATIENTS WITH SYSTEMIC SCLEROSIS

E. De Lorenzis^1^, G. Natalello^1^, L. Berardini^3^, G.B. Canestrari^2^, L. Gigante^1^, L. Verardi^1^, E. Gremese^1,2^, L. Richeldi^3^, S.L. Bosello^2^

^1^*Institute of Rheumatology, Catholic University of the Sacred Heart, Rome, Italy*, ^2^*Department of Rheumatology, Fondazione Policlinico Universitario A. Gemelli - IRCCS, Rome, Italy*, ^3^*Department of Pulmonology, Fondazione Policlinico Universitario A. Gemelli - IRCCS, Rome, Italy*

**Introduction:** Interstitial lung d isease (ILD) is the leading cause of death in Systemic Sclerosis (SSc); esophageal disease is common in SSc and micro-aspiration of both acid and non-acid gastroesophageal reflux could be involved in the pathogenesis of ILD. Esophagogastroduodenoscopy (EGD) is an essential tool to evaluate disease severity of upper gastrointestinal tract involvement in SSc. We evaluated the role of EGD in predicting pulmonary functional deterioration in SSc patients.

**Material and Methods:** One hundred and fifty patients with SSc and suspected esophageal involvement underwent EGD. Pulmonary function tests were performed at baseline and after a 36-months follow-up. Patients were characterized for disease phenotype, BMI, smoking exposure and medication history. A significant ILD progression was defined as a relative decline >= 10% of FVC or a concomitant decline >= 5% of FVC and >= 15% of DLCO.

**Results:** One hundred and thirty-six patients (90.5%) were female with a mean age of 55.6±13.8 years and 12.1% were active smokers. Fifty patients (33.3%) had a diffuse cutaneous disease; 37.4% and 40.8% were positive for anti-centromere and anti-Scl70 antibodies respectively. The mean disease duration from the first non-Raynaud symptom was 5.9±6.7 years. Sixty-one patients (40.8%) showed EGD signs of reflux esophagitis. Among them, 31.3% had an erosive form (9.5% grade A, 15.6% grade B, 4.8% grade C and 1.4% grade D according to Los Angeles classification). At the baseline, 23.1% of the patients had a FVC<=80% and 45.6% had a DLCO<=50%. Patients with erosive esophagitis did not differ in terms of sex, age, duration and disease variant, positivity for anti-centromere, skin score values, FVC and DLCO at baseline compared to patients without erosions, but had a lower prevalence of anti-Scl70 (28.3%vs52.5%, p=0.005) and active smoking (20.0%vs8.4%, p=0.05). At follow-up, patients with esophageal erosions showed a greater relative decrease in FVC (3.4%±9.3%vs1.7%±12.0%; p=0.013) without significant differences in the DLCO change. Overall, 11.0% of patients presented pulmonary disease progression. The presence of esophageal erosions was associated with a significantly greater risk of lung disease progression (OR 5.3, 95% CI 1.7-16.8, p=0.004) after paired correction for sex, age, duration of disease, auto-antibodies, skin involvement variant, baseline FVC and DLCO, smoke exposure and therapy with immunosuppressants, proton pump inhibitors, prokinetics, antiplatelet agents and prostanoids.

**Conclusions:** SSc patients with erosive esophagitis present a higher risk of progression of interstitial lung disease. This evidence supports a role of micro-aspiration of gastric contents in the development of inflammation and fibrosis of the airways.

## P.082

## OUTCOME OF INTERSTITIAL LUNG DISEASE IN PATIENTS WITH SCLERODERMA : A 5 YEAR PROSPECTIVE COHORT STUDY

R. Nanavati, R. Samant, A. Khune

*P.D. Hinduja National Hospital and Medical Research Center, Mumbai, India*

**Introduction:** Systemic sclerosis (SSc) is a multi-system disorder characterized by a disturbance in fibroblast function, microvascular disease, and immune system activation, resulting in fibrosis of the skin and internal organs

**Material and Methods:** To study changes in lung function in patients with scleroderma with ILD on treatment followed up prospectively over a period of 5 years

In this study, 73 patients with scleroderma with ILD were prospectively followed up from July 2013 to July 2018. Patients’ clinical features, investigations, treatment and complications were recorded at regular interval.

**Results:** Of 73 patients, 68 were females and 5 were males. 4 patients had only one visit and 11 patients had incomplete data. Mean age study population was 43.24 ± 11.70 years with mean duration of disease 10.02 ± 6.86 years and mean duration of ILD 6.02 ±2.94 years. Mean prospective follow up duration was 3.80 ± 2.28 years. 43 Patients had GERD and 36 had arthritis and/or arthralgia. Mean PAH at baseline was 35.95 ± 14.26 mmHg and at end of study was 39.44 ± 14.44 mmHg. Immunosuppressant’s used were MMF in 52, cyclophosphamide in 15, azathioprine in 13, and methotrexate in 17 patients. 61 patients received oral prednisolone with mean dose of 6.46 ± 7.10 mg/day during maintenance and mean dose 15 ± 8.95 mg/day during exacerbation of ILD. There was no statistically significant difference in mean FVC% of 59 patients at baseline (59.76% ± 16.91%) and at end of the study (57.03% ± 16.77%) and in subgroup analysis of patients who received MMF (p > 0.05). Mean 6MWD at baseline was 370 ± 58.83 meters and at end of study was 377 ± 88.96 meters. Comorbidities seen were hypertension in 15, DM in 6, osteoporosis in 23 (of which one had vertebral fracture), AVN in 3 patients. 5 patients died because of ILD. 13 patients needed home O2.

**Conclusions:** This prospective study was carried out with aim to determine progression of ILD in patients with systemic sclerosis with ILD over period of 5 years

In our study, there was no statistically significant change of FVC over period of time. Worsening of lung function with deterioration in FVC >10% was seen in only 13 patients (22%) (P<0.01).Our findings are similar to study done by Le Gouellec etal in which 75 patients with scleroderma and ILD were followed over period of 72 months of which 26% patients showed significant worsening of FVC

## P.083

## ANALYSIS OF SERUM CARBOHYDRATE ANTIGENS (CA 19-9, CA 125, CA 15-3), CARCINOEMBRYONIC ANTIGEN AND ALPHA FETOPROTEIN IN SYSTEMIC SCLEROSIS PATIENTS WITH ASSOCIATED INTERSTITIAL LUNG DISEASES (ILD)

C. Naclerio^1^, A. Cavallera^2^, T. Urraro^1^, S. Scarpato^1^

^1^*Unit of Rheumatology-Scarlato-Hospital, Scafati, Italy*, ^2^*Unit of Radiology-Scarlato-Hospital, Scafati, Italy*

**Introduction:** Interstitial lung diseases (ILDs) can be associated with Connective tissue diseases. This study aims to detect serum carbohydrate antigens levels in Systemic Sclerosis (SSc) associated interstitial lung disease (CTD-ILD) patients and to demonetate their values in evaluating the activity of SSc-ILD.

**Material and Methods:** The study included 26 Systemic Sclerosis ILD patients ( 25 females, 1 men, mean age 57 years). Correlations of serum carbohydrate antigens (CA 19-9, CA 125, CA15-3), carcinoembryonic antigen and alpha fetoprotein with disease severity parameters (pulmonary function and involvement score on high resolution computed tomography) were analyzed.

**Results:** Serum CEA, alpha fetoprotein, serum carbohydrate antigens were normal. No correlation was demonstrated between serum antigens values and pulmonary function test and radiological parameters. Our study provides for the setting up of one wide population of patients with SSc.

**Conclusions:** On the other hand, other Authors have studied different pathologies such as rheumatoid arthritis, polymyositis, dermatomyositis, Sjogren syndrome, SSc; we have analyzed serum antigens in SSc patients only.

There wasn’t any correlation between pulmonary funcion test and serum antigens values.

## P.084

## A NEW FUNCTIONAL STAGING OF SSC-RELATED INTERSTITIAL LUNG DISEASE WITH PROGNOSTIC IMPLICATIONS

A. Santaniello^1^, L. Bettolini^1^, C. Bellocchi^1^, M. Cassavia^1^, G. Montanelli^1^, B. Vigone^1^, A. Severino^1^, M. Caronni^1^, C. Campochiaro^2^, E. De Lorenzis^3^, L. Cavagna^4^, G. De Luca^2^, S.L. Bosello^3^, L. Beretta^1^

^1^*Scleroderma Unit, Fondazione IRCCS Ca Granda Ospedale Maggiore Policinico di Milano, Milan, Italy*, ^2^*Unit of Immunology, Rheumatology, Allergy and Rare Diseases IRCCS S Raffaele Hospital, Università Vita-Salute S Raffaele, Milan, Italy*, ^3^*Rheumatology Unit, Fondazione Policlinico Universitario A. Gemelli IRCCS, Rome, Italy*, ^4^*Rheumatology, University and IRCCS Policlinico S. Matteo Foundation, Pavia, Italy*

**Introduction:** The staging of interstitial lung disease (ILD) is important to monitor disease progression and for prognostication. A disease severity scale of Systemic Sclerosis (SSc)-related lung disease has long been proposed (i.e. Medsger’s severity scale), however it lacks specificity for ILD. This scale was mostly developed by discussion and consensus and stage thresholds were not mathematically computed. Hidden Markov models (HMM) are methods to estimate population quantities for chronic diseases with a staged interpretation which are diagnosed by markers measured at irregular intervals. HMM are thus suitable for the defining SSc-ILD lung stages from observational data.

**Material and Methods:** SSc patients (2013 criteria) at risk for or with ILD enrolled in a discovery (207 cases, Milan1) and in a validation (100 cases, Milan2, Pavia and Rome) cohort, satisfying the following inclusion criteria were studied: 1) absence of anticentromere antibodies, 2) dcSSc subset or 3) other subsets with either 3a) ILD-related antibodies (Scl70, PmScl, Ku) or 3b) HRCT evidence of ILD, 4) disease duration < 5 years at the time of the first pulmonary function test (PFT). Serial PFTs were retrieved and the time up to the last available visit -if the patient alive-, or to death due to pulmonary complications, was recorded. SSc-ILD-related mortality was modelled with HMM considering Medsger’s classes or a 3-stage model (normal/mild, moderate, severe) of functional values (minimum of FVC and DLco). The explained residual variation (R2) of prediction errors (the higher the better), was used to evaluate predictions from the training model.

**Results:** Patients were followed for 11±5.6 and 7.4±5.7 years in the two sets with a crude mortality of 13% and 12%, respectively. Fifteen-years survival estimates for Mesdger’s classes were: normal=0.88, mild=0.86, moderate=0.84 and severe=0.71; 15-years survival estimates for the 3-stage model were: normal/mild=0.89, moderate=0.82 and severe=0.63. R2 values at 15 years in the training and testing sets were equal to 0.25 and 0.16 for Medsger’s classes and to 0.31 and 0.23 for the 3-stage model, demonstrating a better prognostic capability of the 3-stage model. The thresholds in the model derived from our data were: normal/mild, FVC and DLco >=75%, moderate: 74-55%, severe: <55%.

**Conclusions:** A simplified 3-stage functional model of SSc-ILD yields better survival estimates and long-term prognostic information than Medsger’s classes in patients at risk for or with ILD. This model is simple and reproducible for the use in the daily practice or in clinical trials.

## P.085

## PREVALENCE AND CLINICAL PRESENTATION OF SSC-ASSOCIATED ILD ACCORDING TO WORLDWIDE SPATIAL REPARTITION IN THE EUSTAR DATABASE

A. Lescoat^1^, D. Huscher^2^, E. Hachulla^3^, C.P. Denton^4^, M. Matucci-Cerinic^5^, G. Riemekasten^6^, F. Del Galdo^7^, C. Caimmi^8^, M.-E. Truchetet^9^, N. Schoof^10^, O. Distler^11^, Y. Allanore^12^

^1^*CHU Rennes, Internal medicine and clinical immunology and IRSET UMR 1085, Rennes, France*, ^2^*Institute of Biostatistics and Clinical Epidemiology, Charité Universitätsmedizin Berlin and Berlin Institute of Health, Berlin, Germany*, ^3^*CHRU-Lille, Internal Medicine and Clinical Immunology, Lille, France*, ^4^*Department of Rheumatology, University College London, Royal Free Hospital, London, United Kingdom*, ^5^*Department of Experimental and Clinical Medicine, Division of Rheumatology, University of Florence, Florence, Italy*, ^6^*Department of Rheumatology and Clinical Immunology, Justus Liebig Universitat Giessen, Bad Nauheim, Germany*, ^7^*Leeds Biomedical Research Centre and Leeds Institute of Rheumatic and Musculoskeletal Medicine, Leeds, United Kingdom*, ^8^*Rheumatology Unit, University of Verona, Verona, Italy*, ^9^*Rheumatology Department, Bordeaux University Hospital, Bordeaux, France*, ^10^*Boehringer Ingelheim GmbH, Ingelheim, Germany*, ^11^*Department of Rheumatology, University Hospital Zurich, Zurich, Switzerland*, ^12^*Department of Rheumatology, Cochin Hospital, University of Paris Descartes, Paris, France*

**Introduction:** Systemic sclerosis (SSc) is a heterogeneous disease with a wide range of clinical presentations. Some SSc-associated characteristics differ between countries, ethnicities, and geographical regions. SSc-associated interstitial lung disease (SSc-ILD) is considered as the leading cause of death in SSc. Nonetheless, little is known about the direct comparison of the characteristics of SSc-ILD between geographical regions worldwide. This study aims to determine and compare the prevalence, clinical presentation, management and prognosis of SSc-ILD among predefined geographical regions.

**Material and Methods:** EUSTAR inclusion centres were located within 39 different countries that were clustered into 7 pre-defined geographical regions: “southern Europe”, “Western Europe and Nordic countries”, “Eastern Europe, Russia and Baltic countries”, “Central Europe”, “Africa and Middle East”, “America (north and south)”, “Asia and Oceania”.

**Results:** 7380 patients were included in the cross-sectional evaluation. The mean prevalence of SSc-ILD (attested by the presence of signs of lung fibrosis on HRCT and/or X-Rays, or when a date for a diagnosis of ILD was informed) was 47.9%, with high differences among regions: the region “Eastern Europe, Russia and Baltic countries” had the highest prevalence (68.1%) of SSc-ILD, whereas “Western Europe and Nordic countries” had the lowest (41.6%) (p<0.0001). FVC (%pred) was significantly lower in patients with SSc-ILD than in unaffected patients (86.6+21.6 vs 101.8 +19.6) (p<0.0001) and this remains valid in all geographical regions taken separately except in “Eastern Europe, Russia and Baltic countries” where FVC only tended to be lower in SSc-ILD patients (83.5+21.5 vs 90.0 +15.9, p=0.055). SSc-associated characteristics also differed according to regions: Scl70 antibodies were associated with SSc-ILD in all regions (p<0.001) except in America were where this association was not statistically significant (positive in 19.6% of patients without ILD and positive in 29.9% of patients with ILD, p>0.99).

Subsequent longitudinal analyses were performed on the 2389 patients with SSc-ILD and at least one follow-up visit: “Asia and Oceania” had a significantly worse survival in comparison with all other regions. “Asia and Oceania” had the lowest use of immunosuppressive therapies (7.2% ; p<0.0001 in comparison with all other regions). Regarding deaths, the highest proportion of death due to SSc was in “Africa and Middle East” (90.0%) and the lowest in “America” (57.1%).

**Conclusions:** Prevalence, severity and clinical presentation of SSc-ILD significantly varies among geographical regions worldwide. Lower use of immunosuppressive drugs seems to be associated with lower survival rate. Socio-environmental, geographical but also health system organisation factors may influence these results.

## P.086

## EVALUATION OF THE RITUXIMAB LOW DOSES EFFECT ON SYSTEMIC SCLEROSIS CLINICAL MANIFESTATIONS AND ACTIVITY

O. Koneva, L. Ananyeva, L. Garzanova, O. Desinova, O. Ovsyannikova, M. Starovoytova

*Nasonova Research Institute of Rheumatology, Moscow, Russia*

**Introduction:** Cyclophosphamide (CyP) is a drug of choice for the treatment of interstitial lung disease(ILD) in the patients with systemic sclerosis(SSc).However, according to the literature, the CyP use leads to rather limited and transient improvement of the pulmonary fibrosis. In this context the search for novel,more efficacious agents has been continued,such as attracting much attention retuximab(RTM).

Objective:To compare the impact of CyP and RTM low doses on SSc clinical manifestation and activity, and the safety of these agents in the open-label prospective non-randomized study.

**Material and Methods:** 107 patients with SSc diagnosis and ILD evidence based on HRCT findings were enrolled into the study. All patients received low-dose prednisolone regimens.36 patients(Group A) received parenteral CyP for 12±6 months at total dose 10.6±5 g(the average age 47±12 years,females 92%, SSc duration 5.0±4.8 years,d/l forms 1.6/1).71 patients(Group B) received RTM at total dose 1.43±0.66 g over the follow-up period 13.2±2 months(the average age 46±13 years,females 83%, SSc duration 5.6±4.4 years,d/l forms 1.4/1);to 32(45%) of them RTM was added to immunosupressants due to inadequate efficacy of the latter.The time courses FVC(%),modified skin count(mRss,points),activity index(EScSG,points),left ventricle ejection fraction(LVEF,%), mean pulmonary arterial pressure(EchoCG),and cardiac rhythm and conductivity disorders(ECG) were evaluated.

**Results:** In Groups A and B the therapy was associated with significant decrease in mRss(11.2±9.8 vs 7.9±6.8, p=0.009 and 11.3+9.6 vs 8.0+6.6,p=0,001,respectively) and EScSG(2.8±2 vs 1.4±1.17,p=0.000165 and 2.8+1.8 vs 1.3+1.1,p= 0.001,respectively).Increase in LVEF(61.8+7.3 vs 63.6+7.3. p=0.02) was observed only in RTM-treated patients.

Evaluation of FVC time course in Groups A and B revealed significant FVC increase(80.5±20.1 vs 85.9±20.5,p= 0.034 and 77.3+20 vs 82.6+21,p=0.000045,respectively),with median increment about 5%.

In Group A FVC 10% FVC increase was found in the third(31%) of the patients, thus exceeding respective parameter in Group B(19,7%,p=0.2).The patient percentage with FVC decrease by more 10% was similar in both groups(5,6%).

During the follow-up period no change of the other studied parameters was observed.

The therapy was better tolerated in RTM-treated group:during RTM therapy adverse reactions emerged in significantly lower proportion of the patients(11/14%) compared with CyP-treated group(19/53%),p=0,0000.

**Conclusions:** Both agents effectively alleviated skin induration and EScSG,and significantly improved FVC. However,CyP use for a year slightly more frequently resulted in clinically significant FVC increase,probably due to low RTM cumulative dose.RTM was better tolerated compared to CyP.The study findings substantiate potential use of anti-B-cell therapy both as a first-line agent for ILD treatment in the patients with SSc,and in the event of CyP inefficacy of poor tolerability,especially in the patients with cardiopathy.

## P.087

## EVALUATION OF THE RITUXIMAB MONOTHERAPY EFFICACY IN THE PATIENTS WITH SYSTEMIC SCLEROSIS ASSOCIATED WITH INTERSTITIAL LUNG DISEASE

O. Koneva, L. Ananyeva, L. Garzanova, O. Desonova, O. Ovsyannikova, M. Starovoytova

*Nasonova Research Institute of Rheumatology, Moscow, Russia*

**Introduction:** Rituximab (RTM) is considered as a promising therapeutic agent for insterstitial lung disease (ILD) treatment in the systemic sclerosis (SSc) patients. However,the limited number of RTM-treated patients, heterogeneity of the studies in relation to main parameters, considerably different dose regimens, cumulative doses, and observation periods does not allow univocal conclusions on RTM efficacy or definitive recommendations on RTM use in the patients with SSc. The question whether to combine RTM with immunosupressants(IS) or it is possible to use it as a single-agent therapy in the SSc patients with ILD is still relevant.

Objective: To compare the time courses of pulmonary function parameters and dermal fibrosis parameters during the RTM use as a single-agent therapy and in combination with IS in the SSc patients with with ILD in the open-label prospective non-randomized study.

**Material and Methods:** 90 patients with the confirmed SSc diagnosis and ILD evidence based on MSCT were enrolled into the study. All patients received low- and moderate-dose glucocorticoids regimens. Group A(n=45) received RTM as a single therapy agent in a total dose 2.7±1g(average age 45.0±15 years,female proportion 82%,SSc duration 6.7±5.6 years,diffused/limited forms 1.5/1). Group B(n=45) received a total RTM dose 3.1±1.2g in combination with IS(27/60% mycophenolate mofetyl, 16/35.6% cyclophosphamide, 2/4.4% methotrexate; the patient’s average age was 47.4±11.6 years, with female proportion 82%; SSc duration 4.6±3.5 years; diffused/limited forms 1.3/1). The age, gender proportion, SSc form, forced vital capacity(FVC), diffusive lung capacity(DLCO), and RTM cumulative doses were similar in the both groups. The follow-up period was 42 months. The time courses of FVC, DLCO, modified skin count(mRss), activity index(EScSG) were assessed in the study.

**Results:** In Groups A and B during the therapy significant decrease in mRss (11.5±9.3 vs 5.6±4.2 p=0.000002 and 11.0±9.3 vs 7.2±5.6, p=0.00034, respectively) and EScSG(2.7±1.6 vs 1.2±0.98, p=0.000000 and 3.2±1.9 vs 1.6±1.3, p=0.00011, respectively),FVC increase(77.8±19.7 vs 86.8±19.7, p=0.00001 and 76±20.3 vs 82.7±22.5, p=0.00017, respectively), and DLCO stabilization were observed.

The treatment groups did not differ significantly in the median FVC increment, clinically meaningful FVC and DLCO increments of decrements, and mRss and EScSG time courses.

**Conclusions:** RTM administration both in combination with IS and as a single agent therapy in the SSc patients with ILD effectively alleviated skin induration and SSc activity, improved or stabilized the pulmonary function parameters. The absence of statistically significant difference in the time course of evaluated parameters between the groups substantiate potential RTM use as a single-agent therapy. This is most important for the patients with poor tolerability or contraindications to IS administration.

## P.088

## USE OF IMMUNOSUPPRESSANTS AND CLINICAL OUTCOMES AMONG PATIENTS WITH SYSTEMIC SCLEROSIS AND SYSTEMIC SCLEROSIS-ASSOCIATED INTERSTITIAL LUNG DISEASE: A US COHORT STUDY

Q. Li^1^, L. Wallace^2^, P. Patnaik^2^, M. Alves^3^, M. Gahlemann^4^, V. Kohlbrenner^2^, C. Raabe^5^, J.R. Wang^6^, L.M. Garry^6^

^1^*Boehringer Ingelheim (China) Investment Co. Ltd - Global Epidemiology, Shanghai, China*, ^2^*Boehringer Ingelheim Pharmaceuticals Inc. - Global Epidemiology and Real World Evidence, Ridgefield, USA*, ^3^*Boehringer Ingelheim International GmbH - TA Inflammation Med, Ingelheim am Rhein, Germany*, ^4^*Boehringer Ingelheim (Schweiz) GmbH, Basel, Switzerland*, ^5^*Boehringer Ingelheim International GmbH - Real World Data Analytics Center of Excellence, Ingelheim am Rhein, Germany*, ^6^*Aetion, Inc. - Science, Boston, USA*

**Introduction:** This cohort study investigated the use of immunosuppressants (IS) and incident clinical outcomes among patients with Systemic Sclerosis (SSc) and SSc-associated interstitial lung disease (SSc-ILD).

**Material and Methods:** We used IBM Marketscan (2008–17) US claims to identify an SSc cohort of patients aged >=18 years with >=1 incident SSc diagnostic claim (365-day washout) preceded by >=365 days of enrolment. Patients who additionally had an ILD claim within 365 days of SSc diagnosis were selected into the SSc-ILD cohort. Initiation of IS during the follow-up period was collected and dose escalation was defined as a change in IS type or increase in dose following stable regimen (no change for >=6 months). We performed descriptive analyses of IS treatment course and outcomes during follow-up using Aetion Evidence PlatformTM (v3.12).

**Results:** When the overall cohort of SSc-ILD patients (N=8,252) was compared to SSc patients (N=34,820), analyses of incident outcomes per 100,000 person-years reported similar rates of skin disorders (2.33 vs 2.03), but increased rates of gastroesophageal reflux (2.26 vs 1.44), arterial hypertension (1.11 vs 0.61), Raynaud syndrome (0.99 vs 0.68) and chronic obstructive pulmonary disease (1.08 vs 0.42).

Among IS-naïve SSc and SSc-ILD patients (30,088 and 6,320 patients), 8.2% and 12.7% had a new prescription claim for IS within a median of 145 and 115 days after diagnosis. The most frequently initiated IS was methotrexate for SSc (48.4% vs 23.4% for SSc-ILD) and mycophenolate mofetil (MMF) for SSc-ILD patients (31.3% for SSc vs 52.6%). Among those with >=180 days of follow-up (2,002 and 653 patients), 42.5% and 45.0% started a stable regimen within a median of 88 and 87 days after IS initiation. Among those who achieved stable regimen (850 and 294 patients), 21.6% and 19.4% had an escalation after a median duration of 289 and 292 days of stable use. Among patients with sufficient enrolment to determine a subsequent stable regimen (141 and 44 patients), 61.0% and 65.9% were able to achieve stable regimen for a median duration of 397 and 365 days. The median time between start of first and subsequent stable regimen was 436 and 451 days.

**Conclusions:** In this large database, SSc-ILD patients were more likely than SSc patients to be treated with MMF, and had a higher incidence rate of comorbid outcomes over an average of ~2 years’ follow-up. Time to initiation of first stable IS was ~1 month sooner for SSc-ILD than SSc patients, though duration of stable IS was similar.

## P.089

## DISEASE FREQUENCY AND CHARACTERISTICS OF PATIENTS WITH SYSTEMIC SCLEROSIS AND SYSTEMIC SCLEROSIS-ASSOCIATED INTERSTITIAL LUNG DISEASE: A US COHORT STUDY

Q. Li^1^, L. Wallace^2^, P. Patnaik^2^, M. Alves^3^, M. Gahlemann^4^, V. Kohlbrenner^2^, C. Raabe^5^, J.R. Wang^6^, E.M. Garry^6^

^1^*Boehringer Ingelheim (China) Investment Co. Ltd - Global Epidemiology, Shanghai, China*, ^2^*Boehringer Ingelheim Pharmaceuticals Inc. - Global Epidemiology and Real World Evidence, Ridgefield, USA*, ^3^*Boehringer Ingelheim International GmbH - TA Inflammation Med, Ingelheim am Rhein, Germany*, ^4^*Boehringer Ingelheim (Schweiz) GmbH, Basel, Switzerland*, ^5^*Boehringer Ingelheim International GmbH - Real World Data Analytics Center of Excellence, Ingelheim am Rhein, Germany*, ^6^*Aetion, Inc. - Science, Boston, USA*

**Introduction:** Interstitial lung disease (ILD) is the leading cause of death in Systemic Sclerosis (SSc). In this cohort study, we investigated the prevalence and incidence rate (IR) of SSc and SSc-associated ILD, and described patient characteristics at incident diagnosis.

**Material and Methods:** We used IBM Marketscan (2008–17) US claims to identify all patients from 2009–2017 aged >=18 years with >=365 days of enrolment, and included patients with >=1 diagnostic claim for SSc. Patients who additionally had an ILD claim within 365 days prior to, on, or after SSc diagnosis were included as SSc-ILD. Sensitivity analyses further required two SSc and two ILD claims, using the latter claim as the diagnosis date. Incident cases were defined using a 365-day washout. Prevalence included both incident and existing cases. Analyses were performed using Aetion Evidence Platform™ (v3.12).

**Results:** Among all patients at risk (N=78,964,708), the prevalence of SSc and SSc-ILD per 100,000 persons was 72.09 and 19.00, respectively; it was higher among females (115.84 and 30.20) than males (23.98 and 6.69). There were 34,820 patients with incident SSc and 8,252 with incident SSc-ILD overall (18.28 and 4.33 per 100,000 patient-years [PY]); rates were higher among females (28.98 and 6.71) than males (6.32 and 1.67). When we required two claims, prevalence was 41.45 and 9.84 per 100,000 persons, and the IR was 8.83 and 1.64 per 100,000 PY.

Incident cohorts of SSc and SSc-ILD patients were largely female (83.7% and 81.8%) with mean ages of 53.6 and 58.3 years. Comorbidities per 100,000 persons within the 365-day baseline period were higher among SSc-ILD patients compared with SSc patients, including skin disorders (3.45 vs 2.88), gastroesophageal reflux disease (2.85 vs 1.79), chronic obstructive pulmonary disease (2.07 vs 0.76), Raynaud syndrome (1.45 vs 0.78), arterial hypertension (1.38 vs 0.63), acute respiratory failure (0.51 vs 0.15), pulmonary hypertension (1.12 vs 0.32) and pulmonary arterial hypertension (0.05 vs 0.01). Further, SSc-ILD patients had more diagnostic procedures during baseline, including spirometry (43.9% vs 15.2%), chest X-ray (64.2% vs 34.9%) and chest computed tomography (44.3% vs 13.4%).

**Conclusions:** The high prevalence and IR of SSc in this large database analysis may be attributed to the methodology and was reduced when using two claims for diagnosis. The prevalence and IR of SSc and SSc-ILD were higher among females than males. SSc-ILD patients were older and had a higher burden of disease than SSc, as reflected by the increase in comorbidities and diagnostic procedures.

## P.090

## EFFECT OF NINTEDANIB ON PROGRESSION OF SYSTEMIC SCLEROSIS-ASSOCIATED INTERSTITIAL LUNG DISEASE (SSC-ILD): FURTHER ANALYSES OF THE SENSCIS TRIAL

D. Khanna^1^, D.E. Furst^2^, M. Aringer^3^, M. Cutolo^4^, I. Castellvi^5^, T. Luckhardt^6^, C. Stock^7^, M. Alves^8^, M. Gahlemann^9^, O. Distler^10^

^1^*Department of Medicine, University of Michigan, Ann Arbor, Michigan, USA*, ^2^*Department of Medicine, David Geffen School of Medicine at University of California, Los Angeles, California, USA*, ^3^*Division of Rheumatology, Department of Medicine III, University Medical Center & Faculty of Medicine Carl Gustav Carus, TU Dresden, Dresden, Germany*, ^4^*Research Laboratory and Academic Unit of Clinical Rheumatology, Department of Internal Medicine, University of Genova, Genova, Italy*, ^5^*Department of Rheumatology, Hospital de la Santa Creu i Sant Pau, Barcelona, Spain*, ^6^*Department of Medicine, University of Alabama at Birmingham, Birmingham, Alabama, USA*, ^7^*Boehringer Ingelheim Pharma GmbH & Co. KG, Biberach, Germany*, ^8^*Boehringer Ingelheim International GmbH, Ingelheim am Rhein, Germany*, ^9^*Boehringer Ingelheim (Schweiz) GmbH, Basel, Switzerland*, ^10^*Department of Rheumatology, University Hospital Zurich, Zurich, Switzerland*

**Introduction:** In the SENSCIS trial, patients with SSc-ILD were randomised to receive nintedanib or placebo until the last patient reached week 52 but for no longer than 100 weeks. Over 52 weeks, nintedanib reduced the rate of decline in FVC (mL/year) by 44% vs placebo. We assessed the effect of nintedanib on categorical changes in FVC % predicted and other measures of disease progression in the SENSCIS trial.

**Material and Methods:** In post-hoc analyses, we assessed the proportions of patients with absolute declines or increases in FVC % predicted at week 52 using a worst observation carried forward approach. We also assessed i) the time to an absolute decline in FVC >=10% predicted or death and ii) the time to an absolute decline in FVC >=10% predicted, absolute decline in FVC >=5 to <10% predicted with an absolute decline in DLco >=15% predicted, or death, both over 52 weeks ± 7 days.

**Results:** At baseline, mean (SD) FVC was 72.4 (16.8) % predicted in the nintedanib group (n=288) and 72.7 (16.6) % predicted in the placebo group (n=288). Over 52 weeks, in the nintedanib and placebo groups, an absolute decline in FVC >5% predicted occurred in 20.6% and 28.5% of patients (OR 0.65 [95% CI 0.44, 0.96]; p=0.03) and an absolute decline in FVC >10% predicted occurred in 7.0% and 8.3% of patients (OR 0.82 [0.44, 1.52]; p=0.53), respectively (Figure). An absolute decline in FVC >=10% predicted or death occurred in 13.9% and 21.5% of patients in these groups, respectively (HR 0.63 [0.42, 0.93]; p=0.02). An absolute decline in FVC >=10% predicted, absolute decline in FVC >=5 to <10% predicted with an absolute decline in DLco >=15% predicted, or death occurred in 15.9% and 24.0% of patients in the nintedanib and placebo groups, respectively (HR 0.63 [0.43, 0.92]; p=0.02).

**Conclusions:** In the SENSCIS trial, treatment with nintedanib was associated with a significantly lower chance of decline in FVC >5% predicted and of an absolute decline in FVC >=10% predicted or death over 52 weeks compared with placebo. These results support a clinically meaningful effect of nintedanib on ILD progression in patients with SSc-ILD.

**Figure:**
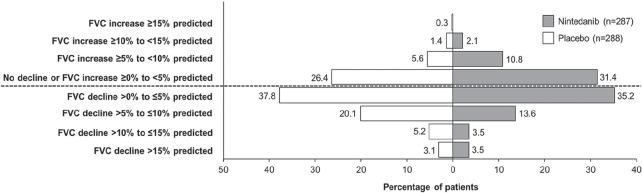


## P.091

## EVIDENCE-BASED CONSENSUS STATEMENTS FOR THE IDENTIFICATION AND MANAGEMENT OF INTERSTITIAL LUNG DISEASE IN SYSTEMIC SCLEROSIS

A. Hoffmann-Vold^1^, T.M. Maher^2,3^, A. Ashrafzadeh^4^, O. Distler^5^

^1^*Department of Rheumatology, Oslo University Hospital, Oslo, Norway*, ^2^*National Heart & Lung Institute, Imperial College London, London, United Kingdom*, ^3^*Interstitial Lung Disease Unit, Royal Brompton Hospital, London, United Kingdom*, ^4^*Rheumatology Center of Excellence, IQVIA, San Diego, USA*, ^5^*Department of Rheumatology, University Hospital Zurich, Zurich, Switzerland*

**Introduction:** Interstitial lung disease in systemic sclerosis (SSc-ILD) occurs frequently and carries a high mortality risk; expert guidance is needed to aid early recognition and treatment. We aimed to gain the first expert consensus for the identification and management of SSc-ILD applying a state of the art methodology.

**Material and Methods:** Using a robust, modified Delphi process based on a systematic literature analysis (of articles published between January 1992-April 2018), evidence-based consensus statements for SSc-ILD management were established for the following topics: risk factors (including biomarkers); screening; diagnosis and severity assessment; treatment initiation and options; disease progression; and treatment escalation. An expert panel, including 27 pulmonologists, rheumatologists and internists with expertise in SSc-ILD, participated in three rounds of online surveys, a face-to-face discussion and a WebEx meeting to establish consensus. Consensus was considered achieved if >=80% of panellists indicated their agreement or disagreement with proposed statements. A supplemental Delphi process was carried out to incorporate two further rounds of voting on statements concerning treatment, following recently published/presented data (since May 2018) on potential therapies for SSc-ILD.

**Results:** Following the initial Delphi process, consensus statements were agreed by the panel across six topics (Table). Following the supplemental Delphi process, the panel agreed that nintedanib (as monotherapy or in combination with mycophenolate mofetil) may be an effective option for treatment initiation or escalation, subject to licensed availability.

**Conclusions:** Through a robust modified Delphi process developed by a diverse panel of experts, the first evidence-based consensus statements were established on guidance for the identification and medical management of SSc-ILD.

**Funding:** Boehringer Ingelheim International GmbH, Germany

**Figure:**
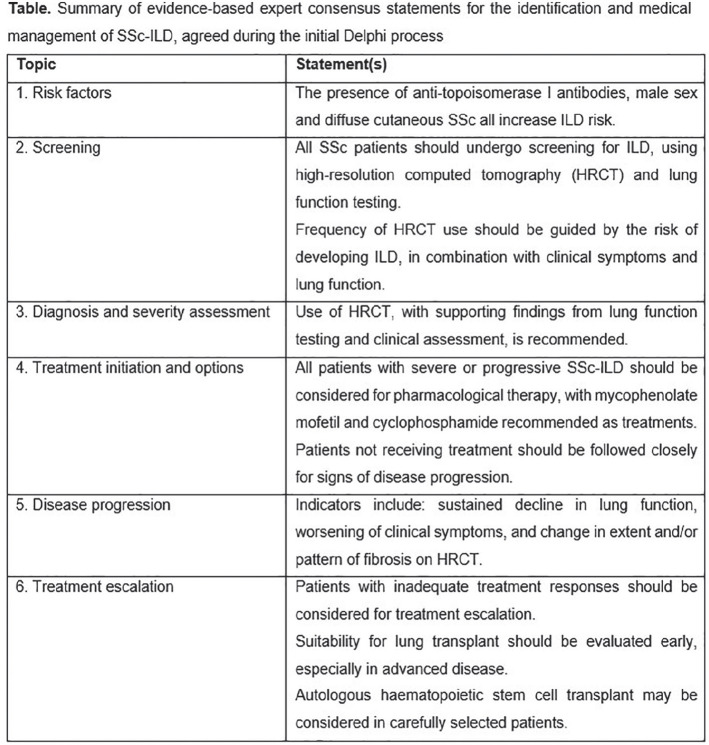


## P.092

## PREVENTION OF INTERSTITIAL LUNG DISEASE WITH IMMUNOSUPPRESSIVE THERAPY IN SYSTEMIC SCLEROSIS

S. Hoa^1^, S. Bernatsky^2^, S. Proudman^3^, W. Stevens^4^, J. Sahhar^5^, M. Wang^6^, R.J. Steele^7^, M. Baron^8^, M. Nikpour^4^, M. Hudson^8^

^1^*Division of Rheumatology, Department of Medicine, Centre Hospitalier de l’Universite de Montreal, University of Montreal, Montreal, Canada*, ^2^*Division of Rheumatology, Department of Medicine, McGill University Health Centre, McGill University, Montreal, Canada*, ^3^*Rheumatology Unit, Department of Medicine, Royal Adelaide Hospital, University of Adelaide, Adelaide, Australia*, ^4^*Department of Rheumatology, St. Vincent’s Hospital, University of Melbourne, Fitzroy, Australia*, ^5^*Department of Rheumatology, Monash Health, Clayton, Australia*, ^6^*Lady Davis Institute, Jewish General Hospital, Montreal, Canada*, ^7^*Department of Mathematics and Statistics, McGill University, Montreal, Canada*, ^8^*Division of Rheumatology, Department of Medicine, Jewish General Hospital, McGill University, Montreal, Canada*

**Introduction:** Interstitial lung disease (ILD) is a leading cause of mortality in systemic sclerosis (SSc). Immunosuppressive drugs are used to treat established SSc-ILD. However, little is known about whether immunosuppression might prevent ILD in SSc. The aim of this study was to determine if, in SSc patients without ILD, immunosuppression (given for non-pulmonary manifestations) was associated with a lower risk of incident SSc-ILD.

**Material and Methods:** A retrospective cohort of SSc subjects without ILD at baseline and at least one follow-up was constructed from the Canadian Scleroderma Research Group and the Australian Scleroderma Interest Group registries. The primary exposure of interest was any use of cyclophosphamide, mycophenolate, methotrexate and/or azathioprine. Time to new onset ILD was compared between exposed and unexposed subjects, using a marginal structural Cox model incorporating inverse probability of treatment weights to address potential time-varying confounding. Multiple imputation was used to account for missing data. Weights were constructed using variables most likely to influence a decision to initiate immunosuppression, namely age, sex, race, disease duration and subtype, anti-centromere (ACA), anti-topoisomerase I (ATA), anti-RNA polymerase III (ARNAP) and anti-Ro52 autoantibody status, modified Rodnan skin scores, forced vital capacity (FVC), presence of inflammatory arthritis and myositis, and disease activity scores.

**Results:** A total of 1,587 subjects met the inclusion criteria. Mean (SD) age and disease duration at cohort entry were 56.7 (12.4) and 11.7 (9.7) years. The cohort was followed for a mean (SD) of 4.7 (2.9) years. During this time, 204 subjects were newly diagnosed with ILD, corresponding to a crude incidence of 31.5 (95% CI, 27.0 to 36.3) per 1,000 person-years. 872 (15.6%) person-visits were exposed to immunosuppressive drugs (70% methotrexate, 26% mycophenolate, 9% azathioprine, 2% cyclophosphamide). Exposed person-visits were younger, more likely to be male, non-white, with diffuse disease and shorter disease duration, and had more ATA and ARNAP and less ACA positivity, more arthritis and myositis, higher disease activity scores and lower FVC. The marginal structural Cox analysis produced a weighted adjusted HR of 0.68 (95%, CI 0.43 to 1.08) for incident ILD in subjects exposed to immunosuppression compared to unexposed subjects.

**Conclusions:** On average, exposure to immunosuppressive drugs decreased the risk of incident SSc-ILD by over 30%, although uncertainty remains around this estimate. Early treatment of SSc before clinical detection of ILD may represent a window of opportunity for preventive intervention. The role of immunosuppressive drugs in mitigating SSc-ILD risk should be explored in prospective studies, including controlled trials.

## P.093

## ANALYSIS OF SYSTEMIC SCLEROSIS LUNG DISEASE WITH CELLPHONEDB

M. Hinchcliff, I. Odell

*Yale School of Medicine, New Haven, USA*

**Introduction:** Interstitial lung disease (ILD) is a leading cause of death in patients with systemic sclerosis (SSc). Several groups have applied single-cell RNA sequencing to lung tissue from patients with SSc and healthy donors at the time of lung transplantation to shed light on the altered signaling events in specific cell types that drive SSc-ILD. However, single-cell RNA sequencing generates complex data that can defy attempts to understand pathobiology. Recently CellphoneDB was created to identify cell to cell interactions (e.g. ligand-receptor protein complexes, and extracellular matrix proteins and proteases). We tested the hypothesis that reanalysis of single-cell RNA sequencing data using CellphoneDB can provide novel insights into SSc-ILD pathobiology.

**Material and Methods:** To examine the utility of applying CellphoneDB to single-cell RNA sequencing data, we downloaded two publicly available independent SSc-ILD datasets from the NCBI Gene Expression Omnibus. We used the Seurat v3 package for data preprocessing to filter low quality samples, followed by a standard Seurat pipeline with data normalization, scaling, PCA analysis, and clustering. The resulting normalized data and cluster identification were input into CellphoneDB v2 with statistical analysis. The interacting protein pairs in the normal and SSc gene lists were filtered by rank, excluding those with rank > 0.05 (analogous to p > 0.05). We then determined the list of interacting pairs common to SSc lung disease compared to healthy lung tissue in both datasets.

**Results:** 16 significant pairwise cell-cell interactions were identical between GEO GSE122960 and GSE128169. Biologically relevant interactions included members of pathways previously shown to be involved with SSc-ILD including Wnt7B:FZD4, BMPR1B:BMPR2:BMP5 and COL6A6:a10b1 complex as well as unexpected pathways including PLXNB2:PTN (PLXNB2 regulates Rho kinases that has been implicated in myofibroblast differentiation in SSc while PTN plays a role in nerve regeneration that may be important following lung injury) and JAG1:NOTCH3 (JAG1 is a ligand of the Notch receptor, and their binding triggers a cascade of proteolytic cleavage that leads to the release of the intracellular part of the receptor from the membrane, allowing it to translocate to the nucleus where it activates transcription factors that play key roles in cell differentiation and morphogenesis).

**Conclusions:** We discovered concordant pathways in two independent single-cell RNA sequencing SSc lung tissue datasets. Identification of previously confirmed pathways thought to underlie SSc-ILD demonstrates the validity of using CellphoneDB for analysis of complex human disease such as SSc. Further work will be performed to investigate the roles of the novel protein interactions in SSc.

## P.094

## A THIRTEEN-YEAR SINGLE-CENTER EXPERIENCE IN SYSTEMIC SCLEROSIS AND LUNG TRANSPLANTATION

P. Gubern Prieto^1^, A. Guillén-Del-Castillo^1^, C. Berastegui García^2^, A. Román-Broto^2^, C. Bravo-Masgoret^2^, M. López-Meseguer^2^, I. Bello-Rodríguez^3^, E.L. Callejas-Moraga^4^, I. Sanz-Pérez^1^, M. Roca-Herrera^1^, V. Fonollosa-Pla^1^, C.P. Simeón-Aznar^1^

^1^*Hospital Universitario Vall d’Hebron - Internal Medicine - Department of Autoimmune Diseases, Barcelona, Spain*, ^2^*Hospital Universitario Vall d’Hebron - Pneumology - Department of Lung Transplantation, Barcelona, Spain*, ^3^*Hospital Universitario Vall d’Hebron - Thoracic Surgery - Department of Lung Transplantation, Barcelona, Spain*, ^4^*Consorcio Corporación Sanitaria Parc Taulí de Sabadell - Internal Medicine - Department of Autoimmune Diseases, Sabadell, Spain*

**Introduction:** Currently, cardiopulmonary disease is the main cause of death in patients diagnosed with systemic sclerosis (SSc). In cases where medical treatment fails, lung transplantation (LT) has emerged as an alternative.

The objective of this study was to describe the clinical characteristics of a patient cohort that had received LT for SSc, as well as, explaining their post transplant evolution focusing on different types of complications and causes of death.

**Material and Methods:** All patients diagnosed with SSc in a single tertiary hospital centre from Jun 2006 to Feb 2019 were included. Amongst which, 19 LT recipients were identified and their clinical data were analysed retrospectively from electronic medical records.

**Results:** Thirteen out of nineteen of the LT patients were women, the mean age at transplant was 51.6 years old. The majority of the patients (9, 47.4%) were diagnosed with diffuse cutaneous SSc and 42.1% with limited cutaneous SSc. In 89% of the cases, the transplant indication was intersticial lung disease, associated with pulmonary hypertension in 9 cases. Fifteen patients underwent bilateral LT and 4 required extracorporeal circulation. The main complications in the immediate post transplantation period were surgical: 9 cases of gastric palsy and 4 of diaphragmatic palsy. Gastroparesia was transitory in most cases, although 2 patients required nutrition through percutaneous endoscopic gastrostomy. In 63% there was worsening of gastroesophageal reflux disease (GERD) and among them, 4 patients presented with bronchoaspiration after transplant. In most patients symptoms were controlled with medical treatment and none required further surgery. In regard to immunological complications: 4 patients developed primary graft dysfunction, 9 acute cellular rejection and 3 chronic lung allograft dysfunction. Malignancies were found in 5 patients, but most were cutaneous skin neoplasms. Within the subgroup of less frequent complications, one case of scleroderma renal crisis induced by corticosteroids and tacrolimus therapy and one case of sirolimus-induced pulmonary alveolar proteinosis were reported.

The mean of follow-up was 70 months, only one patient required home oxygen therapy. Nine out of the 19 LT patients died. Infection was the most common cause of death. The cumulative survival proportion was 74%, 68%, 60% and 60% at 12, 36, 60 and 84 months, respectively.

**Conclusions:** Lung transplant has proved to be a useful therapy for pulmonary SSc-associated disease refractory to medical treatment. Based on the data presented, GERD does not seem to affect outcomes of LT. However, accurately selecting appropriate candidates among SSc patients is essential for ameliorating short- and long-term results.

## P.095

## THE ROLE OF NAILFOLD CAPILLAROSCOPY IN MONITORING LUNG INVOLVEMENT IN SYSTEMIC SCLEROSIS

L. Groseanu^1^, P. Paraschiva^2^, A. Balanescu^1,2^, V. Bojinca^1,2^, D. Opris-Belinski^1,2^, A. Borangiu^1,2^, I. Saulescu^1,2^, D. Mazilu^1,2^, S. Daia-Iliescu^1,2^, F. Berghea^1,2^, C. Constantinescu^1,2^, M.-M. Negru^1,2^, M. Abobului^1,2^, C. Cobilinschi^1,2^, R. Ionescu^1,2^

^1^*Sf Maria Clinical Hospital- Departemnt of Rheumatology and Internal Medicine, Bucharest, Romania*, ^2^*Carol Davila University of Medicine and Pharmacy, Bucharest, Romania*

**Introduction:** The usefulness of capillaroscopy in the follow-up of scleroderma patients and the possible prognostic role for the appearance of visceral involvement is suggested by many authors but still under debate.The aim of this study was to assess the role of monitoring capillaroscopic abnormalities (qualitative and semiquantitative) in relation with parameters of interstitial lung involvement (ILD) and pulmonary arterial hypertension(PAH).

**Material and Methods:** We conducted a longitudinal prospective study that included 118 SSc patients monitored between 2013-2017. All patients had a demographic,clinical, laboratory and instrumental assessment according to MEDS evaluation sheets. SSc patients was performed by a trained rheumatologist using a videocapillaroscope with a X200 magnification probe according to the standard method: qualitative and semiquatitative. The microangiopathy evolution score (MES) was selected to assess the progression of the vascular damage.

**Results:** The study group included 118 patients, 103 females (87,29%), 63(53,39%) had a diffuse extend of skin involvement, mean age was 50.58(12,71) years, mean disease duration at first NVC evaluation 2,81(4,2) years.

43,22% of the patients had lung involvement at the first evaluation: 33,89% had ILD, 18,64% had PAH. 9,32% of the patients had both ILD and PAH.Patients with ILD or PAH had higher MES than patiens without ILD or PAH: 5,58(1,35) vs 4,35 (1,28), p<0,001, 5,64(1,09) vs 4,56(1,42), p<0,001. 10 more patients (8,47%) had new lung involvement at the second evaluation : 42,37% ILD, 24,42% PAH; 15,25% of the patients had both ILD and PAH.23 patients had an increase of one step of the NVC pattern, 2 patients with 2 steps. Patients with ILD had higher MES scores than those without ILD (6,68(1,53) vs 5,29(1,65), p<0,001). Patients with PAH had higher MES than those without PAH (7(1,5) vs 5,591,65), p<0,001).

A strong correlation was identified between initial capillaroscopy scores and FVC (r=-.47, p=0.002), DLCO (r=-.51, p< 0.001) and sPAP (r=0.34, p<0.001). Active and late capillaroscopic pattern were correlated with diagnosis of lung fibrosis (chi2=14, p=0.007) and PAH at follow-up examinations (chi2=14,2, p=0.007). Progression of capillaroscopic pattern at follow-up evaluations was not correlated with significant worsening of lung volumes, DLCO, sPAP. Instead, progression of microangiopathy evolution score (>1) was asociated with worsening of FVC (r=0.32,p<0.001), DLCO (r=0.21,p=0.02) and new diagnosis of lung fibrosis on HRCT (r=0,19,p=0.035).

**Conclusions:** Semiquantitative scoring, rather then qualitative capillaroscopic assessment can have a predictive role for new involvement or worsening of previous lung involvement (especially interstitial lung disease) in scleroderma patients, confirming the putative role of capillaroscopy as biomarker in SSc.

## P.096

## CALIPER: EVALUATION OF INTERSTITIAL LUNG DISEASE IN SYSTEMIC SCLEROSIS

G. Leodori^1^, C. Pellicano^1^, A.L. Villa^1^, A. Iacolare^1^, A. Gigante^1^, A.M. Ferrazza^2^, M.L. Gasperini^1^, I. Carbone^2^, E. Rosato^1^

^1^*Department of Translational and Precision Medicine, Sapienza University of Rome, Rome, Italy*, ^2^*Department of Radiological, Oncological and Pathological Sciences, Rome, Italy*

**Introduction:** Interstitial lung disease (ILD) represents a major cause of morbidity and mortality in systemic sclerosis (SSc). Up to 90% of SSc patients present radiological abnormalities and in 40–75% of cases will show restrictive disorders in pulmonary function tests (PFTs). CALIPER (Computer Aided Lung Informatics for Pathology Evaluation and Rating) is a computational platform for the near-real-time characterization and quantification of lung parenchymal patterns on computed tomography (CT) scans. The aim of this study is to characterize and quantify at baseline lung disease in scleroderma related ILD with CALIPER and to evaluate at 12 months of follow-up which radiological pattern is predictive of the worsening of lung function.

**Material and Methods:** Sixty six SSc patients, according to American College of Rheumatology/European League Against Rheumatism criteria were enrolled. All high-resolution computed tomography (HRCT) examinations were performed according to standard protocol. CT images has been processed by CALIPER. Pulmonary function test (PFTs) were performed at baseline and after 12 months of follow-up.

**Results:** In all SSc patient at baseline the mean value of PFTs are: FEV1 91,7±19%, FVC 95,3±20,6 and DLCO 68±18,8%. The CALIPER analysis highlighted: normal lung parenchyma 87,4±9,8%, ground glass 2,8±5,3%, reticular 4±5,7% and honeycombing 1±1%.

At baseline FEV1 (p<0,0001) and FVC (p=0,001) had a negative correlation with percentage of reticular pattern according to CALIPER analysis, respectively (r=-0,57, beta coefficient=-0,56) and (r=-0,55, beta coefficient=-0,48). Also DLCO showed a negative correlation (p<0,0001) with percentage of reticular pattern according to CALIPER analysis (r=-0,58, beta coefficient=-0,75).

After 12 months (T1) of follow-up PFTs showed: FEV1 90,5±19,5% with Delta of 1,2±17,9%, FVC 90,8±21,3% with Delta of 4,5±9,4% and DLCO 67,2±20,2 with Delta of 0,9±10,2.

Delta DLCO showed a positive correlation (p<0,001) with percentage of ground glass pattern (r=0,33, beta coefficient=0,51) according to CALIPER analysis.

In the ROC curve analysis ground glass score is a good predictor (0,75, p=0,009; 95% CI 0,59-0,91) of DLCO worsening, defined as a decrease of more than 10% of DLCO. Using a cut off > 4,5 for ground glass score the RR for DLCO worsening is 6,8 (p<0,01; 95% CI 1,6-29,2).

**Conclusions:** Ground glass score is the main radiological finding in order to predict the worsening of pulmonary function assessed by PFTs, defined as a decrease of more than 10% of DLCO.

## P.097

## SELF-REPORTED SYSTEMIC SCLEROSIS-RELATED AND PULMONARY SYMPTOMS ARE MORE PREVALENT IN SUBJECTS WITH RAYNAUD’S IN THE GENERAL POPULATION

A. Eman Abdulle^1^, K. de Leeuw^2^, L. Brouwer^2^, S. Arends^2^, C.T. Gan^3^, A. Herrick^4^, H. van Goor^5^, U. Mulder^1^

^1^*Department of internal medicine, University Medical Center Groningen, Groningen, The Netherlands*, ^2^*Department of rheumatology, University Medical Center Groningen, Groningen, The Netherlands*, ^3^*Department of Pulmonology, University Medical Center Groningen, Groningen, The Netherlands*, ^4^*Division of Musculoskeletal & Dermatological Sciences, The University of Manchester, Manchester, United Kingdom*, ^5^*Department of Pathology and Medical Biology, University Medical Center Groningen, Groningen, The Netherlands*

**Introduction:** Puffy fingers and Raynaud’s phenomenon (RP) are considered important predictors of early systemic sclerosis (SSc). Pulmonary involvement may occur in early SSc, and may often be irreversible. We aim to assess the prevalence of SSc-related symptoms, and explore the prevalence of pulmonary problems in subjects with RP.

**Material and Methods:** We analysed data from a large population-based cohort study. Participants with known connective tissue disease (CTD) were excluded. Patient characteristics, self-reported pulmonary problems, and spirometry were obtained. The CTD questionnaire provided information on SSc-related symptoms (i.e., RP, puffy fingers, distal/proximal skin thickening, and finger-tip lesions). Participants were categorized into definite RP (n=3911) and non-RP (n=88694).

**Results:** In the total cohort (n=92605), the prevalence of at least one SSc-related symptom (other than RP) was 8.7%. Prevalence of SSc-related symptoms was 23.5% in subjects with RP, and 7.1% without RP (p<0.001). Distal skin thickening was the most prevalent symptom in both groups. Participants with RP and SSc-related symptoms more frequently reported dyspnoea at rest, dyspnoea after exertion, pulmonary fibrosis, and had the lowest mean forced vital capacity as compared to the other groups (all p<0.001). In multivariate regression, dyspnoea at rest/on exertion remained associated with increased risk of SSc-related symptoms in subjects with RP (both p<0.001).

**Conclusions:** Prevalence of SSc-related symptoms was approximately three-fold higher in subjects with RP. Pulmonary problems are more prevalent in subjects with RP who also reported SSc-related symptoms. These patient-reported data suggest that (suspected) early SSc develops more insidiously than acknowledged, warranting further research on early detection.

## P.098

## CIRCULATING BIOMARKERS IN INTERSTIAL LUNG DISEASE IN SYSTEMIC SCLEROSIS: A META-ANALYSIS

M. Elhai, J. Avouac, Y. Allanore

*Cochin Hospital, Department of Rheumatology, Paris, France*

**Introduction:** Interstitial lung disease (ILD) is the main cause of death in systemic sclerosis (SSc). Management of SSc-ILD is challenging because individual prognosis is unpredictable. With the recent development of new and effective treatments for SSc-ILD, it is critical to identify patients with lung disease at an earlier stage and to rapidly identify those who will progress to extensive lung disease. To better delineate the input and pitfalls of biomarkers in SSc-ILD, we performed a systematic literature review followed by a meta-analysis.

**Material and Methods:** We performed a systematic literature review searching in MEDLINE and Embase databases from January 1960 to February 2019. Eligible studies were those (i) reporting the use of biomarkers in SSc-ILD, this latter being defined by high-resolution computed tomography and (ii) controlled. We focused on circulating biomarkers as having the highest generalizability. Eligibility of references retrieved by the search was assessed independently by two authors (Y.A. and M.E.) and disagreements resolved at each step. Data were extracted from the selected studies using a predefined standardized form. We calculated an odd ratio (OR) based on the number of patients with concentration of the studied biomarker above the cut-off value for SSc-ILD diagnosis or progression compared to the number of patients with concentration of the studied biomarker below the cut-off value.

**Results:** A total of 8 studies were included in the meta-analysis corresponding to 1296 patients: 208 (16%) were males, 439/1230 (35.7%) had the diffuse cutaneous subset, 368/1230 (29.9%) were tested positive for anti-Scl70 antibodies and 460/1230 (37.4%) positive for anti-centromere antibodies. Three biomarkers were studied: KL6, SPD and CCL18.

KL-6, SP-D, and CCL-18 could diagnose SSc-ILD, with KL-6 having the strongest diagnostic value with an OR of 21.87 (95% CI 5.07-94.2) (p<0.001), followed by SP-D (OR: 13.24, 95% CI 3.84-45.71) (p<0.001). Both CCL-18 and KL-6 were accurate for the outcomes of SSc-ILD with CCL18 being the most accurate (CCL18: OR: 2.62, 95% CI 1.71-4.03, p<0.001, vs. KL6: OR: 1.80, 95% CI 1.02-3.17, p<0.001).

**Conclusions:** KL-6 appears as the most promising biomarker for the diagnosis of SSc-ILD, whereas KL-6 and CCL-18 are relevant as prognostic factors. Prospective longitudinal studies are warranted to confirm these results and to monitor response to treatment with the recent availability of new effective therapies in SSc-ILD.

## P.099

## SYSTEMIC MICROANGIOPATHY IN PULMONARY ARTERIAL HYPERTENSION: PRELIMINARY DATA FROM A CROSS-SECTIONAL STUDY

T. Dimitroulas^1^, A. Arvanitaki^1,2^, G. Giannakoulas^2^, E. Triantafyllidou^1^, C. Feloukidis^2^, P. Rouskas^2^, S. Mouratoglou^2^, H. Karvounis^2^, A. Garyfallos^1^

^1^*Fourth Department of Internal Medicine, Hippokration University Hospital, Aristotle University of Thessaloniki, Thessaloniki, Greece*, ^2^*First Department of Cardiology, AHEPA University Hospital, Aristotle University of Thessaloniki, Thessaloniki, Greece*

**Introduction:** Limited amount of evidence suggests the presence of systemic vasculopathy in pulmonary arterial hypertension (PAH). Nailfold video-capillaroscopy (NVC) is an established, non-invasive imaging technique for the assessment of the microcirculation. The aim of this study is to investigate the presence of capillaroscopic abnormalities in patients with PAH and to evaluate the differences between subgroups.

**Material and Methods:** We present preliminary data regarding capillaroscopic abnormalities in patients with PAH. All patients underwent NVC, performed on all fingers of both hands, excluding thumbs, using Optilia Digital Capillaroscope. PAH was confirmed with right heart catheterization performed at the same day with NVC. Capillary quantitative and qualitative parameters were measured.

**Results:** NVC was performed in 30 patients with PAH, [ mean age 52.6 ± 16.7 years, 86.7% female, 27% with idiopathic PAH (IPAH), 30% with PAH associated with systemic sclerosis (SSc-PAH) and 43% with Eisenmenger syndrome (PAH-CHD)], 14 patients with SSc without PAH [mean age 51.6 ± 11.4 years, 86 % female] and 15 healthy controls [mean age 50.9 ± 12 years, 80% female]. Capillaroscopic abnormalities were detected among all PAH subgroups (Figure). Capillary density was significantly decreased in SSc-PAH vs. IPAH vs. healthy controls (6.0 ± 1.1 loops/mm vs. 8.1 ± 0.8 loops/mm vs. 9.8 ± 0.6 loops/mm p<0.001). No differences were detected in capillary density and width between SSc-PAH and SSc-non-PAH patients, which were severely impaired in both groups. Apart from decreased capillary density, IPAH patients presented also more avascular areas/mm compared to healthy controls (0.7 ± 0.3 areas/mm vs. 0.1 ± 0.2 areas/mm, p=0.012). Although capillary density and width were found normal among patients with PAH-CHD, shape abnormalities and signs of neoangiogenesis were more frequent compared with healthy controls (2.7 ± 1.1 abnormal capillaries/mm vs. 1.3 ± 0.7 abnormal capillaries/mm, p=0.005 / 0.8 ± 0.4 ramified capillaries/mm vs. 0.2 ± 0.1 ramified capillaries/mm, p=0.015).

**Conclusions:** These data support the hypothesis of the presence of systemic microvascular involvement in IPAH and PAH-CHD patients, apart from the known microvascular disease in SSc-non-PAH and SSc-PAH patients.

**Figure:**
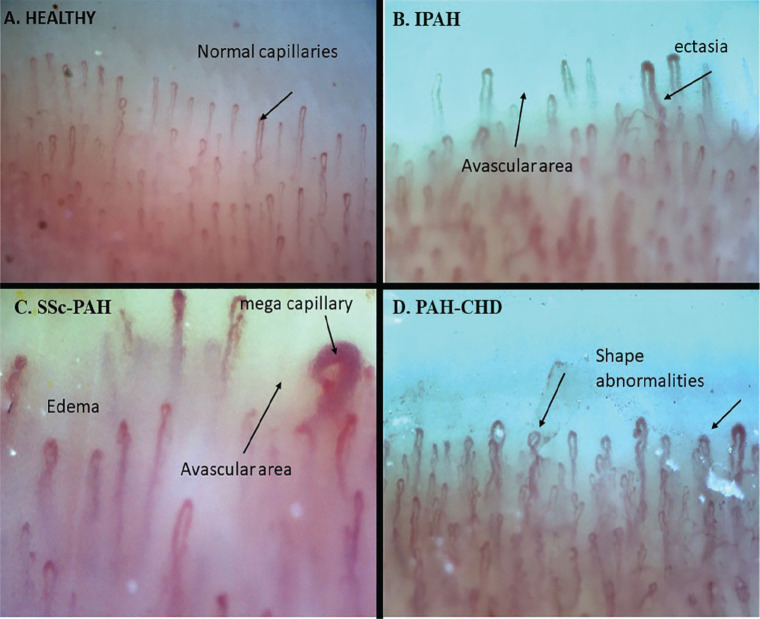


## P.100

## CORRELATION BETWEEN HRCT SCORE WITH PULMONARY FUNCTION TEST ON SYSTEMIC SCLEROSIS PATIENTS

A. Sudianto^1^, H. Soekersi^1^, I. Hasan Hikmat^1^, S. Dewi^2,3^, H. Yani^2,3^, I. Desrianda^1^

^1^*Padjadjaran University, Faculty of Medicine, Department of Radiology, Bandung, Indonesia*, ^2^*Padjadjaran University, Faculty of Medicine, Department of Internal Medicine, Division Rheumatology, Bandung, Indonesia*, ^3^*Padjadjaran University, Immunology Study Center, Bandung, Indonesia*

**Introduction:** Systemic sclerosis is an autoimmune disease characterized by fibrosis of the skin and internal organs, vasculopaty, high in morbidity and mortality. Improved understanding of systemic sclerosis has allowed better systematic screening, assessment, management, and follow-up of the disease. Screening strategies facilitate timely recognition of life-threatening complications and initiation of targeted therapies. Internal organ involvement include pulmonary fibrosis, pulmonary hypertension, dismotility esophagus, cardiac fibrosis, and scleroderma renal crisis. The aim of this study is to determine the relationship of pulmonary fibrosis scores using High Resolution Computed Tomography (HRCT) scan with pulmonary function tests (PFTs) by spirometry in systemic sclerosis.

**Material and Methods:** This is observational study with a cross-sectional design on systemic sclerosis patients who visited the rheumatology outpatient clinic at Hasan Sadikin General Hospital Bandung Indonesia and have had PFTs by spirometry and lung HRCT examination from Juny 2019 to August 2019. We assessed the lung HRCT using Warrick score method to evaluate appearance of pulmonary fibrosis and pulmonary function using spirometry to evaluate restrictive values. Data were analyzed using the Rank Spearman correlation test between HRCT pulmonary fibrosis scores with PFTs by spirometry.

**Results:** Twenty nine female systemic sclerosis patients were analyzed, all subjects were diffuse cutaneous type. The average age was 18 to 58 years old, with median disease duration was 3 (1-23 years). The HRCT score describe as ground glass appearance, irregular pleural margins, septal/subpleural lines, honeycombing and subpleural cysts. By Warrick score method we found the grading of the HRCT were grade 1 at 13 subjects (44,8%), the grade 2 were 13 subjects (44,8%), the grade 3 were 1 subject (3,4%), and normal HRCT were 2 (6,9%). We found the pulmonary function tests (PFTs) based on FVC restrictive pulmonary disease criteria by spirometry in majority of subjects have had moderate restrictive pulmonary abnormality at 22 (75,9%), mild restrictive pulmonary abnormality at 7 (24,1%), and no found of severe restrictive lung disease. There is a significant correlation between HRCT score with restrictive lung disease by spirometry with r = 0.468, p value = 0.010.

**Conclusions:** There is a significant correlation between pulmonary fibrosis scores using HRCT with spirometry values in systemic sclerosis.

## P.101

## HAEMOSTATIC FUNCTION AND FIBRIN CLOT PROPERTIES IN PATIENTS WITH SYSTEMIC SCLEROSIS

J. Colic^1^, A. Antovic^2^, I. Pruner^3^, J. Vojinovic^4^, M. Sefik-Bukilica^1^, N. Damjanov^1^

^1^*Institute of rheumatology-Department of Rheumatology Unit, Belgrade, Serbia*, ^2^*Karolinska Institute-Department of Medicine, Rheumatology Unit, Stockholm, Sweden*, ^3^*Karolinska Institute-Department of Molecular Medicine and Surgery, Stockholm, Sweden*, ^4^*Clinical Center of Nis-Department for Pediatric Rheumatology, Nis, Serbia*

**Introduction:** Vasculopathy, the main pathologic event in Systemic sclerosis (SSc), is connected with the activation of coagulation. However, the role of fibrinolytic system remains unclear since both depressed and normal fibrinolytic profiles have been observed. We investigated the haemostatic function, fibrin clot density and clot lysis time (Lys50t0) in SSc patients and healthy controls (HC) to determine their relation to disease.

**Material and Methods:** Our study included 58 SSc patients [36 limited (lcSSc) and 22 diffuse cutaneous SSc (dcSSc)] and 46 sex/age matched HC. Clinical evaluation of patients was obtained, including computed tomography and pulmonary function tests. Disease activity was assessed by the revised EUSTAR activity index. Serum concentration of ICAM1 and von Willebrand factor antigen (VWF) were measured by ELISA. Haemostatic potential parameters; including overall haemostasis (OHP), overall coagulation (OCP) and overall fibrinolysis (OFP) potential, were assessed and endogenous thrombin potential (ETP) was determined. Maximum absorbance (Cmax) and Lys50t0 were calculated from OHP and OCP curves. Statistical analyses was done in STATA.

**Results:** The OFP value was significantly decreased, Lys50t0 prolonged (p<0.05), while OHP and ETP were increased (p<0.05) in patients. In dSSc group, Cmax and Lys50t0 were higher respect to HC (p<0.05). In SSc cohort, positive association was found between coagulation parameters (OCP,OHP,Cmax) and the erythrocyte sedimentation rate (ESR), fibrinogen and ICAM1 (respectively p<0.05). Lys50t0 was positively correlated with ICAM1, ESR and VWF (respectively p<0.001, p<0.05, p<0.05). Inverse correlation was found between Cmax and both diffusing capacity of the lungs for carbon monoxide (r=-0.408, p<0.01) and forced vital capacity (r=-0.378, p<0.01). Increased Cmax was found in interstitial lung disease group (15/58) (p<0.01) and in patients with active disease (29/58) (p<0.01). Longer Lys50t0 was observed in piting scars group. In the multivariate analysis, prolonged Lys50t0 was independently associated with ICAM1 (OR 1.12, 95% CI 1.03–1.2, p<0.01).

**Conclusions:** Our results provide evidence of enhanced coagulation and depressed fibrinolysis in SSc, in connection with inflammation and endothelial injury. Increased ICAM-1 levels could independently reflect impaired fibrinolysis. Denser plasma fibrin clots might be indicative for lung involvement in these patients.

## P.102

## EVOLUTION OF SYSTEMIC SCLEROSIS-RELATED INTERSTITIAL LUNG DISEASE ONE YEAR AFTER TREATMENT WITH AUTOLOGOUS HEMATOPOIETIC STEM CELL TRANSPLANTATION OR CYCLOPHOSPHAMIDE

J. Ciaffi^1^, N.M. Van Leeuwen^1^, M. Boonstra^1^, L.J. Kroft^2^, A.A. Schouffoer^1^, M.K. Ninaber^3^, T.W. Huizinga^1^, J.K. De Vries-Bouwstra^1^

^1^*Leiden University Medical Center - Department of Rheumatology, Leiden, The Netherlands*, ^2^*Leiden University Medical Center - Department of Radiology, Leiden, The Netherlands*, ^3^*Leiden University Medical Center - Department of Pulmonology, Leiden, The Netherlands*

**Introduction:** Autologous hematopoietic stem cell transplantation (HSCT) and cyclophosphamide (CYC) are treatment options for interstitial lung disease (ILD) associated with systemic sclerosis (SSc). The aims of our study are to evaluate the effectiveness of HSCT and CYC in inducing reduction of ILD extension assessed through high resolution computed tomography (HRCT) in SSc patients, to investigate how pulmonary function test (PFT) changes are associated with HRCT modifications, and which patients are most likely to show ILD reduction.

**Material and Methods:** We included SSc patients with evidence of ILD at HRCT, treated with either HSCT or pulsed CYC. Two experienced researchers scored baseline and 1-year post-treatment HRCTs using the “Goh system”. A cut-off of 5% was used to define improvement/progression. PFTs close to the HRCT time points were collected. Linear association of relative changes in forced vital capacity (FVC) and diffusing lung capacity for carbon monoxide (DLCO) with changes in HRCT score was studied. In addition, regression analysis including age, disease duration and subset, autoantibody positivity, baseline skin score, along with pre-treatment PFTs and HRCT scores, was performed to determine which patients are most likely to show improvement in ILD extent.

**Results:** Fifty-one patients (n=20 HSCT; n=31 CYC) were included. Mean Goh score decreased from 27±14.7% to 21.7±11.8% (p=0.043) in the HSCT group, and from 25.2±13.8% to 24.3±13.7% (p=0.56) in the CYC group (Table 1). Seven HSCT patients improved (35%), 11 remained stable (55%), and 2 progressed (10%). In the CYC group 6 patients improved (19%), 22 remained stable (71%), and 3 progressed (10%) (all p>0.05). One year after treatment, mean FVC and DLCO increased in both groups with the FVC increase being significant in the HSCT group (HSCT: FVC from 77.8±18.1% to 84.7±19.2% (p<0.001), DLCO from 53.4±19.2% to 55.1±15% (p=0.431); CYC: FVC from 80.2±16.7% to 84.6±19.7% (p=0.077), DLCO from 53.3±11.6% to 55.6±13.1% (p=0.209); p not significant between groups). For all patients, evolution in mean Goh score was clearly associated with changes in FVC (p<0.001), and DLCO (p=0.015). Among the characteristics analysed, higher ground-glass and total Goh scores, and lower DLCO, predicted ILD improvement at follow-up HRCT.

**Conclusions:** In SSc, both HSCT and CYC can induce reduction of ILD extent and improvement of FVC, with only HSCT showing significant improvement in total Goh score and FVC. In the combined population, changes in PFTs were associated with changes in HRCT findings. Patients with more extensive lung disease at baseline had better response to immunosuppression.

**Figure:**
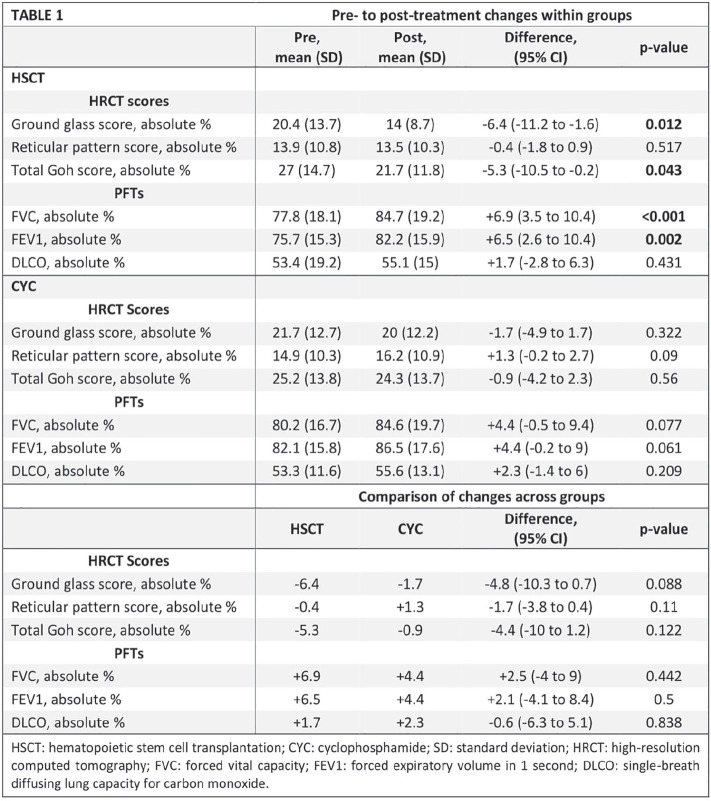


## P.103

## EFFECTS OF NINTEDANIB IN PATIENTS WITH EXTENSIVE AND LIMITED SYSTEMIC SCLEROSIS-ASSOCIATED ILD (SSC-ILD): FURTHER ANALYSES OF THE SENSCIS TRIAL

D. Christopher^1^, N. Goh^2^, D. Lynch^3^, T.M. Maher^4^, V. Smith^5^, V. Cottin^6^, R. Spiera^7^, C. Stock^8^, M. Gahlemann^9^, M. Alves^10^, A.U. Wells^11^

^1^*University College London Division of Medicine, Centre for Rheumatology and Connective Tissue Diseases, London, United Kingdom*, ^2^*Respiratory and Sleep Medicine, Austin Health, and Institute for Breathing and Sleep, Melbourne, Victoria, Australia*, ^3^*Department of Radiology, National Jewish Health, Denver, Colorado, USA*, ^4^*National Heart and Lung Institute, Imperial College London and NIHR Clinical Research Facility, Royal Brompton Hospital, London, United Kingdom*, ^5^*Dept. of Rheumatology, Ghent University Hospital; Dept. of Internal Medicine, Ghent University, Ghent, Belgium*, ^6^*National Reference Center for Rare Pulmonary Diseases, Louis Pradel Hospital, Lyon, France*, ^7^*Division of Rheumatology, Hospital for Special Surgery, New York, USA*, ^8^*Boehringer Ingelheim Pharma GmbH & Co. KG, Biberach, Germany*, ^9^*Boehringer Ingelheim (Schweiz) GmbH, Basel, Switzerland*, ^10^*Boehringer Ingelheim International GmbH, Ingelheim am Rhein, Germany*, ^11^*NIHR Respiratory Biomedical Research Unit, Royal Brompton and Harefield NHS Foundation Trust, London, United Kingdom*

**Introduction:** In the SENSCIS trial in patients with SSc-ILD, nintedanib reduced the annual rate of decline in forced vital capacity (FVC) (mL/year) by 44% vs placebo. Previous studies showed that patients with SSc-ILD who had more extensive lung disease on an HRCT scan had a worse prognosis than patients with less extensive disease. We assessed the effect of nintedanib in subgroups of patients with limited and extensive SSc-ILD.

**Material and Methods:** Subjects with first non-Raynaud symptom <7 years before screening, extent of fibrotic ILD >=10% on an HRCT scan performed <=12 months before screening, and FVC >=40% predicted were randomised to receive nintedanib or placebo. We analysed outcomes over 52 weeks in subgroups by limited ILD (extent of fibrotic ILD on HRCT <=10%, or extent of fibrotic ILD >10% to <=30% with FVC >=70% predicted) or extensive ILD (extent of fibrotic ILD on HRCT >30%, or extent of fibrotic ILD >10% to <=30% with FVC <70% predicted) at baseline.

**Results:** Of 288 patients per treatment group, 180 (62.5%) and 178 (61.8%) of subjects in the nintedanib and placebo groups, respectively, had extensive ILD. Mean (SD) FVC was 2787 (732) mL and 83.1% predicted in patients with limited ILD and 2325 (752) mL and 66.1% predicted in patients with extensive ILD. The effect of nintedanib vs placebo on the annual rate of FVC decline was numerically greater in patients with extensive ILD (difference 49.4 mL/year [95% CI 0.6, 98.1]) than limited ILD (difference 27.5 mL/year [95% CI -33.6, 88.5]), but statistical testing did not indicate a heterogenous treatment effect between subgroups (p=0.58 for treatment-by-time-by-subgroup interaction). The effect of nintedanib vs placebo on the annual rate of FVC decline was numerically greater in patients with extent of fibrotic ILD >=30% (difference 66.2 mL/year [95%.7 CI 15.8, 116.7]) than <30% (difference 8.8 mL/year [95% CI -49.1, 66.7]), but statistical testing did not indicate a heterogenous treatment effect between subgroups (interaction p=0.14). The effect of nintedanib vs placebo was consistent in subjects with FVC <70% predicted (difference 32.6 mL/year [95% CI -25.2, 90.5]) and >=70% predicted (difference 44.5 mL/year [95% CI -5.9, 94.9]) (interaction p=0.76). The adverse event profile of nintedanib was consistent across subgroups.

**Conclusions:** Nintedanib reduced the rate of ILD progression in patients with SSc-ILD. Nintedanib had a numerically greater treatment effect in patients with extensive than limited lung disease, but statistical testing did not indicate a heterogenous treatment effect between subgroups.

**Figure:**
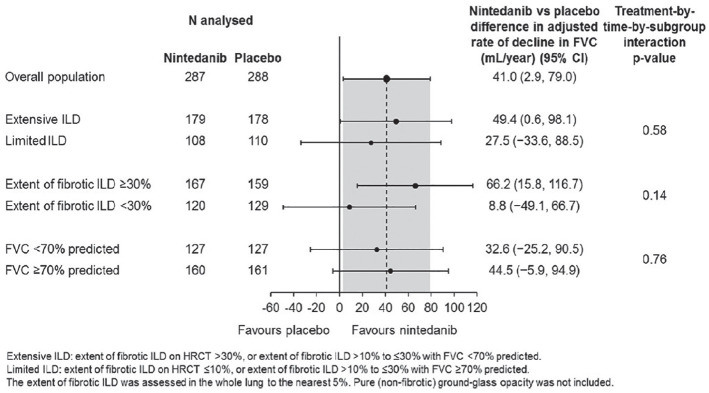


## P.104

## FOLLOW-UP EVALUATION OF A PROTOCOL-BASED TREATMENT FOR SYSTEMIC SCLEROSIS RELATED-INTERSTITIAL LUNG DISEASE

J. Caetano^1^, F. Paula^1,2^, M. Amaral^1,2^, S. Oliveira^1^, J. Delgado Alves^1,2^

^1^*Systemic Immune mediated Diseases Unit, Medicine IV, Fernando Fonseca Hospital, Amadora, Portugal*, ^2^*CEDOC, Nova Medical School, Lisboa, Portugal*

**Introduction:** Interstitial lung disease (ILD) is a main cause of morbidity and mortality in systemic sclerosis (SSc), and effective treatment is limited. Our aim is to evaluate the outcome of patients (pts) with SSc-related ILD from our SSc cohort treated with a defined protocol.

**Material and Methods:** Retrospective analysis of 74 consecutive SSc pts, followed from 2010-2018. Pts with SSc-ILD at least with 1 year of stable treatment were included. Protocol treatment: 1) limited ILD: mycophenolate mofetil (MMF); 2) extensive ILD: endovenous (ev) cyclophosphamide followed by MMF; 3) progressive disease (>=10% relative decline in FVC or >=5% to <10% relative decline in FVC and >=15% in DLCO; respiratory failure): ev tocilizumab. Outcomes: change in ILD extent (chest HRCT), lung function tests (LFT) (% predicted FVC and TLC, DLCO), symptoms (cough, dyspnea), Medsger disease severity scale (MDSS) for ILD, long-term oxygen therapy (LTO), death.

**Results:** From a total of 28 pts with SSc-ILD, 22 were included - 72.7% women; mean age 59.6±12.3years (yrs); time since SSc-onset 8.3±5.1yrs; 45.5% diffuse skin subtype; 3 most common autoantibodies: 41% anti-scl70, 13.6% anti-RNA polymerase III, 13.6% anti-U1RNP. 12 Pts had extensive ILD and 10 had limited ILD. After the 1st year of treatment until the last follow-up the extent of ILD in HRCT was worse in 7 pts with extensive ILD, of which 5 fullfield criteria for progressive ILD. ILD extent remained stable in the other pts. The mean treatment duration for each group was: extensive - 3.3±2.9yrs, limited - 5.6±3.1yrs, and progressive - 4.9±2.3yrs. Comparing with baseline values mean FVC, TLC and DLCO increased in limited ILD, not statistically significant. In extensive disease, mean FVC decreased (-7.4±16.4%), TLC and DLCO were stable. LFT remained stable during follow-up in pts with progressive ILD. Dyspnea was present initially in 73% (68% extensive ILD) and cough in 27% (100% extensive ILD). At last follow-up, dyspnea decreased in 50%. MDSS was higher from baseline in extensive and progressive ILD (1.8±0.9 vs 2.4±1.2; 1.5±1.1 vs 2.25±1.2, respectively), and stable in limited ILD (1±0.8 vs 1±1.0). LTO was required in 3 pts. 4 Pts died (18.2%), 3 with progressive ILD, 1 due to ILD.

**Conclusions:** This treatment protocol was effective in limited ILD, but not in an important proportion of pts with extensive disease, regarding symptoms, ILD extent and LFT. Based on this, we are reformulating our protocol with earlier combination of immunossuppressants and anti-fibrotics for this subgroup.

## P.105

## THE LINK BETWEEN PARENCHYMAL AND VASCULAR FEATURES IN SYSTEMIC SCLEROSIS-INTERSTITIAL LUNG DISEASE (SSC-ILD): A QUANTITATIVE ANALYSIS OF IMAGING FEATURES AT CHEST CT

C. Bruni^1^, M. Occhipinti^2,3^, G. Camiciottoli^2^, M. Bartolucci^4^, M. Pienn^5^, G. Lepri^1^, A. Fabbrizzi^2^, A. Tottoli^1^, G. Ciardi^2^, D. Giuggioli^6^, G. Cuomo^7^, F. Masini^7^, H. Olschewski^5,8^, F. Lavorini^2^, S. Colagrande^3^, M. Matucci Cerinic^1^

^1^*University of Florence, Dept. Experimental and Clinical Medicine, Division of Rheumatology, Florence, Italy*, ^2^*University of Florence, Dept. Experimental and Clinical Biomedical Sciences Mario Serio, Div. Pulmunology, Florence, Italy*, ^3^*University of Florence, Dept. Experimental and Clinical Biomedical Sciences Mario Serio, Div. Radiology, Florence, Italy*, ^4^*Azienda Ospedaliera Universitaria Careggi, Dept. of Services, Div. Emergency-Urgency Radiology, Florence, Italy*, ^5^*Medical University of Graz, Ludwig Boltzann Institute For Lung Vascular Research, Institute of Physiology, Graz, Austria*, ^6^*University of Modena and Reggio Emilia, Rheumatology Unit, Modena, Italy*, ^7^*University Luigi Vanvitelli of Campania, Dept. of Precision of Medicine, Div. Internal Medicine, Naples, Italy*, ^8^*Medical University of Graz, Dept. Internal medicine, Div. Pulmonology, Graz, Austria*

**Introduction:** Interstitial lung disease (ILD) and pulmonary arterial hypertension carry a negative impact on SSc prognosis. Chest CT is the gold standard in assessing ILD and helps in evaluating associated vascular involvement. As qualitative analysis of CT scans is limited by low reproducibility and time constraints, we aimed at evaluating parenchymal and vascular features in SSc-ILD by quantitative analysis (QA) of CT scans and testing the relationship with clinical-functional data.

**Material and Methods:** We prospectively enrolled 80 patients who underwent PFTs and chest CT scan spirometry gated at TLC on the same day. Clinical, lung functional and diffusion data, as well as disability indexes were collected. CT images were analyzed by a computational platform for texture analysis of ILD patterns (CALIPER), through Imbio LTA. It quantified the extent of normal pattern (NP%), ground glass opacities (GG%), reticulation (RET%), honeycombing (HC%) and hyperlucent (HL%). For lung vessel analysis, a software developed by the Ludwig Boltzmann Institute for Lung Vascular Research was used. This software determined total, arterial, and venous vascular volumes (TV, AV, VV), and relative volumes (TV%, AV%, VV%), as well as density and number for total, arterial and venous vessels.

**Results:** 44 patients/CT scans were eligible (89% female, 42% diffuse, 4.5% PAH) for both software analyses. CALIPER showed GG% as the most frequent radiological pattern (mean 3.8±6.0%), with significant association with CRP increase and pericardial effusion. GG% and RET% were significantly positively correlated with mRSS and negatively correlated with %FVC (r=-0.546 and r=-0.370), %TLC (r=-0.552 and r=-0.386) and blood oxygen pressure and saturation. Similarly, TV% and total vessel density were correlated positively with mRSS and negatively with %FVC (r=-0.523 and r=-0.360) and %TLC (r=-0.511 and r=-0.648). This was confirmed when separately analyzing arterial and venous counterparts. There was a positive correlation between %ILD patterns and %vascular volumes, being more significant for %AV and arterial density then for the whole vasculature. Conversely, %ILD patterns were negatively correlated with VV and number of veins detected.

**Conclusions:** This is the first study showing in SSc a direct correlation between ILD and the increase in lung vascular volume, which is characterized by increase in arterial volume and density and reduction in venous volume and number. The reduction of pulmonary volume due to fibrosis or a para-physiological mechanism of redistribution of blood flow in lung areas less involved by ILD could explain these results. Further studies on lung vessel quantification and distribution are ongoing

**Figure:**
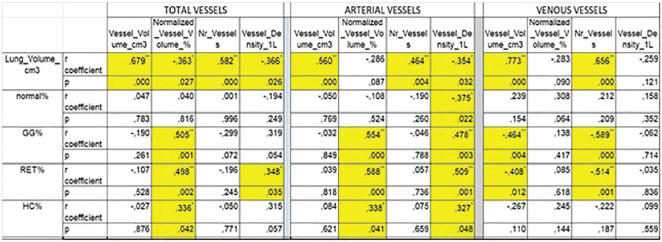


## P.106

## SCREENING TOOLS FOR PULMONARY ARTERIAL HYPERTENSION (PAH) IN SYSTEMIC SCLEROSIS (SSC): A SYSTEMATIC LITERATURE REVIEW (SLR)

C. Bruni^1^, G. De Luca^2,3^, M.-G. Lazzaroni^4^, E. Zanatta^5^, M. Matucci-Cerinic^1^

^1^*University of Florence, Dept. Experimental and Clinical Medicine, Florence, Italy*, ^2^*IRCSS San Raffaele Hospital; Immunology, Rheumatology, allergology and rare diseases unit, Milan, Italy*, ^3^*Vita-Salute San Raffaele University, Milan, Italy*, ^4^*ASST Spedali Civili di Brescia - University of Brescia, Rheumatology and Clinical immunology unit, Brescia, Italy*, ^5^*University Hospital of Padua, Dept. Medicine, Rheumatology Unit, Padua, Italy*

**Introduction:** PAH carries a high morbidity and mortality burden in SSc. We searched the literature for all screening modalities for SSc-PAH in reference to right heart catheterization as diagnostic gold standard.

**Material and Methods:** papers from 2 previously published SLRs [22 from Glaude et al 2014 – from inception to 19/06/2012 - and 22 from Young et al 2018 – from 20/06/2012 to 02/10/2017] were included. The articles’ database was integrated with a systematic search on Pubmed, EMBASE, Web of Science for papers published from 03/10/2017 to 31/12/2018. A total of 199 papers were reviewed and 32 were finally extracted. Bias risk was assessed through QUADAS2 tool.

**Results:** 167 papers were excluded from data extraction mainly for PAH screening non as main focus or for non-including SSc patients. The 32 papers extracted presented a low bias risk according to QUADAS2.

Screening methods reported were:

- Echocardiographic parameters in 31/32 studies, in particular systolic pulmonary arterial pressure (sPAP) in 22 papers; 40 mmHg was the most frequently used cut-off (in 12/22 papers); sPAP was part of a composite algorithm in 9/22 papers. Among others, tricuspid regurgitation velocity (TRV) was used in 6/31 (as part of composite 5/6) and right atrial pressure (RAP) in 3/31 papers.
- Pulmonary function tests parameters in 22/32 papers, with % predicted Lung diffusion for carbon oxyde (DLCO) in 21 papers, with a 50% cut-off in 11/21 and as part of composite algorithm in 13/21 studies. Moreover, walked distance at six minutes walking test was a screening parameter in 3/32 papers.
- Serum biomarkers in 12/32 papers, with anti-centromere antibodies (6/12), NT-proBNP (6/12) and uric acid (5/12) being the most frequently reported.
- Clinical parameters in 15/32 papers, with unexplained dyspnoea in 9/15 and telangiectasias in 5/15 papers.
- Composite algorithms were used in 18/32 manuscripts: among them, DETECT (5/18), ESC/ERS 2009 (4/18) or 2015 (3/18) guidelines, ASIG (2/18) e ITINER-air (1/18). In different cohorts, DETECT and ASIG showed higher sensitivity and negative predictive value than ESC/ERS 2009.

**Conclusions:** today, SSc-PAH is mostly evaluated on echocardiographic parameters, in particular sPAP and TRV, both as single items or part of a composite algorithm including also serum biomarkers, clinical and functional parameters.

(supported by an Actelion Pharmaceuticals unrestricted research grant)

**Figure:**
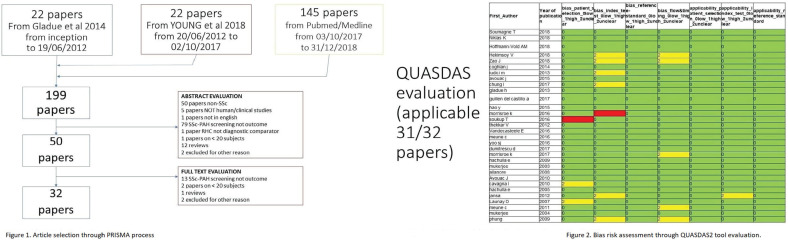


## P.107

## CARDIAC CATHETERIZATION VERSUS ECHOCARDIOGRAPHY FOR MONITORING PULMONARY PRESSURE: A PROSPECTIVE STUDY IN PATIENTS WITH CONNECTIVE TISSUE DISEASE-ASSOCIATED PULMONARY ARTERIAL HYPERTENSION

V.-K. Bournia^1^, I. Tsangaris^2^, L. Rallidis^2^, D. Konstantonis^2^, F. Frantzeskaki^2^, A. Anthi^2^, S. Orfanos^2^, E. Demerouti^3^, P. Kariofyllis^3^, V. Voudris^3^, A. Laskari^1^, S. Panopoulos^1^, P. Vlachoyiannopoulos^4^, P. Sfikakis^1^

^1^*First Department of Propedeutic Internal Medicine, Medical School, National and Kapodistrian University of Athens, Athens, Greece*, ^2^*Pulmonary Hypertension Clinic, Attikon University General Hospital, Medical School, National and Kapodistrian University, Athens, Greece*, ^3^*Invasive Cardiology Department, Onassis Cardiac Surgery Center, Athens, Greece*, ^4^*Department of Pathophysiology, Medical School, National and Kapodistrian University of Athens, Athens, Greece*

**Introduction:** Standard echocardiography is important for pulmonary arterial hypertension (PAH) screening in patients with connective tissue disease (CTD), but PAH diagnosis and monitoring should be performed with cardiac catheterization. Herein, using cardiac catheterization as reference, we tested the hypothesis that follow-up echocardiography is adequate for clinical decision-making in these patients.

**Material and Methods:** We prospectively studied consecutive patients with systemic sclerosis (n=58), systemic lupus erythematosus (n=5) and mixed connective tissue disease (n=6) associated PAH. Invasive baseline pulmonary artery systolic pressure (PASP) was 60.19±16.33 mmHg (mean±SD) and pulmonary vascular resistance (PVR) was 6.44±2.95 WU. All patients underwent hemodynamic and echocardiographic follow-up after 9.47±7.29 months; 27 patients had a third follow-up after 17.2±7.4 months from baseline. We examined whether clinically meaningful hemodynamic deterioration of follow-up catheterization-derived PASP (i.e. >10% increase) could be predicted by simultaneous echocardiography.

**Results:** Echocardiography could predict hemodynamic PASP deterioration with 59% sensitivity, 85% specificity, and 63/83% positive/negative predictive value, respectively. In multivariate binary logistic regression analysis, successful echocardiographic prediction correlated only with higher PVR in previous catheterization (p=0.05, OR=1.235). Notably, in patients having baseline PVR>5.45 WU, echocardiography had both sensitivity and positive predictive value of 73% and both specificity and negative predictive value of 91% for detecting hemodynamic PASP deterioration.

**Conclusions:** In selected patients with CTD-PAH non-invasive monitoring can predict PASP deterioration with a high specificity and negative predictive value. Additional prospective studies are needed to confirm that better selection of patients can increase the ability of standard echocardiography to replace repeat catheterization.

## P.108

## A SINGLE HUNGARIAN PULMONOLOGY CENTER EXPERIENCE ON SCLERODERMA RELATED INTERSTITIAL LUNG DISEASES

A. Bohács^1^, K. Vincze^1^, E. Kiss^2^, A. Polgár^2^, Á.D. Tárnoki^3^, D.L. Tárnoki^3^, K. Karlócai^1^, K. Karlinger^3^, V. Müller^1^

^1^*Dep. of Pulmonology of Semmelweis University, Budapest, Hungary*, ^2^*National institute of rheumatology and physiotherapy, Budapest, Hungary*, ^3^*Dep. of Radiology of Semmelweis Univerity, Budapest, Hungary*

**Introduction:** Interstitial lung disease (ILD) and pulmonary hypertension (PH) is a common manifestation of systemic sclerosis (SSc) and a leading cause of systemic sclerosis related death. Clinically significant ILD is seen in up to 40% of patients and PH in up to 20%. The purpose of this study was to examine the correlation between the radiological imaging pattern of interstitial pneumonia in SSc and the pulmonary function test data.

**Material and Methods:** A retrospective study comparing lung functional parameters of SSc patients with or without ILD who visited our center from 2017 to 2018. Lung function parameters and diffusion capacity (Tlco) were compared with the ILD pattern on chest high resolution computer tomography (HRCT). Pulmonary hypertension (PH) was diagnosed if the patient is under PH therapy in our PH center.

**Results:** Six patients of the full 83 SSc cohort were excluded, because of missing data. 77 were analysed. On the chest HRCT, 20 (26%) patients had no ILD, 24 (31.2%) subjects had non specific interstitial pneumonitis (NSIP), 5 (6.5%) patients had emphysema+NSIP or pUIP, and 7 (9,1%) had ground glass opacity (GGO), 10 (12.9%) had possible usual interstitial pneumonitis (pUIP), 7 (9.1%) had UIP pattern. All of our patients (n=5) who had obstuctive ventilatory disorder on lung function test (FEV1/FVC was 65.8%±1.3) emphysema had on HRCT. The Tlco was significant lower (p<0.05) if emphysema+ILD or NSIP or UIP pattern was determine (92.22±6.02 vs. 63.8±5.5 vs. 69.48±4.1 vs. 42.29±8.2). 16 (20.8%) SSc patients had PH, 5 had NSIP, 3 had pUIP, 3 had UIP, 3 had no ILD, 1 had other, 1 had emphysema+pUIP.

**Conclusions:** 74% of our SSc patient had ILD, the most common pattern was the NSIP and 20,8% of them had PH. Pulmonology follow-up and chest HRCT of SSc patients is highly important. Novel antifibrotic therapy in SSc-ILD could diminish the decline of lung function. We have to recognize the pulmonary involvement of SSc for further treatment.

## P.109

## TREATMENT WITH A THROMBIN INHIBITOR, DABIGATRAN ETEXILATE, REDUCES PROTOTYPIC MARKER OF DIFFERENTIATED MYOFIBROBLASTS IN SCLERODERMA LUNG

G. Bogatkevich, I. Atanelishvili, R. Silver

*Medical University of South Carolina, Charleston, USA*

**Introduction:** Myofibroblasts are the principal mesenchymal cells responsible for tissue remodeling, collagen deposition, and the restrictive nature of lung parenchyma characteristic of systemic sclerosis-associated interstitial lung disease (SSc-ILD). A characteristic feature of myofibroblasts is increased contractile activity resulting from high smooth muscle alpha-actin (SMA) expression. Previously, we demonstrated that thrombin differentiates normal lung fibroblasts to a myofibroblast phenotype. The objective of this study was to investigate effects on SSc lung myofibroblasts when the direct thrombin inhibitor dabigatran etexilate is administered to scleroderma patients.

**Material and Methods:** Lung myofibroblasts were isolated by bronchoalveolar lavage (BAL) from 14 SSc-ILD patients before and after receiving dabigatran etexilate (Pradaxa) in a dose of 75 mg twice daily for 6 months (ClinicalTrials.gov Identifier NCT02426229). Total RNA was extracted from lung fibroblasts and purified with RNeasy Mini Kit from Qiagen. SMA expression was determined by RT-PCR. Thrombin activity in BAL fluid and plasma was measured by a spectrophotometric method.

**Results:** Our recently published clinical trial demonstrated that dabigatran etexilate is both safe and well tolerated when administered in a dose of 75mg twice daily to patients with SSc-ILD. Lung myofibroblasts isolated from BAL fluid from scleroderma patients before dabigatran treatment demonstrated enhanced contractile activity and high levels of SMA expression. Relative lung fibroblast differential gene expression of SMA was reduced in a majority of SSc-ILD patients after 6 months of treatment with dabigatran. Analysis of paired samples from each of 14 patients showed that SMA gene expression decreased by up to 38.75±15.55% in 8 patients and remained unchanged in 3 patients. Three patients showed a slight increase in SMA gene expression (1.16±0.26-fold, 1.19±0.26-fold, and 1.66±0.03-fold). Measurements of BAL fluid thrombin activity revealed that the most profound reduction was achieved in patients having the highest baseline thrombin activity. These patients were also characterized by the greatest reduction of SMA lung fibroblast gene expression following treatment with dabigatran.

**Conclusions:** This is the first study to demonstrate antifibrotic effects of dabigatran etexilate treatment at the molecular level in scleroderma patients with high thrombin activity. A low dose of dabigatran etexilate sufficiently reduces lung fibroblast contractility and SMA gene expression in lung myofibroblasts suggesting that dabigatran etexilate might prove to be a promising drug for the treatment of patients with SSc-ILD.

## P.110

## EFFECT OF NINTEDANIB ON PROGRESSION OF SYSTEMIC SCLEROSIS-ASSOCIATED INTERSTITIAL LUNG DISEASE (SSC-ILD) OVER 100 WEEKS: DATA FROM THE SENSCIS TRIAL

S. Assassi^1^, O. Distler^2^, Y. Allanore^3^, T. Ogura^4^, J. Varga^5^, S. Vettori^6^, B. Crestani^7^, U. Von Wangenheim^8^, M. Quaresma^9^, M. Alves^9^, S. Stowasser^9^, T.M. Maher^10^

^1^*Division of Rheumatology and Clinical Immunogenetics, University of Texas McGovern Medical School, Houston, Texas, USA*, ^2^*Department of Rheumatology, University Hospital Zurich, Zurich, Switzerland*, ^3^*Department of Rheumatology A, Descartes University, APHP, Cochin Hospital, Paris, France*, ^4^*Department of Respiratory Medicine, Kanagawa Cardiovascular and Respiratory Centre, Yokohama, Japan*, ^5^*Northwestern Scleroderma, Feinberg School of Medicine, Chicago, Illinois, USA*, ^6^*Università degli Studi della Campania Luigi Vanvitelli, Naples, Italy*, ^7^*Hôpital Bichat, Pneumologie, Paris, France*, ^8^*Boehringer Ingelheim Pharma GmbH & Co. KG, Biberach, Germany*, ^9^*Boehringer Ingelheim International GmbH, Ingelheim am Rhein, Germany*, ^10^*National Heart and Lung Institute, Imperial College London and NIHR Clinical Research Facility, Royal Brompton Hospital, London, United Kingdom*

**Introduction:** In the SENSCIS trial, patients with SSc-ILD were randomised to receive nintedanib or placebo until the last patient reached week 52 but for no longer than 100 weeks. Patients who prematurely discontinued treatment were asked to attend visits up to week 100 or the end of the trial. Nintedanib reduced the annual rate of decline in FVC over 52 weeks (primary endpoint) by 44% versus placebo (-52.4 vs -93.3 mL/year; difference 41.0 mL/year [95% CI 2.9, 79.0]). We assessed the effect of nintedanib on progression of ILD over the whole trial.

**Material and Methods:** We assessed the rate of decline in FVC (mL/year) over the whole trial (up to 100 weeks) using three approaches. The first analysis was based on all available data, including data collected after treatment discontinuation. Secondly, we conducted an analysis including post-treatment data only from patients who prematurely discontinued treatment. Thirdly, we conducted an analysis including only on on-treatment data (i.e., measurements taken between randomisation and the date of last trial drug intake or week 100, whichever occurred first) to explore the expected effect of nintedanib in patients who remained on treatment. Between-group differences in FVC (mL) at week 100 were estimated. Safety was assessed based on adverse events reported up to the last drug intake plus 28 days. Analyses were descriptive.

**Results:** At baseline, mean (SD) FVC was 2459 (736) mL in the nintedanib group (n=288) and 2541 (816) mL in the placebo group (n=288). Median exposure to study drug over the whole trial was 15.4 and 15.6 months in the nintedanib and placebo groups, respectively. The adjusted mean (SE) annual rates of decline in FVC over 100 weeks based on the three approaches are presented in the Table. Adverse events leading to permanent discontinuation of study drug were reported in 17.4% and 10.1% of patients treated with nintedanib and placebo, respectively.

**Conclusions:** Collectively, data from the SENSCIS trial suggest that the effect of nintedanib on slowing the progression of SSc-ILD persists beyond 52 weeks. The adverse event profile of nintedanib over the whole trial (up to 100 weeks) was consistent with that reported over 52 weeks.

**Figure:**
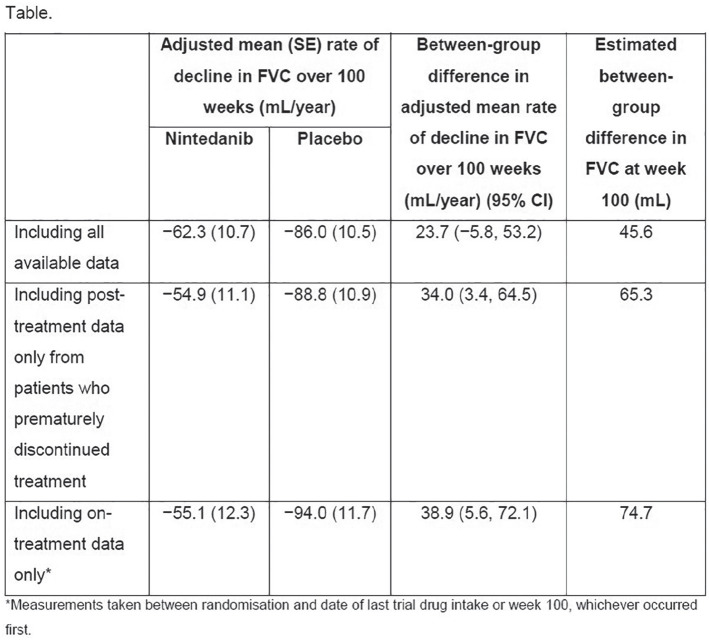


## P.111

## DLCO IN MONITORING AND MANAGING DISEASE PROGRESSION IN PATIENTS WITH SYSTEMIC SCLEROSIS AND INTERSTITIAL LUNG DISEASE

C. Ancuta^1^, C. Pomirleanu^1^, R. Paiu^1^, E. Ancuta^2^, T. Cernomaz^3^, R. Chirieac^4^

^1^*Clinical Rehabilitation Hospital, Iasi, Romania*, ^2^*Elena Doamna Clinical Hospital, Iasi, Romania*, ^3^*Grigore T Popa University of Medicine and Pharmacy, Iasi, Romania*, ^4^*SANOCARE Medical and Research Center, Iasi, Romania*

**Introduction:** Interstitial lung disease (ILD) remains a major challenge in systemic sclerosis (SSc), significantly limiting quality of life and survival. Unmet needs still focus on overall impact, opportunities for screening, active monitoring of progression and management strategies.

The main objectives of our study were to determine lung function in SSc and to explore correlations with SSc phenotypes, early diagnosis of pulmonary involvement and heterogeneity of progression in SSc with ILD.

**Material and Methods:** 85 consecutive patients with SSc from a single center were included. Pulmonary function tests (PFT) e.g. forced vital capacity (FVC), diffusing capacity of the lung for carbon monoxide (DLCO); N-terminal pro-B-type natriuretic peptide (NT-proBNP) and 6-minute walk distance (6MWD) were performed every 6 months. Lung HRCTs were available for fibrosis quantification at baseline and follow-up, as well as ehcocardiography with Doppler.

Data were analyzed for the relationship of DLCO and SSc-related characteristics (clinical, serological, activity, severity) at baseline and follow-ups, and the rate of decline in DLCO was compared across disease subtypes and prognostic groups (Mann-Whitney, chi-squared, Pearson rank correlations, ANOVA) (SPSS v.19, p<0.05).

**Results:** At baseline, about half of patients had ILD by HRCT, while only one third DLCO levels <75% predicted. Impaired DLCO were noticed in diffuse (dcSSc) vs. limited (lcSSc) disease (p<0.001), with lowest DLCO levels in dcSSc (p<0.001). Moreover, patients presenting with ILD (80%), especially clinically symptomatic (dyspnea) (78%), males (62%), and anti-topoisomerase I positive disease (71%) showed significantly decreased DLCO (p<0.01). No patient had pulmonary hypertension.

At follow-ups, up to half cases displayed a decline in pulmonary function (predicted values) consistent with ILD progression. dcSSc had a higher annual rate of decline in FVC and DLCO than those with lcSSc (p<0.001). Changes in DLCO in the previous year predict DLCO change in the subsequent year.

Long-term follow-up evaluation (mean follow up of 5.0 years) also showed a significantly increased risk to DLCO decline in dcSSc vs. lcSSc patients (p<0.001).

**Conclusions:** An altered DLCO is more frequent and more evident noticed in diffuse as compared to limited SSc. All SSc patients should undergo a baseline and regular follow-up DLCO assessment to diagnose and monitor ILD progression in SSc and to improve clinical decision-making.

## P.112

## PROFIBROTIC PROTEINS DOWNSTREAM OF THROMBIN RECEPTOR PAR-1 ARE DECREASED IN LUNG SCLERODERMA FIBROBLASTS FOLLOWING DABIGATRAN TREATMENT

G.S. Bogatkevich, I. Atanelishvili, R.M. Silver

*Medical University of South Carolina, Charleston, USA*

**Introduction:** Activation of the coagulation cascade leading to generation of thrombin has been well documented in various forms of lung injury including systemic sclerosis-associated interstitial lung disease (SSc-ILD). We previously demonstrated that the direct thrombin inhibitor dabigatran inhibits *in vitro* thrombin-induced profibrotic signaling in lung fibroblasts isolated from scleroderma patients. The objective of the present study was to characterize and compare lung fibroblasts from SSc-ILD patients before and after dabigatran treatment.

**Material and Methods:** Lung fibroblasts isolated by bronchoalveolar lavage (BAL) from 14 SSc-ILD patients before and after receiving dabigatran (Pradaxa^®^) 75 mg twice daily for 6 months (ClinicalTrials.gov Identifier NCT02426229) were analyzed. The total RNA was extracted from lung fibroblasts, purified, and subjected to RNA-Seq libraries generation followed by sequencing 20M reads per sample Paired End 150 at Novagene (Sacramento, CA). Differential gene expression analyses were performed using the DESeq2R and the EdgeR packages. Genes with an adjusted p-value < 0.05 were considered as differentially expressed.

**Results:** Dabigatran treatment of SSc-ILD patients resulted in 336 differentially expressed lung fibroblast genes decreasing in expression as compared to basal levels before treatment. Using Kyoto Encyclopedia of Genes and Genomes pathway enrichment analysis, we have determined that among these 336 genes, 129 represent thrombin-regulated signal transduction pathways. We observed a strong downregulation of genes involved in Protease-Activating Receptor-1 (PAR-1) protein activity. The transcripts of G-protein alpha transducing activity polypeptide 2 (GNAT2), regulator of G-protein signaling 4 (RGS-4) and RGS-11, Rho GTP-ase activating protein 6 (GAP-6) and Rho GAP-15, Rho guanine nucleotide exchange factor (GEF-37), Rap GEF-3, cyclin D2, protein kinases C beta (PRKCB) and protein kinase C eta (PRKCH) were reduced. Fibrosis-associated genes, such as smooth muscle alpha-actin (SMA, ACTA2), tenascin C, collagen 1 alpha 1 (COL1A1), collagen 3 alpha1 (COL3A1), collagen 8 alpha 2 (COL8A2), collagen 10 alpha 1 (COL10A1), collagen 5 alpha 1 (COL5A1), fibronectin 1, connective tissue growth factor (CTGF), and procollagen-lysine-2-oxoglutarate-5-dioxygenase-2 (PLOD2) were down regulated following dabigatran treatment as well.

**Conclusions:** Inhibition of thrombin in SSc-ILD patients using low dose dabigatran etexilate restrains PAR-1-activated signal transduction pathways and downregulates profibrotic proteins in lung fibroblasts suggesting it as a new therapeutic approach for the treatment of SSc-ILD.

## 3. Arterial Hypertension

## P.113

## SKIN INVOLVEMENT AND PERIPHERAL VASCULAR SYNDROME IN PATIENTS WITH SYSTEMIC SCLEROSIS AND ASSOCIATED PULMONARY ARTERIAL HYPERTENSION

N. Yudkina, A. Volkov, E. Nikolaeva, I. Kurmukov

*VA Nasonova Research Institute of Rheumatology, Moscow, Russia*

**Introduction:** The most severe course and extremely unfavorable prognosis are patients (pts) with pulmonary arterial hypertension (PAH) associated with systemic sclerosis (PAH-SSc). It was shown that survival in SSc-PAH pts is much worse than in pts with idiopathic pulmonary arterial hypertension (IPAH). Unfavorable outcomes due to late recognition can be explained by predominance of subtle, clinically poor manifest SSc types, especially in terms of cutaneous and vascular syndromes.

**Material and Methods:** 14 pts with SSc sine scleroderma (ssSSc)-PAH were analyzed in comparison with 54 pts with clinically manifest skin involvement SSc-PAH (3 pts with diffuse (dcSSc-PAH) and 51 pts with limited cutaneous involvement (lcSSc-PAH)), and 48 pts with IPAH. Pts’ examination and verification of clinical diagnosis were performed in accordance with current guidelines.

**Results:** Pts with IPAH were younger than both type SSc-PAH – 37 (28; 44), 48 (37; 56) and 54 (48; 62) y, respectively. In SSc-PAH pts with skin involvement and the diagnosis of PAH was established earlier (within 18 (10; 44) mo) than in pts with ssSSc (23 (15; 47) mo), although differences are not statistically significant. The PAH functional class was slightly higher in ssSSc-PAH, than in IPAH and SSc-PAH. Raynaud’s phenomenon (RP) was present in all SSc-PAH pts, although in cutaneous SSc pts digital ischemic lesions were more frequent (51% vs 14%, p=0.03), as well as contractures (53% vs 7%, p=0.006). There were no other differences in clinical features between the groups. Anticentromere antibodies (ACA) were present in 7 (50%) pts with ssSSc-PAH and in 36 (65%) pts with cutaneous SSc. Anti-topoisomerase-I antibodies (anti-Scl-70) were found only in 2 pts with lcSSc. More than 1 type of autoantibodies was detected in the majority of SSc pts. A wide range of antinuclear ABs was found in pts with ssSSc-PAH with prevailing ACA (in 7 pts). 100% pts with ssSSc-PAH met ACR-EULAR 2013 criteria. 5-year survival in ssSSc-PAH was somewhat lower, than in SSc-PAH - 50.6% vs 64.9%, respectively; IPAH pts had the best survival rates of 82.5%, and these differences are close to significant.

**Conclusions:** Clinical features and survival ssSS-PAH are very similar to those in pts with cutaneous SSc-PAH with the exception of skin involvement and associated symptoms (digital ischemic lesions and contractures). Rheumatologists should be aware of such specific features as similar survival rates in cutaneous and ssSSc pts, and late recognition of PAH in pts with ssSSc, as well as its similarity with IPAH.

## P.114

## SUBTYPE OF PULMONARY ARTERIAL HYPERTENSION ASSOCIATED WITH SYSTEMIC SCLEROSIS

N. Yudkina, A. Volkov, E. Nikolaeva, I. Kurmukov

*VA Nasonova Research Institute of Rheumatology, Moscow, Russia*

**Introduction:** Despite the similar pathogenesis and clinical picture, pulmonary arterial hypertension in systemic sclerosis (PAH-SSc) in comparison with idiopathic pulmonary arterial hypertension (IPAH) is characterized by a more severe course, an unsatisfactory response to PAH-specific therapy, a poor survival and a disappointing prognosis. Before the doctors of different specialties there arises the need to distinguish the features of PAH depending on the presence of SSc.

**Material and Methods:** The study included 51 patients with PAH-SSc and 50 patients with IPAH. All patients underwent catheterization of the right heart and pulmonary artery to confirm pulmonary arterial hypertension. The diagnosis of SSc was established in accordance with the classification criteria 2013. Univariate logistic regression was used to calculate the probability (odds ratio (OR) of SSc-associated symptoms.

**Results:** We identified 29 symptoms that were associated with PAH-SSc. The most significant of them, increasing the risk of detection of SSc in patients with PAH, were age over 45 years (OR 9.7, 95% CI 3.9-24.3, p <0.00001), serum uric acid level >387 mcmol/l (OR 3.8, 95% CI 1.5-9.6, p = 0.003), diffusion lung capacity (DLCO) <60% (OR 13.8, 95% CI 4.3-44.7, p <0.00001), ratio of forced vital capacity to DLCO <1.7 (OR 13.0, 95% CI 3.9-42.9, p <0.00001), level of C-reactive protein >2 mg/l (OR 12.9, 95% CI 3.96-41.8, p <0.00001), the presence of pericardial effusion (OR 2.3, 95% CI 1.0-5.1, p = 0.04), mean right ventricular pressure >15.5 mm Hg. (OR 8.9, 95% CI 3.4-23.1, p <0.00001), diastolic right ventricular pressure >4.5 mm Hg. (OR 4.6, 95% CI 1.9-11.2, p = 0.0003). Symptoms that reduce the risk of detecting SSc in patients with PAH include mean pulmonary artery pressure > 55 mmHg. (OR 0.4, 95% CI 0.2-0.9, p = 0.03), pulmonary vascular resistance >12 units Wood (OR 0.4, 95% CI 0.2-0.9, p = 0.02), hemoglobin level >146 g/l (OR 0.3, 95% CI 0.2-0.8, p = 0.007), presence of syncope (OR 0.3, 95% CI 0.1-0.9, p = 0.02).

**Conclusions:** The selection of features of the course of PAH that increase the likelihood of detection of SSc will contribute to the timely diagnosis of SSc, will allow to predict the course of PAH, to approach more carefully the selection and monitoring of the effectiveness of PAH-specific therapy. That will contribute to improving the survival and functional status of patients with PAH-SSc.

## P.115

## ASSOCIATION BETWEEN IRON STUDIES AND PULMONARY HAEMODYNAMICS IN SYSTEMIC SCLEROSIS

A. Sari^1,2^, S.I. Nihtyanova^1^, B.E. Schreiber^3^, G. Coghlan^3^, C.P. Denton^1^, V.H. Ong^1^

^1^*Centre for Rheumatology and Connective Tissue Diseases, University College London Medical School, Royal Free Hospital, London, United Kingdom*, ^2^*Hacettepe University Medical School, Department of Rheumatology, Ankara, Turkey*, ^3^*Department of Cardiology, Royal Free hospital, London, United Kingdom*

**Introduction:** Iron deficiency (ID) is more prevalent in systemic sclerosis (SSc) patients with pulmonary hypertension (PH) than those without and associated with worse prognosis. Few data are available about the relationship between serum iron parameters and pulmonary haemodynamics.

**Material and Methods:** Right heart catheterisation (RHC) reports of SSc patients were retrospectively reviewed. Subjects were included in the study if they had serum iron studies done within 12 months of RHC and were either diagnosed as 1) pulmonary arteral hypertension (PAH, group 1 PH) or 2) no PH. Patients with gastrointestinal (GI) disorders that may cause low iron levels (coeliac disease, vascular ectasia, angiodysplasia, gastrectomy and history for GI bleeding) and glomerular filtration rate below 60 ml/min/1.73m2 were excluded. We recorded serum iron concentration (micromol/L), total iron-binding capacity (TIBC, micromol/L), transferrin saturation (TS, %), ferritin (microg/L), red cell distribution width (RDW, %), as well as mPAP (mmHg) and pulmonary vascular resistance (PVR, dynes/sec/cm-5). Univariable associations were assessed using Mann Whitney test and Spearman’s correlation as appoppriate.

**Results:** We included 122 subjects in the analysis, 84% were female and mean age at RHC was 57 years. The majority (71%) had limited cutaneous SSc and 34% carried anti-centromere antibodies, followed by 17% with anti-Scl70 and 7% with anti-U3RNP.

At RHC, 53/122 (43%) of the patients were diagnosed with PAH. Among them we observed substantially lower levels of serum iron (8.7 vs. 12.1 micromol/L, p<0.001), TS (15.3% vs. 22.5%, p<0.001) and ferritin (68.1 vs.112.5 microg/L, p=0.07) compared to subjects in whom PH was excluded. RDW was significantly higher among PAH patients (16.4% vs. 14.8%, p<0.001), while there was no difference in TIBC (57.4 vs. 54.3 micromol/L, p=0.163).

mPAP showed moderately strong negative correlation with iron concentration (Spearman’s rho= -0.35, p<0.001) and TS (Spearman’s rho= -0.39, p<0.001) and positive correlation with RDW (Spearman’s rho= 0.46, p<0.001).

PVR was significantly inversely correlated with iron concentration (Spearman’s rho= -0.30, p=0.001), TS (Spearman’s rho= -0.37, p<0.001 and ferritin (Spearman’s rho= -0.22, p=0.016), while it showed positive correlation with TIBC (Spearman’s rho= 0.28, p=0.003) and RDW (Spearman’s rho= 0.37, p<0.001).

**Conclusions:** To our knowledge, this is the first study to demonstrate a significant relationship between ID state and haemodynamic measures of SSc-PAH. Our data also support RDW, as an indicator for functional iron deficiency, may serve as a biomarker for severity of pulmonary vasculopathy in SSc.

## P.116

## DELAYED RECOVERY OF ELEVATED PULMONARY ARTERIAL PRESSURE AFTER EXERCISE AS AN EARLY SIGN OF PULMONARY VASCULAR REMODELING LEADING TO PULMONARY HYPERTENSION IN PATIENTS WITH SYSTEMIC SCLEROSIS

K. Sakurai^1^, Y. Yamasaki^1^, K. Suzuki^1^, Y. Asari^1^, T. Suzuki^1^, T. Kiyokawa^1^, Y. Akashi^2^, S. Kou^2^, H. Yamada^3^, K. Kawahata^1^

^1^*Rheumatology and Allergology, St. Marianna University School of Medicine, Kawasaki, Japan*, ^2^*Cardiology, St. Marianna University School of Medicine, Kawasaki, Japan*, ^3^*Center for Rheumatic disease, Seirei-Yokohama Hospital, Yokohama, Japan*

**Introduction:** Pulmonary arterial hypertension (PAH) is caused by irreversible remodeling of pulmonary arteries (PA). It is not clear that reversible vascular damage precedes the irreversible decrease in pulmonary vascular bed in systemic sclerosis (SSc)-associated PAH. To clarify this hypothesis, we investigated characteristics by exercise echocardiography among patients with SSc having normal PA pressure.

**Material and Methods:** We retrospectively studied 36 patients with SSc without severe interstitial lung disease. The patients underwent exercise echocardiography with Master two-step test and showed mean PA pressure (mPAP) <25mmHg by right heart catheterization (RHC). According to the RHC data, patients were divided into 3 groups; those having mPAP <=17mmHg (n =16), 18-20mmHg (n =9), and 21-24 mmHg (n =11). We compared the values of tricuspid regurgitation peak gradient (TRPG) at the following 4 points: at rest, just and at 3 and 5 minutes after the exercise.

**Results:** Mean follow-up period (SD) of the 36 patients [mean age (SD) of 67 (8) year-old] was 62 (32) months. The values of TRPG at rest were comparable between the 3 groups (89% had <40mmHg). The TRPG values just after the exercise were higher in patients with mPAP 18-20 and 21-24 mmHg than in those with <=17mmHg (mean TRPG, 59 vs. 49 mmHg, P =0.0063). Patients with mPAP 18-20 mmHg tended to have higher TRPG (mean, 46 mmHg) at 3 minutes after the exercise, close to that in those with mPAP 21-24 mmHg (mean, 49 mmHg), than those with mPAP <=17 mmHg (mean, 38 mmHg) (P =0.078). Forty-four % of the patients with mPAP 18-20 mmHg had TRPG >=50 mmHg at 3 minutes after the exercise while no patients with mPAP <=17 mmHg had TRPG >=50 mmHg (P =0.01). Among 27 patients having mPAP <=20 mmHg, 5 developed PH (mPAP >20 mmHg) during the follow-up. While 3 of 5 patients (60%) had elevation of TRPG (>50 mmHg) just after but not at 3 or 5 minutes after the exercise at the baseline, 4 (80%) showed an elevation of TRPG at 3 or 5 minutes after the exercise at the onset of pulmonary hypertension.

**Conclusions:** Delayed recovery of elevated PA pressure after exercise may reflect an early sign of vascular remodeling losing reversibility by repeated PA spasm. This phase may represent ‘windows of opportunity’ for treatment before developing irreversible PA remodeling or PAH. Left heart disease might be another possible contributing factor which needs to be studied in the future.

## P.117

## CHANGE IN PHYSICAL FUNCTION IN SYSTEMIC SCLEROSIS PATIENTS WITH INTERMEDIATE/HIGH RISK FOR PULMONARY HYPERTENSION – CLINICAL IMPLICATION OF ENDOTHELIN-RECEPTOR ANTAGONIST TREATMENT

J.W. Park^1^, H.-M. Choi^2^, E.H. Park^3^, H.M. Kwon^4^, S. Cho^1^, E.B. Lee^1^

^1^*Division of Rheumatology, Department of Internal Medicine, Seoul National University College of Medicine, Seoul, South Korea*, ^2^*Division of Cardiology, Department of Internal Medicine, Hallym University College of Medicine, Anyang, South Korea*, ^3^*Division of Rheumatology, Department of Internal Medicine, Seoul National UnivSeoul National University Bundang Hospital, Seongnam, South Korea*, ^4^*Department of Rheumatology, Mediplex Sejong Hospital, Incheon, South Korea*

**Introduction:** The optimal treatment strategy in systemic sclerosis (SSc) patients with pulmonary hypertension (PH) other than isolated pulmonary arterial hypertension (PAH) remains uncertain. In this study, we investigated the clinical factors which affect physical function of patients with SSc and intermediate/high risk for PH.

**Material and Methods:** A total of 62 SSc patients with intermediate/high probability of PH (defined as tricuspid regurgitant jet velocity > 2.8m/sec on baseline echocardiography) were included from a prospective, single centre cohort in South Korea. Physical function of the patients was assessed using 6-minute walk distance (6MWD). Primary outcome of this study was change in 6MWD from the baseline. Clinical factors which affect the outcome were investigated using linear mixed model with a fixed effect for time (in years) and a random effect for the subject.

**Results:** Mean (SD) follow-up duration of the patients was 2.3 (1.1) years. Mean (SD) age of the patient was 58.1 (11.1) years and most (56/62, 90.3%) were female. Thirty-four (54.8%) patients were diffuse subtype. Forty-eight (77.4%) patients concomitantly had interstitial lung disease (ILD) at baseline and mean forced vital capacity (FVC, %) and diffusing capacity of carbon monoxide (DLCO, %) were 75.3 (21.7) and 53.9 (21.0), respectively. Endothelin receptor antagonist (ERA) for PAH was treated in 17 (27.4%) patients.

Mean 6MWD at baseline was 424.0 (87.5) meter. It was not significantly changed over time in the whole population (beta = -5.2 [95% CI -16.1 to 5.8] meter/year). However, univariable analysis showed that ERA treatment for PAH was significantly associated with decrease in 6MWD over time (beta = -34.3 [-58.5 to -10.0] meter/year). This result was consistent in the multivariable model where effects of baseline FVC and 6MWT distance and immunosuppressive treatment for ILD on the outcome were adjusted (adjusted beta = -39.1 [-67.4 to -10.8] meter/year) (Table). Phosphodiesterase type 5 inhibitor treatment was not significantly associated with the change in 6MWD. There were no significant changes in FVC and DLCO during the follow-up.

**Conclusions:** SSc Patients with intermediate/high probability of PH in real world commonly have concomitant ILD. ERA treatment for PAH in such patients could impair the physical function.

**Figure:**
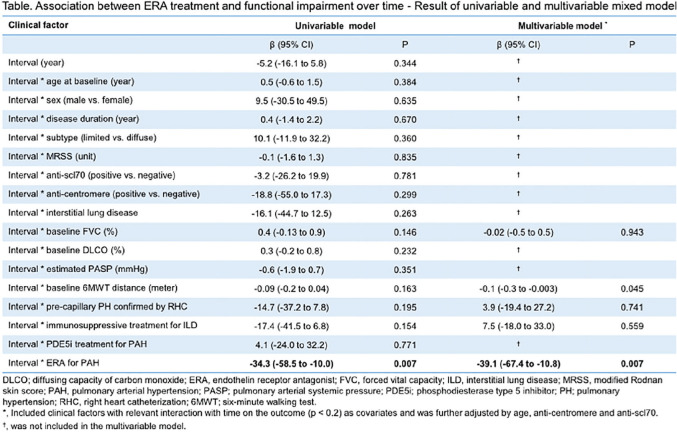


## P.118

## EFFICACY AND SAFETY OF ORAL PROSTACYCLIN RECEPTOR AGONIST SELEXIPAG IN PATIENTS WITH SYSTEMIC SCLEROSIS -ASSOCIATED PULMONARY ARTERIAL HYPERTENSION IN DAILY CLINICAL PRACTICE, A CASE SERIES

J. Lemmers^1^, H. Fretheim^2^, H. Knaapen-Hans^1^, F. van den Hoogen^1^, J. van Haaren-Willems^1^, T. Duijnhouwer^1^, A. van Dijk^1^, C. van den Ende^1^, A.-M. Hoffmann-Vold^2^, M. Vonk^1^

^1^*Radboud University Medical Centre, Nijmegen, The Netherlands*, ^2^*Oslo University Hospital, Oslo, Norway*

**Introduction:** Selexipag added to background therapy improves prognosis of Systemic Sclerosis (SSc)-associated Pulmonary Arterial Hypertension (PAH) in the published pivotal trial by Sitbon et al in 2015. To date information on results of SSc-PAH patients treated with any combination of targeted therapies including selexipag in daily clinical practice is lacking. The objective of this study is to describe the efficacy and safety in all patients with SSc-PAH started on Selexipag between 09-2016 and 06-2018 in two PAH expert centres.

**Material and Methods:** All patients with SSc-associated PAH (WHO group 1) diagnosed by right heart catheterization that initiated selexipag treatment were included. Every 12 weeks we recorded the consensus-based evaluation of treatment of the treating expert team (improved, stabilized, deteriorated). Additionally treatment effect was assessed according to the abbreviated risk assessment, i.e. only non-invasive parameters (functional class, six minute walking distance and NT-pro-BNP level) recorded at baseline and during follow-up. Side effects and adverse events were registered.

**Results:** We included 13 SSc-PAH patients, ten patients were female. Median age was 68 years (IQR 58-75), median SSc disease duration 7.4 years (IQR 4.7-13.5) and median PAH duration 4 years (IQR 2.5-7.5).Two patients discontinued selexipag within 4 weeks due to side effects. The remaining 11 patients had a median follow-up duration of 48 weeks (IQR 24-72). Two patients died, one at 12 weeks caused by right heart failure and one at 48 weeks because of gastro-intestinal bleeding due to angiodysplasias. No previously unrecorded side effects were reported. According to the expert team at 12 weeks 8 out of 11 patients who continued treatment were stabilized or improved. At 24 weeks 9 out of 10 and at 48 weeks 5 out of 7 patients were at least stabilized. At their last moment of follow-up, 3/11 patients had 1 low risk criterion.

**Conclusions:** Adding selexipag to background therapy in a high risk cohort of SSc-PAH patients provides sustained stabilisation of symptoms with an acceptable safety profile. Improvement is reached in only a minority of our patients. Further research examining multiple target therapy, including selexipag, in patients with early SSc-associated PAH is warranted.

## P.119

## APPLYING THE PROPOSED DEFINITION FOR PULMONARY HYPERTENSION IN A SYSTEMIC SCLEROSIS COHORT

D.A. Langli^1^, H. Fretheim^2^, H. Marjavara Inselseth^2^, Ø. Midtvedt^2^, T. Garen^2^, Ø. Molberg^2^, E. Gude^2^, A. Andreassen^2^, A.-M. Hoffmann-Vold^2^

^1^*Innlandet Hospital Trust, Hamar, Norway*, ^2^*Oslo University Hospital, Department of Rheumatology, Oslo, Norway*

**Introduction:** Precapillary pulmonary hypertension (PH) develops in about 15-20% of patients with systemic sclerosis (SSc) and is to date defined by a mean pulmonary arterial pressure (mPAP) >= 25 mmHg and pulmonary artery wedge pressure (PAWP) <= 15mmHg at rest, measured by right heart catheterization (RHC). At the 6th World symposium on Pulmonary hypertension a new hemodynamic definition of PH was proposed, including mPAP >20 mmHg, PAWP <= 15mmHg and pulmonary vascular resistance (PVR) >=3Wood Units (WU) for all forms of pre-capillary PH. Here we explore the effect of these new definitions on the prevalence of precapillary PH in a well-defined and large SSc cohort.

**Material and Methods:** We included all SSc patients in the Oslo University Hospital (OUH) who had conducted at least one RHC. The prevalence of precapillary PH was assessed (1) according to the existing criteria, and (2) according to the proposed definition. Pulmonary arterial hypertension (PAH) and PH due to interstitial lung disease (ILD) were defined as precapillary PH in absence or presence of ILD on high resolution CT scan with </>10 % pulmonary fibrosis, respectively.

**Results:** Of 454 SSc patients, 191 (42%) had performed at least one RHC. Mean age at first RHC was 60 years (SD 11,3), mean follow up from SSc diagnosis was 12 years (SD 8,7); 150 patients (79 %) were female and 149 (78 %) had limited cutaneous SSc. According to the existing criteria, 87 patients (46%) had precapillary PH (60 (31%) PAH and 27 (14%) PH-ILD), giving a prevalence of 60/454 (13%) for PAH and 27/454 (6%) for PH-ILD. Applying the new PH criteria, 33 patients (17%) had mPAP 21-24 mmHg, but only 5 of these fulfilled the proposed definitions of precapillary PH, with an PVR >= 3 WU and PAWP <=15 (3 PAH and 2 PH-ILD). Also, 12 patients (9 PAH and 3 PH-ILD) had PVR <3 WU. Hence, according to the proposed definitions, the prevalence of PAH would be 54/454 (12%) and of PH-ILD 26/454 (6%). Clinical and demographic data of the patients with precapillary PH according to the existing and proposed criteria are shown in the table.

**Conclusions:** The prevalence of PAH and PH-ILD in SSc is not significantly changed by applying the proposed definitions of precapillary PH. However, some patients fulfilling the existing criteria do not fulfill the proposed ones and it is uncertain how to manage these patients.

**Figure:**
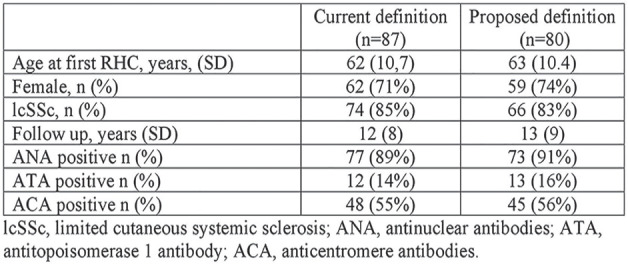


## P.120

## NAILFOLD CAPILLAROSCOPY PARAMETERS AS PREDICTORS OF PULMONARY ARTERIAL HYPERTENSION IN PATIENTS WITH SYSTEMIC SCLEROSIS

N. Klyaus^1^, M. Simakova^2^, A. Maslyanskiy^1^, O. Moiseeva^2^

^1^*Almazov National Medical Research Centre - Department of rheumatology, Saint-Petersburg, Russia*, ^2^*Almazov National Medical Research Centre - Department of cardiology, Saint-Petersburg, Russia*

**Introduction:** Pulmonary arterial hypertension (PAH) is one of the most important complications of systemic sclerosis (SSc). The characteristic features of vascular lesions in SSc is obliterating vasculopathy, which plays an important role in the pathogenesis of PAH . Nailfold capillaroscopy is an established method for the evaluation of the microvessels in patients with SSc. It is determined that the degree of the changes in microvessels reflects the severity of such complication as PAH. Nailfold capillaroscopy may be a useful as non-invasive method for identifying PAH in patients with SSc.

The aim of this study was to assess the possibility of using nailfold capillaroscopy morphological pattern as potential predictor of PAH in patients with SSc.

**Material and Methods:** 101 patients (9 males and 92 females) with SSc were enrolled in the study. To assess the severity of skin changes the modified skin score of G. Rodnan was used. The nailfold capillaroscopy was conducted, the density of the capillary loops and the pattern were estimated, and a semi-quantitative evaluation was carried out. Echocardiography, right heart catheterization were performed as well as the body plethysmography and diffusion capacity of the lung for carbon monoxide (DLCO).

**Results:** The median age was 55 years (Q25-Q75 46-61, respectively). PAH was detected in 27 (26.7%) patients. Patients with verified PAH were significantly older than patients without PAH (58±8 vs 52±11 years, p=0.007). Patients with PAH had significant higher score of the number of alterations according to nailfold capillaroscopy (p=0.009). The capillary density was lower in patients with PAH than patients without PAH (6±1 vs 7±1, p=0.003). Differences in the other nailfold capillaroscopy characteristics between patients of the two groups have not been established.

It was established that decrease in the capillary density less than 7/mm increases the risk of PAH (sensitivity 68.97%, specificity 55.00%, AUC = 0.724, p = 0.0029).

The higher PAH risk was associated with older age, reduced capillary density and lowered DLCO (p<0.001, p<0.001 and p<0.001, respectively).

**Conclusions:** Parameters determined in nailfold capillaroscopy can be used to predict PAH in patients with SSc.

## P.121

## TREATMENT EFFECTS ON OUTCOME IN SYSTEMIC SCLEROSIS PATIENTS WITH MILDLY ELEVATED PULMONARY PRESSURE

H. Fretheim^1^, D.A. Langli^1,2^, H.M. Inselseth^1^, Ø. Midtvedt^1^, T. Garen^1^, Ø. Molberg^1^, E. Gude^1^, A.K. Andreassen^1^, A.-M. Hoffmann-Vold^1^

^1^*Oslo university hospital, 1, Norway*, ^2^*Innlandet Hospital Trust, 2, Norway*

**Introduction:** The new pulmonary hypertension (PH) definitions proposed by the 6th world Symposium on pulmonary Hypertension lowers the limit of mean pulmonary artery pressure (mPAP) to >= 21 mmHg but the impact of treatment in this subgroup on the outcome is unknown. In this study, we aimed to identify patients with systemic sclerosis (SSc) with mPAP 21-24mmHg receiving PAH treatment and assess functional outcomes and mortality compared to treatment-naive patients with mPAP 21-24 mmHg.

**Material and Methods:** Out of 454 SSc patients, 191 (42%) from the Oslo University Hospital had conducted at least one right heart catheterization (RHC). Incident SSc patients with mild precapillary PH defined as mPAP values of 21-24mmHg and pulmonary artery wedge pressure (PAWP) <= 15mmHg at RHC were included. PAH medication regardless of indication at the time of the RHC was registered. To assess the functional outcome, clinical parameters including six minutes walking distance (6MWD) and NT-proBNP at time of the RHC, after 6 and 12 months were assessed. Vital status at study end (September 2019) was available in all patients. Follow-up was defined from the time of RHC to study end.

**Results:** In total, 46/191 (24%) patients had an RHC with mPAP values of 21-24 mmHg and PAWP <= 15mmHg. Of these, 7 (15%) patients were treated with PAH medication (5 patients on bosentan and 2 patients on sildenafil for digital ulcers); 39 (85%) patients were treatment-naive at the time of RHC.

At the time of the RHC, the 6MWD of treated patients and treatment-naive patients was 549m and 443m; NT-proBNP was 71ng/L and 105ng/L. Follow-up data at 6 and 12 months are shown in the table. 1-and 5- year survival in the treated group compared to treatment naive patients was 100% and 86% compared to 92% and 77%, respectively.

**Conclusions:** SSc patients with mildly elevated mPAP on PAH treatment had a trend to increased survival despite a greater reduction in 6MWD than treatment naïve patients. The number of treated patients was low and larger trials are needed to assess the potential benefit of an earlier introduction of PAH specific medication.

**Figure:**
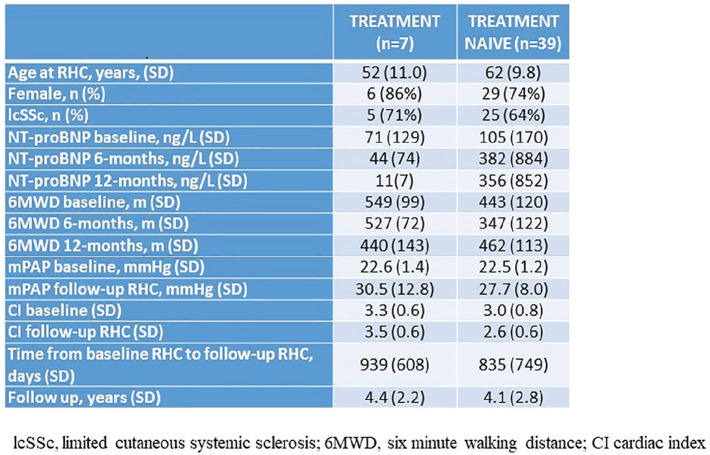


## P.122

## EFFICACY OF TARGETED THERAPIES FOR PULMONARY ARTERIAL HYPERTENSION IN PATIENTS WITH CONNECTIVE TISSUE DISEASES: A SYSTEMATIC REVIEW AND META-ANALYSIS

M. Erdogan, S.N. Esatoglu, V. Hamuryudan, G. Hatemi

*Istanbul University-Cerrahpasa, Medical Faculty, Department of Rheumatology, Istanbul, Turkey*

**Introduction:** Pulmonary arterial hypertension (PAH) is associated with a high rate of mortality and morbidity despite recent advances in treatment. Prognosis is thought to be worse in patients with connective tissue disease-associated PAH (CTD-PAH) compared to idiopathic PAH. In randomized controlled trials (RCT) with different PAH-targeted treatment modalities, patients with idiopathic PAH comprised a significant proportion of the total number of included cases and subgroup analyses of patients with CTD-PAH have yielded conflicting results. We aimed to determine the effect size of each PAH-targeted therapy in patients with CTD-PAH and to compare s with idiopathic-PAH.

**Material and Methods:** We performed a systematic literature search using PubMed from its inception to March 2019 without language restriction. Randomized controlled studies of PAH-targeted therapies including patients with CTD-PAH were eligible. The mean (SD) change in 6-minute walk distance (6MWD) over the study period and all-cause mortality were the main outcomes that were selected for analyses. We planned to contact study authors for missing data. Risk ratio (RR) and standardized mean difference (SMD) were calculated using Review Manager 5.3.

**Results:** The literature search yielded 1167 articles. Fifteen studies that met the inclusion criteria were identified. Eight of them provided data on at least one of our determined outcomes (mortality in 7 studies and 6MWD in 2 studies) for patients with CTD-PAH. The risk ratios for all-cause mortality across the included studies are shown in the Figure. The difference in all-cause mortality was not significantly different for any of the PAH-targeted therapies compared to the control group in that study. There was a significant difference in mean change in 6MWD between sitaxentan vs placebo arms (SDM= 0.95 (95% CI: 0.18 to 1.72) whereas the difference was not significant between riociguat and placebo arms (SDM= 0.36 (95% CI: -0.1 to 0.82).

**Conclusions:** Our limited and preliminary analyses showed that PAH-targeted therapies do not seem to be associated with decreased mortality in patients with CTD-PAH, whereas a significant benefit may be obtained on 6MWD.

**Figure:**
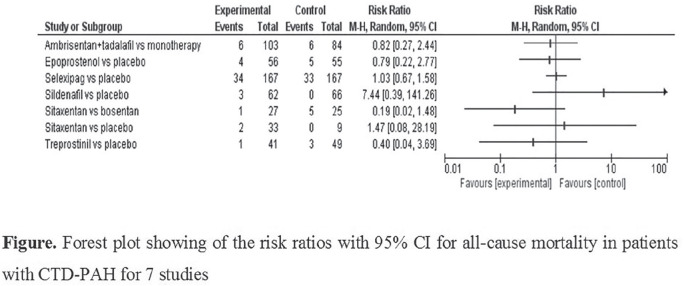


## P.123

## COMPARISON OF DIFFERENT PULMONARY HYPERTENSION SCREENING ALGORITHMS IN PATIENTS WITH SYSTEMIC SCLEROSIS

M. Erdogan^1^, B. Kilickiran Avci^2^, Y. Ersoy^1^, C. Ebren^2^, Z. Ongen^2^, G. Ongen^3^, V. Hamuryudan^1^, G. Hatemi^1^

^1^*Istanbul University-Cerrahpasa, Medical Faculty, Department of Rheumatology, Istanbul, Turkey*, ^2^*Istanbul University-Cerrahpasa, Medical Faculty, Department of Cardiology, Istanbul, Turkey*, ^3^*Istanbul University-Cerrahpasa, Medical Faculty, Department of Chest Diseases, Istanbul, Turkey*

**Introduction:** Pulmonary hypertension (PH) is an important cause of morbidity and mortality in patients with systemic sclerosis (SSc). Different screening algorithms have been proposed for identifying patients who have a high probability of PH and require right heart catheterization (RHC), which is the gold standard for diagnosing PH. We aimed to compare the performance of PH screening algorithms in our patients with SSc

**Material and Methods:** Forty-eight consecutive pts, fulfilling ACR/EULAR 2013 SSC criteria, were screened for PH using the, 2015 ESC/ERS, DETECT and ASIG algorithms.Pulmonary function tests (PFT), diffusing capacity of the lung for carbon monoxide (DLCO), trans-thoracic echocardiography, serum NT-proBNP, uric acid assay and high-resolution computed tomography (HRCT) were performed as needed.Pts with known PH, severe interstitial lung disease and severe left ventricular dysfunction (LVD) were not included. RHC was performed in all patients with positive screening according to any one of the screening algorithms. Pts with PH were classified according to the updated PH classification criteria. Sensitivity and specificity of the 3 screening algorithms were evaluated according to the established cut-off value of 25 mmHg for mean systolic pulmonary artery pressure and for the recently proposed cut-off value of 20 mmHg.

**Results:** Among the 69 SSc pts, 27 were excluded due to ILD(n=6), LVD(n=6), already diagnosed PH(n=4) no measurable TRV(n=5) and lung cancer(n=2), pulmonary embolism(n=1) and nephrotic syndrome(n=1). Among the remaining 42 patients,17 required RHC according to at least one of the screening algorithms(Table 1). Number of patients who had suspected pulmonary hypertension and required RHC according to ESC/ERS 2015, DETECT and ASIG were 7 (%17),13(%31), and 12 (%29) respectively(Figure 1).PH was present in 3pts according to the 25-mmHg cut-off (Group 2 in 2, Group 3 in 1) and in 9pts according to the 20-mmHg cut-off (Group 1 in 5, Group 2 in 3, Group 3 in 1).The sensitivity and specificities were presented in Table 2. ASIG and DETECT had better sensitivity for 25-mmHg cut-off and was better with ASIG for 20 mmHg cut-off.The specificity was better with ESC/ERS for both cut-off values.

**Conclusions:** The ASIG algorithm has a better sensitivity and ESC/ERS algorithm has a better specificity for detecting PH in patients with SSc.A limitation of this study was that RHC was not performed in patients who did not fulfill criteria according to any of the screening algorithms.The sensitivities may be lower than what we propose if there are patients with PH who are asymptomatic and not captured with any of the algorithms.

## P.124

## SCREENING FOR THE EARLY DETECTION OF PULMONARY ARTERIAL HYPERTENSION (PAH) IN PATIENTS WITH SYSTEMIC SCLEROSIS (SSC): A META-ANALYSIS OF LONG-TERM OUTCOMES

Z. Brown^1^, D. Hansen^2^, W. Stevens^2^, S. Proudman^3^, M. Nikpour^1^

^1^*Department of Medicine, The University of Melbourne at St. Vincent’s Hospital, Melbourne, Australia*, ^2^*Department of Rheumatology, St. Vincent’s Hospital, Melbourne, Australia*, ^3^*Department of Rheumatology, Royal Adelaide Hospital, Adelaide, Australia*

**Introduction:** One of the major contributors to mortality in patients with SSc is PAH. International recommendations advise annual screening for the early detection of PAH in all patients with SSc.

**Material and Methods:** Manuscripts published until 12th March 2019 were identified through searching Medline, Embase and Cochrane Central Register of Controlled Trials and Database of Systematic Reviews. Eligible studies included abstracts or full reports investigating patients with SSc undergoing screening by any protocol to detect PAH. Risk of bias was assessed with reference to the QUADAS-2 tool.

The primary outcome was survival (reduced mortality) in patients with SSc-PAH identified by screening vs those identified through usual practice, and secondary outcomes were parameters which are included in risk stratification scores (WHO functional class, haemodynamic parameters (PVR, mPAP, CI), 6-minute walk distance (6MWD), respiratory function parameters (RFT), and serum NT-proBNP) at PAH diagnosis.

Mean difference (MD) with 95% confidence intervals (CI) was calculated for continuous variables. We used a random effects model and for pooled analyses, we quantified statistical heterogeneity using the I2 statistic. Significant statistical heterogeneity was defined by I2 greater than 25%. For the meta-analysis of time-to-event data (mortality) we used the methods described by Tierney J et al.

**Results:** The review resulted in 525 unique citations. Six manuscripts met criteria for meta-analysis. Meta-analysis showed improved survival in patients with SSc-PAH diagnosed as a result of screening. Two studies (total participants 192) demonstrated reduced mortality risk, Hazard Ratio (HR) 0.31 (95% CI 0.18 – 0.53, I2 = 0%, p < 0.0001) in screened patients compared to an unscreened cohort (Figure 1).

Three studies (222 participants) reported a statistically significant difference in 6MWD in patients diagnosed with SSc-PAH favouring screening compared to those without screening. The mean difference was 77.96m (95% CI 40.47 to 115.45, I2 = 0%, p < 0.0001), which is greater than the minimal clinically important difference.

There were also differences in the other parameters included in risk stratification assessments, although these did not reach statistical significance.

**Conclusions:** This meta-analysis demonstrates the long-term benefit of systematically screening patients with SSc for the early detection of PAH. While a contribution to the lower mortality of screened cohorts by lead-time bias cannot be excluded, the size of the reduction suggests a benefit from early therapy. Screened patients were also more likely to have more favourable risk stratification criteria, particularly 6MWD, at the time of PAH diagnosis when compared to cohorts who were not screened.

**Figure:**
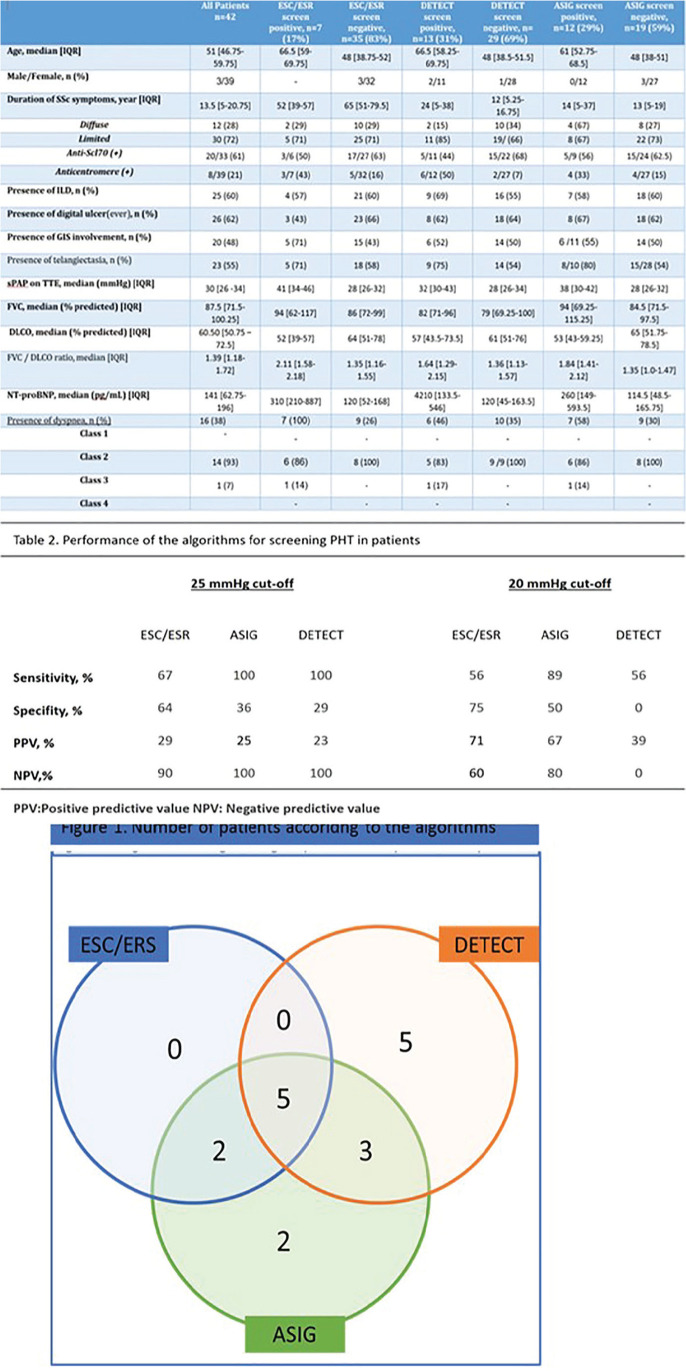


**Figure:**
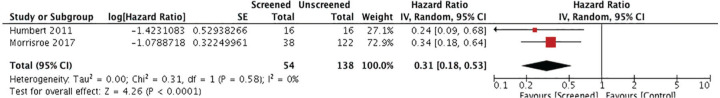


## P.125

## RETROSPECTIVE EVALUATION OF THE ASIG AND DETECT ALGORITHMS FOR THE EARLY DETECTION OF PULMONARY ARTERIAL HYPERTENSION (PAH) IN SYSTEMIC SCLEROSIS (SSC)

Z. Brown^1^, D. Hansen^2^, M. Wilson^2^, L. Mcwilliams^3^, L. Spargo^3^, J. Walker^4^, W. Stevens^2^, S. Proudman^3^, M. Nikpour^1^

^1^*Department of Medicine, The University of Melbourne at St. Vincent’s Hospital, Melbourne, Australia*, ^2^*Department of Rheumatology, St. Vincent’s Hospital, Melbourne, Australia*, ^3^*Department of Rheumatology, Royal Adelaide Hospital, Adelaide, Australia*, ^4^*Immunology, Allergy and Arthritis Department, Flinders University at Flinders Medical Centre, Adelaide, Australia*

**Introduction:** SSc is a chronic multisystem autoimmune disease with a high burden of mortality. One of the major contributors to mortality is PAH, which affects up to 10% of individuals. Current international recommendations advise annual screening for PAH incorporating transthoracic echocardiography (TTE), the DETECT algorithm or by combining respiratory function tests (RFT) parameters and serum N-terminal brain natriuretic peptide (NT-proBNP) as in the Australian Scleroderma Interest Group (ASIG) algorithm.

We sought to retrospectively evaluate the performance of the ASIG and DETECT PAH screening algorithms in a cohort of Australian patients SSc patients.

**Material and Methods:** The Australian Scleroderma Cohort Study (ASCS) is a prospective registry which includes annual clinical assessment, TTE, RFT and stored serum samples enabling evaluation of variables required for both algorithms. Algorithm performance was retrospectively evaluated in patients attending the Royal Adelaide Hospital who had undergone right heart catheterisation (RHC). PAH was defined as mPAP >= 25mmHg, PAWP <= 15mmHg and PVR >= 3 Woods Units.

**Results:** Between 2015 to 2019, 264 patients meeting ACR/EULAR 2013 classification criteria for SSc who had undergone at least one complete screening assessment were identified. Twenty-three patients (8.7%) were diagnosed with PAH as a result of screening. There were no significant differences in autoantibody profile or disease subtype between patients with PAH and those without.

Evaluating only patients who had undergone RHC, the ASIG algorithm had a sensitivity of 100% (95% CI 79.4 - 100%), specificity of 42.4% (95% CI 25.5 - 60.8%), positive predictive value (PPV) of 45.7% (95% CI 28.8 - 63.3%) and negative predictive value (NPV) of 100% (95% CI 76.8 - 100%) for detection of PAH. The DETECT algorithm had a sensitivity of 95% (95% CI 75.1 - 100%), specificity of 18.2% (95% CI 7 - 35.5%), PPV of 41.3% (95% CI 27 - 56.8%) and NPV of 85.7% (95% CI 42.1 - 99.6%).

Adjusting for indications for RHC in patients who had not undergone RHC, by using variables shown to be predictive of referral for RHC (sPAP, DLCO, WHO functional class, and right ventricular function), the ASIG and DETECT algorithms respective performances were sensitivity 100% vs 83%, specificity 58% vs 24%, PPV 38% vs 30% and NPV 100% vs 78%.

**Conclusions:** The ASIG and DETECT algorithms have high sensitivity to detect PAH in patients with SSc. Adjusted for selection bias, the ASIG algorithm outperformed the DETECT algorithm. This supports the use of the ASIG algorithm for screening patients with SSc for PAH.

## P.126

## RETROSPECTIVE VALIDATION OF THE ESC/ERS RISK PREDICTION MODEL IN SYSTEMIC SCLEROSIS (SSC) PATIENTS WITH NEWLY DIAGNOSED PULMONARY ARTERIAL HYPERTENSION (PAH)

Z. Brown^1^, D. Hansen^2^, M. Wilson^2^, J. Roddy^3^, G.-S. Ngian^4^, J. Sahhar^4^, K. Morrisroe^1^, J. Walker^5^, S. Proudman^6^, W. Stevens^2^, M. Nikpour^1^

^1^*Department of Medicine, The University of Melbourne at St. Vincent’s Hospital, Melbourne, Australia*, ^2^*Department of Rheumatology, St. Vincent’s Hospital, Melbourne, Australia*, ^3^*Department of Rheumatology, Fiona Stanley Hospital, Perth, Australia*, ^4^*Department of Rheumatology, Monash Health, Melbourne, Australia*, ^5^*Immunology, Allergy and Arthritis Department, Flinders University, Adelaide, Australia*, ^6^*Rheumatology Department, Royal Adelaide Hospital, Adelaide, Australia*

**Introduction:** The emergence of prognostic risk stratification tools has transformed therapeutic strategy for PAH. We retrospectively evaluated the ESC/ERS PAH risk stratification criteria in SSc-PAH patients enrolled in the Australian Scleroderma Cohort Study (ASCS).

**Material and Methods:** ASCS data were used to analyse Kaplan-Meier (KM) survival curves from PAH diagnosis to PAH-related mortality according to risk status at baseline and at first follow-up within 2 years.

Patients were classified according to the number of ESC/ERS ‘low-risk’ criteria present, including WHO FC I - II, 6MWD > 440m, no progression of symptoms, right atrial pressure <8mmHg, and cardiac index > 2.5 L/min/m2.

**Results:** Between 2008-2018, 109 patients met the ACR/EULAR criteria for SSc and were diagnosed with SSc-PAH. Mean disease duration of SSc at PAH diagnosis was 9.69 years. Mean time from PAH diagnosis to PAH-related mortality was 4.13 years (SD 2.88).

At baseline, 20.2% of patients had >= 2 low-risk criteria, 34.9% had one low-risk criterion and 50% had no low-risk criteria. There was no significant difference in survival between patients with >= 2 low-risk criteria at baseline and those with no low-risk criteria (log rank KM p = 0.075), however the Hazard Ratio (HR) for mortality with >= 2 low-risk criteria at baseline was significantly lower compared with patients with no low-risk criteria (HR 0.26, 95% CI 0.08 - 0.88, p = 0.030). Long-term survival was significantly better in patients who had >= 2 low-risk criteria compared to those with no low-risk criteria at first follow-up (KM p = 0.002), (HR of 0.15, 95% CI 0.05 - 0.42, p < 0.01). Long-term survival was also significantly better in patients who at first follow-up were in low-risk profile or who had improved from baseline (defined as ‘improved risk’), compared to those who remained high-risk or who lost a low risk criterion (defined as ‘increased risk’) (KM p = 0.005), (HR for morality with ‘increased risk’ 3.13, 95% CI 1.35 - 7.25, p = 0.008).

**Conclusions:** This analysis validates the use of the ESC/ERS risk stratification criteria to predict mortality in SSc-PAH patients when applied at baseline and at first follow-up within 2 years of PAH diagnosis.

**Figure:**
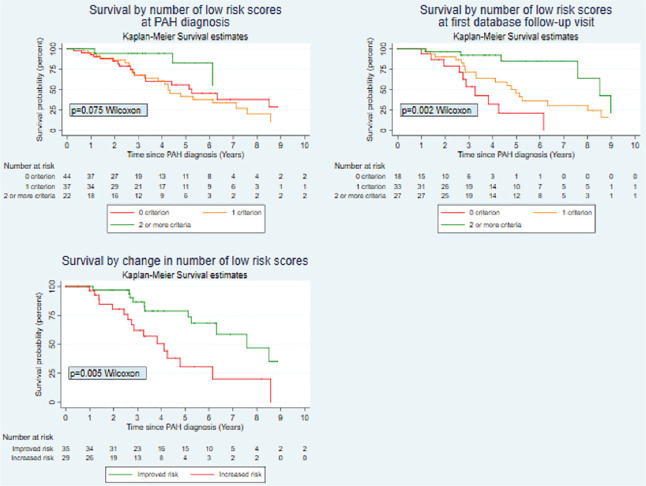


## P.127

## FINDINGS OF EXERCISE ULTRASOUND CARDIOGRAPHY IN PRE-PAH PATIENTS WITH SYSTEMIC SCLEROSIS ARE CORRELATED WITH EXTENT OF CAPILLARY LOSS DETECTED BY NAILFOLD VIDEOCAPILLAROSCOPY

K. Ashihara^1^, T. Naganawa^1^, M. Hoshino^2^, Y. Kato^2^, S. Fukaya^1^, S. Yoshida^1^, H. Yasuoka^1^

^1^*Fujita Health University School of Medicine.Division of Rheumatology, Department of Internal Medicine., Toyoake, Japan*, ^2^*Fujita Health University School of Medicine, Department of Cardiology, Toyoake, Japan*

**Introduction:** Systemic sclerosis (SSc) is characterized by excessive fibrosis, injuries in vasculature, and production of autoantibodies. Patients with SSc are complicated with various organ involvements, which can give a great impact on their prognosis. Of these, pulmonary arterial hypertension (PAH) is one of leading causes of death and the determinant of prognosis in SSc. And early diagnosis is an important key to improve their prognosis. The nailfold videocapillaroscopy (NVC) is a non-invasive modality which can evaluate the status of microvasculature of patients with SSc. However, the significance of NVC in patients with “pre-clinical” PAH was unclear.

**Material and Methods:** Nineteen patients with SSc enterd the study.All the patients fulfilled the 1980 preliminary classification criteria for SSc. The diagnosis of PAH was based on the definition in 2015 ESC/ERS guideline. The NVC and transthoracic ultrasound cardiography (UCG) was performed, and exercise loading was done. Ejection fraction, E/e’, velocity of tricuspid regurgitation jet (TR jet), tricuspid annular plane systolic excursion (TAPSE) were examined in a supine position. Tricuspid regurgitation pressure gradient (TRPG) was calculated using velocity of TR jet based on Bernoulli principle. Exercise loading was started at 25 Watt, and then increased 25 Watt every 3 minutes and TRPG at maximum exercise was also calculated. Parameters of NVC were compared with those of UCG.

**Results:** Male to female ratio was 4:15, mean age 63 ± 13 years, and mean disease duration was 138 ± 109 months. Ratio of limited skin type was 79 %. 37 % were with ILD and 11 % were with PAH confirmed by RHC. There is no correlation between TRPG at rest and the density of capillaries. However, there is a significant, negative correlation between TRPG at exercise and the capillary density (r = 0.57, P < 0.02). Furthermore, the same result was observed even with seventeen “pre-PAH” cases (r = 0.67, P < 0.04).

**Conclusions:** Our study suggests that the finding of NVC, especially the extent of capillary loss, is correlated with disease process of PAH, even subclinically, and may predict the progression to PAH in SSc. Since NVC is non-invasive, NVC can detect earlier PAH candidates and contribute to the improvement of prognosis.

## 4. Cardiac

## P.128

## MEAN PLATELET VOLUME AS A POTENTIAL BIOMARKER OF IMPAIRED RIGHT VENTRICULAR FUNCTION IN PATIENTS WITH SYSTEMIC SCLEROSIS

S. Jaksic Jurinjak^1,5^, S. Tomasinec^2,5^, M. Lucijanic^3,5^, D. Delic-Brkljacic^4,5^, M.-I. Culo^2,5^, J. Morovic-Vergles^2,5^

^1^*University Hospital Dubrava, Department of Cardiology, Zagreb, Croatia*, ^2^*University Hospital Dubrava, Department of Clinical Immunology, Allergology and Rheumatology, Zagreb, Croatia*, ^3^*University Hospital Dubrava, Department of Hematology, Zagreb, Croatia*, ^4^*Clinical Hospital Center Sestre milosrdnice, Cardiology Clinic, Zagreb, Croatia*, ^5^*Universitiy of Zagreb, School of Medicine, Zagreb, Croatia*

**Introduction:** Myocardial involvement, often initially clinically asymptomatic, is an important prognostic factor in patients with systemic sclerosis (SSc). Cardiac manifestations of SSc can be a result of primary myocardial involvement, such as myocardial fibrosis, or secondary phenomenon due to pulmonary arterial hypertension. Impaired echocardiographic parameters of right ventricular (RV) function, signs of RV dysfunction due to myocardial involvement or pulmonary arterial hypertension in SSc are predictors of poor outcome in these patients. Therefore, detection of potential markers is beneficial. Mean platelet volume (MPV) is an indicator of platelet activation and, combined with the fact that multiple pathophysiologic processes might affect platelet volume, it has been associated with many cardiovascular diseases, heart failure, and progression of vascular damage and fibrosis.

The aim of this study was to evaluate echocardiographic parameters of right ventricular function in patients with SSc without pulmonary hypertension and its correlation to MPV.

**Material and Methods:** 27 consecutive SSc patients underwent echocardiography with tissue Doppler imaging to assess RV function. 19 study SSc patients without echocardiographic signs of pulmonary hypertension were investigated at baseline and prospectively in three consecutive visits of one-year follow-up. Echocardiographic measurements of RV function and hematological parameters, including MPV, were assessed at each visit (0-month visit, 4-month visit, 8-month visit, 12-month visit).

**Results:** Mean age was 54.3 ±12.4 years; all patients were females. Mean baseline MPV was 7.8 ±1.2 fL, and it increased during the follow-up period (P<0.001 for linear trend of increase). Besides higher MPV being related to lower platelet count, no consistent associations were present between MPV and investigated hematological parameters at different time points. During the one-year follow-up time frame, when comparing changes, a negative correlation was found of increasing values of MPV and lower tissue Doppler tricuspid annular systolic velocity (TDIs,) and pulmonary valve acceleration time (PVAcct), (P<0.044) and (P<0.005) respectively.

**Conclusions:** Results of our study showed that patients with SSc experienced an increase of MPV over one-year follow-up and had a decrease of TDIs, an echocardiographic marker of systolic RV function, and PVAcct, a secondary indicator of elevated pressure in pulmonary arteries. MPV significantly changed during the follow-up period. This can suggest that MPV might be a helpful indicator of impaired cardiorespiratory function and right ventricular function in patients with systemic sclerosis without pulmonary hypertension. These observations need to be evaluated on larger cohorts of patients during a longer period, but MPV may represent a useful biomarker of right ventricular involvement in systemic sclerosis.

## P.129

## CORONARY ARTERY CALCIFICATIONS ARE ASSOCIATE TO HEART ARRHYTHMIA BUT NOT TO CARDIOVASCULAR EVENTS IN SYSTEMIC SCLEROSIS PATIENTS

C. Rotondo, S. Sciacca, L. Lops, A. Corrado, F.P. Cantatore

*Department of Medical and Surgical Sciences - Rheumatology Unit, University of Foggia, Foggia, Italy*

**Introduction:** Cardiovascular (CV) system involvement is a frequent complication of systemic sclerosis (SSc). An increased subclinical coronary atherosclerosis has been observed in these patients (SSc-pts), probably due to vasculopathy and endothelial cell injury. The aim of this study is to assess the presence of coronary artery calcifications (CC) in SSc-pts and to evaluate if the CC are associated to higher risk of cardiovascular events (CVE) or heart dysfunction.

**Material and Methods:** A total of 35 (female: 31; male: 4) SSc outpatients (mean age 60,3 ± 11 ys and disease duration of 12,8±9 ys), who fulfilled ACR/EULAR 2013 SSc classification criteria, were recruited. Clinical evaluation included assessment of CC by means of computed tomography; body mass index; lipid profile; electrocardiogram abnormalities, echocardiographic measurements. The data were collected retrospectively.

**Results:** CC was found in 13 (37%) SSc-pts. The CC-pts was significantly older than those without CC (69,7±8,5 years vs 54,7±9,8 ys; p=0,0001). Furthermore, CC-pts had higher frequency of CENP-B positivity (46%; p=0,011), pulmonary arterial hypertension (PAH) (25% vs 0%; p=0,014), dyslipidemia (38,5% vs 4,5%; p=0,010), arterial hypertension (61,5% vs 13,6%), diabetes ((23% vs 0%; p=0,018) and a trend to a longer disease duration (15±11,3 ys vs 11,5±7,3 ys; p=0,271). Considering only the pts with age ranged between 35 and 69 ys, even though the cardiovascular risk was higher in CC-pts than those without (8,9±5,9 vs 2,8±3,3), just one CC-pts developed myocardial infarction during the follow-up. The odds ratio (OR) for CVE was 0,35 in CC-pts. Conversely, we noted a higher rate of heart arrhythmia (including extrasystoles, tachyarrhythmia and atrial fibrillation) in CC-pts (38,5%; p=0,36; OR: 6,2 (CI95%: 0,99-39). By binary regression analysis the CC is an independent factor associated to the heart arrhythmia (p=0,05). No differences between CC-pts and pts without CC were found in capillaroscopic scleroderma patterns, interstitial lung disease, subcutaneous calcinosis.

**Conclusions:** The coronary artery calcifications in SSc seems to be associated with older age and the presence of atherosclerosis risk factors as dyslipidemia, diabetes and arterial hypertension. Anyway, an important role of vasculopathy related to SSc in the pathogenesis of coronary artery calcifications should be take into account, due to the presence of higher expression of CENP-B positive and PAH in CC-pts. The CC in SSc-pts should be considered a main factor associated to high risk to develop heart arrhythmias; so, a strictly electrocardiogram assessment is suggested. Lastly, no association between coronary artery calcification and cardiovascular events was noted.

## P.130

## FACTORS ASSOCIATED WITH CARDIAC INVOLVEMENT IN SYSTEMIC SCLEROSIS. EVALUATION IN A COHORT OF 139 PATIENTS

A.J. Raschia, P. Sansinanea, M.V. Martire, L. Garcia

*HIGA San Martin - Department of Rheumatology, La Plata, Argentina*

**Introduction:** Cardiac involvement in Systemic Sclerosis clinically appears in 15% of patients. However, according to autopsy reports, subclinical involvement could reach 81%. Cardiac manifestations include pericardial effusion, diastolic dysfunction of both ventricles, ventricular hypertrophy, heart failure, coronary, valvular and arterial disease, arrhythmias/conduction disturbances. Cardiac involvement is a factor of poor prognosis and is associated with increased morbidity and mortality

**Objective:** To determine the frequency of cardiac involvement in patients with Systemic Sclerosis and to evaluate the factors associated with this manifestation

**Material and Methods:** Patients with systemic sclerosis (SS) according to ACR 1980 and / or ACR / EULAR 2013 classification criteria were included from 1983 to July 2019. Demographic, clinical and laboratory variables and causes of death were analyzed. The following cardiac manifestations were evaluated: diastolic dysfunction, systolic dysfunction, pericardial effusion, arrhythmias, dilated right cavities and ischemic heart disease. Descriptive statistics were performed, quantitative variables were expressed as mean, Standard Deviation (SD) or median and interquartile range (IQR), and qualitative variables as percentages (%). The characteristics of the patients with and without cardiac compromise were compared by Student Test or Mann Whitney and Chi Square or Fisher’s Exact Test. Binary logistic regression was performed using the presence of cardiac involvement as dependent variable

**Results:** A total of 139 patients were included, of which 121 (87%) are women, with a mean age at diagnosis of 47.5 years (SD 15). The evolution of the disease was 4 (IQR 1/10) years. Of the total of patients, 48.2% had some type of cardiac compromise, being 66.7% male, with an average age of 50.46 (± SD 14.23). Regarding cardiac manifestations, 26 patients (18.7%) had diastolic dysfunction, 16 (11.5%) ischemic heart disease, 10 (7.2%) dilated right cavities, 9 (6.5%) pericardial effusion, 7 (5%) systolic dysfunction and 6 (4.3%) arrhythmias.

Table 1 shows the bivariate analysis. Older age, pulmonary involvement, presence of pulmonary arterial hypertension (PAH) and diffuse subtype were significantly associated with the development of heart disease. In the multivariate analysis, PAH was the only variable independently associated with cardiac involvement [OR 6.44 (95% CI 2.04-20.35); p 0.001].

**Conclusions:** Cardias involvement was frequently detected, predominantly in the form of diastolic dysfunction. PAH was independently associated with the development of cardiac involvement.

Therefore, we consider vital the screening of this manifestation, even in asymptomatic cases

**Figure:**
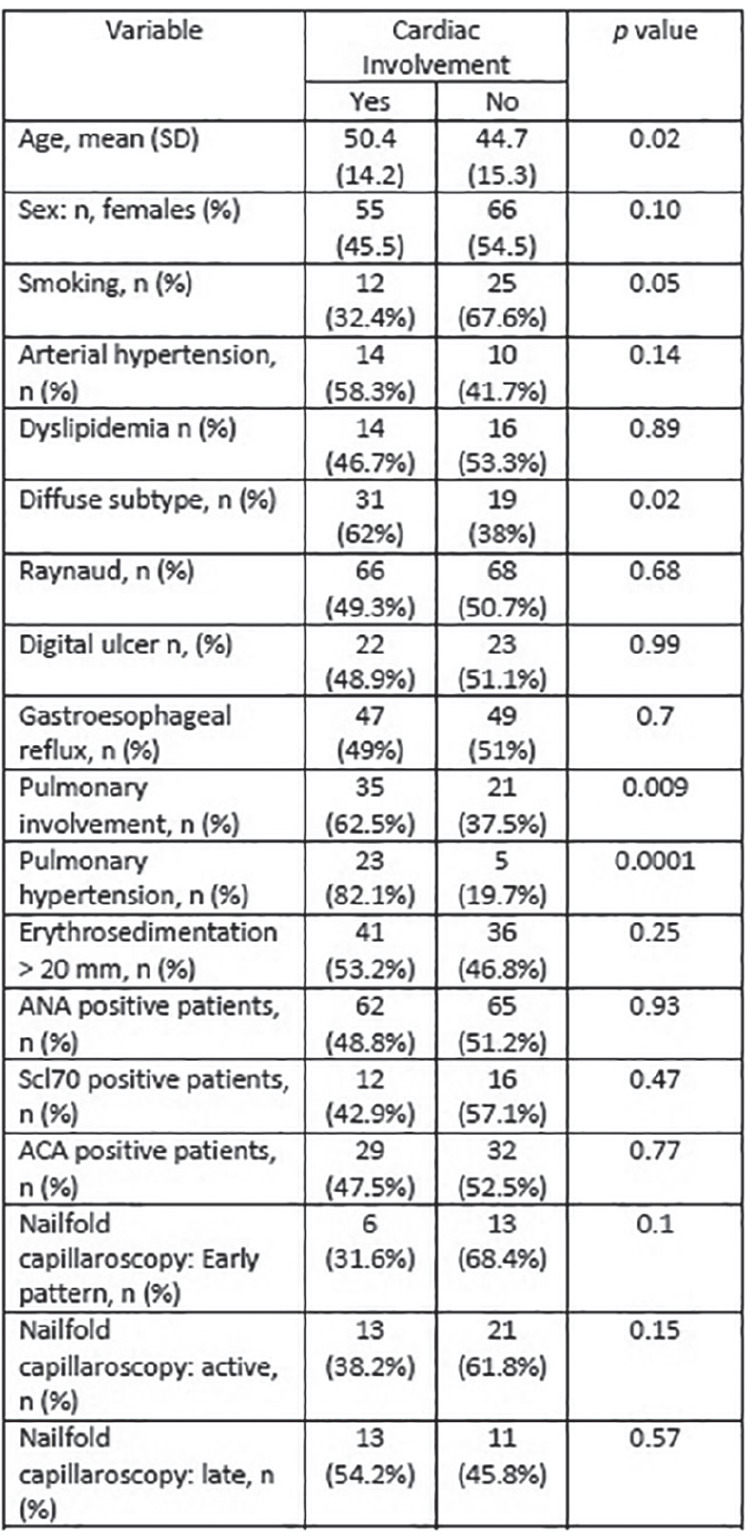


## P.131

## RIGHT HEART CATHETERISATION AND CARDIAC MAGNETIC RESONANCE IMAGING FINDINGS IN SYSTEMIC SCLEROSIS PATIENTS REFERRED FOR AUTOLOGOUS HAEMATOPOIETIC STEM CELL TRANSPLANT

R. Penglase^1^, A. Jabbour^1,2^, E. Kotlyar^1,2^, L. Girgis^1,2^, D. Ma^1,2,3^, J. Moore^1,2,3^

^1^*St Vincent’s Hospital, Sydney, Australia*, ^2^*University of New South Wales, Sydney, Australia*, ^3^*St Vincent’s Centre for Applied Medical Research, Sydney, Australia*

**Introduction:** Autologous haematopoietic stem cell transplantation (AHSCT) is the current gold standard treatment for severe SSc. Nonetheless, transplant-related mortality (TRM) remains a key issue, with particular regard to cardiac toxicity. As such, right heart catheterisation (RHC) and cardiac magnetic resonance imaging (CMR) are both recommended screening tests prior to AHSCT with the intention of identifying those at greatest risk.

**Material and Methods:** This prospective cohort study analysed data from all patients who were referred to our centre for consideration of AHSCT using standard cyclophosphamide (CYC) dose conditioning (200mg/kg) since 2013. All patients underwent either RHC or both RHC and CMR. Patients were subsequently categorised according to whether they received AHSCT or not. Cardiovascular risk factors, RHC and CMR findings were compared between groups.

**Results:** 49 patients with severe SSc were considered for AHSCT. 10 patients were excluded for non-cardiac reasons or refused therapy. 19 patients were deemed ineligible for standard CYC dose AHSCT due to cardiac disease: 7 patients with prior cardiac disease and 12 patients found to have an abnormal RHC or CMR in the screening process. Compared to the AHSCT group (n=20), the ineligible group were significantly older (age 45 vs 53, p=0.008), had a greater proportion of males (20% vs 63%, p=0.006) and had a history of smoking (15% vs 68%, p = 0.0006). When patients with resting mPAP >/= 25mmHg were removed from the ineligible group, this group still had significantly higher pulmonary pressures on RHC, including post fluid challenge. On CMR, a pericardial effusion was seen in 60% of all patients. The ineligible group had significantly higher rates of post-gadolinium enhancement (27% vs 67%, p= 0.047) and regional wall motion abnormality (0% vs 50%, p=0.03). There was 1 TRM in the AHSCT group caused by acute cardiomyopathy; this patient had a normal RHC and CMR, but was notably an ex-smoker and had a BMI >30.

**Conclusions:** SSc patients considered ineligible for AHSCT were more likely to have existing cardiovascular risk factors, especially smoking. In adjusted analyses, this group had higher pulmonary pressures and rates of CMR abnormalities. RHC and CMR proved critical investigations in this cohort, identifying subclinical cardiac disease in >30% of referred patients, reinforcing current screening recommendations. Significant caution is advised when a history of smoking is identified. Further research is needed to optimise AHSCT protocols to reduce adverse cardiac outcomes. Extending treatment to SSc patients with identified cardiac disease remains an unmet need.

## P.132

## ELECTROCARDIOGRAPHIC ABNORMALITIES AND PULMONARY INVOLVEMENT IN A MONOCENTRIC COHORT OF PATIENTS WITH SYSTEMIC SCLEROSIS

G. Pellegrino, F. Facioni, K. Stefanantoni, C. Angelelli, C.D. Vizza, R. Badagliacca, S. Morelli, G. Valesini, V. Riccieri

*Sapienza University, Rome, Italy*

**Introduction:** Cardiac involvement is a common feature in Systemic Sclerosis (SSc). Electrocardiographic (EKG) abnormalities are described in 25-75% of cases and are associated with other systemic manifestations such as pulmonary fibrosis (PF) and pulmonary arterial hypertension (PAH). The exact prevalence of cardiac rhythm diseases is difficult to determine due to the variety of symptoms and the lack of wide and well-defined cohorts. With this study we evaluated, in a monocentric cohort of SSc patients, all the existing EKG data, to identify the prevalence of the main abnormalities, as well as any possible association with other disease parameters, focusing on pulmonary involvement.

**Material and Methods:** We retrospectively investigated 276 SSc patients, fulfilling the 2013 ACR/EULAR classification criteria, attending our Scleroderma Clinic from 2004 to 2018. Patients were evaluated at baseline and for at least 2-year follow-up and records included demographic, clinical and laboratory assessments. Specific instrumental investigation such as EKG, 24 hours-EKG, transthoracic echocardiogram, HRTC and respiratory function test were included.

**Results:** 256 patients were females, with an average age (±SD) of 54 ±14 years; 65 subjects (23%) presented a diffuse form of the disease, 202 (73%) a limited one; 82 (29%) had anti-Topoisomerase I antibodies and 109 (39%) had anti-centromere antibodies. We found PF in 131 cases (47%), while PAH was present in 29 (10.5%) patients, confirmed by right heart catheterization. EKG abnormalities were found in 85 subjects (31%), and the most frequents and significant were: supraventricular ectopic heartbeat (25 cases/29,4%), atrial fibrillation (16 cases/18.8%), supraventricular paroxysmal tachycardia (9 cases/10.5%) and atrial flutter (3 cases/3,5%). Among the cardiac conduction disorders, 14 cases (16,5%) showed right branch-bundle-block, 12 (14%) left branch-bundle-block, 9 (10.5%) left anterior-hemiblock and 9 (10,5%) first-degree atrium-ventricular (AV) block.

In those patients with EKG abnormalities we found more FP cases (38 cases; 47%; p=0.02) than in those without, whereas PAH cases where significantly less frequent (14 cases; 16%; p<0.0001). The same result was confirmed analyzing separately those patients with rhythm disorders (p<0.0001 for PF; p=0.0009 for PAH), but not in those with conduction diseases

**Conclusions:** We detected EKG abnormalities in one third of our SSc cohort, a rather high percentage, that should be taken in account in the management of patients and for an appropriate follow-up. Besides their association with PF but not with PAH, deserve further investigation to better define the role for ILD as additional risk factor in the development of often life-threatening EKG findings.

## P.133

## ANTI-NEUTROPHIL CYTOPLASMIC ANTIBODIES IN SYSTEMIC SCLEROSIS, A CLUE FOR PRIMARY HEART INVOLVEMENT

G. Natalello^1^, G. De Luca^3^, G.B. Canestrari^2^, E. De Lorenzis^1^, C. Campochiaro^3^, L. Gigante^1^, L. Verardi^1^, B. Tolusso^2^, E. Gremese^1,2^, L. Dagna^3^, S.L. Bosello^2^

^1^*Institute of Rheumatology, Catholic University of the Sacred Heart, Rome, Italy*, ^2^*Department of Rheumatology, Fondazione Policlinico Universitario A. Gemelli - IRCCS, Rome, Italy*, ^3^*Department of Immunology, Rheumatology, Allergology and Rare Diseases (UNIRAR), San Raffaele Hospital - IRCCS, Milan, Italy*

**Introduction:** Primary heart involvement is common in Systemic Sclerosis (SSc) and portends a poor prognosis. The early detection and the risk stratification of SSc patients with cardiac complications are fundamental to promptly establish a treatment and to improve outcomes; biomarkers with prognostic meaning are eagerly needed. We evaluated the prevalence of Anti-Neutrophilic Cytoplasmic Antibodies (ANCA) in a two-centre Italian cohort of SSc patients and their possible clinical and prognostic significance, in particular in patients with heart involvement.

**Material and Methods:** Consecutive SSc patients were enrolled and prospectively followed. All the patients underwent ANCA assessment by anti-perinuclear/cytoplasmic staining (p/c-ANCA) and ELISA. All patients underwent a comprehensive disease assessment. Primary heart involvement was defined as the presence of signs/symptoms suggestive of cardiac disease and/or increase of high-sensitive cardiac enzymes and NT-proBNP values plus at least one of the following: LV-EF<55% on echocardiography; 24h-ECG Holter abnormalities; T1 edema, early and late gadolinium enhancement on cardiac magnetic resonance (CMR), in absence of coronary artery disease

**Results:** We evaluated 440 SSc patients with a mean age 60.9±14.7 years, mean disease duration of 15.1±8.8 years, 78.2% females and 34.2% with diffuse cutaneous disease (dcSSC). The mean follow-up time was of 43 months from the first time of ANCA assessment. ANCA positivity was detected in 37 patients (8.4%) and confirmed at least one time after 4 weeks; 15 of them (40.5%) presented a PR3+/cytoplasmic pattern and 22 (59.5%) an MPO+/perinuclear pattern. SSc patients with p/c-ANCA positivity were more likely to have anti-Scl70 antibodies positivity (59.5% vs 38.2%, p=0.01), dcSSc (54.1% vs 32.2%%, p=0.01) and interstitial lung involvement (73.0% vs 53.1%, p=0.02) . Fourteen out of 37 ANCA+ patients had primary heart involvement, compared to 72 out of 403 ANCA- patients (37.8% vs 17.9%, p=0.008). No association emerged between ANCA positivity and the presence of PAH, renal involvement, history of digital ulcers. Evaluating the possible predictive role of ANCA positivity for primary heart involvement in a model comprising anti-Scl70 antibodies positivity and presence of dcSSc, ANCA performed well showing 2.2-fold increase in relative risk (p=0.04). Survival rate estimated by Kaplan-Mayer curve was worse in patients with ANCA positivity for SSc-related deaths during the follow-up (χ²=6.13, p=0.01).

**Conclusions:** ANCA should be routinely tested in SSc, as they identify individuals with worse prognosis and primary cardiac involvement who require strict monitoring.

**Figure:**
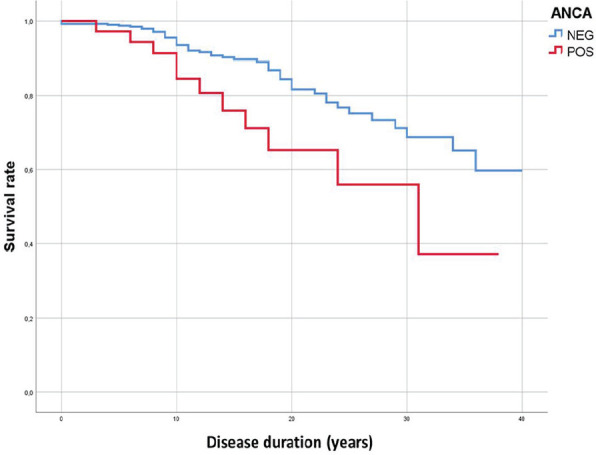


## P.134

## THE ROLE OF CARDIOPULMONARY EXERCISE TEST (CPET) FOR DETECTING PULMONARY ARTERIAL HYPERTENSION IN SYSTEMIC SCLEROSIS PATIENTS

I. Litinsky^1^, A. Man^2^, D. Paran^1^, S. Borok^1^, M. Berman^1^, U. Arad^1^, M. Anouk^1^, R. Zemach^1^, O. Elkayam^1^

^1^*Department of Rheumatology, Tel Aviv Sourasky Medical Center,Sackler Faculty of Medicine, Tel Aviv University, Tel Aviv, Israel*, ^2^*Institute of Pulmonary and Allergic Diseases, Tel Aviv Sourasky Medical Center, Tel Aviv, Israel*

**Introduction:** Pulmonary arterial hypertension (PAH) affects up to 15% of Systemic Sclerosis patients (SSc) and is associated with poor prognosis.

A definite diagnosis of PAH is obtained by right heart catheterization (RHCT), but the initial suspicion is raised by noninvasive methods, such as pulmonary function tests ( PFT) and ECHO Doppler, that have moderate sensitivity (71%) and specificity (69%).

CPET is a noninvasive test, measuring gas exchange during exercise and is considered a gold standard for cardiorespiratory functional assessment,showing characteristic changes in pulmonary vasculopathy. Although CPET application to pulmonary vasculopathy among SSc patients is relatively new.

**Material and Methods:** We performed a retrospective chart review of 157 SSc patients who were followed at our SSc clinic between January 2009- 2018.

Among this population, 23 patients (22 women, 1 men; 11(47.8%) with diffuse type, 12(52.2%) with limited type; mean age 61.73±14.67; disease duration 10.95±9.96 years), underwent CPET and were included in this analysis.

All these patients performed Echo Doppler and PFT prior to CPET.

The clinical laboratory and demographic data were also included in the analysis.

**Results:** The reasons for CPET referral were: Group 1. Increased Echo estimated pulmonary artery systolic pressure (mean PASP> 41.0±5.56) – 3 patients; Group 2: Decreased out of proportion predicted DLCO (mean 50.41±10.12) -10 patients; Group 3: Increased PASP (mean 35.95±9.96) and decreased predicted DLCO (mean 57.98±11.82) – 5 patients; Group 4: Unexplained dyspnea and palpitation without abnormalities on ECHO or PFT – 5 patients.

Of the whole group of 23 patients, CPET was found normal in 14 patients. All these patients didn’t undergo RHCT. All but 1 (lost of follow up) were annually followed up by Echo Doppler . Among these patients, no significant change in the PASP was observed during the mean 3.1 years follow up period.

CPET was abnormal in 9 patients. Increased VE/VCO2 (ventilator equivalent for CO2 at anaerobic threshold), was found abnormal in 8 patients (34.7%) . Percent of maximum predicted oxygen consumption (VO2 max) were decreased in 11 patients (47.8%). Both abnormalities were observed in 5 patients (21.7%).

Five patients with abnormal CPET (the mean Ve/VCO2 57.6±14.8), underwent RHCT. Three of them were diagnosed as PAH (mean PAP 44.6±4.5; PCWP 10.6±1.52). One had left heart failure with diastolic dysfunction and one patient shown normal PAP .

**Conclusions:** Normal CPET in SSc patients may be helpful to exclude the possibility of SSc-associated PAH, provides a potentially useful diagnostic information and may reduce unnecessary invasive RHC.

## P.135

## EFFICACY OF INTRAVENOUS IMMUNOGLOBULIN IN THE TREATMENT OF SYSTEMIC SCLEROSIS-RELATED MYOCARDITIS; A RETROSPECTIVE STUDY FROM IRANIAN SYSTEMIC SCLEROSIS REGISTRY

H. Kavousi, Z. Javadi Nejad, A. Javinani, A.R. Jamshidi, Z. Tamartash, F. Gharibdoost

*Rheumatology Research Center - Tehran University of Medical Sciences, Tehran, Iran*

**Introduction:** Systemic sclerosis (SSc) involved all three cardiac layers. Myocardial involvement is one of the disastrous manifestations of SSc which is poorly-recognized. Generally, atherosclerosis is the primary cause of heart failure among SSc patients. However, the myocardial fibrosis and inflammation are occurred by SSc per se. There is no approved treatment for these manifestations.

**Material and Methods:** In the present study, we have retrospectively reported the patients diagnosed with SSc-related myocarditis from Iranian SSc registry. All of the patients were treated by intravenous immunoglobulin (IVIg) with a dosage of 2 g/kg. The inclusion criteria were clinical signs of heart failure or sudden onset drops of ejection fraction (EF) in the presence of myocardial edema and fibrosis in cardiac MRI (CMR) study. Patients with concomitant ischemic heart disease, inflammatory myopathies, or overlap syndromes were excluded. The treatment efficacy assessed by trans-thoracic echocardiography. The Wilcoxon signed-rank test was used to compare pre- and post-treatment EF.

**Results:** Among 660 patients, nine (1.36%) were diagnosed with SSc-related myocarditis. All of them were female with a median age of 42.5 (37.7-56.7) years and the median disease duration of 8.0 (2.7-17.5) years. Two of them expired with a fulminant picture of heart failure and did not receive IVIg. The rest of them treated with IVIg for 3 to 6 monthly courses. Three of the patients (42.8%) had lung involvement with the median predicted forced vital capacity and Rodnan skin score of 77.0 (69.0-93.0) and 18.0 (15.0-26.5), respectively. The serological studies revealed that 5/5 had positive anti-Scl-70 antibody, 3/4 had a positive rheumatoid factor, and 1/4 had positive anti-double strand DNA antibody. At the baseline, fibrosis was detected in all of the CMR studies; however, three of them did not have the obvious sign of myocardial edema and inflammation. None of the individuals had elevated troponin I level. After the treatment by IVIg, all of the patients were recovered by improving the EF. The median baseline EF was 45.0% (35.0-50.0) that was changed to 55.0% (50.0-55.0) with a P-value of 0.026. The median recovery time was 12 (6-18) months following the IVIg administration. None of the patients experienced any significant side effect.

**Conclusions:** Myocarditis is a rare manifestation of SSc. It has a subtle clinical sign and symptoms in comparison to other types of myocarditis. The findings clearly indicate that IVIg could be a safe and effective choice of treatment for this rare condition.

## P.136

## NON-INVASIVE CARDIAC IMAGING TECHNIQUES FOR THE EVALUATION OF THE CARDIOVASCULAR INVOLVEMENT IN SYSTEMIC SCLEROSIS

A. Guillen-Del-Castillo, C. Espinet, M.N. Pizzi, A. Roque, V. Pineda, I. Sanz, E. Callejas-Moraga, P. Gubern, S. Aguade, V. Fonollosa, C.P. Simeón-Aznar

*Hospital Universitari Vall D’Hebron, Barcelona, Spain*

**Introduction:** To describe myocardial perfusion abnormalities and potentially associated coronary arteries lesions using non-invasive imaging techniques in a group of patients with SSc and suggestive symptoms of myocardial involvement (symptomatic) in comparison with a control group of patients with SSc without cardiac symptoms.

**Material and Methods:** A retrospective observational study was performed including a total of 61 patients diagnosed with SSc, 52 symptomatic, defined as dyspnea and/or chest pain (57.98 ± 12.3 years, 45 women) and 9 asymptomatic controls (50.2 ± 15.2 years, 8 women).

All patients underwent a post-stress (treadmill or pharmacological) myocardic perfusion gated-SPECT, a cold-induced stress SPECT, that were compared to a rest SPECT (to assess ischemia and / or necrosis), as well as an cardiac CT-angiography (to asses significant coronary arteries lesions, considering stenosis of more than 50%).

**Results:** Twenty-one out of the 52 symptomatic patients (50%) showed myocardial perfusion defects in the stress-rest SPECT: 13 (25%) showed ischemia, 13 (25%) fibrosis/necrosis, and 5 (9.6% ) ischemia and necrosis.

In the cold-induced SPECT images, 17 patients (32.7%) had myocardial perfusion abnormalities: 10 (19.2%) presented ischemia, 13 (25%) fibrosis/necrosis and 6 (11.5%) ischemia and necrosis.

In the other hand, of the 9 asymptomatic patients only a person (11%) had ischemia and necrosis in the stress-rest SPECT, being only positive for necrosis in the cold-induced SPECT images.

In the cardiac CT-angiography, 7/52 patients (13.4%) showed significant coronary lesions, 4 (57.2%) of them with perfusion defects in the SPECT images, and 3 (42.8%) without significant perfusion alterations.

Of the 9 asymptomatic patients, 1 (11%) had significant coronary lesions, being the same patient who presented perfusion defects in myocardial SPECT images.

**Conclusions:** Twenty-one out of the 52 symptomatic patients (50%) showed myocardial perfusion defects in the stress-rest SPECT: 13 (25%) showed ischemia, 13 (25%) fibrosis/necrosis, and 5 (9.6% ) ischemia and necrosis. .

In the cold-induced SPECT images, 17 patients (32.7%) had myocardial perfusion abnormalities: 10 (19.2%) presented ischemia, 13 (25%) fibrosis/necrosis and 6 (11.5%) ischemia and necrosis.

In the other hand, of the 9 asymptomatic patients only a person (11%) had ischemia and necrosis in the stress-rest SPECT, being only positive for necrosis in the cold-induced SPECT images.

## P.137

## MYOCARDIAL FIBROSIS IN SYSTEMIC SCLEROSIS ASSESSED BY CARDIAC MAGNETIC RESONANCE IS ASSOCIATED WITH VASCULAR ENDOTHELIAL GROWTH FACTOR EXPRESSION

G. Leodori^1^, C. Pellicano^1^, A.L. Villa^1^, A. Iacolare^1^, A. Gigante^1^, N. Galea^2^, M.L. Gasperini^1^, I. Carbone^2^, E. Rosato^1^

^1^*Department of Translational and Precision Medicine, Sapienza University of Rome, Rome, Italy*, ^2^*Department of Radiological, Oncological and Pathological Sciences, Sapienza University of Rome, Rome, Italy*

**Introduction:** Systemic sclerosis (SSc) is characterized by endothelial dysfunction and fibrosis of the skin and the internal organs, including the heart. Fibrosis and its complications are main cardiac involvement related to SSc.

Vascular damage and chronic tissue hypoxia promote angiogenesis by production of pro-angiogenic factors such as vascular endothelial growth factor (VEGF) in SSc.

Cardiac magnetic resonance (CMR) is employed to detect myocardial fibrosis showing patchy fibrosis. In this pilot study we aimed to evaluate myocardial fibrosis in SSc using CMR with late gadolinium enhancement (LGE) and VEGF expression.

**Material and Methods:** Twenty-eight SSc patients [20 women, aged 40 (36-48) years] were enrolled. Serum VEGF levels were determined in SSc patients. CMR imaging was performed with a 1.5-T unit .

**Results:** Serum median value of VEGF was 187 pg/ml (128-251). In 17 (60,7 %) SSc patients focal areas of LGE were found predominantly with patchy mesocardial distribution. Focal myocardial oedema was observed in 4 (14,3%) SSc patients. The median value of VEGF was higher (p<0,01) in SSc patients with LGE than in SSc patients without LGE [233 (183-270) vs 100 (67-191)].

**Conclusions:** VEGF increases in SSc patients with myocardial fibrosis. We can suppose that in primary cardiac involvement related to SSc, microvascular damage stimulates impaired angiogenesis. The defect in the vascular repair and new blood vessels formation promotes myocardial fibrosis.

## P.138

## ASSOCIATED FACTORS OF EARLY-ONSET PULMONARY HYPERTENSION AND CLINICAL DIFFERENCE BETWEEN EARLY- AND LATE-ONSET PULMONARY HYPERTENSION IN SYSTEMIC SCLEROSIS

C. Foocharoen, T. Krikeerati, B. Pussadhamma, A. Mahakkanukrauh, S. Suwannaroj, R. Nanagara

*Khon Kaen University, Khon Kaen, Thailand*

**Introduction:** Pulmonary hypertension (PH) is a major cause of death in systemic sclerosis (SSc). Early onset PH (early-PH) detection and finding out its associated factors would be helpful for improving the patients care in daily practice. Our aims were to determine the factors associated with early-onset pulmonary hypertension and to define the differences between early- and late-onset pulmonary hypertension in SSc patients

**Material and Methods:** A historical cohort study was conducted of adult SSc patients who had followed-up at Srinagarind Hospital between January 2014 -December 2016. PH was defined by mean pulmonary arterial pressure by right heart catheterization >20 mmHg. Early-PH is defined when the onset of PH was diagnosed within 5 years of the disease. Logistic regression analysis was applied for finding out the factors associated with early-PH.

**Results:** A total of 409 SSc patients were enrolled. By 3,409 person-years, 50 cases were diagnosed PH, of which 26 cases were defined as early-PH with the incidence of 0.7 per-100 person-years (95%CI 0.5-1.1). Of whom diagnosed early-PH, female to male ratio was 2.7:1 and 69.2% were diffuse cutaneous SSc (dcSSc). The respective mean age and mean duration of disease at time of diagnosis early-PH diagnosis was 57.1±14.2 years and 2.2±1.4 years. Most common PH-classification in the patients who had early-PH was PH due to interstitial lung disease (PH-ILD) 18 cases (69.2%), followed by pulmonary arterial hypertension (PAH) 5 cases (19.2%), combined PAH and PH-ILD 2 cases (7.7%) and pulmonary veno-occlusive disease 1 case (3.9%). was By logistic regression analysis, WHO functional class (WHO-FC) II, cardiomegaly by chest radiography, tricuspid regurgitation velocity (TRV) >2.8 m/sec was associated with early-PH with OR 20.12 (95%CI 1.59-255.35), 7.42 (95%CI 1.35-40.88) and 8.20 (95%CI 1.17-57.64), respectively, while stomach symptoms had a negative association with early-PH (OR 0.08; 95%CI 0.01-0.56).

**Conclusions:** Early-PH is revealed in SSc patients and majority cause is ILD. Poor WHO-FC, cardiomegaly and high TRV are associated with early-PH among SSc patients. Stomach involvement is a protective factor for early-PH in SSc.

## P.139

## CLINICAL OUTCOMES OF MYOCARDITIS AFTER STEROID THERAPY IN SYSTEMIC SCLEROSIS: A PILOT STUDY

C. Foocharoen, B. Pussadhamma, N. Chaosuwannakit, T. Tipparot, A. Mahakkanukrauh, S. Suwannaroj, R. Nanagara

*Khon Kaen University, Khon Kaen, Thailand*

**Introduction:** Myocarditis is reported in systemic sclerosis (SSc); however, treatment options and respective outcomes are limited. The aims was to define the cardiac outcomes after moderate dose steroid therapy in SSc patients with myocarditis

**Material and Methods:** An open-label study was conducted among Thai adult SSc patients with myocarditis defined by cardiovascular magnetic resonance (CMR) per the Lake Louise Criteria, disease onset <5 years, and a NYHA functional class (FC) more than FC I . We excluded patients taking steroids and/or immunosuppressants or having a prior known heart disease. All enrolled patients received prednisolone (30 mg/d) which would be tapered off by week 24 and CMR was followed up at the end of treatment.

**Results:** A total of 20 SSc patients were enrolled. The female to male ratio was 1.9:1. The majority (75%) had diffuse cutaneous SSc (dcSSc). The respective mean age and mean duration of disease was 52.8±15.3 and 3.2±1.3 years. Twelve patients completed the study. At week 24 after treatment, 8 of the 12 cases (66.7%) experienced improvement of the myocarditis. Compared to those with no improvement, these 8 patients had significantly longer disease duration (p=0.03); higher heart rate at baseline (p=0.049) and week 24 (p=0.04); lower LV and RV stroke volume at baseline (p=0.002 and p=0.01) and week 24 (p=0.01 & p=0.02); and lower LV and RV cardiac output at week 24 (p=0.01 and p=0.01). Four cases died during follow-up (3 due to cardiac complications, 1 due to renal crisis); all four had dcSSc, high hs-cTnT, and high NT-proBNP. Two who died from heart failure had impaired LV systolic function and 1 who died from arrhythmia had very high hs-cTnT.

**Conclusions:** Moderate dose steroid therapy may improve myocarditis in SSc. A proportion of patients had cardiac complications and died during treatment; particularly those with dcSSc who had high hs-cTnT, high NT-proBNP, and impaired LV systolic function. Long-term outcomes should be further investigated.

Clinical trial number: NCT03607071

## P.140

## SYSTEMIC SCLEROSIS IS ASSOCIATED WITH IMPAIRED CORONARY FLOW RESERVE ON 82-RUBIDIUM POSITRON EMISSION TOMOGRAPHY/ COMPUTED TOMOGRAPHY

A. Feher, N. Boutagy, E. Oikonomou, S. Thorn, Y.-H. Liu, E. Miller, A. Sinusas, M. Hinchcliff

*Yale University School of Medicine, New Haven, USA*

**Introduction:** Cardiovascular (CV) mortality is the leading cause of death in autoimmune conditions. Coronary microvascular dysfunction has been frequently described in patients with secondary Raynaud phenomenon (RP). Positron emission tomography (PET) is considered to be the noninvasive gold standard for the evaluation of coronary microvascular function. However, the utility of PET-derived coronary flow reserve (CFR) to identify microvascular disease in patients with primary and secondary RP is unknown.

**Material and Methods:** Patients with a diagnosis of RP in the electronic health record who underwent dynamic rest-stress 82-Rubidium PET/CT from 09/2012-09/2019 for evaluation of chest pain or dyspnea were retrospectively studied. Rate-pressure product corrected CFR was calculated (Corridor 4DM, INVIA). Patients were grouped based on their comorbid autoimmune conditions and were compared to healthy controls. One-way-ANOVA was used to assess between-group differences in CFR with Dunnett’s test used for multiple comparisons, whereas clinical predictors of reduced CFR (< 2.0) were evaluated using multivariable logistic regression (SPSS, IBM).

**Results:** 49 patients with RP (84% female, age: 65 ± 11 years, BMI: 33 ± 11 kg/m2) were included in this study. Of these, 11 had primary RP, 18 had systemic sclerosis (SSc) and 20 had other autoimmune diagnosis (n=12 rheumatoid arthritis, n=7 systemic lupus erythematosus, n=3 Sjogren’s syndrome). CFR was significantly different between the groups (p=0.0045, Fig. 1A). Patients with primary RP had CFR comparable to healthy controls (n=17, 35% female, age: 35 ± 5 years, BMI 27 ± 4 kg/m2). In patients with secondary RP, only those with underlying SSc had significantly reduced CFR compared to controls. In multivariable logistic regression, SSc and chest pain were independent predictors of reduced CFR (Fig. 1B). In addition, there was a weak, but significant, correlation between time since autoimmune disease diagnosis and CFR (r= -0.37; 95% CI: -0.61 to -0.09; p=0.01). The level of inflammatory markers (sedimentation rate: r= -0.19, c-reactive protein: r= -0.31) did not correlate with CFR (p>0.05).

**Conclusions:** In this preliminary study, our findings suggest that patients with primary RP had CFR values that were comparable to healthy controls. In patients with secondary RP, only SSc was associated with reduced PET CFR compared to healthy controls. Moreover, SSc may be an independent predictor of reduced CFR. Larger prospective studies are warranted to fully elucidate the prognostic value of CFR in patients with RP.

**Figure:**
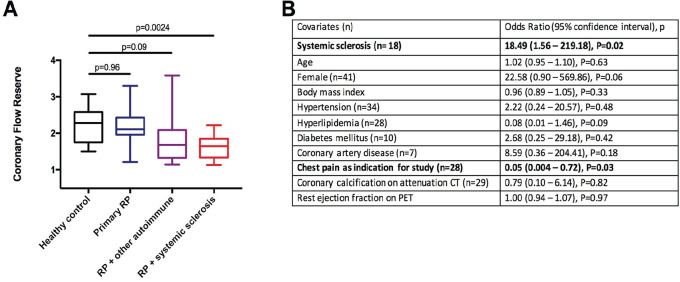


## P.141

## DECLINE IN SUBCLINICAL SYSTEMIC SCLEROSIS PRIMARY HEART INVOLVEMENT ASSOCIATES WITH POOR PROGNOSTIC FACTORS AND ACTIVE INTERSTITIAL LUNG DISEASE

R.- B. Dumitru^1^, L.A. Bissell^1^, B. Erhayiem^2^, G. Fent^2^, A. Kidambi^2^, G. Abignano^1^, J.P. Greenwood^2^, J. Biglands^3^, F. Del Galdo^1^, S. Plein^2^, M.H. Buch^1,4^

^1^*Leeds Institute of Rheumatic and Musculoskeletal Medicine, University of Leeds, Leeds, United Kingdom*, ^2^*Department of Biomedical Imaging Science, Leeds Institute of Cardiovascular and Metabolic Medicine, University of Leeds, Leeds, United Kingdom*, ^3^*National Institute for Health Research, Leeds Biomedical Research Centre, Leeds, United Kingdom*, ^4^*Division of Musculoskeletal & Dermatological Sciences, University of Manchester & NIHR Manchester Biomedical Research C, Manchester, United Kingdom*

**Introduction:** Primary systemic sclerosis heart involvement (pSSc-HI) is described in the majority of SSc patients when sensitive methods such as cardiovascular magnetic resonance (CMR) are used. The natural history of these subclinical findings are unknown. This study aimed to evaluate for interval change in subclinical pSSc-HI and whether disease modifying antirheumatic (DMARD) or vasodilator treatment influence the CMR course.

**Material and Methods:** SSc patients, fulfilling the 2013 ACR/EULAR criteria, with no cardiovascular (CV) disease, diabetes and no more than 2 CV risk factors had two CMRs performed (V1 & V2; minimum 1 year apart). A 3T CMR with late gadolinium enhancement (LGE), T1 mapping for extracellular volume (ECV) quantification and stress perfusion was undertaken.

**Results:** 31 SSc patients were included, with median (IQR) follow up (between the 2 CMR scans) of 33 (17, 37) months. Their median (IQR) age was 52 (47,60), 32% had diffuse cutaneous SSc, 52% interstitial lung disease (ILD), 29% Scl70+.

4/31 patients had a non-ischaemic LGE pattern suggesting focal fibrosis at V1, with no change in the pattern, distribution, or median (IQR) LGE scar mass between V1 and V2 [1.88 (1.01, 6.34) vs 1.70 (1.21, 4.18)]. At V2, 2 additional patients showed focal fibrosis, of which one had an episode of clinically diagnosed myocarditis. No significant change in ECV, T1 native, myocardial perfusion reserve (MPR) or left ventricle (LV) volumes and function were noted at V2 compared with V1 (p>0.01). SSc patients with either increase in pre-existing LGE scar mass (n=1) or new fibrosis were all dcSSc, with ILD, 2 Scl70+. A reduction in forced vital capacity and total lung capacity associated with a reduction in LV ejection fraction (LVEF) (rho=0.413, p=0.021; rho-0.335, p=0.07) and MPR (rho=0.543, p=0.007; rho=0.627, p=0.002).

Patients receiving DMARD treatment showed greater decrease in LVEF [median (IQR) 0.97(-2.76, 1.51) vs 2.47 (0.06, 7.98), p=0.020] and an increase in T1 native [median (IQR) 60 (-6,110) vs -4 (-50, 49), p=0.049] (Figure 1). No significant change in CMR measures in those receiving vasodilator or angiotensin-converting-enzyme inhibitor treatment was noted (p>0.01).

**Conclusions:** This first, pilot longitudinal study of CMR-defined subclinical pSSc-HI demonstrates interval changes with progression of/new focal fibrosis and decline in LV function and MPR associating with poor prognostic factors of SSc and ILD progression. Consistent with this, individuals on DMARD also appeared to show interval decline. Larger longitudinal studies are warranted to confirm these findings and inform application of CMR in monitoring subclinical pSSc-HI.

**Figure:**
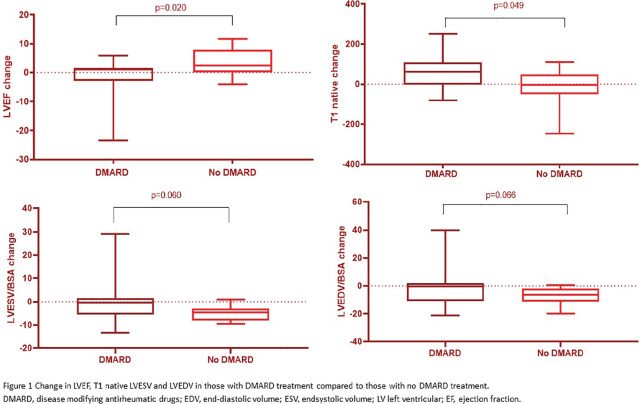


## P.142

## PROGRESSION OF SUBCLINICAL MYOCARDIAL INVOLVEMENT IN PATIENTS WITH SYSTEMIC SCLEROSIS

D. Benfaremo^1^, L. Manfredi^1^, L. Zuliani^1^, G. Stronati^2^, F. Guerra^2^, A. Ferrarini^1^, C. Fischetti^1^, M.G. Danieli^1^, A. Capucci^2^, A. Gabrielli^1^

^1^*Clinica Medica, Dipartimento di Scienze Cliniche e Molecolari, Università Politecnica delle Marche, Ancona, Italy*, ^2^*Clinica di Cardiologia e Aritmologia, Università Politecnica delle Marche, Ancona, Italy*

**Introduction:** Systemic sclerosis (SSc) is a progressive autoimmune disease affecting the skin as well as internal organs, including the heart. A few studies have identified a subclinical heart involvement in patients with no pulmonary hypertension. Changes in myocardial deformation are consistent with the idea of SSc-related cardiomyopathy as a primary condition affecting the heart globally through microvascular dysfunction and subsequent myocardial fibrosis. The aim of the present study is to describe the progression of myocardial deformation in patients with SSc and no overt cardiac disease.

**Material and Methods:** Prospective longitudinal study enrolling consecutive SSc patients referred to the Clinica Medica, University Hospital ‘Ospedali Riuniti’, Ancona, Italy, from February 2016 to December 2018. All patients fulfilled the 2013 ACR/EULAR classification criteria for SSc. Patients with structural heart disease, heart failure, atrial fibrillation or pulmonary hypertension were excluded. Disease subset, antibodies pattern, cardiovascular risk factors and involvement of other organ systems were recorded for each patient. An echocardiographic exam was performed for all patients at baseline and during their follow-up evaluation. Standard and speckle-tracking derived variables for the systolic and diastolic function of the left ventricle (LV) and right ventricle (RV) were acquired. Speckle tracking analysis software (EchoPAC 13.0; GE Medical Systems, Milwaukee, USA) was used to assess the GLS of the left and right ventricle, excluding the ventricular septum from right ventricular GLS calculations.

**Results:** Seventy-two patients (68 females, age 56.6±15.4 years) were enrolled. Common echocardiographic parameters of left and right systolic function were within normal range at baseline and did not change during follow-up. Mean GLS, however, worsened for both left (from -19.8±3.5% to -18.7±3.5%, p=.034) and right ventricle (from -20.9±6.1% to -18.7±5.4%, p=.013) during a median follow-up of 20 months (1st-3rd quartile 12-24 months). The increased impairment registered in SSc patients was homogenous across endocardial layers (LV from -22.5±-3.9 to -21.4±3.9, p=.041; RV from -24.2±6.2 to -20.6±5.9, p=.001), mesocardial layers (LV -19.7±3.6 to -18.7±3.5, p=.043; RV from -21.3±5.9 to -18.8±5.7, p=.012) and epicardial layers (LV from -17.1±3.0 to -16.4±3.1, p=.112, RV -18.8±6.3 to -16.0±8.4, p=.035), as well as myocardial segments. No difference in progression rate was observed stratifying patients according to disease subset or other clinical parameters.

**Conclusions:** GLS impairment progressed over a 20-month follow-up period in a cohort of SSc patients without clinically overt cardiac involvement. Further studies are needed to assess the significance of subclinical heart involvement and its progression in patients with SSc.

## P.143

## ROLE OF RIGHT VENTRICULAR STRAIN AND 2D-SPECKLE TRACKING ECHOCARDIOGRAPHY IN A COHORT OF PATIENTS WITH SYSTEMIC SCLEROSIS

A. Spinella^1^, P. Macripo’^2^, E. Cocchiara^1^, E. Galli^1^, F. Lumetti^1^, L. Magnani^3^, F. Coppi^2^, A.V. Mattioli^4^, R. Rossi^2^, G. Boriani^2^, C. Salvarani^1^, D. Giuggioli^1^

^1^*Scleroderma Unit, Chair of Rheumatology, University of Modena and Reggio Emilia, Modena, Italy, Modena, Italy*, ^2^*Chair of Cardiology, University of Modena and Reggio Emilia, Modena, Italy, Modena, Italy*, ^3^*Unit of Rheumatology, AUSL-IRCCS of Reggio Emilia, Italy, Reggio Emilia, Italy*, ^4^*Department of Surgical, Medical and Dental Department of Morphological Sciences, University of Modena and Reggio Emilia, Modena, Italy*

**Introduction:** Systemic Sclerosis-SSc is a connective tissue disease, characterized by endothelial dysfunction and fibrosis, potentially affecting internal organs and reducing life expectancy. Cardio-pulmonary involvement is common: pulmonary fibrosis, pulmonary hypertension-PH, and electrical disorders are the most serious complications with decreased life expectancy.

We evaluated SSc features related with the onset and development of PH. We studied ecocardiographic abnormalities in detail, by means of 2D-speckle tracking echocardiography (STE) with specific reference to the right ventricular strain measure (RV-strain).

**Material and Methods:** We retrospectively analyzed data from 50 SSc patients (pts) referred to our University-based Rheumatology Centre and SSc Unit from January 2007 to June 2019 (F/M 45/5; lc/dcSSc 45/5; mean age 59.20±14.357 years; mean disease duration 12.08±8.75 years). All pts underwent general and cardio-pulmonary assessment in our Cardio-Rheumatology Clinic, included RV-strain measured by 2D-STE. The following parameters were also considered: blood tests, in particular inflammation indexes, uric acid test and serum autoantibodies; pulmonary function tests; high resolution scan of the lungs (HRCT) and standard electrocardiogram (ECG). The clinicians decided to perform these examinations according to clinical picture and current methodologies. We compared SSc pts with (10/50) and without (40/50) PH diagnosis during follow-up period regardless of treatments.

**Results:** SSc pts with PH didn’t show significant results concerning RV-strain if compared with pts without PH (p=0.707). Nevertheless, RV-strain value was modified in relation to TAPSE alterations in all pts but this data correlated with right ventricular dilatation only in PH subjects. Furthermore, interesting values were observed with respect to dilatation of right and left atria (p=0.007, p=0.048)) as well as dilatation of inferior vena cava (p=0.037) and right ventricle (p=0.023). Left ventricular hypertrophy (p=0.012) and valvular insufficiencies (mitral and aortic) were more frequent in PH group too (p=0.016). These subjects showed higher incidence of skin ulcers (p=0.0001), higher values of uric acid (p=0,027) and anti-centromere antibodies positivity (p=0.0001). An interesting correlation between PH and blood pressure was also documented (p=0.04).

**Conclusions:** Our findings provide further evidence of the prognostic value of echocardiographic alterations in SSc subjects with PH. Population enlargement is ongoing in order to identify more accurate results about RV-strain, considering the efficacy of PH treatments on cardiac contractility. Speckle tracking echocardiography appears to be a sensitive, low-cost, non-invasive and reliable tool that aims to detect early cardiac involvement in SSc, with interesting future prospects.

## P.144

## SUCCESSFUL TAVI PROCEDURE FOR REPAIR OF AORTIC STENOSIS IN SSC PATIENTS

A. Balbir Gurman^1^, A. Kerner^2^, Y. Agmon^2^, Y. Braun-Moscovici^1^

^1^*Rheumatology Institute, Rambam Health Care Campus, Rappaport Faculty of Medicine-Technion, Haifa, Israel*, ^2^*Cardiology Institute, Rambam Health Care Campus, Rappaport Faculty of Medicine-Technion, Haifa, Israel*

**Introduction:** Patients with systemic sclerosis (SSc) are at risk for developing aortic valve changes. The safety of trans catheter aortic valve implantation (TAVI) in SSc need to be assessed.

**Material and Methods:** A retrospective study on aortic valve pathology and treatments including TAVI performance.

**Results:** We reviewed 373 records of SSc patients at our site EUSTAR cohort. Clinical significant aortic stenosis (AS) was confirmed by ECHO cardiography in 13 (3.4%) patients (12 females 92.3%; mean age 70.3 (SD 7.7) years, disease duration 15.4 (SD 6.3) years, limited disease 10 (76.9%, all cared centromere antibodies) and 2 diffuse scleroderma (1 RNAP3 and 2 anti-topoisomerase antibodies); 5 (38.5%) patients had significant coronary disease (3 underwent CABG, 2 had several PTCA). Eight patients dead (61.5%) during years 2004 - 2019. AVR (1 biological) was performed in 5 patients; 2 patients did not undergo AS repair; 6 patients underwent TAVI between January 2013 and September 2019 (5 at Rambam Cardiology Institute). All SSc patients underwent trans femoral TAVI under conscious sedation. The procedure was successfully performed in all patients. The length of hospitalization was 14-5 (mean (8.2 days); 3 patients needed pacemaker implantation. The follow-up duration after TAVI was between 5 and 63 months (mean 20.7): one patient had bacterial endocarditis resolved with prolonged antibiotics treatment without sequela; the same patient had TIA two years after TAVI; one patients dead from urosepsis 11 months after TAVI (not related to procedure); one patient had anemia and need blood transfusion close to the procedure.

**Conclusions:** AS is not rare in SSc patients especially in those with long lasting limited disease and positivity to centromere antibodies. AS in SSc patients may be associated with clinically significant coronary atherosclerosis. TAVI was safe in our SSc patients without in-hospital mortality and benign prognosis.

## 5. Outcomes, Quality of Life, Psychological & Social

## P.145

## THE CLINICAL CHARACTERISTICS OF SYSTEMIC SCLEROSIS WITH LUNG CANCER: DATA FROM SINGLE CENTER IN CHINA

H. Zhong^1.3^, J. Zhou^1,3^, S. Zhang^1,3^, Y. Xu^2,3^, Y. Hou^1,3^, M. Li^1,3^, D. Xu^1,3^, M. Wang^2,3^, X. Zeng^1,3^

^1^*Peking Union Medical College Hospital - Department of Rheumatology, Beijing, China*, ^2^*Peking Union Medical College Hospital - Department of Respiration, Beijing, China*, ^3^*Chinese Academy of Medical Sciences & Peking Union Medical College, Beijing, China*

**Introduction:** Systemic sclerosis (SSc) with malignancies is not uncommon, especially lung cancers, and more attention should be paid from clinicians. We hope to increase the knowledge of this condition in clinicians through analyzing the clinical features of patients of SSc with lung cancer in China.

**Material and Methods:** Medical records of inpatients admitted in Peking Union Medical College Hospital from March 1992 to December 2018, were retrospectively collected and analyzed. SSc patients without lung cancer during the same period, matched by age and gender, were selected as the controls.

**Results:** Nineteen cases were identified, with 17 (89.5%) females. The mean onset age of SSc is (37.8±12.0) years old and the diagnosis age of lung cancer is (54.4±10.2) years old . One (5.3%) male had smoking history. Eight (42.1%) patients had family history of cancer, which is significantly higher than those without lung cancer (4 patients, 5.3%, P=0.000). Limited cutaneous SSc were 63.2%, and 18 (94.7%) cases have interstitial lung diseases (ILD), and the difference between the two groups is not statistically significant. ILD were all diagnosed before the onset of lung cancer, with a median interval of 9.2 (range 1.6-28.1) years. SSc patients with lung cancer have more pulmonary hypertension (75.0% vs. 25.0%, P=0.022) and more esophagus involvement (84.2% vs. 57.4%, P=0.029), and less myositis (0% vs. 27.6%, P=0.032) than control group. All patients developed lung cancer after the diagnosis of SSc, with a median interval of 10.5 (range 2.0-36.2) years. Lung cancers in 18 cases (94.7%) happened after at least 6 years of SSc onset. Newly happened cough (9 cases), new decrease in activity endurance (3 cases), chest pain (2 cases), hemoptysis (2 cases), nodes in lung through regular CT scans (3 cases) were the first presented in lung cancer. Seven (36.8%) patients were at or prior to stage IIIA and had significantly longer survival than those with stage IIIB or IV(29.1±15.1 vs. 6.2±4.5 months, P=0.006). Eight patients tested EGFR gene mutation or ALK gene rearrangement, and only 2 are positive.

**Conclusions:** SSc with lung cancer is not uncommon, especially for those with long disease duration and family history of malignancy. Due to the subtle onset of lung cancer, clinicians should pay attention to it during clinical practice.

## P.146

## DEVELOPMENT OF A PATIENT-CENTRED ILOPROST AND ALPROSTADIL INFUSION INFORMATION SHEET FOR PATIENTS WITH SYSTEMIC SCLEROSIS

M.-C. Yelovich^1^, K. Beattie^1^, A. Gardner^2^, C. Whisken^3^, N. Khalidi^1,2^, M. Larche^1,2^

^1^*McMaster University, Hamilton, Canada*, ^2^*St. Joseph’s Healthcare Hamilton, Hamilton, Canada*, ^3^*Charlton Health, Hamilton, Canada*

**Introduction:** Intravenous prostanoids such as iloprost have been demonstrated effectiveness in healing digital ulcers and potentially preventing new digital ulcers in patients with systemic sclerosis (SSc). The most recent EULAR recommendations included iloprost as a treatment for severe Raynaud’s Phenomenon and digital ulcers, particularly when oral therapies have failed. In Canada, available prostanoid infusions used include iloprost and alprostadil. An important issue raised by patients who have received these infusions has been the limited patient-directed information available to them. A quality improvement initiative was undertaken to address this care gap by developing a patient-centred information sheet about prostanoid infusions.

**Material and Methods:** To assess the need for this information sheet and the contents it should include, a questionnaire was circulated to patients receiving prostanoid infusions between November 2017 and February 2019. Questions posed related to their level of knowledge and preparation for the infusion, where prior information had been obtained, and any further information about the infusion that they wished they had received. Of 17 respondents, 6 (35.3%) mentioned specific information about side effects, pain management, and what to expect after the infusion that they would have liked to have received.

Based on this feedback, a preliminary draft of a patient information sheet was assessed and altered to address common patient questions about the infusion experience, including common and uncommon side effects to monitor for, symptoms that could be addressed by a nurse during the infusion with medication to treat pain or nausea, and what symptoms after the infusion should prompt a call to their doctor. This information sheet then underwent 2 Plan-Do-Study-Act (PDSA) cycles between February 2019 and September 2019. During this time, feedback was elicited from patients receiving prostanoid infusions. Significant revisions were incorporated into the next iteration. The information sheet was also reviewed by two rheumatologists with expertise in SSc and a pharmacist with expertise in rheumatological medications.

**Results:** Approximately one third of patients receiving prostanoid infusions reported lacking information even after their initial discussion with their health care provider. Specific concerns elicited from initial questionnaires were incorporated into a patient information sheet. The information sheet has undergone 2 PDSA cycles to date, with 3 patients participating per cycle, and after each cycle the sheet was revised based upon patient feedback and feedback from other reviewers, including a rheumatology pharmacist.

**Conclusions:** Patient input and feedback was instrumental in the development of a prostanoid infusion patient-centred information sheet.

## P.147

## THE RELATIONSHIP BETWEEN DISEASE ACTIVITY AND SEVERITY IN SYSTEMIC SCLEROSIS: A PROSPECTIVE ANALYSIS OF 278 PATIENTS

Y. Yalcinkaya, N. Aliyeva, S. Amikishiyev, Y. Çagatay, B. Artim Esen, A. Gul, L. Ocal, M. Inanc

*Istanbul University, Istanbul Medical Faculty, Departement of Internal Medicine, Division of Rheumatology, Istanbul, Turkey*

**Introduction:** We aimed to investigate the relationship between disease activity and severity in a large systemic sclerosis (SSc) cohort.

**Material and Methods:** This is a cross-sectional prospective analysis of 278 (253 females) patients fulfilling ACR/EULAR (2013) classification criteria for SSc. Disease activity and severity were calculated by using validated indexes (EscSG and Medsger). The patients were grouped as inactive if EscSG score=0, mildly active if EscSG score>0<3, active if EscSG score>3. Disease activity and severity of were calculated seperately for disease subgroups according to cutaneous involvement

**Results:** The mean age, duration of Raynaud’s and non-Raynaud features were 48.5±13.1, 12.1±9.8 and 8.3±7.5 years respectively. Characteristics of the SSc patients were summarized in table-1.

Ninety-three (34%) and 151(54%) patients were evaluated as having active and mildly active disease. Only 34(12%) patients had inactive disease. The patients with diffuse cutaneous involvement (dcSSc) who were active had higher modified Rodnan Skin score(mRSS) and severity scores of general, skin and joint-tendon involvements; the patients who had mildly active disease also had higher scores of mRSS and severity scores of skin compared to those with inactive disease (table-2).

The patients with limited cutaneous involvement (lcSSc) who were assessed as having active disease had higher mRSS and higher severity scores of general, skin, peripheral vascular, lung, joint-tendon and gastrointestinal involvements; the patients who had mildly active disease also had higher scores of mRSS and severity scores of skin, lung, joint-tendon and gastrointestinal involvements compared to those with inactive disease (table-2).

**Conclusions:** One third of our cohort was found to have active disease despite treatment and only 12% had inactive disease. Skin involvement and severity of different organs were shown to be higher in patients with active disease in both cutaneous subsets, together with severity of lung, peripheral vascular and gastrointestinal involvements in active lcSSc. LcSSc and dcSSc patients who had mildly active disease also had severe disease similar to those with active patients. Disease activity and severity should be assessed as separate measurements to highlight the course of the disease and may guide to the management of patients with SSc.

**Figure:**
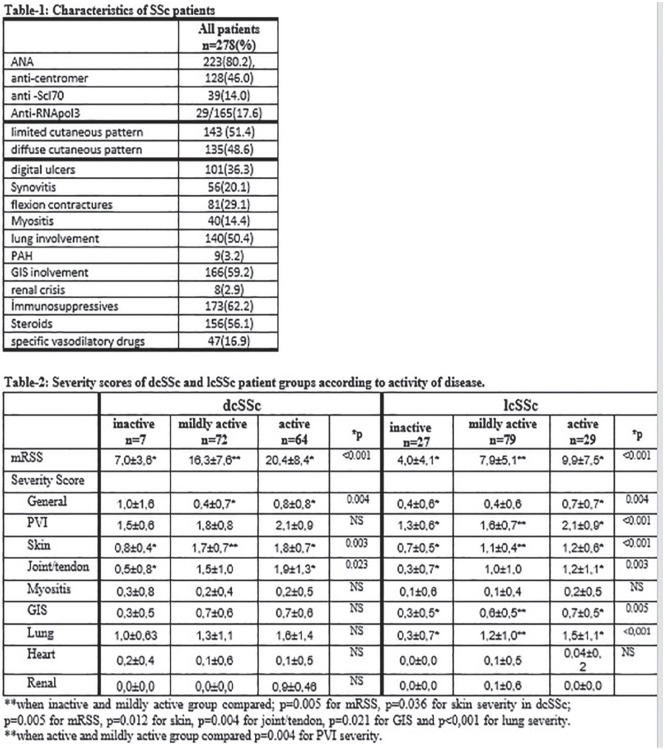


**Figure:**
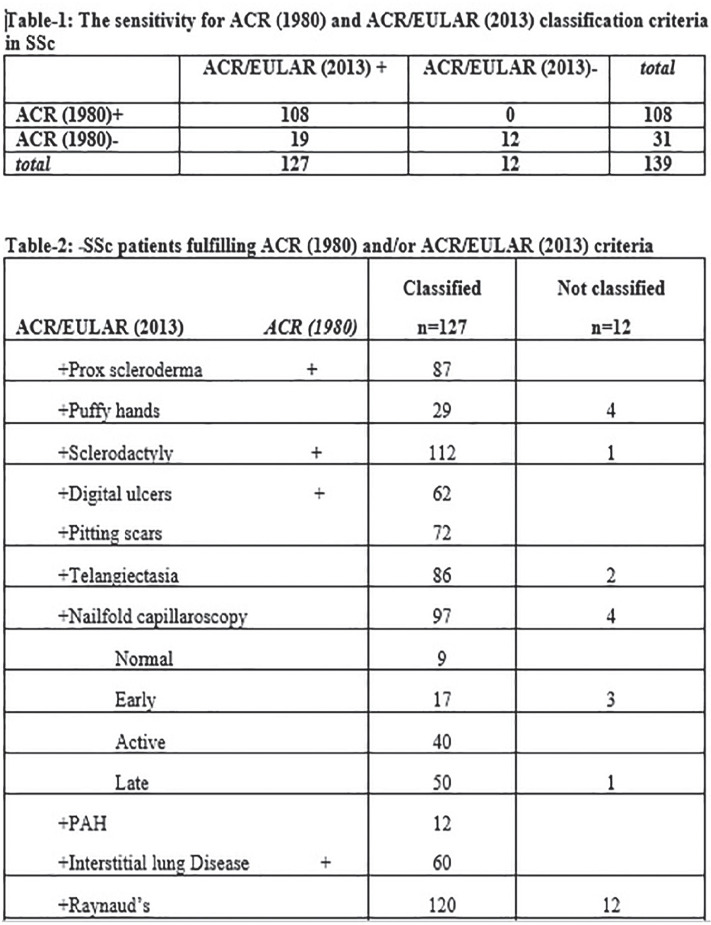


## P.148

## EVALUATION OF DIFFERENT CLASSIFICATION CRITERIA IN SYSTEMIC SCLEROSIS IN A TURKISH COHORT: THE IMPORTANCE OF NON-SKIN MANIFESTATIONS, SEROLOGY AND CAPILLAROSCOPY

Y. Yalcinkaya, S. Amikishiyev, N. Aliyeva, B. Artim Esen, A. Gul, L. Ocal, M. Inanc

*Istanbul University, Istanbul Medical Faculty, Departement of Internal Medicine, Division of Rheumatology, Istanbul, Turkey*

**Introduction:** Proximal scleroderma is the major criterion in both 1980 and 2013 classification criteria for sytemic scleroris (SSc). ACR(1980) criteria included digital lesions and bibasiler fibrozis, nonetheless ACR/EULAR(2013) criteria based on a scoring system including digital lesions, telangiectasia, abnormal nailfold video-capillaroscopy (NVC), PAH, Raynaud’s and specific autoantibodies. We aimed to implement both criteria in a Turkish SSc kohort to evaluate the contribution of non-skin manifestations, NVC and autoantibodies.

**Material and Methods:** A consecutive hundred and thirty-nine (125 females) SSc patients diagnosed and evaluated by the same experts (YY, MI) with relevant NVC records and at least 6 months follow-up were included into the study. Classificaiton criteria were used retrospectively using a preformed database.

**Results:** Characteristics of the SSc patients were summarized in table-1. The mean age, duration of Raynaud’s and non-Raynaud symptoms were 47.1±11.9, 8.9±7.9 and 5.7±5.8 years, respectively. Diffuse and limited cutaneous disease were diagnosed in 62(44.6%) and 60(43.2%) patients respectively. Asclerodermic disease was present in 17(12.2%) patients. ANA, anti-centromere and anti-Scl70(+) positivity was 80.5%, 18.0% and 37.4%, respectively.

Twelve patients (8.6%) could not be classified as SSc by both criteria; 5 with Raynaud’s+specific antibodies (2 anti-centromere+, 2 anti-Scl70+), 4 with Raynaud’s+puffy hands+NC abnormalities, 2 with Raynaud’s+telangiectasia and a patient with Raynaud’s+sclerodactyly. Nineteen (13.7%) patients could not be classified as SSc according to ACR (1980) can be classsified according to ACR/EULAR (2013) (table-1 and -2).

The sensitivity for ACR/EULAR (2013) and ACR (1980) criteria were found to be 91,4% vs 75,5%; 98.4% vs 96.8% in diffuse cutaneous SSc, 98.3% vs 68.3% in limited cutaneous SSc and 47.1 vs 23.5% in asclerodermic SSc, respectively.

**Conclusions:** The sensitivity of ACR/EULAR (2013) criteria was shown to be higher than ACR (1980) criteria in our Turkish SSc cohort with established cases. Although in diffuse cutaneous subgroup, the sensitivity was >%96 for both criteria, in limited cutaneous subgroup, the sensitivity was preserved for ACR/EULAR(2013) while apparently decreased for ACR(1980) criteria (<%70). The sensitivity for both of the two sets were lowest in the asclerodermic group. In SSc patients with limited or no skin involvement, non-skin manifestations, NVC findings

## P.149

## DIFFERENCES BETWEEN LATE AND EARLY-ONSET SYSTEMIC SCLEROSIS

E. Wielosz, M. Dryglewska, M. Majdan

*Department of Rheumatology and Connective Tissue Diseases Medical University, Lublin, Poland*

**Introduction:** In the majority of cases, the onset of systemic sclerosis (SSc) is observed between 30-50 years of age; however, in some cases the diagnosis is established in patients after 60-65 years of age. The course of late-onset SSc is markedly different from that in early- onset disease. The aim of the study was to compare the course of disease in patients with late-age and early-age onset SSc.

**Material and Methods:** The study was performed in 157 (119-female and 38-male) consecutive SSc patients treated in the Department of Rheumatology and Connective Tissue Diseases. All patients fulfilled the American College of Rheumatology (ACR)/ European League against Rheumatism (EULAR) classification criteria of SSc; 69 had diffuse cutaneous SSc-dcSSc and 88 limited SSc –lcSSc. The mean age in the entire study group was 58.1 ± 13.15 years (range 23-81), the duration of the disease was 5.15 ±5.41 years (range 0-23). Clinical and serological features of SSc were obtained from each patient. The SSc patients who developed the disease after the age of 60 were classified as late - onset SSc patients (n=39) and those who developed the disease before the age of 60 - as early-onset SSc patients (n=118). The subtypes of SSc, incidence of internal organ involvement, prevalence of malignancy, mortality and serological profiles were compared between the two study groups. The statistical data analysis was performed using Statistica v.13.1 .

**Results:** According to our data, the late-onset SSc was observed in 39/157 patients with SSc (approximately in 25%). Moreover, the late-onset SSc group was characterized by a significantly higher prevalence of pulmonary arterial hypertension (p=0.014), heart involvement (p=0.0014) and renal involvement (p=0.0002). The occurrence of arthralgias was less common in late-onset SSc group (p=0.02), as compared to the early-onset SSc group. The incidence of other clinical symptoms, such as interstitial lung disorder, gastrointestinal tract involvement, calcinosis, contractures, myalgia, scleroderma renal crisis, arthritis, neoplastic diseases, and overlap syndromes as well as mortality rates were comparable in both groups.

**Conclusions:** 1. Approximately 25 % of SSc patients had the onset of disease after 60 years of age

2. The course of late-onset systemic sclerosis is markedly different from that in early- onset disease.

3. Pulmonary arterial hypertension, heart involvement and renal involvement were frequently found among SSc late- onset patients whereas arthralgias were less common in SSc late –onset patients.

## P.150

## THE SCLERODERMA PATIENT-CENTERED INTERVENTION NETWORK – SCLERODERMA SUPPORT GROUP LEADER EDUCATION (SPIN-SSLED) PROGRAM: NON-RANDOMISED FEASIBILITY TRIAL

B. Thombs^1^, L. Dyas^2^, M. Pépin^1^, K. Aguila^1^, M.-E. Carrier^1^, L. Tao^1^, S. Harb^1^, V. Malcarne^3^, G. El-Baalbaki^4^, S. Peláez^1^, M. Sauve^5^, M. Hudson^1^, R. Platt^1^

^1^*Jewish General Hospital and McGIll University, Montreal, Canada*, ^2^*Scleroderma Foundation, Michigan Chapter, Southfield, USA*, ^3^*San Diego State University/University of California, San Diego, USA*, ^4^*Université du Québec à Montréal, Montreal, Canada*, ^5^*Scleroderma Society of Ontario, Hamilton, Canada*

**Introduction:** Many people with systemic sclerosis (SSc) rely on peer-led support groups for disease-specific education and emotional and practical support. Some, however, cannot access support groups. In other cases, support groups are not sustained due to factors that include demands on group leaders living with a burdensome disease and the limited organizational and group management skills of the group leaders. To address this gap, the Scleroderma Patient-centered Intervention Network (SPIN) developed a training program for SSc patient support group leaders, the Scleroderma Support group Leader EDucation (SPIN-SSLED) Program. The SPIN-SSLED Program was designed to improve confidence and self-efficacy and to reduce burden for support group leaders. Objectives of the feasibility trial were to (1) evaluate feasibility of program delivery, including required resources, management issues, and scientific aspects; and (2) assess user satisfaction and identify any modifications needed to improve program content or delivery based on participant feedback.

**Material and Methods:** Design: Non-randomised feasibility trial.

Setting: North American scleroderma patient organisations.

Participants: Current support group leaders or potential new leaders referred by patient organisations.

Intervention: The program included 13 modules delivered live via videoconference over 3 months (April to July 2018) in 60- to 90-minute sessions.

Outcome Measures: (1) Elements of feasibility, including enrolment and consent procedures, percentage of referred group leaders who consented to participate, session attendance, and technical support requirements; (2) program usability, understandability, organisation, and clarity; (3) leader satisfaction with the program; and (4) planned trial outcome measures, including support group leader self-efficacy, burnout, emotional distress, and physical function.

**Results:** All 12 referred potential participants consented to enrol, and 10 were included in 2 training groups of 5 participants each. Participants attended 95% of sessions. Required technical support was minimal, and videoconferencing technology functioned well. Overall program satisfaction rating was 9.4/10. Mean item rating on the 8 items of the Client Satisfaction Questionnaire-8 was 3.83 (1 = low satisfaction; 4 = high satisfaction). Pre-post scores on the Scleroderma Support Group Leader Self-efficacy Scale increased by 1.7 standard deviations (large effect); scores on burnout, emotional distress, and physical function improved by 0.44, 0.38, and 0.45 standard deviations (moderate effects).

**Conclusions:** The SPIN-SSLED Program was feasibly delivered, including management, resource, and scientific aspects. Participant satisfaction was high. The program is ready to be tested in a full-scale randomised controlled trial.

## P.151

## PERCEIVED BARRIERS AND FACILITATORS OF USING SYNCHRONOUS TELEREHABILITATION OF PHYSICAL AND OCCUPATIONAL THERAPY IN MUSCULOSKELETAL DISORDERS: A SCOPING REVIEW

L. Tao^1^, L. Kwakkenbos^2^, A. Carboni-Jiménez^1,3^, K. Turner^1,3^, K. Aguila^1^, J. Boruff^4^, S. Ahmed^5^, B. Thombs^1,3,6,7^

^1^*Jewish General Hospital - Lady Davis Institute for Medical Research, Montreal, Canada*, ^2^*Behavioral Science Institute, Clinical Psychology, Radboud University Nijmegen, Nijmegen, The Netherlands*, ^3^*McGill University - Department of Psychiatry, Montreal, Canada*, ^4^*McGill University - Life Sciences Library, Montreal, Canada*, ^5^*McGill University - School of Physical & Occupational Therapy, Montreal, Canada*, ^6^*McGill University - Department of Medicine, Montreal, Canada*, ^7^*McGill University - Department of Psychology, Montreal, Canada*

**Introduction:** Rehabilitation interventions, such as physical therapy (PT) and occupational therapy (OT), play a crucial role in limiting disability and improving health-related quality of life in people with musculoskeletal disorders. People with rare diseases, including systemic sclerosis (SSc; scleroderma), however, often have difficulty accessing appropriate services. E-health interventions delivered via videoconferencing with a healthcare professional are increasingly common and can be an effective way to overcome barriers to delivering PT/OT interventions in a rare disease context. The Scleroderma Patient-centered Intervention Network (SPIN) is an international collaboration dedicated to developing, testing, and disseminating internet-based, patient-centered interventions for people living with SSc. The SPIN infrastructure is an ideal framework to develop and test a telerehabilitation intervention to address function limitations in SSc. However, knowledge is needed on possible facilitators and barriers of successful use of telerehabilitation. Thus, the objective of this scoping review was to identify barriers and facilitators of using synchronous telerehabilitation to deliver PT/OT interventions for musculoskeletal disorders, to inform the development of a telerehabilitation intervention for SSc.

**Material and Methods:** MEDLINE, EMBASE, CINAHL, PsycInfo, Cochrane Library, and Proquest Dissertations and Theses databases were searched from inception until August 25, 2017. Publications that described perceived barriers and facilitators of synchronous telerehabilitation in patients with musculoskeletal disorders were eligible for inclusion. Two investigators independently evaluated titles/abstracts and full-text publications for eligibility, and extracted data from each included publication.

**Results:** The database searches yielded 1330 unique citations. Of these, 1165 articles were excluded after the title/abstract review, leaving 165 publications for full-text review to further assess their eligibility. Of these, 18 publications were included in the review. In total, 52 different barriers were identified. The most commonly reported barriers were technological issues, privacy concerns, cost of attendance, and patient’s lack of confidence. Among the 71 facilitators identified, the most commonly reported were convenience and accessibility of services, audio and visual clarity, time savings, and financial savings.

**Conclusions:** Videoconferencing can be an effective tool to deliver PT/OT interventions for musculoskeletal diseases, including SSc, because it improves accessibility of services for patients with its high-quality, ease-to-use features. Strategies to combat barriers to using telerehabilitation include using a low-cost, stable and high-quality videoconferencing platform, enhancing self-efficacy to using videoconferencing, and addressing privacy concerns. The knowledge obtained from this study will inform the development and evaluation of a telerehabilitation program for SSc patients.

## P.152

## CHARACTERISTICS OF CRP POSITIVE SYSTEMIC SCLEROSIS (SSC)

K. Takagi^1^, Y. Kawaguchi^2^, T. Higuchi^2^

^1^*Tokyo Women’s Medical University Medical East, Tokyo, Japan*, ^2^*Tokyo Women’s Medical University, Tokyo, Japan*

**Introduction:** Some of the patients with systemic SSc exhibit elevated levels of CRP. However, roles of CRP in the clinical features of SSc remains controversial. The purpose of this study is to clarify the characteristics of CRP-positive SSc patients.

**Material and Methods:** Four hundred and twenty-five SSc patients were involved in this study. The cases accompanied by infection were excluded. Clinical features were compared between CRP-positive 100 cases and CRP-negative 318 cases.

**Results:** The frequency of arthritis (p 0.00658, OR 2.109), interstitial pneumonia (IP) (p 0.00163, OR 2.166), pulmonary hypertension (PH) (p 0.01289, OR 2.525) and lower gastrointestinal tract failure (p 0.02999, OR 2.837) was significantly higher in the CRP-positive group. The patients with rheumatoid arthritis (p=0.00389, OR 3.114), systemic lupus erythematosus (p 0.00771, OR 4.153) and anti-DNA antibody positivity (p 0.04365, OR 2.536) was also significantly higher in the CRP-positive group. The frequency of TBX21 rs11650354 CC genotype was not significantly different between these groups.

**Conclusions:** SSc patients with an elevated level of CRP positive was associated with IP or PH that determine poor prognosis. This therefore suggests CRP is a useful marker to predict for the prognosis or disease activity of SSc.

## P.153

## RELIABILITY OF WORLD SCLERODERMA FOUNDATION (WSF) DEFINITION OF SKIN ULCERS IN SCLERODERMA, RESULTS FROM THIRD CONSENSUS MEETING

Y. Suliman^1^, C. Bruni^2^, E. Praino^3^, D. Khanna^4^, Y. Allanore^5^, A. L Herrick^6^, C. Denton^7^, M. Matucci-Cerinic^8^, D. E. Furst^9^

^1^*Rheumatology and Rehabilitation Dept., Assiut University Hospital, Assiut, Egypt*, ^2^*Department of Experimental and Clinical Medicine, Division of Rheumatology, University of Florence, Firenze, Italy, Firenze, Italy*, ^3^*DIM, Rheumatology Unit - D.E.T.O. - University of Bari (ITALY), Bari, Italy, Bari, Italy*, ^4^*Division of Rheumatology, Department of Internal Medicine, University of Michigan Scleroderma Program, University of Mic, Michigan, USA*, ^5^*Service de Rhumatologie A, Hôpital Cochin, Paris, France, Paris, France*, ^6^*Centre for Musculoskeletal Research, Division of Musculoskeletal and Dermatological Sciences, The University of Manchest, Manchester, United Kingdom*, ^7^*UCL Division of Medicine, Royal Free Campus, London, United Kingdom, London, United Kingdom*, ^8^*Department of Experimental and Clinical Medicine, University of Florence, Florence, Italy, Florence, Italy*, ^9^*Medicine, University of California, Los Angeles, David Geffen School of Medicine, Los Angeles, CA, California, USA*

**Introduction:** Development of a consistent uniform validated definition of systemic sclerosis related skin ulcer (SSc-skin ulcer) for use in clinical trials is of utmost importance. Our group previously published a consensus derived definition of SSc-skin ulcer under the patronage of WSF(1) . The definition was constructed on the basis of a systematic literature review followed by a nominal group technique to establish a consensus.

Face validity and feasibility were well demonstrated. We further evaluated the reliability of the WSF definition . Forty ulcer pictures were emailed twice/investigator to test inter- and intra-rater reliability, the results showed excellent intra-rater while only fair agreement for Inter-rater Fleiss Kappa was 0.3.

**Aims:**

Step I : Re-evaluate the WSF definition and reporting any additional remarks to improve its ability to identify/exclude ulcers for SSc clinical trials.

Step II:Examine the percent agreement with a gold standard scoring of the images

Step III: Examine the intra-rater and inter-rater reliability of the WSF definition among investigators.

**Material and Methods:** A face to face meeting was conducted during EULAR 2019, where 8 SSc experts with different levels of SSc experience participated, 20 minutes discussion period were allowed to discuss any remarks regarding the WSF definition. Forty images were utilized, to be scored by each investigator twice for inter and intra rater agreement. And to be compared with a gold standard scores ( SSc Expert)

**Results:** Step I:

Modifications to the WSF skin ulcer definition were added, The ulcer site was added (to be mainly digital ulcers ), elbow, forearms or lower limbs ulcers were excluded. Additional modification was to include the wording: “ included in a clinical trial” within the definition. A special remark ensured the exclusion of any infected ulcer o which seems to be infected in a relevant clinical setting.

Step II: Percent agreement with Gold standard scores varied from (43%-75%)

Step III Intra-rater reliability using ICC was 0.99 (excellent)

Inter-rater reliability using Fleiss Kappa was 0.27 ( fair, given a range of 0-1.0).

**Conclusions:** We clarified the definition of SSc ulcers to be included into a clinical trial based on consensus. Among a group of rheumatologists with varying SSc experience, Reliability exercises showed the need for repeated training sessions prior to embarking in a clinical trial, because this may decrease variability among investigators. As mRSS, it will be necessary in clinical trials to have the same investigator examining a given patient over time, as inter-rater reliability is not sufficient

**Figure:**
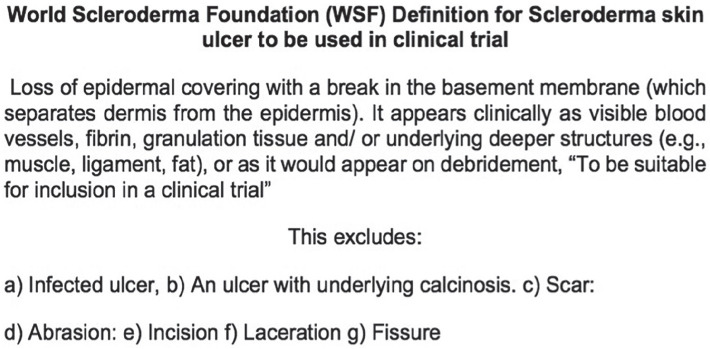


## P.154

## ENERGY LEVEL : AN OVERLOOKED FACTOR IN ASSESSMENT OF SCLERODERMA PATIENTS

Y. Suliman^1^, S. Kafaja^2^, M. Alemam^3^, D. E Furst^4^

^1^*Rheumatology and Rehabilitation, Assiut university hospital, Assiut, Egypt*, ^2^*Rheumatology Division, Dept of Medicine, UCLA, California, USA*, ^3^*Laboratory medicine, South valley university, Qena, Egypt*

**Introduction:** Scleroderma (SSc) is a chronic multi-system autoimmune disease which directly affects health related Quality of Life. We evaluated how the patient comprehends and translates their inability to work and their perspective regarding energy levels during the daily routines. We hypothesized that there are abnormal constitutional symptoms in SSc patients (i.e. energy levels, sleep quality and time to feeling fatigued), that may directly affect patient’s quality of life.

**Aim**

1-To evaluate the proportion of SSc patients with abnormal energy levels, sleep and with shorter time to fatigue.

2-To assess the relation between energy levels and quality of sleep, time to fatigue, pain visual analogue scale (VAS), Pt global VAS, MD global VAS

**Material and Methods:** SSc patients meeting the ACR/EULAR 2013 SSc criteria were recruited from Pacific Arthritis Scleroderma Clinic. Laboratory and clinical data were obtained from charts for statistical correlations..

Energy levels were weighed by the question ‘how is your energy?’ and Sleep was assessed as: How well do you sleep?” or “How is your sleep?” For these questions, answers were categorical: “Good” (0), “OK” (1), “Fair”(2) or” Low”(3).

Fatigue was questioned as “how long after you get up, you start to get tired or fatigued?”. Answers were in hours, including 0 for fatigued immediately upon awakening to 12+(maximum); responses were in 0.5-hour increments. Pearson correlation to evaluate energy levels correlation with other PRO and QOL measures. Regression model was further created with energy levels as dependent variable and time to fatigue, sleep quality pain VAS, pt global MD global as independent variables

**Results:** Eighty -five SSc patients’ characteristics were as follows: mean age 55 (13%), female 76

ILD 37(43%), mean MRSS 7 (±7.5) Table 1. Energy levels were fair-poor in more than 50%, while significantly shortened time to fatigue (<=3 hrs) occurred in 29(34%). Sleep quality was only fair-poor in 37/85(43%) Table 2.

Regression model detected sleep, fatigue, pain VAS, patient global and MD as strong predictors of Energy levels in SSc pts (p =0.0001, 0.0001, 0.009,0.0001 and 0.0001 respectively).

**Conclusions:** Energy status, amongst other constitutional symptoms (ie, sleep and fatigue ) are significantly altered in SSc patients, more care is to be given to address potential causes, measurement tools and management plans.

**Figure:**
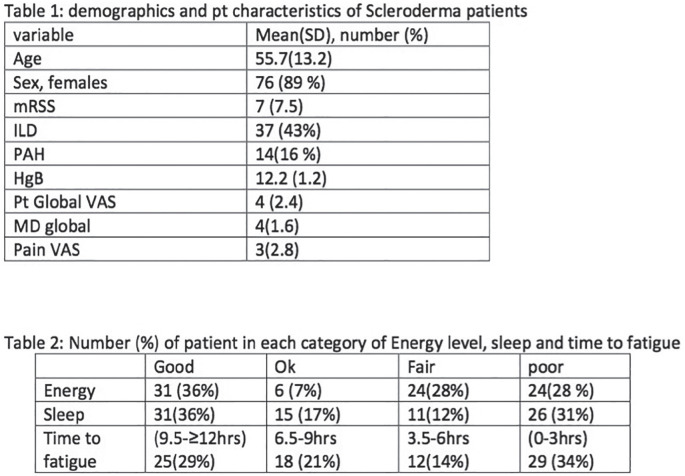


## P.155

## HOW DO PATIENTS WITH SYSTEMIC SCLEROSIS EXPERIENCE CURRENTLY PROVIDED HEALTH CARE AND HOW SHOULD WE MEASURE ITS QUALITY?

J. Spierings^1^, C. Van Den Ende^2,3^, R. Schriemer^4^, H. Bernelot Moens^5^, E. Van Der Bijl^6^, F. Bonte-Mineur^7^, M. De Buck^8^, M. De Kanter^9^, H. Knaapen-Hans^3^, J. Van Laar^1^, U. Mulder^10^, J. Potjewijd^11^, L. De Pundert^12^, T. Schoonbrood^13^, A. Schouffoer^14^, A. Stel^15^, W. Vercoutere^16^, A. Voskuyl^17^, J. De Vries-Bouwstra^18^, M. Vonk^3^

^1^*Department of Rheumatology and Clinical Immunology, University Medical Centre Utrecht, Utrecht, The Netherlands*, ^2^*Department of Rheumatology, Sint Maartenskliniek, Nijmegen, The Netherlands*, ^3^*Department of Rheumatology, Radboud University Medical Center, Nijmegen, The Netherlands*, ^4^*NVLE, Dutch patient organization for systemic autoimmune diseases, Utrecht, The Netherlands*, ^5^*Department of Rheumatology, Ziekenhuisgroep Twente, Hengelo, The Netherlands*, ^6^*Department of Rheumatology, Isala, Zwolle, The Netherlands*, ^7^*Department of Rheumatology, Maasstad ziekenhuis, Rotterdam, The Netherlands*, ^8^*Department of Rheumatology, Haaglanden, The Hague, The Netherlands*, ^9^*Department of Rheumatology, Elisabeth-TweeSteden Ziekenhuis, Tilburg, The Netherlands*, ^10^*Department of Internal Medicine, division Vascular Medicine, University of Groningen, Groningen, The Netherlands*, ^11^*Department of Clinical immunology, Maastricht University Medical Center+, Maastricht, The Netherlands*, ^12^*Department of Physical Therapy, Haga Ziekenhuis, The Hague, The Netherlands*, ^13^*Department of Rheumatology, Maastricht University Medical Center+, Maastricht, The Netherlands*, ^14^*Department of Rheumatology, Haga Ziekenhuis, The Hague, The Netherlands*, ^15^*Department of Rheumatology, University Medical Center Groningen, Groningen, The Netherlands*, ^16^*Department of Rheumatology, Zuyderland Medical Center, Heerlen, The Netherlands*, ^17^*Amsterdam Rheumatology and Immunology Center, Amsterdam UMC, loc. VUMC, Amsterdam, The Netherlands*, ^18^*Department of Rheumatology, Leiden University Medical Centre, Leiden, The Netherlands*

**Introduction:** Providing optimal care for patients with systemic sclerosis (SSc), can be challenging. Patients may present with different signs and symptoms, and may experience high morbidity as well as increased mortality. Medical treatments are applied with varying results and the patient’s journey towards diagnosis and treatment varies greatly among individual patients. Evaluation of the quality of the currently provided care can help to identify aspects for improvement. However, their is no uniform way to evaluate the quality of care in SSc. Two Delphi exercises with physicians resulted in a list of preferred process indicators. This is an important first step in making quality of SSc care tangible. However, criteria for quality of care from the perspective of patients with SSc are still missing and would be of value.

The aim of this study was to gain insight in systemic sclerosis (SSc) patients’ perspective of quality of care in the Netherlands, and to survey the preferred quality indicators.

**Material and Methods:** An online questionnaire about health care setting, perceived quality of care (CQ index) and quality indicators, was sent to 2,093 patients with SSc from 13 Dutch hospitals.

**Results:** 650 patients (mean age 59 years, 75% women, 32% limited cutaneous SSc, 20% diffuse cutaneous SSc) completed the questionnaire. Mean time to diagnosis was 4.3 years (SD 6.9) and was longer in women compared to men (4.8 (SD 7.3) versus 2.5 (SD 5.0) years). Treatment took place in a SSc expert center in 58%, regional center in 29% or in both in 39% of patients. 13% of patients was not aware if their hospital was specialized in SSc. The perceived quality of care was rated with a mean score of 3.2 (SD 0.5) (range 1.0 - 4.0). There were no relevant differences between expert and regional centers. The three prioritized process indicators were: good patient-physician interaction (80%), structural multidisciplinary collaboration (46%) and receiving treatment according to SSc guidelines (44%). Absence of disease progression (66%), organ involvement (33%) and digital ulcers (27%) were the three highest rated outcome indicators (figure 1).

**Conclusions:** The perceived quality of care evaluated in our study was fair to good. No differences between expert and regional centers were observed. Our prioritized process and outcome indicators can be added to indicators suggested by SSc experts in earlier studies and can be used to evaluate the quality of care in SSc.

**Figure:**
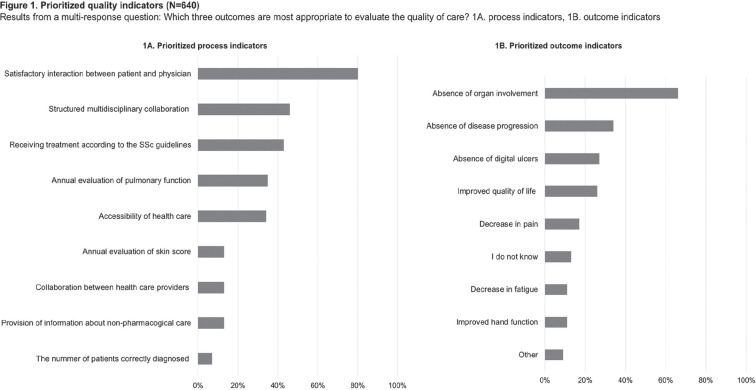


## P.156

## TREATMENT DECISION-MAKING IN DIFFUSE CUTANEOUS SYSTEMIC SCLEROSIS: A PATIENT’S PERSPECTIVE

J. Spierings^1^, F. Van Rhijn- Brouwer^1,2^, C. De Bresser^1^, P. Mosterman^3^, A. Pieterse^4^, M. Vonk^5^, A. Voskuyl^6^, J. De Vries-Bouwstra^7^, M. Kars^8^, J. Van Laar^1^

^1^*Department of Rheumatology and Clinical Immunology, University Medical Centre Utrecht, Utrecht, The Netherlands*, ^2^*Department of Nephrology and Hypertension, Regenerative Medicine Center Utrecht, University Medical Center, Utrecht, The Netherlands*, ^3^*Patient Sounding Board of the Department of Rheumatology and Clinical Immunology, University Medical Center Utrecht, Utrecht, The Netherlands*, ^4^*Department of Medical Decision Making, Leiden University Medical Center, Leiden, The Netherlands*, ^5^*Department of Rheumatology, Radboudumc, Nijmegen, The Netherlands*, ^6^*Amsterdam Rheumatology and Immunology Centre, Amsterdam UMC- location VU, Amsterdam, The Netherlands*, ^7^*Department of Rheumatology, Leiden University Medical Center, Leiden, The Netherlands*, ^8^*Center of Expertise Palliative Care, Julius Center for Health Sciences and Primary Care, University Medical Center Utrec, Utrecht, The Netherlands*

**Introduction:** Autologous hematopoietic stem cell transplantation (HSCT) is increasingly performed in diffuse cutaneous systemic sclerosis (dcSSc). HSCT has been shown to lead to superior outcomes with regard to survival, quality of life, skin fibrosis and prevention of disease progression in comparison with intravenous cyclophosphamide. There are, however, no specific guidelines on patient selection or optimal timing for HSCT, and the position of HSCT as treatment regimen compared to other available immunosuppressive therapies is unclear. Therefore, the treatment choice is primarily based on the preferences of the patient and rheumatologist. It is important that rheumatologists and multidisciplinary teams treating patients with dcSSc, provide optimal guidance in making this treatment decision. The aim of this study was to improve understanding of the decision-making process in dcSSc from a patient’ perspective and to develop recommendations that support shared decision-making.

**Material and Methods:** A qualitative semi-structured interview study was done in a purposefully selected heterogenous patient sample. Thematic analysis was used. Shared decision-making (SDM) was assessed with the SDM-Q-9.

**Results:** Twenty-five patients were interviewed: five pre-HSCT, 16 post-HSCT and four following other treatment. Six recurrent aspects influencing decision-making were identified; health status, poor prospects, knowledge, expectations, patient-physician interaction and social interaction. Treatment decisions were predominantly made by the rheumatologist. Still, patients perceived the decision to be shared (mean SDM-Q-9 81/100 (range 15.6)). Seventeen patients felt they had no choice, and more than half of the patients anticipated HSCT was the only option to avert premature death. Additionally, rapid deterioration of health contributed to their feelings of having no other options left. Importantly, patients reported that they could not oversee and balance all available therapeutic options due to lack of accessible, reliable and SSc-specific information. Expectations of the effects of HSCT differed. Remarkably, male patients generally believed they had lower risks of treatment complications compared to other patients. Lastly, patients struggled sharing information and feelings with loved ones, which hindered essential social support in the decision-making. Peer support was highly appreciated.

**Conclusions:** Decision-making in dcSSc is negatively impacted mainly by a lack of accessible, reliable, disease-specific education about treatment options. Clinicians should explore patient’s preferences and background knowledge to optimise the treatment decision-making process and to provide tailormade information on HSCT.

**Figure:**
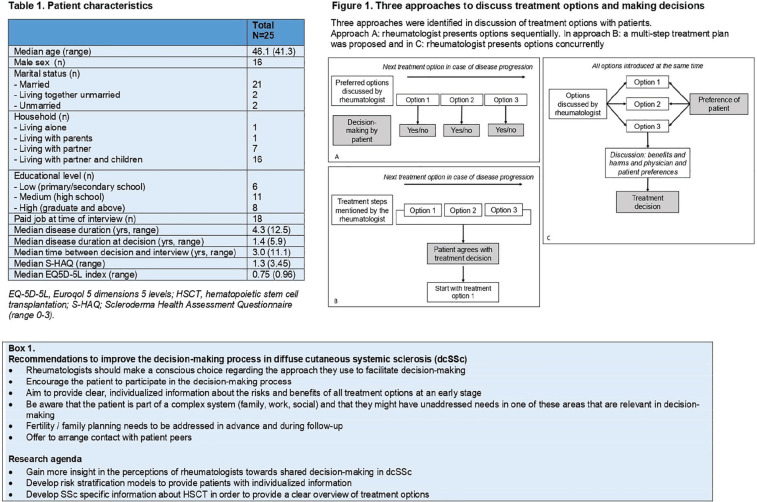


## P.157

## REVIEW OF MALIGNANCIES IN OUR WAIKATO COHORT OF PATIENTS WITH SYSTEMIC SCLEROSIS

K.K. Solanki^1^, M. Rees^2^, C. Frampton^3^, D. White^1^

^1^*Waikato Hospital, Hamilton, New Zealand*, ^2^*Biomedical Engineering Dept, Auckland University, Auckland, New Zealand*, ^3^*Christchurch School of Medicine, University of Otago, Christchurch, New Zealand*

**Introduction:** There is an increased risk of malignancy in Systemic sclerosis (SSc). It has been noted in some studies to be associated with certain SSc specific antibodies and other studies have shown a preferential increase in the diffuse subtype of SSc.

We aimed to to assess the types of malignancies in our cohort of Systemic Sclerosis and the association with the antibody profiles and the two main subtypes.

**Material and Methods:** Waikato Hospital Systemic Sclerosis Clinics held regularly, and after ethics approval specific data were collected and assessed in association with the NZ National Cancer Registry. Data. Standardised incidence rates (SIRs) and Fisher exact tests where appropriate were evaluated.

**Results:** Our cohort compromised of 164 patients of which 32 developed a malignancy (19.5%).

The most common form of malignancy was in the skin (14, 43.75%), followed by breast (6, 18.75%) and then lymphoma (5, 15.63%). There was not enough evidence to suggest increased lung malignancy in our cohort.

Overall SIR was 2.25 (95% CI 1.4-3.4) and it was a 2-fold increased for men (4.40, 95% CI 1.4-10.3). No significant difference in the SIRs was noted between the dcSSc and lcSSc patients (2.25 and 2.35). Nevertheless, it was noted that the average age of patients with malignancy was around 8 years older than patients without.

**Conclusions:** Our study showed an increased risk of malignancy for patients within our Waikato SSc cohort. Our study did not show increased lung malignancies as seen in few other studies.

Similarly, like some previous studies, we did not see any preferential increase in malignancies in patients with RNA Polymerase antibodies over the non-RNA Polymerase SSc antibodies nor was it increased in dcSSc compared to lcSSc. Importantly, our patients with malignancies were noted to be on average of 8 years older than those without.

## P.158

## HEALTH ASSESSMENT QUESTIONNAIRE DISABILITY INDEX (HAQ-DI) AND PATIENT GLOBAL ASSESSMENT OF HEALTH (PTGA) CORRELATE WITH CHANGES IN PATIENT-REPORTED OUTCOMES (PROS)

R. Spiera^1^, L. Hummers^2^, L. Chung^3^, T. Frech^4^, R. Domsic^5^, V. Hsu^6^, D. Furst^7^, J. Gordon^1^, M. Mayes^8^, R. Simms^9^, E. Lee^10^, S. Constantine^10^, B. Conley^10^, Q. Dinh^10^, B. Bloom^10^, B. White^10^

^1^*Weill Cornell Medical College, New York City, USA*, ^2^*Johns Hopkins University School of Medicine, Baltimore, USA*, ^3^*Stanford University School of Medicine and Palo Alto VA Health Care System, Palo Alto, USA*, ^4^*University of Utah and Salt Lake City VA Health Care System, Salt Lake City, USA*, ^5^*University of Pittsburgh School of Medicine, Pittsburgh, USA*, ^6^*Rutgers Robert Wood Johnson Medical School, New Brunswick, USA*, ^7^*Arthritis Association of Southern California, Los Angeles, USA*, ^8^*University of Texas Health Center at Houston, Houston, USA*, ^9^*Boston University School of Medicine, Boston, USA*, ^10^*Corbus Pharmaceuticals, Inc., Norwood, USA*

**Introduction:** Health Assessment Questionnaire Disability Index (HAQ-DI) and Patient Global Assessment of Health (PtGA) are the most consistently used patient-reported outcomes (PRO) in recent trials in diffuse cutaneous systemic sclerosis (dcSSc). There is limited information on whether HAQ-DI and PtGA baseline and change scores correlate with each other and other PROs used in trials. The hypothesis was HAQ-DI scores would correlate with PROs that assess patent function and PtGA would correlate with PROs that assess patient symptoms.

**Material and Methods:** Baseline and change (δ) scores for HAQ-DI and PtGA were correlated (Spearman) with each other, PROMIS-29 domain scores, and Systemic Sclerosis Skin Symptoms Patient-reported Outcome (SSPRO) scores in the double-blind placebo-controlled (Part A) study of lenabasum in SSc (N = 42) and its open-label extension (OLE, N = 36). Change scores were correlated at Month 3 of Part A (N = 38) and Months 6, 12, 18, and 24 (N = 36, 31, 30, and 24, respectively) of the OLE. For descriptive purposes in this abstract, correlations coefficients (r) were categorized as no (0 to 0.19), weak (0.20 to 0.34), moderate (0.35 to 0.59), strong (0.60 to 0.79) correlations, and very strong (>= 0.80) correlations.

**Results:** All correlations were directionally correct (Table 1). All moderate, strong, or very strong correlations were statistically significant, P <= 0.05, except P = 0.06 for a few moderate correlations. Baseline HAQ-DI and PtGA values correlated moderately or strongly with each other and all other PRO except PROMIS-29 sleep disturbance in Part A, but not the OLE. HAQ-DI and PROMIS-29 physical function were redundant at baseline in Part A, r = 0.80. Change in HAQ-DI consistently correlated most strongly with δ PROMIS-29 physical function and social role in Part A and the OLE (P < 0.01), except not with δ social role at 3 months in Part A. δ PtGA correlated moderately and occasionally strongly with δ patient symptoms as assessed by δ PROMIS-29 anxiety, depression, fatigue, and pain interference domains in Part A and the OLE, at all visits.

**Conclusions:** The correlations between changes in HAQ-DI and other patient-reported functioning and between change in PtGA and patient-reported symptoms were notable for their consistency over 2 years in this study. Both δ HAQ-DI and δ PtGA are included in the calculation of the ACR Combined Response Index in diffuse cutaneous Systemic Sclerosis (CRISS) score, suggesting this composite score may reflect both patient function and symptoms.

**Figure:**
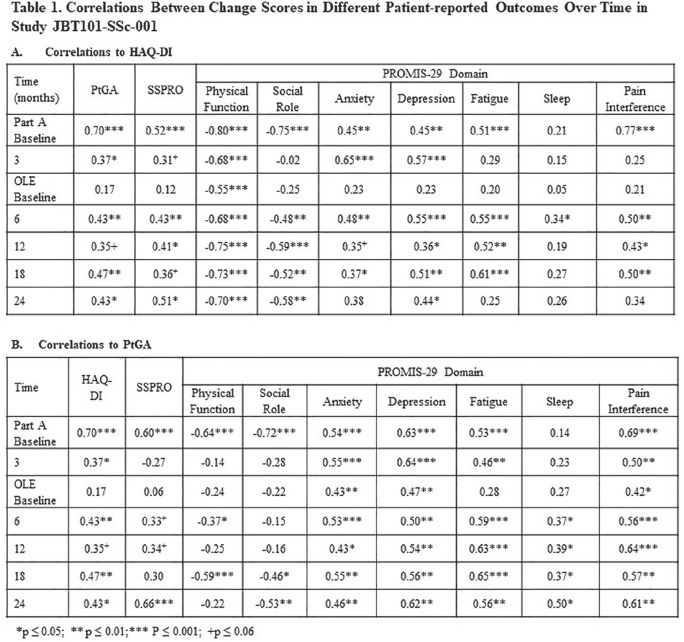


## P.159

## PATIENT AND PHYSICIAN OPINIONS OF CLINICAL BENEFIT AT 3 MONTHS IN A CLINICAL TRIAL CORRELATE WITH PATIENT-REPORTED OUTCOMES (PROS)

R. Spiera^1^, L. Hummers^2^, L. Chung^3^, T. Frech^4^, R. Domsic^5^, V. Hsu^6^, D. Furst^7^, J. Gordon^1^, M. Mayes^8^, R. Simms^9^, E. Lee^10^, S. Constantine^10^, B. Conley^10^, Q. Dinh^10^, B. Bloom^10^, B. White^10^

^1^*Weill Cornell Medical College, New York City, USA*, ^2^*Johns Hopkins University School of Medicine, Baltimore, USA*, ^3^*Stanford University School of Medicine and Palo Alto VA Health Care System, Palo Alto, USA*, ^4^*University of Utah and Salt Lake City VA Health Care System, Salt Lake City, USA*, ^5^*University of Pittsburgh School of Medicine, Pittsburgh, USA*, ^6^*Rutgers Robert Wood Johnson Medical School, New Brunswick, USA*, ^7^*Arthritis Association of Southern California, Los Angeles, USA*, ^8^*University of Texas Health Center at Houston, Houston, USA*, ^9^*Boston University School of Medicine, Boston, USA*, ^10^*Corbus Pharmaceuticals, Inc., Norwood, USA*

**Introduction:** A SSc patient’s and treating physician’s opinion of clinical benefit early in treatment are each likely important in maintaining adherence and persistence on therapy thereafter in the real-world setting. Disease-specific and non-disease-specific PROs are used to assess how people with SSc feel and function. The hypothesis of this evaluation was that both the subject’s and physician’s opinion of clinical benefit early in treatment (3 months) in a lenabasum phase 2 study would correlate with change in PROs.

**Material and Methods:** Spearman correlations were performed between a subject’s and treating physician’s opinion “yes/no” on whether the subject had received clinical benefit from study product and change (δ) in PROs and efficacy outcomes at 3 months in a double-blind placebo-controlled phase 2 study of lenabasum (JBT101-SSc-001), with 38 (88.5%) subjects completing 3 months dosing. The PROs included HAQ-DI, Patient Global Assessment of Health related to SSc (PtGA), Scleroderma Skin Symptoms Patient-reported Outcome (SSPRO) questionnaire, and PROMIS-29 questionnaire domain T-scores for physical function, social role, fatigue, sleep disturbance, pain interference, anxiety, and depression domains. ACR CRISS score and δ mRSS were obtained.

**Results:** Subject opinion of clinical benefit at 3 months moderately correlated in a statistically significant manner with δPtGA and δ SSPRO, but not δ HAQ-DI or δ PROMIS-29 physical function, social role, and pain domains. Subject opinion of benefit at 3 months had low, not statistically significant, correlations with ACR CRISS score and δ mRSS. Physician opinion of clinical benefit at 3 months correlated moderately and statistically significantly or near statistically significant with δ PtGA, δ SSPRO, δ PROMIS-29 social role and pain domains, as well as efficacy outcomes (ACR CRISS and δ mRSS).

**Conclusions:** Both the subject’s and physician’s opinion of clinical benefit early in treatment at 3 months correlated with the subject’s overall assessment of health related to SSc and change in skin symptoms. Larger and longer studies will help further elucidate what PROs reflect a SSc patient’s assessment of clinical benefit from treatment.

**Figure:**
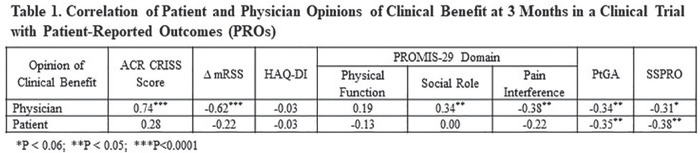


## P.160

## CHANGES IN INITIAL CLINICAL PRESENTATIONS OF SYSTEMIC SCLEROSIS OVER 25 YEARS OF OBSERVATION

V. Semenov^1^, I. Molokova^1^, T. Lysunets^2^, O. Kuryata^1^

^1^*Dnipropetrovsk Medical Academy - Department of internal medicine 2, Dnipro, Ukraine*, ^2^*Mechnikov Dnipropetrovsk Regional Hospital - Department of rheumatology, Dnipro, Ukraine*

**Introduction:** Systemic sclerosis (SSc) is an autoimmune disorder with a wide variety of clinical manifestations. Its heterogeneity causes difficulties in diagnosis establishment and leads to revisions of diagnostic criteria. The aim: to analyze clinical presentations of patients with SSc at the moment of diagnosis establishment depending on the time of recruitment to the local registry.

**Material and Methods:** 217 patients (18 males and 199 females) were recruited to the local registry of patients with SSc. Patients with Raynaud’s phenomenon (RP) and localized SSc were excluded from the study. Patients in the analysis (12 males and 171 females) were divided into 5 groups according to the year of SSc diagnosis: 1993-1998, 2001-2004, 2005-2008, 2009-2012 and 2014-2018. Median age (interquartile range), frequency of diagnosed RP, skin thickening, pulmonary fibrosis, hypertension, C-reactive protein (CRP)>5 mg/l and rheumatoid factor (RF)>14 IU/ml for each year were analyzed. In purpose to reflect values for time intervals means of age and symptoms’ frequencies were calculated. All the patients receive medical care according to European and local standards. Data only for the moment of SSc diagnosis were analyzed.

**Results:** Median age of patients at the moment of SSc diagnosis for analyzed periods was 47 [39;52], 47 [40;51], 46 [41;51], 44 [40;49] and 41 [38;44] years. Frequencies of some SSc manifestations remained stable or changed randomly: RP (79.1%, 85.9%, 92.2%, 94.4% and 85.9%) and skin thickening (60.4%, 52.3%, 62.5%, 72.6% and 50.8%). Arthritis and hypertension showed trends on decrease of frequency: 87.5%, 76.8%, 82.4%,83.9% and 71.8% for arthritis; 62.5%, 33.9%, 17.2%, 11.3% and 25.6% for hypertension. Following manifestations showed rise of frequency: pulmonary fibrosis (12.5%, 22.0%, 25.3%, 32.1% and 16.5%), proteinuria (25.0%, 28.5%, 34.9%, 44.6% and 29.9%), CRP>5 mg/l (0%, 47.5%, 58.9%, 30.9% and 36.6%) and RF>14 IU/ml (0%, 3.1%, 11.8%, 35.0% and 37.5%). All the data represent following time intervals respectively: 1993-1998, 2001-2004, 2005-2008, 2009-2012 and 2014-2018.

**Conclusions:** Clinical pattern of patients with SSc changed sufficiently over 25 years. The most prominent changes were a rise of inflammatory activity (elevated CRP and RF), an abrupt decrease of hypertension incidence and on average 6 years earlier onset of SSc.

## P.161

## DESIGNING DUTCH RECOMMENDATIONS ON NONPHARMACOLOGIC INTERVENTIONS FOR PATIENTS WITH SYSTEMIC SCLEROSIS

M.R. Schriemer^1^, J. Spierings^2^, C. Van Den Ende^3^, L. De Pundert^4^, M. Vonk^5^, J. De Vries-Bouwstra^6^, S. Dittmar^1^

^1^*NVLE, Dutch Organization for systemic auto-immune disease, Utrecht, The Netherlands*, ^2^*Department of Rheumatoloy and clinical immunology, University Medical Center Utrecht, Utrecht, The Netherlands*, ^3^*Department of Rheumatology, St Maartenskliniek, Nijmegen, The Netherlands*, ^4^*Department of Physcal Therapy, Haga Ziekenhuis, The Hague, The Netherlands*, ^5^*Department of Rheumatology, Radboud University Medical Center, Nijmegen, The Netherlands*, ^6^*Department of Rheumatology, Leiden University Medical Center, Leiden, The Netherlands*

**Introduction:** Systemic Sclerosis (SSc) is a rare autoimmune disease with multiple organ involvement, impaired hand and mouth function and limitations such as fatigue and pain. Due to lack of treatment options and the unpredictable disease course, SSc is challenging for patients. Nonpharmacologic interventions can relieve some disease burdens. To improve awareness and stimulate the use of these interventions, the ARCH study group – consisting of clinicians, health care professionals and patients - embarked on the establishment of HCP recommendations for nonpharmacologic interventions for SSc patients.

**Material and Methods:** For the creation of such recommendations, a combination of literature review and consensus was adopted. At first, previous attempts and an unfinished and unpublished concept report on nonpharmacologic interventions were collated. We then we asked - and were given -consent from the chair/main author from the unfinished product to extend and finish the project. The previous co-authors were re-invited to join the working group on the new recommendations. We then selected additional working group members (table 1), such as consultants from non-specialist care hospitals and experienced health care professionals from clinical or non-clincal settings. These HCPs includes dietitians, mount hygienists, social workers, occupational therapists and psychologists. We held a conference call with the new working group in which we requested consent on new working procedure by using 15+ starting questions describing primary symptoms or daily lives problems for SSc patients, after which we collated evidence for these interventions and frequently given nonpharmacologic advice given by the members.

**Results:** We are currently preparing the consensus meeting for November 1st 2019. We are collating evidence and frequently provided advise to put to the members at the consensus meeting. The working group selected additional invitees, such as a speech therapist, skin therapist and patient representatives. After reaching consensus on these recommendations, an article will be published and the recommendations will be disseminated among hospitals, health care professionals and patients. Additionally, a tool will be designed to improve referrals to health care professionals.

**Conclusions:** There is support and demand for de development for a nonpharmacologic care plan for SSc patients based on evidence and/or clinical experiences and by consensus, by clinician’s, health care providers and patients. Although the document is not yet finished, the approach to reach consensus could be implemented in other countries. It improves the knowledge of and access to this part of care which can relieve patients of certain disease burdens for which there are no known pharmacological remedies.

**Figure:**
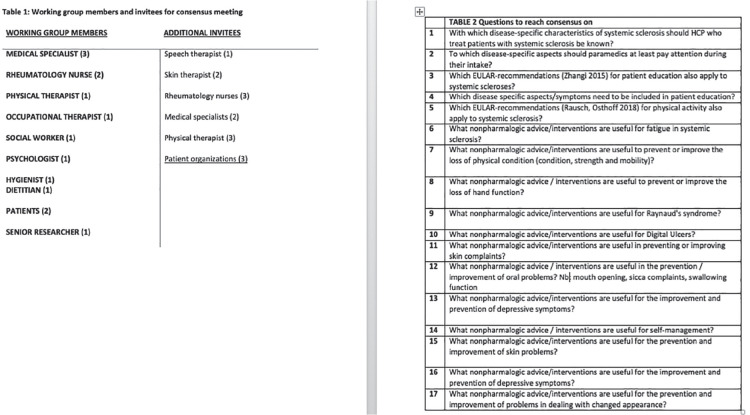


## P.162

## CONCURRENT AUTOIMMUNE DISORDERS AND THEIR IMPACT ON CLINICAL PRESENTATION IN A COHORT OF SYSTEMIC SCLEROSIS PATIENTS

C. Rotondo, A. Corrado, G. Bellantuono, F.P. Cantatore

*Department of Medical and Surgical Sciences - Rheumatology Unit - University of Foggia - OORR, Foggia, Italy*

**Introduction:** The concurrence of non-rheumatic autoimmune comorbidity (nRAD) in Systemic sclerosis (SSc) is described in previous studies. How the nRAD presence could affect the clinical presentation of SSc has never been studied. The aims of this work are to assess the nRAD frequency in patients (pts) with SSc and to evaluate the nRAD impact on clinical features related to SSc.

**Material and Methods:** A total of 92 SSc-pts, who fulfilled ACR/EULAR 2013 SSc classification criteria and underwent to tests for nRAD (thyroiditis, hepatitis, cholangitis, atrophic gastritis, pancreatitis, diabetes, thrombocytopenia, anaemia, inflammatory bowel disease, celiac disease) were recruited. For each patient clinical, laboratory and instrumental data were recorded. The data were collected retrospectively.

**Results:** In the study population 32 (34,8%) SSc-pts had nRAD, with a higher rate of female (93%). Autoimmune thyroiditis was the most common nRAD (15%), followed by atrophic gastritis (14,1%) and autoimmune thrombocytopenia (6,5%). Primary biliary cholangitis was identified in one patient. The SSc-pts with nRAD, compared to those without, had a significant higher rate of dysphagia (78% vs 50%; p=0,009; odds ratio (OR): 3,5), diffuse cutaneous subset (d-SSc) (28% vs 8%; p=0,01); anti Scl70 positive (59% vs 30%, p=0,016; OR: 3,35), heart diastolic disfunction (HDD) (50% vs 22%, p=0,02) and late scleroderma pattern (39% vs 10%).

A mild trend to have a higher rate of hypovitaminosis D (81,3% vs 68,3%), arthralgia (47% vs 32%), GERD (66% vs 52%), ILD (36% vs 27%), anti CCp positive (12% vs 7%), electrocardiogram abnormalities (19% vs 10%) and PAH (50% vs 32%) was observed in pts-SSc with nRAD compared to those without.

Also considering the group of SSc-pts with anti-Scl-70+ the trend to a higher rate of ILD (63% vs 58%) and d-SSc (50% vs 16%) was observed in nRAD-pts than those without. Restricting the analysis to CENP-B+ pts group the PAH was more frequent in nRAD-pts than those without (46% vs 30%).

**Conclusions:** The presence of n-RAD in SSc-pts is frequently observed. Furthermore, the SSc-pts with nRAD seems to have more several clinical manifestations related to SSc. In these pts a greater grade of autoimmune dysregulation is supposed. Taking into account the higher risk to develop gastro-intestinal involvement, fibrosis, probably due to the wider positivity of anti-Scl-70, dysphagia and the more aggressive vascular impairment, with probably worse quality of life, a more strictly clinical and instrumental follow-up is suggested in n-RAD-pts. Therefore, extended screening for existing autoimmune diseases during the routine assessment of SSc-pts is recommended.

## P.163

## STUDY OF THE EPIDEMIOLOGICAL, CLINICAL AND ANALYTICAL CHARACTERISTICS IN PATIENTS WITH SYSTEMIC SCLEROSIS AND CANCER IN VALL D’HEBRON HOSPITAL

M. Roca^1^, A. Guillén^1^, E. Callejas^2^, D. Bernal^3^, J. Hernando^1^, P. Gubern^1^, I. Sansano^1^, J. Perurena^1^, M. Sanz^1^, J. Tabernero^1^, V. Fonollosa^1^, C.P. Simeón^1^

^1^*Vall D’Hebron Hospital, Barcelona, Spain*, ^2^*Parc Taulí Hospital, Sabadell, Spain*, ^3^*Fuenlabrada Hospital, Fuenlabrada, Spain*

**Introduction:** Systemic sclerosis (SSc) is associated with an increased risk of certain types of cancer, particularly lung, liver, hematological and non-melanoma skin. Despite this increasement, the relative risk of developing cancer is still low. In the literature, neoplasms have been described in 3-11% of patients with SSc. Our objective is to analyze the epidemiological, clinical and analytical characteristics previously described as possibly linked to the development of a cancer in patients with systemic sclerosis in the Vall d’Hebron Hospital cohort.

**Material and Methods:** We analyzed 615 patients in the Vall d’Hebron Hospital cohort of SSc. The inclusion criteria were age >18 years and the diagnosis of SSc limited, diffuse and SSc sine scleroderma with all available data. The different variables were analyzed by univariate statistical analysis with SPSS v21.

**Results:** We included 481 patients with a diagnosis from January 1985 to September 2019. 88.8% were women, with an average age of 53. 100 patients were diagnosed with neoplasia. The analyzed variables were divided in: general characteristics (sex, age >65y at diagnosis, mortality, smokers, ex-smokers, subtype (limited/diffuse/sine scleroderma) and capillaroscopy pattern (slow/active)), clinical manifestations (Raynaud’s phenomenon, skin ulcers, telangiectasias, calcinosis cutis, esophageal involvement, arthralgia, arthritis, pulmonary hypertension, interstitial lung disease, cardiac affection), immunology pattern (presence of Ab anti-Slc70 (ATA), ACA, RNA polymerase III, Pm/Scl) and treatment (cyclophosphamide, azathioprine, mycophenolate, methotrexate, antiplatelet, ACEIs, ARBs, CCBs, corticoids, prostaglandins).

In the data analysis, mortality presents a statistically significant difference in patients with scleroderma and cancer (p= 0.01). The use of ARBs is associated with the development of neoplasia in patients with SSc (p=0.02).

The types of cancer developed by our cohort patients has also been described. 113 malignancies have been described in 100 patients. The prevalence of the different types of cancer was: breast 22.12%, non-melanoma skin 15.93%, lung 15.93%, hematologic malignancies 14.16%, gynecological 7.08%, colon 4.42%, urothelial 4.42%, gastroesophageal 4.42%, thyroid 4.42%, head and neck 2.65%, melanoma 1.77% and others 2.65%.

**Conclusions:** In the analyzed cohort, mortality presents a statistically significant difference in patients with scleroderma and cancer. The use of ARBs is associated with the development of neoplasia in patients with SSc. In our cohort 100 patients with SSc have developed cancer. The most prevalent types of cancer were breast cancer, non-melanoma skin cancer and lung cancer.

**Figure:**
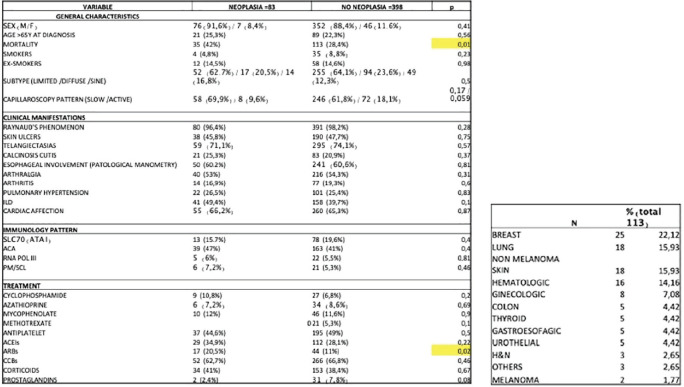


## P.164

## TRAJECTORIES OF PAIN IN SYSTEMIC SCLEROSIS

R.W. Robyn Wojeck^1^, L. Kwakkenbos^2^, M.-E. Carrier^3^, D.E. Bailey^1^, M. Knisely^1^, T.J. Somers^4^, S. Bartlett^5^, J. Pope^6^, W.R. Nielson^7^, K. Nielson^8^, B.D. Thombs^9^, S. Silva^1^ and the SPIN Investigators

^1^*Duke University, School of Nursing, Durham, USA*, ^2^*Radbound University, Nijmegan, The Netherlands*, ^3^*Lady Davis Institute of Medical Research, Jewish General Hospital, Montreal, Canada*, ^4^*Duke University, School of Medicine, Durham, USA*, ^5^*McGill University, Montreal, Canada*, ^6^*University of Western Ontario, London, Canada*, ^7^*St. Joseph’s Health Care, London, Canada*, ^8^*Scleroderma Society of Ontario, Ottawa, Canada*, ^9^*Lady Davis Institute of Medical Research, Jewish General Hospital and McGill University, Montreal, Canada*

**Introduction:** Systemic sclerosis (SSc; scleroderma) is a rare, chronic multisystem autoimmune disease associated with significant pain burden. While previous research has focused on the prevalence and severity of pain in SSc, few studies have addressed changes in pain across the disease trajectory. The purpose of this study was to compare trajectories of pain over three years in patients with SSc experiencing different levels of pain at the initial assessment.

**Material and Methods:** A prospective longitudinal cohort study was conducted using data collected at initial enrollment assessment (baseline) and at each three-month follow-up over 36-months for patients enrolled in the Scleroderma Patient-centered Intervention Network (SPIN) Cohort. Enrolling physicians provided basic medical data at baseline. Pain outcomes were pain interference and pain intensity, measured with the Patient-Reported Outcomes Measurement Information System-29v2. Baseline pain assessment scores for each outcome were collapsed into three levels: (1) low/mild, (2) moderate, or (3) high. Covariates included baseline patient demographic (i.e., age, gender, race) and clinical (i.e., diffuse/limited subset of SSc, time since diagnosis, rehabilitation in past three months, presence of finger ulcers, an overlapping autoimmune disease) characteristics. The analysis sample was 427 adults with SSc who met eligibility criteria for the SPIN Cohort, and included patients who completed baseline and a month-36 assessment. Non-directional statistical tests were performed with significance set at 0.05. Hierarchical mixed-effects models for longitudinal data, covarying for patient characteristics, were used to compare trajectories of pain interference and intensity across 36-months for patients with the three pain levels (groups) at baseline.

**Results:** The groups differed significantly in their trajectories of pain interference and intensity (both group-by-time interaction: p<0.001). The low/mild group experienced worsening of pain interference and intensity, while the moderate and high groups showed improvement in both pain outcomes. Participation in rehabilitation and the presence of finger ulcers were significantly associated with less pain interference, but greater pain intensity during the 36-month period. Shorter time since diagnosis was significantly associated with less pain interference, while the presence of an overlapping autoimmune disease was significantly related to greater pain intensity during the 36-month period. Additionally, African Americans/Blacks reported significantly greater pain intensity.

**Conclusions:** The findings suggest that patients with initial low/mild pain levels experience worsening pain over the disease trajectory, whereas patients with initial moderate and high pain levels experience improvements in pain. Targeted self-management interventions could be used to improve pain in SSc patients experiencing variable pain levels.

## P.165

## LOWER MORTALITY RATE BUT COMPARABLE CLINICAL CHARACTERISTICS OF SYSTEMIC SCLEROSIS IN CRETE-GREECE AS COMPARED TO OTHER EUROPEAN COUNTRIES

A. Repa, S. Pitsigavdaki, A. Molla Ismail Sali, N. Kougkas, M. Terixaki, N. Avgoustidis, P. Sidiropoulos, G. Bertsias

*Department of Rheumatology, University of Crete, Iraklion, Greece*

**Introduction:** Systemic Sclerosis (SSc) is a rare, autoimmune disease characterized by microvascular damage and fibrosis of multiple organs. SSc is associated with significantly increased morbidity and mortality as compared with the general population. Our aim was to describe the clinical characteristics and examine mortality, main causes of death and prognostic factors in a single-center South European cohort of SSc patients.

**Material and Methods:** We reviewed the demographics, clinical features, autoantibodies, capillaroscopy findings and the causes of death from the SSc cohort followed at the Rheumatology Department, University Hospital of Heraklion (Crete, Greece). Univariate and multivariable-adjusted analyses were performed to identify predictors of adverse outcomes.

**Results:** 102 patients (88% women, mean age at diagnosis 49.3 years) were included. Diffuse SSc (dSSc) was present in 32.3%, while an overlap of SSc with another connective tissue disease was found in 19.6%. Frequencies of anti-Scl70 and anti-centromere antibodies were 65.7% and 20%, respectively. Joint involvement and telangiectasia were the most frequent manifestations (76.5% and 74.5% respectively) followed by lung involvement [Interstitial lung disease (ILD): 62.7%, pulmonary arterial hypertension (PAH): 12.0%], whereas only a single patient developed renal crisis. A total 19 deaths occurred, corresponding to crude (all-cause) mortality rate of 2.2 per 100 person-years. The main cause of death was sepsis (21.0%) followed by cardiac arrest (15.7%). Of the SSc-related deaths (20.6%), 40% were attributed to PAH and 20% to renal crisis. Mean (± standard deviation) disease duration until death was 4.6 (±3.2) years in cases of SSc-related death, as compared to 17.5 (±12.7) years in deaths not related to SSc. In multivariable logistic regression, presence of ILD (Odds Ratio [OR]: 18.58, p=0.009) and tendon friction (OR: 8.19, p=0.046) were positively associated, while anti-Scl70 antibodies were negatively associated (OR: 0.13, p=0.003) to all-cause mortality.

**Conclusions:** In a contemporary SSc cohort in Crete, mortality is lower than previously reported for other European countries albeit with comparable clinical characteristics and causes of death. ILD and tendon friction are poor prognostic risk factors. Mortality due to SSc occurs early in the disease course, emphasizing the importance of early disease recognition and treatment.

## P.166

## ASSESSMENT OF THE ACCURACY OF USING ICD-10 CODES TO IDENTIFY SYSTEMIC SCLEROSIS

S. De Almeida Chaves, H. Derumeaux, P. Do Minh, M. Lapeyre-Mestre, G. Moulis, G. Pugnet

*CHU Toulouse, Toulouse, France*

**Introduction:** With the increased use of data from electronic medical records for research, it is important to validate International Classification of Diseases, tenth Revision (ICD-10) codes for their respective diagnoses. The aim of this was to assess the accuracy of using ICD-10 codes to identify systemic sclerosis (SSc) in the French hospital database.

**Material and Methods:** Retrospective database analysis. The setting of the study’s in-patient database was the Toulouse University Hospital, a tertiary referral center (2,880 beds) that serves approximately 2.9 million inhabitants. Participants were patients with ICD-10 discharge diagnosis codes of SSc seen at Toulouse University Hospital between January 1, 2010, and December 31, 2017.

The main outcome was the positive predictive value (PPV) of discharge diagnosis codes for identifying SSc. The PPVs were calculated by determining the ratio of the confirmed cases found by medical record review to the total number of cases identified by ICD-10 code.

**Results:** Of the 2,766 hospital stays, 216 patients were identified by a SSc discharge diagnosis code. Two hundred were confirmed as SSc after medical record review. The overall PPV was 93% (95% CI, 88%-95%). The PPV for limited cutaneous SSc was 95% (95% CI, 85%-98%).

During the same period, 172 SSc patients were included in the Toulouse University Hospital SSc cohort following a hospitalization. The sensitivity of the SSc discharge diagnosis codes was 91% (95% CI, 85%-94%).

**Conclusions:** Our results suggest that using ICD-10 codes alone to capture SSc is reliable in The French hospital database.

**Figure:**
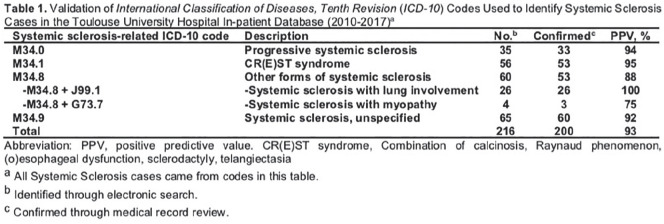


## P.167

## TREATMENT MODALITIES AND DRUG SURVIVAL IN A SYSTEMIC SCLEROSIS REAL –LIFE PATIENT COHORT

S. Panopoulos^1^, K. Chatzidionysiou^1^, M. Tektonidou^1^, A. Drosos^2^, N. Liossis^3^, T. Dimitroulas^4^, L. Sakkas^5^, D. Boumpas^1^, P. Voulgari^2^, D. Daoussis^3^, K. Thomas^1^, G. Georgiopoulos^1^, G. Vosvotekas^4^, G. Bertsias^6^, P. Sidiripoulos^6^, D. Vassilopoulos^1^, P. Sfikakis^1^

^1^*Joint Rheumatology Program, Medical school, National and Kapodistrian University of Athens, Athens, Greece*, ^2^*Medical School, University of Ioannina, Ioannina, Greece*, ^3^*Faculty of Medicine, University of Patras, Patra, Greece*, ^4^*School of Medicine, Aristotle University of Thessaloniki, Thessaloniki, Greece*, ^5^*Faculty of Medicine, University of Thessaly, Larissa, Larissa, Greece*, ^6^*Medical School, University of Heraklion, Heraklion, Greece*

**Introduction:** Mainstays of treatment in Systemic sclerosis (SSc) include immunosuppressive/antiproliferative and vasoactive drugs. Recently, the EUSTAR group reported a decrease in standardised mortality rate in SSc over time due to a decrease in mortality unrelated to SSc, while the rate of deaths related to SSc has increased, thus, questioning the efficacy of current treatments and denoting the need for more effective agents. Our aim is to analyze current treatment modalities and drug survival in a real-life patient cohort.

**Material and Methods:** Medical records of alive consecutive SSc patients from all 6 academic rheumatology departments in Greece were analyzed and immunosuppressive/antiproliferative (methotrexate, mycophenolic mofetyl, cyclophosphamide, azathioprine, rituximab, tocilizumab) and vasoactive (endothelin receptor antagonists (ERAs), sildenafil, iloprost, calcium channel blockers (CCB)) drugs administered anytime during the disease course were recorded. Reasons for treatment cessation were classified either as side effects, or lack of response, or disease stabilisation, or miscellaneous. Kaplan Meier analysis was performed to examine drug survival. Curves were compared with the Log-Rank test.

**Results:** A total of 497 patients (243 diffuse, mean disease duration 11.8±8.4 years) were included. Methotrexate was the most frequent immunosuppressive/antiproliferative drug administered in 53% of patients, followed by cyclophosphamide in 27% and mycophenolate in 12% of patients. Regarding vasoactive agents, CCB were administered in 67%, ERAs in 39%, sildenafil in 8% and iloprost in 7% of patients respectively. Of note, 23% of patients with pulmonary fibrosis had never received immunosuppressive/antiproliferative treatment, 34% of patients with digital ulcers had never received vasoactive therapy and 20% of all SSc patients were never administered either immunosuppressive/antiproliferative or vasoactive treatment. The 12- and 24-month survival of methotrexate, azathioprine, mycophenolate, and cyclophosphamide was 83/74%, 73/66%, 72/60%, 56/40%, respectively. In contrast vasoactive agents had high retention rates, as the 12- and 24-month survival for ERAs, sildenafil and CCB were 88/86%, 82/80%, and 97/91% respectively.

**Conclusions:** Risk stratification and evidence-based treatment are mandatory in order to avoid under- or over treatment of SSc patients. The low retention rates of the currently used immunosuppressive/antiproliferative drugs reflect the need to use more potent and targeted disease modifying agents for SSc. Conversely, vasculopathy seems to be managed more rigorously after the introduction of advanced vasoactive regimens.

## P.168

## PREVALENCE, SEVERITY AND CLINICAL CORRELATES OF SYMPTOMS OF AUTONOMIC DYSFUNCTION IN PATIENTS WITH SYSTEMIC SCLEROSIS

P. Ostojic, M. Atanaskovic Popovic, M. Vujovic, M. Perovic

*Institute of Rheumatology, School of Medicine, University of Belgrade, Belgrade, Serbia*

**Introduction:** This study aims to assess prevalence, severity and clinical correlates of symptoms of autonomic dysfunction in patients with SSc

**Material and Methods:** Fifty five consecutive SSc patients were included. 37 (67.3%) patients had limited (lcSSc), whilst 18 (32.7%) patients had diffuse cutaneous SSc (dcSSc). Anticentromere antibodies (ACA) were positive in 31 (56.4%) of patients, whilst 20 (26.4%) patients had anti-topoisomerase I antibodies (ATA). All patients completed the Composite Autonomic Symptom Score (COMPASS-31) questionnaire, which consists of 31 items, quantifying six autonomic domains: orthostatic intolerance (OI), vasomotor dysfunction (VD), secretomotor (SD), gastrointestinal (GD), bladder (BD) and pupillomotor dysfunction (PD). The total score ranges from 0 to 100, whilst scores for particular domains range as follows: 0-40 for OI, 0-5 for VD, 0-15 for SD, 0-25 for GD, 0-10 BD, and 0-5 for PD. Higher values representing more severe symptoms. Differences in total COMPASS-31 score and domain-specific scores were assessed with respect to disease subtype, antibody status, capillaroscopic findings, lung diffusing capacity and joint involvement. Moreover, we assessed the correlation between COMPASS-31 scores, disease status (asssessed using the Scleroderma Assessment Questionnaire – SAQ) and severity of gastrointestinal symptoms (assessed using the UCLA SCTC GIT 2.0 questionnaire)

**Results:** Percentage of SSc patients with a score >0 in particular domains of the COMPASS-31 were as follows: OI – 32/55 (58.2%), VD – 49/55 (89.1%), SD – 36/55 (65.5%), GD – 40/55 (72.7%), BD - 26/55 (47.3%), PD – 30/55 (54.5%). The COMAPSS-31 score did not correlate with age or disease duration. There was no relationship between the COMPASS-31 total or subdomain scores and SSc subtype or autoantibody status. Similar mean values for COMPASS-31 scores were found among patients with different capillaroscopic patterns. Patients with DLCO < 80% had significantly higher mean values of GD, BD and PD scores, compared to patients with normal DLCO (4.42 vs 2.75, 1.51 vs 0.38, 1.93 vs 1.09, respectively). Moreover, the total COMAPSS-31 score was significantly higher in patients with decreased DLCO (16.24 vs 11.34, p=0.008). Patients with joint involvement had higher COMPASS-31 score than patients without (17.74 vs 9.85, p=0.012). We have found a statistically significant (p<0.001) positive correlation between the COMPASS-31 score and the Index of disease status (IDS), as well as the total UCLA SCTC GIT score (rho=+0.45, rho=+0.51, respectively).

**Conclusions:** Symptoms of dysautonomia are common in SSc patients. Patients with more severe disease, especially decreased lung diffusing capacity, joint pain, and severe gastrointestinal involvement, report more symptoms of autonomic dysfunction.

## P.169

## TOLERABILITY AND SAFETY OF ACETYLSALICYLIC ACID IN PATIENTS WITH SYSTEMIC SCLEROSIS

L. Verardi^1^, E. De Lorenzis^1^, G. Natalello^1^, G.B. Canestrari^2^, L. Gigante^1^, E. Gremese^1,2^, S.L. Bosello^2^

^1^*Institute of Rheumatology, Catholic University of the Sacred Heart, Rome, Italy*, ^2^*Department of Rheumatology, Fondazione Policlinico Universitario A. Gemelli - IRCCS, Rome, Italy*

**Introduction:** Systemic Sclerosis (SSc) is characterized by an increased incidence of macro- and microvascular complications. Current evidences on efficacy, safety and tolerability of acetylsalicylic acid (ASA) in SSc patients are limited, and the indication to this treatment is based on the experience of each single centre or specialist. We evaluated the incidence of adverse events and consequent discontinuation of ASA in a group of patients affected by early SSc (disease duration less than 3 years at the moment of the enrollment).

**Material and Methods:** One hundred-sixty patients affected by SSc in therapy with ASA 100 mg/day were evaluated for a mean follow-up period of 7.2 years (range: 1.2-10.1) taking note of all adverse events attributable to the antiplatelet therapy. All demographic and clinical characteristics were recorded.

**Results:** Patients had a mean age of 54.9±13.5 years; 91.9% were female; 47.8% were smokers, 43.6% had a BMI> 25. The patients showed a diffuse cutaneous involvement in 35.2% of cases, ulcers history in 45.9%, positivity to anti-centromere and anti-Scl70 antibodies respectively in 34.6% and in 46.5% of cases. The prevalence of arterial hypertension, ischemic heart disease, peripheral arteriopathy, diabetes, stroke or transient ischemic attack history was respectively 30.6%, 12.5%, 5.7%, 5.7% and 4.5%. All patients took concomitant therapy with gastric protectors. During the observational period 2 patients displayed epistaxis (1.9 per 1000 person years) and 62 showed onset or worsening of heartburn (59.3 per 1000 person yeas). Fifteen of them (14.3 per 1000 person years) presented mucosal erosions on esophagogastroduodenoscopy (EGDS).

Ten patients (9.6 per 1000 person years) needed to discontinue ASA (2 for epistaxis, 8 for gastrointestinal intolerance with or without erosions and/or bleeding assessed on EGDS). Six patients died for causes not correlated to ASA. Cox regression showed that female sex (HR=3.2 IC 95% 1.1-9.4; p=0,037), overweight (HR=2.9 IC 95% 1.6-5.2; p<0,001) and absence of digital ulcers history (HR 0.4 IC 95% 0.2-0,7; p=0.002) were associated with gastrointestinal intolerance.

**Conclusions:** In our cohort of SSc patients, ASA resulted safe and well tolerated in most cases, despite the high prevalence of gastroesophageal abnormalities due to disease.

Although this comforting results, taking in account the lack of controlled-randomized trials about efficacy and safety, the choice to start antiplatelet therapy with ASA should be mandatorily preceded by a careful evaluation of risks and benefits. Furthermore, an attentive monitoring for possible adverse effects is needed during ASA treatment.

## P.170

## PATIENT-LEVEL EVALUATION OF COMPONENTS OF THE ACR COMBINED RESPONSE INDEX IN SYSTEMIC SCLEROSIS (CRISS) USING PATIENT-REPORTED ANCHORS

V. Nagaraja^1^, J. Powers^2^, C. Lin^3^, V. Berrocal^1^, D. Khanna^1^

^1^*University of Michigan, Ann Arbor, USA*, ^2^*University of Maryland School of Medicine, Baltimore, USA*, ^3^*Genentech Inc, San Francisco, USA*

**Introduction:** Treatment benefit is demonstrated by evidence that interventions have positive impacts on how patients feel, function, and/or survive (FDA Guidance 21CFR314.510). The ACR CRISS, a composite endpoint for trials in systemic sclerosis (SSc), uses a weighted score that combines outcome assessments that directly measure how patients feel and function [HAQ-DI and patient (PGA) global assessment] with outcome assessments that are indirect measures of patient symptoms/function [FVC, modified Rodnan skin score (mRSS), and physician global assessments (MDGA)]. Understanding the relationship and magnitude of effects on these indirect assessments would provide confidence that each component of CRISS would reliably predict an effect on direct measures of patient benefit. Our objective was to provide data to support evaluation of CRISS using patient-reported outcome (PRO) anchors, i.e. HAQ-DI and PGA.

**Material and Methods:** We evaluated 2 cohorts: an early diffuse cutaneous SSc (dcSSc) cohort that was used for development of ACR CRISS and a phase 2 trial of TCZ vs. placebo in dcSSc [faSScinate trial]. Using the early dcSSc cohort, we assessed the effect size (ES) at the patient-level for non-PRO variables (mRSS, MDGA, and FVC%) in those we defined as “responders” who met minimal clinically important differences (MCID) estimates for HAQ-DI (defined as improvement of > or = 0.22) and PGA improvement of > or = 1.0 (range 0-10). We interpreted the ES using the Cohen’s criteria: < 0.20= negligible, 0.20-0.49 = small, 0.50-0.79 = medium, > 0.80 = large (3). We then assessed whether ES in patients who met the responder criteria in HAQ-DI and PGA in the faSScinate trial was associated with larger improvements in the ACR CRISS scores at week 24 and 48.

**Results:** In the dcSSc cohort, the ES were generally of greater magnitude for patients for responders vs. non-responders (Table 1), except for HAQ-DI and FVC% when using PGA as an anchor. In the faSScinate trial, statistically significant improvements in the median ACR CRISS scores were seen in those who attained MCID vs. patients who did not (Table 1).

**Conclusions:** In a dcSSc cohort, patients who achieved MCID in HAQ-DI and PGA are associated with larger magnitude of improvement in ACR CRISS non-PRO variables. Limitations of the analysis include: 1. HAQ-DI and PGA are part of the ACR CRISS score, and 2. Assessing the relationships in an observational cohort. Ongoing trials should confirm the relationships between non-PRO variables (mRSS, MDGA, and FVC%) vs. PRO anchors.

**Figure:**
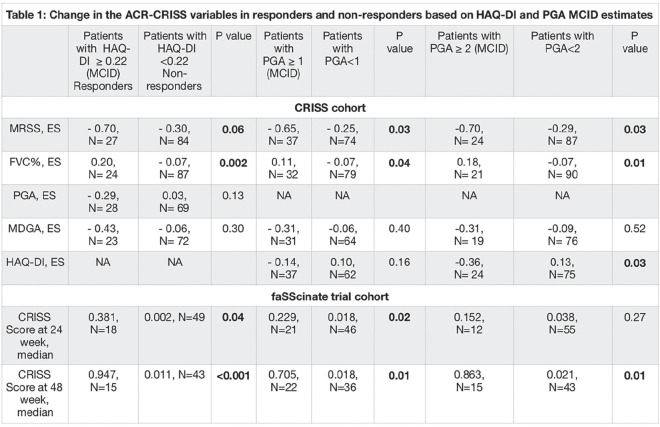


## P.171

## A LATE ONSET OF SYSTEMIC SCLEROSIS IS ASSOCIATED WITH A MORE RAPIDLY PROGRESSING CLINICAL PHENOTYPE - DATA OF THE GERMAN NETWORK FOR SYSTEMIC SCLEROSIS

P. Moinzadeh^1^, S. Hamacher^2^, E. Siegert^3^, U. Mueller-Ladner^4^, G. Riemekasten^5^, C. Guenther^6^, I. Koetter^7^, J. Henes^8^, N. Blank^9^, G. Zeidler^10^, C. Pfeiffer^11^, A. Juche^12^, I. Jandova^13^, J. Ehrchen^14^, M. Schmalzing^15^, L. Susok^16^, T. Schmeiser^17^, C. Sunderkoetter^18^, J.H. Distler^19^, N. Hunzelmann^1^

^1^*University Hospital of Cologne - Department of Dermatology and Venerology, Cologne, Germany*, ^2^*University of Cologne - Institute of Medical Statistics and Computational Biology, Cologne, Germany*, ^3^*Charite University Hospital - Department of Rheumatology, Berlin, Germany*, ^4^*Justus Liebig University of Giessen - Kerckhoff Clinic Bad Nauheim - Department of Rheumatology, Bad Nauheim, Germany*, ^5^*University Medical Center UKSH - Department of Rheumatology, Luebeck, Germany*, ^6^*University Hospital Carl Gustav Carus Dresden - Department of Dermatology, Dresden, Germany*, ^7^*Asklepios Clinic Altona - Department for Internal Medicine, Rheumatology, Immunology and Nephrology, Hamburg, Germany*, ^8^*University Hospital Tuebingen - Department of Internal Medicine II, Tuebingen, Germany*, ^9^*University Hospital Heidelberg - Department of Rheumatology, Heidelberg, Germany*, ^10^*Johanniter Hospital Treuenbrietzen - Department of Rheumatology, Treuenbrietzen, Germany*, ^11^*University Hospital Ulm - Department of Dermatology, Ulm, Germany*, ^12^*Immanuel Hospital Berlin-Buch - Department of Rheumatology, Berlin, Germany*, ^13^*University Hospital Freiburg - Department of Rheumatology, Freiburg, Germany*, ^14^*University Hospital Muenster - Department of Dermatology, Muenster, Germany*, ^15^*University Hospital Wuerzburg - Department of Rheumatology, Wuerzburg, Germany*, ^16^*Ruhr-University Bochum - Department of Dermatology, Bochum, Germany*, ^17^*St. Josef Hospital Wuppertal - Department of Dermatology, Wuppertal, Germany*, ^18^*University Hospital Halle - Department of Dermatology, Halle, Germany*, ^19^*University Hospital Erlangen - Department of Rheumatology, Erlangen, Germany*

**Introduction:** Systemic sclerosis (SSc) is a heterogeneous multisystem connective tissue disease. The majority of patients with SSc develop initial clinical symptoms in early or late adulthood. It is not yet known whether the age of onset of the disease has an influence on the development of a distinct clinical phenotype.

**Objectives:** Investigating the relationship between age at disease onset and the development of clinical characteristics using data of the German Network for Systemic Scleroderma.

**Material and Methods:** Clinical data of the patient registry, currently including 3281 data of patients, were evaluated. Three age ranges at disease onset (<40years, 40-60years, and >60years) were correlated with clinical characteristics.

**Results:** Among all SSc patients, 24.5% of patients developed non-raynaud symptoms initially at the age <40 years, 53.0% developed SSc between 40-60 years, and 22.5% were older than 60 years of age. In particular, SSc patients with disease onset > 60 years developed significantly (p<0.0001) more often the lcSSc subtype (65.0%) as well as anti-centromere antibodies (44.1%), had a significantly lower modified Rodnan Skin Score (mRSS) (mean mRSS, 9.4±8.4; p=0.023). However, they had more often pulmonary hypertension (PH) (19.5%) but less often digital ulcerations (20%). Elderly patients were significantly more often female within this cohort (p=0.008).

Remarkably, using the follow-up data over 15 years the course of the disease is more rapidly progressing in the older cohort (> 60 years) compared to the younger cohort, except for gastrointestinal and musculoskeletal involvement. No significant difference was found for the use of corticosteroids; however, significant less patients older than 60 years at disease onset underwent immunosuppressive treatment.

**Conclusions:** In this registry, approximately 25% of patients developed SSc at an age above 60 years with an increased frequency of the lcSSc subset. In this age group onset of internal organ involvement was accelerated across all subsets. These findings have an important impact on the urgency of adapted follow-up examinations and treatment.

## P.172

## DECREASED ORAL APERTURE IS A POOR PROGNOSTIC FACTOR IN SYSTEMIC SCLEROSIS

T. Minier^1^, V. Lóránd^2^, Z. Bálint^1^, D. Komjáti^1^, B. Németh^1^, K. Filipánits^3^, A. Kovács^1^, G. Nagy^1^, C. Varjú^1^, L. Czirják^1^, G. Kumánovics^1^

^1^*Department of Rheumatology and Immunology, University of Pécs, Pécs, Hungary*, ^2^*Department of Rheumatology, Polyclinic of the Hospitaller Brothers of St John of God, Budapest, Hungary*, ^3^*Medical School, University of Pécs, Pécs, Hungary*

**Introduction:** Decreased mouth opening (microstomia) is a characteristic clinical sign in systemic sclerosis (SSc) which can exert a remarkable impact on quality of life, because it can lead to poor oral hygiene, inability of eating, and may also affect self-esteem. Our objectives were to compare the available measurements for the assessment of maximal oral aperture (e.g. interlabial distance, interincisial distance) and to look for more sensitive measurements. Furthermore we aimed to investigate the prognostic value of microstomia on survival of SSc patients.

**Material and Methods:** One hundred and thirty-one consecutive SSc patients from a single tertiary care center were enrolled between 2005 and 2006. Forty-one patients had diffuse cutaneous SSc, and 90 patients had limited cutaneous SSc. Mean age at enrolment was 55.9±11.6 years (±SD) with mean disease duration of 8.1 ±7.2 years. Data on laboratory parameters, physical examination, clinical investigations, and patient reported outcomes were collected. Survival data were calculated based on the available data on patients at 31st December 2018. Median follow up time was 11 years (IQR: 5; 12 years). 46 patients died during the follow-up period, 28 due to SSc related causes. 18 patients have moved to other centres, and 2 patients had been lost to follow-up. Sixty-three healthy control individuals with mean age of 53.6 ±17.4 years (±SD) were also enrolled for comparison.

Vertical interlabial (VILD) and interincisial distance (VIID) at maximally opened mouth, horizontal width at maximally opened mouth (HO) and at closed mouth (HC) were measured. The oral area (OA) and oral circumference (OC) parameters were also generated from the measured variables, considering that the mouth by good approximation resembles the shape of an ellipse.

**Results:** Patients with decreased VILD and OC at baseline investigation had worse SSc-related mortality (Log Rank chi-square: 5.405, p=0.02, and 4.455, p=0.035, respectively). No correlations with the modified Rodnan skin score, age at enrolment and parameters of oral aperture were identified. Markers of functional disability (Health Assesment Questionnaire, Disabilities of the Arm, Shoulder and Hand, Hand Anatomic Index) showed weak to moderate correlation (rho: -.194 - -.475) with VIID, VILD, OC and OA. Change in OC and OA correlated weakly with change in HAQ at one year follow-up (rho: -.203, p= 0.025, and rho: -.198, p=0.029, respectively).

**Conclusions:** Decreased oral aperture seems to be related to functional impairment in systemic sclerosis. Decreased vertical interlabial distance and oral circumference may predict a worse survival in consecutive SSc patients.

## P.173

## THE RELATIONSHIP OF PATIENT SOCIODEMOGRAPHIC AND SCLERODERMA DISEASE CHARACTERISTICS WITH SOCIAL INTERACTION ANXIETY: A SCLERODERMA PATIENT-CENTERED INTERVENTION NETWORK COHORT STUDY

A. Meier^1^, S. Gholizadeh^1,2^, B. Ataseven^1,3^, S. Roesch^1^, M.-E. Carrier^2^, L. Kwakkenbos^4^, B. Thombs^2,5-10^, V. Malcarne^1^, K. Gottesman^11^, Spin Investigators

^1^*San Diego State University, San Diego, USA*, ^2^*Lady Davis Institute for Medical Research, Jewish General Hospital, Montreal, Quebec, Canada*, ^3^*Istanbul Kultur University, Istanbul, Turkey*, ^4^*Behavioural Science Institute, Clinical Psychology, Radboud University, Nijmegen, The Netherlands*, ^5^*Department of Psychiatry, McGill University, Montreal, Quebec, Canada*, ^6^*Department of Epidemiology, Biostatistics, and Occupational Health, McGill University, Montreal, Quebec, Canada*, ^7^*Department of Medicine, McGill University, Montreal, Quebec, Canada*, ^8^*Department of Psychology, McGill University, Montreal, Quebec, Canada*, ^9^*Department of Educational and Counseling Psychology, McGill University, Montreal, Quebec, Canada*, ^10^*Biomedical Ethics Unit, McGill University, Montreal, Quebec, Canada*, ^11^*Scleroderma Foundation, USA*

**Introduction:** The implications of systemic sclerosis (SSc, scleroderma) for health-related quality of life can be extensive and include reduced physical and psychological functioning and poorer overall well-being. Social anxiety has been identified as a quality of life issue that may be particularly relevant for diseases that cause disfigurement. This study evaluated how sociodemographic variables and disease characteristics of people with scleroderma relate to levels of social interaction anxiety, which is a form of anxiety that negatively impacts the process of interacting with others, and whether these associations are moderated by gender.

**Material and Methods:** Cross-sectional baseline data were analyzed from 1,554 patients who participated in the Scleroderma Patient-centered Intervention Network (SPIN) international cohort and who completed the Social Interaction Anxiety Scale (SIAS). Sociodemographic and disease variables were analyzed as predictors of SIAS scores, with gender considered as a moderator, using hierarchical linear and logistic regression analyses.

**Results:** There was no significant difference in total SIAS scores between men and women. Age and education were significantly negatively related to SIAS total scores consistently within two linear regression and two logistic regression analyses. The number of appearance-related symptoms (B = 0.217) and the number of appearance-unrelated symptoms (B = 0.266) were both related to SIAS scores. Patients with gastrointestinal symptoms, including presence of diarrhea, bloating and/or constipation (B = 0.670) and/or presence of early satiety and/or vomiting due to SSc (B = 0.653), showed significantly higher levels of social interaction anxiety than patients without these symptoms.

**Conclusions:** On average, patients with SSc had low levels of social interaction anxiety relative to clinically anxious samples, although some patients reported high levels of social interaction anxiety. Both appearance-related and appearance-unrelated symptoms were significantly positively related to social anxiety, with more symptoms of both types associated with higher levels of social anxiety. Gastrointestinal problems seemed of notable importance, and patients who experienced diarrhea, bloating, constipation, early satiety, or vomiting had higher levels of social interaction anxiety than those with other symptoms that are not related to appearance. These types of gastrointestinal distress may make patients more prone to social problems. The identification of social interaction anxiety within some patients with SSc, combined with the understanding of what aspects of the disease may contribute to this anxiety, will allow researchers to identify patients who may be risk for scleroderma-related social difficulties and at need for intervention.

## P.174

## THE IMPORTANCE OF A SYSTEMIC SCLEROSIS CLINIC IN A TERTIARY REFERRAL CENTER

P. Martins^1^, E. Dourado^1^, J. Fonseca^1,2^, I. Cordeiro^1,2^, C. Resende^1^

^1^*Serviço de Reumatologia e Doenças Ósseas Metabólicas, Hospital de Santa Maria, Centro Hospitalar Universitário Lisboa No, Lisboa, Portugal*, ^2^*Unidade de Investigação em Reumatologia, Instituto de Medicina Molecular, Faculdade de Medicina, Universidade de Lisboa, Lisbooa, Portugal*

**Introduction:** Systemic sclerosis (SSc) is a rare systemic rheumatic disease (SRD) characterized by small vessel inflammation and fibrosis of not only skin but also internal organs. Pulmonary and cardiac involvement contribute to both morbidity and mortality associated with the disease. A multidisciplinary approach with strict monitoring is therefore key to attain clinical success.

Objectives: To describe the organization and patient pathways of our SSc outpatient clinic.

**Material and Methods:** Observational study using data extracted from Reuma.pt/SSc (the Portuguese national registry of SSc patients). Data extracted included demographic variables, clinical manifestations and immunological expression. The disease was classified according to the 2013 ACR/EULAR criteria.

Experience: Our SSc clinic is managed by two dedicated Rheumatologists and up to two Rheumatology residents on a weekly basis, but it is a dynamic multidisciplinary clinic where various medical specialties collaborate closely. There are two associated subspecialty clinics (pulmonary hypertension and pulmonary fibrosis) where the Rheumatologists engage with pneumologists and cardiologists, allowing greater collaboration in the management of these patients. Patients’ data is systematically registered in Reuma.pt/SSc as a part of the routine activity of this clinic, contributing to real-world data on SSc.

**Results:** A total of 220 patients were registered between July 2011 and June 2019. 196 (89.1%) were female, with a mean age of 58.9±14 years and a mean disease duration of 14.6±9 years. Nineteen seven patients (44.1%) had limited cutaneous SSc, 52 (23.6%) had diffuse cutaneous SSc, 35 (15.9%) had overlap SSc 24 (10.9%) had preclinical SSc and 12 (5.4%) had SSc sine scleroderma. Raynaud phenomenon was present in 92% of the SSc patients and 40% had a history of digital ulcers. Gastrointestinal manifestations included esophageal dismotility in 39.5% of patients, gastric disease in 24.4% and intestinal involvement in 15.5%. Pulmonary involvement was found in 47.6% of SSc patients, heart disease in 43.6% and kidney involvement in only two patients. Antinuclear antibodies were positive in 92.2% of SSc patients, anti-centromere in 44.1%, anti-topoisomerase I antibodies in 39.1%, anti-U1RNP in 4.5% and only three patients had anti-PM-Scl and one had anti-RNA polymerase III. 31 patients with SSc were lost to follow-up and 32 died. 18 patients are currently followed in the pulmonary hypertension clinic and seven in the pulmonary fibrosis clinic.

**Conclusions:** The implementation of a standardized approach with regular multidisciplinary work has proven very helpful in evaluating patients with SSc. The continuous registry of patients in Reuma.pt/SSc has been essential for patient care, research and healthcare planning.

## P.175

## RELATIONSHIP BETWEEN DISEASE ACTIVITY/SEVERITY/DAMAGE AND HEALTH-RELATED QUALITY OF LIFE IN PATIENTS WITH LOCALIZED SCLERODERMA: A CROSS-SECTIONAL STUDY

A. Lis-Swiety, A. Skrzypek-Salamon, I. Ranosz-Janicka, L. Brzezinska-Wcislo

*Department of Dermatology, Medical University of Silesia, Katowice, Poland*

**Introduction:** Localized scleroderma (LoS) is a chronic disorder of the skin and subcutaneous tissues with variable clinical presentation. Extensive clinical heterogeneity, presence of extracutaneos features, psychoemotional impact of disease and lack of available specific laboratory test hinder appropriate assessment of LoS. Few clinical scoring methods were proposed for the evaluation of the disease, but most of them was not validated yet. LoS Cutaneous Assessment Tool (LoSCAT) that differentiates between disease activity and damage is the most popular scoring method. The Visual Analogue Scales (VASs) have been used to assess the Physician (Phys-) and Patient (Pt-) Global Assessment (GA) of the disease activity/severity (-A/-S) and damage (-D) in juvenile LoS. For now VASs are the only tools that take into account not only cutaneous but also extracutaneous variables of LoS.

The aim of a study was to compare VASs and LoSCAT measures and related quality of life in adult patients with LoS.

**Material and Methods:** Features of the overall LoS activity and damage were identified and the instructions with detailed rules for VASes as well as for LoSCAT were prepared. Physician-reported measures (LoSCAT, PhysGA-A/-D) and patient-derived GA-S/-D were obtained twice with 48 hours interval in 40 adult patients. Health-related quality of life was measured with Skindex-29 and SF-36.

**Results:** The inter- and intra-rater reliabilities of the VASes and LoSCAT were satisfactory with the LoSCAT predominance. Modified LoS Skin Severity Index (mLoSSI), PhysGA-A and PtGA-S correlated with each other as well as LoS Skin Damage Index (LoSDI) and PhysGA-D. Many significant associations was found between VASes and HRQoL: PhysGA-A correlated with the SF-36 PCS and PtGA-D with all Skindex-29 subtypes and also with the SF-36:MCS. The mLoSSI correlated only with MCS domain of the SF-36.

**Conclusions:** Strong correlation between VASes and HRQoL opposite to LoSCAT suggest that this tool provides comprehensive evaluation of the disease and should not be omitted in the assessment of LoS.

## P.176

## DEVELOPMENT AND EVALUATION OF A PATIENT DECISION AID FOR AUTOLOGOUS HEMATOPOIETIC STEM CELL TRANSPLANTATION IN SYSTEMIC SCLEROSIS

L. Kwakkenbos^1^, M. Hudson^2,3^, L. Schraven^4^, J. Thonen-Velthuizen^5^, B. Thombs^2,3^

^1^*Radboud University, Nijmegen, The Netherlands*, ^2^*Jewish General Hospital, Montreal, Canada*, ^3^*McGill University, Montreal, Canada*, ^4^*Federation of European Scleroderma Associations, Saint Maur, Belgium*, ^5^*Scleroderma Framed, Nijmegen, The Netherlands*

**Introduction:** Shared decision making is a fundamental component of evidence-based practice. Individuals living with systemic sclerosis (SSc) often need to make complex decisions about their treatment. This can occur when there is more than one option and neither is clearly better or when options have benefits and harms that people value differently. Patient decision aids are educational tools that can support people with SSc and their health care providers to engage in effective shared decision making by providing balanced information about treatment choices and helping patients construct, clarify and communicate what is important to them in making health care choices. There are currently no patient decision aids for any treatment decisions in SSc.

A complex treatment decision in SSc involves autologous hematopoietic stem cell transplantation (AHSCT). Recent evidence suggests that AHSCT reduces the risk of all-cause mortality and leads to significant improvements in skin involvement and lung function compared with pulse intravenous cyclophosphamide, and greater improvements in health-related quality of life. On the other hand, the risk of treatment-related mortality and other severe adverse events is higher in AHSCT. Thus, there is no single ‘best choice’ and different people may weigh these potential benefits and risks differently.

The objective of this project is to develop a decision aid for AHSCT in SSc and to collect feedback on the comprehensibility and perceived utility.

**Material and Methods:** A five-step approach will be taken to the development of the decision aid. First, a research team will be established consisting of clinicians, patients, researchers and experts in decision making (Phase 1). Phase 2 includes reviewing and synthesising the evidence on the possible harms and benefits of AHSCT versus other treatments. In phase 3, we will conduct focus group interviews to gather input on patients’ decision needs. This information will lead to the development of an initial decision aid in phase 4. Phase 5 consists of a pilot-test during which we will collect feedback from patients with SSc and clinicians to assess the comprehensibility and perceived utility of the decision aid. After revising the decision aid if necessary, we will broadly disseminate the tool to patients and healthcare providers.

**Results:** Results are forthcoming and will be presented at the World Scleroderma Congress.

**Conclusions:** A decision tool for AHSCT in SSc will become available to patients and clinicians caring for them around the globe. This will also provide a model for the development of decision aids for other SSc treatments.

## P.177

## REASONS FOR NOT PARTICIPATING IN SCLERODERMA PATIENT SUPPORT GROUPS: A COMPARISON OF RESULTS FROM THE NORTH AMERICAN AND EUROPEAN SCLERODERMA SUPPORT GROUP SURVEYS

L. Kwakkenbos^1^, A. Carboni-Jiménez^2^, M.-E. Carrier^2^, M. Pépin^2^, S. Peláez^2,3^, V. Malcarne^4,5^, G. El-Baalbaki^6^, B. Thombs^2,3,7-9^, Support Group Advisory^10^

^1^*Radboud University, Behavioural Science Institute, Clinical Psychology, Nijmegen, The Netherlands*, ^2^*Jewish General Hospital, Lady Davis Institute for Medical Research, Montreal, Canada*, ^3^*McGill University, Department of Educational and Counselling Psychology, Montreal, Canada*, ^4^*San Diego State University, Department of Psychology, San Diego, USA*, ^5^*San Diego State University/University of California, Joint Doctoral Program in Clinical Psychology, San Diego, USA*, ^6^*Université du Québec à Montréal, Department of Psychology, Montreal, Canada*, ^7^*McGill University, Department of Psychiatry, Montreal, Canada*, ^8^*McGill University, Department of Epidemiology, Biostatistics, and Occupational Health, Montreal, Canada*, ^9^*McGill University, Department of Psychology, Montreal, Canada*, ^10^*Scleroderma Patient-centered Intervention Network, Montreal, Canada*

**Introduction:** Many people with scleroderma rely on peer-led support groups as a coping resource. Reasons for not attending support groups in scleroderma have been investigated only in North American participants. It is important to determine the degree to which reasons for not attending support groups among North American are similar to reasons for non-attendance among European patients, or if barriers to participation differ. If barriers for participation are similar across Europe and North America, patient organizations who facilitate these support groups may benefit from collaborating to develop strategies to improve accessibility to support groups for patients with scleroderma who may wish to join. This study assesses reasons for non-attendance in European countries and compares results with previously published North American findings.

**Material and Methods:** An open survey was completed by a convenience sample of people with scleroderma who were not current members of support groups. The same 21-item survey as used in the North American sample assessed possible reasons for not attending scleroderma support groups, clustered in three themes relating to (1) personal reasons, (2) practical reasons, and (3) beliefs about support groups. Proportions of items rated Important or Very Important were compared between samples.

**Results:** Consistent with the North American survey findings (N=242), the two items most commonly rated as (Very) Important reasons for non-attendance among 228 European participants were (1) already having enough support (57%), and (2) not knowing of any local scleroderma support groups (58%). Compared to North American non-attenders, European patients were significantly more likely to rate not knowing enough about what happens at support groups (46% vs 19%), not having reliable ways to get to meetings (35% vs 17%), and being uncomfortable sharing experiences with a group (22% vs 11%) as (Very) Important reasons for non-attendance.

**Conclusions:** The results of this study confirm that the reasons for not attending support groups are overlapping between two large international samples of patients with scleroderma, and thus, that it could be beneficial for European and North American scleroderma organizations to work together to identify ways to improve access to support groups and their ability to meet needs of people with scleroderma. In addition, improving education about support groups and what they entail may be an important strategy to improve support group attendance in the European context.

## P.178

## MARKERS OF COLLAGEN TURNOVER ARE ALTERED IN PATIENTS WITH VERY EARLY SYSTEMIC SCLEROSIS: EARLY MARKERS OF TISSUE CHANGES

R. Dobrota^1^, S. Jordan^1^, P. Juhl^2^, B. Maurer^1^, L. Wildi^1^, A.-C. Bay-Jensen^2^, M.A. Karsdal^2^, A. Herrick^3^, J. Distler^4^, Y. Allanore^5^, A.-M. Hoffmann-Vold^6^, A.S. Siebuhr^2^, O. Distler^1^

^1^*Department of Rheumatology; University of Zurich, Zurich, Switzerland*, ^2^*ImmunoScience, Nordic Bioscience, Biomarkers and research, Herlev Hovedgade 205-207, Herlev, Denmark*, ^3^*Division of Musculoskeletal & Dermatological Sciences, University of Manchester, Salford Royal Hospital NHS, Manchester, United Kingdom*, ^4^*Department of Internal Medicine 3, Friedrich-Alexander-Universität Erlangen-Nürnberg, Erlangen, Germany*, ^5^*Department of Rheumatology, University Paris Descartes and Cochin Hospital, Paris, France*, ^6^*Department of Rheumatology, Rikshospitalet, Oslo University Hospital, Oslo, Norway*

**Introduction:** The very early diagnosis of patients with systemic sclerosis (veSSc) is important for a personalized follow-up and optimal timing of treatment. In this study, we hypothesise that changes in extracellular matrix (ECM) turnover, measured by ECM-degradation and formation markers are present very early in SSc, before clinical fibrosis occurs. We investigated serum-based ECM turnover markers as potential diagnostic biomarkers for veSSc.

**Material and Methods:** Patients with veSSc, defined as presence of Raynaud’s syndrome and at least one of the following: puffy fingers, positive antinuclear antibodies or pathological nailfold capillaroscopy, without meeting any classification criteria for SSc, were included, and compared to healthy controls (HC, n=29). Data and sera collection were conducted by EUSTAR/VEDOSS standards. ECM-degradation (BGM, C3M, C4M, C6M) and -formation markers (PRO-C3, PRO-C4, PRO-C5) were measured in serum using ELISA-based assays. The statistical analyses included Mann-Whitney U, Spearman correlation and ROC analysis.

**Results:** Compared to HC, veSSc patients had higher levels of degradation markers of type III and IV collagens (C3M, C4M, both p<0.0001) and of formation marker of type III collagen (PRO-C3, p=0.001) with an overall lower turnover of type III and IV collagen (PRO-C3/C3M, PRO-C4/C4M, both p<0.0001). Higher levels of the biglycan degradation marker BGM (p=0.005) and lower levels of the type VI collagen degradation marker C6M (p=0.004) were observed in veSSc (Figure 1). In ROC analysis, markers of type III and IV collagen turnover could distinguish between veSSc and HC: C3M, AUC=0.95, p<0.0001; C4M, AUC=0.97, p<0.0001; PRO-C3/C3M, AUC=0.80, p<0.0001; PRO-C4/C4M, AUC=0.96; p<0.0001.

**Conclusions:** ECM turnover is altered in veSSc patients compared to HC. Biomarkers of type III and IV collagens could distinguish between veSSc patients and HC, which may indicate that these markers are potential diagnostic biomarkers complement clinical and immunological veSSc criteria to assess risk of developing fibrosis related to SSc.

## P.179

## TYPE III AND TYPE IV COLLAGEN BIOMARKERS PREDICT PROGRESSION OF FIBROSIS IN SYSTEMIC SCLEROSIS

R. Dobrota^1^, S. Jordan^1^, P. Juhl^2^, B. Maurer^1^, L. Wildi^1^, A.-C. Bay-Jensen^2^, M.A. Karsdal^2^, A. Herrick^3^, J.H. Distler^4^, Y. Allanore^5^, A.-M. Hoffmann-Vold^6^, A.S. Siebuhr^2^, O. Distler^1^

^1^*Department of Rheumatology, University of Zurich, Zurich, Switzerland*, ^2^*ImmunoScience, Nordic Bioscience, Herlev Hovedgade 205-207, Herlev, Denmark*, ^3^*Division of Musculoskeletal & Dermatological Sciences, The University of Manchester, Salford Royal Hospital NHS, Manchester, United Kingdom*, ^4^*Department of Internal Medicine 3, Friedrich-Alexander-Universität Erlangen-Nürnberg, Erlangen, Germany*, ^5^*Department of Rheumatology, University Paris Descartes and Cochin Hospital, Paris, France*, ^6^*Department of Rheumatology, Oslo University Hospital, Oslo, Norway*

**Introduction:** Remodeling of the extracellular matrix (ECM) plays a key pathogenic role in systemic sclerosis (SSc). In this study, we evaluated ECM neo-epitopes as potential serum biomarkers for progression of fibrosis in SSc.

**Material and Methods:** Patients meeting the 2013 ACR/EULAR classification criteria for SSc and healthy controls (HC) from a derivation and validation cohort were analyzed. Fibrosis progression was defined as decline in FVC% predicted equal or more than 10% or increase in mRSS equal or more than 25% AND >5 points at one-year follow-up. Longitudinal assessment and biobanking were performed by EUSTAR standards. ECM-degradation (biglycan [BGM], type III, IV and VI collagen [C3M, C4M, C6M]) and ECM-formation biomarkers (formation of type I, III, IV, V and VI collagen [PRO-C1, PRO-C3, PRO-C4, PRO-C5, PRO-C6]) were measured in serum using validated ELISA. Statistical analysis included Mann-Whitney U, receiver operating curve, logistic regression, and Bonferroni correction for multiple testing.

**Results:** The derivation cohort included 29 HC and 158 SSc patients. Twenty-three patients (14.5%) showed fibrosis progression. Type III and IV collagen biomarkers and BGM were significantly higher in SSc compared to HC. Type III, IV and VI collagen markers were higher in SSc patients (acc. to the ACR1980 criteria) indicating more advanced disease (PRO-C3/C3M, p=0.002; PRO-C6, p=0.001) with positive Scl70 antibodies (PRO-C4, p=0.001; C6M, p<0.001) and active disease defined by Valentini activity index >=3 points (PRO-C3, p<0.001; PRO-C3/C3M, p=0.001; PRO-C6, p<0·001). Degradation of type III and IV collagens and their turnover ratios distinguished between progressive and non-progressive patients (C3M, p<0.0001, AUC=0.90; PRO-C3/C3M, p<0.0001, AUC=0.85; C4M, p<0.0001, AUC=0.82; PRO-C4/C4M, p<0.0001, AUC=0.9). PRO-C3/C3M and PRO-C4/C4M predicted fibrosis progression (OR 2.83 95%CI [1.78-4.49], p<0.0001 and OR 1.33, 95%CI [1.09-1.62], p=0.006, respectively). The validation analysis in 384 patients (102, 26.6% progressors) and 60 HC confirmed that higher levels of PRO-C3 and PRO-C4/C4M at baseline were associated with progression of fibrosis at one-year follow-up. Further, these biomarkers were confirmed as independent predictors of progression of skin and lung fibrosis, separately, in a pooled analysis of both cohorts.

**Conclusions:** ECM turnover markers are dysregulated in SSc, associated with more severe disease phenotype and their baseline levels distinguished between progressive and non-progressive fibrosis at one-year follow up. The turnover ratio of type IV collagen (PRO-C4/C4M) and the formation marker of type III collagen (PRO-C3) were identified as predictors of progression of fibrosis at one-year, suggesting they could be prognostic biomarkers for SSc.

**Figure:**
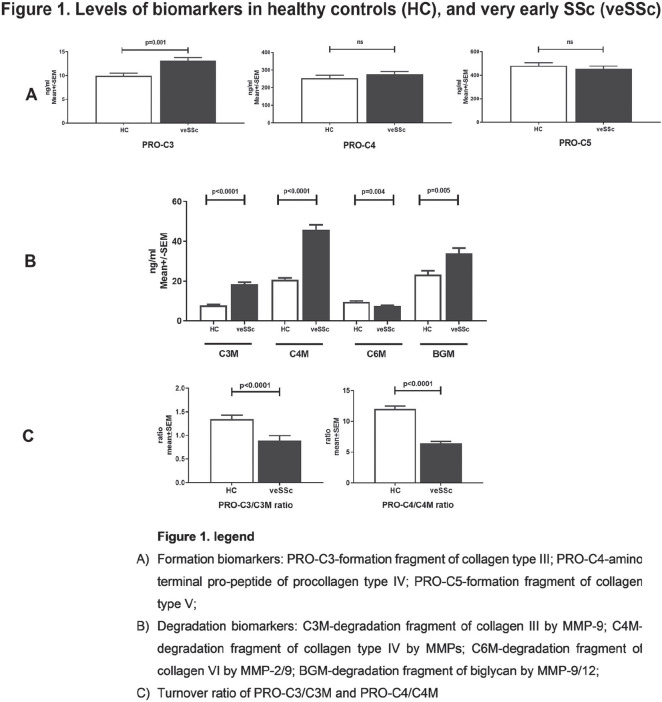


## P.180

## EPIDEMIOLOGY OF EMPLOYMENT OUTCOMES IN SYSTEMIC SCLEROSIS. A SYSTEMATIC REVIEW

J. Lee^1,2,3^, K. Devakandan^1,2^, M. Anderson^4^, M. Gignac^5^, S. Johnson^1,2,3^

^1^*Toronto Scleroderma Program, Toronto, Canada*, ^2^*Mount Sinai Hospital, Toronto Western Hospital, Department of Medicine, Division of Rheumatology, Toronto, Canada*, ^3^*University of Toronto, Toronto, Canada*, ^4^*University Health Network Library Services, Toronto General Hospital, Toronto, Canada*, ^5^*Institute for Work and Health, Dalla Lana School of Public Health, University of Toronto, Toronto, Canada*

**Introduction:** Systemic sclerosis (SSc) is a chronic autoimmune condition that predominantly occurs between the ages of 30-50 years old, putting individuals at significant risk of poor workforce health and work disability. Work disability can be defined as either loss of employment or work-related productivity. The epidemiology, including determinants of SSc-related work disability, are poorly understood. The objectives of this study were to evaluate unemployment rates, work productivity outcomes, and risk factors associated with work disability in patients with SSc.

**Material and Methods:** A systematic search of Ovid Medline, Embase, and Cochrane Central Register of Controlled Trials, PsycINFO, CINAHL, and Clinicaltrials.gov was performed to identify studies from inception to July 2018. Included search terms were relevant to employment and systemic sclerosis. Studies were included for data extraction if they met the following inclusion criteria: 1) human participants with a diagnosis of SSc, 2) controlled trials or observational studies, 3) the study described employment status, or reported an outcome related to employment or work-related disability, and 4) the study presented original data. We extracted information on study setting, design, patient population and size, employment outcomes (both work loss prevalence and word productivity outcomes), and risk factors for work disability.

**Results:** From 4319 abstracts, we included 43 quantitative studies from 13 countries. Thirty-five studies identified the employment status of 11,315 individuals. The majority of these studies (86%) were cross-sectional in design and employment definitions were based on self-report. Unemployment rates varied from 4% and 92% (mean unemployment prevalence: 40.53%) after a mean disease duration of 10.1 years. Twenty-three studies reported work-related productivity outcomes. Productivity definitions varied significantly and in 80% of the studies they were captured by non-standardized, self-report measures (e.g. self-defined as work disabled, having professional difficulties, or required sick leave). Significant risk factors for unemployment included older age, less education, worse physical function, longer disease duration, and reduced quality of life scores.

**Conclusions:** Work disability is an important outcome for patients with SSc conferring significant unemployment and work productivity burden. Determinants of work disability relate to demographic and disease-related characteristics, but few studies evaluate the impact of work-context related factors on employment sustainability or productivity. Few studies utilized validated work productivity measures and there are no studies evaluating employment interventions in the SSc population.

## P.181

## GASTROINTESTINAL SYMPTOM BURDEN AND QUALITY OF LIFE IN SYSTEMIC SCLEROSIS: UNDERSTANDING THE ROLE OF DIET

K. Jensen^1^, L. Wang^2^, R. Kovacic^2^, M. Kowalczyk-Lammi^3^, L.A. Saketkoo^1^

^1^*Tulane University School of Medicine, New Orleans, USA*, ^2^*Tulane University School of Public Health and Tropical Medicine, New Orleans, USA*, ^3^*Ochsner Health System, New Orleans, USA*

**Introduction:** Gastrointestinal (GI) impairment is experienced by >90% of systemic sclerosis (SSc) patients. Symptomatic complications occur diffusely throughout the GI tract with dysmotility and obstruction causing pain, nausea, vomiting, diarrhea, constipation, and soiling eroding health-related quality of life (HRQoL). Currently GI involvement garners only symptomatic treatment with limited medication options. While diet has been an area of interest for SSc patients, the role of dietary intervention in SSc is not clear. The impact of diet on SSc symptom burden and HRQoL measures are examined here.

**Material and Methods:** An international three-armed randomized controlled single-blind study comparing 4-week interventions of gluten-free (GF) vs Mediterranean (M) vs low FODMAP (LF) diets without portion/calorie restriction. Inclusion required rheumatologist ACR/EULAR SSc documentation with negative celiac serum or tissue assay. Telephone counseling provided diet instruction and support. Primary and secondary endpoints are patient-reported outcomes measures (PROMs). SSc Gastrointestinal Tract (GIT) is primary with Geissen SSc Gastrointestinal (GGIQ) as exploratory co-primary, secondary endpoints included SF-36, Cochin Hand Function Scale (CHFS), Scleroderma Health Assessment Questionnaire (SHAQ) and Fatigue Severity Scale (FSS) at 0, 4, 8 weeks via REDCap, paper or telephone. Medication changes, adherence and self-efficacy were controlled for via interval history, adapted MOS adherence scale and Illness Behavior Questionnaire (IBQ), respectively. Data was analyzed using STATA ANOVA models.

**Results:** To date 42 subjects completed the study (12, 14 and 16 respectively in M, GF, and LF groups). No differences emerged in baseline demographics age, gender, race, ethnicity, skin distribution or disease duration between the three groups. No significant differences occurred overall or between diet groups in SF-36 total score, CHFS, FSS, and HAQ. The LF group demonstrated significant improvement in GGIQ day-time heartburn (p=0.03), SHAQ GI and Raynaud VAS (p=0.03, 0.01) with non-significant improvement in GGIQ cough, swallow, and vomiting categories (p=0.06, 0.07, 0.07) as well as GIT reflux, bloating and overall scores (p=0.08, 0.07, 0.08). Mean adherence was 82.3%; the GF group demonstrated higher adherence (90.3%) than M and LF groups (75.3% and 80.5% respectively) (p=0.023).

**Conclusions:** At the time of analysis, 50% of powered recruitment had been met. As such, these preliminary findings are interesting; however should be interpreted cautiously. The low FODMAP group demonstrated significant improvement as well as non-significant improvement trends in several symptom areas suggesting that dietary changes may play a role in management of SSc symptom burden.

## P.182

## A SYSTEMATIC REVIEW OF INFORMATION AVAILABLE ON THE INTERNET FOR INDIVIDUALS WITH RAYNAUD’S PHENOMENON AND PATIENTS WITH SYSTEMIC SCLEROSIS

V. Devgire, A.F. Martin, R. Sandler, M. Hughes

*Sheffield Teaching Hospitals NHS Foundation Trust, Department of Rheumatology, Sheffield, United Kingdom*

**Introduction:** Patients are increasingly using internet-based information to inform healthcare utilisation and treatment decisions. Therefore, this information needs to be of both high quality and appropriate readability. Our aim was to examine the quality and readability of the information available on the internet relating to Raynaud’s phenomenon (RP) and systemic sclerosis (SSc).

**Material and Methods:** Two independent reviewers searched three engines (Google®, Yahoo® and Bing®) using the terms “Raynaud’s phenomenon” and “systemic sclerosis”. The first 30 websites identified per search engine were examined. Quality was assessed using the DISCERN questionnaire (range 0-80) comprising of 16 questions, individually rated 1 (low) to 5 (high). Questions relate to the reliability of the publication (Q 1-8), information on treatment choices (Q9-15), and an overall view of the website (Q16). Readability was assessed using validated methods including the Flesch Reading Ease score (0-100: a higher score indicates higher readability, meaning it is easier to read), Flesch-Kincaid Grade Level and the SMOG Index, both of which propose the minimum attained US educational grade required to read the text. It is proposed that health care literature should be written at US 6th grade reading level or below.

**Results:** We included 30 websites for RP and 22 for SSc after duplicates and those with specified exclusion criteria were removed.

For RP and SSc, the overall quality, as per DISCERN (range 0-80), was low (mean, [SD]): 29.46 (10.25) and 31.31 (11.64) respectively. This included website reliability (Q 1-8): 1.99 (0.91) and 2.21 (0.86), information on treatment choices (Q9-15): 1.70 (0.51) and 1.68 (0.49), and overall grade (Q16): 1.73 (1.11) and 1.81 (1.18).

For RP and SSc, readability was poor (i.e. the texts were difficult to read). The Flesch Reading Ease scores (range 0-100) were 52.44 (10.77) and 43.22 (13.52) respectively. Flesch-Kincaid Grade Levels were 9.7 (1.85) and 11.31 (2.22), and SMOG index was 9.06 (1.46) and 10.55 (1.66).

**Conclusions:** Overall, the information available on the internet relating to RP and SSc is of low quality and of inadequate readability. The content of internet information needs to be improved, both in terms of quality and readability, to facilitate early diagnosis and to support patients in making informed decisions regarding their care. Future studies should also examine ‘accuracy’ of internet information, which would require the development of patient standards of care. The RP and SSc international community should strongly consider developing an information standard for internet-based resources for healthcare users.

## P.183

## ASSESSMENT OF THE QUALITY OF LIFE OF PATIENTS WITH SYSTEMIC SCLEROSIS –QUANTITATIVE ANALYSIS OF SOCIODEMOGRAPHIC, CLINICAL AND PSYCHOLOGICAL INFLUENCING FACTORS

S. Heyne^1^, E. Haufe^2^, S. Beissert^2^, C. Günther^1^

^1^*Department of Dermatologym, University Hospital Dresden, Dresden, Germany*, ^2^*Center for Evidence-Based Health Care, Technical University Dresden, Dresden, Germany*

**Introduction:** Systemic Sclerosis (SSc) is a rare progressive and life quality limiting disease. Life time prevalence of depression in patients with SSc (23-46%) is higher compared with prevalence in general German population (11,6% ). The relationship between disease burden and depressive symptoms is currently not well known. This indicates a high need for defining risk factors for depression and main determinants of quality of life in SSc.

**Material and Methods:** We performed a cross-sectional study with 79 patients in 2018 and a longitudinal study with 33 patients over 10 years (2008-2018). We used validated questionnaires such as Health Assessment Questionnaire (HAQ), disease specific quality of life score of EuroQOL (EQ-5D-3L-Index), the Center for Epidemiologic Studies Depression scale (CES-D), Score of EUlar Scleroderma Trials and Research Group (EUSTAR) and modified Rodnan-Skin-Score (mRSS).

**Results:** The population consisted of 79 patients (64 women=81%) with a mean age of 61,51y (±12,59y) and mean disease duration of 12,12y (±10,27y). Most patients had a limited SSc (62,0%). 46,2% of the patients had an EUSTAR-Score>3 which represents an active disease. Depressive symptoms, indicated by CES-D > 16, were found in 42,3 % of patients with SSc. Mean EQ-5D-3L-Index was 0,726 (±0,256), which was less compared with the mean EQ-5D-3L-Index in general German population (0,93) and it indicates a reduced quality of life in SSc patients. Furthermore, mean EQ-5D-VAS was 59,38 (±21,74) demonstrating a low subjective state of health. CES-D, HAQ, EQ-5D-Index and-VAS correlated significantly (p<0,001) with each other and CES-D>16 correlated significantly with pain (p=0,002), muscle weakness (p=0,008) and PGA (p=0,009).

The longitudinal analysis revealed a significantly increasing mean EUSTAR- Score (2008: 1,069(±0,740), 2018: 2,266 (±1,581), p<0,001), showing a disease progress. In parallel we observed a significant increase in disease associated disability (HAQ: 2008: 0,689 (±0,565), 2018: 1,101 (±0,717), p<0,001). Mean CES-D also tended to increase: 2008: 17,31 (± 9,94), 2018: 18,727 (± 10,88), p=0,523). EQ-5D-3L-index (2008: 0,743 ±0,291 2018: 0,656 ±0,307, p=0,057) and EQ-5D-VAS decreased (2008: 62,61% ±19,15%, 2018: 55,97%±22,20% p=0,113), which demonstrated that quality of life and subjective state of health worsened.

**Conclusions:** In conclusion, patients with SSc have a higher burden of depression compared with the general population. Disease progress is paralleled by increasing disability, lower quality of life and by an increase in depressive symptoms. The high psychosomatic morbidity warrants interdisciplinary models of care for SSc-patients.

## P.184

## DOES SCLERODERMA HAVE AN IMPACT ON SEXUAL FUNCTION AND PELVIC FLOOR FUNCTION? A CROSS-SECTIONAL STUDY IN 80 WOMEN WITH SYSTEMIC SCLEROSIS

B. Hermankova^1^, S. Oreska^2,3^, H. Storkanova^2,3^, M. Spiritovic^1^, K. Bubova^2,3^, H. Smucrova^2^, P. Cesak^1^, K. Pavelka^2,3^, L. Senolt^2,3^, J. Vencovsky^2,3^, R. Becvar^2,3^, M. Tomcik^2,3^

^1^*Faculty of Physical Education and Sport, Charles University, Prague, Czech Republic*, ^2^*Institute of Rheumatology, Prague, Prague, Czech Republic*, ^3^*1st Faculty of Medicine, Charles University, Prague, Czech Republic*

**Introduction:** Systemic sclerosis (SSc) is a chronic multisystem disease that may affect all aspects of life including sexual function. The purpose of this study was to assess sexual function, pelvic floor function and sexual quality of life in 80 women with SSc compared to age-/sex-matched healthy controls (HC).

**Material and Methods:** In total 80 women with SSc (mean age:49, disease duration:6.1 years, lcSSc/dcSSc: 53/27, mRSS:9.3, ESSG activity index:2.1), who fulfilled the ACR/EULAR 2013 criteria, and 80 healthy women with identical age (mean age:49) filled in 12 well-established and validated questionnaires assessing sexual function:Female Sexual Function Index (FSFI), Brief Index of Sexual Function for Women (BISF-W), pelvic floor function:Pelvic Organ Prolapse/Urinary Incontinence Sexual Questionnaire (PISQ-12), Pelvic Floor Distress Inventory Questionnaire (PFIQ7); sexual quality of life:Sexual Quality of Life Questionnaire (SQoL-F); fatigue:Fatigue Impact Scale (FIS), Multidimensional Assessment of Fatigue (MAF); physical activity:Human Activity Profile (HAP); health status:Health Assessment Questionnaire (HAQ), Scleroderma Health Assessment Questionnaire (SHAQ); overall quality of life:36-Item Short Form Survey (SF-36) and depression: Beck’s Depression Inventory-II (BDI-II). Data are presented as mean±SEM.

**Results:** Compared to HC, women with SSc had significantly higher prevalence and greater severity of sexual dysfunction [FSFI in total score (SSc:16.4±1.3,HC:25.6±1.2, p<0.0001) as well as in all subscales (p<0.0001), BISF-W total score (SSc:17.2±2.3,HC:32.0±1.9,p<0.0001)], dysfunction of pelvic floor [(PISQ-12(SSc:3.6±0.7,HC:8.9±0.6, p<0.0001), PFIQ7(SSc:33.4±5.6,HC: 6.8±1.4,p<0.0001)], and worse sexual quality of life [SQol-F(SSc:55.3±3.3,HC:82.1±2.1,p<0.0001)]. Worse scores in SSc patients were associated with higher disease activity [ESSG activity index:FSFI Arousal domain (r=-0.300,p=0.007)], greater fatigue [FIS:BISF-W (r=-0.410,p=0.0002), MAF:FSFI (r=-0.399,p=0.0002)], more severe depression [BDI-II:BISF-W (r=-0.381,p=0.002)], deteriorated quality of life [SHAQ:FSFI (r=- 0.409,p=0.000), BISF-W (r=-0.451,p=0.0002), SQol-F (r=-0.390,p=0.005)] and worse ability to perform physical activities [HAP:SQoL-F (r=0.383,p=0.0006), BISF-W (r=0.456,p=0.0001)]. Worse scores (in several domains of FSFI and BISF-W;p<0.05 for all comparisons) were detected in SSc women with increased CRP (>5 vs. <5mg/L) and ESR (>18 vs. <18mm/h). Better scores (FSFI total, BISFW total, PISQ-12;p<0.05 for all) were observed in SSc women with tertiary than in secondary education.

**Conclusions:** Women with SSc reported significantly impaired sexual function and sexual quality of life and pelvic floor function compared to healthy controls with identical age. Worse scores in SSc were associated with disease activity, more severe functional impairment, physical inactivity, fatigue, depression and decreased quality of life.

Acknowledgement: Supported by MHCR-023728, SVV–260373, GAUK-1578119.

## P.185

## ERECTILE DYSFUNCTION AND SEXUAL HEALTH IN MEN WITH SYSTEMIC SCLEROSIS

B. Hermankova^1^, S. Oreska^2,3^, H. Storkanova^2,3^, M. Spiritovic^1^, K. Bubova^2,3^, H. Smucrova^2^, P. Cesak^1^, K. Pavelka^2,3^, L. Senolt^2,3^, J. Vencovsky^2,3^, R. Becvar^2,3^, M. Tomcik^2,3^

^1^*Faculty of Physical Education and Sport, Charles University, Prague, Czech Republic*, ^2^*Institute of Rheumatology, Prague, Czech Republic*, ^3^*1st Faculty of Medicine, Charles University, Prague, Czech Republic*

**Introduction:** Erectile dysfunction (ED) is a common issue in systemic sclerosis (SSc) and it has been observed in around 80 to 90% of men with this connective tissue disorder. The purpose of this study was to assess sexual function, pelvic floor function and sexual quality of life in men with SSc compared to healthy men with identical age (HC).

**Material and Methods:** In total 14 men with SSc (mean age:51.6, disease duration:3.1 years, lcSSc/dcSSc:6/8, mRSS:12.9, ESSG activity index:2.2), who fulfilled the ACR/EULAR 2013 criteria, and 14 healthy men with identical age (mean age:51.6) filled in 11 well-established and validated questionnaires assessing sexual function: International Index of Erectile Function (IIEF), Male Sexual Health Questionnaire (MSHQ); pelvic floor function: Pelvic Floor Distress Inventory Questionnaire (PFIQ7); sexual quality of life: Sexual Quality of Life Questionnaire (SQoL-M); fatigue: Fatigue Impact Scale (FIS), Multidimensional Assessment of Fatigue (MAF); physical activity: Human Activity Profile (HAP); health status: Health Assessment Questionnaire (HAQ), Scleroderma Health Assessment Questionnaire (SHAQ); overall quality of life: 36-Item Short Form Survey (SF-36) and depression: Beck’s Depression Inventory II (BDI II). Data are presented as mean±SEM.

**Results:** Compared to HC, men with SSc had significantly higher prevalence and greater severity of sexual dysfunction [IIEF-Erectile function (EF) (SSc:15.4±3.0,HC:26.2±2.1,p=0.004), IIEF-Sexual Desire (SD) (SSc:12.8±2.0,HC:19.8±1.4,p=0.006), IIEF-Orgasmic function (OF) (SSc:6.4±1.2,HC:9.0±0.6,p=0.045), MSHQ-Erectile Function (SSc:9.0±1.2,HC:12.9±0.9,p=0.005), MSHQ-Satisfaction (SSc:19.2±2.1,HC:26.8±0.9,p=0.001)] and worse sexual quality of life [SQol-M (SSc:68.5±7.4,HC:86.7±6.2,p=0.023)]. According to the IIEF classification, 71% of SSc men reported mild to severe ED, whereas half of them suffer from severe ED. Worse scores in SSc patients were associated with higher disease activity [ESSG activity index:IIEF-EF (r=-0.597,p=0.024), MSHQ-Satisfaction (r=-0.686, p=0.013)], increased systemic inflammation [CRP:IIEF-OF (r=-0.668, p=0.008), ESR:MSHQ-Satisfaction (r=-0.586, p=0.044)], more extensive skin involvement [mRSS:MSHQ-SD/AS (r=0.555, p=0.061)], greater fatigue [FIS:IIEF-EF (r=-0.772, p=0.001), IIEF-SD (r=-0.750, p=0.002)], more severe depression [BDI-II:IIEF-EF(r=-0.801,p=0.0005)], deteriorated quality of life [HAQ:IIEF-EF (r=-0.545,p=0.043), MSHQ-Satisfaction (r=-0.633, p=0.027)] and worse ability to perform physical activities [HAP:IIEF-EF (r=0.639,p=0.006), IIEF-SD (r=0.634,p=0.014)].

**Conclusions:** Men with SSc reported significantly impaired sexual function and sexual quality of life than healthy controls with identical age. Worse scores in SSc were associated with disease activity, inflammation, skin involvement, more severe functional impairment, physical inactivity, fatigue, depression and decreased quality of life. 71% of SSc men reported erectile dysfunction.

Acknowledgement: Supported by MHCR 023728, SVV–260373 and GAUK-1578119.

## P.186

## INVESTIGATING DIFFERENTIAL ITEM FUNCTIONING ON THE SOCIAL APPEARANCE ANXIETY SCALE FOR FRENCH- AND ENGLISH-SPEAKING PATIENTS: A SCLERODERMA PATIENT-CENTERED INTERVENTION NETWORK COHORT STUDY

S. Sommer^1^, D. Harel^1^, S. Mills^2^, L. Kwakkenbos^3^, M.-E. Carrier^4^, S. Gholizadeh^5^, A. Portales^6^, K. Nielson^7^, S. Bartlett^8^, V. Malcarne^9^, B. Thombs^4,5,10^

^1^*New York University - Department of Applied Statistics, Social Science, and Humanities, New York, USA*, ^2^*University of North Carolina - Department of Health Behavior, Chapel Hill, USA*, ^3^*Radboud University Nijmegen - Behavioural Science Institute, Clinical Psychology, Nijmegen, The Netherlands*, ^4^*Lady Davis Institute for Medical Research - Jewish General Hospital, Montreal, Canada*, ^5^*McGill University - Department of Psychiatry, Montreal, Canada*, ^6^*Asociación Española de Esclerodermia, Madrid, Spain*, ^7^*Scleroderma Society of Ontario, Hamilton, Canada*, ^8^*McGill University - Department of Medicine, Montreal, Canada*, ^9^*San Diego State University - Department of Psychology, San Diego, Canada*, ^10^*McGill University - Department of Psychology, Montreal, Canada*

**Introduction:** Social appearance anxiety, a fear of appearance-based evaluation, is relevant among patients with scleroderma because of appearance changes in socially-relevant body areas. The 16-item Social Appearance Anxiety Scale (SAAS) is a measure of social appearance anxiety that has been validated in patients with scleroderma. Patients enrolled in the Scleroderma Patient-centered Intervention Network (SPIN) can complete the SAAS in French or English, depending on their native language. Therefore, it is necessary to investigate whether items on the 16 SAAS function in a similar manner in English- and French- speaking respondents. If the scores are not equivalent, the scale is said to contain differential item functioning (DIF). This research investigated DIF for the SAAS with the goal of either confirming that scores are comparable across languages or providing guidance on how to calculate comparable scores. A secondary objective was to separately investigate the existence of DIF for patients of differing sex and disease status.

**Material and Methods:** This study involved 1640 patients enrolled in SPIN who completed the SAAS in either French (N=600) or English (N=1040). A generalized partial credit model (GPCM) was used to model the latent factor (i.e. social anxiety with appearance) underlying the SAAS. Detection of items with DIF was done using the lordif package in R at the alpha = 0.01 level. After identifying items with potential DIF, the impact on estimated latent factor scores was then assessed by comparing initial scores (not accounting for DIF) to final scores (accounting for DIF).

**Results:** We identified six items with language-based DIF, four items with sex-based DIF, and one item with disease type-based DIF. Thus, we found systematic differences in how patients with the same level of social appearance anxiety responded to some items based on their language of administration, their sex, and their disease type. However, scores before and after accounting for DIF were similar (correlation > .99). Thus, despite these item differences, overall, there was no evidence that the DIF items influenced SAAS scores substantially.

**Conclusions:** We conclude that scores are comparable across language, sex, and disease-type groups, despite differences in how patients respond to some items. Therefore, scores generated with the SAAS in English and French scleroderma patients can be reasonably pooled without adjustment for linguistic differences. The importance of assessing cross-language measurement equivalence prior to pooling outcomes obtained in different languages should be emphasized.

## P.187

## USE OF A COHORT-BASED SURVEY TO IDENTIFY IMPORTANT BARRIERS AND FACILITATORS TO PHYSICAL ACTIVITY FOR PEOPLE WITH SCLERODERMA: A SCLERODERMA PATIENT-CENTERED INTERVENTION NETWORK COHORT STUDY PROTOCOL

S. Harb^1^, S. Peláez^2,3^, B.D. Thombs^2-7^, I. Shrier^2^, S.-P. Patient Advisory Team^2^

^1^*Jewish General Hospital - Lady Davis Institute for Medical Research; McGill University - Department of Psychiatry, Montreal, Canada*, ^2^*Jewish General Hospital - Lady Davis Institute for Medical Research, Montreal, Canada*, ^3^*McGill University - Department of Educational and Counselling Psychology, Montreal, Canada*, ^4^*McGill University - Department of Epidemiology, Biostatistics and Occupaitonal Health, Montreal, Canada*, ^5^*McGill University - Department of Medicine, Montreal, Canada*, ^6^*McGill University - Department of Psychiatry, Montreal, Canada*, ^7^*McGill University - Department of Psychology, Montreal, Canada*

**Introduction:** Despite the many barriers that people with systemic sclerosis (SSc; scleroderma) face to being physically active, regular physical activity continues to be a key component of SSc self-management. We conducted nine nominal group technique sessions with 41 total SSc patients in which we identified 20 physical activity barriers and 103 physical activity facilitators (N = 91 barrier-specific and 12 general) that allowed patients to remain physically active. Our objective is therefore to assess the frequency and the importance attributed to barriers to physical activity as identified by people with SSc, as well as patient-perceived likelihood of using the facilitators to overcome physical activity barriers.

**Material and Methods:** We will invite 1706 SSc patients, fluent in English or French, enrolled in the Scleroderma Patient-centered Intervention Network (SPIN) Cohort to complete a survey that was developed based on the nominal group technique results. Participants who choose to complete the survey will first be presented with a list of 20 barriers, and they will select 1-10 barriers that they experience as important to them. Next, participants who select > 1 barrier will then rank selected barriers in order of importance. They will rate each of their selected barriers on a 4-point Likert scale from 1 (‘not important’) to 4 (‘very important’) based on the perceived importance of the barrier to them. In addition, using a 4-point Likert scale from 1 (‘not likely’) to 4 (‘very likely’), they will rate, for each of their selected barriers, the proposed barrier-specific facilitators based on their perceived likelihood of using the facilitators to overcome the corresponding barriers to be physically active. Following this, participants will rate all proposed general facilitators using the same scale based on their perceived likelihood of using the facilitators to be physically active. For all facilitators that are presented to participants, they will indicate whether they had tried facilitators. At the end of the survey, participants will have the opportunity to provide suggestions for additional barriers and facilitators that were not presented.

**Results:** SPIN Cohort participants will complete outcome assessments every 3 months, and survey results will be available in November 2019 for presentation in March 2020.

**Conclusions:** Results of the current study will determine which barriers and facilitators need to be prioritized when adapting a widely used online physical activity promotion program to the needs and safety concerns of people with SSc. The adapted program will be subsequently tested in a large randomized controlled trial.

## P.188

## IDENTIFYING BARRIERS AND FACILITATORS TO PHYSICAL ACTIVITY FOR PEOPLE WITH SCLERODERMA: A NOMINAL GROUP TECHNIQUE STUDY

S. Harb^1^, J. Cumin^2^, D.B. Rice^2,3^, S. Peláez^2,4^, M. Hudson^2,5^, S.J. Bartlett^5^, A. Roren^6-8^, D.E. Furst^9^, T.M. Frech^10^, C. Nguyen^11-13^, W.R. Nielson^14^, B.D. Thombs^2-5 ,15^, I. Shrier^2^, SPIN-PACE Patient Advisory Team^2^

^1^ *Jewish General Hospital - Lady Davis Institute for Medical Research; McGill University - Department of Psychiatry, Montreal, Canada*

^2^Jewish General Hospital - Lady Davis Institute for Medical Research, Montreal, Canada, ^3^McGill University - Department of Psychology, Montreal, Canada, ^4^McGill University - Department of Educational and Counselling Psychology, Montreal, Canada, ^5^McGill University - Department of Medicine, Montreal, Canada, ^6^AP-HP Hôpital Cochin, Paris, France, ^7^Université Paris Descartes Sorbonne Paris Cité, Paris, France, ^8^INSERM U1153, Paris, France, ^9^University of California, Los Angeles, Geffen School of Medicine - Division of Rheumatology, Los Angeles, USA, ^10^University of Utah - Department of Internal Medicine, Salt Lake City, USA, ^11^Université de Paris, Faculté de Santé - UFR Médecine Paris Descartes, Paris, France, ^12^AP-HP Hôpital Cochin - Service de Rééducation et de Réadaptation de l’Appareil Locomoteur et des Pathologies du Rachis, Paris, France, ^13^INSERM UMR 1124, Paris, France, ^14^St. Joseph’s Health Care and the Lawson Health Research Institute, London, Canada, ^15^McGill University - Department of Epidemiology, Biostatistics and Occupational Health; Department of Psychiatry, Montreal, Canada

**Introduction:** Most people with systemic sclerosis (SSc; scleroderma) can perform aerobic and resistance exercise safely. Moreover, regular physical activity is often encouraged. Many of these patients, however, face barriers to being physically active. Our objective was to identify physical activity barriers and facilitators experienced by people with SSc. These will later be used in a survey that will guide the development of an online physical activity promotion program for people with SSc.

**Material and Methods:** We conducted a series of 90-120 minute nominal group technique sessions with 3-8 SSc patients per session at patient conferences in Canada, the USA, and France. In each session, participants first identified a personal list of examples of physical activity barriers. Participants shared one barrier at a time from their list, in a round-robin format, that was projected onto a screen. Two group moderators led an interactive discussion to: reword those examples that were unclear, add new ones, remove or merge those overlapping, and separate individual from multiple component examples. After all barriers were shared and refined, participants identified, shared, and refined examples of barrier-specific facilitators and general facilitators. Finally, participants independently rated importance of barriers and likelihood of using facilitators from 0-10, and indicated whether they had tried facilitators. Following NGT sessions, examples across sessions were merged into single items to eliminate overlap; items were edited based on feedback from investigators, patient advisors, and clinicians; and items were consolidated into four categories using content analysis.

**Results:** Nine sessions were conducted (n=41 total participants) and initially generated 181 barriers, 457 barrier-specific facilitators, and 20 general facilitators. The number of consolidated barriers (and barrier-specific facilitators in parentheses) for each category were: 14 (61) for health and medical; 4 (23) for social and personal; 1 (3) for time, work, and lifestyle; and 1 (4) for environmental. There were also 12 consolidated general facilitators. Among consolidated items, the number of barrier-specific facilitators per barrier ranged from 2-10. There were 15 barriers, 69 barrier-specific facilitators, and 9 general facilitators for which at least 1/3 of participants’ ratings were > 7.

**Conclusions:** The most common barriers were related to health and medical aspects of SSc. Both barrier-specific and general facilitators of physical activity were identified. The list of barriers and facilitators will be used to survey a large international SSc cohort to inform the development of an online SSc-specific physical activity promotion program.

## P.189

## A POSTER TO EXPLAIN TO THE PATIENT THE MAIN SIGNS AND SYMPTOMS OF EARLY SSC: RESULTS FROM A GP CLINIC

T. Pucci, L. Salvi, L. Paganelli, C. Fantauzzo, G. Gambardella, S. Bellando Randone, M. Matucci- Cerinic, S. Guiducci

*Dep Experimental and Clinical Medicine, Division of Rheumatology, University of Florence, Florence, Italy*

**Introduction:** To date, the largest effort of the community have been dedicated to the early recognition of SSc, in order to start a precocious treatment and avoid disease evolution and patient’s disability. The nurse is increasingly engaged in the search for new materials and new educational methods to be effective in a health prevention and promotion plan

The aim of this study was to create a poster that could schematically present to the patient the early signs and symptoms of SSc. The poster was displayed in general practitioners (GP) clinics to allow patients to read it and understand which are the signs and symptoms of the early onset of SSc

**Material and Methods:** A questionnaire was administered to 200 patients present in the GP’s waiting room, after reading the poster. The patients’ understanding of signs and symptoms, and the clarity of the poster were investigated. Moreover, 20 GPs were interviewed about their knowledge of SSc disease and the usefulness of the poster as a prevention tool

**Results:** In 200 patients the poster read in an average duration of 15 minutes, allowed the patient to memorize the main signs and symptoms of SSc, From the data analyzed, 96% of the patients considered the poster clear and effective in presenting SSc. The SSc disease was unknown in 81% of the sample.

**Conclusions:** The investigation shows that the poster presenting SSc may efficiently inform the patient and GPs about the disease, alerting about the signs and symptoms of an early SSc. The investigation should be extended to a wider population and to achieve a greater involvement of the GPs. The role of the nurses in health education and in the prevention and promotion of health, may allow the patient to approach the screening and control programs of his health status.

**Figure:**
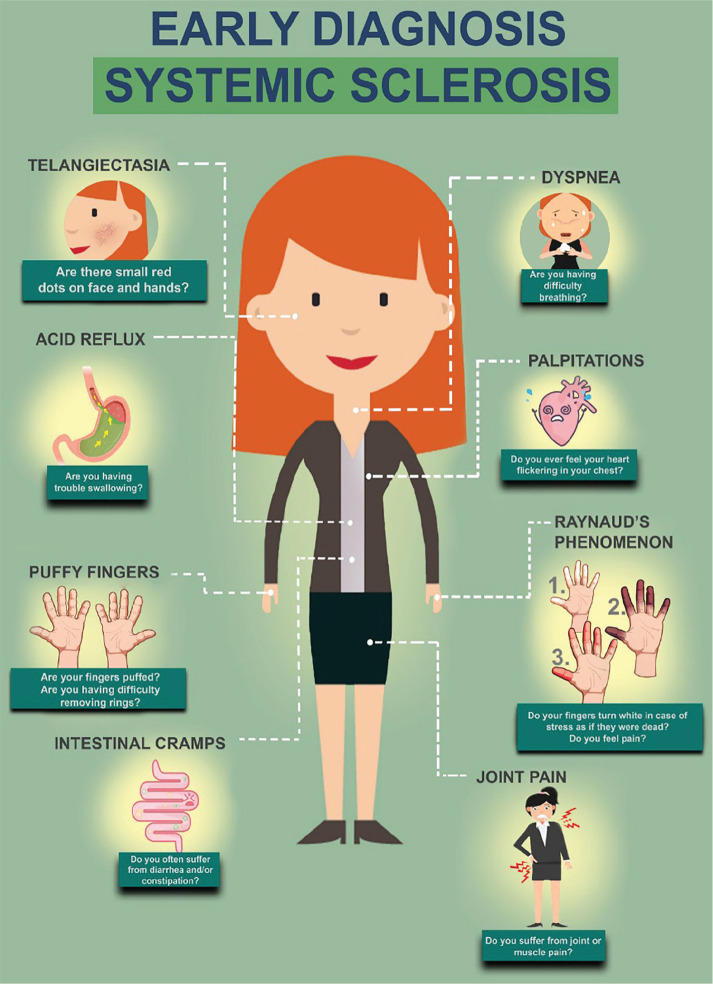


## P.190

## THE NURSE-PATIENT RELATIONSHIP: ANIMAL ASSISTED INTERVENTION (AAI) AS A FACILITATOR

G. Fiori^1^, T. Pucci^1^, M. Zolferino^1^, E. Corsi^1^, A. Bartoli^1^, D. Buttram^2^, M. Matucci Cerinic^1^, S. Guiducci^1^

^1^*Dep of Experimental and Clinical Medicine, Division of Rheumatology, University of Florence, Florence, Italy*, ^2^*Natura Animale, Florence, Italy*

**Introduction:** AAI previously known as Pet therapy, through human-animal interaction and relationship, may complement and support traditional therapies. AAI can be implemented with patients affected by Systemic Sclerosis (SSc), improving health and quality of life from behavioral and psychosocial points of view. The nurse-patient relationship, based on trust and empathy, represents a crucial link for care of patient, but may be difficult to establish. Thanks to comfort and relaxation, which can be induced during interaction with AAI interventions, an empathetic relationship can be created and social relations amongst SSc patients can be facilitated. The animal often acts as an ice breaker, helping the patient to lower emotional barriers and encouraging him to face new treatments. This work aims to understand if AAI, on days of medical treatments, facilitates the nurse-patient connection, improving normal therapeutic activities, offering the possibility of well-being when healing is almost impossible.

**Material and Methods:** The study was conducted through the administration of a questionnaire, to investigate the knowledge of AAI and changes in nurse-patient relationship and disease vision through AAI with a dog. The sample was represented by a group of 20 SSc patients (pts) and a group of 10 nurses who participated in the AAI projects carried out at the University of Florence

**Results:** AAI promoted an improvement of psychophysiological of SSc pts. The answers of questionnaire showed that AAI facilitated emotional openness and communication of SSc pts with nurses, thus laying the background for development of a more stable relationship. There was a psychological benefit for pts as the meetings with animals and their handlers in the AAI setting, promoted a feeling of integration and offered opportunities for social exchanges. Both nurses and pts answered that their mutual relationships have greatly benefited from presence of animal, due to a sense of spontaneity which is usually absent in the presence of people unknown to them. This spontaneity allowed them to open up, to tell about themselves, starting a dialogue. Finally, the project, especially the presence of the dog, transformed the often boring and painful activities into “something special” and were more willingly carried out both by the patients and the staff.

**Conclusions:** AAI was a valuable aid for the establishment of a good nurse and patient relationship, facilitating emotional openness and deeper knowledge. This results in an improvement in the quality of care and the efficacy of the treatment and gives staff a chance to better implement the nursing process.

## P.191

## DISEASE ACTIVITY INDICES IN SYSTEMIC SCLEROSIS- OLD OR NEW?

L. Groseanu^1^, S. Petrescu^1,2^, A. Balanescu^1,2^, D. Opris-Belinski^1,2^, F. Berghea^1,2^, V. Bojinca^1,2^, A. Borangiu^1,2^, I. Saulescu^1,2^, D. Mazilu^1,2^, S. Daia-Iliescu^1,2^, C. Constantinescu^1,2^, M.-M. Negru^1,2^, M. Abobului^1,2^, C. Cobilinschi^1,2^, R. Ionescu^1,2^

^1^*Sf Maria Clinical Hospital, Bucharest, Romania*, ^2^*Carol Davila University of Medicine and Pharmacy, Bucharest, Romania*

**Introduction:** Currently there is no fully validated index for assessing disease activity in patients with systemic sclerosis (SSc) in daily practice. Our objective was to estimate the effect of disease activity as measured by 4 disease activity indices on the risk of subsequent organ damage in a EUSTAR center cohort.

**Material and Methods:** We performed a longitudinal observational study on 118 SSc patients, between 2000 and 2017. Patients were assessed according to MEDS evaluation sheets. European Systemic sclerosis study group disease activity index (EScSG DAI), revised EUSTAR disease activity index (r-EUSTAR DAI), 12 point activity index proposed by Minier (12point DAI) were calculated; the CRISS (The Combined Response Index for Systemic Sclerosis ) only for patients included after 2016.

**Results:** 91 patients were selected, 50,58(12,71) years old at diagnosis, 53.39% diffuse subset.

EscSG DAI correlated with FVC( p<0.01), DLCO (p<0.001), digital ulcers( p=0.05), presence of lung fibrosis(r p<0.0), muscle atrophy,( p<0.001), cutaneous subset (p<0.01), capillaroscopic pattern (p<0.01). r-EUSTAR DAI correlated with FVC (p<0.001), DLCO (p<0.001), diffuse subset (p<0.01), contractures(p=0.001), muscle weakness and muscle atrophy (p<0.001), scleroderma renal crisis (p=0.02), capillaroscopic pattern (p<0.001) and the value of sPAP(p<0.001). 12 point DAI correlated with FVC, DLCO, capillaroscopic scoring and sPAP (p<0.001).

EscSG predicted well lung fibrosis (AUC=0.79,p<0.001), digital ulcers (AUC=0.66,p<0.001), gastric involvement (AUC=0.73, p<0.01)and scleroderma renal crisis (AUC=0.9, p=0.01). R-EUSTAR index also lung fibrosis (AUC=0.76,p<0.001), digital ulcers( AUC=0.82, p<0.01) and scleroderma renal crisis(AUC=0.84 p=0.04). 12point DAI was a good predictor for lung fibrosis (AUC=0.74, p<0.01), digital ulcers (AUC=0.78, p=0.05), gastric involvement (AUC=0.76,p=0.01).

In the Liniar regression analyises, lung fibrosis (beta= 0.5,95% CI=1,21-2,58, p<0.01), muscle atrophy (beta=1.49, 95% CI=1.02-2.19, p=0.03) and rhtym and conduction disturbancies (beta=1.98, 95CI=1.31-2.99, p<0.001) were independent predictors for disease activity evaluated by EScSG. For 2-EUSTAR DAI none of the evaluated parameters proved to independently contribuite to disease activity. For 12point DAI items independently contributing to disease activity were gastric involvement (beta=2.46, 95%CI=1.19-5.09, p=0.01) and muscle atrophy (beta=2.05, 95%CI=1.03-4.08, p=0.03). None of the patients had a CRISS score with a probability of improvement more then 0.6.

**Conclusions:** We identified a number of composite and organ-specific indices in SSc, developed to quantify disease activity, severity, and response in clinical trials. Especially ESsSG and r-EUSTAR DAI could predict severe organ involvement as lung fibrosis, digital ulcers and scleroderma renal crisis, but not pulmonary hypertension.However, none of the indices was developed to exclusively quantify organ damage. There is a need to develop and validate a disease damage index in SSc.

## P.192

## THE INFLUENCE OF BIOFEEDBACK COURSE ON THE QUALITY OF LIFE OF PATIENTS WITH SYSTEMIC SCLEROSIS

R. Grekhoff

*Zborovsky’ Research Institute for clinical and experimental rheumatology, Volgograd, Russia*

**Introduction:** A lot of methods of non-medicinal therapy were proposed for systemic sclerosis (SS), but desirable success was not achieved. It is well known that active participation of patient in treatment can improve efficiency of the therapy. One of promising method is biofeedback, referred on active participation of patient in the therapy.

Our aim was to improve the efficiency of complex therapy of SS patients by means of biofeedback therapy.

**Material and Methods:** We observed 90 SS patients of middle age equal to 38,19±12,1 years, and average duration of illness 11,2±3,4 years. The minimum degree of activity of disease (I) has been diagnosed for 47 (52,2%), medium (II) degree - in 38 (42,2%) and high (III) degree - in 5 (5,6%) patients. A chronic course of the disease was diagnosed in 39 (43,3%), subacute - in 48 (53,3%), and acute - in 3 patients (3,3%). The stage of initial changes has been diagnosed in 37 (41%), a stage of generalization of process - in 50 (55,5%), a late (terminal) stage – in 3 patients (3,5%). Depending on degree of skin changes limited SS has been diagnosed in 66 (73,3%), and diffuse SS form - in 24 (26,7%) patients.

SS patients have been divided on two groups randomly: the basic (n=60) and control (n=30). Patients of basic group received 12-14 sessions of multimodal (temperature and EEG) biofeedback training using Reacor rehabilitation complex.

Clinical assessment of biofeedback efficiency was assessed by dynamics of following clinical and laboratory indices, pain by VAS, joint count, number of swelling joints, swallow index, ESR, antibodies to Scl-70, SF-36 questionnaire was applied for quality of life evaluation.

**Results:** It was founded that indices reflecting a physical component of quality of life - physical functioning (p<0,001), role physical functioning (p<0,01), somatic pain (p<0,001), and also overall health (p<0,001), social (p<0,05) and role emotional functioning (p<0,01) were reliable raised in SS patients of basic group. The results of assessment of physical and psychological health are similar in group of SS patients receiving conventional therapy, but changes of smaller quantity of indices were reliable.

**Conclusions:** Results of our researches are the evidence of considerable efficiency of biofeedback training in complex therapy of SS patients and its positive influence on quality of patients’ lives along with clinical and laboratory indices of the disease. The use of biofeedback will allow improving SS patients’ quality of life.

## P.193

## INFLUENCE OF PATIENT REPORTED ‘‘ARTHRITIS ACTIVITY’’ IN DETERMINING SHAQ, HAQ-DI AND COCHIN SCORES IN SYSTEMIC SCLEROSIS

K. Gjeloshi^1^, F. Danzo^1^, G. Abignano^2,3,4^, A.-M. Dean^3,4^, F. Masini^1^, G. Cuomo^1^, F. Del Galdo^3,4^

^1^*Complex Operative Unit (UOC) Internal Medicine Department, University of Campania ‘‘Luigi Vanvitelli’’, Napoli, Italy*, ^2^*Rheumatology Institute of Lucania (IReL), Rheumatology Department of Lucania, San Carlo Hospital, Potenza, Italy*, ^3^*Leeds Institute of Rheumatic and Musculoskeletal Medicine, University of Leeds, Leeds, United Kingdom*, ^4^*NIHR Leeds Biomedical Research Centre, Leeds Teaching Hospitals NHS Trust, Leeds, United Kingdom*

**Introduction:** Arthritic involvement is a common manifestation in systemic sclerosis (SSc). The aim of this study is to assess the weight of patient reported ‘‘arthritis activity’’ in determining Patient Reported Outcome Measures (PROMs) in an observational cohort of SSc patients.

**Material and Methods:** We conducted a retrospective study of 330 clinic episodes from 121 unselected patients diagnosed with SSc according to EULAR/ACR 2013 classification criteria, in annual follow-up (for a total of 165 patients/year) with Pulmonary Function Tests (PFTs), Health Assessment Questionnaire - Disability Index (HAQ-DI), Scleroderma Health Assessment Questionnaire (sHAQ) and Cochin Hand Function Score (CHFS). Hand disability index was assessed by CHFS and global disability index was assessed by HAQ and sHAQ. Patient reported arthritis activity was assessed by Visual Analogical Scale for Arthritis Activity (VAS3). Based on the median of VAS3, patients were classified in two groups and the evaluation of global and hand disability index was performed for each group. Furthermore, we assessed the correlation between the change of VAS3 and the modification of disability scores (δ HAQ, δ SHAQ, δ CHFS) over 12 months of follow-up. Following analysis of distribution, Spearman or Pearson Test were used to determine correlation coefficients, as appropriate (Prism 7).

**Results:** The median disease duration was 5 years (IQR 3-10). The median of VAS3 was 35 (IQR 2 - 66). In patients with VAS3 <35 and VAS >=35 the HAQ-DI medians were 0.625 (IQR 0.25 - 1.5) and 1.75 (IQR 1.125-2.25) respectively, (p<0.0001); the sHAQ medians were 0.628 (IQR 0.255 - 1.114) and 1.701 (IQR 1.234-2.059), respectively, (p<0.0001); and the CHFS medians were 4 (IQR 0 – 19) and 28 (IQR 10 – 46) respectively, (p<0.0001). A significant correlation was observed between VAS3 and HAQ (r= 0.463, p<0.0001), SHAQ (r=0.651, p<0.0001), CHFS (r=0.497, p<0.0001); between δ VAS3 and δ SHAQ (r=0.493, p<0.0001).

**Conclusions:** This analysis of a monocentric non-selected population supports the key role of joint involvement in determining global patient reported functional and hand disability in SSc. Severity of musculoskeletal involvement should be carefully considered when interpreting PROs in patients with SSc.

## P.194

## A TIME SERIES ANALYSIS OF AIR QUALITY AND THE NUMBERS OF HOSPITALIZATION IN THAI SYSTEMIC SCLEROSIS PATIENTS

C. Foocharoen, U. Peansukwech, P. Pongkulkiat, A. Mahakkanukrauh, S. Suwannaroj

*Khon Kaen University, Khon Kaen, Thailand*

**Introduction:** Occupational and environmental association with SSc has been revealed, however; the association between air quality and hospitalization in systemic sclerosis (SSc) has not been reported. Aim of the study was to define the association between air quality and numbers of hospitalization in Thai SSc patients.

**Material and Methods:** A time stratified case crossover design was performed using a nationwide national database of hospitalized patients covered by the National Health Security Office during 2014-2018. Data extracted included all patients over 15 having a primary diagnosis of SSc (ICD-10:M34). Spatial resources used Thailand’s map information based on GPS-related satellites. Air quality included temperature, humidity, ozone, carbon dioxide, nitric oxide, organic carbon, black carbon and dust were assessed from NASA satellites MERRA-2 Model M2TMNXFLX v5.12.4. Time series regression was used for analysing associations between time series of air quality and numbers of hospitalization.

**Results:** Of total 2,480 SSc patients with 3,684 admissions were included. Most (64.3%) were female. By time series regression analysis, temperature, humidity, ozone and carbon dioxide were associated with hospitalization in Thai SSc patients with incidence rate ratio of 0.90 (95%CI 0.86-0.94), 0.000021 (95%CI 0.000024-0.0019), 1.027 (95%CI 1.021-1.033) and 1.005 (95%CI 1.001-1.008), respectively.

**Conclusions:** Air quality affects the hospitalization in Thai SSc. High temperature and high humidity decreased the incidence rate ratio of hospitalization while high ozone and high carbon dioxide increased the rate of hospitalization.

## P.195

## EFFECT OF SEASON ON THE CLINICAL OUTCOMES OF THAI SYSTEMIC SCLEROSIS: AN ANALYSIS OF A NATIONWIDE THAILAND HEALTHCARE DATABASE

C. Foocharoen, U. Peansukwech, P. Pongkulkiat, A. Mahakkanukrauh, S. Suwannaroj

*Khon Kaen University, Khon Kaen, Thailand*

**Introduction:** Cold can induce vasospasms as well as Raynaud’s phenomenon, and may aggravate or trigger vascular injury in systemic sclerosis (SSc). A few studies have investigated the effect of cold on the outcomes of SSc. Our aim was to determine the rate of admission, the mortality rate, and the causes of death in SSc patients after stratifying by season.

**Material and Methods:** A cross-sectional analysis was performed using a nationwide national database of hospitalized patients covered by the National Health Security Office (NHSO) for the five years between 2014 and 2018. Data extracted from the pooled hospitalization database included all patients over 15 having a primary diagnosis of SSc (ICD-10: M34). The seasons were stratified into 3 subgroups: summer, rainy, and winter according to the Thai Meteorological Department.

**Results:** Included were 2,480 SSc patients with 3,684 admissions seen between 2014 and 2018. Most (1,595 cases, 64.3%) were female. The respective mean age and median length of stay was 56.9±12.3 years (range, 15-91) and 3 days (IQR, 2-6). The admission rate was highest during the rainy season (1,574 visits, 42.7%), followed by winter (1,183 visits, 32.1%) then summer (927 visits, 25.2%). There was no statistically significant difference between seasons whether by sex, age at admission, number of admissions, length of stay, and hospital cost (p=0.32, 0.42, 0.47, 0.19 and 0.17, respectively). Of the total 13,180 person-days, 1,660 SSc patients died for a mortality rate of 12.1 per 100 person-days. The median time of death was 5 days after admission. The proportion of SSc patients who died in winter was significantly higher than in summer or the rainy season with p=0.04. SSc-related death was the primary cause of death in all seasons. Pulmonary involvement in SSc was the common causes of death in the summer, which is greater than in the other seasons (p=0.004); whereas death due to cardiac involvement in SSc was more common during the rainy season and winter than during the summer (p=0.04)

**Conclusions:** The admission rate among Thai SSc patients was greatest in the rainy season, while the death rate was highest durning the winter. The most common causes of death were SSc-related notwithstanding the season, particularly cardiopulmonary involvement.

## P.196

## FATIGUE IN A SERIES OF CANADIAN PATIENTS WITH SCLERODERMA

A. Fernandez-Codina, M. Kazem, Z. Jiang, J. Pope

*Rheumatology Division, Western Ontario University, London, Canada*

**Introduction:** Systemic Sclerosis (SSc) is systemic autoimmune disease that can involve multiple organs. Moreover, fatigue is a common and unsolved problem in SSc patients, which can be linked to organ damage and other comorbidities. Vitamin D (VD) is considered to have an immunomodulator role in lupus, and high doses supplements have been reported to improve fatigue.

**Material and Methods:** We did a cross-sectional study in a group of SSc patients at our institution in Canada and participating in a registry. The objectives were to assess the level of fatigue, scleroderma involvement, comorbidities, VD levels and treatment status.

**Results:** Data from 69 patients were analyzed. Fifty-six (82%) were women with a median age of 64 years, and all but 3 were Caucasian. Sixty-four (94.1%) met the 2013 ACR/EULAR classification criteria for SSc and the distribution by phenotypes was 69.6% limited cutaneous SSc, 26% diffuse, and 4% sine scleroderma. Fifteen percent had overlap syndromes. One third had sicca symptoms, 7% fibromyalgia and 19% depression. In terms of scleroderma-related ongoing organ involvement: 54% had gastroesophageal reflux, 30% interstitial lung disease, 15% required esophageal dilatations, 9% had digital ulcers, 7% small bowel bacterial overgrowth and arthritis, 4% gastric antral vascular ectasias, 2% cardiac involvement and pulmonary arterial hypertension, and 1% scleroderma renal crisis. Median modified Rodnan skin score was 4, Medsger’s severity scale 5, Charlson comorbidity index 4.

The outcome measures median results were: physician scleroderma severity visual analogue scale (VAS 0-100) 14, scleroderma severity VAS according to patients 30, health assessment questionnaire HAQ-II (0-3) 0.7, fatigue VAS (0-10) 4.5, functional assessment of chronic illness therapy-4 (FACIT, 0-52) 31, pain VAS (0-10) 4 and PROMIS self-efficacy for managing symptoms short form (22.7-63.9) 48.9.

The median 25-hydroxycalciferol serum value was 85 nmol/L (range 244, normal values 75-250). Twenty-four (35%) patients had 25-hydroxycalciferol < 75nmol/L and 28 (41%) took a median of 1000mg of vitamin D per day. Patients on vitamin D were evenly distributed between patients with/without 25-hydroxycalciferol deficiency. The were no statistically significant differences in the outcome measure scales (including fatigue VAS and FACIT) analyzing the median values between patients with and without 25-hydroxycalciferol < 75nmol/L or patients with/without VD supplements.

**Conclusions:** In our study, patients with scleroderma had fatigue levels similar to other studies. Neither 25-hydroxycalciferol levels, nor treatment with low doses of VD showed differences in fatigue scores. A randomized clinical trial of high doses of VD versus usual supplementation might clarify its role in fatigue.

## P.197

## A RETROSPECTIVE ANALYSIS OF CHRONIC USE OF INTRAVENOUS PROSTACYCLIN ANALOGUES IN THE TREATMENT OF RAYNAUD’S PHENOMENON: LIMITED LONG-TERM EFFICACY

T. Elling^1^, A.E. Abdulle^2^, A.M. Van Roon^2^, A.J. Stel^3^, D.J. Mulder^2^

^1^*Department of Haematology; University Medical Center Groningen, Groningen, The Netherlands*, ^2^*Department of Internal Medicine, division Vascular Medicine; University Medical Center Groningen, Groningen, The Netherlands*, ^3^*Department of Rheumatology and Clinical Immunology; University Medical Center Groningen, Groningen, The Netherlands*

**Introduction:** Intravenous prostacyclin analogues (PGA) are used for the treatment of Raynaud’s Phenomenon (RP) in patients who do not respond to oral drug therapies. In some patients, PGA are used chronically. However, data evaluating the effects of PGA on RP in the long-term use are limited. Nonetheless, the high costs, frequent hospitalization and possible side-effects of PGA make a structural evaluation of this treatment necessary. Therefore, this study evaluates the evidence of various treatment regimens of PGA in literature and the effect of intravenous PGA on RP symptoms, health-related quality of life (HRQoL) and blood perfusion in chronically treated patients.

**Material and Methods:** This study included 19 patients chronically treated with PGA for severe RP (age 55.0 (43.0-62.0); female, n=17; primary RP, n=8; secondary RP, n=11 (systemic sclerosis, n=6)) without recent digital ulcers, compared to 19 patients with severe RP without PGA treatment (age 49.0 (44.0-57.0); female, n=10; primary RP, n=4; secondary RP, n=15 (systemic sclerosis, n=14)) and 19 healthy controls (age 53.0 (44.0-63.0); female, n=13). The SF-36 questionnaire was used to analyze HRQoL. In a subset (n=10) RP symptoms, defined as frequency, duration and severity (Raynaud Condition Score) of RP attacks were reported in daily diaries one week before and after PGA treatment. Blood perfusion was assessed with photoelectric plethysmography on the fingers during a standardized cooling experiment, directly before and after PGA infusion (n=10). Mean ischemic time was defined as the time between loss and gain of perfusion for the five fingers.

**Results:** A significantly impaired HRQoL was observed in almost all domains, compared to healthy controls (p<0.05) and on ‘social functioning’ and ‘general health’ compared to patients with RP without PGA (p<0.05). In the substudy, no differences were seen in patient reported RP symptoms and mean ischemic time during cooling, before and after PGA infusion.

**Conclusions:** These results suggest that the effect of long-term use of intravenous PGA on RP symptoms and digital blood flow are very limited. Therefore we advocate for a careful evaluation of patients chronically treated with PGA. The results were partly in line with previous literature, in which minimal to significant improvement in RP symptoms were seen in patients with different treatment regimens in the long-term use.

## P.198

## SYSTEMIC SCLEROSIS IN PATIENTS AGED LESS AND MORE THAN 60 YEARS - CLINICAL CHARACTERISTIC

M. Dryglewska, E. Wielosz, A. Lipska, M. Majdan

*Department of Rheumatology and Connective Tissue Diseases Medical University of Lublin, Lublin, Poland*

**Introduction:** According to literature, the course of systemic sclerosis (SSc) in adults and elderly patients is different. The aim of our study was to asses whether there were differences in clinical and serological symptoms of disease between the patients with SSc before and over 60 years of age.

**Material and Methods:** The study included 157 (119 female and 38-male) patients with SSc treated in the Department of Rheumatology and Connective Tissue Diseases. Patients fulfilled the American College of Rheumatology (ACR)/ European League against Rheumatism (EULAR) classification criteria of SSc; 69 had diffuse cutaneous SSc-dcSSc and 88 limited SSc –lcSSc. The mean age in the study group was 58.1 ± 13.15 years (range 23-81) while the duration of disease was 5.15 ±5.41 years (range 0-23). According to age, the SSc patients were divided into two groups: I- SSc before 60 – 82 patients, and II- SSc over 60- 75 patients.The two study groups were assessed according to the SSc subtype and incidence of internal organ involvement. The statistical data analysis was performed using Statistica v13.3.

**Results:** In our study, statistically significant differences were found in the prevalence of gastrointestinal tract involvement (p=0.04) and renal involvement (p=0.0014), which were significantly higher in the group with SSc over 60, compared to the group with SSc before 60. Additionally, diffuse cutaneous (dc) SSc was observed less commonly in the over 60 group than in the before 60 group (p=0.03) Moreover, in the group with SSc over 60 pulmonary arterial hypertension was more common but not statistically significant, as compared to the other group (25.3% vs14.6%). Otherwise, mortality was more common in the group with SSc before 60, as compared to the other group (19.5%vs 10.7%). The incidences of other clinical symptoms such as, interstitial lung disease, calcinosis, contractures, myalgia, scleroderma renal crisis, arthralgias, arthritis, neoplastic diseases, or overlap syndrome were found to be comparable in both study groups.

**Conclusions:**

1. The course of systemic sclerosis in patients over 60 years of age is markedly different from that in patients before 60 years of age.
2. The incidence of diffuse cutaneous systemic sclerosis was lower in the group over 60 years, compared to that before 60 years.
3. The incidence of gastrointestinal tract involvement and renal involvement was higher in the group of patients over 60, compared to those before 60 years of age.

## P.199

## PATHWAY TO SCLERODERMA: CONCERNING PATIENTS’ EXPERIENCES DURING THE DIAGNOSTIC PROCESS

P. Fennell^1^, L. Shapiro^2^, N. Dorr^3^, R. Lukasiewicz^4^, F. Houser^3^, M. Taylor^3^

^1^*Albany Health Management Associates, Steffens Scleroderma Foundation, Albany, USA*, ^2^*Albany Medical College, Director of Scleroderma Center, Community Care Rheumatology, Albany, USA*, ^3^*The College of Saint Rose - Department of Psychology, Albany, USA*, ^4^*Steffens Scleroderma Foundation, Albany, USA*

**Introduction:** Receiving a diagnosis of systemic sclerosis (SSc) can be straightforward process for some, whereas others have a circuitous route. To explore these differences, we retrospectively examined the process patients with SSc experienced from the point of first symptoms through diagnosis.

**Material and Methods:** Patients with SSc were approached to participate by their rheumatologist (specializing in scleroderma) during an office visit. Sixty-four patients (42% diffuse, 45% limited, 13% unknown) completed open-ended questions (e.g., what was your experience when you first felt unwell and when you were diagnosed with scleroderma). Two researchers independently categorized responses; discrepancies were resolved by discussion. One researcher reviewed patients’ records.

**Results:** Regarding length of time from first symptoms to SSc diagnosis, 48% of patients reported being diagnosed within one year, 31% within 1-4 years, and 21% were diagnosed more than 4 years after the first symptoms appeared.

Overall, 75% of patients reported an initial symptom(s) involving their hands (53% Raynaud’s). The 25% without symptoms involving hands commonly reported pain (50%), shortness of breath (31%), and gastrointestinal issues (19%).

Patients reported seeing up to seven health care providers (HCP) during the diagnostic process (M=2.64). When patients first noticed symptoms, 33% saw their primary care physician (PCP) and/or a rheumatologist (27%), and 19% saw their PCP subsequently. Thirty-one percent of patients had a HCP suspect scleroderma before it was diagnosed (13% of HCPs were a rheumatologist who referred them to a scleroderma specialist, 6% were their PCP). One-third of patients were diagnosed with another condition before receiving the scleroderma diagnosis. Some of these were a mis-diagnosis (e.g., carpel tunnel syndrome, lupus); others likely did have scleroderma as the underlying, but unrecognized, cause (e.g., arthritis, pulmonary arterial hypertension). Fifty percent of patients reported a scleroderma diagnosis from a scleroderma specialist, 38% from a different rheumatologist, and 3% from their PCP.

**Conclusions:** With 1 in 5 of patients being undiagnosed for more than four years, and only about 30% of patients having a HCP suspect a scleroderma diagnosis, educational efforts should make scleroderma a more salient diagnosis for HCPs. Three-quarters of patients reported initial symptoms involving their hands. About half of patients saw their PCP at some point in the diagnostic process, but only 9% reported their PCP suspected scleroderma.

Taken together, these data suggest educational efforts targeting PCPs and specialists to consider scleroderma more readily as a diagnostic possibility. Scleroderma should be, but often is not, included in differential diagnosis of persistent hand complaints.

## P.200

## THE DIAGNOSIS OF SCLERODERMA AS A TRAUMATIC EXPERIENCE: PATIENTS’ REACTIONS TO THE DIAGNOSTIC PROCESS

P. Fennell^1^, L. Shapiro^2^, N. Dorr^3^, R. Lukasiewicz^4^, F. Houser^3^, M. Taylor^3^

^1^*Albany Health Management Associates, Steffens Scleroderma Foundation, Albany, USA*, ^2^*Albany Medical College, Director of Scleroderma Center, Community Care Rheumatology, Albany, USA*, ^3^*The College of Saint Rose - Department of Psychology, Albany, USA*, ^4^*Steffens Scleroderma Foundation, Albany, USA*

**Introduction:** Both the process of being diagnosed with a chronic illness and the illness itself can be traumatic experiences for patients. Diagnosis often does not happen quickly and learning that one has a potentially life-threatening disease for which there is no cure can create negative reactions and a fear of the unknown. This is a growing issue due to the increased prevalence of chronic illness worldwide. The current study examines the diagnosis of a chronic illness, systemic sclerosis (SSc), as a traumatic experience.

**Material and Methods:** Patients with SSc were approached to participate by their rheumatologist (specializing in scleroderma) during an office visit. Sixty-four patients (42% diffuse, 45% limited, 13% unknown) completed open-ended questions (e.g., what messages about scleroderma have you received, how did you react to the diagnosis). Two researchers independently categorized responses; discrepancies were resolved by discussion. One researcher reviewed patients’ records.

**Results:** Seventeen percent of patients received potentially traumatic messages from a health care provider (HCP) during the diagnostic process. For example, one patient reported learning there was no treatment for scleroderma and that it would continue to get worse, another patient was told she/he had a 5+ year life expectancy, one reported being told she/he was dying (before scleroderma was diagnosed), and another reported physicians involved in the diagnostic process were dismissive of independent and seemingly unrelated symptoms.

Fear was the most common patient reaction (78%). For example, 48% of patients reported being afraid of the future and 30% reported being afraid of dying. Twenty percent of patients reported feeling upset about not having a diagnosis (before scleroderma was diagnosed), 17% were concerned because of the disease progression they witnessed in others, and 14% reported anticipatory grief (e.g., grieving potential future loss of hand functioning). Thirty percent of patients learned about scleroderma on the internet and many found this fear-provoking. Sixty-eight percent of patients reported their rheumatologist helped to reduce their concerns.

**Conclusions:** Diagnosing and treating chronic illness can be difficult for both patients and practitioners; all involved face ambiguity regarding the course illness can take. These data underscore the importance of physicians attending to the way information regarding scleroderma is conveyed to patients. Given the diagnostic process for scleroderma can be lengthy, patients may be feeling afraid and/or traumatized for years. HCP working with scleroderma patients should address patients’ emotions and the causes of them (e.g., internet, statement by HCP) and know that their conversations with patients can make a difference.

## P.201

## CLINICAL SIGNIFICANCE OF COGNITIVE IMPAIRMENT AND MALNUTRITION IN PATIENTS WITH SYSTEMIC SCLEROSIS

L. Manfredi, D. Benfaremo, G. Silvi, A. Gabrielli

*Clinica Medica, Dipartimento di Scienze Cliniche e Molecolari, Università Politecnica delle Marche, Ancona, Italy*

**Introduction:** Previous studies reported a high prevalence of cognitive dysfunction in systemic sclerosis (SSc). Cognitive impairment was estimated to involve 60% to 80% of SSC patients and to be correlated with older age, disease severity, diffuse cutaneous subset and poor quality of life. The aim of our study was to evaluate the association between cognitive impairment, nutritional status and the quality of life of SSc patients.

**Material and Methods:** Sixty-eight consecutive SSc patients followed at our Institution were evaluated for cognitive impairment using the validated Italian version of the Montreal Cognitive Assessment (MoCA). Scores <26 were considered abnormal. We also assessed other domains and quality of life measures such as UCLA SCTC GIT 2.0 for gastrointestinal involvement, BDI-II and PHQ-9 for anxiety and depression, EAT-10 for dysphagia symptoms, SHAQ and SF-36 for function and quality of life (QoL). The risk and the presence of overt malnutrition were assessed using the MUST questionnaire and the GLIM criteria, respectively. Clinical and demographic parameters such as age, sex, BMI, disease subset, organ involvement, autoantibody profile and modified Rodnan Skin Score were also recorded for each patient. Data were analysed by Student t-test or chi-square test and regression analyses were used to assess the association between variables.

**Results:** A total of 68 SSc patients [47 (69.1%) limited SSc (lSSc) and 21 (30.9%) with diffuse SSc (dSSc), 59 female; mean age 60.2 (±13.4) years; mean disease duration 9 (±8.2) years; mean mRSS 8.1 (±7.6)] were included in the study.

Cognitive impairment was identified in 30 (44.1%) SSc patients; the mean MoCA score was 24.7 (±4.3). According to GLIM criteria, 16 (23.5%) patients were malnourished. Compared to patients with a MoCA>26, patients with cognitive impairment were older (p<0.001), had more comorbidities (p<0.0001) and a worse QoL as assessed by the physical and general health domains of the Sf-36 (p<0.05). Malnourished patients were significantly more dysphagic (p<0.01) and had a worse HAQ (p<0.01) compared to well-fed patients. On regression analyses, cognitive impairment was related to increasing age (OR 1.08, 95%CI 1.03 to 1.14, p=0.001), but not to malnutrition, disease subset or symptoms. Malnutrition was associated with dysphagia (OR 1.10, 95%CI 1.01 to 1.20, p=0.01) and HAQ score (OR 2.69, 95%CI 1.24 to 5.82, p=0.01), but was not predicted by cognitive impairment.

**Conclusions:** Cognitive dysfunction is frequently observed in SSc and mostly associated with increasing age and number of comorbidities. Malnutrition and cognitive impairment are both associated to QoL but seem to be unrelated.

## P.202

## SCLERODERMA PROGRESSION AND PREGNANCY: A HOPEFUL MESSAGE FROM THE CANADIAN SCLERODERMA RESEARCH GROUP

S. Deshauer^1^, M. Junek^1^, K.B. Beattie^2^, M. Larché^2^

^1^*McMaster University - Department of Internal Medicine, Hamilton, Canada*, ^2^*McMaster University - Department of Rheumatology, Hamilton, Canada*

**Introduction:** There are limited data available to guide clinicians and women with SSc in making informed decisions about pregnancy and their disease post-partum. While peripartum maternal and fetal complications have been reported, little is known about disease activity after pregnancy. We explored the trajectory of disease activity in women who experienced a pregnancy after SSc diagnosis and compared this with women who were nulliparous.

**Material and Methods:** We conducted a retrospective cohort study of the Canadian Scleroderma Research Group (CSRG). We identified two groups of women: nulliparous (NP) and those who experienced one or more pregnancies after the diagnosis (PAD) of SSc. Patients with underlying cardiac, lung or renal disease unrelated to SSc were excluded. Baseline characteristics of the two cohorts were compared using t-tests. Generalized estimating equations (GEEs) determined if the cohorts differed in the following SSc outcomes: force vital capacity, diffusing capacity of the lungs for carbon monoxide, right ventricular systolic pressure, glomerular filtration rate, physician global assessment of activity and physician global assessment of severity. Each analysis was controlled for age, time since SSc diagnosis, SSc subtype (limited vs. diffuse), and smoking status (pulmonary outcomes only).

**Results:** At their respective time of entry into the CSRG database, 153 women were nulliparous and 45 women had a pregnancy after their diagnosis of scleroderma. The NP group was younger at SSc diagnosis (p<0.001), had a shorter disease duration (p<0.001) and a higher rate of inflammatory arthritis (p=0.009). Six years after entry to the database, 48 remained in the NP group and 21 remained in the PAD group. At 9 years, 18 and 9 remained respectively. At baseline and at annual follow-up, pregnancy was not found to significantly affect markers of disease progression, including forced vital capacity (p=0.898), (p=0.620), RVSP (p=0.313), eGFR (p=0.426), patient global assessment of activity (p=0.686) and physician global assessment of severity over time (p=0.754).

**Conclusions:** This cohort study demonstrated that having one or more pregnancy after a diagnosis of SSc did not appear to significantly impact patient outcomes across measurements of renal, respiratory or global function over 9 years of follow-up. These results suggest that pregnancy does not impact the course/severity of SSc. Study limitations include a small sample size and biases inherent to retrospective data. This is a hopeful message for patients with scleroderma who are planning a pregnancy.

## P.203

## A META-ANALYSIS TO DETERMINE THE KEY FACTORS PREDICTING SKIN SCORE REGRESSION IN SYSTEMIC SCLEROSIS CLINICAL TRIALS

A. Degachi, J. Avouac, Y. Allanore

*Rheumatology Department, Hôpital Cochin, Université de Paris, Paris, France*

**Introduction:** The modified Rodnan skin score (mRSS) is a validated criterion used to assess the skin involvement in systemic sclerosis (SSc) clinical trials and is part of the emerging composite scores currently under investigation (CRISS). The recent failure of several randomized controlled trials (RCTs) questions the choice of inclusion criteria in these studies. Our objective was to find the factors able to predict the spontaneous regression of mRSS in the control groups of SSc RCTs.

**Material and Methods:** A systematic review of RCTs carried out on SSc patients and with an available prospective assessment of mRSS (17 sites) was performed. We searched [(systemic sclerosis OR scleroderma) AND rodnan] in MEDLINE, EMBASE, CENTRAL and clinicaltrials.gov databases from their inception to July 1st, 2019. An additional search was performed from the references cited by the included articles. We focused on the trials assessing mRSS as primary outcome. The sensitivity to change was measured by the standardized response mean (SRM) in each group (negative values for mRSS improvement).

**Results:** The search retrieved 2556 results. After assessment for eligibility and exclusion of duplicates, 34 trials were included in the meta-analysis. A total of 1618 patients assigned to the control groups of these trials was analyzed. 78.1% of patients were women. The mean age of the control groups varied between 36 and 57.8 years old. The mean disease duration at inclusion varied between 0.6 and 17.2 years. 85.5% of patients had a diffuse cutaneous form of SSc. The mean baseline mRSS varied between 10.3 and 30 (interquartile range IQR 14.3;26.0).

17 trials assessed mRSS as primary outcome (697 patients). The results showed that the mean baseline mRSS was the sole predictor of mRSS change in our work. Among various cut-off values, a baseline mRSS of 20 was the most potent to identify control groups with a spontaneous skin improvement: SRM median (IQR) -0.66 (-0.90;-0.28) for groups with a baseline mRSS > 20 versus +0.19 (-0.39;+1.01) for groups with a baseline mRSS < 20 (p=0.02). According to the analyses performed at the group level, no other variable was found to explain significantly mRSS changes including disease duration, concomitant immunosuppressive treatments (permitted or not), trial duration, year of publication, methodological quality.

**Conclusions:** The baseline mRSS is the key element to define the inclusion criteria in trials dealing with skin involvement in SSc. A baseline mRSS > 20 should be considered for trials aiming to accelerate the spontaneous mRSS improvement.

## P.204

## CURRENT PATIENT REPORTED OUTCOMES (PROS) POORLY REFLECT CHANGES IN LUNG FUNCTION IN PATIENTS WITH SYSTEMIC SCLEROSIS

F. Danzo^1^, K. Gjeloshi^1^, G. Abignano^2,3,4^, A.-M. Dean^3^, F. Masini^1^, G. Cuomo^1^, F. Del Galdo^3,4^

^1^*Complex Operative Unit (UOC), Internal Medicine Department, University of Campania Luigi Vanvitelli, Naples, Italy*, ^2^*Rheumatology Institute of Lucania (IReL), Rheumatology Department of Lucania, San Carlo Hospital, Potenza, Italy*, ^3^*Leeds Institute of Rheumatic and Musculoskeletal Medicine, University of Leeds, Leeds, United Kingdom*, ^4^*NIHR Leeds Biomedical Research Centre, Leeds teaching Hospital, Leeds, United Kingdom*

**Introduction:** In a recent clinical trail the reduction of FVC was not accompanied by a benefit with respect to health-related quality of life and patient-reported outcomes (PROs). The aim of this study is to assess how the change in Pulmonary Function Test (PFTs) parameters correlates with the Patient Reported Outcomes (PROs) in an observational cohort of patients with Systemic Sclerosis (SSc).

**Material and Methods:** We conducted a retrospective study of 330 clinic episodes from 121 unselected patients diagnosed with systemic sclerosis according to EULAR/ACR 2013 criteria, in annual follow-up (for a total of 165 patients/year) with PFTs, Health Assessment Questionnaire Disability Index (HAQ-DI), Scleroderma Health Assessment Questionnaire (sHAQ), Modified Borg Dyspnea Scale (Borg) and Cochin Hand Function Score (CHFS). We assessed the correlation between the HAQ and the Visual Analogical Scale 1-7 at baseline (VAS1 pain, VAS2 disease severity, VAS3 arthritis activity, VAS4 intestinal problems, VAS5 dyspnea, VAS6 Raynaud’s phenomenon, VAS7 digital ulcers). We evaluated the correlation of PFTs with PROs at every time period and the correlation between the change of PFTs parameters (δFVC, δDLCO) with the change of the PROs over a year of follow-up. Following analysis of distribution, Spearman or Pearson Test were used to determine correlation coefficients, as appropiate (Prism 7).

**Results:** The median disease duration was 5 years (IQR 3-10). The median of 12 months δFVC% and δDLCO% were 0 (IQR -5.81 to 3.28) and -2.439 (IQR -8.76 to 5.98), respectively. The analysis evidenced a strong positive correlation between VAS1-7 and HAQ. We observed also significant correlation between FVC%, DLCO% and HAQ-DI (r= - 0.355 and -0.266, respectively; p<0.0001 for both), Borg (r= -0.403 and -0.379, respectively; p<0.0001) and CHFS (r = -0.355 and -0.256, respectively; p<0.0001). Nevertheless, in longitudinal setting there was no significant correlation between δPROs and changes lung function, as continuous variables, neither there was any significant PROs difference in patients that did or did not lose more than 10% of FVC and DLCO over a year of follow-up.

**Conclusions:** This analysis of a monocentric non-selected population evidenced that the current commonly used PROs in SSc while showing a good correlation with lung function are poorly sensitive to change or to reflect changes in lung function over 12 months. In this sense, prudent interpretation of the lack of correlation between FVC and patient-reported outcomes in studies of phase 3 is warranted.

## P.205

## LUNG FUNCTION IS ASSOCIATED WITH EQ-5D CHANGES OVER TIME IN PATIENTS WITH SYSTEMIC SCLEROSIS

J. Ciaffi^1^, N.M. Van Leeuwen^1^, M.K. Ninaber^2^, T.W. Huizinga^1^, J.K. De Vries-Bouwstra^1^

^1^*Leiden University Medical Center - Department of Rheumatology, Leiden, The Netherlands*, ^2^*Leiden University Medical Center - Department of Pulmonology, Leiden, The Netherlands*

**Introduction:** Although in systemic sclerosis (SSc) therapeutic efforts are often directed to prevent progressive respiratory impairment, it is unclear to what extent changes in pulmonary function tests (PFTs) are associated with health-related quality of life (HRQoL). The aim of this study is to evaluate how PFTs contribute to variations in HRQoL assessed through the multidimensional questionnaire EQ-5D over time in patients with SSc.

**Material and Methods:** We included all patients enrolled in the Leiden Combined Care in SSc cohort, fulfilling ACR 2013 criteria, with forced vital capacity (FVC%), diffusing lung capacity for carbon monoxide (DLCO%) and EQ-5D-3L assessed in at least two visits. The EQ-5D-3L consists of two parts. In part I, patients choose among three severity levels in five domains: mobility, self-care, usual activities, pain/discomfort, anxiety/depression, resulting in an utility score from -0.59 to 1. In part II, current health status is rated on a 0-100 Visual Analogue Scale (VAS), with higher values representing better health. The associations between changes in FVC% and DLCO%, and evolution of EQ-5D over time, were investigated through generalised estimating equations using utility score or VAS as dependent variable and adjusting for age, disease duration, smoking status, pulmonary comorbidities and skin fibrosis involving chest or abdomen. Percentages of patients reaching minimum clinically important difference in utility score (MCID 0.05 to 0.10 for improvement and -0.12 to -0.14 for worsening)) were determined.

**Results:** We included 378 patients with median follow-up of 3.7 years (IQR 1.9-6.4) and 2 to 11 annual visits, accounting for 1619 measurements. The models showed that improvement in FVC% is significantly associated with increase in both utility score (beta=0.001; 95%CI 0 to 0.002; p=0.003) and VAS over time (beta=0.188; 95%CI 0.111 to 0.264; p<0.001). Improvement in DLCO% is longitudinally associated with increase in utility score (beta=0.001; 95%CI 0 to 0.002; p=0.038), while for VAS results are non-significant (beta=0.02; 95%CI -0.079 to 0.12; p=0.690). Changes in EQ-5D utility scores exceeding the lower limit of MCID for either improvement or worsening, between any 2 consecutively available time points, were detected in 72% of patients and in a total of 519 measurements. In one case only, the improvement in FVC% could account for the increase in EQ-5D.

**Conclusions:** Our findings show that improvement in pulmonary function is contributing to HRQoL in patients with SSc. However, we also show that the impact of PFTs on HRQoL is small and that other determinants should be taken into account when evaluating efficacy of treatment

## P.206

## PREDICTORS OF PERCEIVED FUNCTIONAL STATUS IN EARLY SYSTEMIC SCLEROSIS: A PROSPECTIVE LONGITUDINAL STUDY OF THE GENISOS COHORT

J. Chernis^1^, M.B. Buni^2^, S. Kazzaz^4^, J. Ying^3^, S. Assassi^1^, M. Mayes^1^

^1^*McGovern Medical School Department of Rheumatology and Clinical Immunogenetics, Houston, USA*, ^2^*University of Texas Health Science Center at Houston MD Anderson Cancer Center, Houston, USA*, ^3^*Formerly University of Texas Health Science Center at Houston, Houston, USA*, ^4^*Private Practice, Houston, USA*

**Introduction:** Systemic sclerosis (SSc) is associated with substantial morbidity and mortality. Despite advances in diagnosis and treatment, SSc continues to have a detrimental impact on patients’ quality of life. Self-reported measures, such as the MHAQ (Modified Health Assessment Questionnaire), are important tools to assess and quantify functional status. The aim of this study was to evaluate the association of disease characteristics and functional status (MHAQ) over time.

**Material and Methods:** This study included 388 patients with baseline MHAQ scores from the Genetics versus Environment in Scleroderma Outcome Study (GENISOS) cohort and a median follow-up of 7.2 years. Baseline disease characteristics were recorded and the association of these characteristics with the log of the MHAQ score was then analyzed at baseline and longitudinally.

**Results:** (See Tables 1–5)

BASELINE: At baseline, an income greater than or equal to $50,000, and higher education were associated with a lower MHAQ (indicating better function) (p-value < 0.001), as was marital status (p-value 0.0021).

Autoantibodies: Positive ACA was inversely associated with baseline MHAQ (p-value 0.034). Positive RNA polymerase III was associated with higher MHAQ (indicating worse function) at baseline (p-values <0.001), whereas no significant association was noted with positive ATA.

CONTINUOUS VARIABLES: Hemoglobin and FVC % predicted were inversely correlated with MHAQ at baseline (p-values of 0.026 and 0.015, respectively). MRSS score was positively correlated with MHAQ (p-value <0.0001). CK, age at presentation, and disease duration were not significantly correlated with baseline MHAQ.

OVER TIME: Univariate generalized linear mixed model analysis using log-MHAQ score as the outcome estimated the effect of baseline or longitudinal predictors to log-MHAQ over time. African Americans and patients with positive RNA poly III had higher MHAQ over time (p-value <0.0001, and 0.0008, respectively). Smokers and non-married patients also had worse MHAQ over time (p-value 0.0014 and 0.0026, respectively). Again, higher education and higher income at baseline were associated with better MHAQ over time. An inverse correlation was again noted between hemoglobin and FVC % predicted at baseline (p-values 0.029, and 0.0004, respectively), and a positive correlation was noted for MRSS score (p-value <0.0001). Patient death during the study period, analyzed with a student’s T-test, was associated with a higher MHAQ score (p-value <0.0001).

**Conclusions:** This is the first, largest, longest, and the most extensive cohort study investigating the predictive factors of MHAQ in individuals with SSc at baseline and over time. These results further emphasize the value of self-reported measures in long-term studies.

## P.207

## RANDOMIZED FEASIBILITY TRIAL OF THE SCLERODERMA PATIENT-CENTERED INTERVENTION NETWORK SELF- MANAGEMENT (SPIN-SELF) PROGRAM

M.-E. Carrier^1^, L. Kwakkenbos^2^, W.R. Nielson^3^, C. Fedoruk^1^, K. Nielson^4^, K. Milette^1^, J. Pope^5^, T. Frech^6^, S. Gholizadeh^7^, L. Hummers^8^, S.R. Johnson^9^, P. Piotrowski^10^, L. Jewett^1^, J. Gordon^11^, L. Chung^12^, D. Bilsker^13^, K. Turner^1^, J. Cumin^1^, J. Welling^14^, M. Sauve^4,15^, T.S. Rodriguez Reyna^16^, M. Hudson^1,17^, M. Larche^18^, S.J. Bartlett^17^, V.L. Malcarne^7^, M.D. Mayes^19^, I. Boutron^20^, L. Mouthon^20^, F. Wigley^8^, B.D. Thombs^1,17^

^1^*Lady Davis Institute of Jewish General Hospital, Montreal, Canada*, ^2^*Behavioural Science Institute, Clinical Psychology, Radboud University, Nijmegen, The Netherlands*, ^3^*St. Josephs Health Care, London, Canada*, ^4^*Scleroderma Society of Ontario, Hamilton, Canada*, ^5^*University of Western Ontario, London, Canada*, ^6^*University of Utah, Salt Lake City, Canada*, ^7^*San Diego State University, San Diego, Canada*, ^8^*Johns Hopkins University School of Medicine, Baltimore, USA*, ^9^*Toronto Scleroderma Program; Mount Sinai Hospital &Toronto Western Hospital; University of Toronto, Toronto, Canada*, ^10^*Private practice - Nutrition, Hamilton, Canada*, ^11^*Hospital for Special Surgery, New York City, USA*, ^12^*Stanford University, Stanford, USA*, ^13^*Simon Fraser University; University of British Columbia, Burnaby; Vancouver, Canada*, ^14^*NVLE Dutch patient organization for systemic autoimmune diseases, Utrecht, The Netherlands*, ^15^*Scleroderma Canada, Hamilton, Canada*, ^16^*Instituto Nacional de Ciencias Médicas y Nutrición Salvador Zubirán, Mexico City, Mexico*, ^17^*Department of Medicine, McGill University, Montreal, Canada*, ^18^*McMaster University, Hamilton, Canada*, ^19^*University of Texas McGovern School of Medicine, Houston, USA* ^20^*Université Paris Descartes, Paris, France*

**Introduction:** Systemic sclerosis (SSc) often results in significant disruptions to daily activities and can negatively affect physical and psychological well-being. Because there is no known cure, SSc treatment focuses on reducing symptoms and improving health-related quality of life (HRQL). Self-management programs are known to increase self-efficacy for disease management in many chronic diseases, but the availability of self-management tools for patients with SSc is limited. Self-management is complex in SSc and must address issues such as skin care, gastrointestinal symptoms, and disfiguring appearance changes, which are typically not part of generic self-management programs. The Scleroderma Patient-centered Intervention Network (SPIN) was established to address this issue and has developed an online self-management program (SPIN-SELF) to increase self-efficacy for disease management and improve HRQL in SSc patients. The aim of this study was to assess the feasibility of conducting a full-scale randomized controlled trial (RCT) of the SPIN-SELF program by evaluating the trial implementation processes, required resources and management, scientific aspects, and participant acceptability and usage.

**Material and Methods:** The SPIN-SELF feasibility trial was conducted via the SPIN Cohort, which was developed as an online platform for embedded pragmatic trials using the cohort multiple RCT design. In total, 40 English speaking Cohort participants with low disease-management self- efficacy (Self-Efficacy for Managing Chronic Disease Scale score less than or equal to 7) who indicated interest in using an online self-management program were randomized with a 3:2 ratio to be offered the SPIN-SELF program or usual care for 3 months. Usage data was examined at 3-months post-randomization; qualitative interviews were conducted to assess user acceptability and satisfaction. Trial personnel time requirements and implementation challenges were logged (Clinicaltrials.gov trial registration: NCT03914781).

**Results:** Between July 6, 2019 and July 27, 2019, 40 SPIN Cohort participants were included in the SPIN-SELF feasibility trial: 26 were allocated to the intervention arm, and 14 to the control arm. Automated eligibility and randomization procedures via the SPIN Cohort platform performed as desired. Additional results (e.g., participant acceptability and usage) are forthcoming and will be presented at the World Scleroderma Congress.

**Conclusions:** The SPIN-SELF Program is a self-help tool that may improve disease-management self-efficacy and HRQL in patients with SSc. The SPIN-SELF feasibility trial confirmed that trial methodology is robust, feasible, and consistent with trial participant expectations. The results will guide adjustments that need to be implemented before undertaking a full-scale RCT of the SPIN-SELF Program to assess effectiveness.

**Figure:**
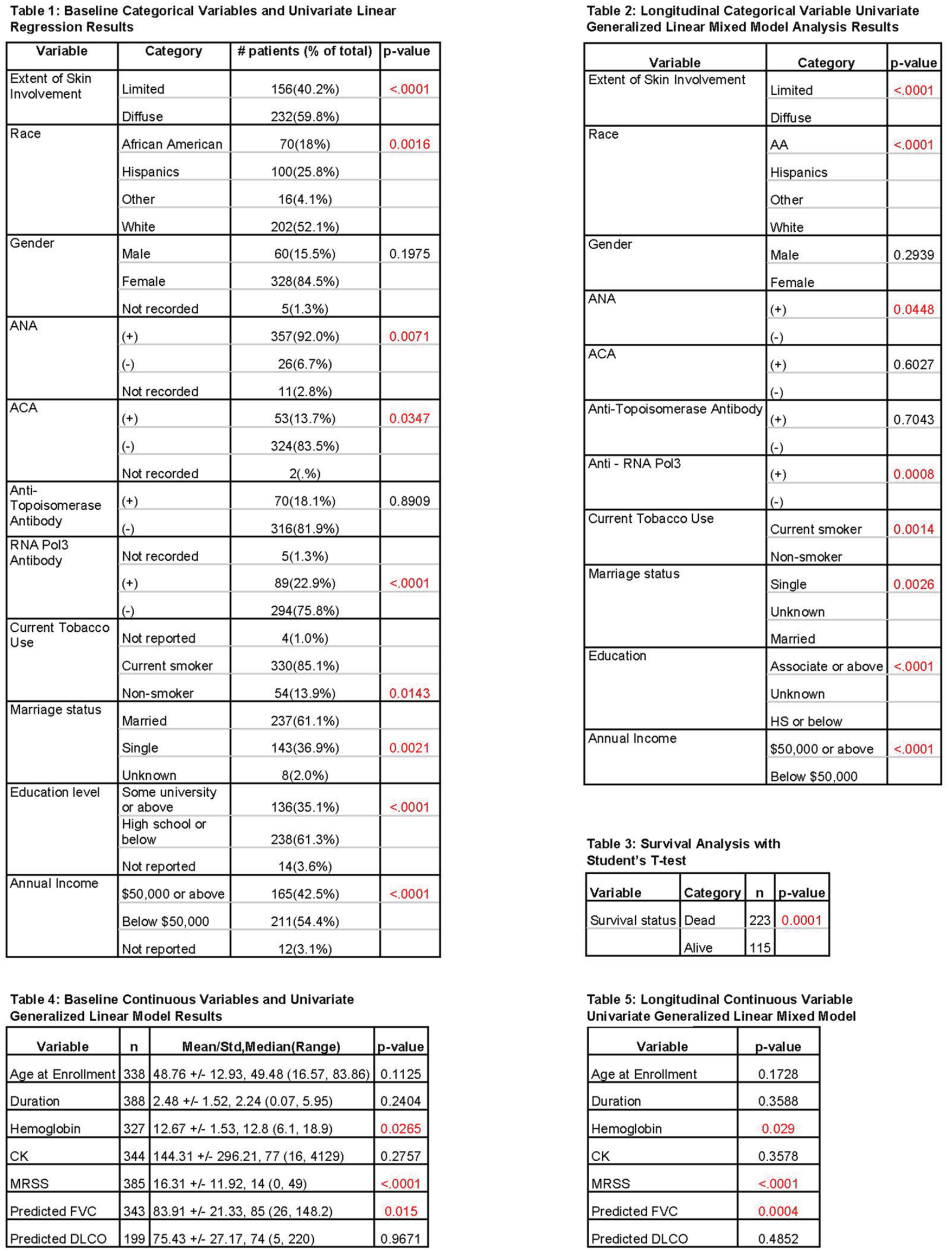


## P.208

## DISABILITY AND HIGH COSTS - TWO IMPORTANT ISSUES IN SYSTEMIC SCLEROSIS

A. Cardoneanu, A. Burlui, E. Rezus

*University of Medicine and Pharmacy Grigore T Popa, Department of Rheumatology, IASI, Romania*

**Introduction:** Systemic sclerosis (SS) has a negative impact on patients’ quality of life and is a problem for society because of the associated costs. Through this study we evaluated the impact of the disease on patients’ wellbeing (degree of disability), as well as on society.

**Material and Methods:** We performed a retrospective study that included 30 patients with SS. Patients were presented at the Rheumatology I Clinic, Clinical Rehabilitation Hospital, from January to March 2019. Disability was measured using the Health Assessment Questionnaire Disability Index (HAQ-DI), Visual Analogue Scale (VAS) scores: VAS - pain, VAS - ulcers, VAS - Raynaud phenomenon. The social costs related to the disease were evaluated by: the number of days of hospitalization and the degree of re-admission of the patients, the cost of the treatment and of the paraclinical investigations, the number of days of sick leave, early retirement.

**Results:** Of the 30 patients included in the analysis, 19 were female (63.3%). The average age was 42.6 years. 22 subjects (73.3%) had a diffuse cutaneous form of SS, and 8 cases presented with a limited form of the disease. Most patients had a marked disability, with VAS scores above 80 mm - VAS-pain (93.3%), VAS-ulceration (63.3%), VAS-Raynaud (86.6%). In 24 of the cases (80%) there was highlighted a HAQ-DI score> 2. 22 patients (73.3%) needed a prolonged hospitalization (over 7 days), 19 (63.3%) were retired due to the disease, and 10 (33.3%) required a re-admission in the next period of time.

**Conclusions:** The results of our study are in correlation with the data from the literature and sustain that SS is a rare and aggressive illness that has a negative impact on patients’ life, correlating with an increased degree of disability, which implies high direct and indirect costs.

## P.209

## PERCEIVED BURDEN AMONG INFORMAL CAREGIVERS OF PERSONS WITH SYSTEMIC SCLEROSIS COMPARED TO OTHER CHRONIC MEDICAL CONDITIONS: A SYSTEMATIC REVIEW

A. Carboni-Jimenez^1^, D.B. Rice^2-3^, M. Cañedo-Ayala^3^, B. Levis^3-4^, M. Imran^3^, M. Chiovitti^3^, B.D. Thombs^2-7^

^1^*McGill University - Department of Psychiatry; Lady Davis Institute of the Jewish General Hospital, Montreal, Canada*, ^2^*McGill University - Department of Psychology, Montreal, Canada*, ^3^*Lady Davis Institute of the Jewish General Hospital - Department of Psychiatry, Montreal, Canada*, ^4^*McGill University - Department of Epidemiology, Biostatistics and Occupational Health, Montreal, Canada*, ^5^*McGill University - Department of Psychiatry, Montreal, Canada*, ^6^*McGill University - Department of Medicine, Montreal, Canada*, ^7^*McGill University - Department of Educational and Counselling Psychology, Montreal, Canada*

**Introduction:** Informal caregivers provide ongoing assistance to a loved one with a health condition, typically without receiving financial compensation or formal training. People living with systemic sclerosis (SSc) often rely on informal caregivers to assist with daily tasks. Caring for a family member or friend has been associated with a high level of burden and reduced quality of life in more common chronic diseases. The current study aimed to compare perceived burden among informal caregivers of people living with SSc versus informal caregivers of patients with other chronic medical conditions.

**Material and Methods:** A systematic search of Cochrane Central, CINAHL, EMBASE, MEDLINE, and PsycINFO databases was conducted from inception through April 18, 2018 to identify original studies that included measures of perceived burden (12 or 22-item Zarit Burden Interview [ZBI-12; ZBI-22]) among caregivers for people with chronic diseases. The ZBI-12 is scored from 0 to 48, where greater scores represent greater caregiver burden. Means and standard deviations (SD) were extracted from each included article. For diseases with at least 2 included studies, means were pooled using random effects meta-analysis.

**Results:** There were 3307 unique references identified from the database search. A total of 85 eligible articles were included in the review. ZBI-12 scores were reported in 9 articles (11.0%), including one study of caregivers to individuals with SSc. SSc caregivers (N = 202) had an average ZBI-12 score of 13.5 (SD = 9.8). All other caregiver groups reported lower burden, including caregivers for essential tremor (N studies = 1; N participants = 57; mean = 6.4; SD = 8.4), spinal cord injury (N studies = 2; N participants = 266; pooled mean = 9.6; 95% confidence internal [CI] = 5.3 to 13.9; I2 = 95.6%), cirrhosis (N studies = 1; N participants = 58; mean = 11.5; SD = 8.4), amyotrophic lateral sclerosis (N studies = 1; N participants = 18; mean = 12.4; SD = 7.9), and heart failure (N studies = 3; N participants = 200; pooled mean = 12.6; 95% CI = 9.5 to 15.7; I2 = 77.2%). No ZBI-22 studies were found of caregivers to individuals with SSc.

**Conclusions:** Perceived burden was higher among SSc caregivers than caregivers of people living with more common diseases, including essential tremor, spinal cord injury, cirrhosis, amyotrophic lateral sclerosis, and heart failure. Interventions designed to reduce caregiver burden in SSc should be developed and tested.

**Figure:**
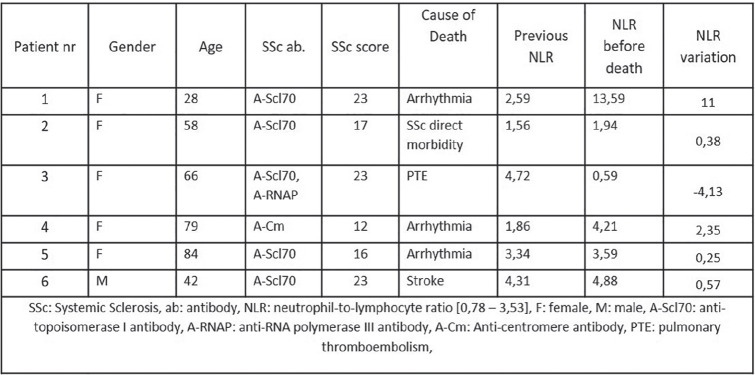


## P.210

## NEUTROPHIL TO LYMPHOCYTE RATIO VARIATIONS IN PATIENTS DECEASED WITH SYSTEMIC SCLEROSIS

V. Bernardino, A.C. Rodrigues, M. Fernandes, A. Lladó, A. Panarra

*Hospital Curry Cabral - Centro Hospitalar Universitário Lisboa Central, Lisbon, Portugal*

**Introduction:** Neutrophil to lymphocyte ratio (NLR) is usually applied as a prognostic marker in cardiovascular disease, infections, inflammatory disorders or cancers. The physiopathology of systemic sclerosis (SSc) is mediated by inflammation. Therefore, NLR can act as predictor of morbidity and/or mortality. The aim of this study is to determine NLR variations in patients deceased in our department having SSc.

**Material and Methods:** This is a retrospective study. Clinical files from deceased patients with SSc were analyzed and data was anonymized when introduced on the database. All patients fulfilled 2013 classification criteria (CC) for SSc. White blood count results included the last values before death and prior results, from months to years before. NLR was calculated dividing absolute neutrophil count by the absolute lymphocyte count. A cut-off between 0,78 and 3,53 for NLR was used. Spearmen correlation was applied, on SPSS software, for p value < 0,05.

**Results:** We identify 6 deaths in our cohort of SSc (table 1), 5 of them were women. Median age was 62 years. Most patients were positive for Anti-topoisomerase I antibody (83,3%). Median SSc score on CC was 20. Causes of death were mainly cardiovascular related. NLR values were not adjusted for geriatric population. NLR variation had no statistic significance (p = 0,507).

**Conclusions:** Although patients had different causes of death, all of them were directly or indirectly related to SSc complications. NLR was above or under the normal values in most of the patients, mainly before just before death. Although this study had a small sample size, these high rates of NLR suggest a possible relation with a systemic inflammation status and should prompt a careful follow-up in these patients. NLR role as a biomarker in SSc is yet to be clarified.

## P.211

## NEUTROPHIL TO LYMPHOCYTE RATIO AND SYSTEMIC SCLEROSIS CLINICAL IMPACT

V. Bernardino, A.C. Rodrigues, M. Fernandes, A. Lladó, A. Panarra

*Hospital Curry Cabral - Centro Hospitalar Universitário Lisboa Central, Lisbon, Portugal*

**Introduction:** Neutrophil to lymphocyte ratio (NLR) is being used as a prognostic marker in cardiovascular disease, infections, inflammatory disorders and cancers. The physiopathology of systemic sclerosis (SSc) is mediated by inflammation. Therefore, NLR can act as a biomarker, to predict morbidity and/or mortality. This study aims to relate NLR and SSc clinical impact.

**Material and Methods:** This is a retrospective cross-sectional study. Clinical data from patients with SSc was consulted. Data was anonymized when introduced on the database. Only patients that fulfilled the 2013 SSc classification criteria were included. These criteria also were used as a marker of SSc clinical impact, by using the value of each patient’s score. White blood count (WBC) was used to calculate the NLR. NLR ratio was considered normal between 0.78 and 3.53. SPSS was the software used for statistics analyses. Mann-Whitney test, two-tailed, for 2 independent samples was used to test correlation between variables, for p< 0,05.

**Results:** Overall, 30 patients met the SSc criteria, 29 were female, with a mean age of 61,4 years. About 76,7% patients presented normal values of NLR. The mean value for SSc criteria score was 15,3 (±4,5); the mean value of NLR was 2,6 (±1,4). There was no relation between NLR and SSc criteria score (p = 0,83).

**Conclusions:** This wass a small sample and SSc classification criteria may not be the best score to access SSc clinical impact. The NLR values used on our population were not adjusted for elderly subjects, which can create a bias in the interpretation of results. WBC can further depend on the patient’s metabolic status. NLR does not relate to SSc clinical impact, when determined by 2013 SSc classification criteria. This result suggests that clinical damage may be present without ongoing active inflammation. A larger sample size is required in order to make appropriate conclusions, as well as a validated SSc score for disease activity.

## P.212

## THE ROLE OF DIFFERENT SIGNS ON THE EVOLUTION OF RAYNAUD’S PHENOMENON (RP) PATIENTS TO DEFINITE SYSTEMIC SCLEROSIS (SSC): RESULTS FROM AN INTERNATIONAL MULTICENTRE STUDY ON VERY EARLY DIAGNOSIS OF SYS

S. Bellando Randone^1^, G. Lepri^1^, D. Huscher^2^, T. Minier^3^, C. Bruni^1^, D.E. Furst^4^, L. Czirjak^3^, M. Cutolo^5^, V. Smith^6^, J. Avouac^7^, S. Guiducci^1^, Y. Allanore^7^, O. Distler^8^, M. Matucci-Cerinic^1^

^1^*University of Florence, Florence, Italy*, ^2^*Charitè Universitaetsmedizin Berlin,Germany, Berlin, Germany*, ^3^*University of Pecs, Pecs, Hungary*, ^4^*UCLA, Los Angeles, USA*, ^5^*University of Genoa, Genoa, Italy*, ^6^*University of Ghent, Ghent, Belgium*, ^7^*Hopital Cochin, Paris, France*, ^8^*University of Zurich, Zurich, Swaziland*

**Introduction:** A definition of the Very Early Diagnosis of SSc (VEDOSS) is needed to support the earliest possible treatment. The aim of the VEDOSS project was to identify, through an at-risk RP population, the predictive factors for the progression of very early SSc patients toward a definite diagnosis of SSc.

**Material and Methods:** We aimed to recruit patients with RP in a prospective, longitudinal, multicenter observational study. Patients had an annual assessment to determine disease progression toward the 2013 ACR/EULAR SSc classification criteria. The proposed VEDOSS criteria were: antinuclear antibodies (ANA), puffy fingers (PF), SSc-specific antibodies (anti-topoisomerase I (ATA), anticentromere (ACA), anti-polymerase III (antipolIII), capillaroscopic abnormalities (giant capillaries/capillary loss) . The endpoint was the fulfilment of the 2013 ACR/EULAR SSc classification criteria. Predictors of evolution were determined by univariate and multivariate Cox regression.

**Results:** Of 1,150 enrolled patients, 553 had at least one follow up visit. After up to 5 years of follow up, 149 (26.9%) fulfilled the 2013 ACR/EULAR criteria and were classified as definite SSc. At multivariate analysis, on a background of RP, the most powerful combinations of signs which significantly predicted the evolution were: SSc-specific antibodies (RR=3.7, 95%CI 2.5-5.5), PF (RR=3.0, 95%CI 2.0-4.4) and ANA (RR=4.7, 95%CI 1.8-12.1) The dual combination of disease specific antibodies (RR=5.4, 95%CI 3.7-7.9) with PF (RR=3.0, 95%CI 2.0-4.4) (p<0.0001, X2=108.2) had a comparable model score as the 2 triple combinations. It is important to note that specific antibodies and PF are always present in the 3 most predictive combinations, thus both becoming signs of pivotal interest when combined with RP toward the evolution to definite SSc. The capillaroscopic modifications were significant when added to specific antibodies/PF (p<0.0001) but seemed not to improve the predictive value of combination models.

**Conclusions:** Our data show that 26.9% of RP patients progressed to definite SSc according to 2013 classification criteria within 5 years of follow up. In addition to RP, the VEDOSS criteria (ATA/ACA/RNAPOLIII, ANA, PF), are the most predictive signs of evolution. In the future, an in depth analysis of capillaroscopic abnormalities is warranted. These data are important for risk stratification of RP patients in clinical practice and clinical studies.

## P.213

## THE INFLUENCE OF SKIN CALCINOSIS ON THE PROGNOSIS OF DIGITAL ULCERS IN PATIENTS WITH SSC

V. Venturini, S. Barsotti, M. Di Battista, A. Della Rossa, M. Mosca

*Rheumatology Unit - Pisa University Hospital, Pisa, Italy*

**Introduction:** Digital ulcers (DUs) are one of the main burdens in patients with systemic sclerosis (SSc) as they have a major impact on quality of life and prognosis. Some DUs are associated with the presence of subcutaneous calcinosis (SC): their correct management may be difficult, and the prognosis of these DUs is still not well defined. The purpose of the present study was to assess whether calcinosis has an impact on the outcome of DUs.

**Material and Methods:** We prospectively collected data from DUs of the hands evaluated at our dedicated wound-care outpatient clinic from October 2018 to August 2019. Sixty patients were enrolled (55 females, 21 with limited-SSc and 39 with diffuse-SSc, mean age 62.3±17.2 years). All the ulcers were managed with weekly treatment following a definite protocol: wound cleansing, disinfection, mechanic debridement, application of antiseptic dressing. For every DU we collected: presence/absence of calcinosis, pathogenesis (spontaneous, post-traumatic), area of DU, location (fingertip, periungual area, metacarpophalangeal, proximal/distal interphalangeal-PIP/DIP), VAS-pain at baseline and after two weeks, local signs of infection (edema, redness), deep wound swab results and time to healing.

**Results:** Out of 101 DUs enrolled, 23 (22.8%) were associated with SC. Six ulcers (2 SC, 4 non-calcinosis) were post-traumatic. There were no significant differences between the mean areas of DUs (SC 22mm2 vs non-calcinosis 30.8mm2). The location of DUs was not different between calcinosis and non-calcinosis: fingertip (14-61% vs 34-49.3%), periungual area (4-17.4% vs 16-23.2%), PIP (2-9% vs 13-18.9%), DIP (2-9% vs 9-13%) and MCP (1-4% vs 4-5.8%). VAS-pain was not statistically different at baseline (6.0 for SC vs 5.4), neither after 2 weeks (3.8 vs 3.2). Although the presence of local signs of infection was similar (5-21.7% vs 13-16.9%), the positivity for wound swab was higher in SC compared with those without calcinosis (6-26.1% vs 9-11.5%; p=0.05). In patients with calcinosis, S.Aureus was the most frequent (4), whereas E.Coli and C.Albicans were found in 1 patient each. S.Aureus was the most frequent bacteria also in patients without calcinosis (6), followed by M.Morganii (2) and E.Coli (1). All DUs treated in our outpatient clinic healed but those with SC required longer time(10.4±8.1 vs. 7.3±5.8 weeks; p=0.05).

**Conclusions:** DUs with or without calcinosis are similar for pathogenesis, location and pain. Despite these aspects, DUs associated with calcinosis are more prone to be infected and require more time to heal. The presence of calcinosis may represent a negative prognostic factor in the management of SSc-DUs.

## P.214

## DEPRESSION IN SCLERODERMA PATIENTS, SYSTEMATIC REVIEW

A. Balbir Gurman^1^, D. Israeli-Cohen^2^, Y. Braun-Moscovici^1^

^1^*Rheumatology Institute, Rambam Health Care Campus, Rappaport Faculty of Medicine-Technion, Haifa, Israel*, ^2^*Psychological Service, Rambam Health Care Campus, Haifa, Israel*

**Introduction:** SSc is associated with pain, compromised physical health, fatigue, damage, disfigurement, exhausting, poor prognosis and disability. SSc-related complications, chronic progressive course, long-term treatments and often visits to clinic or hospitalizations have negative affect on emotional status, personal and social well-being; patients have difficulties in coping with the disease. Depression (unproportioned unhappiness) may be masked by SSc related features. Studies on depression in SSc are mainly based on patient’ self-interview which include validated questionnaires; more solid diagnosis of depression is based on psychologist or psychiatrist consultation or on the documented use of antidepressants. Questionnaires on mental health are a part of Patient Reported Outcome (PRO) and Patient-Reported Outcomes Measurement Information System (PROMIS), they are validated for general population, chronic diseases, and SSc; some instruments for assessment SSc patients include scleroderma specific related features and general aspects of patients’ quality of life (QoL).

**Material and Methods:** We performed a search of the online databases MEDLINE and EMBASE between years 1990 and the end of 2018 for articles regarding depression in SSc. Only full journal articles in English language (reviews, meta-analysis, original articles) were included (N-123).

**Results:** The relative risk of depression in SSc patients is about 1.5; in different SSc cohort the rate of depression was 16-76%.; two third of SSc patients have mild to moderate depression; 10-22% reported on major depression. Major contributors for depression were pain, sleep disturbances and fatigue. Among SSc-related complications, depression correlated with Raynaud’s phenomenon, digital ulcers, diffuse skin involvement, pruritus, dyspnea, digestive problems especially reflux and ano-rectal dysfunction, urinary incontinence and sexual dysfunction, disfigurement and low self-estimation. Patients’ perception of ‘serious consequences’ and ‘illness identity’ correlated with depression and ineffective coping with scleroderma. Depression contributes to mortality of SSc patients independent of the cause of death; out-patient and hospitalized SSc patients with depression have reduced survival. Registered treatment of depression has lower rates than depression itself.

**Conclusions:** Depression is associated with severe negative impact on patients’ QoL, survival and mortality. Recognition of mood disorder is crucial; discordance in high prevalence of mood disorders and treatment may have bad influence on patients’ compliance and copping with scleroderma. Care of scleroderma patients, along with physical assessment, medications and physical rehabilitation, should include psychological assessment, support and depression treatment. Such approach will provide to patients better understanding of their condition and improved coping with scleroderma.

## P.215

## SERUM LEVELS OF VITAMIN D AND THE RELATIONSHIP WITH DISEASE ACTIVITY IN PATIENTS WITH SYSTEMIC SCLEROSIS

C. Pomirleanu^1^, A. Jitaru^1^, E. Ancuta^2^, R. Chirieac^3^, C. Ancuta^1^

^1^*Grigore T Popa University of Medicine and Pharmacy, Iasi, Romania*, ^2^*Elena Doamna Clinical Hospital, Iasi, Romania*, ^3^*SANOCARE Medical and Research Center, Iasi, Romania*

**Introduction:** The aim of this study was to analyze the relationship between serum 25-OH vitamin D levels with disease activity, clinical and laboratory characteristics in patients with systemic sclerosis (SSc).

**Material and Methods:** We performed a prospective observational study on 48 consecutive patients with SSc attending at least once a single academic rheumatology center (EUSTAR center 162). Patients were analyzed according to a standard protocol including clinical (modified RODNAN skin score, Raynaud’s phenomenon, organ involvement) and immunological parameters, nailfold video-capillaroscopy, disability (Scleroderma Health Assessment Questionnaire - Disability Index), activity (EUSTAR score) and severity (MEDSGER Disease Severity Index). 25-OH vitamin D was determined by the immunochemical method with electrochemiluminescence detection (under 20 ng / ml deficiency, 21-29 ng / ml insufficient level, 30-55.5 ng / ml optimal level). Statistical analysis (descriptive, non-parametric correlations) was performed in SPSS-19, p <0.05 in both globally and disease subtypes (diffuse cutaneous and limited cutaneous SSc).

**Results:** Mean 25-OH vitamin D serum level was 17.9 ± 6.7ng/ml. 22 patients (45.8%) had deficient and 19 (39.5%) insufficient vitamin D levels. Moreover, vitamin D concentrations were significantly lower in dcSSc vs. lcSSc (p<0.05), with no association between vitamin D concentration and severity of organ involvement. Significant correlations were found between low vitamin D levels and dcSSc, severe skin involvement, high activity disease and severe disability (p<0.05).

**Conclusions:** Patients with SSc, especially the diffuse cutaneous scleroderma subtype, have low levels of vitamin D; low vitamin D may be an accelerating factor for the severity of scleroderma.

## P.216

## CAUSES OF MORTALITY IN A COHORT OF PATIENTS WITH SYSTEMIC SCLEROSIS

M. Aguilar Zamora^1^, L. Montolio Chiva^1^, A.V. Orenes Vera^1^, I. Vazquez Gomez^1^, E. Valls Pascual^1^, D. Ybañez Garcia^1^, A. Martinez-Ferrer^1^, A. Sendra García^1,2^, I. Torner Hernández^1^, V. Nuñez Monje^1,2^, J.J. Alegre Sancho^1^

^1^*Rheumatology Department. Hospital Universitario Dr. Peset, Valencia, Spain*, ^2^*Fundación para el Fomento de la Investigación Sanitaria y Biomédica de la Comunidad Valenciana-FISABIO., Valencia, Spain*

**Introduction:** Systemic sclerosis (SS) is a systemic autoimmune disease with high morbidity and mortality. In the past years, and due to the improvement in therapy, there has been a change in the pattern of mortality in patients with SS.

Objective: To assess the causes of mortality in a cohort of patients with SS.

**Material and Methods:** Retrospective observational descriptive study. We reviewed the medical records of patients included in a cohort with SS of a tertiary hospital between 2003 and 2017 and those who had died were selected. We collected demographic and clinical variables and the cause of death. The statistical analysis was performed with the SPSS Statistics 22.0 program.

**Results:** There were 45 registered deaths (31.9%) of a total of 141 patients with SS in the specified period. Baseline characteristics compared to the rest of the cohort are shown in Table 1. As expected, patients who died had a higher age and a greater presence of arterial hypertension (p <0.001). More than half of the patients died due to causes not attributable to SS (51.1%), 20% due to SS related causes, and 13 patients (28.9%) due to an unknown cause. Regarding the deaths attributable to SS, half of them were due to heart disease and the other half due to pulmonary disease (37.5% interstitial lung disease and 12.5% pulmonary arterial hypertension). There was no death due to renal crisis.

Regarding deaths unrelated to SS, vascular events were the leading cause (47.8%: 8 cardiac, 2 cerebral and 1 peripheral), followed by infection (26.1%: 3 respiratory, 1 urinary, 1 sepsis sacral ulcers and 1 esophageal candidiasis), although only one patient received immunosuppressive treatment, and neoplasms (21.7%: 2 pulmonary, 1 hepatic, 1 breast, and 1 hematologic).

**Conclusions:** In our cohort of patients with SS, the most common cause of mortality was the non-SS-related and the cardiovascular disease was the major cause in this group. In these patients, in whom the current treatment has managed to control part of the burden of the disease, we should also consider the prevention and treatment of vascular damage as a therapeutic objective.

## P.217

## HAND IMPAIRMENT IN SYSTEMIC SCLEROSIS IN THE PATIENTS FROM THE REPUBLIC OF MOLDOVA

S. Agachi, L. Groppa, E. Deseatnicova, S. Popa, L. Dutca

*Nicolae Testemitanu State University of Medicine and Pharmacy of the Republic of Moldova, CHISINAU, Republic Of Moldova*

**Introduction:** Research on variants and frequency of hand impairment in patients with systemic sclerosis depending on the clinical form of the disease.

**Material and Methods:** The study included 250 patients with systemic sclerosis (SS) with a mean age of 47.3 ± 3.6 years and average duration of the disease - 16.8 ± 5.4 years. Of them 193 (77.2%) presented the limited form, and 57 (22.8%) - the diffuse form of the disease.

**Results:** Raynaud’s syndrome was present in most patients (241 (96.4%)), complicated with digital ulcers in 75.5% (182 patients).

Joint involvement was diagnosed in 78 (31.2%) patients, predominantly in those with the diffuse form (43 versus 35 with limited form).

Tenosynovitis was registered in 63 (25.2%) patients, especially with the diffuse form (33 versus 30).

Digital contractures were registered in 54 (21.6%) patients, especially installed in the early period in diffuse form (41 versus 13).

Skin changes of puffy-fingers type were observed in 27 (10.8%) patients, skin sclerosis (including sclerodactyly) in 195 (78%) and atrophy of the skin of the hands in 28 (11.2%) patients.

Calcinosis was present in 57 (22.8%) of patients, predominantly affecting those with limited form (46 versus 11 with diffuse form).

Acroosteolysis was diagnosed in 38 (15.2%) of the patients, more frequent in the diffuse form group (22 versus 16 with the limited form).

**Conclusions:** The causes of hand dysfunction in patients with systemic scleroderma are various, targeting the skin, the locomotor system, vessels, digital contractures, calcinosis and acroosteolysis, being most commonly recorded in the patients with the diffuse form of the disease.

**Figure:**
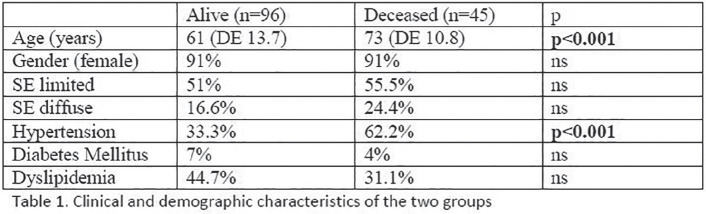


## 6. Imaging

## P.218

## FAST TRACK ALGORITHM”: HOW TO DIFFERENTIATE A “SCLERODERMA PATTERN” FROM A “NON-SCLERODERMA PATTERN

V. Smith^1,2,3^, A. Vanhaecke^1,2^, A.L. Herrick^4,5^, O. Distler^6^, M.G. Guerra^7^, C.P. Denton^8^, E. Deschepper^9^, I. Foeldvari^10^, M. Gutierrez^11^, E. Hachulla^12^, F. Ingegnoli^13^, S. Kubo^14^, U. Mueller-Ladner^15^, V. Riccieri^16^, A. Sulli^17^, J.M. van Laar^18^, M.C. Vonk^19^, U.A. Walker^20^, M. Cutolo^17^

^1^*Department of Internal Medicine, Ghent University, Ghent, Belgium*, ^2^*Department of Rheumatology, Ghent University Hospital, Ghent, Belgium*, ^3^*Unit for Molecular Immunology and Inflammation, VIB Inflammation Research Center (IRC), Ghent, Belgium*, ^4^*Div of Musculoskeletal & Dermatological Sciences, The University of Manchester, Salford Royal NHS Foundation Trust, Manchester, United Kingdom*, ^5^*NIHR Manchester Biomedical Research Centre, Manchester Univ NHS Foundation Trust, Manchester Academic Health Sciences, Manchester, United Kingdom*, ^6^*Department of Rheumatology, University Hospital Zurich, Zurich, Switzerland*, ^7^*Rheumatology Department, Centro Hospitalar Vila Nova de Gaia/Espinho, Vil Nova de Gaia, Portugal*, ^8^*Department of Rheumatology, University College London, Royal Free Hospital, London, United Kingdom*, ^9^*Biostatistics Unit, Department of Public Health, Ghent University, Ghent, Belgium*, ^10^*Centre for Paediatric and Adolescent Rheumatology, Hamburg, Germany*, ^11^*Division of Musculoskeletal and Rheumatic Disorders, Instituto Nacional de Rehabilitación, Mexico City, Mexico*, ^12^*Univ. Lille, CHU Lille, Dépt de Médecine Interne et Immunologie Clinique, CCERAINO, LIRIC, INSERM, Lille, France*, ^13^*Department of Clinical Sciences and Community Health, University of Milan; Division of Rheumatology, ASST G. Pini, Milan, Italy*, ^14^*The First Department of Internal Medicine, University of Occupational and Environmental Health, Fukuoka, Japan*, ^15^*Department of Rheumatology and Clinical Immunology, Justus-Liebig University of Giessen, Campus Kerckhoff, Bad Nauheim, Germany*, ^16^*Department of Internal Medicine and Medical Specialties, Sapienza University of Rome, Rome, Italy*, ^17^*Research Laboratory and Academic Division of Clinical Rheumatology, Department of Internal Medicine, University of Genoa, Genoa, ITALY*, ^18^*Department of Rheumatology and Clinical Immunology, University Medical Center Utrecht, Utrecht, The Netherlands*, ^19^*Department of Rheumatology, Radboud University Medical Center, Nijmegen, Nijmegen, The Netherlands*, ^20^*Department of Rheumatology, University Hospital Basel, Basel, Switzerland*

**Introduction:** The European League Against Rheumatism Study Group on Microcirculation in Rheumatic Diseases (EULAR SG MC/RD) is a non-profit international network of expert centres. Its main research focus is to investigate the morphology and function of the microcirculation with different non-invasive techniques such as nailfold videocapillaroscopy (NVC). NVC is of paramount importance for the differential diagnosis of primary and secondary Raynaud’s phenomenon, and is part of the 2013 ACR/EULAR classification criteria for systemic sclerosis (SSc). As there is a wide variety of “non-scleroderma patterns”, the categorisation of capillaroscopic images as “non-scleroderma patterns” may be a challenge to the capillaroscopist. This study was designed to propose a simple “Fast Track algorithm” for capillaroscopists of any level of experience to differentiate “scleroderma patterns” from “non-scleroderma patterns” on capillaroscopy and to assess its inter-rater reliability (see Figure).

**Material and Methods:** Based on existing definitions to categorise capillaroscopic images as “scleroderma patterns” and taking into account the real life variability of capillaroscopic images described standardly according to EULAR SG MC/RD, a fast track decision tree, the “Fast Track algorithm” was consented by the principal expert (VS) to facilitate swift categorisation of an image as “non-scleroderma pattern (category 1)” or “scleroderma pattern (category 2)” (see Figure 1). Mean inter-rater reliability between all raters (experts/attendees) of the 8th EULAR capillaroscopy course (Genoa, 2018) and, as external validation, of the 8th EUSTAR course on SSc (Nijmegen, 2019) versus the principal expert, as well as reliability between the rater pairs themselves was assessed by mean Cohen’s and Light’s kappa coefficients.

**Results:** Mean Cohen’s kappa was 1/0.96 (95% CI 0.95-0.98) for the 6 experts/135 attendees of the 8th EULAR capillaroscopy course and 1/0.94 (95% CI 0.92-0.96) for the 3 experts/85 attendees of the 8th EUSTAR SSc course. Light’s kappa was 1/0.92 at the 8th EULAR capillaroscopy course, and 1/0.87 at the 8th EUSTAR SSc course.

**Conclusions:** For the first time, a clinical expert based fast track decision algorithm has been developed to differentiate a “non-scleroderma” from a “scleroderma pattern” on capillaroscopic images, demonstrating excellent reliability when applied by capillaroscopists with varying levels of expertise versus the principal expert and corroborated with external validation.

**Figure:**
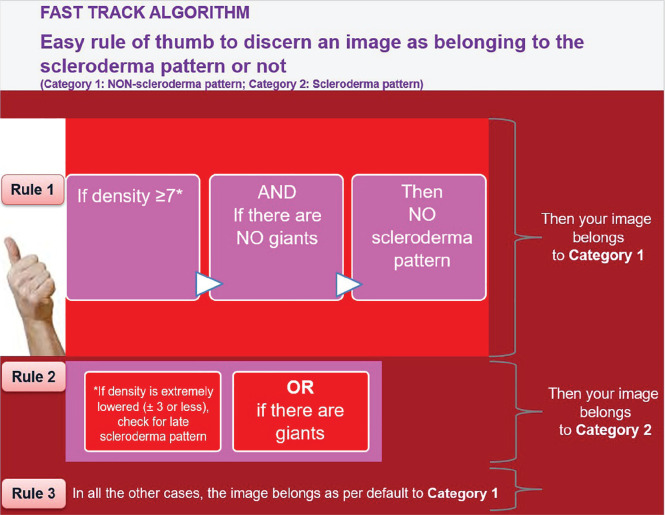


## P.219

## ASSESSING SKIN STIFFNESS IN SYSTEMIC SCLEROSIS THROUGH SHEAR-WAVE ELASTOGRAPHY: A FIVE-YEAR FOLLOW-UP STUDY

T.S. Santiago^1^, M. Santiago^1,2^, M.J. Salvador^1,2^, J.A. P. Da Silva^1,2^

^1^*Rheumatology Department, Centro Hospitalar e Universitário de Coimbra, Coimbra, Portugal*, ^2^*Faculty of Medicine, University of Coimbra, Coimbra, Portugal*

**Introduction:** Measurement of skin involvement is essential for diagnosis and assessment of prognosis and disease progression in systemic sclerosis (SSc). The mRSS is the gold standard measure of skin thickness, but it has been criticised for lack of objectivity, poor inter-observer reproducibility and lack of sensitivity to change. Recently, shear-wave elastography (SWE) provided a potential for objective and quantitative assessment of skin in SSc patients. However, no studies have evaluated its sensitivity to change over-time. Our objective was to assess changes in skin stiffness in SSc patients using shear-wave elastography during a five-year follow-up.

**Material and Methods:** Skin stiffness [i.e. shear-wave velocity values (SWV) in m/s] was assessed by SWE ultrasound (using virtual touch image quantification) at the 17 sites corresponding to the mRSS, in each participant, at baseline and follow-up. mRSS was performed at both time points. Differences between groups were analysed using the related-samples Wilcoxon Signed Rank test and the Mann–Whitney U test.

**Results:** We included 21 patients [85.7% females; mean age 56.3 (10.4) years at baseline, 57.1% with limited SSc] and, 15 healthy controls [73.3% females; mean age 53.6 (14.1) years)]. The median follow-up was 4.9 (0.4) years.

Skin stiffness decreased significantly at all skin Rodnan sites (p<0.001), (except in the fingers), in SSc patients, over time. The same phenomenon occurred in controls, but to a lesser degree in terms of percentage change reduction (Figure 1).

The percentage change reduction in skin stiffness varied in the different skin Rodnan sites and in different phases of the disease. Namely, patients in an oedematous phase at baseline had a higher degree of percentage change reduction in skin stiffness, in the majority of skin Rodnan sites, than patients in a fibrotic phase.

In addition, SWV values also decreased significantly in 15/16 skin sites with local normal Rodnan at baseline (i.e. equal to zero), whereas local Rodnan skin score only changed significantly in the upperarm (p=0.046) and forearm (p=0.026).

**Conclusions:** Although preliminary, this study provides first-time evidence suggesting that skin SWV values are more sensitive to change over time than mRSS; and, reduce significantly over time in SSc and normal controls. Our results highlight the discriminant ability of SWE in detecting subtle skin changes not identified by mRSS. SWE may offer a significant improvement in the evaluation of skin stiffness and may provide relevant insights into the biology of healthy and scleroderma skin.

## P.220

## EVALUATING USE OF T1-MOLLI MAPPING IN ASSESSING SYSTEMIC SCLEROSIS CARDIAC INVOLVEMENT

S.-A. Ng^1^, S. Marchesseau^2^, J.C. Allen^3^, J. Totman^2^, A.H. Low^1,3^

^1^*Department of Rheumatology and Immunology, Singapore General Hospital, Singapore, Singapore*, ^2^Clinical Imaging Research Center, A*STAR & National University of Singapore, Singapore, Singapore, ^3^*Duke-National University of Singapore, Singapore, Singapore*

**Introduction:** Systemic sclerosis (SSc) is a multi-systemic autoimmune disease characterized by fibrosis and vascular changes. Diffuse cutaneous systemic sclerosis patients with symptomatic cardiac involvement have high disease-associated mortality, with the greatest impact occurring in the first 5 years (14% mortality rate). Cardiac involvement, however often remains subclinical in the majority of SSc patients. Development of a new magnetic resonance imaging (MRI) sequence, T1-MOLLI (modified-look-locker inversion recovery) mapping, has enabled the detection and quantification of diffuse fibrosis, without the need for contrast. T1-MOLLI values have also been correlated with the degree of biopsy-quantified cardiac fibrosis.

Our primary objective was to compare cardiac MRI T1-MOLLI mapping in SSc patients and healthy controls. We also sought to investigate the relationship between findings of cardiac fibrosis on T1-MOLLI mapping and indices of SSc severity in other organs.

**Material and Methods:** Sixteen patients fulfilling the 2013 ACR/EULAR criteria for SSc and 17 healthy age-matched controls were recruited and underwent cardiac MRI T1-MOLLI mapping. Association of T1-MOLLI with severity of skin thickening (using modified Rodnan skin score, mRSS), gastrointestinal tract (GIT) involvement (using validated SSc GIT questionnaire score) and restrictive lung disease (using %predicted forced vital capacity, FVC) was investigated using linear regression.

**Results:** Eleven patients had limited cutaneous SSc and 5 had DcSSc. Majority of SSc patients were female (87.5%). Mean (SD) cardiac T1 value (ms) was significantly higher in SSc patients than controls [1329 (88.0) vs 1238 (93.7), p=0.0075], indicating presence of cardiac fibrosis.

13 out of 16 (81.3%) SSc patients had elevated T1 MOLLI values, using a normal cut-off threshold of 1276 ms. However, only 1 patient was clinically symptomatic with arrhythmia.

No significant association was found between cardiac T1 values and skin scores, GIT scores or %predicted FVC).

**Conclusions:** Cardiac MRI T1-MOLLI mapping demonstrated that SSc patients who were predominantly asymptomatic showed evidence of cardiac fibrosis. Cardiac MRI T1-MOLLI has potential to be developed as a useful diagnostic and monitoring tool for SSc cardiac disease.

## P.221

## PROGNOSTIC ROLE OF CARDIAC MAGNETIC RESONANCE IN SYSTEMIC SCLEROSIS PATIENTS WITH PRIMARY HEART INVOLVEMENT, A FOLLOW-UP STUDY

G.B. Canestrari^2^, G. De Luca^5^, E. De Lorenzis^1^, G. Natalello^1^, L. Gigante^1^, L. Verardi^1^, F.A. Gabrielli^3^, R. Marano^4^, E. Gremese^1,2^, S.L. Bosello^2^

^1^*Institute of Rheumatology, Catholic University of the Sacred Heart, Rome, Italy*, ^2^*Department of Rheumatology, Fondazione Policlinico Universitario A. Gemelli - IRCCS, Rome, Italy*, ^3^*Department of Cardiology, Fondazione Policlinico Universitario A. Gemelli - IRCCS, Rome, Italy*, ^4^*Department of Radiology, Fondazione Policlinico Universitario A. Gemelli - IRCCS, Rome, Italy*, ^5^*Department of Immunology, Rheumatology, Allergology and Rare Diseases (UNIRAR), San Raffaele Hospital - IRCCS, Milan, Italy*

**Introduction:** Cardiac magnetic resonance (CMR) represents the “gold standard” to detect, in a non-invasive way, the several patterns that lead to myocardial damage in primary heart involvement during Systemic Sclerosis (SSc). We evaluated the possible prognostic value of repeated CMR in SSc patients with symptomatic heart involvement.

**Material and Methods:** Consecutive SSc patients with symptoms of cardiac involvement or signs of cardiac failure and elevation of cardiac enzymes or NT-proBNP underwent CMR. The CMR was repeated after one year in all patients and subsequently if clinically appropriate. Patients with secondary heart involvement were excluded from the study.

**Results:** We enrolled 23 SSc patients with a mean age of 46.6±13.5 years, mean disease duration of 6.3±5.9 years, 86.9% female, 61.0% with diffuse cutaneous SSc and 56.5% with anti-Scl70 antibodies positivity. The mean follow-up time was of 65.0±22.2 months; a total of 81 repeated CMR studies were performed. At baseline, CMR demonstrated abnormalities in 15 patients (65.2%). Of those 6 (42.0%) patients presented T2 hyperintensity and 8 (57.1%) presented late gadolinium enhancement (LGE; distinguished in subepicardial, mid-wall and subendocardial) while none presented early gadolinium enhancement. Seven patients (30.0%) showed hypo/akinetic areas. CMR abnormalities were confirmed in 12 patients, while the development of new areas of T2 hyperintensity, subepicardial and subendocardial LGE and hypo/akinesia was detected in 5 patients; finally, 6 CMR previously negative remained unremarkable despite new cardiac symptoms. A significant reduction of the ejection fraction of left (LVEF) and right ventricles (RVEF) was noticed over time (59.4±10.6%vs56.1±11.3%, p<0.001 and 54.6±12.0%vs52.1±12.3%, p<0.001, respectively). During the follow-up, 7 patients (30.0%) died for arrhythmias or heart failure. No differences in baseline LVEF, RVEF, left and right ventricular end-systolic volume (ESV) and left and right ventricular end-diastolic volume (EDV) emerged between patients who died compared to survivors. At 1 year CMR, patients who died presented an increased right EDV compared to survivors (105.3±29.9 ml vs 79.8±16.9 ml; p = 0.03), an increased right ESV (61.9±25.3 ml vs 34.3±14.6 ml; p = 0.001) and a lower RVEF (42.1±10.2%vs58.0±9.1%; p=0.001). Deterioration inRVEF during the first year was greater in patients who died compared to survivors (10.3±16.0%vs0.6±9.1%; p = 0.03). No differences were observed in left ventricular CMR parameters between patients who died and survivors during the follow-up.

**Conclusions:** CMR represents a useful and comprehensive tool to assess myocardial damage in SSc patients. Our data suggest that repeated CMR could identify patients with a poor cardiac outcome and worse prognosis especially when a worsening of right ventricle parameters occurs.

## P.222

## USING DEEP LEARNING IN OPTOACOUSTIC AND OPTICAL NAILFOLD CAPILLAROSCOPY IMAGES TO DIFFERENTIATE BETWEEN PATIENTS WITH SSC AND CONTROLS

S. Nitkunanantharajah^1^, K. Haedicke^2^, T. Moore^3^, J. Manning^3^, G. Dinsdale^3^, M. Berks^4^, D. Jüstel^1^, V. Ntziachristos^1^, C. Taylor^4^, M. Dickinson^5^, A. Herrick^3^, A. Murray^3^

^1^*Technische Universität München, Munich, Germany*, ^2^*iThera Medical, Munich, Germany*, ^3^*Centre for Musculoskeletal Research, University of Manchester, Manchester, United Kingdom*, ^4^*Centre for imaging Science, University of Manchester, Manchester, United Kingdom*, ^5^*Department of Physics and Astronomy, University of Manchester, Manchester, United Kingdom*

**Introduction:** SSc-related microvascular changes can be easily observed cutaneously at the finger nailfold. Nailfold capillaroscopy is now used routinely for inspection of the capillaries. The characteristic changes in capillary structure (decreased capillary density, increased capillary width and angiogenesis) form part of the 2013 classification criteria for SSc. The new technique of optoacoustic mesoscopy, a combination of optical and ultrasound imaging offers a new, 3D, non-invasive perspective of capillaries. We have previously reported that the images obtained by optoacoustic mesoscopy offer similar resolution to some capillaroscopy techniques. Quantitative measures of density and vascular volume significantly differ between optoacoustic images from patients with SSc and controls. The aim of this study was to use deep learning to determine whether an artificial neural network could be used to correctly classify images from patients with SSc and healthy controls.

**Material and Methods:** Optoacoustic (3D, iThera, Germany) and ‘standard’ capillaroscopy images (2D, Optillia, Sweden) of the bilateral ring finger nailfold in 24 patients with SSc and 19 controls were acquired. From the 3D optoacoustic images 2D, greyscale, maximum intensity projections were created. Optillia images were downsized to a similar resolution to the optoacoustic mages. For data augmentation purposes each image, from both the optoacoustic and capillaroscopy data sets, was sliced into multiple overlapping image sections of fixed size. ‘Disease’ classification (SSc vs control) was performed on the image sections (using transfer learning) with pretrained neural networks The model learns general image features on images and, subsequently, was fine-tuned on the given data to classify based on the previously learned features.

**Results:** A validation of the model on 50 random data splits showed an average classification accuracy of 0.81 ± 0.15 on the subject level, with an area under the ROC curve of 0.88 ± 0.13 for optoacoustic data. The classification sensitivity and specificity were 0.77 ± 0.21 and 0.84 ± 0.22 respectively. Performing the same task on capillaroscopy images, achieved an average accuracy of 0.86 ± 0.12 on patient level (AUC: 0.92 ± 0.09).

**Conclusions:** Deep learning is able to achieve excellent differentiation between images from patients with SSc and controls for both optoacoustic and standard capillaroscopy. Limitations of the study include the relatively small participant numbers. In addition, direct comparison of accuracy between images from the two techniques may be limited since whilst images were acquired in the same area of the finger the same capillaries may not have always have been captured.

## P.223

## WHICH NON-INVASIVE TECHNIQUES CAN BEST BE USED TO IMAGE CALCINOSIS?

T. Moore^1^, E. Marjanovic^1^, J. Manning^1^, G. Dinsdale^1^, S. Wilkinson^1^, M. Dickinson^2^, A. Herrick^1^, A. Murray^1^

^1^*Division of Musculoskeletal and Dermatological Sciences, MAHSC, Salford Royal NHS Foundation Trust, University of Manche, Manchester, United Kingdom*, ^2^*Dept of Physics and Astronomy, University of Manchester, Manchester, United Kingdom*

**Introduction:** Patients with systemic sclerosis (SSc) frequently develop subcutaneous calcinotic deposits which are painful and can perforate the skin, causing ulceration. There is currently no effective treatment. Calcinosis affects up to 25% of patients with SSc and the underlying aetiology is unknown. Diagnosis is often made with plain x-ray; however this is unsuitable for long-term monitoring. Thus identifying techniques to measure calcinoses longitudinally will improve our understanding of and will facilitate improved development of potential treatments .

**Material and Methods:** Twenty-one female patients with SSc (median age 63 (IQR 55 - 70) years; median Raynaud’s phenomenon duration 23 (IQR 12- 36) years; median disease duration since onset of first non-Raynaud’s feature 14 (IQR 9 - 23) years) underwent imaging of calcinosis. Measurements of perfusion (laser speckle imaging and thermography [a pseudo measure of perfusion]), oxygenation (multispectral imaging), vessel size and depth (high frequency ultrasound) were performed at the site of calcinosis and adjacent skin

**Results:** Perfusion at the site of the calcinosis was decreased as compared to the adjacent area as measured by speckle imaging, median 107.3 [IQR 60.3-213.4] vs 312.1 [IQR 107.8-432.2] arbitrary perfusion units; p<0.01. Skin temperature was also reduced (median calcinosis, 31.4 (IQR 28.4 - 35.6) vs median adjacent 32.8 (IQR 28.4-35.7); p= 0.052. There was no difference in the oxygenation at the site of calcinosis vs adjacent, median 0.15 (IQR 0.07 - 0.22) vs. 0.16 (IQR 0.00 - 0.21) arbitrary units; non-significant. The median depth of the calcinoses was 1.5 (IQR 1.11 - 2.07) cm and the median lesion area was 3.06 (IQR 2.33 - 4.62) cm2. No relationships were identified between perfusion, oxygenation or calcinosis depth and area.

**Conclusions:** Laser speckle imaging and thermography indicated significant differences between calcinotic and adjacent areas. That perfusion was decreased in the area of the calcinosis vs the adjacent skin may be due to the pressure on the skin leading to ischaemia, or it may be that calcinosis develops in areas which are under perfused. This study has demonstrated the feasibility of imaging calcinosis properties. Of particular interest is the ability to measure the depth and subcutaneous area with high frequency ultrasound which may aid assessment of treatment efficacy.

## P.224

## IMAGING THE STRUCTURE AND FUNCTION OF SYSTEMIC SCLEROSIS-RELATED TELANGIECTASES; A FEASIBILITY STUDY

E. Marjanovic^1^, T. Moore^1^, J. Manning^1^, G. Dinsdale^1^, S. Wilkinson^1^, M. Dickinson^2^, A. Herrick^1^, A. Murray^1^

^1^*Centre for Musculoskeletal Research, Division of Musculoskeletal and Dermatological Sciences, University of Manchester, Manchester, United Kingdom*, ^2^*Dept of Physics and Astronomy, University pf Manchester, Manchester, United Kingdom*

**Introduction:** Telangiectases are a hallmark feature of systemic sclerosis (SSc), representing its systemic microvascular changes. Patients with SSc report that telangiectases can lead to significant psychological distress due to their (often) unsightly appearance. Telangiectases can be resistant to laser treatment and often reoccur after initially successful treatment. Improving understanding of their subsurface structure may facilitate improved treatment response. The objective of this study was to use non-invasive imaging techniques to examine SSc-related telangiectases. The hypothesis of the study was that relationships existed between oxygenation, perfusion, skin colour and size.

**Material and Methods:** Twenty-two patients (non-smokers) with SSc-related cutaneous telangiectases (2 men, 20 women; 18 limited and 4 diffuse cutaneous SSc) with a median age of 62 years (IQR 55-69 years) were recruited into the study. The median number of years since onset of Raynaud’s phenomenon was 23 years (IQR 13-36 years). Patients underwent imaging of telangiectases and adjacent unaffected skin to measure perfusion (laser Doppler imaging), oxygenation (multispectral imaging), vessel diameter and depth (high frequency ultrasound), colour and lesion size (dermoscopy, images split for colour into red, green and blue channels; areas manually marked in bespoke software). The relationship between these different microvascular measures was assessed.

**Results:** Median perfusion was 661 (IQR 240-1039) in telangiectases vs 144 (86-334) in adjacent tissue, perfusion units, p<0.01; Colour (green channel intensity) 127 (115 to 149) vs.156 (137 to 187), p<0.01; indicative that telangiectases are ‘redder’. Oxygenation was 0.16 (0 to 0.24) vs. 0.08 (0 to 0.16) arbitrary units, non-significant (NS); mean vessel diameter was 0.19 (0.09 to 0.39) and mean depth was 1.06 (0.9 to 2.02) mm; lesions were composed of between 2 and 5 measureable vessels. Perfusion was associated with oxygenation (R=0.597, p= 0.031). Oxygenation within the lesion was negatively associated with the colour of the lesion (green channel of the images -0.56, p=0.04). Vessel diameter was not related to depth, lesion size or colour.

**Conclusions:** This study demonstrated the feasibility of imaging telangiectases with different techniques. There was increased perfusion and ‘redness’ of the skin at the site of the telangiectases. Oxygenation increased with perfusion indicating that telangiectatic vessels do not appear to function abnormally. Of particular interest was that individual vessels could be resolved with high frequency ultrasound which therefore offers a possible new modality to allow imaging pre and post treatment. This will allow further understanding of the mechanisms underpinning why some lesions (but not others) recur after treatment.

## P.225

## INFRARED THERMOGRAPHY FOR DIAGNOSTIC MANAGEMENT OF MICROVASCULOPATHY IN SYSTEMIC SCLEROSIS: IS IT A GOOD ALTERNATIVE FOR NAILFOLD VIDEOCAPILLAROSCOPY?

B. Miziolek, A. Lis-Swiety, A. Skrzypek-Salamon, L. Brzezinska-Wcislo

*Department of Dermatology, School of Medicine in Katowice, Medical University of Silesia, Katowice, Poland*

**Introduction:** Systemic sclerosis (SSc) is a multisystemic disease with an extensive microvasculopathy. The gold standard for its investigation is nailfold videocapillaroscopy (NVC). Another noninvasive technique with increasing availability due to falling prices of mobile devices is infrared thermal thermography (IRT). This is an imaging technique which allows for an indirect assessment of blood flow by measuring a surface temperature of the skin. Although its utility was demonstrated for diagnostic management or an evaluation of a response to treatment in Raynaud’s phenomenon, studies which investigate correlations between IRT parameters and vascular abnormalities seen at the nailfolds are scarce in literature. The aim of this study was to assess a value of IRT for an assessment of microvasculopathy in patients with SSc.

**Material and Methods:** There were enrolled 19 patients with limited cutaneous SSc. They were submitted to IRT imaging and NVC. An average temperature (Tavg) at the nailfold, and a gradient of temperatures (δTavg) between the central metacarpus of the hand and the nailfold was determined for all fingers. NVC pictures were classified to capillaroscopic patterns according to Cutolo et al. system as well as they were analyzed quantitatively to measure a density of capillaries and to calculate capillaroscopic skin ulcers risk index (CSURI) for each finger separately.

**Results:** There was only a moderate correlation (r greater than 0,4, but below 0,6) between thermographic parameters and density of capillaries in fingers II-V (r=0,5, p<0,001 for Tavg and r= -0,45, p<0,001 for δTavg), but none in thumbs (r=0,29, p=0,089 for Tavg and r= -0,19, p=0,275 for δTavg). Early pattern was associated with significantly greater surface temperature (Tavg) of nailfolds and essentially milder δTavg in fingers II-V when compared to all other capillaroscopic patterns in fingers II-V. Surface temperature (Tavg) was significantly lower and δTavg was markedly more pronounced in fingers II-V with calculated by CSURI a greater risk of development of digital ulcers (DU).

**Conclusions:** Although IRT measurements correlate only moderately with density of capillaries, this technique seems to be substantial to distinct between capillaroscopic patterns as well as to identify those patients at greater risk of DU development.

## P.226

## THYROID DISORDERS IN PATIENTS WITH SYSTEMIC SCLEROSIS: BIOCHEMICAL AND SONOGRAPHIC CHARACTERISTICS

K. Meridor^1^, P. Rotman-Pikielny^2^, M. Werner^3^, Y. Levy^1^

^1^*Department of Internal Medicine, Meir Medical Center, Kfar-Saba, Israel*, ^2^*Department of Endocrinology, Meir Medical Center, Kfar-Saba, Israel*, ^3^*Department of Radiology, Meir Medical Center, Kfar-Saba, Israel*

**Introduction:** Previous studies have shown elevated risk for thyroid autoimmune diseases in patients with systemic sclerosis (SSc). It is also known that patients with SSc have an increased risk of malignancy compared with the general population. Increased risk for thyroid nodules and cancer was demonstrated in other autoimmune diseases like systemic lupus erythematosus, but the data regarding thyroid nodules and cancer in SSc patients is scarce.

The aim of our study was to evaluate the thyroid gland in SSc patients using biochemical and sonographic tools.

**Material and Methods:** Complete thyroid workup was conducted in a group of consecutive patients fulfilling the 2013 ACR/EULAR classification criteria for SSc. This included measurement of Thyroid-stimulating hormone (TSH), free thyroxine (fT4), anti-thyroglobulin (aTG) and anti-thyroid peroxidase (aTPO) autoantibodies as well as performing thyroid ultrasound and fine needle aspiration cytology when necessary.

**Results:** Fifty patients (44 females, 6 males; mean age 50.4±14.6 years) with dsSSC and lsSSC (40 and 10 cases, respectively) were evaluated. Median disease duration was 6.5 years (range 0.5-38 years) with clinical manifestations involving mainly the skin, gastrointestinal tract and the respiratory system (in 90%, 82%, and 56%, respectively). Ten patients were previously diagnosed with hypothyroidism, 8 patients (16%) had autoimmune thyroid disease, two had a hemi-thyroidectomy. A third of our patients with SSc had first degree relatives with autoimmune thyroid disease. Mean TSH level was 2.24±1.18 mIU/l (normal range 0.23-4) and mean fT4 level was 1.28±1.76 (normal range 0.8-2.0 ng/dL). Out of forty patients without a known thyroid disorder 3 had mildly elevated TSH level (5.2±0.76 mIU/l), and 5 patients had positive anti-thyroid antibodies (5 were positive for aTPO and 2 were positive for both aTG and aTPO). Thyroid volume was lower than normal in 4 patients (8%). Twenty two patients (44%) had 1-6 thyroid nodules, which were 1 cm or more in 12 of them (24% of the patients). Two nodules were hypoechoic, and two others were calcified. Overall 6 patients underwent fine needle aspiration procedures: 5 were diagnosed as colloid nodules, and one as papillary carcinoma.

**Conclusions:** New cases of clinically significant autoimmune thyroid disease were not detected in our study. Nevertheless, almost half of the patients had thyroid nodules. The clinical significance of these findings and their relation to thyroid cancer in SSc patients remains to be determined.

## P.227

## SYSTEMIC SCLEROSIS- SPECTRUM DISEASES ELUCIDATED BY NAILFOLD VIDEOCAPILLAROSCOPY PATTERNS AND DIGITAL OCCLUSIVE ARTERIAL DISEASE WITH LASER DOPPLER FLOWMETRY

A. Makol^1^, Y. Radwan^1^, T. Gunderson^2^, C. Crowson^2^, A. Hinze^1^, D. Liedl^3^, P. Wennberg^3^, K. Warrington^1^

^1^*Division of Rheumatology, Mayo Clinic, MN, Rochester, USA*, ^2^*Division of Health Sciences Research, Mayo Clinic, MN, Rochester, USA*, ^3^*Division of Cardiovascular disease, Mayo Clinic, MN, Rochester, USA*

**Introduction:** While structural abnormalities of the microcirculation are best seen on nailfold videocapillaroscopy (NVC), laser doppler flowmetry (LDF) with a thermal challenge is a noninvasive means to detect digital occlusive arterial disease (DOAD). In a cohort of patients evaluated for Raynaud’s or suspected connective tissue disease (CTD), we reviewed NVC patterns and DOAD on LDF, their correlation, and their predictive value in diagnosing systemic sclerosis (SSc) spectrum diseases.

**Material and Methods:** Medical records of patients who underwent NVC and LDF at our institution between 1/2017 and 3/2019 were retrospectively reviewed. NVC results were classified as normal or abnormal (non-specific or SSc specific pattern). Presence or absence of DOAD on LDF was abstracted. Clinical diagnosis of CTDs and fulfilment of ACR/EULAR 2013 SSc classification criteria and Very early diagnosis of Systemic sclerosis (VEDOSS) criteria was ascertained.

**Results:** Among 190 patients (mean age 46 ± 15y, 81% females, 93% Caucasians) who underwent NVC and LDF, NVC was abnormal in 88 (Non-specific 31[35%], SSc pattern 57[65%]).

On LDF, 78% of patients had vasospasm and 30 (16%) had DOAD. Among 30 patients with DOAD, a CTD was diagnosed in 24 (80%) and 17 (57%) met SSc criteria. Among 30 patients with DOAD, 24 (80%) had abnormal NVC patterns with SSc NVC pattern in 18 (60%). Among 160 patients without DOAD, 93 (58%) did not have any CTD and NVC was normal in 96 (60%) patients.

SSc NVC pattern was strongly predictive of meeting VEDOSS (positive predictive value (PPV):84%) and diagnosis of CTDs (PPV:77%). Addition of DOAD to SSc NVC pattern increased the likelihood of meeting SSc criteria (PPV increased from 46% to 83%), diagnosis of CTDs (PPV increased from 77% to 100%) and slightly improved fulfillment of VEDOSS.

Having normal NVC was associated with very low likelihood of SSc diagnosis (negative predictive value (NPV):98%) and VEDOSS (NPV:91%), but did not help significantly in exclusion of other CTDs (NPV:66%). Adding DOAD did not significantly change the NPV for excluding SSc/CTDs compared to NVC alone.

**Conclusions:** DOAD on LDF was strongly associated with presence of an underlying SSc- spectrum CTD. A SSc pattern NVC in combination with DOAD was very strongly predictive of an underlying CTD, with SSc in >80% cases. A normal NVC virtually excluded the presence of SSc, but not other CTDs. Adding LDF to NVC, can further increase the predictive value of NVC alone for CTD diagnosis.

## P.228

## DIAGNOSTIC PERFORMANCES OF HAND ULTRASOUND PARAMETERS AND THEIR IMPACT ON THE 2013 ACR/EULAR CLASSIFICATION CRITERIA FOR SYSTEMIC SCLEROSIS

M. de Saint Riquier^1^, A. Ballerie^2^, F. Robin^1^, N. Belhomme^2^, C. Cazalets^2^, C. Droitcourt^3^, A. Perdriger^1^, C.M. Yelnik^4^, E. Hachulla^4^, V. Sobanski^4^, P. Jego^2^, G. Coiffier^1^, A. Lescoat^2^

^1^*CHU Rennes, Rheumatology, Rennes, France*, ^2^*CHU Rennes, Internal medicine and clinical immunology, Rennes, France*, ^3^*CHU Rennes, Dermatology, Rennes, France*, ^4^*CHRU Lille, Internal medicine and clinical immunology, Lille, France*

**Introduction:** The hand is almost always affected in Systemic Sclerosis (SSc) and five items included in the 2013 EULAR/ACR classification criteria are related to hand. Recent studies have highlighted that ultrasound (US) examination could offer a better assessment of hand manifestations of the disease. Indeed, US allows simultaneous evaluation of vascular, fibrotic and inflammatory hand features of the disease. Power Doppler US can especially explore macrovascular involvement characterized by obliteration of digital arteries or ulnar arteries. Ulnar artery occlusion (UAO) is especially frequent in SSc patients and could be a relevant marker of the severity of SSc-associated vasculopathy. Among other hand manifestations of SSc, US evaluation can notably explore tenosynovial involvement such as fibrotic tenosynovitis (TS), characterized by a US concentric alternation, of iso- and/or hyperechoic layers illustrating the fibrosis of paratendinous tissues, that is considered to be SSc-specific. This study aims to assess the diagnostic performances of these hand US parameters for the diagnosis of Systemic Sclerosis (SSc).

**Material and Methods:** 244 patients with suspected SSc were consecutively included. They all had US evaluation assessing the presence of fibrotic TS and UAO. The final diagnosis of SSc was based on the evaluation of an expert, independently from US results and from any pre-established classification criteria.

**Results:** 166 patients were finally diagnosed as SSc. 62 SSc and 8 non-SSc patients had UAO (uni or bilateral) (p=0.001). 23 SSc patients and 1 non-SSc patient had US fibrotic TS (p=0.007). A US SSc-pattern (presence of UAO and/or fibrotic TS) was reported in 73 SSc patients and 9 non-SSc patients (p<0.001). UAO had an area under ROC curve (AUC) for the diagnosis of SSc of 0.618 (95%CI 0.539-0.697); with Se= 0.373 (0.304-0.449) and spe=0.862 (0.751-0.928). The presence of a US fibrotic TS had an AUC of 0.561 (0.480-0.643); with Se= 0.139 (0.094-0.199) and spe=0.983 (0.909-0.997). The US-SSc pattern had a AUC of 0.641 (0.563-0.695), with Se=0.440 (0.367-0.516) and spe=0.845 (0.731-0.916). The original 2013 classification criteria had an AUC of 0.982 (0.969-0.996) with Se= 0.946 (0.900-0.971) and spe=0.931 (0.836-0.973). Including UAO and fibrotic TS in this classification had few impact (AUC of 0.979 (0.962-0.996) with Se= 0.940 (0.893-0.967) and and spe=0.931 (0.836-0.973)) but allows the substitution of some items (such as capillaroscopy) by US parameters with similar performances.

**Conclusions:** The use of hand US parameters may help to refine the diagnostic strategy of SSc and their inclusion in a US-modified ACR/EULAR classification could be discussed.

## P.229

## DIAGNOSING SYSTEMIC SCLEROSIS WITH PHOTOACOUSTIC AND HIGH-FREQUENCY ULTRASOUND IMAGING

B. Kersten^1^, K. Daoudi^2^, C. De Korte^2^, F. Van Den Hoogen^1^, E. Van Den Ende^1^, M. Vonk^1^

^1^*Radboud university medical center department of Rheumatology, Nijmegen, The Netherlands*, ^2^*Radboud university medical center department of Radiology and Nuclear Medicine, Nijmegen, The Netherlands*

**Introduction:** Vasculopathy is already evident in early systemic sclerosis (SSc); a Raynaud’s phenomenon and typical nailfoldcapillaroscopic findings are part of the criteria of very early diagnosis of SSc (VEDOSs). As not all early SSc patients have alterations in their nailfoldcapillaries, there is need for other diagnostic tools. Photoacoustics and high-frequency ultrasound can fulfill this need. The former can measure the oxygen saturation of hemoglobin by using short pulsed laser light while the latter can provide high-resolution images that allow measuring skin thickening distal from DIP joint, which could be used to determine skin involvement early.

We hypothesize that photoacoustics and high-frequency ultrasound can distinguish (early) SSc patients from healthy controls and subjects with primary Raynaud’s phenomenon (PR) by measuring the oxygenation of the fingertip and skin thickening.

**Material and Methods:** In our cross-sectional study, we compared measurements of the third finger in (early)SSc patients to healthy controls and subjects with PR. Smoking and beta-blockage were exclusion criteria. The level of oxygenation and skin thickness were compared between groups. Nailfoldcapillaroscopy was performed on all subjects and classified as normal/atypical or early/active/late SSc pattern.

**Results:** Thirty-one adult subjects participated in this study: twelve patients with SSc, 5 patients with early SSc, 5 volunteers with PR and 9 healthy controls.

We found a significant difference in oxygen saturation between (early)SSc patients (80.8% ± 8.1 and 77,9% ± 10.5 ) and healthy controls (94.8% ± 2.8) and subjects with PR (93.9% ± 1.1) . No significant difference was found between healthy controls and subjects with PR . (figure 1 A)

Measurements of skin thickening showed a significant difference in (early) SSc patients compared to healthy controls and subjects with PR (0.48 ± 0.06 mm and 0.51 ±0.16 mm vs. 0.316 ± 0.6 mm and 0.27 ± 0.01 mm). There was no significant difference between subjects with PR and healthy volunteers. (figure 1 B)

**Conclusions:** Our results demonstrate that photoacoustic and high-frequency ultrasound can distinguish between (early)SSc, PR and healthy individuals in both oxygenation saturation and skin thickening.

## P.230

## ULTRASOUND MEASUREMENT OF THE NAIL BED MATRIX THICKNESS AS A USEFUL MARKER FOR SCLERODERMA-RELATED INTERSTITIAL LUNG DISEASE

V. Hsu^1^, M. Sidor^1^, R. Zuckerman^1^, N. Schlesinger^1^, R. Sussman^2^, S. Huma^1^

^1^*Rutgers-Robert Wood Johnson Medical School, New Brunswick, USA*, ^2^*University Radiology Group, East Brunswick, USA*

**Introduction:** Lung involvement is the leading cause of death in systemic sclerosis (SSc. Pulmonary function tests (PFTs) and chest high-resolution CT scan (HRCT) are used to confirm this common complication.

We performed high-resolution musculoskeletal (MSK) ultrasound of the nail-bed to assess digital clubbing and attempted to correlate with the presence of scleroderma-related interstitial lung disease (SSc-ILD).

Since Hippocratic times, digital clubbing has been described in patients with hypertrophic osteoarthropathy, associated with chronic illnesses, including pulmonary diseases. Digital clubbing is associated with abnormal proliferation of skin and periosteal tissues of the fingertips characterized by periostosis of tubular bones, uniform swelling of soft tissues of terminal phalanx, and increased nail-to-nail bed angle. The increased nail bed matrix thickening (NBMT) is associated with digital clubbing, normal reported 01.7+/- 0.18mm (see figure 1). We hypothesize that measurement of the NBMT by MSK ultrasound can be an effective marker for clinically significant SSc-ILD.

**Material and Methods:** In this IRB-approved study, we evaluated the dominant hand of 51 subjects by MSK ultrasound. 33 patients met 2013 ACR criteria for SSc and were divided in two groups ( with ILD and without ILD);18 subjects served as the age-matched control group (no SSc).

Using the Phillips Epiq 5, all subjects underwent MSK ultrasound of the dominant hand. The NBMT of all five fingers were examined and a total of 235 nail beds were recorded. SSc-ILD was confirmed by HRCT associated with restrictive pattern on the PFT. We used ANOVA testing to calculate the differences between groups and p<0.05 was statistically significant.

**Results:** Table 1 shows significantly different NBMT for the 2nd, 3rd and 4th fingers in the SSc lung cohort, and inter-digital analysis determined that the third finger NBMT had the highest differences between the fingers. The mean cutoff value for normal NBMT in the third finger was less than 2.27 mm. Table 2, showed a trend towards increased NBMT in those with SSc-ILD, although only %DLCO was significant in our small cohort.

**Conclusions:** Our study suggests common mechanisms that may responsible for digital clubbing in SSc result from chronic local tissue hypoxia due to chronic lung disease. This is readily visible by MSK ultrasound, often before it is palpable on physical exam and may be a predictor of more clinically significant pulmonary disease. We found that the NMBT of 2.27 mm or greater for the third finger was associated with the presence of SSc-ILD. Larger studies are needed to verify our findings.

**Figure:**
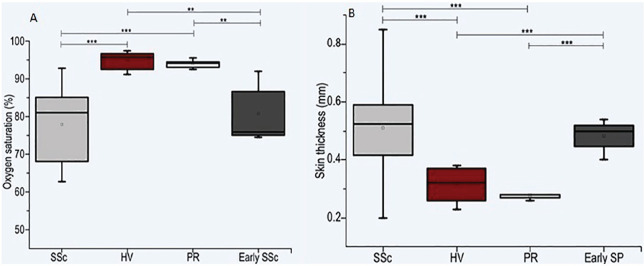


## P.231

## DIGITAL ARTERY VOLUME INDEX (DAVIX©) PREDICTS THE ONSET OF FUTURE DIGITAL ULCERS IN PATIENTS WITH SYSTEMIC SCLEROSIS

K. Gjeloshi^1,2^, F. Danzo^1,2^, G. Lettieri^2,3,4^, G. Abignano^2,4,5^, M. Hinton^6^, A.-M. Dean^2,4^, G. Cuomo^1^, O. Kubassova^6^, F. Del Galdo^2,4^

^1^*Complex Operative Unit (UOC) Internal Medicine Department, University of Campania ‘‘Luigi Vanvitelli’’, Napoli, Italy*, ^2^*Leeds Institute of Rheumatic and Musculoskeletal Medicine, University of Leeds, Leeds, United Kingdom*, ^3^*Radiology Department, San Carlo Hospital, Potenza, Italy*, ^4^*NIHR Leeds Biomedical Research Centre, Leeds Teaching Hospitals NHS Trust, Leeds, United Kingdom*, ^5^*Rheumatology Institute of Lucania (IReL), Rheumatology Department of Lucania, San Carlo Hospital, Potenza, Italy*, ^6^*IAG CEO, London, United Kingdom*

**Introduction:** Digital Ulcer Disease is a main vascular manifestation of Systemic Sclerosis (SSc) caused by progressive narrowing of digital arteries with consequent tissue ischemia. Time of flight angiography (TOF) is an MRI technique based on flow-related enhancement of spins entering into an imaging slice. It can visualise flow within vessels, without the need to administer contrast. Here we measured Digital Artery Volume index (DAVIX ©) as an MRI TOF quantitative score of digital arteries flow and determined its value in predicting the onset of digital ulcers (DUs) in patients with SSc.

**Material and Methods:** We studied 91 consecutive patients, 63 of which fulfilled the 2013 ACR/EULAR classification criteria for SSc and 28 had a score <9. The data collected included: clinical examination, pulmonary function tests (PFTs), echocardiography, nailfold capillaroscopy. DAVIX of the dominant hand was calculated as % mean of the 4 fingers, employing MeVisLab software. The distribution was analysed with D’Agostino-Pearson normality test. Correlation with clinical parameters was performed using Spearman’s or Pearson test, as appropriate (Prism 7).

**Results:** 78/91 patients were females and median disease duration was 4 years (IQR 1.91-9). Complete historical and prospective follow-up data were available for 68 patients. At baseline 7 patients had DUs (5 with a positive history for DUs). 12 patients developed DUs within 12 months, 3 of them had DUs at baseline. 38 patients did not have either previous or current DUs, neither did they develop new DUs within 12 months. The median of DAVIX in this population was 0.65 (IQR 0.46-0.82). DAVIX of patients with current DUs was 3-fold lower than DAVIX of patients without DUs (0.18 vs 0.63 p=0.0093). Further, DAVIX of patients with positive history of DUs was 50% lower than in patient with a negative history (median 0.34 vs 0.64, p=0.0052). In patients without current DUs who developed new DUs within 12 months of follow-up the DAVIX was 3-fold lower than in patients who didn’t develop (0.21 vs 0.65, p=0.0156). Most importantly in patients with no current DUs a DAVIX <0.47 gave a 35% risk of developing DU ( vs 15% overall risk).

**Conclusions:** Outcome measures of vascular involvement in SSc are scanty. We demonstrated that DAVIX© is a promising and feasible surrogate outcome measure of neointima proliferation in SSc. Furthermore, the predictive value of DAVIX for the future onset of DU could be employed as a useful stratification tool in Clinical trials.

**Figure:**
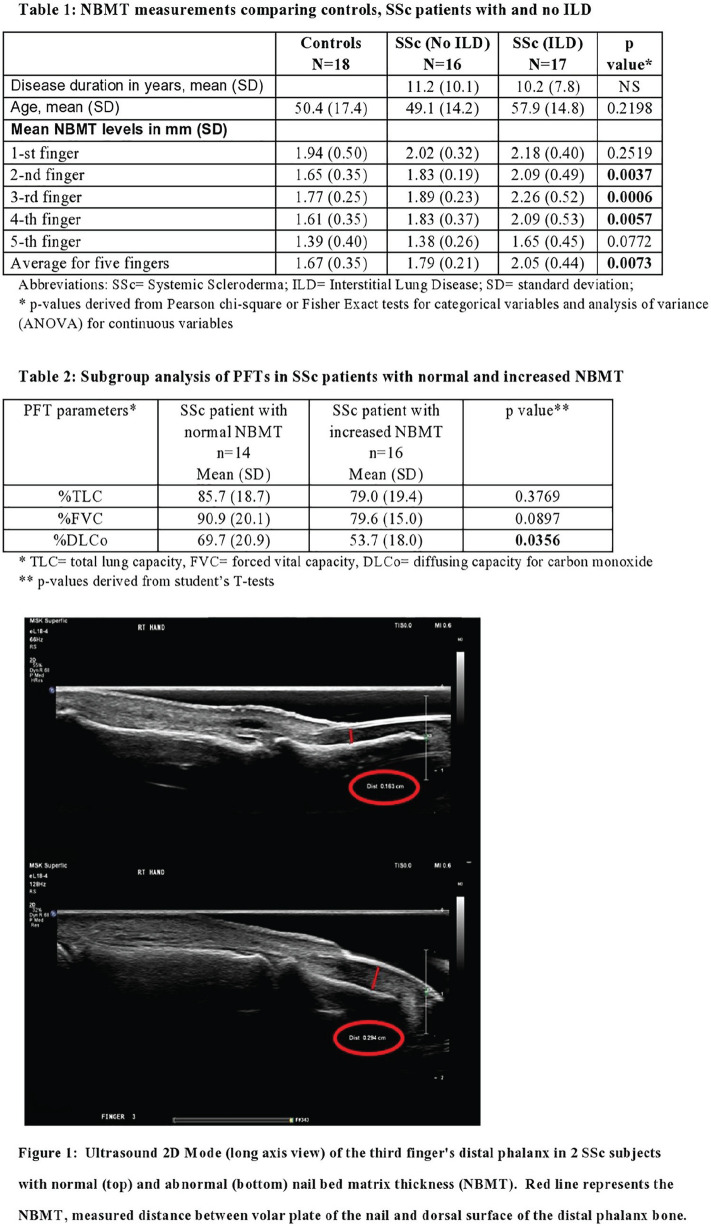


## P.232

## EXAMINATION OF THE VALIDITY OF SKIN ULTRASOUND TO QUANTITATE SKIN INVOLVEMENT FOR MULTI-CENTER CLINICAL TRIALS IN PATIENTS WITH SYSTEMIC SCLEROSIS (SSC)

O. Aly^1^, K. Kafaja^1^, D. Furst^4^

^1^*Pacific Arthritis, 1-2, Los Angeles, USA*, ^2^*University of Southern California, Los Angeles, USA*, ^3^*University of California, Los Angeles, Los Angeles, USA*, ^4^*University of Washington, Seattle, Seattle, USA*, ^5^*University of Florence, Florence, Florence, Italy*

**Introduction:** Introduction There have been a great deal of data published on ultrasound of the skin. However, the validity of ultrasound of the skin has not been established.

Objectives Our objective was to conduct a systematic literature review of ultrasound of the skin in systemic sclerosis (SSc) patients to establish the degree to which ultra-sound of the skin (for use in multi-center clinical trials) has been validated, using the OMERACT Filter.

**Material and Methods:** Methods We conducted a Systematic Literature Review (SLR) using two databases (PubMed and Cochrane library) to examine the validity of ultrasound to quantitate skin involvement of SSc patients. The SLR was done between 1950 and 31 December 2018.

Inclusion criteria were: (1) English; (2) patients met the criteria for the 1980 or 2013 classifications for SSc (i.e. limited or diffuse SSc); (3) studies were either randomized controlled trials, observational studies or case studies involving more than 20 patients; (4) patients age >=18 years; (5) if the article contained a mixed patient population, the SSc patients and results were separable; (6) the ultrasound machine was clearly described.

Exclusion criteria were: (1) not in English; (2) data did not record at least one of the validation criteria; (3) age <18 years of age; (4) patients had other than limited or diffuse SSc (e.g. localized scleroderma or Scleroderma-like disease); (5) letters to the editor/editorials; (6) mRSS <2.

All extractions were double extracted. Any disagreements were resolved by consensus or adjudication by D.E.F. Descriptive statistics were generated for each criterion.

**Results:** Results From an initial, 292 citations, 17 articles (approximately 1100 pts) met inclusion/exclusion criteria and were double extracted. The status of validation of ultrasound is seen in Table 1.

**Conclusions:** Conclusion Based on an SLR through December 31, 2018, ultra-sound of the skin met some but not all validation criteria for use in multi-center, clinical trials... Specifically, discrimination, reproducibility and feasibility (i.e. feasible among centers for a multi-center clinical trial) criteria were not met. Further, the construct validity criterion was only partially met, the validation of ultrasound of the skin requires further research if it is to be used as a fully validated measure in clinical trials.

**Figure:**
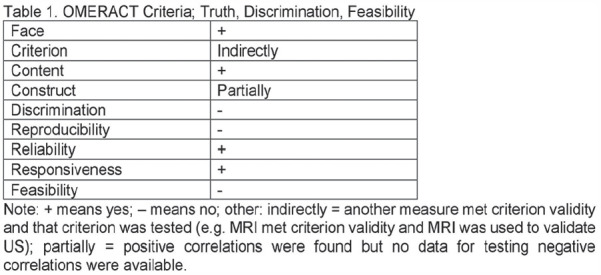


## P.233

## NAILFOLD CAPILLAROSCOPY IN THE SPANISH GROUP OF SYSTEMIC AUTOIMMUNE DISEASES (GEAS). RESULTS OF AN ELECTRONIC SURVEY

P. Fanlo^1^, L. Saez^2^, B. Gracia^3^, M. Freire^4^, J.A. Todoli^5^, B. Marí^6^, G. Espinosa^7^, F. Garcia^8^, A. Guillén^9^, N. Ortego^10^, J.J. Rios^11^, A. Selva^9^, V. Fonollosa^9^

^1^*UEAS. Complejo Hospitalario de Navarra, Pamplona, Spain*, ^2^*UEAS. Hospital Miguel Servet, Zaragoza, Spain*, ^3^*UEAS. Hospital Clinico lozano Blesa, Zaragoza, Spain*, ^4^*UEAS. Complejo Hospitalario Universitario de Santiago, Santiago de Compostela, Spain*, ^5^*UEAS. Hospital La Fe, Valencia, Spain*, ^6^*UEAS. Corporación Sanitaria Parc Taulí, Barcelona, Spain*, ^7^*Servicio de EAS. Hospital Clínic, Barcelona, Spain*, ^8^*Unidad de Colagenosis. Hospital Universitario Virgen del Rocío, Sevilla, Spain*, ^9^*UEAS.Hospital Universitari Vall d’Hebron, Barcelona, Spain*, ^10^*UEAS. Hospital Universitario San Cecilio, Granada, Spain*, ^11^*UEAS. Hospital La Paz, Madrid, Spain*

**Introduction:** A survey about nailfold capillaroscopy was designed to obtain information about the studies they perform, their learning methods, and concordance between interpretation of capillaroscopic images and patterns.

**Material and Methods:** Cross-sectional study by electronical survey sent to all Internists of the Spanish Group of Autoimmune Diseases (GEAS) between January-February 2019, including questions about experience, learning methods and devices used in capillaroscopy. The survey included 10 capillaroscopic images for interpretation, subsequently scored from 0 to 4 points. Univariate analysis was performed to study associations with the global result in capillaroscopic interpretation. Reliability indexes were used to assess consistence between the answers.

**Results:** 77 responses out of 1011 GEAS members were obtained (7.6%), from 63 Hospitals in Spain. The responders reported a mean of 11.97 years of experience as internists (range 1-38) and 8.82 years (range 1-32) performing capillaroscopic studies. 43.3% of them usually performed at least 1 capillaroscopy weekly, and 46.8% at least 25 in a year. Their learning methods were colleagues (67.5%), courses (79.2%), the GREC mobile app (32.5%) and self-learning (35.1%). The USB capillaroscope was the device mostly used (42.9%). A success rate of 58% was achieved in interpretation of morphologic alterations, 70.13% in capillary count and 63,2% in capillaroscopic patterns. The global score in capillaroscopic interpretation showed a mean value of 24,79 points out of 40 (range 12-37). This result was associated with the number of capillaroscopic studies performed and with the GREC Study Group. Low reliability indexes were obtained (Fleiss’s Kappa 0.152 and Krippendorff’s alpha 0.106).

**Conclusions:** Capillaroscopy is performed by few GEAS member. Education and accreditation programs in capillaroscopy are needed.

## P.234

## FIRST PILOT STUDY OF T1 MAPPING MRI OF PERIPHERAL MUSCLE IN SYSTEMIC SCLEROSIS PATIENTS SHOWS DIFFUSE FIBROSIS THAT MAY ASSOCIATE WITH SUBCLINICAL CARDIAC INVOLVEMENT

R.-B. Dumitru^1^, A.F. Goodall^2,3^, D.A. Broadbent^2^, A. Kidambi^4^, S. Plein^4^, F. Del Galdo^1^, A.-L. Tan^1^, J. Biglands^2^, M.H. Buch^1,5^

^1^*Leeds Institute of Rheumatic and Musculoskeletal Medicine, University of Leeds, Leeds, United Kingdom*, ^2^*Medical Physics and Engineering, Leeds Teaching Hospitals NHS Trust, Leeds, United Kingdom*, ^3^*Medical Imaging and Medical Physics, Sheffield Teaching Hospital Trust, Sheffield, Sheffield, United Kingdom*, ^4^*Department of Biomedical Imaging Science, Leeds Institute of Cardiovascular and Metabolic Medicine, University of Leeds, Leeds, United Kingdom*, ^5^*Division of Musculoskeletal & Dermatological Sciences, University of Manchester & NIHR Manchester Biomedical Research Ce, Manchester, United Kingdom*

**Introduction:** Peripheral myopathy is a significant cause of disability in systemic sclerosis (SSc) but remains poorly defined. Inflammatory myositis is associated with poor prognostic factors and myocarditis, but there is little understanding of the clinical association of non-inflammatory myopathy. T1 mapping is applied in cardiovascular magnetic resonance (CMR) to detect diffuse fibrosis. We evaluated the feasibility of T1-mapping in peripheral muscle of SSc patients with and without myopathy and we explored for an association between cardiac and peripheral muscle T1-mapping in SSc.

**Material and Methods:** SSc patients, fulfilling the 2013 ACR/EULAR criteria, with no cardiovascular disease or myositis and healthy volunteers (HV) underwent peripheral muscle T1 mapping-MR for native T1 and extracellular volume (ECV) quantification. Patients also had T1-mapping CMR and creatine-kinase (CK) measured. Non-inflammatory myopathy was defined as current/history of minimally raised CK (<600 IU/l) +/- presence of clinical signs-symptoms (including proximal myasthenia and/or myalgia) +/- a Manual Muscle Testing (MMT) score <5 in the thighs.

**Results:** 12 SSc patients and 10 HV were recruited. SSc patients had a median (IQR) age of 52 (41,65) years, 9/12 had limited cutaneous SSc, 4/12 interstitial lung disease, 7/12 non-inflammatory myopathy. Higher skeletal muscle ECV was recorded in SSc patients compared to HV [mean (SD) 23(11)%, vs 11(4)% p=0.04]. Skeletal muscle native T1 were comparable between the 2 groups although modestly higher in SSc patients [mean (SD) 1396ms (56) vs 1387ms (42)](Figure 1A).

Peripheral muscle ECV associated with CK (R² =0.307, rho=0.554, p=0.0615) and was higher in SSc patients with evidence of myopathy compared to those with no myopathy [28 (10)% vs 15 (5)%, p=0.023] (Figure 1B). An ECV of 22% was determined to best identify myopathy with a sensitivity of 71% and a specificity of 80%.

SSc patients had raised myocardial ECV and native T1 with means (SD) of 31% (3) and 1287ms (54) respectively (normal reference range ECV <29%, native T1 >1240ms). A trend for an association between peripheral muscle native T1 and myocardial ECV was demonstrated (rho=0.470, p=0.123).

**Conclusions:** This first study to implement T1-mapping MR of the peripheral muscle showed markedly higher ECV in SSc patients compared to HV, suggesting the presence of diffuse fibrosis in the peripheral muscle of SSc patients, which may represent the substrate for the non-inflammatory myopathy. An association between myocardial ECV and peripheral muscle native T1 was suggested, implying the presence of interstitial remodelling in both the heart and peripheral muscle. Larger studies are needed to confirm these findings.

**Figure:**
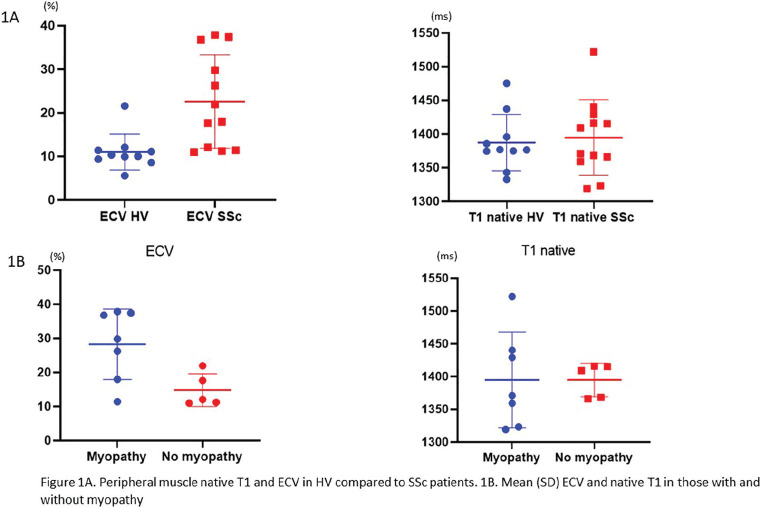


## P.235

## SEVERITY OF HAND VASCULAR INVOLVEMENT AS ASSESSED BY MRI DIGITAL ARTERY VOLUME INDEX (DAVIX©) PREDICTS WORSENING OF CLINICAL PARAMETERS AND PATIENT REPORTED OUTCOMES IN SCLERODERMA

F. Danzo^1,2^, K. Gjeloshi^1,2^, G. Lettieri^3^, G. Abignano^1,4,5^, M. Hinton^5^, A.-M. Dean^1^, G. Cuomo^2^, O. Kubassova^6^, F. Del Galdo^1,4^

^1^*Leeds Institute of Rheumatic and Musculoskeletal Medicine, University of Leeds, Leeds, United Kingdom*, ^2^*Complex Operative Unit (UOC), Internal Medicine Department, University of Campania Luigi Vanvitelli, Napoli, Italy*, ^3^*Radiology Department, San Carlo Hospital, Potenza, Italy*, ^4^*NIHR Leeds Biomedical Research Centre, Leeds Teaching Hospitals NHS Trust, Leeds, United Kingdom*, ^5^*Rheumatology Institute of Lucania (IRel),Rheumatology Department of Lucania, San Carlo Hospital, Potenza, Italy*, ^6^*IAG (Image Analysis Group), London, United Kingdom*

**Introduction:** Neointima proliferation is a key pathologic feature of Systemic Sclerosis (SSc), causing arterial vessel narrowing and being the recognised culprit pathological lesion in Digital Ulcers (DUs), pulmonary artery hypertension and renal crisis. Nevertheless, there are no validated imaging techniques to assess the severity of vascular involvement in SSc. We have previously shown digital artery volume index (DAVIX©) assessed with time of flight MRI angiography, is a reliable measure of neointima proliferation in the hands. The purpose of our study was to identify the value of DAVIX© in predicting worsening of patient reported outcomes (PROs) and clinical parameters in SSc.

**Material and Methods:** Cross-sectional data were available for 91 patients and complete 12 months follow-up data for 68 patients. Data collected included: modified Rodnan Skin Score (mRSS), Pulmonary Function Tests (PFTs), echocardiography, nailfold capillaroscopy, Health Assessment Questionnaire Disability Index (HAQ-DI), and Scleroderma Health Assessment Questionnaire (sHAQ). DAVIX© of the dominant hand was calculated as the %mean of the 4 fingers, employing MeVisLab software. Following analysis of distribution, Spearman or Pearson test were used to determine correlation coefficients, as appropriate (Prism 7).

**Results:** 56/68 were female and median of disease duration was 4 years (IQR 1.91-9). As previously reported DAVIX© correlated with the presence of DUs (p=0.0093). Considering all patients, DAVIX© correlated with mRSS (r=-0.258, p=0.017), DLCO% (r=0.338, p=0.008) and the pattern of capillaroscopy (r=-0.388, p=0.001). In patients with DUs, DAVIX© showed a stronger correlation with DLCO% (r=0.786, p=0.048). Most importantly, DAVIX© predicted the worsening of HAQ-DI (r=-0.295, p=0.029), sHAQ (r =-0.333, p=0.029) and VAS pain (r=-0.269, p=0.038) independently of the presence of DUs.

**Conclusions:** The quantitative assessment of neointima proliferation in the hand by DAVIX© is a useful imaging biomarker of vascular disease activity. The value of DAVIX© in predicting the worsening of PROs and clinical parameters in overall patients, may offer insights on the role of vascular disease activity in the global progression of SSc. The validation of our data in an independent cohort and the sensitivity to change over time of DAVIX© may aid to the implementation of hand MRI as imaging outcome measure of vascular severity in SSc.

## P.236

## DEEP LEARNING FOR CALCINOSIS CUTIS QUANTIFICATION

A. Chandrasekaran^1^, Z. Fu^2^, A. Wang^1^, S. Ren^3^, R. Kraniski^1^, I. Omar^4^, M. Hinchcliff^1^

^1^*Yale School of Medicine, New Haven, USA*, ^2^*Illinois Institute of Technology, Chicago, USA*, ^3^*San Diego State University, San Diego, USA*, ^4^*Northwestern University Feinberg School of Medicine, Chicago, USA*

**Introduction:** Calcinosis cutis (CC), a common systemic sclerosis (SSc) complication, can be extensive and debilitating. There are potential treatments, but a standardized and validated method for precise whole-body CC burden quantification is necessary to enable valid clinical trials. Dual energy computed tomography (DECT) can differentiate between CC and adjacent healthy bone, but radiologists must manually quantify the irregularly shaped lesions, which can be imprecise, time-consuming, and costly. Computer vision deep learning techniques, including convolutional neural networks (CNN), have been successfully applied to solve problems in clinical medicine. The aim of this study is to optimize a deep learning model to facilitate quantification of CC disease burden in the fingers of SSc patients.

**Material and Methods:** De-identified 2-dimensional (2-D) DECT images from SSc patients with clinically apparent CC in the hands were obtained and viewed using Visage Imaging 7 (San Diego, CA). As a pilot study, 30 images with CC in the forefinger were studied. An expert musculoskeletal radiologist manually segmented (identified) the three forefinger phalanges as a gold standard reference for the computer vision algorithm (Image 1). Then, computer scientists trained a U-Net CNN model for forefinger bone segmentation to permit CC visualization. Following finger bone segmentation optimization, computer vision was used to measure the number of pixels that contained CC on each 2-D image. A radiologist measured the area of the lesions per standard of care, recording the time for each measurement.

**Results:** To obtain area estimates for each lesion, the radiologist manually traced the circumference (Image 2), a task that required on average 4 minutes per study. In contrast, computer scientists allowed the computer algorithm to calculate CC area, a task that required <0.9 seconds per study. Via a paired T-test, the means of the radiologist- vs. computer-calculated areas were calculated, which were 27.5 mm2 and 24.8 mm2, respectively [t(29)=-4.10, p=0.0003]. However, the standardized mean difference was only 0.21, indicating that the differences, although statistically significant, are unlikely to be clinically relevant.

**Conclusions:** To our knowledge, the present study is the first to utilize deep learning for CC quantification. We demonstrate that a deep learning model applied to DECT hand images of SSc patients can be trained to differentiate CC lesions from adjacent healthy bone. When compared to gold standard radiologist area estimates, the computer vision method overestimated the area, but the difference is not clinically significant, and the deep learning method was faster. Future work will include measurements of lesional volume and density.

**Figure:**
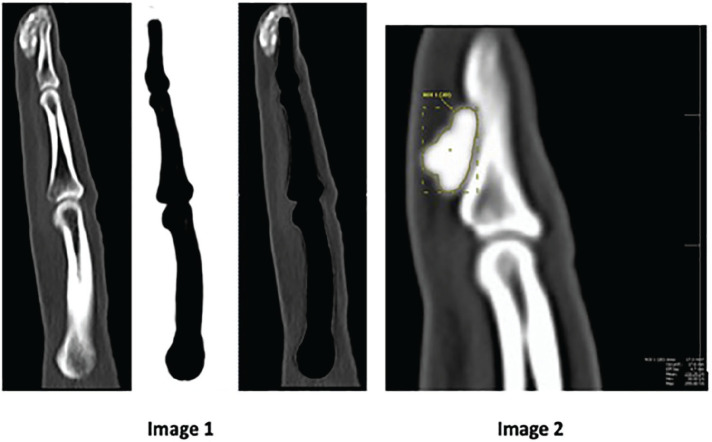


## P.237

## VISUALISATION OF THE ACTIVE CALCIFICATION PROCESS WITH 18-F SODIUM FLUORIDE PET/CT IN LIMITED CUTANEOUS SYSTEMIC SCLEROSIS WITH CALCINOSIS CUTIS IS FEASIBLE: A PILOT STUDY

G.M. Swart^1^, I.M. Atzeni^1^, E.M. Hogervorst^1^, K. de Leeuw^2^, M. Bijl^3^, R. Bos^4^, J. Westra^2^, G.F.H. Diercks^5^, H. van Goor^5^, M.C. Bolling^6^, R.H.J.A. Slart^7^, D.J. Mulder^1^

^1^*Department of Internal Medicine, division Vascular Medicine; University of Groningen; University Medical Center, Groningen, The Netherlands*, ^2^*Department of Rheumatology & Clinical Immunology; University of Groningen; University Medical Center, Groningen, The Netherlands*, ^3^*Department of Rheumatology; Martini hospital, Groningen, The Netherlands*, ^4^*Department of Rheumatology; Medical Center of Leeuwarden, Leeuwarden, The Netherlands*, ^5^*Department of Pathology & Medical Biology; University of Groningen; University Medical Center, Groningen, The Netherlands*, ^6^*Department of Dermatology; University of Groningen; University Medical Center, Groningen, The Netherlands*, ^7^*Medical Imaging Center, Department of Nuclear Medicine & Molecular Imaging; University Medical Center, Groningen, The Netherlands*

**Introduction:** Calcinosis cutis is a major daily challenge to patients with longstanding systemic sclerosis (SSc), negatively affecting their quality of life. Unfortunately, treatment options are very limited due to lack of understanding of the pathogenetic process. Currently, calcinosis cutis is only detected at its irreversible end-stage. Early detection of calcinosis cutis could putatively allow early disease-modifying interventions and monitor treatment effects. The aim of the current study is to assess the feasibility of visualising “active” micro-calcifications with 18-F Sodium Fluoride (NaF) PET scanning, compared to low-dose CT in patients with clinically overt calcinosis cutis.

**Material and Methods:** This was a cross-sectional, observational, pilot study. All patients met 2013 ACR/EULAR criteria for SSc. Patients underwent a whole body NaF PET/low-dose CT scan, scanned 90 minutes post-injection. (Sub)cutaneous calcifications were described and assessed on NaF PET, which was compared to CT images by two independent investigators.

**Results:** A total of 10 female patients with limited cutaneous SSc [median age 56 years (IQR 52-66), median disease duration 17 years (8-19), PAH 10%, ILD 20%] were included, and compared to 10 controls [70 years (65-73)]. NaF uptake showed normal distribution throughout the skeletal bones, arterial tree, and visceral organs, which was comparable between patients and controls. Additionally, NaF uptake was visible in the skin of all SSc patients, but in none of the controls. Cutaneous NaF uptake largely correlated with clinical calcifications. Most common sites of cutaneous NaF uptake were fingers (5 patients) and knees (6 patients). Only 5% of the NaF positive lesions were not accompanied by visible calcifications on CT. Furthermore, of all calcified lesions seen on CT, 51% showed uptake on NaF PET. Small lesions (<1 cm), were generally only visible on CT, due to lower resolution of NaF PET.

**Conclusions:** Imaging of “active” calcinosis cutis in limited cutaneous systemic sclerosis is feasible using NaF PET scanning. Most clinically overt calcifications and half of those seen on CT were positive for NaF uptake. Whether these “active” calcifications behave differently in terms of faster progression, clinical complaints, and infection risk, and whether these are potentially suitable for disease modifying interventions is subject to future study.

**Figure:**
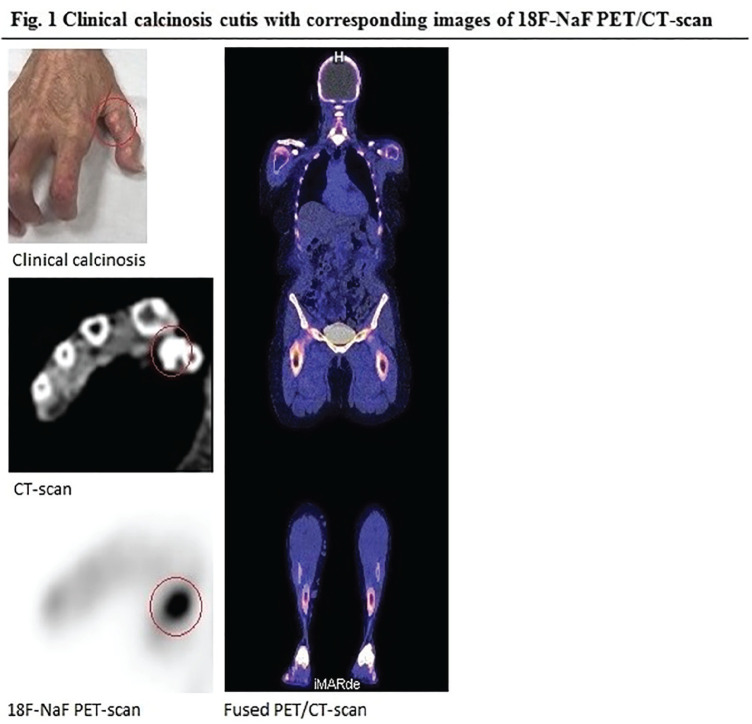


## P.238

## CHANGE IN CALCINOSIS OVER 1 YEAR USING THE SCTC RADIOLOGIC SCORING SYSTEM FOR CALCINOSIS OF THE HANDS IN PATIENTS WITH SYSTEMIC SCLEROSIS

V. Antonia^1^, M. Chung^2^, T. Rodriguez-Reyna^3^, S. Proudman^4^, M. Baron^5^, F. Castelino^6^, V. Hsu^7^, S. Li^8^, D. Fiorentino^8^, K. Stevens^9^, L. Chung^10^

^1^*Pontificia Universidad Católica de Chile, Department of Immunology and Rheumatology, Santiago, Chile*, ^2^*Stanford University School of Medicine, Division of Rheumatology, Palo Alto, Usa*, ^3^*Instituto Nacional de Ciencias Médicas y Nutrición Salvador Zubirán, Department of Immunology and Rheumatology, Mexico City, Mexico*, ^4^*Royal Adelaide Hospital North Terrace, Rheumatology Unit, Discipline of Medicine, University of Adelaide, Adelaide, Australia*, ^5^*Division of Rheumatology, Jewish General Hospital, McGill University, Montreal, Canada*, ^6^*Harvard Medical School, Division of Rheumatology, Boston, USA*, ^7^*Rutgers-RWJ Medical School, Rheumatology Division, New Jersey, Usa*, ^8^*Stanford University School of Medicine, Department of Dermatology, Palo Alto, USA*, ^9^*Stanford University, Division of Radiology, Palo Alto, USA*, ^10^*Stanford University School of Medicine and Palo Alto VA Health Care System, Department of Immunology and Rheumatology an, Palo Alto, USA*

**Introduction:** Calcinosis cutis is a debilitating complication of systemic sclerosis (SSc). We previously developed a radiographic scoring system to assess severity of calcinosis affecting the hands in patients with SSc. We sought to further validate our radiographic scoring system to assess for change over 1 year and to identify factors associated with improvement or progression.

**Material and Methods:** Baseline and 1-year antero-posterior hand radiographs were obtained in 39 SSc patients with calcinosis prospectively enrolled at 6 centers within the US, Canada, Mexico and Australia. Two blinded readers (one radiologist and one rheumatologist) scored all radiographs using the calcinosis scoring system and, on the 1 year set, a 5-point Likert scale (1=A lot better, 2=A little better, 3=No change, 4=A little worse, 5=A lot worse). By maximizing the Kappa coefficient of agreement between grouped Likert scale (better/no change/worse) and the percentage of change of calcinosis in the radiographic scoring system, we defined progressive calcinosis as >25% increase in score from baseline at 1 year, stable calcinosis as change in score between -25% to 25%, and improvement of calcinosis as decrease in score by >25%.

**Results:** Inter-rater reliability of the calcinosis scoring system was high with intra-class correlation coefficient of 0.93 (0.89-0.95). The median percentage of change from baseline to 1 year was 12.8% (range -89.3-290.2%). Sixteen patients (41%) experienced progression of calcinosis over 1 year; 18 (46%) remained stable; and 5 (13%) had improvement. Patients with progressive calcinosis had lower baseline modified Rodnan skin score (mRSS) (3.8 vs. 5.9, p=0.057), lower T-score on bone densitometry (-3.3 vs -1.7, p=0.043), and higher prevalence of loss of digital pulp on physical exam than patients who did not progress (56% vs 22%, p= 0.027). Patients whose calcinosis improved had greater prevalence of digital pitting scars (71% vs 20%, p=0.046), and trends toward having less gastrointestinal disease (40 vs. 82%, p=0.070) and a higher frequency of antibodies against PM-Scl (50 vs. 6%, p=0.09) than patients whose calcinosis did not improve. In multivariable analysis, loss of digital pulp remained a predictor of calcinosis progression (OR 5.8, p=0.023).

**Conclusions:** We confirmed the excellent inter-rater reliability of our radiographic calcinosis scoring system, and, for the first time, its usefulness to detect change over time. We found that loss of digital pulp was predictive of progressive calcinosis. This provides further evidence that digital ischemia contributes to the progression of calcinosis. More research is needed to confirm the association between PM-Scl positivity and improvement of calcinosis.

**Figure:**
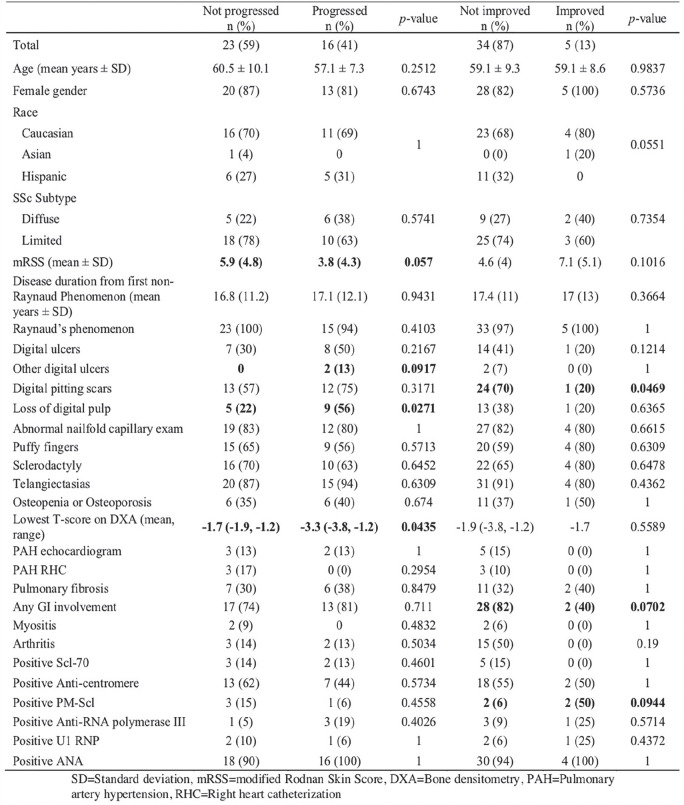


## P.239

## COLOUR DOPPLER ULTRASONOGRAPHY IN DETECTION OF DIGITAL VASCULOPATHY IN SYSTEMIC SCLEROSIS

O. Alekseeva, N. Yudkina, A. Volkov

*V.A. Nasonova Research Institute of Rheumatology, Moscow, Russia*

**Introduction:** Systemic sclerosis (SSc) can lead to ischemic complications such as digital ulcers or pitting scars (DU/PS). The aim of the study was to compare blood flow parameters in SSc patients and healthy individuals and to compare with nailfold capillaroscopy and clinical signs of ischaemia (digital ulcers or pitting scars (DU/PS).

**Material and Methods:** 32 SSc patients, mean age 49,5 [42,0; 59,0] yrs, were included. A total of SSc patients and 15 ‘healthy’ controls underwent Colour Doppler ultrasonography (CDUS) of 376 (256+120) digital arteries to compare blood flow velocity, resistive indices (RIs) and presence of occlusion. Nailfold capillaroscopy, clinical and laboratory data were also evaluated.

**Results:** In digital arteries, pulsatility index (PI), peak systolic velocity (PSV) and end-diastolic velocity (EDV) were significantly lower and RI higher in SSc patients compared with controls (PSV: 13,28[9,88; 16,7] vs14,1[11,45; 17,3] cm/s, p=0,4; EDV: 2,68 [1,78, 4,05] vs 5,0 [2,6; 7,3] cm/s, p=0,02; RI: 0,78 [0,69; 0,81] vs 0,66 [0,56; 0,72], p=0,01; PI: 1,73 [1,32; 2,19] vs 1,23 [0,87; 1,49], p=0,01).

We did not find any correlation between two methods. This could be due to the small group of patients included in this study.

**Conclusions:** Blood flow is significantly decreased in digital arteries in SSc. CDUS is very important in general vascular diagnostic procedures. Its role in determining the extent of vasculopathy in SSc needs further investigation.

## P.240

## CROSS-VALIDATION AND COMPLEMENTARY VALUE OF OPTICAL COHERENCE TOMOGRAPHY AND HIGH FREQUENCY ULTRASOUND FOR THE ASSESSMENT OF SKIN DISEASE IN SYSTEMIC SCLEROSIS

D. Temiz Karadag^1,2^, G. Lettieri^3^, V. Picerno^1^, O. Gundogdu^4^, M.C. Padula^1^, G.A. Mennillo^1^, A.A. Padula^1^, F. Del Galdo^5,6^, S. D’Angelo^1^, G. Abignano^1,5,6^

^1^*Rheumatology Institute of Lucania (IReL), Rheumatology Department of Lucania, San Carlo Hospital, Potenza, Italy*, ^2^*Department of Rheumatology, Kocaeli University, Kocaeli, Turkey*, ^3^*Radiology Department, San Carlo Hospital, Potenza, Italy*, ^4^*Department of Biomedical Engineering, Kocaeli University, Kocaeli, Turkey*, ^5^*Leeds Institute of Rheumatic and Musculoskeletal Medicine, University of Leeds, Leeds, United Kingdom*, ^6^*NIHR Leeds Biomedical Research Centre, Leeds Teaching Hospitals NHS Trust, Leeds, United Kingdom*

**Introduction:** The modified Rodnan skin score (mRSS) is the current gold standard for skin assessment in systemic sclerosis (SSc) both in clinical trials and practice. Several studies have reported that skin high frequency ultrasound (HFUS) and, more recently, optical coherence tomography (OCT) are able to reflect the severity of the skin disease in SSc. Aim of the study was to compare OCT and HFUS in the assessment of skin involvement in SSc.

**Material and Methods:** Dorsal forearm skin of consecutive diffuse cutaneous SSc (dcSSc) patients and matched-healthy controls (HC) were scanned using OCT and HFUS by investigators blinded to the clinical details using Vivosight scanner (1 assessor) and Esaote MyLab70 equipped with a 22 MHZ probe (2 assessors) respectively. Minimum Optical Density (MinOD), Maximum OD (MaxOD) and OD at 300 micron-depth (OD300) (OCT) and skin thickness (HFUS) were measured. Clinical involvement was assessed by a blinded operator using the mRSS and results were cross matched with imaging data. Statistical analysis was performed using GraphPad Prism software V.7.0.

**Results:** A total of 88 OCT images and 176 HFUS images were obtained from 22 dcSSc patients [20 female, mean age 49 (±11) years, 12 with < 5 years disease duration) and 22 HC (20 female, mean age 50.7 (±6.7) years]. All OCT measures (MinOD, MaxOD and OD300) were significantly lower in SSc patients than in HC (p=0.011, p<0.0001, p<0.0001 respectively). HFUS showed a lower performance in discriminating SSc skin vs HC compared to OCT (overall AUC 0.6 vs 0.72, 0.8 and 0.89 for MinOD, MaxOD and OD300 respectively). Nevertheless, mean HFUS skin thickness significantly correlated with mRSS at site of analysis (r=0.47, p=0.0013) and showed overall excellent inter-observer reliability between assessors (ICC >0.8). Importantly, MaxOD and OD300 negatively correlated with HFUS skin thickness (r=-0.32, p=0.035; r=-0.31, p=0.039).

**Conclusions:** OCT of the skin has been previously validated against skin biopsy in SSc. Our results validate HFUS against OCT and indicate that HFUS of the skin is a reliable measure of skin involvement. Further, here we show that HFUS and OCT outperform each other in measuring different aspects of skin involvement in SSc and they offer complementary surrogate outcome measures of disease.

## P.241

## RADIOLOGICAL HAND FINDING AND ITS CORRELATION WITH INTERNAL ORGAN INVOLVEMENT IN SYSTEMIC SCLEROSIS

N. Abdolahi, F. Badiee, S. Tavassoli, M. Aghaei, M.H. Gharib, S. Livani

*Golestan Univercity of Medical Sciences, Golestan Rheumatology Research Center, Gorgan, Iran*

**Introduction:** Systemic sclerosis (SSc) is a connective tissue disease with heterogeneous symptoms and a non-predictable process. Joint involvement occurs in thirds of SSc patients during the course of the disease. It usually diagnosed by physical examination and radiography. Joint inflammation, especially in the patients with high levels of Anti-CCP Antibody and erosive arthritis may be caused by the SSc itself or it can be a sign of SSc-RA overlap syndrome. Keeping in mind these findings, this study was undertaken mainly to determine the prevalence and characteristics of radiological features of the hand in our population of SSc patients and also, to define the relationship between radiographic findings and internal organ involvement, RF and Anti-CCP Antibody.

**Material and Methods:** 43 patients, all fulfilling the American College of Rheumatology criteria for the classification of SSc, were invited by telephone. skin involvement was determined on the basis of modified Rodnan skin score (mRSS). The following biological tests; fasting blood glucose (FBS), Hemoglobin A1c (HbA1c), rheumatoid factor (RF) and anti-cyclic citrullinated peptide (Anti-CCP) antibody were carried out. Standard anteroposterior views of the hands were obtained for all patients. All radiographs were evaluated by a radiologist using a modified version of the radiographic classification of La Montagna and koutaissoff and blinded to the clinical and serological data of the patients. The clinical data were collected from patient’ records. pulmonary involvement was assessed by a new chest radiograph and pulmonary function tests (PFT) and high-resolution computed tomography of patient’ records.

**Results:** Juxta-articular osteoporosis, acro-osteolysis and joint space narrowing in hand were the most common findings observed in SSc patients. Anti-CCP Ab in 9.3% and RF in 30.2% of SSc patients were found. Joint space narrowing and marginal erosion were of high prevalence among patients with positive AntiCCP antibody. Hand joint space narrowing and acro-osteolysis were significantly associated with active skin involvement. Hand marginal erosion was significantly associated with gastrointestinal involvement.

**Conclusions:** This cross-sectional study confirms that the skeletal and articular involvement of the hand is frequent in SSc. So we suggest close monitoring of patients with the diffuse cutaneous for the occurrence or progression of this complication.

## 7. Vascular

## P.242

## LATE ONSET (OVER 65 YEARS) RAYNAUD’S PHENOMENON MAY IDENTIFY A PECULIAR SYSTEMIC SCLEROSIS’ SUBSET?

L. Urso, F. Cacciapaglia, F. Montini, V. Venerito, S. Duma, F. Iannone

*Rheumatology Unit, Department of Emergency and Organs Transplantation, University of Bari, Bari, Italy*

**Introduction:** Raynaud’s Phenomenon (RP) is the most common and precocious sign of Systemic Sclerosis (SSc). No previous studies have explored whether SSc has different outcomes according to the age of onset of RP. The aim of this single-center study was to compare the impact of early onset RP (eo-RP) and late onset RP (lo-RP) on clinical phenotype of SSc

**Material and Methods:** We retrospectively analyzed all SSc patients fulfilling the 1980 ARA and/or the 2013 ACR/EULAR classification criteria for SSc, followed in our Scleroderma Unit since January 2008. Clinical, instrumental and laboratory findings were recorded. Patients were divided into two groups according to age at the onset of RP (<65 years eo-RP, >=65 years lo-RP). Chi-square test was used to identify differences and Pearson test to assess any correlation between the groups for categorical variables, whereas ANOVA and the t-test were used to analyze quantitative variables. An alpha <0.05 was considered statistically significant

**Results:** We included 339 patients (92.0% female), with median age (IQR) at the diagnosis of 52.5 (39-64) years and a median (IQR) follow-up time of 41 (13-69) months. RP was the initial disease manifestation in 95.8% of patients and median time (IQR) to definite diagnosis from first symptom was 2 years (0-9). The eo-RP group consisted of 269 patients (79.35%), whereas in the lo-RP group were included 70 patients (20.65%). The lag time to diagnosis from onset of RP was significantly shorter in the lo-RP (yrs 2.6 ± 3.0) than in eo-RP group (yrs 6.5 ± 8.9, p<0.001). Anti-CENP Abs (46% in the lo-RP group vs 38.2% in the eo-RP group, p = 0.25) and anti-TopoI Abs (42% vs 53.5%, p = 0.14) were similar in the two groups, as well as the prevalence of diffuse cutaneous subset (7.3% vs 15.7%, p=0.07). Major organ involvement, as interstitial lung disease (49.3% vs 46.5%, p=0.68) and severe gastrointestinal involvement (10.1% vs 16.8%, p=0.17) was similar into the two groups. Pulmonary arterial hypertension was slight higher in lo-RP, although not significantly different (13% vs 7.8%, p=0.17). A higher proportion of chronic kidney disease seems to be present in the lo-RP group (10.6% vs 6%, p=0.004), but adjusting for age at symptom onset it was no longer significant

**Conclusions:** SSc with RP onset over 65 years is not a mild disease and present a frequency of major organ involvement comparable with younger patients. The short lag from RP to disease onset suggest a fast microvascular remodeling

## P.243

## THE CORRELATION BETWEEN CARDIOPULMONARY PARAMETERS AND ARTERIAL STIFFNESS IN A CROSS-SECTIONAL STUDY IN SYSTEMIC SCLEROSIS

G. Szucs, M. Kalaszi, L. Bodoki, Z. Szekanecz, S. Szamosi

*University of Debrecen, Department of Internal Medicine, Division of Rheumatology, Debrecen, Hungary*

**Introduction:** Systemic sclerosis (SSc) is a chronic autoimmune disease characterized by dysfunction of the immune system, generalized fibrosis and obliterative vasculopathy. Cardiopulmonary manifestations are the major causes of disease-related mortality in SSc. Some previous studies have shown that restrictive spirometry and reduced forced vital capacity (FVC) were associated with higher arterial stiffness, as a marker of cardiovascular risk in patients with respiratory diseases. Based on these results, we started a cross-sectional study to find correlation between the actual arterial stiffness parameters, pulmonary functions, and cardiac parameters in our patients with SSc.

**Material and Methods:** Forty-five SSc patients were recruited (35 limited, 10 diffuse cutaneous SSc) followed by our department. Flow-mediated vasodilatation (FMD), intima-media thickness (IMT), pulse wave velocity (PWV), forced vital capacity (FVC), CO diffusion (DLCO), cardiac ejection fraction (EF), diastolic dysfunction, right ventricle pressure and renal function were evaluated. The examinations were perfomed in the last one year.

**Results:** In our study the PWV was increased (8,392±2, 336m/s vs. 6,885±1,447m/s, p=0,011), the FMD was decreased (6,125±4,287% vs. 9,065±2,580%, p=0,046) in patients with lcSSc compared to patients with dcSSc. PWV and IMT were significantly increased in patients with diastolic dysfunction compared to patients with normal diastolic function. IMT was increased in wall motion disturbance (p=0,009), arrhythmias (p=0,003) and ischaemic heart disease (p=<0,001). EF correlated with FVC% (R=0,437, p=0,005) and FEV1% (R=0,489, p=0,002). We found correlation between FMD and creatinine level (R= -0,333, p= 0,047), and IMT and GFR (R=-0,434, p=0,007). The arterial stiffness did not show correlation with the definitive pulmonary parameters.

**Conclusions:** In our study the arterial stiffness was not proved as the marker of the restrictive pulmonary function abnormalities, but we found significant correlation between the arterial stiffness and more cardiac parameters.

## P.244

## INFLUENCE OF EPOPROSTENOL INFUSIONS ON SERUM LEVEL OF ADIPOKINES IN SYSTEMIC SCLEROSIS

A. Stochmal, J. Czuwara, M. Zaremba, L. Rudnicka

*Department of Dermatology, Medical University of Warsaw, Warsaw, Poland*

**Introduction:** Adiponectin, resistin and leptin belong to adipokines, a group of molecules secreted mostly by adipose tissue which have been reported recently as a link between prolonged inflammation, progressive fibrosis and vascular abnormalities in systemic sclerosis (SSc). Adiponectin which concentration in SSc patients sera is lower, posesses anti-fibrotic, anti-inflammatory and protective features against endothelial injury. Both leptin and resistin exhibit contradictory to adiponectin properties triggering inflammation and activation of skin fibroblasts.

The aim of the study was to evaluate whether the level of the above mentioned adipokines is modulated by intravenous infusions of epoprostenol, a prostaglandin I2 analogue or intravenous treatment with sulodexide.

**Material and Methods:** Serum concentrations of adiponectin, resistin and leptin were assessed before and after 3 consecutive days of intravenous infusions of either epoprostenol 25 mcg per day in 27 SSc-patients or sulodexide 1200 LSU per day in 5 SSc-patients. Concentrations were measured with ELISA (R&D Systems, Minneapolis, MN, USA).

**Results:** Serum concentration of adiponectin after treatment with epoprostenol was significantly higher than the basal level (mean vs. 9292 ± 6059 ng/ml vs. 6003 ± 2814 ng/ml; P=0.002). Level of adiponectin after sulodexide infusions and concentration of resistin and leptin after administration of epoprostenol or sulodexide did not show significant alterations when compared to the baseline.

**Conclusions:** Adiponectin deficiency may be an important factor of vascular dysfunction in systemic sclerosis. Epoprostenol up-regulates serum concentration of adiponectin which suggests that prostacycline analogue mediates not only vasodilation but may influence fat tissue metabolic pathway preventing endothelial cells damage. Our preliminary data needs further confirmation but present an interesting role of the rheological treatment usefulness in systemic sclerosis.

## P.245

## SMOKING, RAYNAUD’S PHENOMENON, DIGITAL ULCERS AND SKIN THICKNESS IN THE WAIKATO SYSTEMIC SCLEROSIS COHORT

K.K. Solanki, C. Silva, A. Schollum, D. White

*Waikato Hospital, Hamilton, New Zealand*

**Introduction:** Systemic Sclerosis (SSc) is characterized by immune system activation and vascular disease, including Raynaud’s phenomenon and multiorgan fibrosis.

Few studies have shown the impact of smoking on general health issues and the benefits of smoking cessation. Several studies have also looked at the effect between smoking and vascular outcomes in SSc with conflicting results.

We aimed to determine the relationship between smoking, Raynaud’s phenomenon, ulcers and skin thickness in the Waikato Systemic Sclerosis cohort.

**Material and Methods:** Waikato SSc database was reviewed. Variables collected included demographics, age of diagnosis, SSc subtypes, age at first non-Raynaud’s phenomenon, medications used for treatment of Raynaud’s phenomenon or ulcers and maximal modified Rodnan skin score (mRSS). Raynaud’s phenomenon and digital ulcers (severity for each over the past week and since diagnosis) and a Scleroderma Health Assessment Questionnaire (SHAQ) visual analogue 10 cm scale was collected. The lead Rheumatologist completed a Physician’s assessment of Raynaud’s / disease severity questionnaire.

**Results:** Of the cohort of 143 patients, 100 patients were eligible to complete the questionnaires. 75 patients returned completed questionnaires. Of these, the majority were female (88%), 52 (69.3%) had lcSSc, 17 (22.7%) had dcSSc and 6 (8%) had an overlap syndrome.

36 (48%) had a smoking history and 4 (5.3%) were current smokers. Mean ± SD pack years smoked was 17.11 ± 15.29 years.

35 participants had history of ulcers, with a median of 4 (range 1-20). Of 17 patients with dcSSc 12 (70.6%) had ulcers in comparison to 17 of 52 (32.7%) patients with lcSSc. There was a significant relationship between SSc subtype and the number with ulcers (X2=10.1, p=0.007). There was also a significant relationship between Physician severity of Raynaud’s and presence of ulcers (t=6.1, p < 0.001), which was not evident between Patients severity of Raynauds and presence of ulcers (t=1.9, p=0.06).

On the SHAQ score smokers had significantly worse Raynauds phenomenon over the prior week (t=3.08, p=0.03) and were more likely to note ulcers over the preceding week, although the latter was not statistically significant (t=1.95, p=0.055). There was no association between smoking and skin thickness as measured by mRSS (r=0.23, p=0.19).

**Conclusions:** This study demonstrates that smokers had worse Raynaud’s phenomenon over the past week and were also more likely to note digital ulcers. It has also shown that patients with dcSSc had more ulcers in comparison to lcSSc. This study justifies Physicians strongly recommending smoking cessation in patients with SSc.

## P.246

## COPEPTIN AS A BIOMARKER OF PERIPHERAL MICROCIRCULATION DISORDERS IN SYSTEMIC SCLEROSIS

M. Sikora, M. Chrabaszcz, M. Olszewska, L. Rudnicka

*Medical University of Warsaw - Department of Dermatology, Warsaw, Poland*

**Introduction:** Vascular damage is a predominant manifestation of systemic sclerosis. A critical ischemia along with digital ulcers is the most severe form of microcirculation disorder leading to pain, disability and impairment in quality of life. Biomarkers for rapid progression of vasculopathy are still being under study. Vasopressin is a potent vasoconstrictive agent involved in the pathophysiology of Raynaud’s phenomenon. However, a short half-life of vasopressin limits its usage as a potential biomarker. The study aimed to determine the concentration of copeptin (a peptide reflecting the activity of the vasopressinergic system) in patients with systemic sclerosis.

**Material and Methods:** The study involved 40 patients with systemic sclerosis and 40 age and sex-matched controls. The serum concentration of copeptin was determined by enzyme immunoassay (ELISA).

**Results:** Patients with systemic sclerosis had a significantly higher plasma concentration of copeptin compared to the control group (173.4 ± 32.7 vs 81.6 ± 14.3 pg/ml, p <0.05). A positive correlation was found between the severity of Raynaud’s phenomenon and copeptin concentration (r = 0.71, p<0.05). Patients with an ‘active’ and ‘late’ scleroderma nailfold capillaroscopy patterns presented higher copeptin values than those with an ‘early’ pattern. Intravenous prostaglandin analogue treatment resulted in the decrease of copeptin concentration (185.5 pg/ml before treatment vs 136.7 pg/ml after treatment).

**Conclusions:** Copeptin appears to be a promising biomarker of vascular damage and therapeutic response to vasoactive medications. Further research on copeptin may contribute to the identification of patients with rapidly progressing vascular lesions and a high risk of digital ulcers.

## P.247

## SERUM LEVELS OF COL1A1 GENOMIC DNA AS A POTENTIAL DISEASE MARKER FOR PATIENTS WITH SYSTEMIC SCLEROSIS

S. Sawamura, K. Makino, T. Makino, I. Kajihara, S. Fukushima, M. Jinnin, H. Ihn

*Kumamoto University - Department of Dermatology and Plastic Surgery, Kumamoto, Japan*

**Introduction:** Recently, many studies have indicated that various nucleic acids are present in human sera, and attracted attention for the potential as novel disease markers in many human diseases. In this study, we tried to evaluate the possibility that DNA and RNA of collagens exist in the human serum, and determined whether their serum levels can be useful biomarkers in patients with systemic sclerosis (SSc).

**Material and Methods:** Serum samples were obtained from 22 SSc patients and 25 control subjects (10 normal subjects, seven systemic lupus erythematosus patients and eight morphea patients). The RNA or DNA of type I collagen were purified from sera, and detected by polymerase chain reaction or quantitated by real-time polymerase chain reaction.

**Results:** Among approximately 18,360 bases of full-length COL1A1 genomic DNA, various regions were detected by polymerase chain reaction in human sera. On the other hand, COL1A2 genomic DNA or COL1A1/COL1A2 RNA were not detectable. The levels of serum COL1A1 genomic DNA were significantly increased in SSc patients compared to control subjects. According to the receiver–operator curve analysis, serum COL1A1 genomic DNA levels were shown to be effective as a diagnostic marker of SSc. Furthermore, when we determined the association of serum COL1A1 genomic DNA levels with clinical/laboratory features in SSc patients, those with elevated COL1A1 genomic DNA levels showed significantly higher prevalence of pitting scars/ulcers.

**Conclusions:** In conclusion, elevation of serum COL1A1 genomic DNA levels in SSc patients may be useful as the diagnostic marker, reflecting the presence of vasculopathy.

## P.248

## SUBCLINICAL ATHEROMATOSIS IN PATIENTS WITH SYSTEMIC SCLEROSIS UNDER 60 YEARS OLD

I. Sanz Pérez^1^, A. Guillén-del-Castillo^1^, F. Martinez-Valle^1^, O. Orozco Gálvez^1^, C. Marcos Fosch^1^, E. Callejas-Moraga^2^, M. Riveiro Barciela^1^, M. Roca Herrera^1^, P. Gubern Prieto^1^, V. Fonollosa-Pla^1^, C.P. Simeón-Aznar^1^

^1^*Hospital Universitari Vall d´Hebron, Barcelona, Spain*, ^2^*Hospital Parc Taulí, Sabadell, Barcelona, Spain*

**Introduction:** The aim of this study is to compare subclinical atheromatosis by carotid ultrasound in a cohort of patients with Systemic Sclerosis (SSc) under 60 years of old compared to healthy controls.

**Material and Methods:** Sixty-three patients with SSc according to the ACR / EULAR 2013 criteria from Vall d´Hebron Hospital cohort were compared to 63 healthy controls without chronic pathologies, although they could present classic risk factors for cardiovascular disease. Both groups were evaluated with clinical interview, blood analysis and carotid ultrasound for plaque detection, according to the Manheim consensus (1). The project was approved by the Ethics Committee of the Hospital, obtaining the informed consent of the participants.

**Results:** Fifty-two patients with SSc were women (82.5%) with an average of 16 years of evolution of the disease (range 1-45). Thirty-seven (61%) were SSc limited subtype (lSSc), 13 (20.3%) diffuse subtype (dSSc), 7 (10.2%) sine scleroderma subtype (ssSSc) and 5 (6.8%) early subtype. Seven (15.3%) had pulmonary hypertension (PH), 26 (41.3%) had digital ulcers and 27 (42.9%) had interstitial lung disease. They were compared to 63 healthy controls, 36 women (57.1%). There were no statistically significant differences in mean age 48.5 vs 47.4 years old p: 0.47, arterial hypertension 18 (28.6%) vs 9 (14.3%) p: 0.09. diabetes mellitus 1 (1.6%) vs 0 (0%) p: 0.33.

SSc patients received more statin treatment 15 (23.8%) vs. 3 (4.8%) p <0.01 (CI 0.074-0.31) and were less smokers 17 (27%) vs.34 (54%) p <0.01 (IC 3.3-7.9)

Patients with SSc had lower total cholesterol values 196.9 vs. 217.6 mg/dl; p <0.05 (CI 3.02-38.3) and lower LDL cholesterol 118.9 vs. 137.74 mg /dl; p <0.05 (CI 4.34-33.18).

There were no significant differences in HDL cholesterol 55.7 vs. 58.2 mg/dl, triglycerides 114.8 vs. 105.1 mg / dl, Apo A 160.52 vs 162.62 mg / dl, Apo B 94.67 vs. 104.73 mg / dl or PCR 0.36 vs. 0.37 mg / dl.

Nineteen patients with SSc had plaque on carotid ultrasound (30.2%) vs. 8 (12.7%) healthy controls (p <0.01 CI: 0.03-0.31). Multiple regression showed that PH and decreased HDL values are independent factors associated with the presence of subclinical atheromatosis.

**Conclusions:** SSc patients less than 60 years old had more subclinical atheromatosis than healthy matched controls, despite SSc were less smokers and had better levels of total cholesterol and LDL. The presence of PH and decreased HDL cholesterol are independent factors associated with the presence of subclinical atheromatosis in SSc patients.

## P.249

## NAILFOLD CAPILLAROSCOPY FINDINGS IN MIXED CONNECTIVE TISSUE DISEASE

B. Samões, D. Fonseca, R. Vieira, T. Videira, J. Abelha-Aleixo, P. Pinto, M. Guerra

*Centro Hospitalar de Vila Nova de Gaia e Espinho, Vila Nova de Gaia, Portugal*

**Introduction:** Mixed connective tissue disease (MCTD) is a disorder that combines clinical aspects of several systemic diseases such as systemic lupus erythematosus, systemic sclerosis (SS) and polymyositis, while testing positive for anti-U1 RNP antibodies. Nailfold capillaroscopy (NFC) is a non-invasive tool to evaluate microvascular damage, having an established role in SS diagnosis and probable prognostic value. Few authors have described capillaroscopic findings in MCTD, with conflicting results. We aimed to cross-sectionally evaluate the capillaroscopic characteristics in a set of patients with MCTD.

**Material and Methods:** Patients followed at our Rheumatology outpatient department with the diagnosis of MCTD were consecutively included for NFC analysis. NFC was performed with a Dino-Lite capillaroscope, x200 magnification; 2nd to 5th fingers were included bilaterally, with 4 field images taken per finger. Findings were analyzed according to the definitions of the EULAR study group on microcirculation in rheumatic diseases. Clinical and demographic data were obtained through patient records.

**Results:** 10 patients were included, with a mean age of 45±13 years-old and 9 (90%) were female. The mean disease duration was 6.3±5.98 years. Most of the patients had Raynaud phenomenon (n=9, 90%) and arthritis (n=9, 90%). Mucocutaneous symptoms (photossensibility, malar rash, alopecia and oral ulcers) were the second most common disease manifestation (n=8, 80%). Other manifestations were sclerodactyly (n=3, 30%), esophageal dysmotility (n=2, 20%), leukopenia (n=2, 20%) and interstitial lung disease (n=1, 10%) and 1 had had myositis (10%). All patients were positive for anti-U1 RNP antibodies (mean titer = 133.4±48.72 U/mL). When the NFC was performed, 2 (20%) patients were under nifedipine, 3 (30%) were under immunosuppressive therapy [mycophenolate mofetil (n=2, 20%) and azathioprine (n=1, 10%)] and 9 (90%) were under hydroxychloroquine. Qualitatively, only 2 patients had a scleroderma pattern (1 early and other active). The remainder, 7 (70%) had non-specific abnormalities and 1 patient had a normal pattern. Quantitatively, the capillary density was globally normal [median number of capillaries/mm = 8 (3,15)]; the majority of the patients had hemorrhages [n=9 (90%)] and abnormal morphology [n=9 (90%)] and 2 (20%) patients had giant capillaries. Overall, the median number of hemorrhages, giant capillaries and abnormal morphology per mm was 0 ([0,7], [0,2] and [0,7], respectively).

**Conclusions:** In this MCTD cohort, scleroderma pattern was infrequent, in contrast with other studies where it was found in more than half of the patients. Larger multicentric studies are needed to further evaluate if NFC has a potential role in the diagnostic and prognostic approach of MCTD.

## P.250

## A CANDIDATE GENE STUDY REVEALS ASSOCIATION BETWEEN A VARIANT OF THE SRP55 SPLICING FACTOR GENE AND SYSTEMIC SCLEROSIS (SSC)

E. Romano^1^, M. Manetti^1^, J. Kosalka^2^, B.S. Fioretto^1^, I. Rosa^1^, E. Sticchi^1^, S. Guiducci^1^, S. Bellando-Randone^1^, L. Ibba-Manneschi^1^, M. Matucci-Cerinic^1^

^1^*University of Firenze - Department of Experimental and Clinical Medicine, Firenze, Italy*, ^2^*Jagiellonian University Medical College - Department of Medicine, Cracow, Poland*

**Introduction:** In SSc, alternative splicing of the last exon (exon 8) of vascular endothelial growth factor (VEGF)-A pre-mRNA is a key element in the switch from proangiogenic to antiangiogenic VEGF-A isoforms. The mRNA-binding protein serine/arginine protein 55 (SRp55, also known as SFRS6) is a key regulatory splicing factor that promotes distal splice-site selection in the exon 8 region of VEGF-A pre-mRNA and subsequent upregulation of the exon 8b-containing VEGF165b antiangiogenic isoform. Overexpression of either VEGF165b or SRp55 has been implicated in SSc-related angiogenesis impairment and peripheral vascular damage. Of note, previous studies reported the lack of sequence variations in the VEGF-A alternatively spliced region, while a single nucleotide polymorphism (SNP) in the SRp55 gene (rs2235611) has been associated with the control of VEGF-A isoforms and susceptibility to angiogenic eye disease. This case-control pilot study examined the possible implication of SRp55 rs2235611 SNP in the genetic predisposition to SSc susceptibility and clinical phenotype.

**Material and Methods:** A total population of 872 white Italian individuals (414 SSc patients, 458 controls) was studied. SSc patients were clinically classified (limited and diffuse cutaneous subsets) and the presence of autoantibodies (anticentromere, anti-topoisomerase I antibodies), pulmonary fibrosis and digital ulcers (DUs) was evaluated. The SRp55 rs2235611 SNP was genotyped by TaqMan Real-Time PCR.

**Results:** SRp55 rs2235611 genotype distribution and allele frequency were similar in SSc and healthy controls, though a trend toward significance was observed for genotype distribution (p=0.07). The SRp55 rs2235611 AA genotype significantly influenced the predisposition to SSc (OR 2.55, 95% CI 1.11 to 5.57, p=0.02). No significant difference in genotype distribution and allele frequencies was observed between limited and diffuse cutaneous SSc subsets, as well as according to autoantibody status and presence of pulmonary fibrosis. The SRp55 rs2235611 A allele frequency was significantly greater in SSc patients without DUs than in patients with DUs (p=0.04).

**Conclusions:** The SRp55 rs2235611 polymorphism is associated with susceptibility to SSc, consistent with a role of VEGF-A pre-mRNA alternative splicing in disease-related impaired angiogenesis and peripheral vascular phenotype. Further replication studies are warranted to confirm our findings in independent SSc cohorts.

## P.251

## LINKING US AND EUROPE: CONSENSUS BASED STANDARD EVALUATION OF DERMATOSCOPY AND NAILFOLD VIDEOCAPILLAROSCOPY, A EULAR STUDY GROUP ON MICROCIRCULATION IN RHEUMATIC DISEASES PROJECT

M. Radic^1^, T. Frech^2^, M. Snow^3^, L.A. Saketkoo^4^, M. Cutolo^5^, V. Smith^6^

^1^*University Hospital Split, Division of Rheumatology and Clinical Immunology, Center of excellence for Systemic Sclerosis, Split, Croatia*, ^2^*University of Utah, Division of Rheumatology, Salt Lake City, USA*, ^3^*University of Nebraska Medical Center, Department of Internal Medicine, Division of Rheumatology, Omaha, USA*, ^4^*Tulane University School of Medicine, New Orleans Scleroderma & Sarcoidosis Patient Care & Research Center, UMC Comprehe, New Orleans, USA*, ^5^*IRCCS Polyclinic Hospital San Martino, Research Laboratory and Academic Division of Clinical Rheumatology, Postgraduate, Genova, Italy*, ^6^*Ghent University Hospital, Department of Rheumatology, Department of Internal Medicine, Ghent University, Ghent, Belgium*

**Introduction:** Nailfold videocapillaroscopy (NVC) is the current gold standard for detection and quantification of capillary abnormalities in Raynaud’s phenomenon (RP). The objective of this study is to introduce dermatoscopy as a screening tool in RP.

**Material and Methods:** Nailfold capillaries were examined by a hand-held non-contact polarized dermatoscope (HEINE Delta 20 T Dermatoscope, HEINE Optotechnik GmbH & Co., Herrsching, Germany) connected to the digital camera via adapter to improve the quality of images with 10-16x magnification (N=100), and a hand-held non-contact polarized dermatoscope with 10x lens (DermaLite, DL3 model, 3Gen, San Juan Capistrano, CA, USA) connected to an iPad (mini 4) via adapter (DermLite Connection Kit) (N=100). Images taken by both dermatoscopes were marked with an arrowhead. NVC examination was performed at the arrowhead by a device equipped with a 200× lens (Optilia instruments AB, Sollentuna, Sweden) (N=100). All dermatoscopy and NVC examinations were performed by a single reader (MR) blinded for clinical parameters and patient status. Consensus evaluation (MR, TF, MS, MC, VS) of dermatoscopy characteristics/grade was determined in the following categories: qualitative reduction of capillary density, enlarged capillaries (appearance of dilatation), hemorrhages and abnormal shapes. Each dermatoscopic image was then given a final impression of normal, non-specific, or scleroderma pattern. NVC was graded as per standard of EULAR study group on microcirculation in rheumatic diseases.

**Results:** Evaluation results of two hundred dermatoscopic images were: normal 8 (4.0%), non-interpretable 69 (34.5%) and non-specific 80 (40.0%). Overall scleroderma pattern was shown in 83 NVC images, while 17 did not fulfil criterial for ‘early’, ‘active’ or ‘late’ scleroderma patterns. All normal dermatoscopic images were confirmed as normal by NVC. Six (7.2%) non-interpretable and 37 (41.0%) non-specific dermatoscopic images showed a scleroderma pattern in NVC. Although scleroderma pattern was found in 43 (21.5%) dermatoscopic images, it was confirmed in 43 (51.8%) NVC images.

**Conclusions:** Dermatoscopy can be a valuable tool for screening nailfold capillaries in RP in many cases. It has limitations in when capillaries are difficult to visualize and in those with non-specific abnormalities. Non-specific changes and non-interpretable dermatoscopy findings are to be sent for further evaluation by NVC.

## P.252

## INCIDENCE OF PULMONARY ARTERIAL HYPERTENSION AND SCLERODERMA RENAL CRISIS IN SYSTEMIC SCLEROSIS PATIENTS WITH DIGITAL ULCERS ON ENDOTHELIN ANTAGONIST RECEPTORS AND PHOSPHODIESTERASE-5 INHIBITORS

M. Pestana-Fernandez^1^, M. Rubio-Rivas^1^, C. Tolosa-Vilella^2^, A. Guillén-Del-Castillo^3^, M. Freire^4^, J.A. Vargas Hitos^5^, J.A. Todolí Parra^6^, A. Marín Ballvé^7^, D. Colunga Argüelles^8^, C. González-Echávarri Pérez^9^, N. Ortego-Centeno^10^, L. Trapiella Martínez^11^, X. Pla Salas^12^, A.-J. Chamorro^13^, I. Perales Fraile^14^, M. Ruiz Muñoz^15^, E. Callejas Moraga^2^, V. Fonollosa Pla^3^, C.P. Simeón Aznar^3^, On behalf of RESCLE investigators^16^

^1^*Unit of Autoimmune Diseases, Department of Internal Medicine. Hospital Universitario de Bellvitge-IDIBELL, L’Hospitalet de Llobregat, Barcelona, Spain*, ^2^*Department of Internal Medicine. Corporación Sanitaria Universitaria Parc Taulí, Sabadell, Barcelona, Spain*, ^3^*Unit of Autoimmune Diseases, Department of Internal Medicine. Hospital Universitario Vall d’Hebron, Barcelona, Spain*, ^4^ *Unit of Autoimmune Diseases, Department of Internal Medicine. Hospital Clínico Universitario de Santiago, Santiago de Compostela, A Coruña, Spain*, ^5^*Department of Internal Medicine. Hospital Universitario Virgen de las Nieves, Granada, Spain*, ^6^*Department of Internal Medicine. Hospital Universitario y Politécnico La Fe, Valencia, Spain*, ^7^ *Unit of Autoimmune Diseases, Department of Internal Medicine. Hospital Clínico Universitario Lozano Blesa, Zaragoza, Spain*, ^8^*Department of Internal Medicine. Hospital Universitario Central de Asturias, Oviedo, Asturias, Spain*, ^9^*Autoimmune Diseases Research Unit, Department of Internal Medicine. Biocruces Bizkaia Health Research Institute, Hospital Universitario Cruces, University of the Basque Country, Barakaldo, Vizacaya, Spain*, ^10^*Inst Invest Biosanitaria Ibs Granada. Department of Internal Medicine, Unit of Systemic Autoimmune Diseases Department of MedicineFaculty of Medicine, Hospital Universitario San Cecilio, Granada, Spain*, ^11^*Unit of Systemic Autoimmune Diseases, Department of Internal Medicine. Hospital de Cabueñes, Gijón, Asturias, Spain*, ^12^ *Unit of Systemic Autoimmune Diseases, Department of Internal Medicine. Consorci Hospitalari de Vic, Vic, Barcelona, Spain*, ^13^*Department of Internal Medicine. Complejo Asistencial Universitario de Salamanca, Salamanca, Spain*, ^14^*Department of Internal Medicine. Hospital Universitario Rey Juan Carlos, Móstoles, Madrid, Spain*, ^15^*Department of Internal Medicine. Hospital Universitario Fundación Alcorcón, Alcorcón, Madrid, Spain*, ^16^*Autoinmune Diseases Study Group (GEAS), Spain*

**Introduction:** Treatment with Endothelin Antagonist Receptors (ERAs) and Phosphodiesterase-5 inhibitors (PDE5i) is beneficial in Pulmonary Arterial Hypertension (PAH) and digital ulcers (DU) and prevent recurrences of DU. Our study aimed to evaluate the incidence rate of PAH and Scleroderma Renal Crisis (SRC) in patients with Systemic sclerosis (SSc) and DU under or not ERAs/PDE5i.

**Material and Methods:** We conducted a retrospective cohort study including all patients with SSc and DU from the Spanish Scleroderma Registry (RESCLE). Patients were split up in two groups: patients under AREs and/or PDE5i vs. patients not under ERAs/PDE5i (no-treatment group). The primary outcome was the incidence rate of PAH and SRC and the secondary outcome the time from first DU to PAH and SRC diagnosis.

**Results:** 551 patients out of 1817 (30.3%) in the RESCLE database had suffered from DU, 225 (41%) under specific treatment and 326 (59%) without. The dcSSc subset was more prevalent in the treatment group (36% vs. 26%; p=0.023), along with ATA (34% vs. 18%; p<0.001) and tendon friction rubs (13% vs. 6%; p=0.023) whereas the lcSSc subset was more prevalent in the no-treatment group (55% vs. 65%; p=0.021) along with ACA (37% vs. 47%; p=0.037). We did not find differences between groups in terms of the incidence rate of PAH [7.7 (4.7-12.0) per 1,000 person-year] vs. 7.8 (5.2-11.3) per 1,000 person-year; p=0.988] or SRC [3.4 (1.6-6.5) per 1,000 person-year vs. 2.7 (1.3-4.9) per 1,000 person-year; p=0.620]. However, time from the first DU to the diagnosis of SRC was delayed in treated patients (7.6±5.8 years vs. 2.9±5.3; p=0.021).

**Conclusions:** There was no difference in PAH and SRC incidence rate. However, treatment with AREs and/or PDE5i seemed to delay the appearance of SRC.

**Figure:**
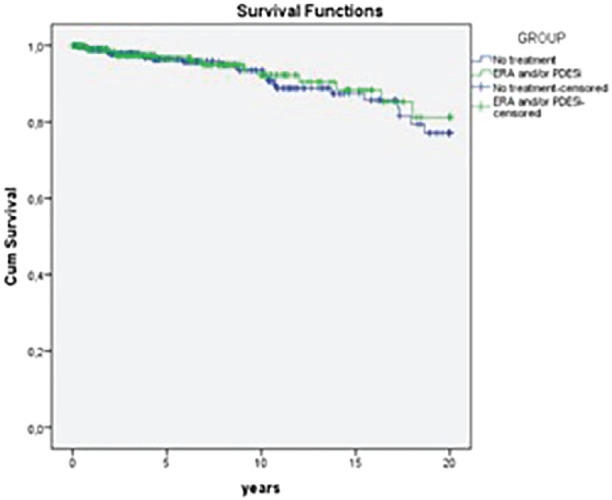


**Figure:**
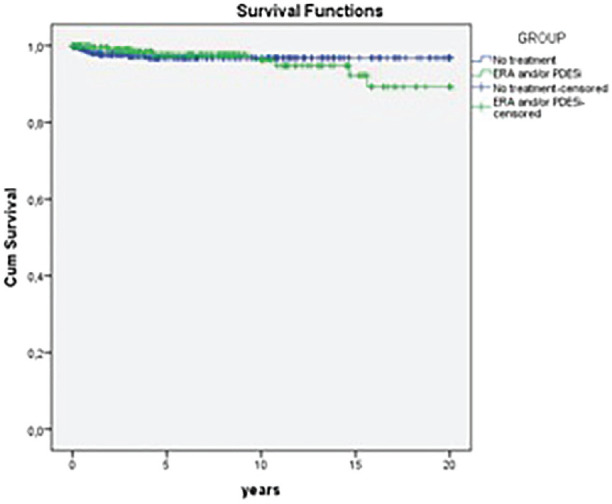


## P.253

## DECREASED CAPILLARY DENSITY AND ALTERED CAPILLARY MORPHOLOGY IS ASSOCIATED WITH INTERNAL ORGAN INVOLVEMENT IN SYSTEMIC SCLEROSIS

G. Nagy, A. Kovács, T. Minier, C. Varjú, L. Czirják, G. Kumánovics

*University of Pécs, Medical School, Department of Rheumatology and Immunology, Pécs, Hungary*

**Introduction:** Background: Nailfold videocapillaroscopy (NVC) is a simple method to evaluate capillary density and morphology. Systemic sclerosis (SSc) is characterized by fibrosis, autoimmune phenomena and vascular abnormalities. Capillary loss and malformation is a general finding in patients with SSc.

Objective: To examine correlation between capillary density (CD) and morphology and internal organ involvement in patients with SSc.

**Material and Methods:** Methods: 140 patients with SSc underwent standardized clinical-laboratory examination. CD and morphology was evaluated with videocapillaroscop. 73 patients had limited cutaneous SSc (lcSSc) and 67 had diffuse cutaneous SSc (dcSSc). 47 patients had maximum of 5 years of disease duration (early SSc) and 93 patients had disease duration>5 years (late SSc) calculated from the first non-Raynaud symptoms.

**Results:** Results: Forced vital capacity (FVC) and diffusing capacity of carbon monoxide (DLCO) showed significant correlation with CD in entire cohort (FVC: rho: 0.236, p<0.01; DLCO: rho: 0.319, p<0.001). Patients with pulmonary fibrosis by HRCT had significantly lower CD (5.7 /4.8;7.1/ vs. 7.4 /6;8.5/, p<0.0001) than patients without pulmonary fibrosis.

Ejection fraction showed significant correlation with ramification score in the entire cohort (rho: -0.185 p<0.05), and in patients with late SSc (rho:-0.249, p<0.05). Calculated right ventricular pressure (cRVP) showed significant correlation with CD in the entire cohort (rho:-0.3215, p<0.001), in dcSSc (-0.360 p<0.05) and in patients with late SSc (rho: -0.296, p<0.05).

Patients with oesophageal dysmotility or stricture had lower CD compared to cases without this abnormality (5.7 /4.8;6.8/ vs. 6.9 /5.2;8.2/, p<0.01). Patients with malabsorption had lower CD compared to ones without it (6.3 /5.3;7.9/ vs. 7.9 /6.0;8.9/, p<0.05). Correlation between CD and body mass index was observed in the entire SSc cohort (rho: 0.190, p<0.05), in dcSSc (rho: 0.351, p<0.01) and in late SSc (rho: 0,328p<0.05). UCLA GIT questionnaire showed correlations with CD in the entire cohort (rho: 0.185, p<0.05), and in lcSSc (rho:0.255, p<0.05). Albumin concentration showed correlation with CD in dcSSc (rho: 0.302, p<0.05).

**Conclusions:** Conclusion: The well-known correlation between CD and cardiopulmonary involvement, including cRVP, FVC and DLCO were confirmed. Furthermore, we also demonstrated associations with gastrointestinal involvement also. With NVC measurements we can suggest that decreased CD may play an important role in the pathogenic process of all major internal organ manifestations of SSc.

**Funding:** ‘PEPSYS GINOP-2.3.2-15-2016-00050’ ‘ÚNKP-19-3: New National Excellence Program of the Ministry of the Human Capacities’.

## P.254

## A PILOT STUDY OF PERFUSION AND OXYGENATION OF SYSTEMIC SCLEROSIS-RELATED DIGITAL (FINGER) ULCERS

J. Manning^1^, E. Marjanovic^1^, T. Moore^1^, G. Dinsdale^1^, S. Wilkinson^1^, M. Dickinson^2^, A. Herrick^1^, A. Murray^1^

^1^*Division of Musculoskeletal and Dermatological Sciences, MAHSC, Salford Royal NHS Foundation Trust, University of Manche, Manchester, United Kingdom*, ^2^*Dept of Physics and Astronomy, University of Manchester, Manchester, United Kingdom*

**Introduction:** Systemic sclerosis (SSc)-related digital ulcers (DU) have a huge effect on patients’ quality of life and hand function. They can be chronic and difficult to treat, and are difficult to measure and to define. Relatively little is known about the pathophysiology of SSc-related DU, although recently it has been shown that both fingertip and extensor surface ulcers are ischaemic. The objective of this study was to use non-invasive imaging techniques to examine (in a pilot study) SSc-related finger ulcers. The hypothesis of the study was to determine whether digital ulcers are hypoxic as well as ischaemic.

**Material and Methods:** Nine patients with SSc (8 female, 1 male, median age 62 [IQR 60 to 69] years, duration of Raynaud’s 18 [12 to 27] years, onset of first non-Raynaud’s feature 17 [12 to 22] years) underwent imaging of DU. Measurements of perfusion (laser Doppler imaging [LDI], laser speckle imaging and thermography), oxygenation (multispectral imaging), and vessel size (LDI) were performed at the site of ulceration to include skin from an adjacent (unaffected) site.

**Results:** One ulcer from each patient was imaged; 8 tip and one extensor. Perfusion as measured by LDI and laser speckle showed significant decreases at the ulcer site as compared to the adjacent site: LDI, ulcer median 215 [IQR 97 to 507] vs. adjacent 344 [222 to 1071] arbitrary perfusion units (PU), p<0.05; laser speckle, 170 [104 to 210] vs. 443 [234 to 790] PU. Thermal imaging did not identify differences in perfusion, 31.9 [28.2 to 33.2] vs.31.9 [28.3 to 33.2]oC, non-significant. Oxygenation was decreased in the ulcer vs. adjacent site but was not statistically significant, 0.083 [0.035 to 0.193] vs. 0.145 [0.002 to 0.254]. The mean lesion area was 3.4 [3.25 to 4.95] cm2. Perfusion was associated with oxygenation (r=0.83, p<0.01).

**Conclusions:** LDI and laser speckle were both sensitive to differences in perfusion between the ulcer site and the adjacent area. That thermography was not, may be due to its lower resolution and also to the fact that thermal imaging obtains its signal from deeper within the tissue as well as more superficially; deeper tissue is likely unaffected. Oxygenation measures were not significantly different between the ulcer and adjacent sites but did show a strong relationship to perfusion, indicating that the ulcers imaged were both ischaemic and hypoxic. As well as providing more insight into pathogenesis, measuring ulcer oxygenation (as well as perfusion) may provide a biomarker of ulcer healing.

## P.255

## PLASMA D-DIMER CONCENTRATION, MACROVASCULAR DISEASE AND MORTALITY IN PATIENTS WITH SYSTEMIC SCLEROSIS

L. Montolio Chiva^1^, J.J. Alegre Sancho^1^, M. Robustillo Villarino^2^, M. Aguilar Zamora^1^, E. Valls Pascual^1^, D. Ybañez Garcia^1^, A. Martinez Ferrer^1^, A.V. Orenes Vera^1^, I. Vázquez Gómez^1^, I. Torner Hernández^1^, V. Nuñez Monje^1^, A. Sendra Garcia^1^

^1^*Rheumatology Department, Dr Peset University Hospital, Valencia, Spain*, ^2^*Rheumatology Department, La Plana Hospital-Villarreal, Castellón De La Plana, Spain*

**Introduction:** Plasma D-dimer (DD) has proven to be a reliable marker of a systemic prothrombotic state and its measurement might be helpful in predicting cardiovascular events and even mortality. An association between high levels of DD and macrovascular disease, diffuse cutaneous involvement and active disease has been suggested in systemic sclerosis (SSc). The objective of this study is to determine the DD measurement as a marker of macrovascular disease and mortality in patients with SSc and to explore its relation with other features and biomarkers of SSc.

**Material and Methods:** Descriptive ambispective observational study. We included, from 2010 to 2015, SSc patients controlled in a tertiary hospital. We gathered demographic, clinical, and analytical variables, including DD levels measured by turbidimetric immunoassay (ACL TOP 700 CTS®, Werfen Spain). Other variables were collected retrospectively from medical record. We explored the extracranial branches of the carotid artery (ESAOTE MyLab XV70, 7-12 MHz linear probe, software RFQIMT) measuring intima media thickness (IMT) by radiofrequency, and the presence of atheroma plaques, as per the Mannheim consensus, was registered. The ankle-brachial index (ABI) was measured by a Vascular Surgeon. We considered an IMT>900µ and/or presence of atheroma plaque and/or an ABI<0.9 as macrovascular damage. We prospectively collected mortality until dec-2017.

**Results:** 115 patients where included consecutively, of which finally 100 were studied (91 women), with a mean age of 60.2 years (SD 15). Mean SSc evolution time was 13.9 years (SD 11.2). LSSc was most frequently diagnosed (50%), followed by DSSc (18%), SSc without scleroderma (17%), overlap syndrome (9%) and pre-SSc (6%). 37% of patients were hypertensive, 45% dyslipidemic, and 7% were diabetic. Overall, 40% had macrovascular damage. The mean values of DD were 437.6 ng/mL (SD 683.5), 60% of the patients having levels of DD (>250 ng/mL). During follow up, there were 16 deaths, 50% due to vascular events. Baseline high levels of plasma DD were associated to macrovascular damage and ischemic digital ulcers, together with advanced age, arthritis, inflammation biomarkers, HTA, sPAP, lower DLCO% and coexistence of an inflammatory disease (overlap, infection, neoplasia), and showed a tendency to an association with mortality. This association became statistically significant when considering DD plasma levels as a quantitative variable (p<0.001) and remained significant after adjustments (age, coexistence of an inflammatory disease) (p 0,003).

**Conclusions:** DD plasma levels are associated with macrovascular involvement and can be helpful in predicting medium-term mortality in our SSc patients. Levels of DD can be modified by systemic inflammation.

## P.256

## LOWER-LIMB ULCERS IN SYSTEMIC SCLEROSIS: A MONOCENTRIC RETROSPECTIVE STUDY OF 16 PATIENTS

E. Montabone^1^, A. Penatti^2^, V. Data^1^, G. Gardini^1^, P. De Nigris^1^, R. Carignola^1^

^1^*Scleroderma Unit, AOU San Luigi Gonzaga, Orbassano, Torino, Italy*, ^2^*Servizio di Reumatologia, ASDAA, Azienda Ospedaliera dell’Alto Adige, Merano, Italy*

**Introduction:** Ulcers are extremely painful, hard to heal manifestations of systemic sclerosis (SSc). Lower-limb ulcers in SSc patients are rarely reported, and could be related to venous insufficiency, ischemic and other causes, such as dystrophic calcinosis or multifactorial pathogenesis. Moreover, lower-limb ulcer outcome is poor, with a consistent amputation rate risk.

**Material and Methods:** We performed a retrospective study to describe the epidemiology, clinical features and outcomes of lower-limb ulcers in SSc patients referred to the Scleroderma Unit of AOU San Luigi Gonzaga, Orbassano (Italy).

**Results:** 189 patients were included; SSc patients with lower-limb ulcers were 16 (estimated prevalence 8%) and including 14 females and 2 males with a median age of 56 years at first ulcer diagnosis. 13 patients were classified as limited cutaneous SSc, 3 patients as diffuse cutaneous SSc, no patients as sine scleroderma. The ulcers were more common in leg (44%, all associated with extensive calcinosis), ankle (38%) and toes (18%). The predominant etiology of the ulcers was multifactorial (56%), vasculopathy (25%), venous disease (12%) and iatrogenic (7%). Ulcer healing was achieved in 53% of cases. Superinfection was associated with a risk of no healing. 2 patients were amputated, mainly for ischemic or infective lesions. Treatment modalities included aggressive wound bed preparation, advanced medications, intensification of pharmacological therapy (immunosuppressive and vasodilatation), surgical interventions, autologous platelet-rich fibrin, photodynamic and vacuum-assisted closure therapy.

Among SSc patients with lower-limb ulcers, 5 were affected by PAH, with an estimated prevalence of 33%; this evidence is two times higher than PAH prevalence in SSc population. One patient survived 6 years in triple therapy for PAH; two patients survived 8 and 9 years, respectively, without therapy for all drug intolerance (except iloprost i.v.) and one died for sepsis and a second for cancer; no patient died for PAH.

**Conclusions:** Management of lower-limb ulcers in SSc patients is challenging and these patients need to be referred to expert centers with experience in their management. Moreover, these results indicate lower limb-ulcers are a potential predictor of PAH risk and poor prognosis in SSc: the report of two patients who survived for more than 8 years without therapy in absence of progression, provides interesting insights into what might characterize these patients and the pathogenesis of SSc-PAH.

## P.257

## SIGNIFICANE OF ANGIOTENSIN II/ACE2/ANGIOTENSIN-(1-7) AXIS FOR THE PATHOGENESIS OF SYSTEMIC SCLEROSIS

B. Miziolek^1^, B. Bergler-Czop^1^, E. Kucharz^2^, P. Kotyla^2^, M. Kopec-Medrek^2^, M. Widuchowska^2^, M. Sienczyk^3^, A. Lupicka-Slowik^3^, R. Grzywa^3^, L. Brzezinska-Wcislo^1^

^1^*Department of Dermatology, Medical University of Silesia, Katowice, Poland*, ^2^*Department of Internal Medicine Rheumatology and Clinical Immunology, Medical University of Silesia, Katowice, Poland*, ^3^*Faculty of Chemistry, Division of Medicinal Chemistry and Microbiology, University of Science and Technology, Wroclaw, Poland*

**Introduction:** Systemic sclerosis (SSc) is a multisystemic disease with an extensive microvasculopathy, but its pathogenesis has been still unclear. Previously, disturbances in plasma levels of angiotensin II and its antagonistic angiotensin-(1-7) were found in SSc patients. Their significance for a pathogenesis of the disease stays however unclear due to discrepancies of the only two available earlier studies on measurements of angiotensins in SSc. A degradation of unfavorable Ang II into more beneficial angiotensin-(1-7) through by angiotensin-converting enzyme 2 (ACE2), was proposed in 2010 to be affected by an existence of inhibitory autoantibodies (anti-ACE2) found in some SSc patients. A prevalence of those autoantibodies was not further explored in SSc settings. The aim of this study was to evaluate a significance of disturbances in the production pathway of angiotensins for the development of SSc, and to investigate the prevalence of anti-ACE2.

**Material and Methods:** There were enrolled 27 patients SSc, 23 subjects at pre-scleroderma stage and 23 healthy controls. All participants were submitted to evaluation of internal organ involvement and blood sampling to measure plasma levels of angiotensin I, angiotensin II and angiotensin-(1-7) with ELISA technique. Anti-ACE2 were assayed in serum with (not commercially available) ELISA technique and Western-Blot.

**Results:** Plasma level of angiotensin-(1-7) was significantly reduced in both SSc (median=47,2 pg/ml; p<0,001) and pre-scleroderma patients (median=102,7 pg/ml) when compared to healthy controls (median=176,1 pg/ml). A tendency to higher than in control group (median=214 pg/ml) plasma level of angiotensin I was seen in SSc group (median=391 pg/ml; p=0,059). Differences in plasma level of angiotensin II were insignificant between all study groups. Those disturbances produced unfavorable angiotensin-(1-7)/angiotensin II [%] ratio in both groups of patients, which achieved statistical significance in SSc subjects. Anti-ACE2 were detected in only five of SSc patients and two healthy volunteers. Their presence in SSc group was accompanied by extremely low plasma level of angiotensin-(1-7) with undetectable plasma level of this angiotensin in three of those anti-ACE2 positive SSc patients.

**Conclusions:** Production of angiotensin-(1-7) was significantly reduced in both SSc patients and those at pre-scleroderma stage, although a significant imbalance between angiotensin II and angiotensin-(1-7) occurred only in SSc subjects. Anti-ACE2 antibodies seem to be nonspecific for SSc, but their occurrence may lead to severe depletion of angiotensin-(1-7).

## P.258

## MICRORNA EXPRESSION PROFILE IN MICROVASCULAR ENDOTHELIAL CELLS FROM THE SKIN OF SYSTEMIC SCLEROSIS

T. Miyamura, K. Makino, M. Ide, T. Makino, H. Ihn

*Kumamoto university Department of Dermatology and Plastic Surgery, Faculty of Life Sciences, Kumamoto, Japan*

**Introduction:** Systemic sclerosis (SSc) is an autoimmune connective tissue disorder characterized by immunological abnormalities, vasculopathy, and fibrosis. Vasculopathy is responsible for severe clinical manifestations of the disease such as pulmonary hypertension, renal crisis, digital ulcers. Though the pathogenesis of SSc is yet to be elucidated, endothelial cell (EC) injury is proposed as an important event leading to vasculopathy.

microRNAs have attracted attention for its involvement in the pathogenesis of various human diseases such as immunological disorders. microRNAs are non-protein-coding small RNAs that bind to complementary sequences in the 3`-untranslated regions (UTRs) of target mRNAs, resulting in inhibiting their translation into protein.

To elucidate the pathogenesis of SSc vasculopathy, we performed microRNA array analysis in microvascular endothelial cells from the skin of patients with SSc.

**Material and Methods:** Microvascular endothelial cells were obtained from skin from three healthy adults and three early diffuse cutaneous SSc patients. Total RNAs were extracted from ECs, then we performed integrated analysis of miRNA and mRNA (3D-Gene® TORAY INDUSTRIES, Japan).

**Results:** microRNA array analysis revealed that vasculopathy related microRNAs, such as miR-155-5p, were up-regulated in ECs from SSc.

**Conclusions:** Aberrant microRNA expressions in ECs might be involved in the pathogenesis of SSc.

## P.259

## MICROPARTICLES IN SYSTEMIC SCLEROSIS: A PROMISING BIOMARKER OF MICROVASCULAR DAMAGE?

S. Maximiano^1^, I.L. Azevedo Teixeira^2^, C. Nunes França^3^, L. Garcia Biagi^4^, M.C. De Oliveira Izar^5^, C. Kayser^6^

^1^*Federal University of São Paulo - Department of Rheumatology, São Paulo, Brazil*, ^2^*Federal University of São Paulo - Department of Cardiology, Molecular Biology Laboratory, São Paulo, Brazil*, ^3^*2, São Paulo, Brazil*, ^4^*Federal University of São Paulo - School of Medicine, São Paulo, Brazil*, ^5^*Federal University of São Paulo - Department of Cardiology, São Paulo, Brazil*, ^6^*1, São Paulo, Brazil*

**Introduction:** Systemic sclerosis (SSc) is a rare autoimmune disease characterized by vascular damage, immune dysregulation and progressive tissue fibrosis. Microparticles (MP) are membrane-derived vesicles released from cells undergoing activation or apoptosis. Depending on cellular origin, MP can act regulating inflammatory and immune responses, homeostasis and vascular function. In addition, MP work mediating intercellular communication. Prior studies have reported elevated plasma levels of MP in SSc, but the association of different cellular MP with distinct clinical manifestations was not completely studied. The present study aimed to analyze the serum levels of MP derived from platelets (PMP), endothelial cells (EMP) and monocytes (MMP) in scleroderma patients compared to healthy controls, and to evaluate the association of MP levels and SSc clinical features.

**Material and Methods:** In this cross-sectional study, 34 SSc patients and 15 age- and sex-matched healthy controls were recruited. Plasma MP were quantified by flow cytometry after staining with fluorescent cell-specific monoclonal antibodies (CD42+/31+ for PMP, CD105+ for EMP, and CD14+ for MMP). Clinical and laboratory evaluation of patients were obtained. Nailfold capillaroscopy was also performed in all patients. All of them fulfilled the 2013 ACR/EULAR classification criteria for SSc and were under stable pharmacological therapy for at least three months.

**Results:** Demographic characteristics of the population studied are summarized on table 1. Serum levels of all MP were significantly increased in SSc patients compared with healthy controls (mean ±SD: 80.02% ±15.49% versus 67.33% ±22.86% for PMP, p = 0.036; 48.55% ±8.59% versus 27.19% ±14.51% for EMP, p < 0.0001; and 5.12% ±2.93% versus 1.29% ±2.05% for MMP, p < 0.0001). Among SSc patients, MMP levels were significantly lower in patients with the late pattern compared to those with the early or active pattern on capillaroscopy (p = 0.04). No associations or correlations were found regarding MP levels and SSc subtype, disease duration, presence of digital ulcers, modified Rodnan skin score, interstitial lung involvement or SSc specific autoantibodies.

**Conclusions:** The serum level of MP derived from platelets, endothelial cells and monocytes are elevated in SSc patients, indicating that MP might play a role in the pathogenesis of SSc. The lower MMP level observed in patients with more severe microangiopathy in capillaroscopy suggests that it might be a biomarker of microvascular damage in these patients.

**Figure:**
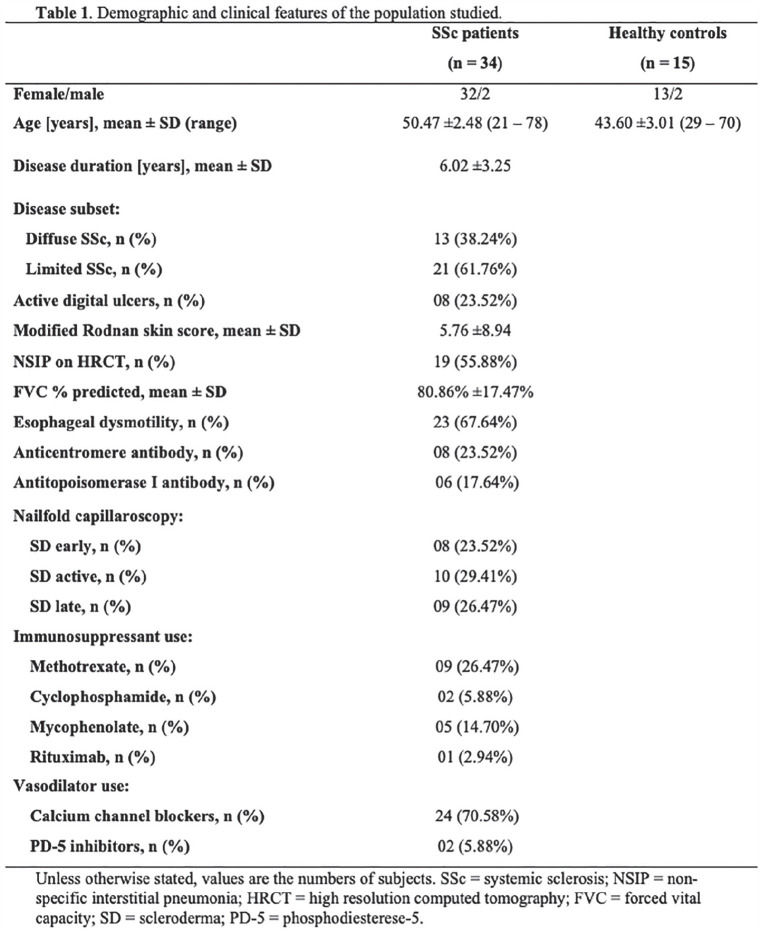


## P.260

## DIFFUSE CUTANEOUS SYSTEMIC SCLEROSIS (SSC) SERUM SIGNIFICANTLY IMPAIRS LYMPHANGIOGENESIS IN VITRO

M. Manetti^1^, E. Romano^1^, I. Rosa^1^, B.S. Fioretto^1^, S. Guiducci^1^, S. Bellando-Randone^1^, E. Pigatto^2^, F. Cozzi^2^, L. Ibba-Manneschi^1^, M. Matucci-Cerinic^1^

^1^*University of Firenze - Department of Experimental and Clinical Medicine, Firenze, Italy*, ^2^*University of Padova - Rheumatology Unit, Department of Medicine-DIMED, Padova, Italy*

**Introduction:** In SSc, despite the focus of several studies on blood microcirculation, lymphatic microvessels remain poorly investigated. In involved SSc skin, few clinical and histological reports underlined the presence of lymphatic circulatory abnormalities, such as decreased lymphatic vessel counts associated with a high risk of developing fingertip ulcers. Lymphatic vessel growth takes place through lymphangiogenesis, in which lymphatic endothelial cells (LECs) sprout, migrate, and proliferate to generate new lymphatic capillaries. This process is thought to be similar to angiogenesis, whose dysregulation is well established in SSc. The aim of this in vitro study was to explore for the first time whether sera from SSc patients may interfere with the lymphangiogenic process.

**Material and Methods:** Three lines of human adult dermal microvascular LECs (HMVEC-dLyAd) were challenged with sera from patients with early diffuse cutaneous SSc (n=8) and healthy subjects (n=8) and tested in different assays. Stimulation with recombinant human pro-lymphangiogenic vascular endothelial growth factor (VEGF)-C served as positive control. Cell proliferation and chemoinvasion were determined by WST-1 and Boyden chamber assays, respectively. Wound healing assay was performed on confluent cells grown in 6-well plates. In vitro lymphangiogenesis was evaluated after 48 hours of cell seeding on Geltrex matrix. Gene and protein expression levels of the lymphangiogenic orchestrators VEGF receptor-3 (VEGFR-3)/Flt-4 and neuropilin-2 (NRP-2) were analyzed by Real-Time PCR and Western blotting, respectively.

**Results:** Conditioning with SSc serum significantly reduced LEC proliferation compared with cells stimulated with healthy serum (p<0.001). Chemoinvasion and wound healing capacity were also greatly inhibited in LECs treated with SSc serum in respect to healthy serum (both p<0.001). The ability of LECs to form lymphatic capillaries on Geltrex was severely compromised in the presence of SSc serum (p<0.001 vs. healthy serum). Challenge with SSc serum resulted in a significant downregulation of either VEGFR-3/Flt-4 or NRP-2 mRNA and protein levels (p<0.001 vs. healthy serum for all comparisons).

**Conclusions:** In SSc, the pathologic environment severely hampers every lymphangiogenesis step, namely LEC proliferation, extracellular matrix invasion, migration and lymphatic tube formation. Likely, this may happen through the inhibition of pro-lymphangiogenic VEGFR-3/NRP-2 co-receptor signaling. The impairment of lymphangiogenesis opens a new scenario underlying SSc vascular pathophysiology which is worth investigating further.

## P.261

## THERMOGRAPHY OF THE TOES IN PATIENTS WITH SYSTEMIC SCLEROSIS AND RAYNAUD’S PHENOMENON

R.Z. Lim, J. Manning, T. Moore, S. Wilkinson, G. Dinsdale, A. Herrick

*University of Manchester- Department of Rheumatology in Salford Royal, Manchester, United Kingdom*

**Introduction:** Studies of Raynaud’s phenomenon (RP), including RP secondary to systemic sclerosis (SSc), have predominantly assessed the hands and few have assessed the toe vasculature. Our aim was to investigate the hypotheses that in patients with SSc:

a. The temperature gradient between the dorsum of the foot and toes (distal-dorsal difference, DDD) is ‘more negative’ (toes cooler) than in healthy controls.

b. That this temperature gradient is greatest along the first (great) toe.

c. That in individual patients, the severity of thermographic abnormality in the feet is associated with the severity of thermographic abnormality in the hands.

**Material and Methods:** Forty patients with SSc (27 limited cutaneous, 13 diffuse) and 20 healthy controls participated. Thermographic images of the dorsum of each hand and foot were captured using a Flir One thermal camera attached to an iphone 4. Temperature gradients (DDD) of the fingers (index, middle, ring and little) and toes (great toe and ‘others’) were measured. The data were analysed as follows:

1) Comparison of the temperature gradients of the great toes (right, left and average of both) and other toes (right, left and average of both) between patients and controls.

2) Comparison of the temperature gradient of the great toes (right and left) and other toes (right and left) on the same feet within patients and controls.

3) Association between temperature gradient of the hands (average of index, middle, ring and little of both hands) and that of the great toes (average of both feet) in patients.

**Results:** There was a trend for the great toes to be colder in patients with SSc, i.e. mean great toe DDD was more negative in patients (right: -2.89 °C, left: -2.91 °C, average: -2.9 °C) than in controls (right: -2.36 °C, left: -2.42 °C, average: -2.39 °C). Patients’ great toes (right: -2.89 °C, left: -2.91 °C) were colder than their ‘other toes’ (right: -2.58 °C, left: -2.63 °C), albeit not significantly. The temperature gradients along the fingers and toes of patients with SSc showed positive correlation, r(37)= 0.406, P= 0.01.

**Conclusions:** Our findings suggest that the great toe is the coldest in patients with SSc. Severe RP symptoms in the hands should prompt podiatry assessment and footcare education. Furthermore, mobile phone thermography was found to be a convenient tool for assessing the vasculature of fingers and toes.

## P.262

## CLASSIFICATION OF FINGER PULP BLOOD FLOW AND ASSESSMENT OF RESISTANCE INDEX OF RADIAL ARTERY IN PATIENTS WITH SYSTEMIC SCLEROSIS AND HEALTHY CONTROLS

A. Lescoat^1^, A. Ballerie^1^, F. Robin^2^, M. De Saint Riquier^2^, N. Belhomme^1^, A. Perdriger^2^, P. Jego^1^, G. Coiffier^2^, C. Cazalets^2^

^1^*CHU Rennes, Internal Medicine and Clinical Immunology, Rennes, France*, ^2^*CHU Rennes, Rheumatology, Rennes, France*

**Introduction:** Ultrasound evaluation with Power Doppler signal provides new outcome measures for the assessment of Systemic sclerosis (SSc)-associated vasculopathy. Resistance index (RI) and finger-pulp blood flow (FPBF) may constitute relevant markers to assess the impact of vasodilators on vascular involvement. The precise definitions and the relevance of these markers are still to be determined.

**Material and Methods:** A semi-quantitative ordinal scale evaluating FPBF was proposed, defining 5 stages of FPBF depending on the depth of positivity of Doppler signal on a sagittal view of the third finger (Cazalets’Classification of FPBF) : ranging from 0 (no signal detected) to 4 (Doppler signal detected at the sub-epidermis level). In this cross-sectional study, a PDUS evaluation of 30 healthy controls was performed, assessing the FPBF and RI of the radial artery. These measures were performed in basal conditions, after a warm bath and, lastly after a cold bath. These parameters were also assessed in basal conditions in 106 patients with SSc.

**Results:** In HC: RI of radial right radial artery was significantly lower after a warm bath in comparison with basal conditions and cold exposure (p<0.01). Changing from one stage of FPBF to a higher stage was associated with warm bath ((OR=3.8 ; IC95(1.15-12.71), p=0.024) and changing to a lower stage was associated with cold exposure (OR=16.78 ; IC95(2.00-140,90) p<0.001). The stages of FPBF were inversely correlated with RI in all conditions.

In SSc: A grade 0 of Cazalets’ classification was recorded in 20.4% of hands from SSc patients and 5% from HC (p<0.05). On the contrary, grade 3 was recorded in 50% of hands from HC and 34% in SSc patients (p<0.05). Patients with systemic sclerosis and history or a current ischemic digital ulcer (DU) were more likely to have a FPBF grade 0 (Grade 0 in 37.5% of patients with it and 10%in patients without, p<0.05). On the contrary, 5% of patients with DU had FPBF grade 4 in comparison with 34.7% in patients without DU (p<0.05).

Inter-rater agreement for the grading of FPBF was 0.82 and an intra-rater agreement was 0.71.

**Conclusions:** IR and FPBF as defined by the Cazalets’ classification seem relevant to assess the level of vasodilation. This classification seems to highlight some severity markers of the vasculopathy but needs to be externally validated.

## P.263

## COMBINATION VASODILATOR THERAPY IMPROVES SKIN MICROVASCULAR BLOOD BUT DO NOT RESTORE ENDOTHELIAL FUNCTION IN SYSTEMIC SCLEROSIS

C. Laurent^1^, G. Hariri^2^, J.-R. Lavillegrand^2^, S. Riviere^1^, J. Joffre^2^, H. Ait Oufella^2^, A. Mekinian^1^

^1^*Intenal mecidine - Hopital Saint-Antoine, Paris, France*, ^2^*Intensive care unit - Hopital Saint-Antoine, Paris, France*

**Introduction:** Systemic sclerosis is a chronic connective tissue disease, characterized by extensive fibrosis, autoimmunity and vascular disorders. Microvascular alterations due to reactive oxygen species generation, endothelial cells apoptosis and perivascular inflammation are responsible for systemic vasculopathy. Several vasodilating agents are available to treat peripheral microvascular disorders in scleroderma patients but the impact of vasodilating agents on microvascular functions remains poorly investigated. Here, we aimed to analyze skin microvascular reactivity of scleroderma patients according to vasodilating treatment, comparing patients receiving one vasodilator drug and patients receiving combination vasodilator therapy.

**Material and Methods:** We prospectively included consecutive patients hospitalized for systemic sclerosis in our internal medicine unit and healthy volunteers (medical students and fellows), in a 670-tertiary bed teaching hospital. Skin microvascular function was measured in the forearm area using acetylcholine iontophoresis flowmeter coupling to laser Doppler, as previously described [5]. Briefly, basal skin blood flow was recorded, and then three acetylcholine simulations were proceeded, leading to endothelium-dependent vasodilatation and consecutive increased local blood flow (Figure 1). We measured skin blood flow at baseline and the maximum increase (% from baseline) after acetylcholine simulation.

**Results:** Nineteen consecutive scleroderma patients and 13 healthy volunteers were included.Baseline skin blood flow was comparable between healthy volunteers and scleroderma patients. After acetylcholine simulation, blood flow increase was significantly higher in healthy volunteers compared to scleroderma patients. Patients under combination therapy had significantly higher baseline skin blood flow but displayed 2-fold lower microvascular reactivity after acetylcholine simulation . We found that Iloprost led to an improvement of microvascular reactivity at day 3 and day 5.

**Conclusions:** Our study suggests that chronic vasodilator treatment improves skin microvascular blood flow but did not fully restore endothelial microvascular function in scleroderma patients. Specifically targeted therapies are still needed to improve endothelial function in patients with systemic sclerosis.

**Figure:**
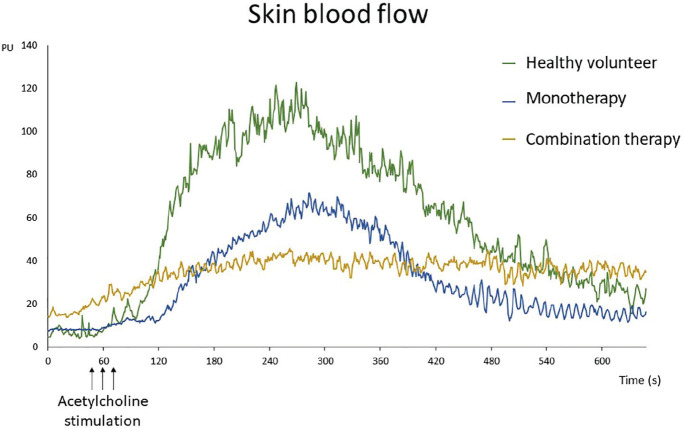


## P.264

## EPIGENETIC REGULATION MEDIATED REDUCTION IN THE EXPRESSION OF PROSTACYCLIN RECEPTOR (IP) AND PROSTACYCLIN SYNTHASE (PTGIS) IN SCLERODERMA SKIN, VASCULAR SMOOTH MUSCLE CELLS (VSMCS), AND MICROVASCULAR

B. Kahaleh, Y. Wang, N. Altorok

*University of Toledo, Toledo, USA*

**Introduction:** Progressive functional and structural vascular disorder is one of the hallmark features of Systemic Sclerosis (Scleroderma, SSc). In this study we examine the expression levels of PTGIS and IP in normal and SSc skin, and in MVECs and vSMCs. We also investigated the effects of the DNA methyltransferase inhibitor 5-Aza-deoxycytidine (Aza), and the histone deacetylase inhibiter trichostatin (TSA) on PTGIS and IP gene expression in SSc and normal MVECs and vSMCs.

**Material and Methods:** MVECs and vSMCs were isolated from involved skin and matched healthy subjects. The expression levels of IP and PTGIS were measured by qPCR and western blot analysis (WB). Epigenetic inhibitors were added to cell cultures in order to assess the role of epigenetic regulation on IP and PTGIS expression levels. SSc-MVECs and SSc-VSMCs are treated with Aza at 5uM for 5 days and TSA at 100nM for 1 day. The mRNA and protein expression levels were detected by qPCR and WB respectively.

**Results:** Results:

The mRNA expression levels of PTGIS and IP were significantly downregulated in SSc-skin by 0.183 fold ± 0.03 for PTGIS ( P < 0.01) and 0.54 fold ± 0.06 for IP (P < 0.01), compared to control skin. The mRNA expression levels of PTGIS and IP were also decreased in SSc-vSMCs, compared to control (0.29 fold ± 0.04, P< 0.01 for PTGIS; 0.46 fold ± 0.06 for IP, P<0.01). WB analysis demonstrated similar reduction on the protein levels in SSc -vSMCs. Addition of Aza and TSA resulted in increased expression levels of IP and PTGIS in SSc-vSMCs to almost normal levels. SSc-MVECs also exhibited lower expression levels of PTGIS than control MVECs at both mRNA level (0.214 fold ± 0.03, P< 0.01) and protein level (0.49 fold ± 0.05, P<0.05). The addition of Aza and TSA corrected the reduced mRNA and protein expression levels of PTGIS in SSc-MVECs.

**Conclusions:** The data demonstrate defective PGI2-IP pathway in SSc-MVECs and vSMCs. This defect is likely to contribute to the vascular dysfunction in SSc through reduction in the vasodilatory PGI2/IP signaling pathway resulting in enhanced vasospasm and in activation of SSc-vSMCs and vascular remodeling. The addition of Aza and TSA corrected the reduced expression levels of PTGIS and IP suggesting that epigenetic regulation influence IP and PTGIS gene expression in SSc. The upregulation of IP and PTGIS expression combined with the use of PGI2 analogue may be an important new therapeutic strategy for SSc.

## P.265

## PREDICTORS OF NEW DIGITAL ULCERS IN SYSTEMIC SCLEROSIS; RESULTS FROM BEST-FITTED MODEL OF MULTIPLE REGRESSION ANALYSIS

A. Javinani, F. Gharibdoost, A.R. Jamshidi, Z. Tamartash, H. Kavosi

*Rheumatology Research Center, Tehran, Iran*

**Introduction:** Systemic Sclerosis (SSc) is a systemic autoimmune disorder characterized by a pathologic triad of fibrosis, vasculopathy, and immune system dysregulation. Digital ulcers are the hallmark of the SSc-related vasculopathy with considerable morbidity and treatment challenges. Knowing the risk factors of digital ulcers make easier to find at-risk patients who are the best candidates for preventive treatments. The present research aimed to find the predictors of new digital ulcers in SSc patients.

**Material and Methods:** In the present study, records from 660 patients in Iranian SSc registry were used. In the database, 572 patients had complete records to enter the study. Twenty-three variables were considered for the primary analysis. These variables consisted of demographic features, clinical manifestations, laboratory parameters, and serologic data. The association of new-onset digital ulcers with these variables were investigated. The variables with significant associations were entered to the multiple regression analysis. By using the forward likelihood ratio method, the best-fitted model for prediction of new digital ulcers was extracted.

**Results:** Findings from the primary analyses revealed eleven statistically significant associations between new digital ulcer and our 23 selected variables. The significant associations were obtained with age at disease onset, body mass index, modified Rodnan skin score (MRSS), hemoglobin level, history of the previous ulcer, telangiectasia, flexion contracture of the hands, SSc cutaneous subtype, gastrointestinal manifestations, interstitial lung disease (ILD) and anti-topoisomerase antibody. After including these eleven independent variables into multiple regression analyses, five variables were retained in the final model. According to this model; presence of imaging-proven ILD (P-value: 0.003), history of previous digital ulcers (P-value: 0.006), younger age at onset (P-value: 0.012), higher MRSS (P-value: 0.018), and lower hemoglobin level (0.049) were the significant predictors of digital ulcers. The overall predictive accuracy of this model was 88.4% with the prediction sensitivity, specificity, and R2 of 98.7%, 33.3% and 0.365, respectively.

**Conclusions:** These findings can be used to develop targeted interventions aimed at reducing the development of new ulcers in vulnerable SSc patients. A key priority should therefore be to plan for the long-term care of patients with prior history of digital ulcer and for whom with ILD, anemia and higher MRSS.

## P.266

## EXPLORING PATIENTS’ PERCEPTIONS AND BELIEFS ON DIGITAL ULCER PATHOGENESIS IN SYSTEMIC SCLEROSIS

M. Hughes^1^, J. Pauling^2,3^, J. Jones^4^, C. Denton^5^, R. Domsic^6^, T. Frech^7^, A. Herrick^1,8^, D. Khanna^9^, M. Matucci-Cerinic^10^, L. McKenzie^11^, L.A. Saketkoo^12^, R. Gooberman-Hill^4,13^, A. Moore^4^

^1^*Centre for Musculoskeletal Research, Faculty of Biology, Medicine and Health, Manchester, United Kingdom*, ^2^*Royal National Hospital for Rheumatic Diseases (at Royal United Hospitals), Bath, United Kingdom*, ^3^*Department of Pharmacy and Pharmacology, University of Bath, Bath, UNITED KINGDOM*, ^4^*Musculoskeletal Research Unit, Translational Health Sciences, Bristol Medical School, Bristol, United Kingdom*, ^5^*Department of Rheumatology, Royal Free Hospital, University College London, London, United Kingdom*, ^6^*University of Pittsburgh Medical Center, Pittsburgh, USA*, ^7^*University of Utah and Salt Lake Veterans Affair Medical Center, Salt Lake City, USA*, ^8^*Salford Royal NHS Foundation Trust, Manchester Academic Health Science Centre, Manchester, United Kingdom*, ^9^*Scleroderma Program, University of Michigan, Michigan, USA*, ^10^*Division of Rheumatology, University of Florence, Florence, Italy*, ^11^*Patient representative, Contact via Professor Herrick, The University of Manchester, Manchester, United Kingdom*, ^12^*Tulane University School of Medicine, New Orleans Scleroderma & Sarcoidosis Patient Care & Research Center, New Orleans, USA*, ^13^*NIHR Bristol Biomedical Research Centre, University Hospitals Bristol NHS Foundation Trust, Brisol, United Kingdom*

**Introduction:** Digital ulcers (DUs) are common in patients with systemic sclerosis (SSc) and often refractory to treatment. However, little is known about the patient perspective around pathogenesis and natural history of DU disease (including emergence and evolution) which presents a significant barrier to developing new ulcer treatments. Understanding patient perceptions and beliefs could provide novel insight into DU pathogenesis, including to inform clinical trial design.

**Material and Methods:** Patient focus groups (FGs) were undertaken across the United Kingdom to explore the patient experience of SSc-DUs. Participants were enrolled according to a purposive sampling framework and FGs conducted using a standardised topic guide. FGs were audio recorded, transcribed, anonymised, and analysed using inductive thematic analysis. The present study reports patient perceptions and beliefs about DU pathogenesis and natural history.

**Results:** Twenty-nine patients with SSc participated in 4 FGs across the UK. The majority of patients were female (n=20) and had the limited subset (n=20) of the disease. The mean (SD) age of patients was 59.9 (13.3) years. Patients had a spectrum of previous DU disease: A solitary previous DU (n=3), 2-4 previous DUs (n=9) and >5 previous DUs (n=17).

Three major themes emerged:

Theme 1 – Underlying causes of SSc-DUs: Most participants believed there was a reason for an ulcer to develop. These comprised ‘external’ and ‘internal’ precipitating factors. ‘External’ causes included trauma, exposure to chemicals or infection, cold or change in temperature, and from cuts or skin splitting. ‘Internal’ causes were Raynaud’s phenomenon including from ‘poor circulation’, calcinosis, and due to previous ulcers.

Theme 2 – Symptoms prior to SSc-DU emergence: The majority of participants reported they could recognise when an ulcer was about to emerge. The most common symptom was pain below the skin, often like an internal pressure effect. Some participants also reported physical skin signs (e.g. a white patch) which would subsequently breakdown/ulcerate.

Theme 3 – Features present at the time of SSc-DU emergence: This was variable between participants. Surface characteristics ranged from being moist (including overt pus) to dry with a superficial crust, and occasionally with a central ‘core’. Associated pain during ulcer healing varied significantly between participants.

**Conclusions:** Our study provides novel patient-perceived insights into the pathogenesis and natural history of SSc-DU. SSc-DUs are not a random occurrence and many patients can explain and even predict the development of ulcers. Of importance to clinical trial design, ulcer symptoms can significantly differ before and after the emergence of SSc-DUs.

## P.267

## DIGITAL ULCER DEBRIDEMENT IN SYSTEMIC SCLEROSIS: A SYSTEMATIC LITERATURE REVIEW

M. Hughes^1^, B. Alcacer-Pitarch^2,3^, A.M. Gheorghiu^4^, E. Praino^5^, R.D. Sandler^1^, Y. Tavor^6^, C. Bruni^7^, M. Matucci-Cerinic^7^

^1^*Department of Rheumatology, Royal Hallamshire Hospital, Sheffield Teaching Hospitals NHS Foundation Trust, Sheffield, United Kingdom*, ^2^*Leeds Institute of Rheumatic and Musculoskeletal Medicine, University of Leeds, Leeds, United Kingdom*, ^3^*Rheumatology Department, Leeds Teaching Hospital NHS Trust, Leeds, United Kingdom*, ^4^*Department of Internal Medicine and Rheumatology, Cantacuzino Hospital, Carol Davila University of Medicine and Pharmacy, Bucharest, Romania*, ^5^*Rheumatology Unit, Department of Emergency and Organ Transplantation, University of Bari, Bari, Italy*, ^6^*B. Shine Rheumatology Unit, Rambam Health Care Campus, Rappaport Faculty of Medicine, Technion, Israel*, ^7^*Department of Experimental and Clinical Medicine, Division of Rheumatology, University of Florence, Florence, Italy*

**Introduction:** Optimal wound care is an essential component in the management of SSc-digital ulcers (DUs). DU debridement has been suggested to reduce ulcer-related pain and improve tissue healing by removing necrotic (devitalised) tissue and releasing trapped pus. Sharp debridement is performed using a scalpel, whereas, autolytic debridement uses dressings to optimise endogenous tissue lysis. However, at present, only a minority of rheumatologists perform ulcer debridement in their routine clinical practice and due to the lack of scientific evidence there is no agreed standard of care/debridement protocol.

Against this background, our two objectives were to: (i) systematically review the literature to evaluate the current evidence for the use of debridement in DU management, and (ii) assess whether there are any specific debridement protocols.

**Material and Methods:** A systematic literature review was performed by searching the PubMed database (between 01/01/1950 to 31/12/2018) in accordance with PRISMA guidelines. Articles in English which focussed on DU debridement/curettage were eligible for inclusion in our review. Exclusion criteria included (but were not limited to) studies of juvenile/paediatric SSc patients and basic/non-clinical research. Two independent reviewers both screened and extracted the abstracts/full manuscripts.

**Results:** Our search identified 1497 studies of which 1408 remained after duplicates were removed. 89 full-text articles were screened for eligibility. Four studies were included in our final analysis. The majority (n=3) were case series and with one non-randomised clinical trial. The mean number of included patients was 32 (range of 6 to 76). The majority of patients (reported in three studies) were on oral drug therapies for SSc digital vasculopathy. Two studies examined chronic DU (minimum of 2 months duration).

Three studies used scalpel debridement and one study used autolytic debridement. No studies specifically reported the effect on DU healing from debridement. Autolytic debridement with hyaluronate-based products can be associated with significant ulcer pain and inflammation. Local anaesthetic (opioid) significantly reduces pain both during and after the ulcer debridement procedure. Combined local and oral analgesia is often required for more severe or infected DUs.

**Conclusions:** DU (scalpel and autolytic) debridement is being used by some clinicians in rheumatology; however, there is no standardised debridement protocol. To improve wound-care for DUs in SSc, future research should focus on developing a standardised protocol for DU debridement, with a view to facilitate randomised controlled trials to demonstrate treatment efficacy.

## P.268

## A MULTI-CENTRE QUALITATIVE STUDY EXPLORING THE PATIENT EXPERIENCE OF DIGITAL ULCERS IN SYSTEMIC SCLEROSIS

M. Hughes^1^, J. Pauling^2,3^, J. Jones^4^, C. Denton^5^, R. Domsic^6^, T. Frech^7^, A. Herrick^1,8^, D. Khanna^9^, M. Matucci-Cerinic^10^, L. McKenzie^11^, L.A. Saketkoo^12^, R. Gooberman-Hill^13^, A. Moore^13^

^1^*The University of Manchester, Centre for Musculoskeletal Research, Manchester, United Kingdom*, ^2^*Royal National Hospital for Rheumatic Diseases (at Royal United Hospitals), Bath, United Kingdom*, ^3^*Department of Pharmacy and Pharmacology, University of Bath, Bath, United Kingdom*, ^4^*Musculoskeletal Research Unit, Translational Health Sciences, Bristol Medical School, Bristol, United Kingdom*, ^5^*Department of Rheumatology, Royal Free Hospital, University College London, London, United Kingdom*, ^6^*University of Pittsburgh Medical Center, Pittsburgh, USA*, ^7^*University of Utah and Salt Lake Veterans Affair Medical Center, Salt Lake City, USA*, ^8^*Salford Royal NHS Foundation Trust, Manchester Academic Health Science Centre, Manchester, United Kingdom*, ^9^*Scleroderma Program, University of Michigan, Michigan, United Kingdom*, ^10^*Division of Rheumatology, University of Florence, Florence, Italy*, ^11^*Patient representative. Contact via Professor Herrick, The University of Manchester, Manchester, United Kingdom*, ^12^*Tulane University School of Medicine, New Orleans Scleroderma & Sarcoidosis Patient Care & Research Center, New Orleans, USA*, ^13^*NIHR Bristol Biomedical Research Centre, University Hospitals Bristol NHS Foundation Trust, Bristol, United Kingdom*

**Introduction:** Digital ulcers (DUs) are a major cause of disease-related morbidity and a difficult to treat vascular complication of systemic sclerosis (SSc). Demonstrating treatment efficacy has traditionally focussed upon clinician assessment of DUs alone. No existing patient reported outcome (PRO) instruments fully capture the multi-faceted impact of SSc-DU. Against this background, the aim of this study was to comprehensively explore the themes and subthemes that comprise the patient experience of SSc-DU.

**Material and Methods:** Patient focus groups (FGs) were undertaken at 3 scleroderma centres across the United Kingdom, following a topic guide devised by SSc patients, experts and experienced qualitative researchers. A purposive sampling framework ensured the experiences of a diverse group of patients were captured. FGs were audio recorded, transcribed, anonymised, and analysed using inductive thematic analysis.

**Results:** Twenty-nine patients with SSc participated in 4 FGs across the UK. The majority of patients were female (n=20) and had the limited subset (n=20) of the disease. The mean (SD) age of patients was 59.9 (13.3) years. Patients had a spectrum of previous DU disease: 1 previous DU (n=3), 2-4 previous DU (n=9) and >5 previous DU (n=17). Many patients were receiving treatment with vasodilator medication for SSc-vasculopathy (including DU disease). These included calcium channel blocker (n=10), phosphodiesterase type-5 inhibitor (n=18) and endothelin receptor antagonist (n=9).

Five major inter-related themes (and sub-themes) were identified which encompass the patient experience of SSc-DUs. Theme 1 - ‘Physical symptoms and signs’: pain, sensitivity & loss of hand function. Theme 2 - ‘Psychological impacts’: fear, anxiety, embarrassment, low mood, constant vigilance & visual appearance. Theme 3 – Functional impacts: hand function, self-grooming, working, relationships with others, socialising, hobbies & sleep disturbance. Theme 4 – Aggravating factors: number of ulcers, location of ulcers, time to heal, rate of ulcer recurrence, trauma & temperature. Theme 5 - Mitigating factors: effective treatments, aids & devices, coping strategies, self-management, adaptations & support from others.

**Conclusions:** The patient experience of SSc-DU is multi-faceted and comprises a complex interplay of experiences associated with significant morbidity. Ours is the first study to examine the multi-faceted patient experience of SSc-DUs which is not captured using existing SSc-DU outcomes. Our findings shall inform the development of a novel PRO instrument to assess the severity and impact of SSc-DUs for use in future SSc-DU clinical trials.

## P.269

## EFFECT OF BIOFEEDBACK ON RAYNAUD’S SYNDROME IN SYSTEMIC SCLEROSIS PATIENTS

R. Grekhoff

*Zborovsky’ Research Institute for clinical and experimental rheumatology, Volgograd, Russia*

**Introduction:** Various methods of complementary medicine were offered for systemic sclerosis (SS) patients, but any of them does not allow achieving desirable success. One of the promising methods is biofeedback.

Our aim was to improve the efficiency of complex therapy of Raynaud’s syndrome in systemic sclerosis patients by means of biofeedback training.

**Material and Methods:** We observed 90 SS patients. The middle age of patients was equal to 38,19±12,1 years, average duration of illness 11,2±3,4 years.

SS patients have been divided on two groups randomly: the basic (n=60) that received 12-14 sessions of multimodal (temperature and EEG) biofeedback training using the Reacor rehabilitation complex and control group (n=30).

Clinical assessment of biofeedback efficiency was assessed by dynamics of following clinical and laboratory indices: skin count by G.P.Rodnan, capillaroscopies of nails, pain by VAS, joint count, number of swelling joints, swallow index, ESR, antibodies to Scl-70, and MOS SF-36 questionnaire was applied for quality of life evaluation.

**Results:** Reliable changes of capillaroscopy index (morphological variations of capillary tubes (χ2=6,19, p=0,013), a pain by VAS, ESR were noted in control group of SS patients after the course of conventional therapy. At the same time reliable positive dynamics of the skin count, joint count, number of swelling joints, swollen index, ESR was founded in patients of basic group after biofeedback therapy. Besides, capillaroscopy indices, namely angiectasia (χ2=8,192, p=0,004), morphological variations of capillary tubes (χ2=4,14, p=0,042) and hemorrhages (χ2=7,906, p=0,005) were reliable improved after the treatment. There is the evidence that biofeedback therapy has positive influence on microcirculation disturbances.

**Conclusions:** Thus, results of the researches are the evidence of considerable efficiency of biofeedback in complex therapy of SS patients and its positive influence on capillaroscopy indices, clinical and laboratory indices of the disease. Use of biofeedback training will allow improving results of treatment of this disease essentially.

## P.270

## INVOLUNTARY WEIGHT LOSS AND BEING UNDERWEIGHT ARE INDEPENDENTLY ASSOCIATED WITH AN INCREASED RISK OF RAYNAUD’S PHENOMENON IN BOTH MEN AND WOMEN

A. Eman Abdulle^1^, S. Arends^2^, H. van Goor^3^, L. Brouwer^2^, A. M. van Roon^1^, J. Westra^2^, A. Herrick^4^, K. de Leeuw^2^, D. J. Mulder^1^

^1^*Department of Internal Medicine, University Medical Center Groningen, Groningen, The Netherlands*, ^2^*Department of Rheumatology, University Medical Center Groningen, Groningen, The Netherlands*, ^3^*Department of Pathology and Medical Biology, University Medical Center Groningen, Groningen, The Netherlands*, ^4^*Division of Musculoskeletal & Dermatological Sciences, The University of Manchester, Manchester, United Kingdom*

**Introduction:** The origin of Raynaud’s phenomenon is multifactorial and complex. Although it is considered common knowledge, clinicians often underestimate the role of low body weight, weight loss, and dietary habits as easily identifiable and putatively modifiable causes of RP. The current study aims to investigate the association between BMI, involuntary weight loss, nutritional restriction diets and RP.

**Material and Methods:** Data from the population based Lifelines cohort were analyzed, in which participants completed the self-administered connective tissue disease questionnaire. Subjects who reported cold-sensitive fingers and bi- or triphasic colour changes were considered to suffer from RP. Patient characteristics, anthropometric measurements (e.g., BMI categorized in 2 groups: <18.5 kg/m2 (underweight), BMI>18.5 kg/m2), recent weight loss, and information on dietary assessment were collected. Statistical analysis was stratified for gender. In the multivariate analyses we correction for known confounders (e.g., age, creatinine level, daily caloric intake, hormonal status (in women)).

**Results:** In total, 93,935 participants completed the questionnaire. Prevalence of RP was 4.2% [95% CI 4.1-4.4], and was three-fold higher in women. Subjects with RP had a significantly lower daily caloric intake, as compared to subjects without RP (p<0.001).The multivariate analyses revealed that BMI (men: OR 0.86 [95% CI 0.84-0.88], p<0.001; women: OR 0.90 [95% CI 0.89-0.91], p<0.001), BMI dichotomized (BMI<18.5: men: OR 5.76 [95% CI 2.93-11.33], p<0.001; women: OR 3.14 [95% CI 2.40-4.10], p<0.001), and involuntary weight loss (men: OR 1.32 [1.17-1.48], p<0.001; women: OR 1.31 [1.20-1.44], p<0.001) were independently associated with the presence of RP. In addition, low-fat diet was also found to be associated with an increased risk of RP in women (OR 1.27 [1.15-1.44], p<0.001), but not in men.

**Conclusions:** We demonstrated that being underweight and involuntary weight loss are independently associated with an increased risk of RP in both men and women. The current study further emphasizes that BMI and weight loss are easily identifiable and potentially modifiable risk factors of RP, and should be structurally assessed and monitored in subjects with RP.

## P.271

## ELEVATED SERUM ENDOTHELIN -1 LEVELS IN PREECLAMPSIA ARE NOT ASSOCIATED WITH LONG-TERM MICROVASCULATURE DAMAGE AS EVALUATED BY NAILFOLD CAPILLAROSCOPY

V. Dalm^1^, J. Roeters van Lennep^2^, J. Duvekot^3^

^1^*Department of Internal Medicine, Division of Clinical Immunology, Erasmus Univeristy Medical Center, Rotterdam, The Netherlands*, ^2^*Department of Internal Medicine, Division of Vascular Medicine, Erasmus Univeristy Medical Center, Rotterdam, The Netherlands*, ^3^*Department of Obstetrics and Gynaecology, Erasmus University Medical Center, Rotterdam, The Netherlands*

**Introduction:** Preeclampsia is a disorder of pregnancy characterized by new-onset hypertension and proteinuria after gestational week 20. Placental ischemic events and the release of placental factors play a crucial role in the pathophysiology of preeclampsia. These factors contribute to a generalized systemic vascular endothelial dysfunction and result in increased systemic vascular resistance and hypertension. One of the factors involved is endothelin-1, which is also elevated in patients with systemic sclerosis and which is associated with vasculopathy in these patients. Women affected by preeclampsia have an increased risk to develop cardiovascular disease later in life as compared with women with normotensive pregnancies. We hypothesized that elevated endothelin-1 levels during preeclampsia result in microvasculature damage which may contribute to the increased risk of cardiovascular disease.

**Material and Methods:** Ten women, admitted at the department of Obstetrics and Gynaecology of Erasmus University Medical Center because of severe preeclampsia were included in this study. Serum endothelin-1 levels were evaluated by ELISA and nailfold capillaroscopy was performed on day of admission. Eight fingers, excluding both thumbs, were evaluated by the same researcher. Six months after delivery both measurements were repeated. Serum endothelin-1 was also measured in 10 normotensive pregnancies.

**Results:** Serum endothelin-1 levels were significantly higher in pregnancies complicated by preeclampsia, when compared to normotensive pregnancies (68.0 ± 7.1 pg/L versus 39.3 ± 5.6 pg/L, P<0.05). Nailfold capillaroscopy images were all found to be normal on admission, with no signs of microvasculature damage. 6 months after delivery all 10 patients were re-evaluated and serum endothelin-1 levels were 24.6 ± 6.6 pg/L. Nailfold capillaroscopy findings were all normal, with again no signs of microvasculature damage.

**Conclusions:** Short-term elevation of serum endothelin-1 during preeclampsia is not associated with long-term microvasculature damage as evaluated by nailfold capillaroscopy. Nailfold capillaroscopy therefore seems not to be a useful tool to predict long-term complications of preeclampsia

## P.272

## THE ROLE OF CANNABIDIOL OIL IN THE MANAGEMENT OF SYSTEMIC SCLEROSIS DIGITAL ULCER-RELATED PAIN

L. Magnani^1^, A. Spinella^2^, E. Cocchiara^2^, F. Lumetti^2^, G. Bajocchi^1^, C. Salvarani^2^, D. Giuggioli^2^

^1^*Rheumatology Unit, AUSL-IRCCS of Reggio Emilia, Reggio Emilia, Italy*, ^2^*Scleroderma Unit, Chair of Rheumatology, University of Modena and Reggio Emilia, Italy, Modena, Italy*

**Introduction:** Systemic Sclerosis (SSc) is an autoimmune disease, characterized by fibrosis of the skin and internal organs. Digital ulcers (DUs), one of the most frequent complications of SSc, are frequently recalcitrant to standard therapy and are related to severe chronic or acute pain.

We evaluated the efficacy and safety of Cannabidiol (CBD) in managing DUs-related pain.

**Material and Methods:** From January 2018 to December 2018 we enrolled 25 SSc patients with DUs and 20 patients as control group. All patients fulfilled the EULAR/ACR classification criteria for SSc painful. DUs were resistant to standard therapy. Clinical assessment of subjective symptoms due to background pain, as well as procedural pain (during local management of DUs), were performed at baseline and during therapeutic procedures. The CBD (10% CBD oil) was administered orally every day for the treatment of DUs-related chronic pain and locally (two drops, equivalent to 20 mg) during surgical debridement and medication of DUs.

**Results:** Local treatment with CBD produced a significant reduction of the DUs-related pain since the first month of therapy [pain VAS decreased from 94.8 ± 8.72 SD to 54.7 ± 9.4 SD (P < 0.0001), total hours of sleep increased from 2.56 ± 1.28 SD to 5.67 ± 0.85 SD (P < 0.0001)]. Additional analgesic therapy was necessary in 12/25 patients: 4/12 assumed paracetamol, 2/12 paracetamol plus codeine, 4/12 oxycodone, 2/11 morphine. After 2 months of therapy, further clinical improvement was observed. The HAQ-DI decreased from 1.1 ± 0.67 SD (baseline) to 0.46 ± 0.46 SD. With CBD treatment the patient’s compliance to the local wound management, especially to surgical debridement, markedly improved, with amelioration of procedural pain. The majority of the DUs (20/25) healed in the treated group, while in the control group the complete healing of the skin lesion was observed in 8/20 (40%). Any severe adverse event was noted, except for itch and perilesional erythema referred by 7/25 (28%). No patient discontinued the CBD treatment due to severe infection while in the control group 4 patients need antibiotic therapy for DUs infections.

**Conclusions:** CBD oil administered locally might be a useful tool to manage chronic procedural pain in SSc-related DUs.

## P.273

## NEUTROPHIL TO LYMPHOCYTE RATIO AND MICROCIRCULATION IMPACT IN PATIENTS WITH SYSTEMIC SCLEROSIS – A CROSS-SECTIONAL STUDY

V. Bernardino, A.C. Rodrigues, M. Fernandes, A. Lladó, A. Panarra

*Hospital Curry Cabral - Centro Hospitalar Universitário Lisboa Central, Lisbon, Portugal*

**Introduction:** Neutrophil to lymphocyte ratio (NLR) is usually applied as a prognostic marker in cardiovascular disease, infections, inflammatory disorders or cancers. Physiopathology of systemic sclerosis (SSc) is mediated by inflammation, so NLR could act as a predictor of morbidity and/or mortality. In this study, we aimed to identify a possible relation between NLR and a worse microvascular damage, through direct visualisation by nailfold capillaroscopy (NFC).

**Material and Methods:** This is a retrospective cross-sectional study. Clinical data from SSc patients on our cohort was consulted. Data was anonymized when introduced on the database. Only patients who fulfilled the 2013 SSc classification criteria were included. NFC was performed using a Videocap biomicroscope, version 3.0. NLR ratio was considered normal between 0,78 and 3.53. White blood count (WBC) that was used to calculate the NLR was the most possibly concomitant to the NFC exam procedure. NFC scleroderma patterns were classified as early, active and late. The statistical analyse used the SPSS software. Chi-square test with Pearson´s correction was used to test correlation between variables, considering p < 0,05.

**Results:** Overall, 30 patients met the SSc criteria, 29 were female and the mean age was 61,4 years. About 76,7% of patients presented normal values of NLR. NFC patterns distributed as early (23,3%), active (36,7%) and late (40,0%). Although most patients who presented late NFC scleroderma pattern had a high NLR, no correlation was established (p = 0,058).

**Conclusions:** The NLR values used on our population were not adjusted for elderly subjects, which can create a bias in the interpretation of the results. Besides, WBC can also depend on the patient’s metabolic status. NLR does not accurately relate to microvascular damage in SSc, which accords with the suggestions that capillary abnormalities arise in an early phase of the disease. Therefore, a patient may have a late scleroderma pattern without an active systemic inflammation status. However, more studies, with larger sample sizes, are needed to clarify our hypothesis.

## P.274

## NON-SCLERODERMA CAPILLAROSCOPIC PATTERN IN PATIENTS WITH RAYNAUD’S PHENOMENON AND POSITIVE ANTIBODIES FOR SYSTEMIC SCLEROSIS

V. Bernardino, A.C. Rodrigues, M. Fernandeds, A. Lladó, A. Panarra

*Hospital Curry Cabral - Centro Hospitalar Universitário Lisboa Central, Lisbon, Portugal*

**Introduction:** Systemic Sclerosis (SSc) evolves due to endothelial damage, immune dysregulation and fibrosis. Microvascular damage is usually visualized by nailfold capillaroscopy (NFC), being the scleroderma pattern (SP) the most frequent among these patients. In this study, we aimed to characterize NFC abnormalities in patients with Raynaud’s phenomenon (RP) and positive SSc antibodies, with a non-scleroderma pattern (NSP) in NFC, and correlate them to particular clinical or immunological aspects.

**Material and Methods:** This is a retrospective study. Patients with RP, NSP on NFC and positive SSc antibodies (anti-centromere (A-Cm), anti-Scl70 (A-Scl70) and anti-RNA polymerase III (A-RNAP)) were included. NFC results were identified in our database and clinical files were consulted, after data anonymization. Patients were separated in groups for 3 different type analysis: clinics (SSc symptoms, morphoea or no symptoms related to scleroderma); immunology (Cm, Scl70, RNAP) and result of previous NFC (SP or NSP). NFC were performed using Videocap Biomicroscope version 3.0 and images were obtained using a magnification of x200. Data was analyzed using SPSS software. Statistics included Fisher exact test for qualitative variables and Mann-Whitney two tailed test for quantitative variables - morphoea and A-RNAP values were excluded from this analysis due to its small sample size.

**Results:** Results are presented in table 1. Overall, 43 patients were included, in which 39 were female with a mean age of 50,2 (±32) years. Clinical aspects of scleroderma were found in 33% patients, morphoea in 7% and no SSc associated symptoms in 60%. In this group, mean density was higher in morphoea patients, as well as the NFC score. Hemorrhages and dilated limbs were more prevalent in SSc patients. A positive correlation was found between scleroderma symptoms and haemorrhages on NFC (p = 0,004). Main antibodies were A-Cm in 70%, A-Scl70 in 26% and A-RNAP in 4%. Among these patients, hemorrhages and dilated limbs were more frequent in A-Cm patients, but neoangiogenesis was most detected on A-Scl70. No correlations were found in this group. Only 9 patients had a previous NFC, 4 with SP and 5 with NSP. Previous NFC with SP correlated with dilated loops.

**Conclusions:** Although scleroderma symptoms and antibodies such as A-Cm, A-Scl70 and A-RNAP are very specific for SSc, related microvascular damage doesn’t necessarily evolve through giant capillaries and worse NFC findings. In patients with NSP, SSc symptoms were related with haemorrhage, which suggests vascular damage. Appropriate treatment can ameliorate microcirculation status, with SP turning into NSP on NFC, as disclosed.

**Figure:**
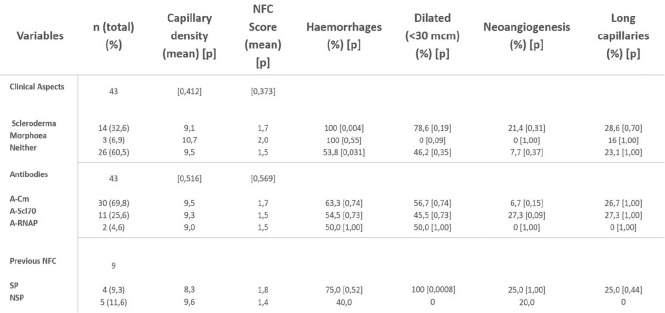


## P.275

## TREATMENT WITH INTRAVENOUS ILOPROST IN A MONOCENTRIC COHORT OF PATIENTS WITH SSC: COMPARISON BETWEEN TWO DIFFERENT THERAPEUTIC REGIMENS

S. Barsotti^1^, M. Di Battista^1^, V. Venturini^1^, S. Bilia^1^, L. Puccetti^1^, V. Lorenzoni^2^, A. Della Rossa^1^, G. Turchetti^2^, M. Mosca^1^

^1^*Rheumatology Unit - Pisa University Hospital, Pisa, Italy*, ^2^*SSSUP, Pisa, Italy*

**Introduction:** Iloprost, a stable synthetic analog of prostacyclin, is a potent vasodilator commonly prescribed in patients with SSc-digital ulcers (DUs). Iloprost is administered intravenously but different schemes have been proposed without clear superiority. The aim of our study was to compare two different administration schedules of Iloprost to identify differences in term of effectiveness, tolerability and costs.

**Material and Methods:** Forty-four patients classified with SSc according to the 2013 EULAR/ACR criteria were enrolled (44 females, 15 diffuse-SSc, 32 limited-SSc, mean age 60 years, mean disease duration 14.6 years). Iloprost was administered in the same patients with two different therapeutic schemes: from October 2014 to May 2015 with a continuous intravenous infusion (inpatient clinic - duration 48 hours) of 100 µg, and from October 2015 to May 2016 with intermittent infusion for two consecutive day of 50 µg (day-care - duration 3-5 hours/day). Data collected included: modified-Rodnan Skin Score (mRSS), patient’s VAS for Raynaud’s phenomenon, number of DUs, digital pitting scars (presence), number and type of adverse events (AE), concomitant vasoactive drugs (calcium channel blockers-CCB, phosphodiesterase-5-inhibitors-PD5I, endothelin-receptor-antagonists-ERA). Direct costs (in Euro) were estimated multiplying data related to resource use by unit cost obtained from the accounting office of the Hospital.

**Results:** No differences were identified for mRSS, patient’s VAS for Raynaud and the number of digital pitting scars. Although without statistical significance, the mean number DUs increased in the day-care (0.61/patient for continuous vs 1.1/patient for intermittent). Fifteen patients stopped the intermittent scheme: 2 for logistics problem, 13 for AE (8 headache, 3 nausea, 2 fever), whereas no patients experienced AE during the continuous scheme. The mean number of cycles was 4.2 infusions/patients in continuous scheme and 5.6 in intermittent scheme (p=0.05). The number of patients treated with vasoactive drugs increased for PD5I (34% for continuous, 47% for intermittent; p<0.001) and ERA (21% continuous, 38% intermittent; p<0.001), whereas the prescription of CCB decreased (66.2% continuous vs 53.2% intermittent; p=0.01). Direct costs were slightly higher in continuous scheme (3132±883 Euros vs 2687±556 Euros) as compared to intermittent scheme, but without statistical significance.

**Conclusions:** Although the two schemes were not clearly different in term of efficacy, the continuous scheme was better tolerated, required a reduced number of infusions and concomitant vasoactive drugs, and the direct costs were not significantly different. The choice of the best Iloprost regimen should therefore be selected on the basis of the clinical features of the patients.

## P.276

## RAYNAUD’S PHENOMENON IN RHEUMATIC DISEASES: INCIDENCE AND CLINICAL MANIFESTATIONS

Z. Bagautdinova^1^, I. Gaisin^2^

^1^*Clinical Diagnostic Centre of the Udmurt Republic, Izhevsk, Russia*, ^2^*Izhevsk State Medical Academy, Izhevsk, Russia*

**Introduction:** To study the incidence and describe secondary Raynaud’s phenomenon (RP) in rheumatic diseases (RD).

**Material and Methods:** A questionnaire survey conducted in 230 patients with RD.

**Results:** RP was detected in 45.6% of RD patients (n=105), 54.4% of patients with RD had no RP (n=125). RP was 4 times more common in females with RD than in males (F:M 4:1). In RP group, there were mainly patients with autoimmune diseases (87, 82%): systemic sclerosis – SSc (55.2%), systemic lupus erythematosus – SLE (17.1%), rheumatoid arthritis – RA (6 %), dermatomyositis (3.8%), cross syndrome (3.8%), mixed connective tissue disease – mixed CTD (1.9%), Sjogren’s disease – SD (0.9%).

Only 84% of RP patients had a positive answer to all three questions that characterize RP (1. Is there an unusual sensitivity of fingers to cold? 2. Do fingers change color when exposed to cold? 3. Do they turn white and / or bluish?). Biphasic color changes (whitening-blueness; whitening-redness; blue-redness) were observed in 33 (31.4%) patients with RP, three-phase changes – in 32 patients (30.5%). Blueness of fingers to cold was more frequent in SLE than in SSc (p=0.027). Redness of fingers to cold occurred more often in cross syndrome, mixed CTD, SD, RA, vasculitis than in SSc (p<0.001) and in vasculitis than in SLE (p=0.035). In SSc patients, whitening of fingers to cold was more common than redness (p=0.037) and two- / three-phase changes of fingers color in the cold were more frequent than single-phase changes (p<0.001). The frequency of RP attacks was detected more than once a day in 44 (42%) patients. In 73% of cases, RP did not show signs of deep digital ischemia. Digital ulcers (active) were observed in 13 (12.3%) patients, fractures in the finger area – 23 (21.9%), digital scars – 15 (14.2%), phalange amputations – 7 (6.6%).

**Conclusions:** Patients with secondary RP in RD most often have SSc (55%), less often – SLE (17%), RA (6%), DM (3%). In SSc and SLE patients, Raynaud’s reddening of the fingers to cold is less common than in other RD. In SLE, fingers turn blue in the cold more often than in SSc.

## P.277

## RAYNAUD’S PHENOMENON IN RHEUMATIC DISEASES AND PROSTANOID THERAPY WITH THE USE OF THE GENERALIZED INDICATOR OF RAYNAUD’S PHENOMENON MANIFESTATION

Z. Bagautdinova^1^, L. Gaisin^2^, M. Giavatskikch^3^

^1^*Clinical Diagnostic Centre of the Udmurt Republic, Izhevsk, Russia*, ^2^*Izhevsk State Medical Academy, Izhevsk, Russia*, ^3^*Udmurt State University, Izhevsk, Russia*

**Introduction:** To determine the level of Raynaud’s phenomenon (RP) expression and to evaluate the long-term efficacy of iloprost and alprostadil in RP patients with rheumatic diseases (RD)

**Material and Methods:** Indicated therapy with prostanoids (intravenous iloprost, alprostadil or their combinations) was carried out for three years in 40 patients with secondary RP in RD. Frequency of Raynaud’s attacks, digital ulcers (DU) formation, pain intensity on visual analogue scale (VAS) were evaluated. A control group included 30 patients with RP in RD who did not receive prostanoid therapy. By factor analysis method an index of RP generalized expression was identified, on the basis of which levels of RP expression were determined

**Results:** “RP expression” scale, revealed as an indicator of RP generalized manifestation, was an average value of two subscales: (1) consisted of 4 indices “digital ulcer”, “digital pitting scars”, “phalange amputation” and “Raynaud’s attack frequency”, (2) included 3 indicators: “intensity of pain”, “duration of illness”, “whitening of fingers”. Correlation of subscales showed their reliability (r=0.294, p=0.053). RP final expression was 1.51±0.86. A low level of RP expression had values below 0.65, high – over 2.37. At baseline, the high level of RP severity was defined in 16 (22.9%) patients, medium – in 43 (61.4%), low – in 11 (15.7%). RP treatment with iloprost was effective in the healing of DU and led to decrease of RP generalized expression from 2.25 [1; 3] to 1.75 [1; 2] (p=0.012). On alprostadil therapy, there was a decrease of RP expression generalized index from 1 [1; 2] to 1 [0.5; 1.5] (p=0.038). Patients on prostanoids combination also had a significant decrease of RP generalized expression

**Conclusions:** Based on RP clinical manifestations, a generalized index of RP severity was identified and levels of RP severity were determined. Treatment with iloprost or alprostadil has significant effects on reducing of RP clinical manifestations with a corresponding decrease in its severity.

## P.278

## ASSOCIATION OF CAPILLAROSCOPIC ABNORMALITIES WITH THICKENING OF THE CAROTID BULB IN PATIENTS WITH SYSTEMIC SCLEROSIS: COMPARISON BETWEEN MICRO AND MACRO-CIRCULATORY CHANGES

C. Angelelli, I. Sciarra, M. Vasile, K. Stefanantoni, G. Pellegrino, G. Valesini, V. Riccieri

*Sapienza Università di Roma, Roma, Italy*

**Introduction:** Atherosclerosis (ATS) is characterized by endothelial damage of the large and medium calibre arteries due to lipid accumulation, cell death and fibrosis. Patients with Systemic Sclerosis (SSc) present other non-traditional cardiovascular risk factors such as inflammation, endothelial dysfunction, vasospasm; in fact, about 30% of premature deaths in SSc are caused by cardiovascular diseases. Nailfold videocapillaroscopy (NVC) is fundamental in monitoring the progression of microvascular disease and its abnormalities are included in the new ACR/EULAR classification criteria for SSc.

The aim of this study is to investigate the presence of both macrovascular and subclinical ATS in a group of SSc patients and to evaluate the possible associations with specific NVC abnormalities, expression of microcirculation damage

**Material and Methods:** We enrolled 42 SSc patients (M/F: 4/36) and 23 controls; all the subjects underwent a supra-aortic trunk echo-Doppler and the carotid intima-media thickness (IMT) was measured. Capillary density, specific capillaroscopy patterns (defined as early, active or late) and a score from 0 to 3 (considering length, density, distribution, morphology, dilation of the loops as well as the presence of avascular areas and haemorrhages) were analysed through NVC.

**Results:** Among the 42 patients, 24 (57%) presented abnormal IMT (>0.9 mm) of the carotid bulb, 15 (35%) of the common carotid artery, 2 (5%) of the internal carotid artery and 1 (2%) of the external carotid artery. The IMT of the internal, common and bulb carotid were more frequently increased in SSc patients than in controls (p=0.0001; p=0.01; p=0.0008 respectively). NVC score >1 was found in 33 (78%) patients and 39 (93%) presented a specific scleroderma NVC pattern. A significantly higher number of megacapillaries was present in SSc patients with IMT of the carotid bulb >9mm. compared to SSc patient without it (p = 0.02).

**Conclusions:** The macrovascular thickening of the arteries seems to be associated with the microvascular dilatation of the capillary loop, suggesting a relationship between endothelial dysfunction of micro-and macro-circulation in SSc patients.

## P.279

## HEMATOLOGICAL DISORDERS WITHIN SYSTEMIC SCLEROSIS: FREQUENCY AND CLINIC FEATURES

B. Doskaliuk, R. Yatsyshyn

*Ivano-Frankivsk National Medical University, Ivano-Frankivsk, Ukraine*

**Introduction:** Although initial signs of the Systemic sclerosis (SSc) are usually skin lesions, the real danger lies in a disruption of vital functions of the internal organs and systems. The aim of the study was to determine the prevalence of hematological disorders in patients with SSc and to conduct a comparative analysis between the clinical and laboratory parameters of the course of SSc and severity of anemia.

**Material and Methods:** The study included 43 patients, that met the SSc classification criteria ACR/EULAR 2013. The structure of the group was following women accounted for 95,3% (41/43), men – 4,7% (2/43); the age rate ranged from 39 to 70 years, on average – 57,5 ± 3,2 years; the duration of the disease was on average 13.7 ± 1.2 years. To all patients was conducted general clinical examination. Modified Rodnan Skin Score (mRSS) was used to assess skin lesions in the studied group of patients. The European Scleroderma Study Group (EScSG) was used to determine the severity of SSc, also was used Spielberger anxiety scale to discover the presence of anxiety disorders.

**Results:** During the study was found that among all patients in the experimental group, reticuloendothelial system issue was observed in 34,9% (15/43). The structure of hematological pathology in patients was as follows: anemia of different severity was found in 25.6% (11/43), lymphopenia - in 16.3% (7/43), leukopenia - in 9.3% (4/43) and only 2 patients had lymphocytosis. By determining of correlation between the SSc course peculiarities and severity of hematologic impairment, all indicators were characterized by the presence of direct connection. The correlation between severity of anemia and CRP, mRSS, ESR was r = 0,74; r = 0,63; and r = 0,69 respectively. During the self-assessment of their psychological status using the Spielberger scale, patients with anemia showed a higher level of anxiety than the individuals without this hematological syndrome.

**Conclusion:** Hematologic pathology is a common manifestation of an extra dermal lesions in SSc and directly correlates with the severity of the disease. That is why during the supervision of patients, special attention should be paid to the performance of the blood system. Correction of hematological disorders will improve the quality of life of patients and long-term prognosis of the disease.

## 8. Renal

## P.280

## CLINICAL FEATURES AND OUTCOME OF CHINESE PATIENTS WITH SCLERODERMA RENAL CRISIS: A CASE CONTROL STUDY

J. Zhou, D. Xu, Y. Hou, Q. Wang, M. Li, X. Zeng

*Peking Union Medical College Hospital-Department of Rheumatology, Beijing, China*

**Introduction:** To study the clinical characteristics, treatments and outcomes of Chinese Scleroderma renal crisis (SRC) patients from a single center.

**Material and Methods:** We retrospectively reviewed the clinical and laboratory data of 538 Systemic sclerosis (SSc) patients from January 2009 to December 2016 in our center, 337 patients contained all the data required for analysis, including 29 SRC patients and 308 SSc patients without SRC (non-SRC-SSc). The treatments of SRC patients were also retrospectively analyzed. Kaplan-Meier analysis was used to estimate survival of SRC patients.

**Results:** The prevalence of SRC is 5.4% in our cohort. The average age at the onset of SRC was 51.6±11.8 years, and the median period between SSc diagnosis and SRC onset was 1.2(0.0-19.6) years. Male patients (OR=3.298 (1.177-9.242), dcSSc (OR=2.869 (1.086-7.577), pericardial effusion (OR=9.490 (3.832-23.502), and Myocardial involvement (OR=7.242 (1.495-35.075) were associated with increased risk of developing SRC in SSc patients. All the 29 SRC patients suffered from hypertension and renal insufficiency during the onset of SRC. Nine patients had left heart failure, 5 patients had hypertensive encephalopathy, and 3 patients had hypertensive retinopathy. Eight patients had evidence of thrombotic microangiopathy. ACE inhibitors were prescribed to 27(93.1%) SRC patients. The permanent dialysis rate was 48.3%. The respective estimated 1- and 5- year survival rates of SRC patients were 62.1% and 47.3%. Patients had serum creatinine levels>500umol/L before SRC treatment (log rank test 5.051, P=0.025) had a poorer prognosis than those who did not, and patients who needed dialysis at the onset of SRC (log rank test 12.870, P=0.000) also had a poorer prognosis than who did not need.

**Conclusions:** SRC is a rare complication of SSc patients, which usually occurred at the early course of SSc. Male, dsSSc, pericardial effusion and myocardial involvement are risk factors of developing SRC ACEIs are the cornerstone in the treatment of SRC, but the prognosis is still poor.

## P.281

## SCLERODERMA RENAL CRISIS IN THE RESCLE COHORT. ASSESSMENT OF PREDICTOR FACTORS AND CHANGE IN PREVALENCE OVER TIME

X. Pla Salas^1^, C. Tolosa Vilella^2^, I. Pons Martín del Campo^1^, E. Callejas Moragas^2^, A. Guillén del Castillo^3^, J.A. Todolí Parra^4^, M. Rodríguez Carballeira^5^, A. Marín Ballvé^6^, I. Perales Fraile^7^, L. Sáez Comet^8^, A. Argibay^9^, A.B. Madroñero Vuelta^10^, M.E. Sánchez García^11^, C. González-Echávarri^12^, S. Sánchez Trigo^13^, J. Sánchez-Redondo^14^, R.Á. Fernández de la Puebla G.^15^, V. Fonollosa Pla^3^, C.P. Simeón Aznar^3^, On behalf of RESCLE Investigators^16^

^1^*Department of Internal Medicine. Xarxa Assistencial Universitaria de Manresa, Manresa, Barcelona, Spain*, ^2^*Department of Internal Medicine. Corporació Sanitaria Universitaria Parc Taulí, Sabadell, Barcelona, Spain*, ^3^*Unit of Autoimmune Diseases, Department of Internal Medicine. Hospital Universitario Vall d’Hebron, Barcelona, Spain*, ^4^*Department of Internal Medicine. Hospital Universitario y Politécnico La Fe, Valencia, Spain*, ^5^*Department of Internal Medicine. Hospital Universitario Mútua Terrassa, Terrassa, Barcelona, Spain*, ^6^*Unit of Autoimmune Diseases, Department of Internal Medicine. Hospital Clínico Universitario Lozano Blesa, Zaragoza, Spain*, ^7^*Department of Internal Medicine. Hospital Universitario Rey Juan Carlos, Móstoles, Madrid, Spain*, ^8^*Department of Internal Medicine. Hospital Universitario Miguel Servet, Zaragoza, Spain*, ^9^*Unit of Systemic Autoimmune Diseases and Thrombosis. Department of Internal Medicine. Complejo Hospitalario Universitari, Vigo, Pontevedra, Spain*, ^10^*Department of Internal Medicine. Hospital General San Jorge, Huesca, Spain*, ^11^*Department of Internal Medicine. Hospital Universitario Virgen de Valme, Sevilla, Spain*, ^12^*Autoimmune Diseases Research Unit. Biocruces Bizkaia Health Research Institute, Hospital Universitario Cruces, Barakaldo, Spain*, ^13^*Department of Internal Medicine. Complejo Hospitalario Universitario de Ferrol, Ferrol, A Coruña, Spain*, ^14^*Department of Internal Medicine. Hospital Universitario de Móstoles, Móstoles, Madrid, Spain*, ^15^*Department of Internal Medicine. Hospital Universitario Reina Sofía, Córdoba, Spain*, ^16^*Autoinmune Diseases Study Group (GEAS), Spain*

**Introduction:** Scleroderma Renal Crisis (SRC) is a serious complication of Systemic Sclerosis (SSc). Nowadays, it seems that there is a reduction in its prevalence and mortality.

Our objectives were to evaluate the characteristics of patients with SRC in a large cohort of SSc pacients, to investigate predictors of SRC, and to indetify an change in prevalence over time.

**Material and Methods:** 1933 patients were collected in ongoing registry of Spanish SSc pacients – RESCLE. A demographic description and epidemiologic analysis was performed.

**Results:** Out of 1933 SSc, 43 (2.2%) developed SRC. Univariate analysis showed significant differences of SRC vs. non-SRC cases: SSc subtypes: diffuse cutaneous SSc (dcSSc), 72% vs. 19%; limited cutaneous SSc (lcSSc), 26% vs. 61%. Demographics: Female gender, 77% vs. 89%; time from SSc onset to SSc diagnosis, 3.0±8.0 vs. 6.6±9.5 years. 1st manifestation: Raynaud’s phenomenon (RP), 68% vs. 82%; puffy hands, 9.5% vs. 2.3%. Clinical manifestations: RP, 89% vs. 96%; digital ulcers, 68% vs. 38%; arthritis, 45% vs. 20%; myositis, 30% vs. 13%; joint contractures, 45% vs. 18%; intestinal involvement, 24% vs. 11%; malabsorption, 24% vs. 7%; interstitial lung disease 57% vs. 41%; pericardial involvement, 34% vs. 8.7%; ischemic cardiopathy, 31% vs. 12%; LV diastolic dysfunction, 67% vs. 34%; sicca syndrome, 12% vs. 30%. Capillaroscopy: active pattern 74% vs. 33%. Immunological data: ATA, 38% vs. 20%; ACA, 15% vs. 49%; anti-RNApol III, 20% vs. 3.3%. Prognosis: Overall mortality, 57% vs. 18%; SSc-related mortality, 84% vs. 49%. Survival at 5, 10, and 20 years was 73% vs. 96%, 56% vs. 92% and 28% vs. 79%, respectively. Treatment: corticoid use, 50% vs. 20%; corticoid use less than 6 month before SRC, 36% vs. 0.8%, and prostaglandine use, 11% vs. 3.7%. Multivariate analysis: dsSSc subtype, OR 14.26 (3.88-52.38) p<0.001; time from SSc onset to SSc diagnosis, OR 0.86 (0.78- 0.94) p=0.001, prostaglandine use, OR 4.54 (1.50-13.76) p=0.008, corticoid use, OR 4.11 (1.65-10.27) p=0.002, and anti-RNApol III, OR 2.85 (1.03-7.89) p=0.044. Prevalence of SRC (P-SCR) in dcSSc subtype was 7.8%, and in lcSSc subtype 0.9%. P-SRC before and after 2003, 3.8% vs. 1.4%, p=0.031, respectively.

**Conclusions:** In RESCLE cohort, SRC predominated in dcSSc patients, was associated to a shorter period between SSc onset and SSc diagnosis as well as to prostaglandine and corticoid use, and to the presence of RNA pol III antibodies. SRC had a very poor prognosis. Finally, we evidenced a decreasing prevalence of SRC over time in our cohort.

**Figure:**
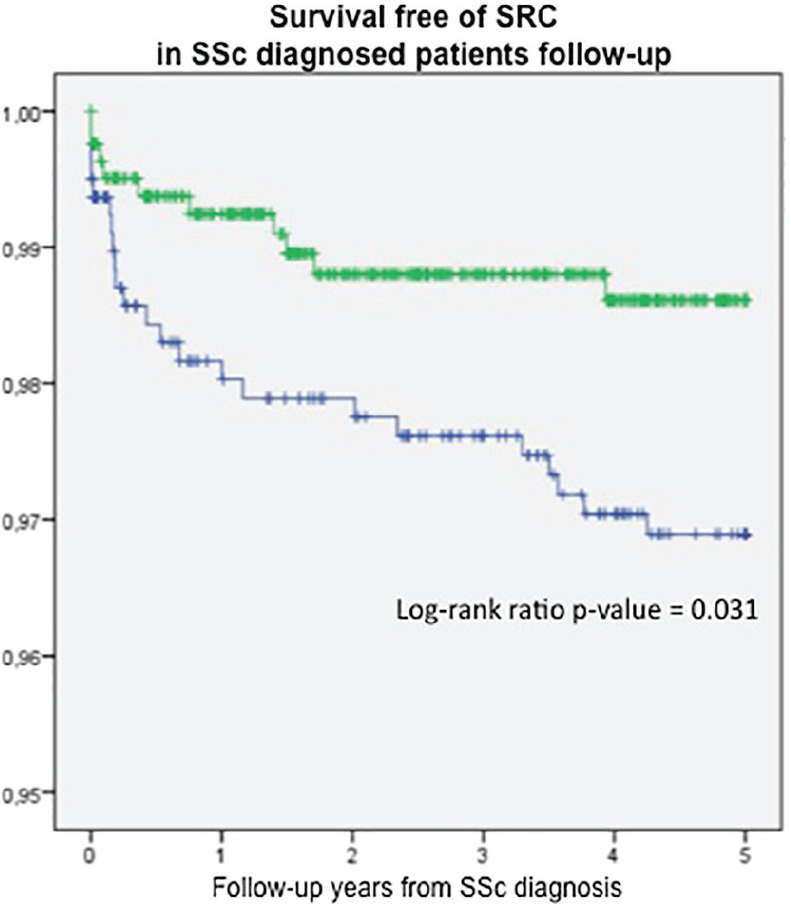


## P.282

## SCLERODERMA RENAL CRISIS OCCURS EARLY IN THE DISEASE COURSE: A CASE SERIES

M. Joubert^1^, L. Olagne^1^, P. Smets^1^, R. Outh^1^, O. Aumaitre^1^, C. Garrouste^2^, A. Lautrette^3^, M. Hermet^4^, M. Andre^1^

^1^*CHU Gabriel-Montpied, Clermont-Ferrand, France*, ^3^*Centre Jean-Perrin, Clermont-Ferrand, France*, ^4^*CH Jacques Lacarin, Vichy, France*

**Introduction:** Scleroderma Renal Crisis (SRC) is a severe manifestation of systemic sclerosis (SS), whose prognosis is bleak.

**Material and Methods:** We report a case series of 6 patients, from an internal medicine departement, in a hospital center, who had SRC between 2010 and 2019, according to criteria described by Steen in 2003. All patients met the ACR/EULAR criteria for SS. We collected risk factors, characteristics and treatment of SRC. The data were taken from the archiving computer files.

**Results:** There were 5 women and 1 man. Patients were aged from 38 to 72. Four patients had diffuse SS and 2 had limited SS. Two were positive for anti-RNA polymerase III antibodies, 2 for anti Scl-70 antibodies, 1 for anti-centromere antibody and 1 for anti-nucleole antibodies. Rodnan’s modified score at the time of the crisis was between 8 and 46.

A patient took 10 mg of Prednisone at the time of the crisis. No patient had used corticosteroid therapy greater than 15 milligrams per day in the 3 months prior to diagnosis. A patient took conversion enzyme inhibitors (ACE ), stopped shortly before SRC.

SRC occurred between 6 and 12 months following the first signs of SS outside Raynaud.

Systolic blood pressure ranged from 150 to 200 mmHg.

Creatininemia, at the time of SRC, was between 114 and 554 micromol/L. Four patients had proteinuria greater than 1.5 grams/grams of creatininuria. Four patients had thrombotic microangiopathy, 1 had encephalopathy, 1 intra-alveolar hemorrhage and 3 stage II or III hypertensive retinopathy. Two patients had a renal biopsy showing for one nephroangiosclerosis and the other ischemic glomerulonephritis.

Treatment with ACE was initiated in all patients and associated with calcium inhibitors for 4 patients. Four patients required extra-renal treatment, 3 in the acute phase of SRC and one, 11 months later. Of these 4 patients, 1 died of septic shock in acute phase of crisis, 2 required chronic hemodialysis and one was transplanted 22 months after the onset of SRC.

**Conclusions:** Our case series highlights some points: SRC occurs, most often, in the first year of SS and requires increased vigilance with self-measurement blood pressure and creatininemia monitoring. It applies to both limited and diffuse systemic scleroderma. Elevation of blood pressure, at the time of SRC, can be moderated. Although difficult to demonstrate in our survey, quick treatment with ACE is probably a major prognosis factor to avoid chronic dialysis or transplantation.

## P.283

## THE PATTERNS OF RENAL PATHOLOGY IN PATIENTS WITH SYSTEMIC SCLEROSIS RECEIVING RENAL BIOPSY: EXPERIENCE FROM A CHINESE TERTIARY MEDICAL CENTER

X. Cao, Y. Hou, D. Xu, M. Li, Q. Wang, X. Zeng

*Department of Rheumatology and Clinical Immunology, Peking Union Medical College Hospital, Chinese Academy of Medical Sc, Beijing, China*

**Introduction:** We sought to investigate the characteristics of renal injury in Chinese systemic sclerosis (SSc) patients who had undergone renal biopsy.

**Material and Methods:** By retrieving the medical records of SSc patients hospitalized at Peking Union Medical College Hospital between January 1990 and October 2017, 25 patients were identified. The clinical characteristics and pathological results were analyzed.

**Results:** We identified 10 patients with scleroderma renal crisis (SRC); 1 patient who underwent renal biopsy twice with diffuse mesangial proliferative glomerulonephritis and SRC, respectively; 2 patients with antineutrophil cytoplasmic antibody (ANCA)–associated glomerulonephritis (GN); 1 with immunoglobulin M nephropathy; 1 with minimal change nephropathy; 7 with lupus nephritis (LN); 1 with scleroderma renal crisis overlap LN; and 2 with drug-related kidney injury (caused by aristolochic acid and D-penicillamine, respectively). Acute tubular necrosis was observed in the patient taking aristolochic acid orally, while minimal change nephropathy was seen in D-penicillamine-induced renal injury.

**Conclusions:** SRC was the most commonly encountered renal damage in SSc patients. As the diagnosis of SSc entails exclusion of LN, ANCA-associated GN, and drug-induced renal injury, renal biopsy could serve as an essential tool for differential diagnosis. Rheumatologists in Eastern countries should be cautious of aristolochic acid nephropathy, especially in remote areas.

## P.284

## SENSORY-MOTOR NEUROPATHY AND RENAL IMPAIRMENT IN A SYSTEMIC SCLEROSIS PATIENT: RARE BUT NOT SO RARE?

CNS A. Schollum, K. Solanki

*Rheumatology Unit, Waikato Hospital, Hamilton, New Zealand*

**Introduction:** Rarely neurological symptoms herald in SSc and more rarely there occurs ANCA like vasculitis in SSc.

**Case:** We describe a lady in mid-fifty with stable SSc (Scl-70 3+) with raynaud’s, telangiectasia, stable NSIP-ILD presenting with increasing sensory-motor neuropathy.

She initially developed numbness of the left 4th and 5th toes then overnight also lateral aspect of her left leg and the ankle.

There was numbness of the dorsum of the left foot, reduced vibration sense on left foot and a reduced ankle jerk. The plantar flexion & extension was fine. Plantar reflexes were down going. There was no tenderness of spine and SLR was 75º.

Subsequently, she developed pins and needles sensation involving the 4th and 5th fingers and thereafter over the dorsal and palmar aspect. A day later she had increased numbness in her hands along with mild finger extension weakness. Her BP was normal.

There was no history of febrile illness, trauma, low back pain nor any worsening of raynaud’s or any other associated CTD symptoms. Cardio-respiratory point of view she was stable.

Investigations revealed pANCA (MPO type) raised at 49 IU/ml, with normal thyroid function, HbA1c, B12 and folate levels. Her urine protein creatinine ratio was 41.6 suggestive of significant proteinuria. MSU showed proteinuria with presence of white cell and hyaline casts and red cells suggestive of glomerular leak. Her CRP was elevated at 88mg/L and her serum creatinine was 72umol/L (normal).

Her nerve conduction study revealed axonal sensory and motor peripheral.

Renal biopsy showed features of focal segmental glomerulosclerosis with fibrosed crescents.

ECHO showed normal LV & RV function with mild diastolic dysfunction and normal RVSP and no significant abnormalities in valves

She was treated with pulse cyclophosphamide with prednisone (with close monitoring of BP), cotrimoxazole (for PJP prophylaxis) and Zoledronic acid infusion for bone health. This was followed by azathioprine for maintenance after 6 pulses of Cyclophosphamide.

Her neuropathic symptoms improved, with only numbness in digit 5 of the right hand and stocking distribution in the feet from the ankle downwards. Vibration at ankle level symmetrical 2+ reflexes in upper extremities, lower extremities knee reflex 2 bilateral, ankle reflex 1 bilateral, plantar reflex down bilateral. The patient could walk on her tiptoes.

**Conclusion:** We need to be aware of rare possibility of development of ANCA like vasculitis even in patients appearing to have stable SSc as a cause for neurological or renal manifestation like in our lady with microscopic polyarteritis.

## 9. Musculoskeletal System & Rehabilitation

## P.285

## PSEUDOTUMORAL CALCINOSIS IN SYSTEMIC SCLEROSIS

E. Zanatta^1^, M. Desportes^2^, D.H. Hoang^2^, J. Avouac^1^, F. Antoine^2^, Y. Allanore^1^

^1^*Rhumatologie A, Hôpital Cochin, Université Paris Descartes, Paris, France*, ^2^*Radiologie B, Hôpital Cochin, Université Paris Descartes, Paris, France*

**Introduction:** Although calcinosis is frequently reported in patients with systemic sclerosis (SSc), scarce data are available in the literature on their frequency, distribution, clinical association and outcomes. In particular, very little is known about the characteristics of large calcified masses in SSc.

**Material and Methods:** We performed a systematic literature review (SLR) of reported SSc-related large calcified masses in the PubMed database between January 1980 and March 2019. Subsequently, upon updating the terminology and definition, a systematic research of cases of “pseudotumoral calcinosis” was performed at Cochin and Padova University Hospitals.

**Results:** The SLR yielded 30 SSc cases with large calcified masses mainly defined as “tumoral” (14) or “pseudotumoral” (4). Among the 629 SSc patients in our cohorts, 19 (3%) living patients and 7 deceased patients were affected by pseudotumoral calcinosis. Within the whole population from literature and our cohorts (56 cases), mean age was 59 ± 11.4 yrs and 7/56 (12.5%) were men; the median disease duration at calcinosis onset was 7 (5-10) yrs. Twenty-six patients (46.4%) had the diffuse cutaneous form of SSc. Anti-topisomerase I and anticentromere antibodies were found equally, whereas one patient exhibited RNA-polymerase III positivity. The analysis of the phenotype showed substantial vascular damage in 80% of our patients. Pseudotumoral calcinosis were commonly symmetrical and ranged from 2 to 15.5 cm (Figure) with the largest one localized at hip-buttock level. Almost all patients (98%) had multiple site involvement: hand/wrist were the most frequent (54% of patients), then shoulders (29%), elbows (27%) and hips (20%). Thirteen patients (23%) were affected by spinal calcinosis, mostly at cervical level. Fistulization/ulceration and infections were reported in 34% and 23% of cases, respectively; nerve compression occurred in 40% of spinal calcinosis and in only one patient with limb calcinosis. There was no clear evidence of clinical and radiological improvement with any treatment. A partial improvement was observed in 7 patients that underwent surgery.

**Conclusions:** Pseudotumoral calcinosis may occur in about 3% of SSc patients, commonly symmetrical and in multiple sites without differences regarding the cutaneous subtype but often in patients with a severe vascular phenotype. Medical treatment seems ineffective, whereas a surgical approach may be considered. We propose that only calcinosis larger than 2-3 cm should be considered “pseudotumoral” in SSc, because of their clinical relevance.

**Figure:**
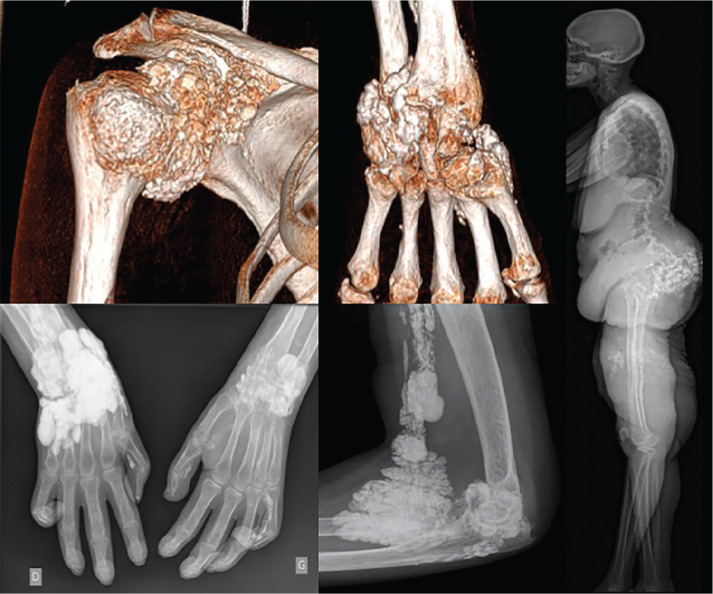


## P.286

## EFFICACY OF A TAILORED HAND/FACE REHABILITATION PROGRAM FOR SCLERODERMA PATIENTS: FINAL RESULTS OF A ONE-YEAR PROSPECTIVE CONTROLLED STUDY

M. Spiritovic^1^, H. Smucrova^2^, S. Oreska^3^, H. Storkanova^3^, B. Hermankova^1^, P. Cesak^1^, A. Rathouska^2^, O. Ruzickova^3^, K. Pavelka^3^, L. Senolt^3^, J. Vencovsky^3^, R. Becvar^3^, M. Tomcik^3^

^1^*Faculty of Physical Education and Sport, Department of Physiotherapy, Charles University, Prague, Czech Republic*, ^2^*Institute of Rheumatology, Prague, Czech Republic*, ^3^*Institute of Rheumatology, Department of Rheumatology, 1st Faculty of Medicine, Charles University, Prague, Czech Republic*

**Introduction:** Involvement of the skin, muscles and joints due to fibrosis in scleroderma (SSc) patients leads to a decrease in their quality of life and functional ability. Rehabilitation can help improve this condition. The aim of our study was to analyze the effect of an intensive tailored hand/face rehabilitation program.

**Material and Methods:** Patients with definite SSc (n=55) according to EULAR/ACR criteria (2013) and hand/mouth(face) involvement were included to our study. Based on their ability to participate in the rehabilitation program, 25 patients were recruited into the intervention group (IG) and 30 into the control group (CG). At baseline, both groups received an educational material for home exercise, but only the IG underwent a 6-month intervention with a subsequent 6-month follow-up period. Patients were assessed by a physician and a physiotherapist blinded to intervention at months 0, 3, 6, and 12. Patients also filled out patient reported outcomes questionnaires and provided blood for routine laboratory analysis and bio-banking. Data analysis was done between groups and within the group, and subsequently adjusted for statistically significantly different baseline clinical parameters between IG and CG (mRSS, disease duration and ESR).

**Results:** In the IG, we demonstrated statistically significant improvement in objectively assessed function and strength of hand, distance between incisors and lips and subjectively assessed hand function and functional ability, compared to the observed statistically significant deterioration in the CG over month 0-6 (all p-values remained < 0.05 after adjusting for mRSS, disease duration and ESR). Only numerical improvements in the IG during the intervention compared to numerical deterioration in CG, that did not reach statistical significance, were observed in subjectively assessed mouth handicap, functional ability and in some domains of QoL and fatigue - in cognitive function. During the follow-up period, there was a significant deterioration or stagnation of the achieved positive results in the IG.

**Conclusions:** Our tailored hand/face rehabilitation program led to a significant improvement in the observed parameters that was clinically significant in a substantial proportion of patients, and to prevention of the expected worsening of hand/face handicap and patients’ quality of life.

**Acknowledgement:** Supported by AZV-16-33574A, MHCR 023728, SVV for FTVS UK 2019-260466.

## P.287

## CLINICAL CHARACTERISTICS IN PATIENTS WITH SYSTEMIC SCLEROSIS AND SYMPTOMATIC SUBCUTANEOUS CALCINOSIS

Y. Shirai, M. Kuwana

*Nippon Medical School Graduate School of Medicine, Tokyo, Japan*

**Introduction:** Subcutaneous calcinosis is a disabling complication in patients with systemic sclerosis (SSc) and can cause inflammation, skin ulceration, and severe pain, leading to impairment of activity of daily living and quality of life. Its clinical pathogenic process has not been well characterized. We herein investigated clinical characteristics of SSc patients with symptomatic subcutaneous calcinosis.

**Material and Methods:** This single-center, cross-sectional study enrolled 260 SSc patients. Demographic, clinical characteristics, laboratory data, and treatment regimens were retrospectively collected by medical chart review. In a case-control study, clinical features were compared between patients with and without calcinosis (1:2), in whom sex, disease subset, age at onset, and SSc-related antibodies were matched.

**Results:** Sixteen patients (6%) had symptomatic subcutaneous calcinosis: including 7 with diffuse cutaneous SSc (dcSSc) and anti-topoisomerase I antibody and 8 with limited cutaneous SSc (lcSSc) and anticentromere antibody. Calcinosis was detected in fingers (n = 11), trunk (n = 5), hands/feet (n = 5), extremities (n = 3), and face (n = 1). Distribution of calcinosis was different between dcSSc and lcSSc patients (P = 0.01): more extensive distribution involving multiple sites versus fingers alone. Calcinosis was diagnosed at 15 ± 9 years from the first non-Raynaud’s symptom. When compared with matched controls, patients with calcinosis showed higher prevalence of history of digital ulcers (P = 0.026). On hand X-ray, acroosteolytis was more frequently observed in patients with calcinosis than in those without (P = 0.005). There was no difference of laboratory data, including serum levels of calcium, phosphate, intact parathormone. In terms of treatment before the diagnosis of calcinosis, patients with calcinosis were more frequently treated with beraprost (oral prostanoid), corticosteroids, and bisphosphonate (P = 0.009, 0.02, and 0.04, respectively), but were less frequently treated with calcium channel blockers (P = 0.001).

**Conclusions:** Unlike previous reports addressing predominance of subcutaneous calcinosis in patients with lcSSc, a subset of dcSSc patients had more extensive calcinosis. Calcinosis may be associated with underlying severe peripheral vascular disease, rather than calcium metabolism.

## P.288

## ASSESSING SKELETAL MUSCLE MASS WITH ULTRASOUND IN PATIENTS WITH SYSTEMIC SCLEROSIS

A. Akdogan^1^, A. Sari^1^, M. Esme^2^, L. Kilic^1^, G. Aycicek^2^, M. Halil^2^

^1^*Hacettepe University Medical School, Department of Rheumatology, Ankara, Turkey*, ^2^*Hacettepe University Medical School, Department of Geriatrics, Ankara, Turkey*

**Introduction:** Patients with systemic sclerosis (SSc) have increased risk for decreased skeletal muscle mass due to inflammation, malnutrition, low physical activity status and musculoskeletal involvement. The role of ultrasonography (USG), which is increasingly used in rheumatology practice, in evaluating muscle mass in this patient group is unknown. The aim of this study was to assess of the utility of USG in the evaluation of muscle mass in SSc.

**Material and Methods:** 93 SSc patients who were admitted to rheumatology outpatient clinic were included. Appendicular skeletal muscle mass indices (ASMI) of patients was calculated using bioelectric impedance analysis (BIA). Decreased muscle mass was defined as an ASMI below 7.26 kg / m2 for men and 5.50 kg / m2 for women. The severity of the gastrointestinal involvement was assessed by the UCLA GIT 2.0 scale and the physical activity status by the International Physical Activity Questionnaire (IPAQ). The presence of malnutrition was determined ESPEN criteria was used to determine the presence of malnutrition. Thicknesses of gastrocnemius, rectus femoris, rectus abdominis, external oblique, internal oblique and transverse abdominis muscles was assessed by USG. Pearson analysis was used to evaluate correlation between ASMI and each muscle thickness. Sensitivity, specificity and predictive values of ultrasonographic cut-off values in predicting decreased muscle mass were determined by ROC analysis.

**Results:** Reduced muscle mass was present in 13 (13.9%) of 93 patients (F/M: 86/7). Diffuse subset (53% vs 17%), antitopoisomerase-1 antibody positivty (76% vs. 47%) and malnutrition (61% vs. 8%) were more frequently observed in patients with decreased muscle mass (p<0,05 for all). Ultrasonographic thickness (cm) of gastrocnemius (1.51 vs. 1.23), rectus abdominis (0.74 vs.0.54) and transversus abdominis (0.34 vs. 0.30) muscles were also significantly low in this group. Gastroknemius thickness showed the highest correlation with ASMI (r = 0.513, p <0.001). The cut-off value of 1.47 cm for the thickness of gastrocnemius had 92.3% sensitivity, 58.7% specificity and 97.9% negative predictive value in predicting decreased muscle mass (AUC: 0.846).

**Conclusions:** Measurement of gastrocnemius thickness by USG can be used as screening test due to its high sensitivity and negative predictive value for detecting decreased muscle mass in SSc patients.

## P.289

## CLINICAL, PARACLINICAL AND RADIOLOGICAL ASSESSMENT OF THE MUSCULOSKELETAL MANIFESTATIONS IN SYSTEMIC SCLEROSIS

H. Poormoghim^1^, N. Azarbani^1^, A. Javadzadeh^1^, E. Andalib^1^, I. Mohseni^1^, A. Jalali^2^

^1^ *Firoozgar hospital.Iran University of Medical Sciences, Tehran, Iran*

^2^Department of Epidemiology and Biostatics, Tehran University of Medical Sciences, Tehran, Iran

**Introduction:** Systemic sclerosis (SSc) is a chronic connective tissue disease with clinical hallmark of skin thickening and tethering. However; musculoskeletal (MSK) symptoms is frequent and should be considered in SSc patients.

**Material and Methods:** We reviewed the records of all patients who had more than one visit and standard hand anteroposterior radiography for clinical MSK manifestation of SSc. Radiographical assessment conducted by two independent radiologists. In case of disagreement the problem was resolved by second evaluation. We used univariate analysis and actors with (p<0.05) were included in linear logistic regression to find out dependent factors.

**Results:** Study population: Total of 180 SSc patients enrolled in our study;161(89.4%) were women. Fifty-one (28.3%) patients were older than 50 y/o. Ninety-five (52%) had a diffuse subtype of the disease. Arthritis or arthralgia, as first presenting symptoms, were recorded in 6.7 % of patients as the first symptom. We found arthritis > 1 joint on physical examination in 35 (19.4%) patients, without significant difference between two subtypes. Joint contractures on physical exam were present in 96 (55.2%) patients and more frequent in dcSSc subtype. Muscle weakness was found in 51(28.3%) patients without significant differences in two groups of patients, of those 66.7% had elevated CPK., p=0.001, and 62.5% of patients with muscle weakness had high aldolase, p=0.07. Concordance of elevated both Aldolase and CPK demonstrated in 63.2% of patients with muscle weakness on physical examination. Radiographic pattern in two subtypes Subgroup analysis showed patients with dcSSC had more acro-osteolysis and flexion contractures; p=0.007 and p=0.05, respectively. Association of radiography with clinical, paraclinical features In multivariate analysis, the results indicate that disease duration correlated with joint erosion, acro-osteolysis, resorption of distal ulna and calcinosis, radiological flexion contracture (FC), p<0.05. Bone demineralization was associated with acro-osteolysis (P=0.001), and acro-osteolysis was more frequent in dcSSc subtype, TFRs, and anti-TOPO I antibody. Radiologic FC also showed association with modified Rodnan skin score, calcinosis on physical examination and hematocrit <30%.

**Conclusions:** The study highlighted musculoskeletal involvement in systemic sclerosis. One-fifth of study subjects had arthritis on physical examination. Muscle tenderness and weakness were prevalent comparable symptoms. CPK elevation accompanied with muscle weakness and can be used as a diagnostic tool. Aldolase did not increase sensitivity of the test for diagnosis. Acro-osteolysis presented in severe disease form. Correlation of disease duration with radiologic findings as an interdependent factor could suggest a cumulative effect of time on developing the lesion.

## P.290

## PREVALENCE OF OSTEOPOROSIS IN SYSTEMIC SCLEROSIS - A POPULATION BASED STUDY

K. Lillpers^1,2^, I. Bartosik^1,2^, D.M. Wuttge^1,2^, K. Andréasson^1,2^, K. Åkesson^2,3^, R. Hesselstrand^1,2^

^1^*Skåne University Hospital - Department of Rheumatology, Lund, Sweden*, ^2^*Lund University - Department of Clinical Sciences, Lund, Sweden*, ^3^*Skåne University Hospital - Department of Orthopedics, Malmö, Sweden*

**Introduction:** Systemic sclerosis (SSc) is a rare autoimmune, vascular and fibrotic disease. Osteoporosis on the other hand is frequent, especially among postmenopausal women. Inflammation is a risk factor for osteoporosis. Hypothetically, it is therefore reasonable to assume a correlation between SSc and osteoporosis. However, data is limited and conflicting.

The aim of this study was to investigate the prevalence of osteoporosis and osteopenia in consecutive SSc patients and to identify related risk factors specific to SSc.

**Material and Methods:** This cross-sectional study includes 211 consecutive patients, 182 (86.3 %) women and 29 (13.7 %) men, with SSc from Region Skåne in southernmost Sweden. Bone mineral density (BMD) was measured using Dual-energy X-ray absorptiometry at the hip and spine. Low BMD includes osteopenia, T-score below -1.0, and osteoporosis below -2.5. Blood samples were taken from all patients at baseline including bone metabolism parameters. Modified Rodnan Skin Score (mRSS), pitting scars, finger ulcers and telangiectasia were assessed. Disease subtype, antibodies, vital capacity, calcinosis at diagnosis and disease duration were collected from medical records. For statistical analysis SPSS was used.

**Results:** Almost 2/3 of all patients had low BMD. Mean T-score spine (±SD) was -1.0 (±1.4) and hip -1.2 (±1.1). Forty-one patients had T-score < -2.5 and 101 had a T-score between -1.0 and -2.5 which corresponded to 19.4 % with osteoporosis and 47.9 % with osteopenia. Among men only one had osteoporosis whereas 55.2 % (n=16) had osteopenia. There were no difference in BMD between patients with limited cutaneous SSc and diffuse cutaneous SSc, and no association between mRSS and BMD. Patients with finger ulcers (n=18) had a mean T-score -1.1 (±1.2) spine and -1.7 (±0.98) hip, with a prevalence of osteoporosis of 33% (n=6). Presence of finger ulcers was associated with T-score at the hip (R=-0.15, P< 0.025).

**Conclusions:** Low BMD, osteopenia and osteoporosis, are prevalent in SSc. Possibly those patients with finger ulcers are at higher risk. The study highlights the importance of screening for osteoporosis in SSc patients.

## P.291

## PHYSICAL THERAPY IN SYSTEMIC SCLEROSIS: PERSPECTIVES FROM PATIENTS AND THEIR PHYSIOTHERAPISTS

S. Liem^1^, N. Van Leeuwen^1^, E. Van Den Ende^2,3^, L. De Pundert^4^, R. Schriemer^3,5^, J. Spierings^6^, M. Vonk^3^, T. Vliet Vlieland^7^, J. de Vries-Bouwstra^1^

^1^*Leiden University Medical Center, Department of Rheumatology, Leiden, The Netherlands*, ^2^*Sint Maartenskliniek, Department of Rheumatology, Nijmegen, The Netherlands*, ^3^*Radboud University Medical Center, Department of Rheumatology, Nijmegen, The Netherlands*, ^4^*Haga Ziekenhuis, Department of Physical Therapy, The Hague, The Netherlands*, ^5^*NVLE, Dutch Patient Organization for Systemic Autoimmune Diseases, Utrecht, The Netherlands*, ^6^*University Medical Center Utrecht, Department of Rheumatology, Utrecht, The Netherlands*, ^7^*Leiden University Medical Center, Department of Orthopedics, Rehabilitation Medicine and Physical Therapy, Leiden, The Netherlands*

**Introduction:** The importance of non-pharmacologic interventions in systemic sclerosis (SSc) is increasingly recognized. Physiotherapists are the most visited non-physician health professionals in SSc, but little is known about the actual content of the provided physiotherapy (PT) and patients’ and physiotherapists’ perspectives on its present and future delivery. Therefore, this study aimed to assess the utilization, contents, needs and preferences regarding PT firstly from the perspective of SSc patients and secondly of their caring physiotherapists.

**Material and Methods:** Four hundred and five SSc patients, fulfilling the ACR 2013 criteria and participating in SSc cohort at Leiden University Medical Center, received a 44-item questionnaire about utilization, contents, needs and preferences regarding PT between July-August 2019. Patients who had visited a physiotherapist were asked to deliver the second questionnaire regarding bottlenecks, needs and preferences for education to their physiotherapist. Non-parametric tests were used to compare PT-users and non-PT-users.

**Results:** In September 2019, 185 (46%) SSc patients (median age:63 years, 79% female) were included. In the past two years, 113 (61%) patients received PT, of whom 56 (53%) because of SSc (PT-SSc), while 72 (39%) had never received PT (never-PT). Disease characteristics were comparable between groups. PT-users most often received muscle strengthening (79%), range of motion (74%) and balance exercises (55%; Figure 1). Regarding SSc-specific exercises, 8% received mouth and 23% hand exercises. Passive treatments were relatively frequent (massage:49%). Additionally, patients preferred to receive more information concerning PT and to continue or (re)start PT in respectively 51% (PT-SSc:68%, never-PT:46%), and 66% (PT-SSc:92%, never-PT:43%). Secondly, 36 physiotherapists completed the questionnaire (median age:49 years, 56% male, 58% with working experience >20 years). The median number of SSc patients currently treated was 1 (range:1-3). Muscle strengthening (91%), range of motion (88%) and balance (77%) exercises were often reported (Figure 1), followed by hand (44%) and oral (11%) exercises. Seventy percent of physiotherapists stated a lack of medical information concerning patients’ disease manifestations in the referral letter, and 78% expressed need for additional courses on SSc, favoring online modality (50%).

**Conclusions:** Forty-six percent of SSc patients received PT in the last two years. Most patients received active therapy, however massage was employed in half of them. In a group of patients annually seen by a team of health professionals including PTs, more than half expressed an unmet need regarding use of PT. These results could be a starting point to improve quality, comprehensiveness, and multidisciplinarity of care for patients with SSc.

**Figure:**
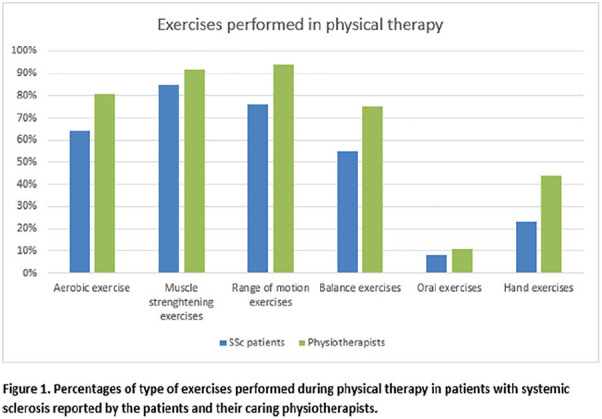


## P.292

## BONE MINERAL DENSITY AND TRABECULAR BONE SCORE ASSESSMENT IN SYSTEMIC SCLEROSIS: A CROSS-SECTIONAL STUDY

A. Lescoat^1^, M. Leroy^2^, G. Coiffier^2^, C. Cazalets^1^, N. Belhomme^1^, A. Ballerie^1^, F. Robin^2^, P. Jego^1^, P. Guggenbuhl^2^

^1^*CHU Rennes, Internal medicine and clinical immunology, Rennes, France*, ^2^*CHU Rennes, Rheumatology, Rennes, France*

**Introduction:** The impact of Systemic sclerosis (SSc) on bone metabolism is still to be further explored, especially in patients fulfilling the more recent ACR/EULAR 2013 classification criteria for SSc. A higher risk of fracture has been documented in SSc patients, but the underlying mechanisms are still debated. In previous studies, mainly based on the 1980 classification criteria or on 2001 Leroy’s criteria, SSc patients had a lower Bone Mineral Density (BMD) compared to healthy controls. The objective of this study was to conduct a complete and non-invasive global bone evaluation (including simultaneous assessment of Bone Mineral Density (BMD), Trabecular bone score (TBS), vertebral fracture assessment (VFA) and FRAX) in a large population of SSc patients fulfilling the 2013 ACR/EULAR classification criteria for SSc, in order to assess the relevance of these parameters in SSc and their association with the main clinical manifestations of the disease.

**Material and Methods:** 116 SSc patients were included in this cross-sectional study. BMD was evaluated and associated with TBS and VFA. FRAX score and TBS-FRAX score were also calculated. Usual clinical characteristics of SSc patients were evaluated at the time of BMD assessment according to standard guidelines

**Results:** 28.4% of the patients had osteoporosis (defined as a T-score under or equal to -2.5 and/or the history of major osteoporotic fractures). Among them, 8.6% had minor or major osteoporotic fractures, and 7 patients had vertebral fractures, with a total count of 22 vertebral fractures. The prevalence of major osteoporotic fractures was 7.8%. VFA allowed the diagnosis of 5 of these 22 fractures (22.7%) and these 5 fractures were all newly diagnosed through VFA. Femoral neck BMD, lumbar spine BMD, and total hip BMD were significantly correlated with TBS. Patients with longer disease duration (>5 years) had significantly lower TBS value (1.284 +0.087 vs 1.343+0.106, p<0.05). FRAX scores in our population did not result in any new indication for starting anti-osteoporotic drugs considering the risk of major fractures.

**Conclusions:** This study highlights that almost one-third of SSc patients fulfilling the ACR/EULAR 2013 classification criteria has osteoporosis. VFA can help to the diagnosis of vertebral fractures in SSc. Considering the low prevalence of patients with steroids in our population TBS did not provide new information regarding the risk of fracture.

## P.293

## THE EFFICACY OF A CONCISE SELF-MANAGEMENT PROGRAM FOR HANDS IN SSC – AN OBSERVATIONAL CASE-CONTROL STUDY

S. Landim^1^, M. De Barros Bértolo^1^, J.L. Poole^2^, A.P. Del Rio^1^, E. De Paiva Magalhães^1^

^1^*Department of Medical Clinic School of Medical Sciences, University of Campinas - UNICAMP, Campinas - SP, Brazil*, ^2^*Department of Pediatrics, Occupational Therapy Graduate Program, School of Medicine, University of New Mexico, Albuquerque-NM, USA*

**Introduction:** Hands are commonly affected in Systemic Sclerosis (SSc). Rehabilitation is recommended from time of diagnosis and throughout the treatment. Self-management programs are well-known adjuvant instruments to instruct patients to deal with chronic conditions. The objective of this research was to evaluate the effect of a concise self-management care program for hands in SSc.

**Material and Methods:** Patients were approached during their regular appointments in our outpatient clinic. They were accessed regarding clinical data, pain-VAS (Visual Analog Scale), hand function (Cochin Hand Function Scale/CHFS), range of motion/ROM (Delta Finger-to-palm/d-FTP), grip, tip and key strength, disability (Health Assessment Questionnaire/ HAQ and Scleroderma Health Assessment Questionnaire/SHAQ), quality of life (SF-36), moisturizing and warming habits. A concise home based self-management program was offered to each patient. Those who were interested in enrolling the program were included in the Intervention Group (IG) and those who could not return for regular evaluations were included in a Control Group (CG) with no intervention. The intervention was a booklet on self-management with 10 hand exercises mostly stretching and 12 items regarding some of the mainly aspects of SSc, education on hand care and disease management. Outcomes were assessed at initial visit (t0) and after 2 (t1), 8 (t2) and 24 (t3) weeks in the IG and in t0 and t3 in the CG.

**Results:** A total of 90 patients were assessed. 47 entered the IG and 36 in the CG. 26 patients (7 from IG and 19 from CG) were excluded because they did not complete the final evaluations. Data from 57 subjects (40 in IG and 17 in the CG were used in final analysis. Significant improvements were observed in the IG for pain-VAS, CHFS, SHAQ, d-FTP, hand strength and SF-36 (emotional, social and mental health domains), warming and moisturizing habits. Patients from the CG did not improve or even worsened.

**Conclusions:** This concise home-based self-management program for hands in SSc resulted in decreased pain and disability and improved joint motion, function and quality of life. Results may be related to exercises as well as to a better attention given to hand care, especially to warming and moisturizing habits.

## P.294

## MUSCULOSKELETAL MANIFESTATIONS OF SYSTEMIC SCLEROSIS: FOCUSING ON RIB OSTEOLYSIS

J.-B. Jun^1^, J.A. Ryu^2^, T.-H. Kim^1^, S. Lee^3^

^1^*Hanyang University Hospital for Rheumatic Diseases, Seoul, South Korea*, ^2^*Hanyang University Guri Hospital, Guri-si, South Korea*

**Introduction:** Systemic sclerosis (SSc) is a multisystemic disease characterized by vasculopathy, autoimmunity, and fibrosis. Although the musculoskeletal manifestations of SSc are well characterized and sporadic cases on rib osteolysis have been reported, there is no well-designed study demonstrating the frequency and its clinical relevance of rib osteolysis in SSc.

**Material and Methods:** Chest X-ray and CT were reviewed by an experienced musculoskeletal radiologist for 690 patients who visited Hanyang University Hospital for Rheumatic Diseases more than once with SSc from January 2005 to March 2019. Clinical information was collected through a chart review.

**Results:** Among 690 patients, 472 (female 416, male 56) patients were recruited and analyzed, except 218 who did not have chest radiographs. Rib osteolysis existed in 48 patients, showing a prevalence of 10.2%. All were asymptomatic and no further evaluation were performed with regard to rib osteolysis. SSc patients with rib osteolysis were younger and more likely being women than SSc patients without rib osteolysis. The rib osteolysis was mainly occurred in the third, fourth, fifth, sixth, and seventh ribs, with about half being two-sided and multiple rib involvement at 40%.

**Conclusions:** Rib osteolysis is much more common in patients with SSc than we know. Long-term and prospective observations should reveal the clinical significance of rib osteolysis in patients with SSc.

## P.295

## REDUCED CIRCULATING LEVELS OF INORGANIC PYROPHOSPHATE ARE ASSOCIATED WITH ECTOPIC CALCIFICATION IN SCLERODERMA SPECTRUM DISORDERS

V. Hsu^1^, N. Schlesinger^1^, Q. Li^2^, J. Varga^3^

^1^*Rutgers- Robert Wood Johnson Medical School, New Brunswick, USA*, ^2^*Thomas Jefferson University, Philadelphia, USA*, ^3^*Northwestern University, Chicago, USA*

**Introduction:** Calcinosis cutis due to ectopic mineralization is a common and disabling complication of systemic sclerosis (SSc) that has poorly understood pathogenesis and no effective treatment. Inorganic pyrophosphate (PPi) is a key regulator of ectopic mineralization acting by inhibiting hydroxyapatite crystal growth. Ectopic mineralization has been attributed to low PPi levels in several genetic disorders, such as pseudoxanthoma elasticum, generalized arterial calcification of infancy, and arterial calcification due to CD73 deficiency. Herein, we sought to test the hypothesis that reduced plasma PPi levels may be associated with SSc and play a pathogenic role in calcinosis.

**Material and Methods:** Subjects meeting 2013 ACR criteria for SSc and age-matched controls without SSc were recruited from Rheumatology outpatient clinics for this IRB-approved study. Calcinosis was confirmed either by clinical criteria or by imaging. Levels of PPi in platelet-free plasma were measured by an enzymatic reaction using 14C-labeled uridine-diphosphoglucose as substrate. Student’s T-test was used to compare mean PPi levels between normal and SSc groups and linear regression to compare means between three groups (normal, SSc –calcinosis and SSc-no calcinosis) with adjustment of potential confounders.

**Results:** Thirty three subjects were studied (19 control and 14 with SSc, 5 (36%) of whom were SSc- calcinosis. In the SSc cohort, 60% had limited cutaneous SSc, as did most (4 of 5) of the SSc-calcinosis group. The disease duration from non-Raynaud symptoms was 18.7 (8.7) years in SSc- calcinosis and 10.3 (7.4) years in SSc- no calcinosis.

Figure 1 shows that SSc cohort had significantly lower plasma levels of PPi (mean= 2.25 vs 3.04, p= 0.0083) compared to control group (see Table 1, p for group difference =0.0137). After adjusting for sex, the highest mean PPi levels were seen in the control group and the lowest in SSc-calcinosis group.

**Conclusions:** Our findings indicate that PPi levels are significantly reduced in SSc patients with calcinosis. The results suggest that PPi deficiency may be due to genetic or environmental influences that may be important in the pathophysiology of calcinosis, and could serve as a biomarker of the ectopic mineralization process. Larger clinical studies to confirm our findings are warranted.

**Figure:**
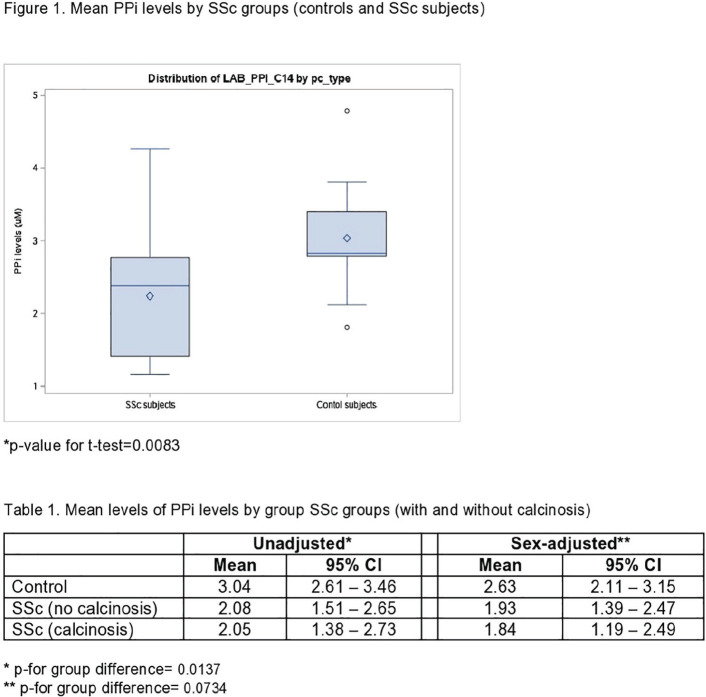


## P.296

## EFFECTS OF A HOME-BASED PULMONARY REHABILITATION PROGRAM, IN PATIENTS WITH INTERSTITIAL LUNG DISEASE RELATED TO SYSTEMIC SCLEROSIS: A PILOT STUDY

S. Faverzani^1^, A. Ariani^1^, A. Becciolini^1^, F. Nocera^2^, F. Mozzani^1^, D. Santilli^1^, L. Monica^1^, L. Barone^1^, V. Alfieri^1^, E. Crisafulli^3^, M. Marvisi^4^, M. Bigliardi^5^, M. Tommasi^5^, C. Micheli^5^, L. Montinaro^5^, G. Scopelliti^6^, N. Salati^2^

^1^*University Hospital of Parma, Parma, Italy*, ^2^*Private Practice, Parma, Italy*, ^3^*University of Verona and Azienda Ospedaliera Universitaria Integrata of Verona, Verona, Italy*, ^4^*Casa di Cura Figlie di San Camillo, Cremona, Italy*, ^5^*University of Parma, Parma, Italy*, ^6^*Cystic Fibrosis Unit Hospital of Ancona, Ancona, Italy*

**Introduction:** Pulmonary Rehabilitation (PR) is an ‘evidence-based, multidisciplinary and comprehensive intervention for symptomatic patients with chronic respiratory diseases with daily life activities impairment’. ¹ The American Thoracic Society and European Respiratory Society consensus report supports PR in patients affected by Interstitial Lung Disease (ILD).² ILD is common in Systemic Sclerosis (SSc), but so far, no studies have investigated the PR impact in this population. Therefore, the aim of this study is to evaluate the effect of PR in SSc-ILD.

**Material and Methods:** Patients with SSc-ILD were enrolled for a home-based PR program. This included 8 weeks of treatment, with 5 weekly sessions: 3 Interval Training sessions with a stepper and 2 sessions to improve upper limbs strength with weights and resistance bands. A Respiratory Physiotherapist trained patients to use the Borg Scale (for dyspnea and muscle fatigue), to increase their weekly workload gradually, and taught the patients exercises for the upper limbs. Patients were not supervised during the 8 weeks, but they were re-evaluated at the end of the program.

Field tests (six minutes step test and 30 second sit to stand chair test) and patients reported outcomes (Saint George Respiratory Questionnaire [SGRQ], Visual Analogue scale for disease activity and modified Medical Resource Council) were carried out at the beginning and at the end of the 8 weeks program. Wilcoxon test for paired data evaluated the differences before and after the 8 weeks of PR. p < 0.05 was considered statistically significant.

**Results:** Seventeen patients with SSc-ILD diagnosed (16 F; mean age 62; 13 ANA positive) were identified. The majority of patients (11 out of 17) admitted they have not always performed the training as planned. The ‘symptom selection’ of the SGRQ showed a statistically significant improvement (from 29 to 21, p=0.0063), while all the other data showed no significant changes.

**Conclusions:** Surely the lack of supervision by health professionals has influenced the adherence to the program. Despite the lack of compliance, the patients have achieved the main objectives of the PR, that is the improvement of the symptoms dyspnea and muscular fatigue. This suggests that even a minimum increase in physical activity and an education in lifestyle changes may lead to a significant increase in patients quality of life.

**References**

1. Nici L, Zuwallack R. Eur J Phys Rehabil Med 2011;47:465–74.

2. Raghu G, et al. Am J Respir Crit Care Med 2011;183:788–824.

## P.297

## IF YOU DON’T USE IT, YOU LOSE IT’: REHABILITATION OF FINGER DEXTERITY AND ABILITY TO PERFORM ACTIVITIES OF DAILY LIVING IN SYSTEMIC SCLEROSIS

E. Eusterwiemann^1^, M. Anderson^2^, M. Robinson^1^, G.J. Barton^1^

^1^*Liverpool John Moores University, Liverpool, United Kingdom*, ^2^*Aintree University Hospital, Liverpool, United Kingdom*

**Introduction:** Hand involvement due to increased skin thickness and skin collagen content is one of the first manifestations of Systemic Sclerosis (SSc). Reduced joint mobility and the development of flexion contractures lead to a reduced ability to perform activities of daily living (ADL). Successful execution of ADLs relies on mobility and dexterity. The activation of small muscles to synchronise hand and finger movement during ADLs is under neural control. Reduced use of muscles leads to inefficient recruitment of motor units, therefore reduced motor skills2. Recommended hand exercises for SSc train range of motion, but less dexterity. Virtual rehabilitation programmes have shown beneficial effects on both range and dexterity. The aim of this study was to compare the effect of physiotherapy and virtual rehabilitation on finger dexterity and ability to perform ADLs.

**Material and Methods:** Twenty SSc patients were recruited (54.8yrs± 23.1yrs; female: n=19, male = 1) and randomly split into two groups (n= 9, drop out: n =2) performing 90min of hand exercises per week for four weeks. One group followed a novel virtual rehabilitation programme (playing a computer game using hand movements). The second group completed physiotherapy exercises. Prior to, immediately after and four weeks after completion of the exercises patients conmpleted the Cochin Hand Function Scale (CHFS). A finger dexterity test on a customised keyboard was completed using Digits 2-4. The average tapping speed over 15 seconds was calculated. A two-way mixed design ANOVA for the CHFS and finger dexterity test was conducted in SPSS and the change in CHFS and dexterity were correlated using bivariate two-tailed Pearson correlation.

**Results:** Both interventions showed significant within-group improvements in ability to perform ADLs (Figure 1) (p = 0.03) and finger dexterity (Figure 2) (p = 0.02) before and after training. Whilst finger dexterity was maintained for four weeks without exercises, the ability to perform ADLs was reduced in the virtual rehabilitation group (p = 0.05). The group effect was insignificant (p = 0.401). Change in CHFS and finger dexterity between test sessions was not correlated (r = -0.09, p= 0.72). This pattern was identical in the non-dominant hand, showing there is not effect of hand dominance.

**Conclusions:** Exercise improved functional and neural aspects of hand function. The improvement in finger dexterity suggests that patients not only suffer a structural limitation but also a loss in fine motor skills. Hand function assessment and rehabilitation in scleroderma should therefore also integrate a dexterity test in addition to mobility.

## P.298

## SYSTEMIC SCLEROSIS OVERLAP SYNDROME IN 10 YEARS

O. Desinova, M. Starovoytova, L. Ananieva

*V.A.Nassonova research Rheumatology Institute, Moscow, Russia*

**Introduction:** Many patients with Systemic Sclerosis (SSc) have features of another autoimmune condition like rheumatoid arthritis (RA), polymyositis/dermatomyositis (PM/DM) and others. SSc-RA and SSc-PM/DM are rare forms of overlap diseases of connective tissue are difficult to diagnose in diagnostic and differently-diagnostic plan.

**Material and Methods:** To study evolution of SSc-RA and SSc-PM/DM. There were 100 pts SSc overlap syndrome: 68 SSc-PM/DM and 32 SSc-RA (17 male, 83 females; mean age 45±14,4; disease duration 7 [2-10] years; 10 years follow-up).

**Results:** 71 % pts has had SSc-PM/DM and SSc-RA within the first three years of disease and 40% of them during the 1st year after onset. The first symptoms included Raynaud’s phenomenon, edema hands, arthralgia and rare isolated joint and skeletal muscle involvement (7% and 2%).

All pts were treated corticosteroids and 74% of them received cytotoxic (methotrexate 48%; cyclophosphamide 10%; azathioprine 5%), hydroxychloroquine 11%, D-penicillamine 7%.

The SSc-PM/DM pts have decreased skin induration (limited SSc/diffuse SSc 68%/32% and 100%/0%), hyperpigmentation (27% and 18%), skin symptoms of DM (44% and 7%), joint involvement (56% and 15%) for 10 years. PM was in remission in all patients. Raynaud’s syndrome progressed with the development mainly scars (18% and 37%), increased telangiectasias (50% and 59%), calcinosis ( 32% and 56%), osteolysis (23,5% and 26%), conduction blocks(53% and 57%) and arrhythmia (15% and 18%), interstitial lung disease (12% and 15%), esophageal involvement (78% and 88%).

The peripheral symptoms SSc-RA have not progressed for 10 years and have decreased: skin induration (limited SSc/diffuse SSc 97%/3% and 100%/0%), hyperpigmentation (34% and 9%), flexion contractures (81% and 72%), arthritis (100% and 78%), rheumatoid nodules disappeared. Raynaud’s syndrome was less expressed. However clinical features SSc increased: telangiectasias (37,5% and 47%), calcinosis ( 31% and 47%), osteolysis (25% and 28%), conduction blocks(53% and 56%) and arrhythmia (9% and 19%), interstitial lung disease (9% and 19%), esophageal involvement (66% and 69%).

The specific features of overlapping SSc evolution included augmentation of SSc-characteristic symptoms - both, peripheral – teleangiectasias, calcification, osteolysis and digital trophic lesions, mainly in SSc-PM/DM pts, and visceral - involving heart, lungs, and esophagus, which determined the unfavorable prognosis. RA manifestations SSc overlap pts tended to decrease, while signs of PM tended to resolve.

**Conclusions:** Timely detection of overlapping SSc pathological symptoms with administration of adequate therapy and dynamic monitoring of patients will improve the prognosis and outcomes of the disease.

## P.299

## COMPARISION OF RELAPSING POLYCHONDRITIS PATIENTS WITH/WITHOUT ARTHROPATHY ONSET

X. Cao, Y. Hou, D. Xu, M. Li, Q. Wang, X. Zeng

*Department of Rheumatology and Clinical Immunology, Peking Union Medical College Hospital, Chinese Academy of Medical Sc, Beijing, China*

**Introduction:** We made the research to explore the distinct clinical characteristics of relapsing polychondritis (RP) patients with arthropathy at presentation.

**Material and Methods:** We retrospectively reviewed the medical records of 201 RP patients who were hospitalized between December 2005 and February 2019 in Peking Union Medical College Hospital, China. After excluding 16 patients who overlapped with other autoimmune diseases and malignancy, 185 RP patients were included. There were 16 RP patients with arthropathy at presentation. The other 169 RP patients were without arthropathy at initial manifestation. Single factor analysis was performed between the RP patient subgroup with arthropathy at presentation and RP patient subgroup without arthropathy at presentation.

**Results:** The percentage of RP patients with arthropathy at presentation in 185 hospitalized RP patients was 8.65%. The average age at diagnosis was 46.19 years ranging from 32 years to 65 years. There were 5 patients misdiagnosed as rheumatoid arthritis (RA), 1 misdiagnosed as reactive arthritis, and 1 misdiagnosed as erythema nodosum. Compared with RP patients without arthropathy at presentation, RP patients with arthropathy at presentation had a longer disease duration (37.5±66.5 months VS 9±11 months, p=0.001), a longer time of diagnostic delay (24±41.25 months VS 7±9 months, p=0.004), and higher incidence of eye (62.5% VS 36.09%, p=0.038) and nervous system involvement (43.75% VS 15.38%, p=0.013)

**Conclusions:** RP patients with arthropathy at presentation were most likely to be misdiagnosed as RA. They were characterized by longer disease duration and diagnostic delay. The eye and nervous system involvement were common.

## 10. Gastrointestinal/Nutrition

## P.300

## SEVERITY AND EVOLUTION OF GASTRO-INTESTINAL INVOLVEMENT IN PATIENTS WITH SYSTEMIC SCLEROSIS IN TWO LARGE AND INDEPENDENT COHORTS

N. Van Leeuwen^1^, H. Fretheim^2^, Ø. Molberg^2^, T. Huizinga^1^, J. De Vries-Bouwstra^1^, A.-M. Hoffman-Vold^2^

^1^*Leiden University Medical Center, Leiden, The Netherlands*, ^2^*Oslo University Hospital, Oslo, Norway*

**Introduction:** In Systemic Sclerosis (SSc), the gastro-intestinal tract (GIT) is reported to be the most affected organ after the skin. The aim of this study was to evaluate the severity and determinants of GIT involvement in SSc, progression of GIT involvement over time and the effect of standard care treatment for SSc.

**Material and Methods:** All SSc patients fulfilling the 2013 SSc criteria followed at the Leiden University Medical Center (LUMC) or Oslo University Hospital (OUH), with available clinical and treatment data and serial GIT measurements using the UCLA GIT 2.0 questionnaire were included. GIT involvement was assessed at baseline and annually in both cohorts and divided into mild, moderate and severe GIT disease. Progression of GIT involvement was determined for the total GIT involvement and for each subdomain using the minimal clinical important difference. Logistic regression was applied to identify risk factors associated with GIT involvement. Linear mixed-effect regression model analysis was used to assess changes in UCLA GIT scores over time, to control for repeated measurements and to identify predictive factors associated with any change in UCLA GIT score during the observation period.

**Results:** In total 834 SSc patients were included (Table 1), of these 536 (64%) had at least two GIT scores, and 270 (32%) had at least four GIT scores available. The severity of GIT involvement at baseline was similar in both cohorts with 6% (LUMC) and 8% (OUH) patients showing severe GIT involvement. Diffuse cutaneous disease (OR 3.7 (1.8-7.7)), presence of anti-centromere antibody (ACA) (OR 1.7 (1.1-2.8)) and corticosteroid use (OR 2.4 (1.4-4.1)) were associated with increased GIT involvement, whereas use of calcium channel blockers (CCB) (OR 0.4 (0.3-0.7)) was associated with less GIT involvement. Mycophenolate mofetil, methotrexate or cyclophosphamide use was not associated with GIT involvement on any domain. General progression of GIT involvement occurred in 28% of the patients during the first year. Reflux (29%), distension/bloating (38%) and constipation (25%) showed the most progression, fecal soilage (7%) the least. In the mixed-effect models, baseline characteristics predictive for progression of total GIT involvement over median 3.4 years were disease duration, female sex, and presence of ACA. Treatment with CCB was protective for progression of GIT involvement.

**Conclusions:** Here we show, that GI disease is frequent and progressive especially in ACA positive and female SSc patients. Strikingly, SSc treatment for other disease related problems can influence GI involvement significantly, with treatment with CCB being protective.

**Figure:**
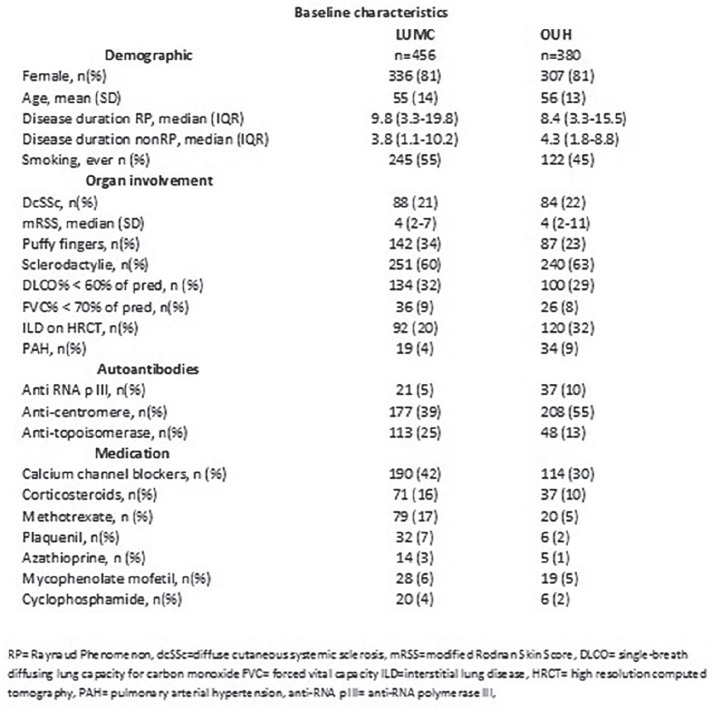


## P.301

## METABOLITES DERIVED FROM GUT MICROBIOTA IN THE BLOOD OF PATIENTS WITH SYSTEMIC SCLEROSIS

M. Sikora, A. Stec, M. Olszewska, L. Rudnicka

*Medical University of Warsaw - Department of Dermatology, Warsaw, Poland*

**Introduction:** Systemic sclerosis is a chronic disease of unknown etiology characterized by diffuse fibrosis and vascular abnormalities. Accumulating evidence suggests that alterations in the composition of the gut microbiome (dysbiosis) and intestinal barrier permeability may play a role in the pathogenesis of systemic sclerosis. Disruption of gut barrier can cause the translocation of bacteria metabolites, which further induce or aggravate the systemic inflammation. The aim of the study was to determine the concentration of bacterial metabolites - indoxyl sulfate and N-trimethylamine oxide (TMAO) in the blood of patients with systemic sclerosis.

**Material and Methods:** The study involved 60 patients with systemic sclerosis and 60 age, sex and BMI-matched controls. The serum concentrations of indoxyl sulfate and TMAO were determined by high-performance liquid chromatography.

**Results:** Systemic sclerosis patients, compared to the control group, were characterized by a significantly higher concentration of TMAO (385.2 ± 93.5 vs 205.6 ± 80.7 ng / ml, p <0.05) and indoxyl sulfate (1096.5 ± 222.7 vs 605.4 ± 125.2 ng / ml, p <0.05). The concentration of bacterial metabolites showed a positive correlation with EUSTAR activity index (TMAO: r = 0.34, p<0.05; indoxyl sulfate: r = 0.52, p<0.05). In addition, progressive involvement of lung and increase in NT-proBNP concentration was demonstrated across TMAO tertiles.

**Conclusions:** Alterations in the microbiota-gut-skin axis play significant role in the pathogenesis of systemic sclerosis. Modulation of intestinal barrier represents a new promising therapeutic approach for autoimmune diseases.

## P.302

## BODY COMPOSITION IN SCLERODERMA PATIENTS AND THE ASSOCIATION WITH THE DISEASE ACTIVITY, SYSTEMIC INFLAMMATORY CYTOKINES/CHEMOKINES, LIPID PROFILE AND NUTRITION STATUS

S. Oreska^1^, M. Spiritovic^2^, P. Cesak^2^, M. Cesak^2^, H. Storkanova^1^, H. Smucrova^3^, B. Hermankova^2^, O. Ruzickova^1^, K. Pavelka^1^, L. Senolt^1^, J. Vencovsky^1^, R. Becvar^1^, M. Tomcik^1^

^1^*Institute of Rheumatology, Department of Rheumatology, 1st Faculty of Medicine, Charles University, Prague, Czech Republic*, ^2^*Faculty of Physical Education and Sport, Department of Physiotherapy, Charles University, Prague, Czech Republic*, ^3^*Institute of Rheumatology, Prague, Czech Republic*

**Introduction:** Fibrosis of the skin and visceral organs, especially digestive tract, and musculoskeletal involvement in systemic sclerosis (SSc) can have a negative impact on body composition, physical activity and nutritional status. The aim of our cross-sectional study was to assess body composition and physical activity of SSc patients and healthy controls (HC) and the association with systemic levels of selected inflammatory cytokines/chemokines, laboratory markers of nutritional status and lipidogram in SSc.

**Material and Methods:** 59 patients with SSc (50 females; mean age 52.5; disease duration 6.7 years; lcSSc:34/dcSSc:25) and 59 age-/sex-matched HC (50 females, mean age 52.5) without rheumatic or tumour diseases were included. SSc patients fulfilled ACR/EULAR 2013 criteria. We assessed body composition (densitometry: iDXA Lunar, bioelectric impedance: BIA-2000-M), physical activity (Human Activity Profile, HAP questionnaire), disease activity (ESSG activity index), serum levels of 27 cytokines/chemokines (commercial multiplex ELISA kit, Bio-Rad Laboratories) and chosen parameters of nutrition and lipidogram. Data are presented as mean SD

**Results:** Compared to HC, patients with SSc had significantly lower body mass index (BMI, 27.4±8.3 vs. 22.4±4.3 kg/m2), body fat % (BF%, iDXA: 38.0±7.6 vs. 32.6±8.2 kg; BIA: 31.3±7.6 vs. 24.3±7.9 kg) and visceral fat weight (VF, 1.0±0.8 vs. 0.5±0.5 kg), and also significantly decreased lean body mass (LBM, iDXA: 51.9±8.4 vs. 47.8±7.0 kg; BIA: 45.4±7.3 vs. 40.9±6.8 kg), and bone mineral density (BMD, 1.2±0.1 vs. 1.0±0.1 g/cm2). Compared to HC, patients with SSc had increased extracellular mass/body cell mass (ECM/BCM, 1.03±0.1 vs. 1.28±0.4) ratio, reflecting deteriorated nutritional status and worse muscle predispositions for physical activity (p<0.05 for all comparisons). Increased ECM/BCM in SSc was associated with higher disease activity (ESSG), increased skin score (mRSS) and inflammation (CRP, ESR), and with worse quality of life (HAQ, SHAQ), fatigue (FSS), and decreased physical activity (HAP). ESSG negatively correlated with BF%. HAP positively correlated with BMD. Serum levels of several inflammatory cytokines/chemokines (especially IL-1b, IL-5, IL-6, IL-8, IL-17, TNF, Eotaxin) and markers of nutrition and lipid metabolism were associated with alterations of body composition (p<0.05 for all correlations).

**Conclusions:** In our study we found significant negative changes in body composition of our SSc patients, compared to healthy age-/sex-matched individuals. These changes are associated with the disease activity and physical activity, and could reflect the nutritional status, and gastrointestinal and musculoskeletal involvement in SSc. Serum levels of certain inflammatory cytokines/chemokines and markers of nutrition and lipid metabolism were associated with alterations of body composition in SSc patients.

Supported by AZV-NV18-01-00161A, MHCR-023728, GAUK-312218

## P.303

## SURVEY ON DYSPHAGIA USING SIMPLE QUESTIONNAIRE EAT-10 IN PATIENT WITH SYSTEMIC SCLEROSIS

T. Naganawa^1^, S. Yoshida^1^, A. Kuwabara^2^, M. Sawada^1^, N. Watanabe^1^, M. Suzuki^1^, A. Umeda^1^, K. Ashihara^1^, M. Kurimizawa^1^, D. Hirano^1^, Y. Inamoto^3^, T. Hashimoto^1^, J. Nishino^1^, S. Fukaya^1^, S. Sibata^4^, E. Saitoh^4^, H. Yasuoka^1^

^1^*Fujita Health University School of Medicine - Department of Internal Medicine, Section of Rheumatology, Toyoake, Japan*, ^2^*Fujita Health University School of Hospital - Department of Rehabilitation, Toyoake, Japan*, ^3^*Fujita Health University School of Health Sciences - Faculty of Rehabilitation, Toyoake, Japan*, ^4^*Fujita Health University School of Medicine - Department of Rehabilitation Medicine I, Toyoake, Japan*

**Introduction:** Gastrointestinal (GI) tract is the most commonly affected among organ involvement in patients with systemic sclerosis (SSc). Symptom of upper GI is thought to be derived from esophageal lesions such as gastroesophageal reflex or motion dysfunction. However, swallowing function in SSc patients has not been evaluated so far. Our aim was to investigate the actual condition of dysphagia, abnormality of swallowing functional process in patient with SSc.

**Material and Methods:** Patients with SSc were randomly selected from outpatient clinic at Fujita Health University between from November to December 2016. Patients with Polymyositis/Dermatomyositis (PM/DM) were selected as controls. The feature of dysphagia was evaluated by the existence of “feeling” difficulty with swallowing and the simple questionnaire for swallowing functional evaluation, EAT-10, which is consisted of 10 questions which reflect the 3 components of swallowing issues: 1)nutritional and quality of life, 2)functional difficulty for liquid swallowing, and 3) difficulty for solid swallowing. Clinical parameters were collected from patients’ record retrospectively.

**Results:** Ten patients with SSc and 11 with PM/DM were included in this study. Male:Female ratio was 3:7 and 3:7, and the mean age of SSc and PM/DM was 68.3±1.5, 55.0±2.5 years, respectively (p=0.002). Based on self-assessment, 50% with SSc felt difficulty with swallowing and 40% did not, whereas 36% with PM/DM did and 46% did not. By EAT-10, there were 3 cases (30%) in SSc and 2 cases (14%) in PM/DM who were suspected to have dysphagia based on the score, which is a same trend with self-assessment. We stratified patients with SSc by existence of gastroesophageal reflux disease (GERD) and then compared between two groups. Interestingly, as for the proportion of patients whose score was 3 or above, which is the threshold of abnormality, only 17% of patients with GERD and 50% without GERD scored 3 or above. Furthermore, when we looked at each item of EAT-10, the ratio in all questions except one were high in patients without GERD, suggesting that this questionnaire can discriminate the patients with esophageal lesion and swallowing difficulties.

**Conclusions:** These results suggest that swallowing difficulty is a newly-identified abnormality in upper GI tract in SSc and can discriminate from esophageal lesions. EAT-10 might be a good questionnaire for help with identification of these patients without invasive evaluations.

## P.304

## ESOPHAGEAL INVOLVEMENT AND DETERMINANTS OF PERCEPTION OF ESOPHAGEAL SYMPTOMS AMONG EAST ASIAN PEOPLE WITH SYSTEMIC SCLEROSIS IN THE ERA OF HIGH-RESOLUTION MANOMETRY

H.-S. Kim, W. Choi, M. Kim, T.H. Lee

*Soonchunhyang university Seoul Hospital, Seoul, South Korea*

**Introduction:** Toward the esophageal involvement in East Asian patients with systemic scleroderma(SSc), our study was aimed to (1) characterize esophageal motor function; (2) evaluate the relationships among data from esophagogastroduodenoscopy (EGD), high-resolution manometry(HRM) and 24-hour esophageal multichannel intraluminal impedance monitoring combined with pH-metry (MII-pH); (3) elucidate determinants of perception of esophageal symptoms.

**Material and Methods:** We reviewed prospectively collected databased of HRM (n=46), EGD (n=41), and MII-pH (n=37) from consecutive 46 patients with systemic sclerosis (42 females; mean age 50.1 years) undergoing esophageal tests at our motility clinic from June 2013 to Sep 2018. The SSc was diagnosed using with the 2013 American College of Rheumatology SSc criteria. The types of SSc were categorized as having limited cutaneous (lcSSc) or diffuse cutaneous (dcSSc) systemic disease according to the distribution of skin involvement according to LeRoy et al. This study was approved by the Institutional Review Board. Patients who had incomplete clinical assessment were excluded. Complete data were available for 46 patients (lcSSc, 26 and dcSSc, 20) with consecutive HRM studies between June 2013 and September 2018. Relevant data were collected on each patient, including age, sex, body mass index (BMI) as well as clinical information.

**Results:** The most common HRM diagnosis was normal (39.1%) followed by ineffective esophageal motility (23.9%) and absent contractility (21.7%). 12.2% of overall SSc patients had erosive esophagitis, which was more frequently observed in patients with absent contractility compared than patients with normal motility (44.5% vs. 0%, P=0.01). There were no significant differences in the proportions of major motility disorders or erosive esophagitis between asymptomatic and symptomatic patients. Pathologic acid exposure was observed in 6 patients (20%) and positive symptom association in 18 patients (60%) from MII-pH tests of symptomatic patients.

**Conclusions:** Esophageal involvement among East Asian patients with SSc was characterized by heterogeneous motility patterns with a higher prevalence of normal motility and lower prevalence of erosive esophagitis. The presence of esophageal symptoms is a poor predictor of esophageal dysmotility and erosive esophagitis. Reflux hypersensitivity or functional heartburn might be partly attributed to the perception of esophageal symptoms in the subset of SSc patients who have neither gastroesophageal reflux disease nor esophageal dysmotility.

## P.305

## PREVALENCE OF GASTROESOPHAGEAL DISEASE IN SYSTEMIC SCLEROSIS AND ITS POTENTIAL IMPACT ON LUNG DISEASE

S. Kafaja, T. Shagroni^1^, R. Saggar^1^, A. Aly^2^, I. Valera^1^, P. Clements^1^, J. Conklin^1^, D. Furst^1,3^

^1^*David Geffen School of Medicine, Department of Medicine, Division of Rheumatology, Los Angeles, USA*, ^2^*Alexandria School of Medicine, Alexxandria, Egypt*, ^3^*Pacific Arthritis Care, Los Angeles, USA*

**Introduction:** Systemic sclerosis (SSc) frequently involves upper gastrointestinal (GI) symptoms, with GI involvement being the leading cause of morbidity. Meanwhile, interstitial lung disease (ILD) and pulmonary hypertension (PH) are the leading cause of mortality in SSc. Emerging data indicates that reflux disease may play a role in ILD through chronic microaspiration. A potential causality between the two variables has yet to be established.

The aim of this study is to investigate the prevalence of Upper GI motility disorders and reflux in SSc patients (pts) and to evaluate the impact of GI findings on pulmonary outcomes.

**Material and Methods:** This is a retrospective chart review that was carried out at the University of California, Los Angeles (UCLA). All patients met the SSc 2013 ACR/EULAR classification criteria. Esophageal evaluation was carried out using at least one of the following modalities: high-resolution esophageal manometry (HREM), ambulatory pH testing and/or EGD. Data collected included patient demographics, scleroderma characteristics, motility data, presence of ILD, PH, oxygen requirement, and pulmonary function tests (PFTs). Statistical analyses included both descriptive as well as ANOVA testing for continuous, and Chi square testing for dichotomous variables.

**Results:** Our study cohort included 122 pts with SSc with a mean age± SD of 66± 9 at GI evaluation, with SSc diagnoses predating GI symptoms by approximately 15 years. 93% of pts were female, 68% with limited and 32% with diffuse disease.

Most pts underwent HREM and EGD. Approximately 70% of the pts had absent contractility on manometry, while 24% of pts had ineffective esophageal motility. Hiatal hernia, an anatomical finding associated with reflux, was found in one third of pts. Of those who underwent EGD, 37% had evidence of erosive esophagitis, while only 23% of pts had a definitive diagnosis of GERD based on EGD. ILD was noted in 55% of pts. For simplicity, we included PFTs done within one year of the index GI study.

There was no significant difference in the PFTs in pts of different HREM diagnoses, between pts with and without a diagnosis of GERD, or between pts with ILD with normal and abnormal motility.

**Conclusions:** Our study is one of the largest single-center cohorts of SSc patients characterizing esophageal motility, reflux and pulmonary outcomes. There was no association found between esophageal dysmotility and/or GERD with pulmonary lung disease (via PFTs). Additional characterization of GI disease and early radiographic findings of ILD is helpful and is underway.

## P.306

## PREDICTORS OF SIGNIFICANT WEIGHT LOSS IN SYSTEMIC SCLEROSIS - A STUDY FROM THE EULAR SCLERODERMA TRIALS AND RESEARCH (EUSTAR) DATABASE

M. Hughes^1^, C. Heal^2^, J. Wilkinson^2^, EUSTAR Investigators^3^

^1^*Centre for Musculoskeletal Research, Faculty of Biology, Medicine and Health, Manchester, United Kingdom*, ^2^*Centre for Biostatistics, Manchester, United Kingdom*, ^3^*EUSTAR, Basel, Switzerland*

**Introduction:** Gastrointestinal involvement occurs in almost all patients SSc and is associated with significant disease-related morbidity and mortality. There is a strong need to identify potential risk factors for use in routine clinical practice which could allow the earlier identification of patients who are likely to develop significant weight loss, with a view to early intervention strategies. The aim of this study was to examine potential clinical risk factors of significant weight loss in patients with SSc.

**Material and Methods:** A longitudinal analysis of the patients with SSc enrolled in the EUSTAR database. Significant weight loss was defined as 4.5Kg at 5 months onwards or least 5% of their body weight at 5 months onward. Patients with a recorded second visit after 3 months and before 12 months were included in the analysis. In comparing the characteristics of significant weight loss patients with non-weight loss patients we used a chi-squared test of independence for categorical variables, Mann-Whitney test for non-normally distributed continuous variables and an independent samples t-test for the only normally distributed continuous variable age.

**Results:** We compared 438 patients with significant weight loss compared to 3169 patients without weight loss. Median (IQR) significant and relative weight loss was 6Kg (4.3 to 8) and 8% (6 to 12%), respectively.

No difference in age was observed between groups at baseline (54.6 vs. 54.1 years). Clinical features associated with significant weight loss was female sex (P=0.022), diffuse cutaneous SSc (P=0.009), digital ulcer disease (P=0.017), shorter disease duration (P=0.002), elevated ESR (P=<0.001), and CRP (P=0.029), elevated CK (P=0.001), pulmonary hypertension (P=<0.001), abnormal diastolic dysfunction (P=0.010) and interstitial lung disease (P=0.001). It is worth noting however that after using Holm-Bonferroni to account for multiple testing, only shorter disease duration, ESR, elevated CK, pulmonary hypertension and interstitial lung disease remain statistically significant.

Malabsorption (P=0.829) and oesophageal (P=0.347), stomach (P=0.502) and intestinal (P=0.368) symptoms were not associated with significant weight loss.

**Conclusions:** Our study has identified a number of clinical associations with significant weight loss in patients with SSc. Relying on gastrointestinal symptoms may be insensitive to identify patients at risk of significant weight loss. Further research including prospective studies is indicated to develop predictive models and to examine potential biomarkers of significant weight loss in SSc.

## P.307

## GASTROINTESTINAL INVOLVEMENT IS ASSOCIATED WITH DEPRESSION AND ANXIETY IN PATIENTS WITH SYSTEMIC SCLEROSIS

A.M. Gheorghiu^1^, C. Vrancianu^1^, B. Busuioc^1^, C. Draganescu^1^, A. Briceag^1^, L. Macovei^1^, M. Sasu^1^, I. Ancuta^1^, V. Stoica^1^, M. Bojinca^1^, C. Mihai^1,2^

^1^*Cantacuzino Hospital, Carol Davila University of Medicine and Pharmacy, Bucharest, Romania*, ^2^*University Hospital Zurich, Zurich, Switzerland*

**Introduction:** Gastrointestinal (GI) involvement is frequent among patients with systemic sclerosis (SSc), sometimes with severe and life-altering manifestations.

Objectives: To evaluate the frequency and characteristics of GI involvement in a single center cohort of patients with SSc, especially the impact on the emotional state of SSc patients.

**Material and Methods:** Methods: Thirty-three consecutive patients with SSc fulfilling the ACR/EULAR 2013 classification criteria and who also presented GI symptoms were included. Superior digestive endoscopy and extensive assessment per the recommendations of EUSTAR were performed in all patients. In addition, the University of California at Los Angeles Scleroderma Clinical trial Consortium Gastrointestinal Tract (UCLA GIT 2.0), Bad Nauheim digestive involvement questionnaire, Hospital Anxiety and Depression Scale (HADS) and Beck’s Depression Inventory (BDI) were done for all patients. IBM SPSS v. 20 was used for statistical analysis. Mann-Whitney U test for continuous variables, chi-square for categorical variables and Spearman’s rho correlation coefficient were used to investigate associations between GI involvement and disease characteristics and GI tract-related and depression and anxiety questionnaires.

**Results:** Results: GI reflux was the most frequent symptom in our cohort (26/33 patients), followed by abdominal distension (18/33/patients). 7 patients had severe GI manifestations (UCLA GIT 2.0). Depression (BDI score greater or equal to 11) was present in a large number of cases (25/33 patients).

The presence of GI reflux was significantly associated with depression (mean BDI 12.6 vs. 4.3, Mann-Whitney U test, p=0.01). Moreover, a higher UCLA GIT 2.0 score was correlated with depression on BDI (p=0.003). Fecal incontinence was significantly associated with anxiety (HADS anxiety mean score 18.3 vs. 9.3, Mann-Whitney U test, p=0.006). Endoscopy changes such as esophagitis and esophageal strictures were associated only with corticosteroids treatment (p=0.01). No other associations with disease characteristics were found.

**Conclusions:** Conclusions: The presence of GI involvement was high, as expected. GI reflux and fecal incontinence had the most impact on the emotional state of patients with SSc.

## P.308

## RELATIONSHIP BETWEEN INTERSTITIAL PULMONARY DISEASE AND ESOPHAGIC PATHOLOGY IN SYSTEMIC SCLEROSIS. STUDY OF ONE COHORT OF 92 PATIENTS

P. Garcia De La Peña Lefebvr^1^, G. González Arribas^2^, M. Novella Navarro^3^, J. González Martín^4^

^1^*Fundación Investigación Inmunes, Madrid, Spain*, ^2^*Hospital Universitario La Paz, Madrid, Spain*, ^3^*Complejo Hospitalario Universitario A Coruña, A Coruña, Spain*, ^4^*Hospital Universitario Hm Sanchinarro, Madrid, Spain*

**Introduction:** Introduction: Digestive involvement, especially esophageal is common in Systemic Sclerosis (SSc). Simultaneously, the presence of Interstitial lung disease (ILD) is a common affectation in these patients. Although the pathogenic mechanisms of ILD are not elucidated, there is evidence that esophageal motility disorders, gastroesophageal reflux (GER) are involved in the development of ILD in different situations, including SSc.

Objective: Establish the association between ILD and esophageal involvement in patients with SSc.

**Material and Methods:** Material and methods: Observational, cross-sectional study of a cohort of 92 patients diagnosed with SSc according to ACR-EULAR 2013 and LeRoy Medsger criteria 2001 for early scleroderma, treated in rheumatology consultations at a University Hospital between 2007 and 2018. It was established as a dependent variable the presence of ILD diagnosed by high-resolution thoracic CT, expressed as dichotomous variable. In addition to take into account respiratory function variables (FVC, DLCO, TLC). The independent variables were those referred to esophageal involvement (pH-metry, DeMeester index and esophageal manometry) in addition to other clinical and sociodemographic variables.

Statistical analysis: Descriptive of frequencies, means and deviations, medians and ranges. Linear correlation model. As level of significance statistical p <0.05, 95% CI. SPSS Statistics v.23®.

**Results:** Results: Of the 92 patients (94.6% women and 5.4% men). The mean age was 48 ± 14 years, and the average evolution time was 5.9 ± 4.9 years. 25% of patients presented with ILD, this being the most frequent affectation in patients with diffuse cutaneous SSc dcSSc. The presence of ILD was related to esophageal manometry pathological (p = 0.005). Another finding was the relationship of the decrease in FVC with pathological pH-metry (p = 0.045).

**Conclusions:** Conclusions: In this study we observed a significant association between pulmonary and esophageal involvement of patients with SS,c probably the alteration of peristalsis and sphincter dysfunction lead to gastric acid microaspirations that influence progressive airway damage.

## P.309

## MALNUTRITION IN SYSTEMIC SCLERODERMA: IS ‘THE 15 PERCENT’ RULE APPLICABLE?

A. Burlui, M. Graur, A. Gherasim, A. Cardoneanu, L. Macovei, E. Rezus

*Grigore T Popa University of Medicine and Pharmacy, Iasi, Romania*

**Introduction:** A systematic review focused on determining the prevalence of severe manifestations of scleroderma described the ‘15 percent rule’ in scleroderma. In support of this theory, Muangchan et al. found a prevalence of close to 15% for cardiac involvement, HTAP, diastolic dysfunction, impaired pulmonary function parameters, arthritis and myositis. The main aim of the present research was to determine the prevalence of certain clinical manifestations and malnutrition in a cohort of patients with systemic scleroderma (SS).

**Material and Methods:** We conducted a single center observational study; the inclusion criteria were: age over 18 years, definite diagnosis of SS, signed informed consent. Exclusion criteria were: age below 18 years, overlap syndromes and patients’ refusal to participate in the study. Malnutrition risk was estimated using the Malnutrition Universal Screening Tool (MUST). The statistical data analysis was performed using IBM SPSS Statistics v20.

**Results:** The study group consisted of 67 patients. The prevalence of arrhythmias and/or conduction disorders was close to 15% (16.4%), and interstitial pulmonary fibrosis was associated with HTAP in proportion of 16.7%. Moreover, the high risk of malnutrition (MUST) was observed in 14.9% of the study group. Whereas for other types of visceral involvement (PAH, arrhythmias and/or conduction disorders) we did not identify a higher MUST risk, subjects with interstitial pulmonary fibrosis had a significant association with the risk of malnutrition. In this regard, patients with pulmonary fibrosis were more often classified in the moderate/high risk categories, the results being statistically significant. Also, patients with cardiopulmonary involvement suffered weight loss (over 5% of the initial body mass) more frequently.

**Conclusions:** Muangchan et al. do not mention the applicability of this rule to the risk of malnutrition, although this variable was considered as a severe manifestation at the time of selection of the relevant resources. Our results suggest that the risk of malnutrition in the SS could fall under the ‘15 percent rule’. Malnutrition is common and is associated with organ damage in patients with SS.

## P.310

## GASTROPARESIS IN SYSTEMIC SCLEROSIS PATIENTS: CLINICAL CHARACTERISTICS

R. Brun^1^, A. Balbir-Gurman^2^, Y. Braun-Moscovici^2^

^1^*Department of Gastroenterology, Rambam Health Care Campus, Haifa, Israel*, ^2^*Rheumatology Institue, Rambam Health Care Campus, Rappaport Faculty of Medicine, Technion, Haifa, Israel*

**Introduction:** Systemic Sclerosis (SSc) often involves the gastrointestinal tract. Esophageal involvement is the most common, although the rest of GI tract is often affected and significantly contributes to patents’ morbidity. Gastroparesis (GP) is a disease with distinct clinical features, diagnosed by measuring gastric emptying. The gastroparesis is linked to malnutrition, significant morbidity and impairment of quality of life, and not uncommon in scleroderma. The goal of the study was to explore the clinical characteristics of gastroparesis in SSc patients.

**Material and Methods:** Retrospective review of clinical records of multidisciplinary gastroenterology- rheumatology clinic of a tertiary referral hospital.

**Results:** Thirty SSc patients visited a multidisciplinary gastroenterology- rheumatology clinic during 2015-2019 years. All patients presented with gastrointestinal complaints. The most common upper GI complaints were heartburn (73%) and regurgitation (73%), followed by early satiety (67%), dysphagia (50%), abdominal pain (47%), vomiting (43%), bloating (37%), and nausea (37%). Malnutrition was present in 40% of patients. 100% patients underwent upper endoscopy, and 50% underwent gastric emptying scintigraphy. Most of the pts underwent gastric emptying test following a visit at the multidisciplinary clinic. Out of 15 patients who underwent gastric emptying scintigraphy, 5 (33%) were diagnosed with gastroparesis. 2 additional patients were diagnosed by presence of food in stomach on upper endoscopy, after prolonged fasting. Overall 7 pts (23%) with GP were identified in our cohort. All patients with prolonged GI emptying had also a clinical presentation of GP. Four of the GP patients had diffuse SSc and 3 had limited SSc, with the mean age 53 yo (18-78 yo). GP patients had lower mean body weight (53 vs 66.9 kg, p= 0.02), were more commonly malnourished (71% vs 30%, p= 0.05), and had significantly more complaints of early satiety (100% vs 57%), nausea (71% vs 26%), vomiting (71% vs 35%), abdominal pain (71% vs 39%), and dysphagia (71% vs 43%).

**Conclusions:** Gastroparesis should be an important consideration when assessing SSc patients with upper GI symptoms. Patients who present with malnutrition or symptoms of early satiation, abdominal pain, nausea or vomiting should be actively referred to diagnostic tests to identify GP and treated accordingly.

## P.311

## DURATION AND SYSTEMIC SCLEROSIS SUBTYPE ARE ASSOCIATED WITH DIFFERENT GUT MICROBIOME PROFILES

Y. Braun-Moscovici^1^, S. Ben Simon^2^, K. Dolnikov^1^, S. Giries^1^, D. Markovits^1^, Y. Tavor^1^, K. Toledano^1^, M.A. Nahir^1^, A. Balbir-Gurman^1^, O. Koren^2^

^1^*B Shine Rheumatology Institute, Rambam Health Care Campus, Rappaport Faculty of Medicine, Technion, Haifa, Israel*, ^2^*Koren Lab, Faculty of Medicine, Bar Ilan University, Safed, Israel*

**Introduction:** A growing body of evidence suggests that the gut microbiota plays a significant role in the development of autoimmune diseases. Altered microbiota composition was associated with gastrointestinal and extraintestinal features in systemic sclerosis (SSc) patients.

Our aims were to look for differences in gut microbiota between SSc patients regarding disease duration, disease subset and occurrence of digital ulcers (DU).

**Material and Methods:** SSc patients seen at our center were recruited in a prospective study. The exclusion criteria included antibiotic or probiotic treatment during the month prior to recruitment, recent hospitalization, BMI>30, diabetes mellitus or concomitant inflammatory bowel disease. Fecal samples were processed and 16S rRNA gene sequences were analyzed using the QIIME2 packageWeighted (quantitative) and unweighted (qualitative) UniFrac distances, alpha diversity for richness and homogeneity, taxa plots for species and phyla and ANCOM analyses were performed.

**Results:** During July 2018-May 2019, 26 SSc patients (mean age [SD] 53[12.7] years) and disease duration 8.8 [7.1] years) fulfilled the criteria and were willing to participate in the study. Thirteen patients had diffuse SSc, 16 patients had active DU, 8 patients had Raynaud’s phenomenon only without DU, 2 patients had past DU. The microbiota was significantly more similar between patients without active DU compared to those with active DU (P=0.024), but species richness did not differ. Patients with SSc duration less than 6 years had significantly different microbiota compared to long-lasting SSc (unweighted PCoA – q=0.031). Significant variations concerning quantitative and qualitative UniFrac distances (q=0.063, q=0.005) and species richness (q=0.009) were found among patients with diffuse compared to limited SSc. Limited SSc was associated with greater species richness. Taxa plot analysis revealed higher relative abundance of Firmicutes in diffuse disease and of Actinobacteria and Bacteroidetes in limited SSc.

**Conclusions:** Disease duration, disease subset and active DU were associated with shifts in the microbiome of SSc patients. The impact of these changes on disease progression needs further elucidation.

**Figure:**
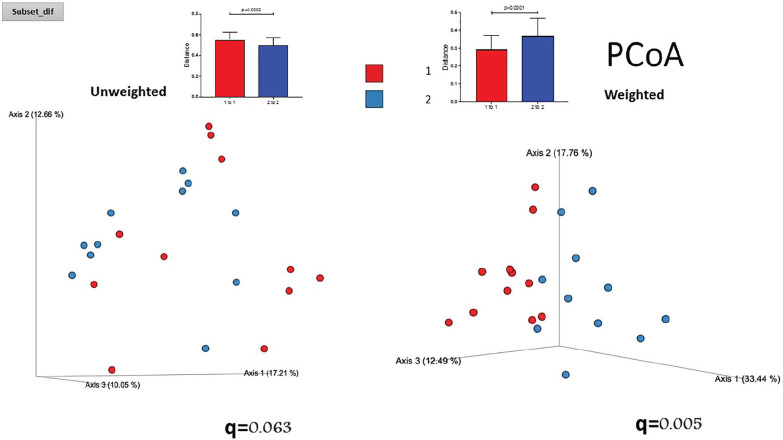


## P.312

## BIOELECTRICAL IMPEDANCE VECTOR ANALYSIS IN A COHORT OF PATIENTS WITH SSC AND IMPLICATION WITH CLINICAL ACTIVITY PARAMETERS

M. Di Battista, A. Rossi, S. Barsotti, A. Monaco, A. Della Rossa, M. Mosca

*Rheumatology Unit - Pisa University Hospital, Pisa, Italy*

**Introduction:** Bioelectrical impedance vector analysis (BIVA) is a common non-invasive method for estimating body composition, which ultimately allows to obtain information on subject’s nutritional status. To date, few data about the use of BIVA have been published in patients with systemic sclerosis (SSc). We used BIVA in a cohort of SSc patients in order to assess their nutritional status and any correlation with the various clinical characteristics of the disease.

**Material and Methods:** Data from 49 consecutive SSc patients [91.8% female; 22 limited, 18 diffuse, 9 sine-scleroderma (ss-SSc); mean age 61.0±12.6 years; mean disease duration 13.4±10.1 years] were collected. Patients underwent BIVA for the assessment of fat mass (FM), free fat mass (FFM), extracellular water (ECW), body cellular mass (BCM) and basal metabolic rate (BMR). Data included anthropometric and clinical features, such as BMI and specific organ involvement with particular attention to gastrointestinal symptoms . Haemoglobin and albumin levels were also collected. Additionally, patients completed UCLA GIT 2.0 questionnaire.

**Results:** Statistical analysis of BIVA results showed that, between the sexes, BMR and BCM were significantly lower in SSc females (p=0.003 and 0.013, respectively), whereas ECW positively correlated with age at the evaluation (r=0.36, p=0.01). Patients with ss-SSc had higher FM values compared to limited and diffuse SSc (p=0.002 and 0.004 respectively). Laboratory tests revealed a positive correlation between the values of haemoglobin and FFM (r=0.498, p<0.001), BMR (r=0.395, p=0.006) and BCM (r=0.376, p=0.009), as well as albumin values did with BMR (r=0.511,p=0.001) and BCM (r=0.595, p<0.001). Conversely, ECW negatively correlated with haemoglobin (r=-0.404, p=0.005). FFM was reduced in patients complaining about early satiety and abdominal distension. A significant difference was found between the presence of digital ulcers and low FFM values in comparison with patients without digital lesions (mean 23.4±3.6% vs 28.6±7.8%; p=0.023). ECW was significantly higher in patients with heart involvement (p=0.02), interstitial lung disease (p=0.017) and pulmonary arterial hypertension (p=0.004); the last two comorbidities were also associated with reduced BCM. No correlations were found with UCLA GIT 2.0 questionnaire. Four patients died during the study: they had significantly lower BMR (p=0.007) and BCM (p=0.01), with an increased ECW as compared to the other patients (mean 72.2±9.3% vs 59.1±7.2%; p=0.002)

**Conclusions:** The parameters obtained with BIVA in SSc patients correlated with serological malnutrition markers (haemoglobin and albumin), with various organ-specific SSc manifestations (cardiopulmonary involvement and digital ulcers) and, more interestingly, with mortality. BIVA could therefore play a role in the prognostic stratification of SSc patients.

## P.313

## SARCOPENIA IN PATIENTS WITH SYSTEMIC SCLEROSIS - PILOT STUDY FROM A SINGLE CENTER

M. Baresic^1^, D. Ljubas Kelecic^2^, B. Karanovic^1^, I. Karas^2^, Z. Krznaric^2^, B. Anic^1^

^1^*Division of Clinical Immunology and Rheumatology, Department of Internal Medicine, University Hospital Center Zagreb, Zagreb, Croatia*, ^2^*Division of Gastroenterology and Rheumatology, Department of Internal Medicine, University Hospital Center Zagreb, Zagreb, Croatia*

**Introduction:** Sarcopenia is a condition characterized by loss of skeletal muscle mass and function and can be age-related (primary sarcopenia) or disease related (secondary sarcopenia). According to some studies sarcopenia can be diagnosed in about 20% of patients with systemic sclerosis.

**Material and Methods:** We conducted a pilot study on 20 patients with systemic sclerosis from a single rheumatology center. We included patients from the Out-patient clinic and Hospital ward. The purpose of this pilot study was to detect the percentage of the patients with sarcopenia and association with nutritional status by using different indices. Nutritional status was assessed with Patient-Generated Subjective Global Assessment (PG-SGA) and anthropometric measurements while body composition was determined with bioelectrical impedance analysis (BIA). Functional status was assessed with hand grip strength (HGS) and 6 minute walk test (6MWT). Sarcopenia was diagnosed according to both, the previous and recently revised European Working Group on Sarcopenia in Older People (EWGSOP) criteria.

**Results:** The results were compared to the characteristics of the patients’ disease - limited vrs. diffuse cutaneous systemic sclerosis, antibody profile, organ affected and the drugs the patients are taking. All of the patients were given written instructions on how to improve their nutritional status.

**Conclusions:** This pilot study will be a basis for further investigations to evaluate the percentage of the patients with systemic sclerosis affected by sarcopenia.

## P.314

## SYSTEMIC SCLEROSIS AND VITAMIN D DEFICIENCY

L. Athanassiou^1^, I. Kostoglou^2^, E. Devetzi^3^, M. Gatsiou^3^, P. Tsakiridis^3^, M. Mavroudi^3^, A. Tzanavari^3^, P. Athanassiou^3^

^1^*First Department of Medicine, Asclepeion Hospital, Voula, Athens, Greece*, ^2^*Department of Endocrinology, Asclepeion Hospital, Voula, Athens, Greece*, ^3^*Department of Rheumatology, St. Paul’s Hospital, Thessaloniki, Greece*

**Introduction:** Vitamin D deficiency has been shown to be associated with autoimmune diseases. In particular, vitamin D deficiency has been found to be associated with disease severity and disease activity in rheumatoid arthritis. Systemic lupus erythematosus has also been found to be associated with vitamin D deficiency, some reports also relating disease activity with vitamin D deficiency. Systemic sclerosis has also been associated with vitamin D deficiency. The aim was to study vitamin D levels in a cohort of systemic sclerosis patients.

**Material and Methods:** In a cohort of 65 systemic sclerosis patients, 58 female and 7 male, aged range 32-82, 62.3±2.3 (mean±SEM) years, 25(OH)D3 levels were measured by radioimmunoassay. 25(OH)D3 levels were also measured in a control group. Statistical evaluation was performed with SPSS.

**Results:** 25(OH)D3 levels in the group of systemic sclerosis patients were 9.35±0.7 ng/ml (mean±SEM) as opposed to those in the control group 25.85±1.59 ng/ml (p<0.001, Student’s t test).

**Conclusions:** Vitamin D deficiency was observed in a cohort of systemic sclerosis patients. Vitamin D deficiency has been reported also by other groups. In particular, Hax et al (J Clin Rheumatol 2019) measured vitamin D levels in a cohort of systemic sclerosis patients in Brazil. They found lower vitamin D levels in the cohort of patients, as opposed to the control group, levels however higher than those observed in our cohort. They also measured cytokine levels, however, they found no association between vitamin D and cytokine levels. Zhang et al (Int J Rheum Dis 2017) measured vitamin D levels in a cohort of Chinese patients with systemic sclerosis. They observed lower vitamin D levels in their cohort as opposed to the control group. It appears, that vitamin D deficiency is observed in systemic sclerosis. Further investigation is required to assess the association of vitamin D deficiency with subgroups of the disease.

## P.315

## FEATURES OF DAMAGE OF THE GASTROINTESTINAL TRACT IN PATIENTS WITH SYSTEMIC SCLERODERMA

V. Aleksandrov^1,2^, L. Shilova^1^, A. Aleksandrov^1,2^, I. Zborovskaya^1,2^

^1^*Volgograd State Medical University, Department of Hospital Therapy, Volgograd, Russia*, ^2^*Research Institute for clinical and experimental rheumatology named after A.B. Zborovsky, Volgograd, Russia*

**Introduction:** Systemic scleroderma (SSc) is an autoimmune disease of connective tissue. Main manifestations of SSc are associated with ischemia and fibrosis of tissues and internal organs. Up to 80% of patients with SSc face various manifestations of the gastrointestinal tract (GIT) lesion. SSc is associated with an increased risk of cancer, especially lung, blood and digestive cancer.

Objective: to study the clinical features and severity of lesions of the gastrointestinal tract in case of SSc.

**Material and Methods:** The study included 83 patients with SSc (97.6% of women, mean age 50.3±11.9, disease duration 8.3±7.1 years). All patients were assigned to laboratory and instrumental studies, including ultrasound of the abdominal organs, fibrogastroduodenoscopy (FGS), and X-ray of the digestive tract. Lesion of the organs of the gastrointestinal tract and liver was diagnosed in 73 (87.9%) patients with SSc. The main complaints of patients were: dysphagia (79.5%), nausea (46.6%), epigastric pain (46.6%), heartburn (39.7%), diarrhea or constipation (38.4%).

**Results:** When conducting an X-ray examination a slowdown in barium passage (more than 10 seconds) was detected in 59% of patients. According to the results of FGS, signs of gastroesophageal reflux were found in 40% of patients. In 3 patients an esophageal erosion was found, and in 2 patients a severe esophageal stenosis accompanied by Barett’s metaplasia was shown.

Ultrasound examination of the abdominal organs in 36 patients (49.3%) revealed signs of damage to the liver and pancreas (hepatomegaly, diffuse changes in the parenchyma of the liver, pancreas, uneven contours, heterogeneity of the structure, pseudocysts).

3 patients (4.1%) had jaundice; laboratory tests (blood amylase, transaminases) were increased in 32 people (43.8%); a coprological examination revealed signs of steatorrhea and creatorrhea in 21 (28.8%) patients.

The presence of gastrointestinal and liver malignancies was found in 5 patients with SSc. SSc was detected before cancer diagnosis in 2 cases (esophageal cancer and liver cancer), but after oncopathology SSc was detected in 3 cases (esophageal cancer, stomach cancer and liver cancer). Moreover, both patients with primary diagnosis of SSc were under the age of 40, and all patients with primary diagnosis of oncopathology were over the age of 50 years.

**Conclusions:** The study of gastrointestinal manifestations in the context of malignant neoplasms can improve the understanding of the pathogenesis of SSc. New therapeutic approaches, taking into account the involvement of the digestive tract, as well as monitoring and reducing risk factors, can affect the development of malignant processes in SSc.

## 11. Gender Related Issues

## P.316

## PREGNANCY OUTCOME IN SYSTEMIC SCLEROSIS; IS THERE ANY CORRELATION BASED ON TOPO I, DISEASE SUBTYPES AND DISEASE ONSET?

H. Poormoghim^1^, M. Shakerian^1^, E. Andalib^1^, Z. Zahra Raufi^1^, A. Jalali^2^

^1^ *Firoozgar hospital .Iran University of Medical Sciences, Tehran, Iran*

^5^Tehran University of Medical Sciences, Tehran, Iran

**Introduction:** To determine correlation between disease subtypes, onset and presence of anti-topoisomerase I on pregnancy outcome in women with systemic sclerosis

**Material and Methods:** One hundred and twenty-nine female patients whose onset of first disease symptoms began before age 45 years from those referred to Firoozgar outpatient scleroderma clinic were asked about pregnancy and its outcome. Demographic and disease data were extracted from our scleroderma data base. Systemic sclerosis was diagnosed according to ACR/EULAR and classified according to LeRoy criteria.

**Results:** Total of 109 (84%) pregnancy reported, 52 (78.8%) in dcSSc, 57(90.5%) in lcSSc. Twenty women (15.5%) never had pregnancy (14 dcSSc, 6 lcSSc). Hundred and two (93.6%) had successful pregnancy. Total reported pregnancy was 278 (mean of 2.55 pregnancy/woman), mean pregnancy rate in dcSSc and lcSSc were 2.36 % vs 2.71% respectively. Women had more live delivery before disease onset [70/71 (98%) vs 12/49 (67%), OR 0.02 (0.003- 0.25), p=0.001]. Number of successful pregnancies before disease onset versus after were 33 (67%),191 (83.3%), [OR 0.4 (0.20-0.81), p=0.01]. Abortion rate, after and before disease onset showed no significant difference p= 0.94 whereas, spontaneous abortion was more prevalent after disease onset, [12/49 (24.4%) vs 22/229 (9.6%), p=0.005, OR 3.05 (1.39-6.69)]. Preterm delivery reported in 9/71 (12.6%) and 8/49 (16.3%) before and after disease onset [OR: 5.51(1.72-17.63), p=0.004]. Preterm delivery before and after disease onset were [10/229 (4.3%), 8/18 (44.4%), OR: 4.76 (1.73-13.08), P=0.002]. Cesarean section, Neonatal death and very low birth weight babies were more prevalent in deliveries after versus before disease onset, 18 (36.7%) vs 48 (20.9%) [ OR: 2.18 (CI:1.12-4.24), P=0.02], 1/49 (2%) vs 3/229 (1.3%), (P=0.57) and 3/49 (6%) vs 2/229 (1%), [7.40 (1.20 - 45.55), p=0.03] respectively. Disease subtypes and anti-topoisomerase I show no correlation with pregnancy complications after disease onset.

**Conclusions:** The result of the study showed successful pregnancy after disease onset occurred in two-third of SSc pregnancies. In Pregnancies after Systemic sclerosis onset rates of preterm delivery, pregnancy loss, very low-birth weight increased. Disease subtypes and anti-topo abs have no effect on pregnancy outcome after disease onset.

## P.317

## CLINICAL CHARACTERISTICS OF MALE PATIENTS WITH SYSTEMIC SCLEROSIS; RESULTS FROM IRANIAN SYSTEMIC SCLEROSIS REGISTRY

A. Javinani, F. Gharibdoost, A.R. Jamshidi, Z. Tamartash, M. Mahmoudi, H. Kavosi

*Rheumatology Research Center, Tehran, Iran*

**Introduction:** Systemic sclerosis (SSc) is an autoimmune disorder that frequently affects females. However, anecdotal results exist regarding the more severity of SSc clinical manifestation between two genders. This study was undertaken to investigate the clinical features of male SSc patients.

**Material and Methods:** This study was a retrospective investigation from Iranian SSc registry. The clinical manifestations, laboratory parameters, and serologic data were compared between male and female patients. Variables were compared by Chi-square and independent sample t-test.

**Results:** Among 660 studied patients, 93 cases (14.1%) were male with the female to male ratio of 6 to 1. Male SSc patients had the mean age at Raynaud’s phenomenon of 34.68 ± 14.30 years that was similar to female cases (P-value: 0.787). Male patients had a higher percentage of diffuse subtype of SSc with the prevalence of 73.4% in comparison to 57.2% (P-value: 0.007). The mean modified Rodnan skin score was 19.60 ± 10.11 in male cases that was significantly higher than female patients (P-value: <0.001). The prevalence of imaging-proven interstitial lung disease was 59.7% with the mean predicted forced vital capacity was 75.83 ± 21.26% among male SSc patients that was not statistically different from females (P-value: 0.466). The mean predicted pulmonary artery pressure was 30.28 ± 10.70 and 29.30 ± 9.10 mmHg among male and female patients, respectively (P-value: 0.425). The scleroderma renal crisis was reported in 4 male patients (4.6%) that was higher than female counterparts (P-value: 0.003). The prevalence of gastrointestinal manifestations was similar in both gender groups (P-value: 0.377). The previous history of digital ulcer and telangiectasia were present in 44.3 and 46.2% of male patients, respectively. The prevalence of arthritis, inflammatory myopathy and calcinosis cutis were not higher in male patients. Nineteen percent of male cases have reported erectile dysfunction. Anti-topoisomerase and anti-centromere antibodies were positive in 54.2 and 2.0% of male cases without significant difference in comparison to female patients.

**Conclusions:** This study has shown that male patients with SSc have a higher percentage of diffuse cutaneous SSc subtype with higher modified Rodnan skin score. Additionally, the prevalence of scleroderma renal crisis is also higher in this population. However, the severity of other manifestations of SSc seems not to be related to gender.

## P.318

## OBSTETRIC COMPLICATIONS PRIOR TO SYSTEMIC SCLEROSIS DIAGNOSIS

M. Chung^1^, H. Zhao^2^, K. Kolstad^1^, P. Manwani^2^, M. Dontsi^3^, D. Postlethwaite^3^, J. Simard^4^, L. Chung^1,5^

^1^*Division of Immunology/Rheumatology, Stanford University School of Medicine, Palo Alto, USA*, ^2^*Kaiser Permanente Santa Clara Internal Medicine Residency, Santa Clara, USA*, ^3^*Division of Research, Kaiser Permanente Northern California, Oakland, USA*, ^4^*Division of Clinical Epidemiology, Department of Health Research and Policy, Stanford University School of Medicine, Stanford, USA*, ^5^*Division of Immunology/Rheumatology, VA Palo Alto Health Care System, Palo Alto, USA*

**Introduction:** Vasculopathy and immune dysfunction likely precede the diagnosis of systemic sclerosis (SSc) as evidenced by the presence of Raynaud’s phenomenon and autoantibodies in the majority of patients prior to diagnosis. Poor obstetric outcomes may reflect underlying vascular and immune system abnormalities in patients who later develop SSc. Many obstetric complications (such as IUGR, pre-eclampsia, and eclampsia) are secondary to vascular abnormalities and abnormal placentation. The purpose of this study was to investigate whether obstetric complications prior to an eventual SSc diagnosis are more common compared to the general obstetric population.

**Material and Methods:** A retrospective case-control study was conducted among Kaiser Permanente Northern California (KPNC) adults with an incident diagnosis of SSc by a rheumatologist from 2007-2017, confirmed by chart review to fulfill 2013 ACR-EULAR classification criteria. The KPNC Infant Cohort Live Birth Database identified women with pregnancies prior to SSc diagnosis. Non-SSc controls were matched 10:1 by maternal age and year of delivery. Exposures of interest included hypertensive disorders of pregnancy (including pre-eclampsia, eclampsia, and gestational hypertension), premature rupture of membranes (PROM), intrauterine growth restriction (IUGR), maternal infections, neonatal intensive care unit (NICU) admission, and preterm birth. Fischer’s exact tests were used to compare categorical variables. Univariate conditional logistic regression models estimated the odds ratio and corresponding 95% confidence intervals for the outcome SSc. Multivariable analysis correcting for race was performed.

**Results:** Seventeen SSc cases and 170 non-SSc matched controls were identified. Mean maternal age at delivery was 33±7 years. Mean time from delivery to SSc diagnosis was 2±2 years, with 13/17 (76.5%) developing limited cutaneous disease. Compared to controls, SSc cases had fewer Whites (17.7% vs. 50.6%), but more Hispanics (41.2% vs. 18.8%) and African-Americans (11.8% vs. 2.4%). Rates of obstetric complications were non-significantly higher in women with an eventual SSc diagnosis compared to matched controls, including hypertensive disorders (23.5% vs. 14.7%, p=0.31), PROM (11.8% vs. 4.1%, p=0.19), IUGR (5.9% vs 1.8%, p=0.32), maternal infection (29.4% vs. 14.1%, p=0.15), NICU admissions (23.5% vs. 7.7%, p=0.053), and preterm birth (29.4% vs. 21.8%, p=0.54). On regression analyses, cases had significantly increased odds of delivering infants requiring NICU admission compared to controls, and this persisted after correcting for race (Table 1).

**Conclusions:** Women who eventually develop SSc had trends towards increased pregnancy complications, particularly NICU admissions, before overt diagnosis. Larger studies are needed to validate these findings.

## P.319

## ARE SILICON IMPLANTS ASSOCIATED SYSTEMIC SCLEROSIS (SSC) AND IDIOPATHIC SSC DIFFERENT ENTITIES?

Y. Braun-Moscovici^1^, M. Braun^2^, D. Markovits^1^, K. Toledano^1^, Y. Tavor^1^, A. Rozin^1^, K. Dolnikov^1^, N. Finkelstein-Yeshurun^1^, S. Giries^1^, M.A. Nahir^1^, A. Balbir-Gurman^1^

^1^*B Shine Rheumatology Institute, Rambam Health Care Campus, Rappaport Faculty of Medicine, Technion, Haifa, Israel*, ^2^*Liver Institute, Beilinson Hospital, Sackler School of Medicine, Tel Aviv University, Petach Tikva, Israel*

**Introduction:** Silicone breast implants (SBI) seem to be associated with higher likelihood of autoimmune/ rheumatic disorders diagnosis including systemic sclerosis (SSc).

Our aims were to assess the prevalence of breast implants in a well characterized SSc cohort followed at our tertiary center and to define charcteristics that might differ between SSc patients with implants and those without.

**Material and Methods:** A retrospective analysis of a prospectively maintained database was performed regarding the prevalence of breast implants and the disease characteristics. Female patients aged 20 to 55y (215 patients) followed at our center between 2003-2018 were included. SBI cases were identified and demographic, clinical and laboratory data were obtained. Each SBI patient was paired with 3 controls matched for age, disease duration and type of disease. We used descriptive statistics, multivariate comparisons and multivariate analysis for correlations.

**Results:** Eleven pts with SBI were found, 6 with diffuse (mean(SD) age 40.3(8.4), mean(SD) disease duration 3.7(2.3) y, mean(SD) mRSS 16.7(9.3), and 5 with limited SSC (mean(SD) age 37.8(4.5), mean(SD) disease duration 9.4(4.6)y, mean(SD) mRSS 3(1.4)). The prevalence of SBI in the 20 to 55 y cohort was 5%, similar to the estimated prevalence in the general population. All had their implants instilled before the diagnosis of SSC (median 10y, range 4-13y). Four pts were first diagnosed with SSC within a year from discovering silicone leakage. RNA polymerase 3 was positive in 3 patients, Scl70 in 3 others, anticentromere in 1 patient and 4 others were positive only for antinuclear antibody. There were no statistically significant differences in the prevalence of digital ulcers, gastrointestinal involvement, skin score or the serologic profiles between the two groups. However, none of the SBI pts developed interstitial lung involvement, compared to 72% of the matched diffuse patients (p=0.001). The mean(SD) vital capacity of the patients with SBI and diffuse disease was higher compared to the matched group (84.7(11.5)% versus 67.7(23), p=0.09) and so was the DLCO/VA (70.2(21)% versus 53.6(16.7)%, p=0.05) There were neither renal crises in the SBI group (compared to 5 cases in the matched group) nor cardiac involvement.

**Conclusions:** The temporal relation and the different clinical features might generate a theory that silicone might induce a particular type of SSC, as the idea that silicone protects against lung involvement seems unreasonable. We plan a further prospective study.

**Figure:**
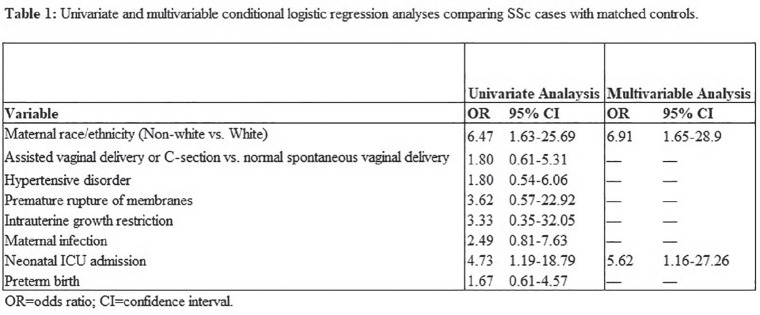


## P.320

## FAVORABLE OUTCOME OF PLANNED PREGNANCIES IN SYSTEMIC SCLEROSIS PATIENTS DURING STABLE DISEASE

Y. Braun-Moscovici, D. Markovits, V. Shataylo, A. Avshalom-Mashiah, M.A. Nahir, A. Balbir-Gurman

*Rambam Health Care Campus, B Shine Rheumatology Institute, Rappaport Faculty of Medicine, Technion, Haifa, Israel*

**Introduction:** Studies evaluating pregnancy outcomes in systemic sclerosis (SSc) are limited. SSc was associated with maternal complications and adverse neonatal outcomes.

Our aim was to assess the impact of stable versus active SSc disease on the maternal and neonatal outcomes of pregnancies of patients followed at our center.

**Material and Methods:** The charts of 354 SSc female patients followed during the years 2003-2018 were reviewed. Data regarding clinical and laboratory features, number of pregnancies close to SSc diagnosis, maternal and neonatal outcomes were analyzed. We divided the patients in 2 groups: active (group 1) versus stable disease (group 2). Active disease was defined as new-onset (less than 5 years), deterioration of skin or visceral involvement or severe vasculopathy.

**Results:** 39 pregnancies occurred in 23 SSc patients (9 diffuse, mean (SD) age at diagnosis 28.9(7.5) years) with 37 live born children. Antitopoisomerase was positive in 10 patients, RNA polymerase3 in one patient, anticentromere in 6 patients and 6 patients were positive for ANA only.

Group 1 (active disease): 11 patients (8 diffuse), 18 pregnancies (47%). SSc was first diagnosed during pregnancy in 2 patients. Median disease duration in the other patients was 2.5 years. In all of them SSc aggravated during the pregnancy or shortly after the delivery, mainly skin involvement and mild lung and heart changes in the diffuse SSc patients and severe vasculopathy in the limited SSc patients. One diffuse patient developed renal crisis a year after the delivery. Another patient died 2 years after 2nd delivery. Pre-eclampsia occurred in 2 patients and hypertensive disease of pregnancy in another (27%). The only twin pregnancy ended at week 24 with rupture of the uterus and foetal death. 17 children were born, 12(70%) with IUGR/ low birth weight. All developed normally afterwards.

Group 2 (stable disease): 15 patients, including 3 from group1, in whom the disease stabilized (4 diffuse), 21 pregnancies. All pregnancies were planned. Three of the pregnancies were after IVF. The patients had a close rheumatologic and gynecologic follow-up. The disease remained stable in all patients, during the pregnancy and afterwards. Pre-eclampsia occurred in 1 patient (7%). Ten out of 21 newborns had low birth weight (50%) with normal development afterwards.

**Conclusions:** Active maternal disease during pregnancy poses increased risk to SSc aggravation. The maternal and neonatal outcomes in planned pregnancy during stable disease are favorable. IUGR/low birth weight is common among neonates even in stable disease.

## 12. Recent Advances in Therapy (case series for new treatment)

## P.321

## THE EFFICACY AND SAFETY OF RITUXIMAB IN 27 CASES OF TREATMENT RESISTANT SYSTEMIC SCLEROSIS WITH SEVERE DISEASE: FAVORABLE EFFECT ON DISEASE ACTIVITY

Y. Yalcinkaya, B. Artim Esen, S. Amikishiyev, N. Aliyeva, A. Gul, L. Ocal, M. Inanc

*Istanbul University, Istanbul Medical Faculty, Depatment of Internal Medicine, Division of Rheumatology, Istanbul, Turkey*

**Introduction:** B-cell targeted therapy with anti-CD20 Rituximab(RTX), is widely available, reports from case series are encouraging as a a rescue therapy and might have an improving effect on organ involvement insystemic sclerosis(SSc). We aimed to analyze the efficacy and safety of RTX courses in patients with severe SSc who were refractory to standard immunosuppressive treatment retrospectively.

**Material and Methods:** Twenty-seven SSc patients fulfilling ACR/EULAR classification criteria (2013) who received RTX treatment due to active disease despite treatment with immunosuppresives were analyzed. Disease activity was evaluated by using EScSG/EUSTAR activity scores prior to and after RTX treatment. Disease severity was also assessed at baseline by Medsger’s index.

**Results:** The demographics and characteristics of SSc patients were as follows: the median age of 50 (30-70), duration of Raynaud’s 10 (3-26) and non-Raynaud symptom 8.5 (3-18) years and summarised in table-1. RTX was given as a single cycle (2 infusions of 1000 mg) in 12 cases, 2 cyles in 5 cases, >3 cyles in 10 cases. DMARDs were prescribed in 19 (73%) patients (14 MMF, 5 MTX) concomitantly with RTX. The main RTX indications were skin and lung involvement (n=9), skin and arthritis (n=6), skin(n=5), lung (n=3), myositis (n=2), cardiac involvement (n=1) and digital vasculopathy (n=1). Medsger severity score was 7.39±3.091(3-13) at baseline.

Disease activity and severity scores prior to and after RTX were summarized table-2. Disease activity scores were improved after RTX in patients who had a median follow-up period of 1 year (0,5-5 years). After RTX treatment, according to EscSG /EUSTAR scores 13 (%46.2) and 10 (%34.6) patients out of 26 were assessed as inactive.

There were severe infections in 4 patients (Pneumonia in 2, infected digital ulcers in 2) and an episode of sinusitis in one during treatment period. One patient deceased because of pneumonia and sepsis after the first cycle of RTX.

**Conclusions:** In our SSc cohort, RTX treatment was used in severe patients, who had predominantly diffuse cutaneous disease with lung and joint involvements, severe vasculopathy and anti-Scl-70 positivity. Concomitant DMARDs were used in three-forth of the patients in addition to RTX cycles. Disease activity scores were shown to be improved after RTX and 37-48% of the cases were assessed as inactive. Serious infections like pneumonia and infected digital ulcers were observed in 14,8% of cases during the follow-up. The addition of RTX treatment is effective in severe patients with active disease despite immunosuppressive therapy, although severe infections may be observed.

**Figure:**
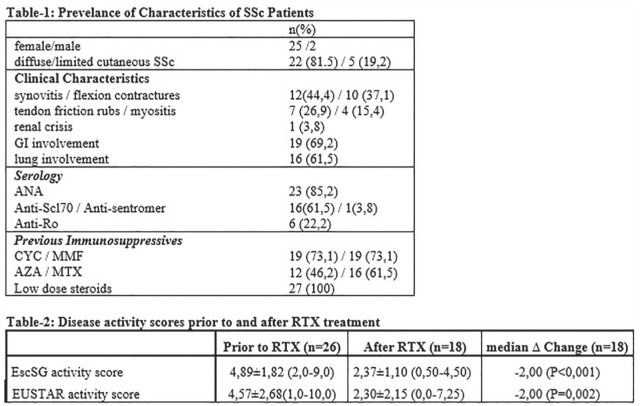


## P.322

## PREDICTIVE FACTORS FOR TREATMENT RELATED MORTALITY AND EVENT-FREE SURVIVAL AFTER AUTOLOGOUS HEMATOPOIETIC STEM CELL TRANSPLANTATION FOR SYSTEMIC SCLEROSIS: RESULTS OF A LONG TERM FOLLOW-UP MULTI-CENT

M. Vonk^1^, S. van Bijnen^1^, M. Boonstra^3^, E. van den Ende^1^, L. Kroft^3^, B. Geurts^1^, M. Snoeren^1^, A. Schouffoer^3^, J. Spierings^2^, J. van Laar^2^, T. Huizinga^3^, A. Voskuyl^4^, W. van der Velden^1^, F. van den Hoogen^1^, J. de Vries-Bouwstra^3^

^1^*Radboud University Medical Centre, Nijmegen, The Netherlands*, ^2^*Utrecht University Medical Centre, Utrecht, The Netherlands*, ^3^*Leiden University Medical Centre, Leiden, The Netherlands*, ^4^*Amsterdam University Medical Centre, Amsterdam, The Netherlands*

**Introduction:** Autologous hematopoietic stemcell transplantation (HSCT) has shown to improve survival of SSc patients with poor prognosis, but is hampered by treatment related mortality (TRM). Better selection of eligible patients could improve outcomes.

Objectives: 1. To evaluate event-free survival, TRM and response after HSCT in SSc and 2. To explore patient characteristics that associate with events

**Material and Methods:** Data on event-free survival of all patients treated with HSCT for SSc in the Netherlands between 1998 and 2017, performed as previously described were collected. Baseline characteristics including hematopoietic cell transplant comorbidity index (HCT-CI) and left ventricular ejection fraction (LVEF) and data for skin involvement (mRSS), pulmonary function (forced vital capacity % predicted (FVC% pred), diffusion capacity % predicted (DLCO% pred)) and staging for interstitial lung disease on HRCT assessed according Goh were collected at baseline and at 1,2, 5 years ; data for events and death were collected until end of study. All deaths were discussed in a consensus meeting and classified as TRM, SSc progression or other. Event-free survival was defined as described before. Relapse was defined as an increase in mRSS of > 25% and > 5 points compared to the lowest value since HSCT, or either a >10% decline in FVC% pred, or >5 – <10% decline in FVC% pred and >15% decline in DLCO% pred or initiation of immunosuppression. The association between event-free survival and baseline characteristics was examined by univariate Cox regression analysis. Factors with a significant association were entered in a multivariate analysis on imputed missing data.

**Results:** In total 92 patients were included. Event-free survival estimates at 5, 10 and 15 years were 0.79, 0.79 and 0.68 respectively. Twenty deaths occurred, which were classified as TRM (n=10, 11%), SSc progression (n=4, 4%) and other (n=6 7%). Relapse occurred in 22 patients in the first 5 years: skin relapse in 5, lung relapse in 11 and initiation of immunosuppression in 12 (in 7 patients without skin or lung relapse). FVC, DLCO, mRSS and Goh scores improved significantly over time in patients surviving the first 5 years (n=75) (Figure 1). Events were independently associated with male sex, LVEF < 50% and older age.

**Conclusions:** Event-free survival at 10 years after HSCT for SSc was 79%; male sex, lower LVEF and older age were identified as independent risk factors for events. Our data confirms efficacy of HSCT in improving survival and skin and lung involvement.

## P.323

## EFFECTIVENESS OF RITUXIMAB IN PATIENTS WITH EARLY DIFFUSE CUTANEOUS SYSTEMIC SCLEROSIS. A MULTICENTER ANALYSIS

I. Vázquez Gómez^1^, J. Lluch Pons^2^, F.J. Narváez García^2^, M. Aguilar Zamora^1^, L. Montolio Chiva^1^, A.V. Orenes Vera^1^, E. Flores Fernández^1^, A. Martínez Ferrer^1^, E. Valls Pascual^1^, D. Ybáñez García^1^, I. Torner Hernández^1^, V. Núñez Monje^1^, A. Sendra García^3^, J.J. Alegre Sancho^1^

^1^*Servicio de Reumatología, Hospital Universitario Dr. Peset, Valencia, Spain*, ^2^*Servicio de Reumatología. Hospital Universitari de Bellvitge, Barcelona, Spain*, ^3^*(FISABIO)-Servicio de Reumatología y Farmacia, Hospital Universitario Dr. Peset, Valencia, Spain*

**Introduction:** Rituximab (RTX) is effective in improving skin affection in patients with diffuse cutaneous systemic sclerosis (DcSSc). However, there are few data on early use of this drug. The objetive of this study is to evaluate RTX effectiveness for skin disease in patients with DcSSc of less than 3 years of evolution.

**Material and Methods:** Multicenter, observational and retrospective study. Patients with DcSSc starting RTX within 3 years since first non-Raynaud symptom were recruited. Demographic variables, time of disease duration at the beginning of RTX, immune pattern and time on RTX treatment were collected. Effectiveness was defined as modified Rodnan skin score (mRSS) improvement. Evaluations were done by the same experienced rheumatologist. Patients subjective perception of skin hardening and/or tightness was evaluated. mRSS changes from baseline to 6 and 12 months after RTX beginning and, later on, to the last available observation were analysed using Wilcoxon test. Statistical analysis was performed with SPSS 20.0.

**Results:** 11 patients (8 women) were recruited from 2 university hospitals. Median age was 48 years (IQR 22). Median time since diagnosis to RTX beginning was 12 months (IQR 8). 5, 3 and 2 patients presented ATA +, RNPIII + and Ro-52 +, respectively. Median duration of RTX treatment was 12 months (IQR 68). Median baseline mRSS was 15.5 (IQR 18). Median mRSS after 6 and 12 months of RTX treatment and at last available mRSS evaluation was 15 (IQR 13), 14.5 (IQR 13) and 11 (IQR 16), respectively. mRSS showed statistically significant improvement at 6 (29%, IQR 37) and 12 months of RTX treatment (35%, IQR 34) and, thereafter, at last available observation (39%, IQR 51), compared to basal mRSS. Most patients reported subjective improvement at 6 (9 of 10 patients) and 12 months (6 of 7), and at last available evaluation (6 of 8); all other patients reported stability.

**Conclusions:** In our experience, patients with DcSSc seem to benefit of early RTX treatment. Improvement may be seen as early as 6 months and seems to reach a plateau at 12 months.

**Figure:**
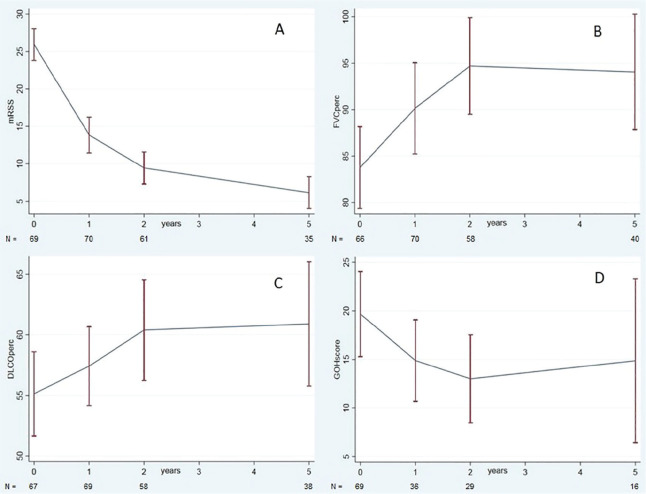


## P.324

## AMINAPHTONE EFFICACY IS NOT INFLUENCED BY DIFFERENT PHARMACOLOGICAL BACKGROUNDS IN SYSTEMIC SCLEROSIS PATIENTS

A. Sulli, E. Gotelli, F. Goegan, B. Ruaro, C. Pizzorni, S. Paolino, M. Cutolo

*Research Laboratory and Academic Division of Clinical Rheumatology, Department Internal Medicine, University of Genova, Genova, Italy*

**Introduction:** In a recent open feasibility study, we demonstrated that aminaphtone can improve clinical symptoms of Raynaud’s phenomenon (RP), as well as peripheral blood perfusion (BP), in patients with either primary or secondary RP, with sustained efficacy up to six months (1). The aim of this study was to evaluate the influence of different pharmacological backgrounds on aminaphtone efficacy in patients with primary or secondary Raynaud’s phenomenon (RP) to systemic sclerosis (SSc) during a six-month follow up.

**Material and Methods:** Forty-six patients with active RP were enrolled during routine clinical assessment (11 primary RP, mean age 49±19 SD years, mean RP duration 6±3 years; and 35 secondary RP to systemic sclerosis (SSc), mean age 61±17 years, mean RP duration 11±9 years). Aminaphtone was orally administered 75 mg twice daily in addition to current standard treatments, and all patients were on a stable drug regimen since at least two months, which remained unmodified during the follow-up. All patients were taking aspirin. Six groups of treatment backgrounds were identified: 1) hydroxychloroquine (2 patients); 2) methotrexate (3 patients); 3) colchicine (5 patients); 4) cyclosporine A (6 patients); 5) mycophenolate (6 patients); 6) proton-pomp inhibitors (12 patients); 7) no further treatments (12 patients). Raynaud’s condition score (RCS) and both frequency and duration of Raynaud’s attacks were assessed at the same time. Blood perfusion was measured by Laser Speckle Contrast Analysis (LASCA) (2) at the level of fingertip, periungual areas, dorsum and palm of hands, and face at baseline (T0), after one (T1), four (T4), twelve (T12) and twentyfour (T24) weeks of treatment. Statistical analysis was performed by non-parametric tests.

**Results:** During aminaphtone treatment, an improvement of RP clinical symptoms, as well as a progressive increase of BP in all skin areas, were observed from T0 to T12 in almost all above reported groups of RP patients with different treatments backgrounds (p<0.05). There was no statistically significant difference between the seven groups of patients concerning both skin BP and Raynaud’s improvement at different times (p=0.60).

**Conclusions:** This study demonstrates that different pharmacological treatment backgrounds do not influence the increase of skin BP as well as the improvement of RP clinical symptoms during aminaphtone administration in a cohort of patients with either primary RP or secondary RP to SSc.

**References**

1. Ruaro B, et al. Front Pharmacol 2019; 10:293.

2. Ruaro B, et al. Ann Rheum Dis. 2014;73:1181-5.

## P.325

## COMBINATION THERAPY OF RITUXIMAB AND MYCOPHENOLATE MOFETIL FOR SEVERE SYSTEMIC SCLEROSIS – SAFETY AND EFFICACY

D. Rimar, A. Silawi, I. Rosner, M. Rozenbaum, K. Kali, N. Boulman, G. Slobodin

*Rheumatology unit Bnai Zion Medical center, Haifa, Israel*

**Introduction:** The optimal treatment for systemic sclerosis-associated interstitial lung disease (SSc-ILD) is not known. Based on the best available evidence Mycophenolate mofetil (MMF) is preferred as initial therapy over cyclophosphamide due to a better safety profile and comparable efficacy. Recently rituximab has become a commonly used second line therapy. We describe herein our experience with upfront combination of rituximab and MMF therapy for patients with severe diffuse SSc with lung disease.

**Material and Methods:** SSc Patients treated in our daycare center were retrospectively evaluated. All patients were diagnosed according to EULAR 2013 classification criteria. Ct scans were evaluated by a radiologist and percent of fibrosis before and after one year of treatment was evaluated. All patients had modified Rodnan skin score (mRSS) evaluated by one experienced rheumatologist at baseline and after a year of treatment. All patients received rituximab 2000 mg every 6 month and MMF 2000 mg daily. All patients received iloprost. One patient received also IVIG and two patients received Lanabasum.

**Results:** Six women and four men were evaluated. Five patients were seropositive for RNA POL III four patients positive for anti-topoisomerase I and one positive only for anti PM-SCL. Five patients had non specific interstitial pneumonia pattern, one with usual interstitial pneumonia, one with organizing pneumonia and 3 with ground glass opacities. Lung fibrosis as evaluated on CT scans reduced from 20.8±23.4% to 10.5±16.4%. Forced vital capacity improved from 66.4±17.3% predicted to 72.8±17.01. DLCO improved from 58.1±19% predicted to 63.2±19.2%. The mRSS reduced from 23.6±14.5 to 9±7.9. There were no severe infections requiring IV antibiotics. One patient developed breast carcinoma. There were no hospitalizations or deaths.

**Conclusions:** Combination therapy of rituximab and MMF is effective and safe in patients with diffuse SSc with respect to skin and lung fibrosis that should be evaluated prospectively in larger cohorts.

## P.326

## FIRST CASE OF AUTOLOGOUS STEM CELL TRANSPLANTATION FOR SYSTEMIC SCLEROSIS IN ESTONIA

E. Pärsik^1^, E.-K. Raussi^1^, D. Loigom^2^, K. Palk^2^, E. Ojassalu^1^, P. Tuvik^1^, Ä. Roose^3^

^1^*North Estonia Medical Centre, Dept of Rheumatology, Tallinn, Estonia*, ^2^*North Estonia Medical Centre, Dept of Oncology and Hematology, Tallinn, Estonia*, ^3^*North Estonia Medical Centre, Dept of Radiology, Tallinn, Estonia*

**Introduction:** Systemic sclerosis (SSci) is a severe chronic disease with heterogeneous clinical presentation and organ manifestations. There is no optimal treatment at present. Autologous stem cell transplantation (ASCT) has shown promising results and can be lifesaving for patients with rapid skin and lung involvement progression.

**Material and Methods:** We present a case of 63yo woman diagnosed with SScl in 2015. She presented with Raynaud’s syndrome, skin induration (modified Rodnan skin score (mRSS) 17/51), teleangiectasiae and arthalgia. Blood test were markable for elevated ERS and ANA positivity 1:1000 ( fibrillarin and Ro-52 autoantibodies). Initial treatment included nifedipine, cyclosporine A and NSAIDs.

In February 2016 exertion dyspnoea developed. DLCO was 63,8%, FVC 3.7l, CT revealed mild NSIP. In March 2017 mRSS progressed to 28/51 and CyA was switched to azathioprine. From June to December 2017 she participated in clinical trial ACT14604 ( SAR156597/placebo) with favourable results.

In March 2018 dyspnoea worsened, walking capacity reduced from 8 to 2km. mRSS was 17/51. DLCO was 53%, FVC 4l, CT showed progression of lung involvement. IV Cyclophosphamide (7 infusions (7105mg) until August 2018) was ineffective: mRSS 17/51, lesions developed in 3 fingers, DLCO 52,5%, FCV 3.12l, CT showed more ground glass opacities.

The patient consented with ASCT. EchoCG was normal (EF 67%), heart MRI revealed mild interstitial fibrosis. Blood tests showed mild aneamia (101g/l), elevated ESR (40mm/h), otherwise normal (including TrT, proBNP and screen for infections).

In Nov/Dec 2018 stem cells were mobilized and collected. The ASCT was performed on 15January2019 according to the protocol (CTX + rATG). The post-tranplantation period was unremarkable with no complications. The patient was discharged on 02February2019 and remains on IVIG prophylaxis until now.

**Results:** mRSS improved rapidly to 5/51 in 2weeks post-transplantation. In March 2019 DLCO was 57%, FVC 4,0. Ground glass opacities were resolving.

In September 2019 the patient was able to walk 5kms in 50min. mRss is 1. DLCO is 49%, FVC 3,6 CT showed further improvement.

**Conclusions:** The first case of ASCT for SScl in Estonia performed on 15January2019 in North Estonia Medical Centre can be considered successful with no short-term complications and fast resolvement of skin and lung involvement. Further follow-up is needed for long-term resul

## P.327

## RESPONSIVENESS TO CHANGE OF THE MODIFIED RODNAN SKIN SCORE IN A PHASE I/II DOUBLE-BLIND RANDOMIZED PLACEBO-CONTROLLED TRIAL

V. Nagaraja^1^, E. Bush^1^, R. Lafyatis^2^, D. Khanna^1^

^1^*University of Michigan, Ann Arbor, USA*, ^2^*University of Pittsburgh, Pittsburgh, USA*

**Introduction:** Modified Rodnan skin score (mRSS) is used as primary and secondary outcome measure in different trials of diffuse cutaneous systemic sclerosis (dcSSc). As part of a Phase I/II trial assessing the safety of tofacitinib 5 mg twice a day versus placebo in dcSSc (clinicaltrials.gov NCT032740762), we assessed the performance of 3 different methods of scoring the modified Rodnan skin score (mRSS).

**Material and Methods:** A 6-month, double-blind, randomized placebo-controlled trial was conducted in dcSSc with disease duration of less than or equal to 60 months (defined as first non Raynaud’s phenomenon) and mRSS between 10 and 45 units. Efficacy end point included the change in the mRSS at 6 months. Each anatomical area was scored as Global Average score where the examiner takes average of the area; Maximum score where an examiner scores the area according to the most severe local involvement; and the Representative area where the examiner assigns a score that is most representative of the area. Responsiveness to change was evaluated using the effect size (ES) and standardized response mean (SRM). Both indices are ratios of observed change to a measure of variance (also known as signal to noise). For two indices, the numerator is the mean change from the baseline to Month 6 for mRSS and the denominators are the standard deviation at baseline (ES) and the standard deviation of change (SRM). Cohen’s rule-of-thumb for interpreting responsiveness to change was applied to determine the magnitude of change where 0.20-0.49 represents a small change, 0.50-0.79 a medium change, and 0.80 or greater a large change.

**Results:** 15 participants were randomized (2:1; 10 to TOFA and 5 to PLA) and formed the mITT group; 10 (100%) and 4 (80%) completed the 6-month treatment period in TOFA and PLA groups, respectively. The mean baseline scores were similar in the Global Average and Representative groups and higher in the Maximum group (Table 1). Using the ES, the magnitude of responsiveness was similar in all 3 groups (Medium change). Using the SRM, the magnitude of change was numerically greater in the Representative and Maximum groups (Large change) vs. Global Average (Medium change).

**Conclusions:** In a small trial of 15 participants, all 3 methods to score the mRSS yielded similar magnitude of responsiveness to change. The mRSS was conducted by experienced researchers in this trial and these results should be validated in a larger trial with multiple centers.

## P.328

## COMPARISON OF SAFETY AND EFFICACY BETWEEN DIFFERENT BIOLOGIC THERAPIES IN SYSTEMIC SCLEROSIS

D. Lobo-Prat, A.M. Millán, L. Sainz, H.S. Park, B.P. Magallares, P. Moya, S. Fernández, S. Ros, C. Díaz-Torné^1^, A.M. Laiz, A. García-Guillén, S.R. Jeria, H. Corominas, I. Castellví

*Hospital Universitari de la Santa Creu i Sant Pau - Department of Rheumatology, Barcelona, Spain*

**Introduction:** Systemic sclerosis (SSc) causes vascular, fibrotic and inflammatory damage. Encouraging results have been found in different SSc manifestations treated with biologic therapies (bDMARD) but there are no specific studies that compare safety and efficacy data between different bDMARD. Our aim was to analyze and compare the use of different bDMARDs in the management of SSc organ involvements in clinical practice.

**Material and Methods:** Retrospective observational study that enrolled patients treated with bDMARD in the last five years from SSc cohort. Epidemiological and clinical data were gathered. We also collected safety data after the introduction of biologic therapy and the following complementary information: concomitant medication, mRSS, PFTs and laboratory tests before, six and twelve months after initiating bDMARD. Thereafter, we compared the safety and efficacy data of each bDMARD between them. Statistical significance was assumed in p values <0.05.

**Results:** In a cohort of 297 SSc patients we found 24 (21 women, mean age 55.7±12.6SD years old) that received a total of 32 bDMARDs in the last five years. SSc disease duration was 8.6±6.9SD years. 10 patients had lcSSc, 9 had dcSSc and 5 had SSc sine scleroderma. 12 patients had overlap syndromes. The most used bDMARD was rituximab (17) followed by tocilizumab (9), abatacept (4) and sarilumab (2). The main bDMARD indication was arthritis (50%), interstitial lung disease (25%), cutaneous involvement (20.8%) and myositis (4.2%). Each patient’s treatment and safety characteristics were summarized in Table 1.

The most prescribed bDMARD was rituximab, especially in patients with lung involvement. Tocilizumab and abatacept were mainly used in joint involvement. Abatacept was more frequently used in ACA+ patients (p<0.03). The number of concomitant immunosuppressive treatments was no different among bDMARDs.

No statistical safety differences were found between bDMARDs. Only four patients developed adverse events during treatment and only one case required to stop bDMARD (mesothelioma during rituximab).

After 12 months of bDMARD treatment, 43.8% of patients stabilized, 25% improved and 15.6% worsened their clinical outcome by physician criteria. None of the treatments showed significant differences to treat skin, lung or articular involvements between them. Tocilizumab significantly reduced acute phase reactants at 6 and 12 months of treatment in comparison with other bDMARDs.

**Conclusions:** Our results suggest that there are no safety differences between bDMARDs. Efficacy data in stabilizing or improving SSc organ involvements is encouraging. Nevertheless, our sample is small and we need more studies and randomized controlled trials to compare the use of different biologic therapies in SSc.

**Figure:**
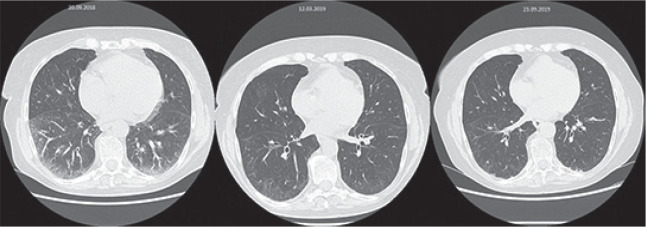


**Figure:**
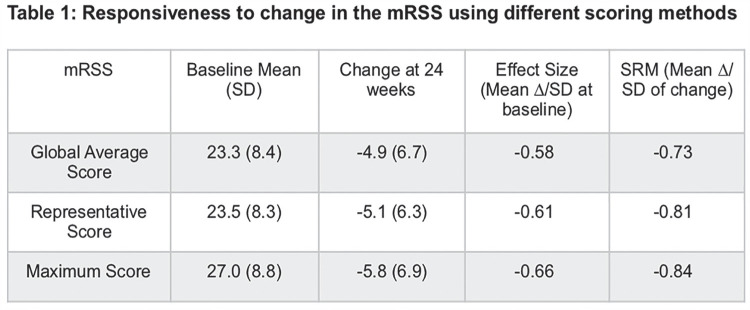


## P.329

## STELLATE BLOCKADE COMBINED TO ILOPROST AS NEW TREATMENT OPTION FOR IMPROVEMENT OF ISCHAEMIC SYMPTOMS IN PATIENTS WITH SYSTEMIC SCLEROSIS – FIRST INTERIMS ANALYSIS OF A PROSPECTIVE OBSERVATIONAL STUDY

F. Höcketstaller^1,2^, U. Henkemeier^2,3^, M. Zimmermann^4^, H. Burkhardt^1,2,3^, F. Behrens^1,2,3^, U. Drott^1^, M. Köhm^1,2^

^1^*Division of Rheumatology Goehte-University, Frankfurt, Germany*, ^2^*Fraunhofer IME Translational Medicine & Pharmacology TMP, Frankfurt, Germany*, ^3^*Centre for Innovative Diagnostics and Therapeutics Rheumatology/Immunology CIRI, Frankfurt, Germany*, ^4^*Clinic for Anaesthesiology, intensive care and pain therapy Goethe-University, Frankfurt, Germany*

**Introduction:** Peripheral ischaemia is a common symptom in systemic sclerosis (SSc) patients. For its treatment, intravenous iloprost is the most effective option. Accompanying pain-symptoms might worsen the ischaemic symptoms, so a combination with anaesthetic procedures might be effectivity as vasodilatative treatment and beneficial on subjective sensation. The aim of this study is to observe the impact of a combined treatment of iloprost with stellate blockade (ILOST) for improvement of ischaemic symptoms compared to iloprost treatment only (ILO).

**Material and Methods:** Twenty SSc-patients with indication for ILO-treatment (prophylactic or due to digital ulcerations (DU)) will be included in a prospective observational study. Patients will be offered to combine ILO with stellate blockade (ILOST). Beside documentation of disease activity characteristics such as mRSS, number of DU, capillary microscopy at baseline, after ILO-treatment and at week 12, patients are interviewed using a questionnaire targeting subjective sensation on pain and function of the hands at week 12.

**Results:** In this first interims analysis of 11 patients, mean baseline characteristics (age and gender) are well balanced. All patients had an indication for iloprost without presence of DU. 100% of the patients in the ILOST-group were diagnosed as limited SSc compared to 60% in the ILO-group (diffuse type with 40%). All patients showed abnormalities in capillary microscopy: 83,3% in the ILOST-group showed a late pattern, another 16.7% an active pattern compared to 80% in the ILO-group with a late pattern and 20% with early pattern. MRSS was low in both groups with 1,8, the disease duration in mean 15.3 years in the ILOST-group compared to 13.2 in the ILO-group, respectively. In both groups at the 12-week follow-up no new DU occurred. In the interview on subjective sensation at week 12 after treatment, questions on function, stiffness, paraesthesia, dumbness and weakness of the hands were rated. From an overall score of 480 (rating from strong agreement (5 points) to missing agreement (1 point)) the ILOST group rated a score of 314 compared to 250 in the ILO-group. 83% of the patients in ILOST answered to be willing to repeat the treatment with stellate blockade.

**Conclusions:** A new treatment approach to improve acute ischaemic symptoms was tested by combining stellate blockade to iloprost treatment. No new DU occurred up to 12 weeks after treatment in both groups. The additional intervention was well tolerated and asked to repeat. Subjective sensation on function, paraesthesia, dumbness and weakness of the hands was improved in the combined group.

**Figure:**
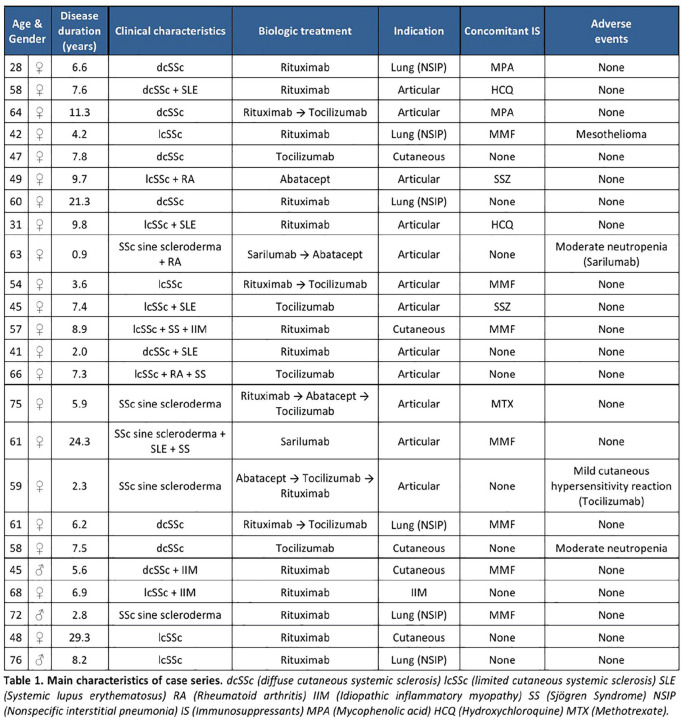


## P.330

## BOTULINUM TOXIN: A PROMISING TREATMENT MODALITY FOR OROFASCIAL DIFFICULTIES IN SCLERODERMA PATIENTS

S. Kafaja^1^, P. Clements^1^, D. Furst^1,2^, D. Zinati^3^, K. Omrani^4^

^1^*David Geffen School of Medicine, Department of Medicine, Division of Rheumatology, Los Angeles, USA*, ^2^*Pacific Arthritis Care Center, Los Angeles, USA*, ^3^*USC School of Dentistry, Los Angeles, USA*, ^4^*Cedars Sinai Medical Center, Los Angeles, USA*

**Introduction:** Systemic Sclerosis (SSc) is an autoimmune disease with multi-organ and diverse manifestations affecting the quality of life of patients, (Baron et al., 2014 and 2015). Even though orofacial involvement is a common concern to SSc patients, this area of care continues to be unmet. Orofacial manifestation in SSc is quite variable ranging from malocclusion, to decreased oral aperture, periodontal disease, cavities, and xerostomia. Decreased oral aperture appears to affect SSc patients not only aesthetically but also functionally. Both surgical and non-surgical approaches to increasing oral aperture in SSc patients have been reported in the literature. Of note, most of the non-surgical techniques can be time-consuming, require ongoing exercise, and yet offer short-lived results. Botulinum toxin, a non-surgical approach, may offer a minimally invasive and sustainable approach to improving oral aperture in SSc patients.

**Material and Methods:** We report our experience using botulinum toxin injections in SSc patients with decreased oral aperture among other orofacial manifestation of SSc. The greatest distance between the incisal edges of the maxillary central incisors to the incisal edge of the mandibular central incisors at the midline when the mouth is open as wide as possible is measured prior to the injections. The anterior and posterior margins of the masseter muscle is confirmed by palpation and then marked. Using a 1 mL ultra-fine syringe with a 30 gauge 1/2 inch needle, 20 to 25 units of Botulinum Toxin is injected to left and right masseter muscles under sterile conditions.

**Results:** We report our findings in three patients with diffuse SSc, all female, age 19 to 72, and all meeting 2013 SSc classification Criteria. All patients underwent a single Botulinum treatment as described in the method section. Maximum interincisal opening prior to the Botox injection ranged from 13- 30 mm. Maximum interincisal opening post Botox improved by 3 mm immediately post procedure and was sustainable in all beyond 6 months. All patients have reported immediate and sustained results for up to one year. Additionally, all patients reported improved ability to masticate, as well as improved accessibility to their dentists and hygienists alike to treat

**Conclusions:** We offer our experience in a series of women with diffuse SSc who share a common difficulty of decreased oral aperture, where Botulinum toxin may offer a sustained result albeit small clinically but can be quite palpable in its effect on patient’s quality of life.

## P.331

## THERAPY WITH RITUXIMAB IN SYSTEMIC SCLEROSIS (SSC): A SINGLE CENTRE COHORT STUDY

I. Jandova, N. Venhoff, J. Thiel, C. Glaser, R. Voll

*Department of Rheumatology and Clinical Immunology, Medical Center University of Freiburg, Faculty of Medicine, Univer, Freiburg, Germany*

**Introduction:** Systemic sclerosis (SSc) is a heterogeneous multisystem connective tissue disease. A standard treatment with cyclophosphamide or mycophenolate in patients with severe skin or progressive lung involvement shows often only partial and temporary effects. B cell depletion with rituximab showed stronger effect in individual patients and constitutes potential therapy of this severe disease.

Retrospective evaluation of the treatment effects of B-cell depletion with rituximab in a cohort of adult SSc patients treated in one rheumatology center.

**Material and Methods:** Ten patients (9 with diffuse SSc, 1 with limited SSc) were included in this retrospective data analysis. All patients had lung involvement. The patients were treated with RTX in median 24 month (range 12-61) with a median cumulative dose of 4 g (range 2-12) in 3,5 cycles (range 2-6). Seven patients were treated additionally with sDMARD (5x methotrexate, MTX; 2x mycophenolate mofetil, MMF).

Laboratory parameters, pulmonary function tests as well as the modified Rodan skin score (mRSS) were recorded.

**Results:** All, but one patient, showed improvement in mRSS (median 2 points improvement with range 0-26) at the end of the follow-up. The pulmonary function tests show improvement of 5,0% (median) forced vital capacity (FVC) and increase of 0,5% (median) of carbon dioxide diffusion (DLCO). The use of MMF did not show any additional benefit.

The patients (5) who were treated with RTX within the first 3 years after diagnosis numerically showed a better outcome (median of 3 points improvement of mRSS; 8,0% and 1,0% increase of FVC and DLCO, respectively), however those differences did not reach statistical significance.

No severe adverse effects related to RTX were recorded.

**Conclusions:** These results suggest that RTX could be an effective and safe therapy of patients with SSc. Our observations are in line with previously published data. A prospective placebo-controlled multicentre study is required to proof the benefit of RTX in systemic sclerosis.

## P.332

## CHANGES IN ANTINUCLEAR ANTIBODY TITERS, ASSOCIATED WITH SYSTEMIC SCLEROSIS, DURING LONG-TERM RITUXIMAB THERAPY

L. Garzanova, L. Ananyeva, O. Koneva, O. Ovsyannikova, O. Desinova, M. Starovoytova, S. Glukhova, M. Cherkasova, A. Alexankin

*V.A. Nasonova Research Institute of Rheumatology, Moscow, Russia*

**Introduction:** There are studies in which treatment with rituximab(RTX) in patients with systemic sclerosis(SSc) led to a decrease in antibody titres(ANA, anti-topoisomerase-1(Scl-70). However, in these works there were a small number of patients (12 and 8 pts), which makes it interesting to study this issue in a larger group of pts.

**Material and Methods:** This study included 88 pts with SSc. Data were collected prospectively. The mean follow-up period was 27months (12-42). The mean age was 47years (17-71), female-73pts(83%), the diffuse cutaneous subset of the disease had 50pts (57%). The mean disease duration was 5,9±4,8years. The cumulative mean dose of RTX was 2,9±1,1grams. All patients received prednisone at a dose of 11,7±4,4mg, immunosuppressants received 42% of them. The results are presented in the form of mean values, median, upper and lower quartiles.

**Results:** As a result of therapy, the Rodnan skin score(mRss) decreased from 11,21±9,33 to 6,19±4,74 (p<0,001). The disease activity index decreased from 2,9±1,74 to 1,36±1,15(p<0,001). Forced vital capacity,% predicted, increased from 76,35±19,65 to 84,37±21,04(p<0,001). Diffusing capacity for carbon monoxide,% predicted, increased from 45,56±17,72 to 47,62±16,96(p<0,019). The absolute number of B-cells decreased from 0,224±0,19 to 0,0175±0,058(p<0,0000). The dose of prednisolone decreased from 11,7±4,4 to 9,2±3,2mg(p<0,000001). Immunosuppressants in the follow-up received 49% of pts, because for some of them they were prescribed for the first time simultaneously with RTX. There was a positive effect of therapy in 96% of pts. An initially positive ANA titer was found in 92% of pts and varied in the range: 1/320-11pts, 1/640-46pts, 1/1280-24pts. Positive Scl-70 was in 75%, positive anti-centromere antibodies(ACA)–3pts. The mean titer of Scl-70 was 125±89units/ml(0,1-200). ANA decreased from 1/640(median)(25th%-1/640; 75th%-1/1280) to 1/320(median) (25th%-1/320; 75th%-1/640),(p<0,001). The ACA hasn’t changed. Scl-70 titer decreased from 125,02±89, 12 to 108,6±86,89units/ml(p<0,007). A moderate positive, statistically significant correlation was found between ANA and Scl-70 (r=0,403; p=0,0001). A statistically significant correlation was revealed between the mRss and ANA(r=0,26; p<0,014).

**Conclusions:** In our study, performed on a large group of pts, a significant decrease in the levels of ANA and Scl-70 was shown at the long-term complex therapy, including RTX. The decrease of ANA was accompanied with a decrease in the level of B-cells, mRss, disease activity index, an increase of pulmonary function tests and the cumulative dose of RTX. A decrease in the titer of all antibodies becomes apparent with a sufficiently long treatment. It can be assumed that the changes of the ANA and Scl-70 may be useful for assessing the effectiveness of long-term therapy.

## P.333

## POSSIBLE BENEFIT OF TADALAFIL CREAM FOR THE TREATMENT OF DIGITAL ULCERS IN SYSTEMIC SCLEROSIS

A. Fernandez-Codina, M. Kazem, J.E. Pope

*Rheumatology Division, Western Ontario University, London, Canada*

**Introduction:** Digital ulcers (DU) in systemic sclerosis (SSc) cause high morbidity and functional impairment1. Treatment options for DU are limited and influenced by access to drugs (geographical and financial). The use of topical treatments has been barely explored.

**Material and Methods:** Using data from a single centre SSc registry, we reviewed the use of tadalafil cream for new DUs in SSc patients from November 2018 to September 2019. Tadalafil 2% cream is compounded and dispensed per individual prescription in Ontario at a lower cost than oral phosphodiesterase-5 inhibitors. The drug was prescribed as 0.5 g of tadalafil 2% cream (equivalent to 10 mg of oral tadalafil) to be applied twice daily in the web spaces adjacent to the finger with the new DU for 4 weeks.

**Results:** Twelve patients, 69% women with a mean age of 55 years had DUs treated with tadalafil cream. Fifty-four percent had diffuse cutaneous SSc, 47% limited and 8% sine scleroderma phenotypes. Three quarters had previous DUs. Upon the diagnosis of the new DU, all but 2 patients were on treatment for RP (median 1 drug). The patients were all treated with tadalafil cream added to current treatment (calcium channel blockers 67%, aspirin 31%, losartan and statins 15%, and prazosin and sildenafil 8%). The targeted ulcers were moderate according to the DU severity visual analogue scales (VAS, 0-100), and no digit with critical ischemia or large necrotic ulcer was treated with tadalafil cream (Table 1). There was a statistically significant improvement in the RP severity VAS assessed both by patients and physicians. DU severity VAS and general assessment (0-100) improved also in both groups, but only statistically significantly from the physician’s perspective. There was a reduced number of new DUs (p=0.088). Topical tadalifil was well tolerated but one person stopped their background nifedipine due to nausea.

**Conclusions:** The addition of tadalafil cream added to standard of care might be an effective treatment for new DU in SSc. Randomized clinical trials are needed to confirm the efficacy of tadalafil given topically for SSc DUs.

## P.334

## EFFECTS OF RITUXIMAB THERAPY IN PATIENTS WITH SYSTEMIC SCLEROSIS

M. Starovoytova, O. Desinova, L. Ananieva, L. Garsonova, O. Koneva, O. Ovsyannikova

*V.A.Nassonova research Rheumatology Institute, Moscow, Russia*

**Introduction:** Systemic sclerosis (SSc) is an autoimmune disease characterized by skin and multi-organ involvements induced by overproduction of autoantibodiies by B cells. Rituximab (RTX) is used for treatment of SS and other autoimmune diseases. The results are quite promising.

**Material and Methods:** There were 90 SSc pts in the prospective study, 75% were women, 57% pts had diffuse SSc. SSc was confirmed ACR/EULAR criteria. The average duration of the disease was 5,8 years and of the study was 27 months. For the purpose of the analysis, a group of pts was selected that had no less than two evaluation points from 12 to 42 months and having received 2,9±1.1 g RTX in average during such period. The examination included both routine clinical laboratory parameters and special research. We evaluated the modified Rodnan skin score (mRSS), forced vital capacity (FVC), diffusing capacity of carbon monoxide (DLCO), the Valentini Disease Activity index, estimated glomerular filtration rate ( eGFR) before and after completed RTX. All pts included in the study received treatment with vascular, anti-inflammatory and/or immunosuppressive medicines. The average dose of prednisolone was 11,8±4,4 mg/day.

**Results:** In general, more than 90% pts demonstrated different kinds of improvements. A reliable improvement was documented based on a number of indicators, which reflect typical features of the disease. Thus, skin induration was reduced (skin score decreased), lung functional capacity increased (increase of FVC of more than 5%). The skin score was reliably decreased from 11,2±9,3 to 6,2 ±4,9 (p<0.0001), FVC was increased from 76,9±19,9 to 84,7±20,9% (p<0.0001), also the increase of DLCO from 46,3±18,3 to 47,8±16,9 % from the proper amount was detected. The Valentini Disease Activity index reliably reduced from 2,93±1,7 to 1,38±1,2 points (p<0,0001). Under the RTX therapy, the decrease of glucocorticoids dose was insignificant, but statistically reliable. Thus, prednisolone dose reduction from 11,8±4,4 to 9,22±3,2 mg/day was achieved. decline of eGFR was detected. After RTX treatment the estimated glomerular filtrate rate (eGFR) has decreased in the general group of patients. This decrease was clinically insignificant but reliable from 100,2±23 to 94,6±22 ml/min/1,73m2.

**Conclusions:** Thus, RTX has a positive influence on the basic parameters of SSc, mainly on skin damage and lung functionality, reduces the general disease activity. The influence of the RTX on kidney function requires further long-term study.

**Figure:**
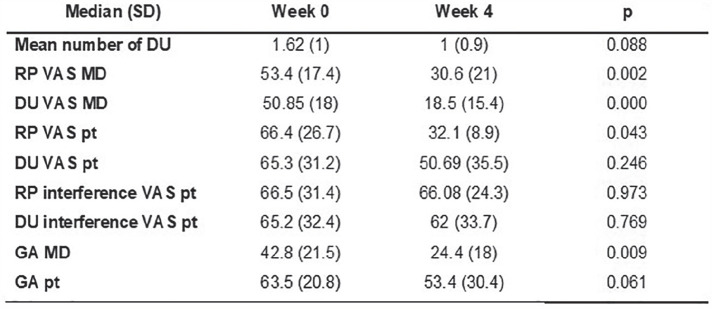


## P.335

## EFFICACY OF SELEXIPAG ON DIGITAL ULCERS IN SYSTEMIC SCLEROSIS: ANALYSIS OF A PRELIMINARY STUDY

N. Del Papa^1^, S. Bilia^2^, D. Giannini^2^, A. Grimaldi^3^, E. Quarta^3^, F. Pignataro^1^, A. Minniti^1^, W. Maglione^1^, P. Migliorini^2^, A. Tavoni^2^

^1^*UOC Rheumatology Day Hospital, ASST G. Pini-CTO, Milano, Italy*, ^2^*UOC Immuno-Allergologia. Dip. Medicina Clinica e Sperimentale, Università di Pisa, Pisa, Italy*, ^3^*UOS Reumatologia, Osp. V. Fazzi, Polo Riabilitativo San Cesario, Lecce, Italy*

**Introduction:** Digital ulcers (DUs) are main features of Systemic Sclerosis (SSc) with a high impact on the quality of life. Management of DUs is challenging and requires a complex treatment, including both systemic and local therapies. EULAR recommendations for the treatment of SSc DUs indicate that intravenous prostanoids (IVPs) should be considered in the treatment of active DUs. However, intravenous administration often presents a few hurdles including difficulties in venous access, high costs for hospitalization, and time taken from work productivity of patients. We evaluated the clinical usefulness and tolerability of selexipag in the treatment of DUs in patients who failed standard treatments and benefited from the long-lasting use of IVPs but experienced important difficulties in venous access.

**Material and Methods:** FIve patients (4 female and 1 male), mean age 54.5+13.2, meeting 2013 ACR/EULAR classification criteria for SSc, presenting DUs were consecutively recruited. Four of five patients had the diffuse form of the disease and one had limited SSc. The mean duration of SSc was 12.5+7.7 years. All patients had been treated with standard treatments (calcium-channel blockers, ACE inhibitors, bosentan, sildenafil) with unsatisfactory results or side-effects. Indeed they were benefiting from the longlasting use of IVPs but experienced important difficulties in venous access. The maintenance dosage of Selexipag was 2700+600mcg. Clinical assessments, status of DUs, patient report of Raynaud’s Condition score (RCS) and visual analogue scale (VAS) for pain, were performed every 4 weeks for six months.

**Results:** In all, 21 ulcers were present at baseline; these decreased to 7 at 3 months (p=0.071) and 1 at the end of the follow-up (p=0.033). At week 24, the number of RP attacks and RCS were decreased without reaching statistical significance (p=0.079 and p=0.074, respectively). The VAS was instead significantly improved (p=0.04). During the follow-up, no new ulcers were observed. Adverse effects typically associated with therapies targeting the prostacyclin pathway (nausea, vomiting, headache) were reported as mild or moderate in intensity and limited to the first weeks of treatment. No patients dropped out.

**Conclusions:** Our data, although obtained in a small number of patients, suggest an effect of Selexipag on healing and prevention in SSc-related DUs. Furthermore, the reduction of DUs led to a significant decrease of pain. According to previous reports, no important adverse effect was observed in the number of RP attacks and RCS. The efficacy of Selexipag in the treatment of DUs in SSc patients should be validated in controlled studies on a wider population.

## P.336

## RITUXIMAB IN SYSTEMIC SCLEROSIS MANAGEMENT: MULTICENTRIC CLINICAL EXPERIENCE AND LONG-TERM FOLLOW-UP

F. Lumetti^1^, L. Magnani^2^, A. Spinella^1^, E. Cocchiara^1^, G. Bajocchi^2^, C. Ferri^1^, C. Salvarani^1^, D. Giuggioli^1^

^1^*Scleroderma Unit, Chair of Rheumatology, University of Modena and Reggio Emilia, Italy, Modena, Italy*, ^2^*Rheumatology Unit, AUSL-IRCCS of Reggio Emilia, Italy, Reggio Emilia, Italy*

**Introduction:** The treatment of systemic sclerosis (SSc) is a clinical challenge because of its complex pathogenesis. Definitive data on Rituximab (RTX) use in SSc are still lacking but preliminary data from case report or non-randomized clinical trial (RCT) are somehow encouraging even if showing contrasting results. According to our previous experience with RTX, we suggested a possible therapeutic role of RTX in SSc. Our aim was to investigate the role and efficacy of RTX in our SSc patients.

**Material and Methods:** We enrolled 24 SSc patients (M/F 7/17, age 55±14SD years, disease duration 11.7± 7.3SD years, lc/dcSSc 11/13) were treated with one or more cycles of RTX (4 weekly/infusions/375 mg/m2) and evaluated them during a mean follow-up period of 12.3±6.6SD years. Patients underwent to RTX if they had cutaneous and/or articular involvement, or interstitial lung disease. Treatment potential benefit were evaluated 6 months after the first cycle and at the end of follow-up. Serum levels of pro-inflammatory cytokines and chemokines (IL-6, IL-8, CXCL9, CXCL10, CXCL11, CCL5) were assessed before the first RTX cycle and 6 months after.

**Results:** The extent of skin sclerosis measured by means of modified Rodnan skin score (mRSS) significantly improved (p<0.0001), and remained stable at the end of the follow-up (p=0.105). This effect was more evident in patients with diffuse cutaneous SSc. Arthritis revealed a good response to RTX treatment leading to a clear-cut reduction of swollen and tender joints in 20/24 patients (p<0.0001). Lung fibrosis detected in 19/24 on chest-CT remained stable during the entire follow-up, as well as pulmonary function tests (PFTs). No significant side effects were observed during the entire follow-up. IL-6 and IL-8 serum level decreased about 100%, but those cytokines were elevated at baseline in few patients only (respectively 9/24 and 11/24). Mean serum levels of chemokines were elevated at baseline in 24/24 patients and decreased about 50% after RTX therapy.

**Conclusions:** Our data support previous trials and preliminary reports on RTX, showing the efficacy and safety of this drug in the management of SSc. The improvement of skin sclerosis, articular symptoms and the stabilization of lung involvement were identified as the main results. Further studies are also required to investigate interactions between chemokines and cytokines in the pathogenesis of SSc.

## P.337

## INTRAVENOUS IMMUNOGLOBULIN THERAPY IN PATIENTS WITH SYSTEMIC SCLEROSIS: A MULTICENTER EXPERIENCE

J.L. Tandaipan^1^, A. Guillen^2^, P. Carreira^3^, F.J. Narváez^4^, M. Rubio-Rivas^5^, V. Ortiz-Santamaría^6^, C. De La Puente^7^, J.M. Pego^8^, R. García-Vicuña^9^, A. Pros^10^, C. Galisteo^11^, B. Atienza-Mateo^12^, J. Lluch^4^, R. Blanco^12^, C.P. Simeón-Aznar^2^, I. Castellví^13^

^1^*Hospital Universitari Mutua de Terrassa. Department of Rheumatology, Terrassa, Spain*, ^2^*Hospital Universitari Vall Hebron, Department of Internal Medicine, Barcelona, Spain*, ^3^*Hospital Universitario 12de Octubre, Department of Rheumatology, Madrid, Spain*, ^4^*Hospital Universitari de Bellvitge, Department of Rheumatology, Hospitalet de Llobregat, Spain*, ^5^*Hospital Universitari de Bellvitge, Department of Internal Medicine, Hospitalet de Llobregat, Spain*, ^6^*Hospital General de Granollers, Department of Rheumatology, Granollers, Spain*, ^7^*Hospital Universitario Ramon y Cajal, Department of Rheumatology, Madrid, Spain*, ^8^*Hospital do Mexoeiro, Department of Rheumatology, Vigo, SPAIN*, ^9^*Hospital Universitario de La Princesa, Department of Rheumatology, Madrid, Spain*, ^10^*Hospital del Mar, Department of Rheumatology, Barcelona, Spain*, ^11^*Corporació Sanitaria Parc Taulí, Department of Rheumatology, Sabadell, Spain*, ^12^*Hospital Universitario Marqués de Valdecilla, Department of Rheumatology, Santander, Spain*, ^13^*Hospital Universitari de La Santa Creu I Sant Pau, Department of Rheumatology, Barcelona, Spain*

**Introduction:** Systemic sclerosis (SSc) is can present different vascular, inflammatory and fibrotic involvements. Intravenous immunoglobulin (IGIV) could be useful to treat some manifestations in autoimmune diseases. Our aim was to describe the safety and efficacy of IGIV use in a large cohort of SSc patients.

**Material and Methods:** retrospective multicenter observational study that enrolled SSc patients treated with IVIG . In each patient we collected the main indication to use IVIG, survival of the treatment and the following data: epidemiological characteristics, cutaneous subset (limited or diffuse), presence of overlap disease, specific antibodies, capillaroscopic findings, concomitant therapies and complications related to SSc. We also collected before and after IVIG treatment the modified Rodnan skin score (mRSS) pulmonary function tests, clinical efficacy and related adverse events.

**Results:** 49 patients (83% women, mean age 57.9 ± 17.1 years old) were recruited. Years since Raynaud’s and disease onset were 12.1±10.1 and 9.5±7.6 years old respectively. The diffuse cutaneous subset was the most frequent (59,2%) and 29/49 had Overlap syndrome . Patient’s characteristics are summarized in table 1.

IGIV was used to treat 6 involvements. The most frequent indication was myositis (24/49) followed by gastrointestinal (12/49) and cutaneous (11/49) involvements. The mean of cycles was 9. Five, fourteen and nine patients have been treated with corticoids, synthetic DMARD and biologic therapy respectively. 9 patients were in ongoing biologic DMARD therapy (mostly rituximab).

Patients with myositis had the best response to IGIV by investigator criteria. We also found in skin involvement a delta improvement of -2.6 (p <0.03) in mRSS at the end of the follow-up.

In the patients whose indication was interstitial lung disease we found no differences in % FVC or % DLCO outcomes during the follow-up, but patients with myositis had significant worse %FVC values previous to IGIV in comparison with patients without myositis. After IGIV we did not find differences between groups.

No benefits in gastrointestinal involvement was observed but it is noteworthy that patients that IGIV was prescribed for digestive involvement received less cycles.

Only 3 adverse effects were notified and two of them required hospitalization. No specific safety concerns was found in all patients with immunosupressive concomitant therapy

**Conclusions:** Our results suggest that IVIG could be useful to treat some conditions in specific profiles with a good safety profile including the use of concomitant immunosuppressive therapy. Prospective studies and randomized clinical trials are warranted to establish the efficacy and safety of IVIG in SSc.

**Figure:**
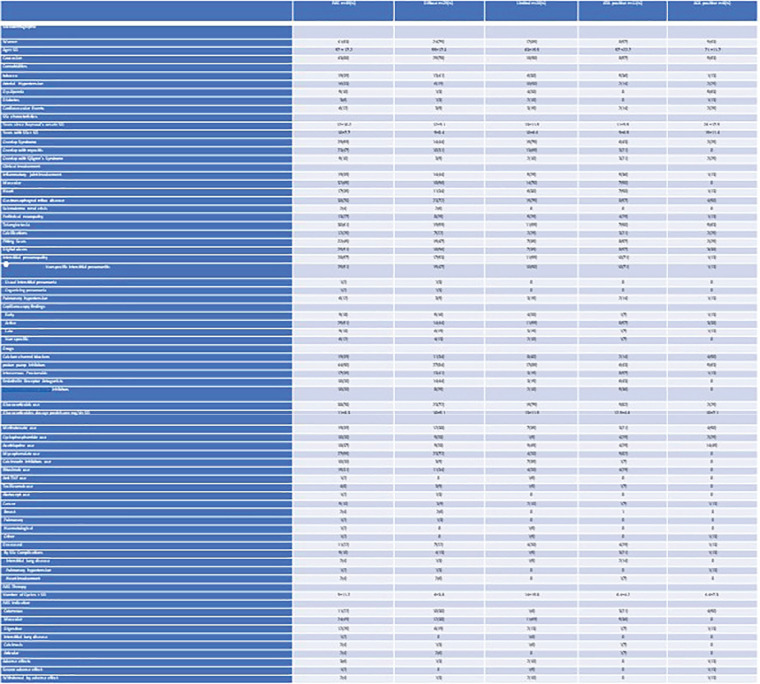


## P.338

## SAFETY AND EFFECTIVENESS OF ABATACEPT IN SYSTEMIC SCLEROSIS IN CLINICAL PRACTICE

I. Castellví^1^, M. Elhai^2^, C. Bruni^3^, P. Airò^4^, S. Jordan^5^, L. Beretta^6^, V. Codullo^7^, C.M. Montecuco^7^, M. Bokarewa^8^, F. Iannonne^9^, A. Balbir^10^, V.M. Hsu^11^, O. Distler^5^, M. Matucci-Cerinic^3^, Y. Allanore^2^

^1^*Hospital Universitari de la Santa Creu I Sant Pau, Department of Rheumatology And Autoimmune Diseases, Barcelona, Spain*, ^2^*Cochin Hospital, Rheumatology a Department, Paris, France*, ^3^*Azienda Ospedialero-Universitaria Careggi (Aouc), Department of Experimental and Clinical Medicine, Firenze, Italy*, ^4^*Spedali Civili Brescia, Uo Reumatologia ed Immunologia Clinica, Brescia, Italy*, ^5^*University Hospital Zurich. Department of Rheumatology, Zurich, Switzerland*, ^6^*Fondazione Irccs Cà Granda Ospedale Maggiore Policlinico di Milano, Milano, Italy*, ^7^*Policlinico San Matteo, Unità Operativa e Cattedra di Reumatologia, Pavia, Italy*, ^8^*Sahlgrenska Hospital. Rheumatology and Inflammation Research. Institute of Medicine, Gothenburg, Sweden*, ^9^*University of Bari. Rheumatology Unit-Deto, Bari, Italy*, ^10^*Rambam Health Care Campus and Rappaport Faculty of Medicine. B. Shibe Rheumatology Unit, Haifa, Israel*, ^11^*Rutgers-Robert Wood Johnson Medical School Scleroderma Program, New Brunswick, USA*

**Introduction:** Systemic Sclerosis (SSc) is a rare autoimmune disease that causes vasculopathy, immune dysfunction and culminates in tissue fibrosis. T cells and costimulatory signals driven by CD28/CD86 surface molecules have a pivotal role in SSc pathogenesis. Therefore, abatacept, a drug that downregulates T cell activity, could be a candidate treatment of SSc. The European Scleroderma Trials and Research (EUSTAR) group published preliminary experience in five and seven SSc patients with arthritis or myopathy treated with abatacept and some improvement of arthritis, but no effect on muscular involvement was found. Currently, a randomized controlled trial of abatacept in diffuse cutaneous SSc (NCT02161406) aiming at evaluating the skin effects is completed and preliminary results have been presented in abstract form. We performed an observational study to analyze the efficacy and safety of abatacept in SSc patients with different organ involvements.

**Material and Methods:** Retrospective multicenter observational study that enrolled patients with SSc treated with ABA from EUSTAR cohort. We collected epidemiological data and clinical outcomes. First, we analyzed the frequency of adverse effects. Secondly, we compared the evolution of different organ manifestations during ABA treatment. We collected data from 6 months before start of therapy to the last follow-up the following parameters: modified Rodnan Skin Score (mRSS), joints, lung and gastrointestinal involvement, concomitant medications, and laboratory tests.

**Results:** Data on twenty-seven patients with SSc were collected (93% females; 67% limited SSc). Rheumatoid arthritis was the most frequent concomitant autoimmune disease. ILD was present in 15 patients. Anti-Scl 70 antibodies were present in 13 patients and rheumatoid factor and ACPA antibodies were present in eight and seven patients respectively. The main indication to use abatacept was joint involvement (59%) followed by myositis (26%). Patient’s characteristics are in table.

A total of 16 adverse effects were reported in 28 months of treatment including five that required hospitalization. Most of them occurred in the first 3 months after starting abatacept .

After 12 months, the number of tender and swollen joints decreased compared to baseline (p<0.03 and p<0.02 respectively). Moreover, a beneficial effect on HAQ-DI at 3 and 6 months (p<0.05) and on morning stiffness at 6 and 12 months (p<0.03) was observed. We also observed a decrease in the modified Rodnan skin score (p<0.05). No changes in lung or gastrointestinal involvement were found.

**Conclusions:** ABA demonstrated a good safety profile and seems to have some effectiveness on joint involvement and related disability in SSc patients treated in routine care.

**Figure:**
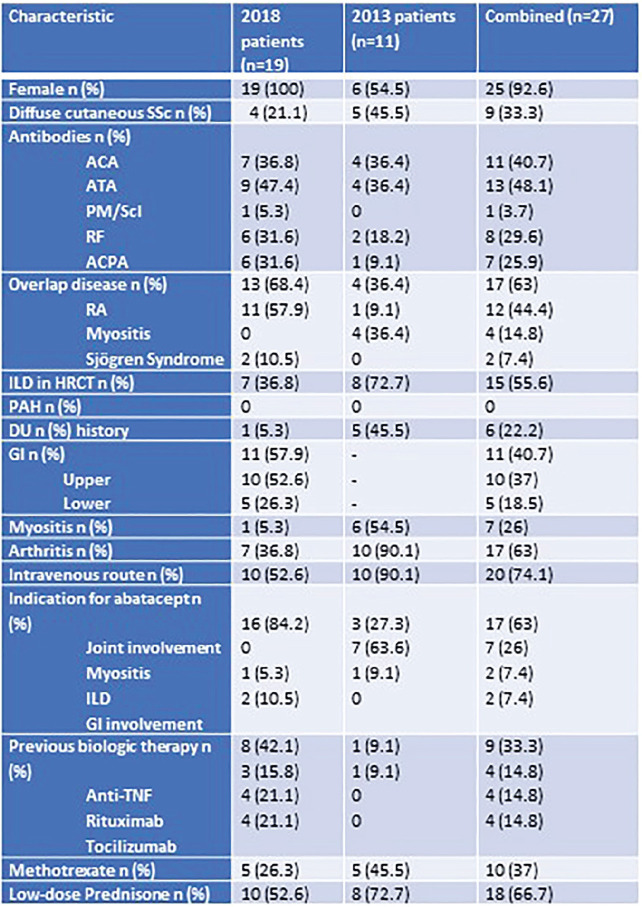


## P.339

## SAFETY AND EFFICACY OF RITUXIMAB BIOSIMILAR IN SYSTEMIC SCLEROSIS: AN ITALIAN MULTICENTER STUDY

C. Campochiaro^1^, G. De Luca^1^, M.G. Lazzaroni^2^, E. Zanatta^3^, S. Bosello^4^, M. De Santis^5^, A. Cariddi^1^, C. Bruni^6^, C. Selmi^5^, E. Gremese^4^, M. Matucci-Cerinic^6^, A. Doria^3^, P. Airò^2^, L. Dagna^1^

^1^*Unit of Immunology, Rheumatology, Allergy and Rare Diseases, IRCCS San Raffaele Hospital, Vita-Salute San Raffaele Unive, Milan, Italy*, ^2^*Rheumatology and Clinical Immunology, Spedali Civili and University of Brescia, Brescia, Italy*, ^3^*Department of Medicine - DIMED, Division of Rheumatology, University of Padova, Padova, Italy*, ^4^*Division of Rheumatology, Fondazione Policlinico Universitario A. Gemelli- IRCCS, Rome, Italy*, ^5^*Division of Rheumatology and Clinical Immunology, Humanitas Clinical and Research Center - IRCCS, Rozzano, Italy*, ^6^*Division of Rheumatology AOUC, University of Florence, Florence, Italy*

**Introduction:** Recent clinical data support the use of rituximab (RTX) in systemic sclerosis(SSc). RTX biosimilar (RTX-B) offers a more affordable option but its efficacy and safety have not yet been evaluated in SSc. We aimed to assess the safety and efficacy of RTX-B(CT-P10) in SSc.

**Material and Methods:** data about SSc patients treated with RTX-B(1gr repeated after 2 weeks) and with at least 6 month follow-up were retrospectively collected from 5 Italian centers. Either SSc patients naïve to RTX(RTX-Bn) or already treated with at least 1 course of RTX originator (RTX-O) and subsequently switched to RTX-B(RTX-Bs) were considered. A comprehensive assessment of disease characteristics and organ involvement was available at baseline and at final follow-up for all the patients. Non parametric tests were used for statistical analysis.

**Results:** 33 SSc patients were enrolled: 29 (87.9%) females; mean age 51.6±14.2 years; mean disease duration 9.8±8.1 years; 21 (64.5%) with diffuse cutaneous SSc(dcSSc); 20(60.1%) with anti-topoisomeraseI; 7(21.2%) with anti-RNA-polymeraseIII; 5(15.1%) with anti-centromere. Seventeen patients(51.5%) were RTX-Bn and 16 RTX-Bs(48.5%). In RTX-Bs patients, the median number of previous RTX-O courses was 2(range 1 – 8). RTX was decided because of skin progression in 18(54.5%), interstitial lung disease(ILD) worsening in 11(33.3%), arthritis in 12(36.4%), myocarditis and myositis in 2 patients each; in 13 patients (39.4%) RTX was administered for multiple reasons. All patients had been previously treated with immunosuppressants: mycophenolate mofetil(MMF) 25(75.7%), methotrexate(MTX) 14(42.4%), cyclophosphamide 9(27.3%), azathioprine 5(15.1%), tocilizumab in 2(6.1%), etanercept and leflunomide in 1 patient each. At RTX-B introduction, 21(63.6%) patients were on concomitant immunosuppressant: 15(71.4%) MMF and 6(28.6%) MTX; 23 patients(69.7%) were also on steroids(mean dose:4.8±1.6 mg/day). At 6 months after RTX-B treatment, a significant reduction of the modified Rodnan skin score(mRSS), DAS28 and erythrocyte sedimentation rate(ESR) was observed in the entire cohort (p=0.002,p=0.005,p=0.009, respectively); mRSS was significantly reduced also in RTX-Bn(p<0.024) and RTX-Bs patients(p<0.031)(Table 1). No significant changes were observed for lung function tests, either in the entire cohort or in the subgroup of patients with ILD (p=ns). Only 1 RTX-Bs patient experienced a transient neutropenia 3 months after the 2nd RTX-B infusion whilst also on MTX.

**Conclusions:** in agreement with previous data published on RTX-O, also RTX-B seems to be effective in improving skin and joint involvement and in stabilizing lung function, either in RTX-Bn or in RTX-Bs SSc patients.

## P.340

## THE PHARMACOKINETICS OF MYCOPHENOLATE MOFETIL IN SYSTEMIC SCLEROSIS VARIES MANIFOLD BETWEEN PATIENTS AND IS ASSOCIATED WITH GASTROINTESTINAL INFLAMMATION

K. Andréasson^1^, K. Neringer^1^, D. Henrohn^2^, A.-C. Mårtensson^3^, D. Wuttge^1^, R. Hesselstrand^1^

^1^*Department of Rheumatology, Lund University, Lund, Sweden*, ^2^*Department of Medical Sciences, Uppsala University, Uppsala, Sweden*, ^3^*Department of Clinical Chemistry, Skåne University Hospital, Malmö, Sweden*

**Introduction:** Mycophenolate mofetil (MMF) was developed and approved to prevent graft-rejection and has since been established as an important immunosuppressant in systemic sclerosis. Individual dose monitoring based on the active metabolite mycophenolic acid (MPA) has improved clinical outcome in renal transplant recipients. A target MPA Area Under the Curve (AUC)_0-12 h at 30-60 mg h/L has been recommended in these patients. We have explored the pharmacokinetics of MMF in patients with systemic sclerosis.

**Material and Methods:** The study was approved by the Swedish Medical Products Agency and the protocol published at ClinicalTrials.gov, Identifier CT03678987. Study participants were invited locally and nation-wide with the help of the National Swedish Patient Organization for Systemic Sclerosis. We included a predefined number of 35 patients fulfilling the ACR/EULAR classification criteria for systemic sclerosis who were on a stable dose of MMF administered twice daily as a tablet. Serum levels of MPA were measured at 4 predefined time points and the MPA AUC_0-12 h calculated. Results were dose adjusted to a daily MMF dose of 2 gram. Gastrointestinal inflammation and disease manifestations were evaluated by measurement of F-calprotectin and the UCLA GIT 2.0.

**Results:** Participants exhibited a wide range of MPA exposure (range 18 – 150 mg h/L). Median MPA AUC_0-12 was 71 (interquartile range 57 – 84) mg h/L. MPA AUC_0-12 exceeded 60 mg h/L in 24 of the cases. MPA exposure was inversely correlated with body weight (rs=-0.55, p=0.003) and gastrointestinal inflammation (rs=-0.45, p=0.012) and no associations were seen with the UCLA GIT 2.0.

**Conclusions:** The interindividual MPA exposure following MMF treatment in systemic sclerosis shows considerable variation. Gastrointestinal inflammation may decrease MMF uptake. Individual dose monitoring may be warranted when prescribing MMF in systemic sclerosis.

**Figure:**
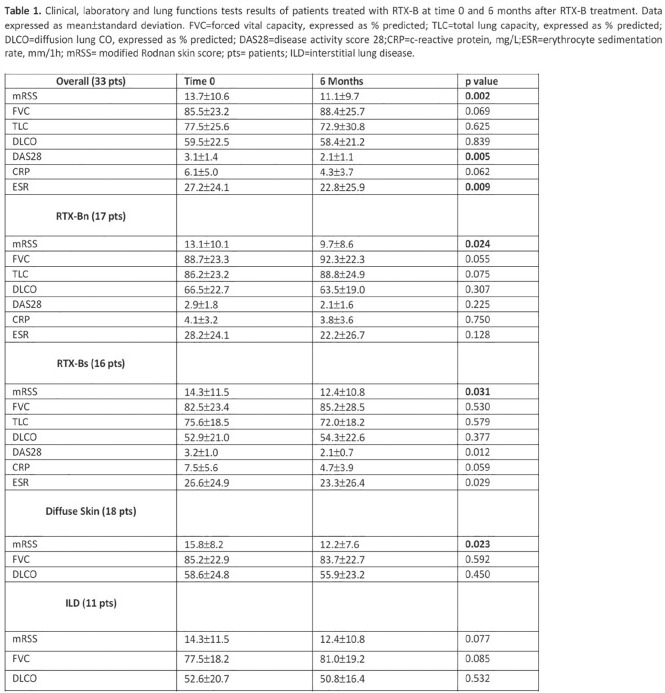


**Figure:**
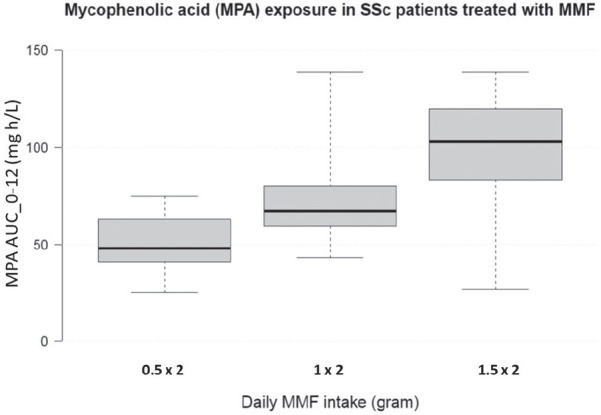


## P.341

## EFFECTIVE ANTI-FIBROTIC THERAPY FOR SYSTEMIC SCLEROSIS: DUAL-TARGETING OF PERIPHERAL CANABINOID-1 RECEPTORS AND INDUCIBLE NO SYNTHASE

R. Cinar, J.K. Park, C.N. Zawatsky, J. Abdalla, M. Iyer, G. Kunos

*Laboratory of Physiologic Studies, National Institute on Alcohol Abuse and Alcoholism, NIH, Rockville, MD, 20852, USA*

**Introduction:** Scleroderma or systemic sclerosis (SSc) is a multi-organ connective tissue disease resulting in fibrosis of the skin, heart, lungs with no effective treatment. Considering complex disease pathogenesis, targeting multiple pathways may improve therapeutic efficacy. Endocannabinoids acting via cannabinoid type 1 receptors (CB1R) promote tissue fibrosis. The activity of inducible nitric oxide synthase (iNOS) is also increased in chronic inflammation and fibrotic disorders. Recently, we showed that simultaneous targeting of CB1R and iNOS by an orally bioavailable peripherally restricted hybrid inhibitor (MRI-1867) yields greater anti-fibrotic efficacy than inhibiting either target alone in liver, lung and kidney fibrosis. Here, we evaluated therapeutic efficacy of dual targeting CB1R and iNOS by MRI-1867 in bleomycin(bleo)-induced skin fibrosis.

**Materials and Methods:** Skin fibrosis was induced in C57BL/6j (B6) and MDR1a/b-BCRP triple knock-out (KO) mice by daily subcutaneous (s.c.) injections of bleo (1μg/100μL) for 28 days. Bleo-challenged mice were treated daily for the last 14 of the 28-day period by oral gavage of vehicle or MRI-1867 (30 mg/kg/day). Endocannabinoids and MRI-1867 levels in skins were measured by mass spectrometry for assessing target exposure and engagement of MRI-1867. Fibrosis was assessed by measuring dermal thickening histologically and hydroxyproline content biochemically. We also evaluated the potential increase of drug-efflux associated ABC transporters by bleo in skin fibrosis, which could affect target exposure to test compounds as reported in bleo-induced lung fibrosis.

**Results:** Bleo-induced skin fibrosis was comparable in wildtype B6 and MDR1a/b-BCRP KO mice. However, the skin level of MRI-1867, an MDR1 substrate, was dramatically diminished in B6 mice (0.023 μM) compared to MDR1a/b-BCRP KO mice (8 μM) due to a bleo-induced increase in efflux activity of MDR1 in fibrotic skin. Furthermore, the endocannabinoids anandamide (AEA) and 2-arachidonylglycerol (2AG) were elevated about 2- 4-fold in the fibrotic vs. control skin in both mice strains. The bleo-induced increase in endocannabinoids was completely abolished by MRI-1867 treatment in MDR1a/b-BCRP KO mice but not in B6 mice, suggesting target engagement by MRI-1867 only in MDR1a/b-BCRP KO mice. Consistently, MRI-1867 treatment attenuated established fibrosis in ble-induced skin fibrosis in MDR1a/b-BCRP KO mice but not in B6 mice.

**Conclusions:** Bleomycin induces an artefact in testing antifibrotic drug candidates that are substrates of drugefflux transporters. The use of MDR1a/b-BCRP KO mice for preclinical testing of such compounds would avoid this pitfall. Furthermore, dual-targeting CB1R and iNOS for inhibition is an effective and novel anti-fibrotic therapeutic strategy for scleroderma.

## 13. Pediatric Scleroderma

## P.342

## PROPOSAL OF OUTCOME MEASURES TO BE USED ON A 12-MONTH OPEN LABEL DRUG TRIAL IN JUVENILE SYSTEMIC SCLEROSIS. RESULTS OF THE 3RD CONSENSUS MEETING IN HAMBURG DECEMBER 2018

I. Foeldvari^1^, K. Torok^2^, J. Anton^2^, M. Blakley^2^, T. Constantin^2^, M. Curran^2^, M. Cutolo^2^, C. Denton^2^, K. Fligelstone^2^, B. Heinrichs^2^, A. Höger^2^, F. Ingegnoli^2^, S. Li^2^, D. Nemcova^2^, C. Orteu^2^, C. Pilkington^2^, V. Smith^2^, A. Stevens^2^, D. Khanna^2^, D. Furst^2^

^1^*Hamburg Centre for Pediatric and Adolescence Rheumatology, Hamburg, Germany*, ^2^*Participants of the Hamburg Outcome measure consensus process, Hamburg, Germany*

**Introduction:** Juvenile systemic sclerosis (jSSc) is an orphan disease, associated with high morbidity and mortality. New treatment strategies are much needed. To develop an open label drug trial for the treatment of jSSc patients, it is necessary to clearly define how to evaluate outcomes in this disease, which are currently not existing. A group of experts in jSSc met for a concensus meeting to develop a set of outcome measures for a future prospective trial lasting 12 months.

**Material and Methods:** In the consensus meeting 26 jSSc international experts with various specialties participated (22 voted). In a nominal group technic, moderated by DEF, was used to select or reject the outcome measures, based on clinical data, which were previously suggested during the 2017 consensus meeting in Hamburg from the same participants. Agreement was defined if 80% or more of the participants approved an item.

**Results:** 10 main domains were selected. 9 of the 10 domains are defined by concrete items. The domains of the “Global Disease Activity” defined by Physician global disease activity, change in the HAQ/CHAQ-DI and the Scleroderma HAQ. The “Skin domain” is determined by the Modified Rodnan Skin Score. The “Raynaud Domain” is assessed by the Scleroderma HAQ question regarding Raynaud. The domain of the “Digital ulcerations” is evaluated with the DUCAS score. The domain of the “Musculoskeletal system” is reflected by the number of active joints, as defined for Juvenile Idiopathic Arthritis. The domain for “Cardiac involvement” is assessed by the left ventricular ejection fraction and the development of clinically significant arrhythmia as a sign of non-response. The domain of the “Pulmonary involvement” is evaluated by the FVC and age defined DLCO. The domain of the “Renal function” is defined by the item of the new occurrence of renal crisis. The domain of the “Gastrointestinal involvement” is reflected by the body mass index and gastrointestinal section of the Scleroderma HAQ. The domain of Global health/ Health related Quality of Life(QOL) was included, but the specific QOL instrument was not defined yet.

**Conclusions:** We reached consensus on domains and items which should be assessed in an open label 12 months duration clinical jSSc trial. We are planning to test this indices in the juvenile scleroderma inception cohort (www.juvenile-scleroderma.com).

## P.343

## NO DISEASE PROGRESSION AFTER 36 MONTHS FOLLOW UP IN THE JUVENILE SYSTEMIC SCLERODERMA INCEPTION COHORT

I. Foeldvari^1^, J. Klotsche^2^, O. Kasapcopur^3^, A. Adrovic^3^, M.T. Terreri^3^, R. Cimaz^3^, T. Avcin^3^, M. Katsikas^3^, D. Nemcova^3^, M.J. Santos^3^, J. Brunner^3^, T. Kallinich^3^, M. Kostik^3^, K. Minden^3^, A. Patwardhan^3^, K. Torok^3^, N. Helmus^1^

^1^*Hamburg Centre for Pediatric and Adolescence Rheumatology, Hamburg, Germany*, ^2^*German Rheumatism Research Center, Berlin, Germany*, ^3^*JSSc Collaborative Group, Hamburg, Germany*

**Introduction:** Juvenile systemic scleroderma (jSSc) is an orphan disease with a prevalence of 3 in 1 000 000 children. There is rare longitudinal prospective follow up data of patients with jSSc. In the international juvenile systemic scleroderma cohort (JSScC) patients are followed with a standardized assessment prospectively.

**Material and Methods:** Patients diagnosed according the ACR 2013 criteria for systemic sclerosis were included, if they developed the first non-Raynaud symptom before the age of 16 and were under the age of 18 at the time of inclusion. Patients were followed prospectively every 6 months with a standardized assessment.

**Results:** 39 patients in the JSScC had 36 months follow up. 80% had a diffuse subtype. 95% of the patients were Caucasian origin. 31 of the patients were female (80%). Mean disease duration at time of inclusion was 3.5 years. Mean age onset of Raynaud’s was 8.8 years and mean age of onset at the first non-Raynaud´s was 9.5 years. Around 30% of the patients were anti-Scl70 positive and none of them anti-centromere positive. The MRSS dropped from the time point of the inclusion into the cohort from 13.9 to 11.8 after 36 months. Pattern of organ involvement did not show any significant change, beside the increase of the nailfold capillary changes from 49% to 73% (p=0.037). No renal crisis occurred. No mortality was observed.

They were positive significant changes in the patient related outcomes. The physician global disease activity decreased from 40.0 to 22.1 assessed on a VAS scale of 0 to 100 (p <0.001).

Patients global disease activity decreased from 43.3 to 20.4 and patients global disease damage from 45.0 to 21.7 both assessed on a VAS scale of 0 to 100 (p<0.001).

**Conclusions:** After 36 months follow up, we could observe a significant improvement of patient related outcomes and only one significant change in organ pattern involvement. In a mostly diffuse subset patient population this is a very promising result regarding outcome.

Supported by the Joachim Herz Stiftung

## P.344

## UNDER DETECTION OF INTERSTITIAL LUNG DISEASE IN JUVENILE SYSTEMIC SCLEROSIS (JSSC) UTILIZING PULMONARY FUNCTION TESTS. RESULTS FROM THE JUVENILE SCLERODERMA INCEPTION COHORT

I. Foeldvari^1^, B. Hinrichs^2^, K. Torok^3^, M.J. Maria Jose^3^, O. Kasapcopur^3^, A. Adrovic^3^, V. Stanevicha^3^, F. Sztajnbok^3^, M.T. Terreri^3^, E. Alexeeva^3^, J. Anton^3^, M. Katsikas^3^, V. Smith^3^, T. Avcin^3^, R. Cimaz^3^, M. Kostik^3^, T. Lehman^3^, W.-. Sifuentes-Giraldo^3^, N. Helmus^1^

^1^*Hamburg Centre for Pediatric and Adolescence Rheumatology, Hamburg, Germany*, ^2^*Kinderklinik Heidberg Pulmologie, Hamburg, Germany*, ^3^*JSSc Collaborative Group, Hamburg, Germany*

**Introduction:** Juvenile systemic sclerosis (jSSc) is an orphan disease with a prevalence in around 3 in a million children. Pulmonary involvement in jSSc occurs in approximately 40 % in the inception cohort. Traditionally in jSSc, pulmonary function testing (PFT) with FVC and DLCO are used for screening and computed tomography (HRCT) was more reserved for those with abnormal PFTs. More recently, it has become apparent that PFTs might not be sensitive enough for detecting ILD in children.

**Material and Methods:** The international juvenile systemic scleroderma cohort (JSScC) database was queried for available patients with recorded PFT parameters and HRCT performed to determine sensitivity of PFTs detecting disease process.

**Results:** Of 129 patients in the jSScC, 67 patients had both CT imaging and an FVC reading from PFTs for direct comparison. DLCO readings were also captured but not in as many patients with tandem HRCT (n =55 DCLO and HRCT scan). Therefore, initial analyses focused on the sensitivity, specificity and accuracy of the FVC value from the PFTs to capture the diagnosis of interstitial lung disease as determined by HRCT.

Overall, 49% of the patients had ILD determined by HRCT, with 60% of patients having normal FVC (>80%) with positive HRCT findings, and 24% of patients having normal DLCO (> 80%) with positive HRCT findings. Fourteen percent (n = 3/21) of patients with both FVC and DLCO values within the normal range had a positive HRCT finding.

**Conclusions:** The sensitivity of the FVC in the JSScC cohort in detecting ILD was only 39%. Relying on PFTs alone for screening for ILD in juvenile systemic sclerosis would have missed the detection of ILD in almost 2/3 of the sample cohort, supporting the use of HRCT for detection of ILD in children with SSc. In addition, the cut off utilized, of less than 80% of predicted FVC or DLCO could be too low for pediatric patients to exclude beginning ILD. This pilot data needs confirmation in a larger patient population.

Supported by the Joachim Herz Stiftung.

## P.345

## HOW THE ADULT CRISS WORKS IN PEDIATRIC JSSC PATIENTS - RESULTS FROM THE JUVENILE SCLERODERMA INCEPTION COHORT

J. Klotsche^2^, I. Foeldvari^1^, O. Kasapcopur^3^, A. Adrovic^3^, K. Torok^3^, V. Stanevicha^3^, J. Anton^3^, R. Cimaz^3^, W.-. Sifuentes-Giraldo^3^, M.T. Terreri^3^, F. Sztajnbok^3^, C. Battagliotti^3^, L. Berntson^3^, D. Eleftheriou^3^, G. Horneff^3^, F. Nuruzzaman^3^, N. Helmus^1^

^1^*Hamburg Centre for Pediatric and Adolescence Rheumatology, Hamburg, Germany*, ^2^*German Rheumatism Research Center, Berlin, Germany*, ^3^*JSSc Collaborative Group, Hamburg, Germany*

**Introduction:** The Composite Response Index in Systemic Sclerosis (CRISS) was developed by Dinesh Khanna as a response measure in patients with adult systemic sclerosis. CRISS aims to capture the complexity of systemic sclerosis and to provide a sensitive measure for change in disease activity. The CRISS score is based on a two-step approach. First, significant disease worsening or new-onset organ damage is defined as non-responsiveness. In patients who did not fulfill the criteria of part one, a probability of improvement is calculated for each patient based the Rodnan Skin Score (mRSS), percent predicted forced vital capacity (FVC%), patient and physician global assessments (PGA), and the Health Assessment Questionnaire Disability Index (HAQ-DI). A probability of 0.6 or higher indicates improvement. The objective of this study was to validate the CRISS in a prospectively followed cohort of patients with juvenile systemic sclerosis (jSSc).

**Material and Methods:** Data from the prospective international inception cohort for jSSc was used to validate the CRISS. Patients with an available 12-months follow-up were included in the analyses. Clinically improvement was defined by the anchor question about improvement (much better or little better versus almost the same, little worse or much worse) in patients overall health due to scleroderma since the last visit provided by the treating physician.

**Results:** Forty seven jSSc patients were included in the analysis. 74.2% had diffuse subtype. The physician rated the disease as improved in 34 patients (72.3%) since the last visit. No patient had a renal crisis or new onset of left ventricular failure during the 12-months follow-up. Three patients (3.4%) each had a new onset or worsening of lung fibrosis and new onset of pulmonary arterial hypertension. In total, 6 patients resulted in a rating of not improved based on the CRISS in part I. The mRSSS, FVC%, CHAQ and PGA significantly improved during the 12-months follow-up in patients who were rated as improved. The predicted probability based on the CRISS algorithm resulted in an area under curve of 0.77 predicting the anchor question of improvement. In summary, 33 (70.0%) patients were correctly classified by the adult CRISS score resulting in an overall area under curve of 0.7.

**Conclusions:** The CRISS score was evaluated in a pediatric jSSc cohort for the first time. It showed a good performance. However, it seems that the formula of part II of the CRISS score needs a calibration to pediatric jSSc patients.

## P.346

## IS THE PRESENTATION AND SEVERITY DIFFERENT OF THE JUVENILE DIFFUSE AND LIMITED SUBTYPE SYSTEMIC SCLEROSIS? RESULTS OF JUVENILE SCLERODERMA INCEPTION COHORT

I. Foeldvari^1^, J. Klotsche^2^, O. Kasapcopur^3^, A. Adrovic^3^, K. Torok^3^, V. Stanevicha^3^, M.T. Terreri^3^, F. Sztajnbok^3^, E. Alexeeva^3^, J. Anton^3^, B. Feldman^3^, M. Katsikas^3^, V. Smith^3^, T. Avcin^3^, R. Cimaz^3^, M. Kostik^3^, T. Lehman^3^, W.-. Sifuentes-Giraldo^3^, N. Vasquez-Canizares^3^, N. Helmus^1^

^1^*Hamburg Centre for Pediatric and Adolescence Rheumatology, Hamburg, Germany*, ^2^*German Rheumatism Research Center, Berlin, Germany*, ^3^*JSSc Collaborative Group, Hamburg, Germany*

**Introduction:** Juvenile systemic scleroderma (jSSc) is an orphan disease with a prevalence of 3 in 1 000 000 children. There are limited data regarding the clinical presentation of jSSc. In the international juvenile systemic scleroderma cohort patients are followed with a standardized assessment prospectively. In the adult systemic scleroderma population, there are large differences regarding organ pattern and severity between diffuse and limited subtypes.

**Material and Methods:** Patients diagnosed according the ACR 2013 criteria for systemic sclerosis were included, if they developed the first non-Raynaud symptom before the age of 16 and were under the age of 18 at the time of inclusion. Patients’ characteristics at time of inclusion were evaluated.

**Results:** 131 patients were included, 72.5% with diffuse subtype. 75% females in the diffuse (djSSc) and 71% in the limited subtype (ljSSc). 86% of patients were Caucasian. Mean age of onset of Raynauds was 9.7 years in the djSSc and 10.7 years in the ljSSc (p=0.8). Mean age of onset of the non-Raynauds was 9.9 years in the djSSc and 11.2 years in the ljSSc (p=0.7). Mean disease duration at time of inclusion was 3.4 years in the djSSc and 2.4 years in the ljSSc There was no significant difference in the ANA, anti-Scl-70 and anticentromere positivity. The mean modified skin score was significantly higher in the djSSc (17.3 compared 7.1, (p=0.3)). They were significantly more telangiectasia in the djSSc group (39% compared to 19% (p=0.003)). Cardiac involvement was significantly higher in the ljSSc group (19% compared to 3% (p=0.005) There was no significant difference in the proportion of ILD, pulmonary hypertension, gastrointestinal involvement and renal involvement. No renal hypertension was observed. There was significantly more muscle weakness observed in the ljSSc group (38% compared to 17% (p=0.029)). There was no significant difference regarding number of joints with contractions. Physician rated disease activity (40 compared to 29, on 100 mm VAS scale (p=0.013)) and disease damage (37 compared 18, on a 100 mm VAS scale (p<0.001)) was significantly higher in the djSSc. This significant difference was not found in rating of patients of disease damage and activity.

**Conclusions:** ljSSc and djSSc seems to be more similar than in adult patient with these subtypes, although physician rating of disease activity and damage found the djSSc more severe.

Supported by the Joachim Herz Stiftung

## 14. Sclerosing skin diseases (Localized scleroderma)

## P.347

## A CASE REPORT OF MICROSURGICAL ALAR RECONSTRUCTION USING VASCULARIZED HELICAL RIM FLAPS FOR LOCAL SCLEROSIS PATIENT

H. T. Xiao

*West China Hospital, Chengdu, China*

**Introduction:** People with local sclerosis have areas of skin that become hard and leathery. Eventually, tissue atrophy occurs and the skin becomes highly colored. We met a 19-year-old female with nasal defects caused by local sclerosis, alar and dorsum were involved.

**Material and Methods:** We reconstructed the alar defect with free transfer of vascularized helical rim flap, filled the volume of the dorsum with dermis-fat graft.

**Results:** Acceptable appearance has been achieved with 1.5 year follow up.

**Conclusions:** It’s a useful method to treat the deformity.

## P.348

## LOW DOSE NALOXONE FOR THE MANAGEMENT OF PRURITUS IN PATIENTS WITH SCLERODERMA

I. Vigdorchick^1^, J. Berookhim^1^, Y. Levy^2^

^1^*Meir Medical Center, Kfar Saba, Israel*, ^2^*Sackler Faculty of Medicine, Tel Aviv University, Tel Aviv, Israel*

**Introduction:** Systemic Sclerosis (SSc) is an autoimmune connective tissue disease that leads to widespread fibrosis. Cutaneous features include thickening of the skin that can transition to sclerodactyly with obliteration of hair follicles and sweat glands. Pruritus is a common symptom in SSc and has been shown to affect many important aspects of an individual’s quality of life. Although pruritus has been seen to be very common in patients with SSc, there is no clear guidelines how to manage this unpleasant symptom.

In previous case series investigators noted the use of naloxone in many other GI diseases. This was the rationale of the use of low dose naloxone, an opioid antagonist with anti-oxidative properties for the pruritus in patients with scleroderma.

**Material and Methods:** In this case series there were four patients with SSc with sever pruritus. All patients were females between the ages of 31 and 74. Three patients were of the diffuse cutaneous SSc and one patient was with limited SSc. Each of these patients had complaints of severe pruritus. Each patient was started on 4.5 mg of PO Naloxone once daily, and their degree of pruritus was followed with the 5D-itch scale. All patients were monitored for side effects.

**Results:** All patients were started on 4.5 mg of PO Naloxone once daily. Within one week all patients reported drastic improvement in the level of pruritus. There were no side effects seen with the use of Naloxone at this dose.

**Conclusions:** We report that our patients showed significant improvement in their pruritus with the use of low-dose naloxone. All patient showed drastic reduction within the first week. All patients also reported less disturbances and improvement in sleep, leisure/social activities, housework/errands, and work/school. Low-dose naloxone is an inexpensive drug that should be used in management of pruritus in patients with scleroderma.

## P.349

## SCLEREDEMA ADULTORUM OF BUSHKE IS ASSOCIATED WITH A HIGHER PREVALENCE OF STROKE AND DYSLIPIDEMIA COMPARED TO DIABETICS WITHOUT SCLEREDEMA

C. Varju^1^, V. Csonka^1,2^, B. Bódis^3^, N. Farkas^4^, D. Kovács^5^, L. Czirják^1^

^1^*Medical School, University of Pécs - Department of Rheumatology and Immunology, Pécs, Hungary*, ^2^*Teaching Hospital Mór Kaposi - Department of Internal Medicine, Pécs, Hungary*, ^3^*Medical School, University of Pécs - 1st Department of Internal Medicine, Pécs, Hungary*, ^4^*Medical School, University of Pécs - Institute of Bioanalysis, Pécs, Hungary*, ^5^*Medical School, University of Pécs - Department of Vascular Surgery, Pécs, Hungary*

**Introduction:** The aim of the study is to screen diabetic patients for the presence of scleredema adultorum of Buschke and to characterize the clinical-laboratory findings of the newly identified cases.

**Material and Methods:** Out of the 113 screened diabetic patients, 11 new scleredema, all with type2 diabetic cases were found. Their clinical-laboratory data were compared to the rest of screened diabetics as well as to another cohort of 15 type2 dabetic scleredema patients already treated in our tertiary clinical centre.

**Results:** Stroke was more frequently found in the case history of both the newly identified scleredema cases (3/11; 27.3%) and in our cohort (6/15; 40.0%), compared to the diabetics without scleredema skin symptoms (6/102; 5.9%; /p=0.021; p<0.001, respectively/).

More patients with hypertriglyceridemia, dyslipidemia (p<0.05), increased mean level of non-HDL-cholesterol and alanine aminotransferase (ALT) in the sera were defined (p<0.01) in both groups with scleredema, compared to diabetics without such skin disorder.

Based on binary logistic regression the high level of non-HDL-cholesterol (OR 3.338, CI 1.77-6.28; p<0.001) and taking insulin therapy (OR 7.64, CI 1.99-29.34; p=0.003) were found to be independent predictors for presence scleredema skin disorder in patients with diabetes mellitus.

In the group of 64 statin-user patients, the non-HDL-cholesterol and triglyceride levels were also higher (P<0.05) in both the newly diagnosed and the formerly treated scleredema groups (n=9 in both) compared to the diabetics without scleredema (n=46).

**Conclusions:** Scleredema was associated with a higher serum level of non-HDL-cholesterol, increased prevalence of dyslipidaemia and stroke compared to diabetics without skin disorder. Besides poorly controlled diabetes requiring insulin therapy, high non-HDL-cholesterol level may also be a risk factor to the development of scleredema.

## P.350

## COEXISTENCE OF SYSTEMIC SCLEROSIS AND MORPHEA

A. Vanhaecke^1,2^, L. Heeman^1,2^, H. Callens^1^, M. Cutolo^3^, F. De Keyser^1,2^, S. De Schepper^4^, S. Nguyen^5^, J. Gutermuth^5^, V. Smith^1,2,6^

^1^*Department of Interal Medicine, Ghent University, Ghent, Belgium*, ^2^*Department of Rheumatology, Ghent University Hospital, Ghent, Belgium*, ^3^*Research Laboratory and Academic Division of Clinical Rheumatology, Dept. of Internal Medicine, Univ. of Genoa, Genoa, Italy*, ^4^*Department of Dermatology, Ghent University Hospital, Ghent, Belgium*, ^5^*Department of Dermatology, Brussels University Hospital, Brussels, Belgium*, ^6^*Unit for Molecular Immunology and Inflammation, VIB Inflammation Research Center (IRC), Ghent, Belgium*

**Introduction:** Scleroderma covers a group of rare autoimmune fibrosing disorders with analogue histopathological findings, divided into localized sclerosis (LoS), which primarily affects the skin and systemic sclerosis (SSc), which can affect both skin and internal organs. SSc and LoS are clinically distinct diseases, due to absence of Raynaud’s phenomenon (RP), scleroderma pattern on nailfold videocapillaroscopy (NVC), SSc-specific antibodies, sclerodactyly and specific organ manifestations in LoS. As the relation of SSc to LoS remains controversial, we critically appraised literature on the coexistence of SSc and LoS and performed an additional chart review in a large consecutive cohort of SSc patients.

**Material and Methods:** Original studies documenting the coexistence of SSc and LoS were identified, by means of a systematic literature search in PubMed, EMBASE and Web of Science, according to the PRISMA guidelines. Additionally, the coexistence of SSc and LoS was studied in a consecutive cohort of SSc patients who had their yearly follow-up visit in 2018 in the Ghent University Scleroderma Unit (GUSU).

**Results:** A total of 3317 unique records were identified through the systematic search of which 16 manuscripts were eligible for full-text review. Finally, 9 studies were included for quality appraisal and data extraction [1-9]. The concomitant presence of SSc and LoS ranged between 0.13% and 10.5% [1-9]. RP was present in nearly all concomitant cases [1-3,6,7]. A scleroderma pattern on NVC was reported in 2/2 concomitant cases by Maricq and in 6/8 concomitant cases by Dilia [1,2]. SSc-specific antibodies tended to be commonly observed in concomitant cases, ranging from 22.2% to 75% [1,2,6,7].

Additionally, the clinical records of 344 consecutive SSc patients of the GUSU were reviewed, of which 6 patients presented both SSc and LoS (1.7%). RP was present in all concomitant cases. A scleroderma pattern on NVC was observed in 5/6 concomitant cases. SSc-specific antibodies (i.e. cenp-B) were only present in 1/6 concomitant cases.

**Conclusions:** This is the first systematic literature review with additional chart review to investigate the coexistence of SSc and LoS. A higher overlap of SSc and LoS compared to the normal population was revealed, which is peculiar for two rare diseases, especially considering their prevalence in the normal population.

**Reference(s)**

1. Dilia G. Dermatol Res Pract.2018;2018:1284687.

2. Maricq H. Arch Dermatol.1992;128(5):630-2.

3. Dehen L. Medicine.1994;73(5):241-5.

4. Lipsker D. Clin Exp Rheumatol.2015;33(4 Suppl 91):S23-5.

5. Peterson LS. J Rheumatol.1997;24(1):73-80.

6. Soma Y. Dermatology.1993;186(2):103-5.

7. Toki S. J Dermatol.2015;42(3):283-7.

8. Uziel Y. Semin Arthritis Rheum.1994;23(5):328-40.

9. Zulian F. Arthritis Rheum.2005;52(9):2873-81.

## P.351

## EFFECTIVENESS OF TOPICAL SODIUM TIOSULFATE FOR THE TREATMENT OF CALCINOSIS-ASSOCIATED CUTANEOUS ULCERS IN PATIENTS WITH SYSTEMIC SCLEROSIS

I. Torner Hernández^1^, A. Sendra García^2^, V. Nuñez Monje^2^, L. Montolio Chiva^1^, A.V. Orenes Vera^1^, I. Vazquez Gomez^1^, E. Flores Fernández^1^, M. Aguilar Zamora^1^, E. Valls Pascual^1^, A. Martinez Ferrer^1^, D. Ybañez García^1^, J.J. Alegre Sancho^1^

^1^*Servicio de Reumatología. Hospital Universitario Doctor Peset, Valencia, Spain*, ^2^*Fundación para el fomento de la investigación sanitaria y biomédica de la Comunidad Valenciana (FISABIO), Valencia, Spain*

**Introduction:** Treatment of calcinosis associated with cutaneous systemic sclerosis (cSS) mainly involves the use of systemic therapies, which have often limited efficacy. But little attention has been paid to local treatment, which is especially useful when associated with skin ulcers.

**Material and Methods:** Descriptive analysis of a case series of patients with cSS who present with calcinosis-associated skin ulcers.

Wound management procedure: wounds and perilesional skin cleaning and disinfection is performed, with additional debridement and optional exeresis, if needed. TST is compounded, at 25% as w/o emulsion, for extensive calcinosis, or as beeler-base or cold-cream ointment, for limited calcinosis. Wounds are covered with a polymeric foam dressing. This cure in moist healing environment shows some advantages over the dry cure (exudate control without damaging the periulceral skin, protection against contamination, healing time, pain and number of needed cures reduction).

**Results:** 8 patients (6 women) with calcinosis-associated skin ulcers and cSS were included: 2 patients with diffuse cSS (DcSSc), 5 with limited (LcSSc) and 1 with overlap syndrome. Median age was 62 years (IQR 21). 5 patients had localized wounds and 3 had extensive involvement and/or tumor calcinosis. They had been refractory to systemic treatment with diltiazem, colchicine, zoledronate, rituximab, and/or acenocumarol and suffered recurrent superinfections.

Follow-up results of more than 3 months are available for 4 patients. Time on TST treatment was up to one year. They have shown clinical improvement (disappearing of most calcifications, wound healing, pain, function and quality of life improvement, together with an increase in patient satisfaction). Radiological improvement was also shown in 1 case.

Non TST related adverse effect has been detected, except for wound edges slight maceration, for the ointment preparation, which was resolved by protecting these with zinc oxide cream.

**Conclusions:** In our experience, treatment with TST for calcinosis-associated skin ulcers in patients with cSS is an effective, safe and easily implementable therapeutic alternative in clinical practice.

## P.352

## CORRELATION OF EXPRESSION OF TLR7 AND MIRNA-146A GENES AND FIBROSIS IN PERIPHERAL BLOOD AND SKIN SAMPLES OF SSC PATIENTS

V. Spasovski^1^, M. Vreca^1^, M. Andjelkovic^1^, M. Stojiljkovic^1^, A. Skakic^1^, K. Klaassen^1^, A. Zekovic^2^, N. Damjanov^2^, S. Pavlovic^1^

^1^*Institute of Molecular Genetics and Genetic Engineering, University of Belgrade, Belgrade, Serbia*, ^2^*Institute of Rheumatology, Clinical Center of Serbia, Belgrade, Serbia*

**Introduction:** Introduction Systemic sclerosis (SSc) is a heterogeneous multisystem autoimmune disease of unknown etiology. It is clinically characterized by progressive fibrosis of the skin and internal organs. Accumulative evidence demonstrates that Toll-like receptors (TLRs) may represent the link between immune activation and tissue fibrosis. Releasing of endogenous TLR ligands during inflammation and local tissue damage and their subsequent binding to TLRs possibly complexed to autoantibodies might be one of the mechanisms that initiate and drive autoimmunity and subsequent fibrosis. On the other hand, recent findings point out that miR-146a might have a role in ‘fine-tuning’ regulation of the TLR/NF-kB signaling pathway through down-regulation of IRAK1 gene. The aim of the present study was to analyze the expression level of TLR7 and miR-146a genes and their relevance for clinical presentation of SSc patients.

**Material and Methods:** Here we examined the expression of TLR7 and miRNA-146a genes in peripheral blood mononuclear cells of 50 SSc patients and 13 healthy individuals using RT-qPCR technique. Additionally, we examined their expression in skin biopsies of five SSc patients using paired tissue samples with severe fibrotic changes and without fibrotic changes of the same patient.

**Results:** In tissue samples the expression of TLR7 gene was 56% lower in samples with severe fibrotic changes (mRSS score>10) compared to samples without fibrotic changes of the same patient. When peripheral blood samples were examined, we found that patients with severe skin involvement (mRSS score>10) showed 26% lower TLR7 expression compared to patients with mild skin involvement (mRSS score<10).

In addition, 19% lower level of miR-146a expression was detected in skin biopsies of the patients with severe fibrous changes compared to samples without fibrotic changes of the same patient. In peripheral blood samples, 37% lower expression of miR-146a was detected in patients with severe skin involvement compared to patients with mild skin involvement.

**Conclusions:** Here we investigated TLR7 signaling in skin and in peripheral blood samples of SSc patients. Comparison showed that both TLR7 and miR-146a genes show synchronized expression in tissue samples and blood samples. Therefore, we suggest both of these molecules should be further investigated as noninvasive biomarkers for skin involvement in SSc patients. A better understanding of the mechanisms of TLR-mediated pathogenesis and therapies targeting individual TLRs, may provide a more specific approach of treating this multi-systemic autoimmune disease.

**Figure:**
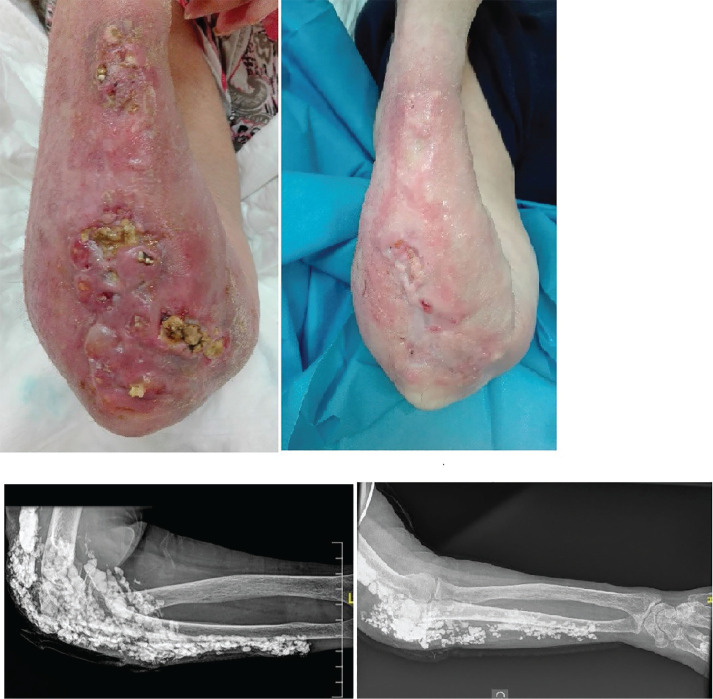


## P.353

## TOCILIZUMAB AS TREATMENT FOR CUTANEOUS INVOLVEMENT IN SYSTEMIC SCLEROSIS: A SERIES OF 3 CASES

F. Ortiz-Sanjuán, C. Alcañiz, I. Cánovas, I. Chalmeta, M. De La Rubia, J. Fragio, R. González, L. González, E. Grau, S. Leal, I. Martínez, C. Nájera, R. Negueroles, C. Pávez, J. Oller, E. Vicens, J. Ivorra, J.A. Román-Ivorra

*Hospital Universitario y Politécnico La Fe-Rheumatology Department, Valencia, Spain*

**Introduction:** Cutaneous involvement in systemic sclerosis (SSc) is often difficult to treat and the evidence in support of many of the usual therapies is limited. Interleukin-6 (IL-6) is involved in SSc pathogenesis and has shown a direct fibrotic effect. Tocilizumab (TCZ) is a recombinant humanized monoclonal antibody against the IL-6 receptor that has shown promising results for cutaneous involvement of SSc.

Our aim was to assess tocilizumab for cutaneous involvement in real-life patients diagnosed of SSc.

**Material and Methods:** Observational study of a wide and unselected series of patients classified as SSc following 2013 ACR/EULAR classification criteria for SSc which presented active diffuse cutaneous involvement. Patients were treated with TCZ due to diffuse cutaneous scleroderma.

**Results:** A total of 3 patients with diffuse cutaneous scleroderma were studied. Two of the patients were women and one man. The mean age was 44.2±6.4 years (range 34-52). The mean time since diagnosis to TCZ beginning was 1,3 months and the mean time since first non-Raynaud symptom was 3,4 years. The 3 patients showed a median modified Rodnan skin score (MRSS) of 31.6 points (range 44-25). Two of the three patients were previously refractory to methotrexate. TCZ treatment regimen employed was 8 mg/Kg/week intravenous therapy in 2 patients and 162 mg/week subcutaneous therapy in the remaining patient.

In all patients there was an improvement of cutaneous involvement. After a mean follow-up of 6.4±2.8 (range 5-9) months during TCZ therapy none of the patients presented any new relapse of cutaneous involvement or MRSS progression.

One of the patients had to discontinue TCZ in the first term due to digital skin ulcer infection and later by drug induced liver injury.

**Conclusions:** TCZ seems to be a great option in real-life for those patients with diffuse cutaneous involvement due to SSc.

## P.354

## IL-16 EXPRESSION IS INCREASED IN THE SKIN AND SERA OF PATIENTS WITH SYSTEMIC SCLEROSIS

T. Makino, K. Makino, S. Sawamura, S. Shimada, T. Yamashita, M. Hayashi, T. Miyamura, S. Ishimatsu, H. Ihn

*Faculty of Life Sciences, Kumamoto University, Kumamoto, Japan*

**Introduction:** Systemic sclerosis (SSc) is an autoimmune disease with chronic and persistent inflammation in its pathogenesis. Levels of interleukin-16 (IL-16) are found to be significantly elevated in the plasma of SSc patients. In this study, we have examined the expression pattern of IL-16 in SSc involved skin, the serum concentration of IL-16 in SSc patients and the relationship between serum IL-16 levels and the disease activity.

**Material and Methods:** Using immunohistochemical analysis, we examined the quantity and localization of IL-16 in affected skin obtained from SSc patients. We also measured serum levels of IL-16 in SSc patients using an enzyme-linked immunosorbent assay. We then validated the correlation between the serum IL-16 levels and clinical symptoms in patients with SSc.

**Results:** In the skin, Il-16 was expressed on the lymphocytes around the capillaries. Furthermore, the ratio of IL-16-positive cells was statistically higher in patients with diffuse cutaneous (dc) SSc than in those with limited cutaneous (lc) SSc patients (43.9% vs. 29.1%, p<0.05). The serum IL-16 levels in SSc patients were statistically significant elevated compared to healthy controls (297.0 pg/mL vs. 194.9 pg/mL, p<0.05). Increased serum IL-16 levels in SSc patients were correlated with ratio of dcSSc, skin score and the presence of cutaneous symptoms of erythema and pigmentation.

**Conclusions:** The regional up-regulation of IL-16 in the skin is not only associated with skin sclerosis, but also with systemic IL-16 activation. IL-16 may play a role in the pathogenesis of SSc. Moreover, serum IL-16 levels may be useful as a biomarker for determining the severity of the skin sclerosis. Inhibiting IL-16 activation may be effective in treating SSc.

## P.355

## LOCO-REGIONAL TREATMENT OF DIGITAL ULCERS IN SYSTEMIC SCLEROSIS: A SYSTEMATIC REVIEW

I. Costedoat^1^, M. Masson^2^, T. Barnetche^2^, E. Lazaro^3.4^, P. Duffau^3.5^, C. Richez^2.3^, J. Seneschal^1^, M.-E. Truchetet^2.3^

^1^*Department of Dermatology and Paediatric Dermatology, National Reference Center for skin diseases, Saint André Hospital, Bordeaux, France*, ^2^*Department of Rheumatology, Pellegrin Hospital, Bordeaux, France*, ^3^*Immunology Laboratory, ImmunoConcept, UMR CNRS 5164, University Hospital of Bordeaux, bordeaux, France*, ^4^*Department of Internal Medicine, Haut-Lévêque, University Hospital of Bordeaux, Pessac, France*, ^5^*Department of Internal Medicine, Saint-André Hospital, University of Bordeaux, Bordeaux, France*

**Introduction:** Digital ulcers (DUs) are common, affecting 50% of patients with systemic sclerosis (SSc). The management of DUs is based on a combination of local and systemic treatments. The latter are well codified on the 2017 EULAR recommendations. This therapeutic approach is partially efficient: two-thirds of patients with new ulcers will recur within 16-24 weeks and no drug has demonstrated a positive effect on refractory DUs healing. This therapeutic loophole has led physicians to develop multiple local-regional treatments for which the levels of evidence are less well known than for conventional treatments. In this context, we conducted a systematic review to summarize the effectiveness of local-regional treatments on DUs in SSc

**Material and Methods:** A systematic review in the Pubmed and Scopus databases from the earliest records to February 2019 was conducted. We included randomized and non-randomized controlled trials, prospective and retrospective studies, case series and case report. Articles were included if they presented results of treatment of digital ulcers in patients with systemic sclerosis and mixed connective tissue disease with a scleroderma phenotype. Outcomes were the healing time of DU, the number of DUs and the pain improvement using the VAS scale.

**Results:** The literature search identified 793 citations and 90 studies were included. The techniques for which we found the most data are: local implantation of autologous progenitor cells, injections of fat-derived cells, botulinum toxin and sympathectomy.

Injections of fat-derived cells have shown on a RCT a decrease in pain and ulcer number. These results were explained by a partial restoration of the capillary bed in the digits. Botulinum toxin also has two RCTs with contrasting results, particularly on pain. Finally, hyperbaric oxygen therapy and blood-derived cell injections showed a decrease in pain and ulcer number, but only in studies with a low level of evidence. Sympathectomy showed contradictory results with frequent recurrences of DUs associated with high amounts of side effects.

Literature on iontopheresis, phototherapy, intermittent compression, extracorporeal shockwave, vitamin E gel and topical dimethyl sulfoxide, diltiazem and glyceril trinitrate was too limited to draw strong conclusions.

**Conclusions:** This systematic review summarizes the complexity of loco-regional interventions proposed for the management of DUs in SSc. Innovative procedures using adipose tissue-derived cells or hand injection of botulinum toxin showed promising results that will need to be confirmed in future clinical trials. The main limitation of our study is the low number of RCTs.

**Figure:**
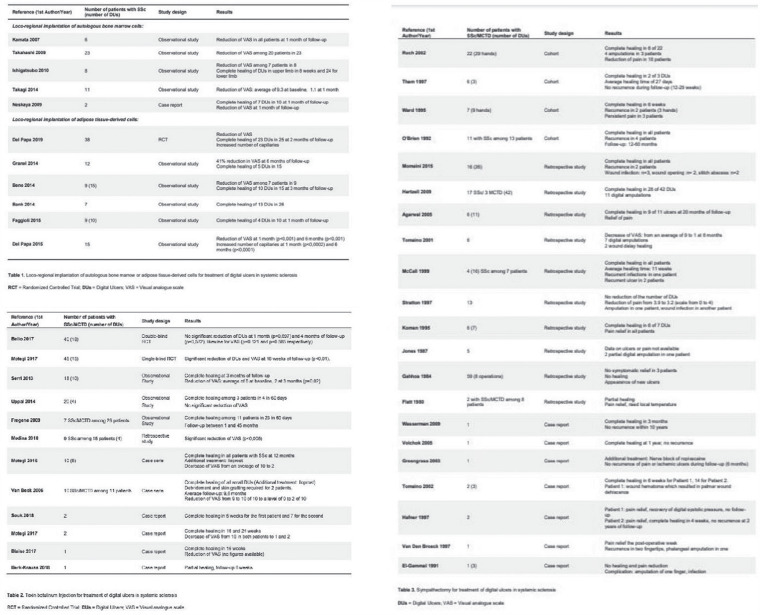


## P.356

## ANALYSIS OF HISTOPATHOLOGICAL CUTANEOUS ALTERATIONS IN SYSTEMIC SCLEROSIS

E. Cocchiara^1^, A. Spinella^1^, E. Veronesi^3^, M. Pignatti^4^, F. Lumetti^1^, L. Magnani^2^, G. Bajocchi^2^, G. De Santis^5^, C. Salvarani^1^, M. Dominici^6^, D. Giuggioli^1^

^1^*Rheumatology Unit, University of Modena and Reggio Emilia, Policlinico of Modena, Italy, Modena, Italy*, ^2^*Rheumatology Unit, AUSL-IRCCS of Reggio Emilia, Italy, Reggio Emilia, Italy*, ^3^*Technology Park for Medicine Mario Veronesi of Mirandola, Mirandola, Italy*, ^4^*Department of Specialized Medicine, Diagnostic and Experimental, University of Bologna, Bologna, Italy*, ^5^*Department of Plastic and Reconstructive Surgery, University of Modena and Reggio Emilia, Policlinico of Modena, Ital, Modena, Italy*, ^6^*Department of Maternal-child and Adult Medical and Surgical Sciences, Modena, Italy*

**Introduction:** Systemic sclerosis (SSc) is an autoimmune disease characterized by an excessive production of collagen resulting in the skin and connective tissue thickness.

The aim of this study was to identify SSc histopathological cutaneous alterations by Hematoxylin and Eosin (HE), Orcein (O) and Picrius Sirius Red staining (PSR).

**Material and Methods:** We evaluated 8 samples of skin from SSc patients and 1 sample of skin from healthy donor.

HE was used to identify morphological features of skin biopsies, O assured the contrast between the elastic fibers and other types of connective fibers while PSR permitted to identify collagen fibers.

**Results:** We found that, independently of the anatomical site of the biopsies, the mean skin thickness differed significantly between SSc biopsies and healthy donors.

We evaluated cellular recruitment: an abundant presence of lymphocytes was detected in 2 patients while in 3 biopsies, a mild distribution of immune cells.

O showed the disruption of elastic fibers in all affected biopsies.

By PSR we found a different pattern of collagen fibers in papillary and in reticular dermis of the pathological samples.

In papillary dermis, as in healthy donors, we observed the collagen fibers run parallel to the epidermis in 3/8 patients.

In 5/8 patients fibers were irregular and fragmented. In reticular dermis 2 patients had reticular fibers like the control, 6 patients presented with an altered pattern (collagen fibers are irregular and fragmented).

Evaluating the pattern of collagen fibers on the total dermis we found a significantly statistical increase in Sirius red positive fibers in 7 biopsies. Polarized light microscopy added information on the structure of collagen: All SSc patients had a significant decrease of larger fibers and 6/8 had an increase of thinner fibers.

**Conclusions:** In our work, we presented the histopatological cutaneous alterations in SSc and we detected skin thickness and fragmentation of elastic fibers in all patients with a fragmentation of the network from the reticular dermis.

An abnormal collagen pattern was found in all patients and it correlates with skin strength, that is increased in SSc.

Morevover, a high percentage of lymphocytes could correlate with the first stage of the disease.

All patients showed a decrement of synthesis of larger collagen fibers whereas no homogenous behavior was detected in distribution of thinner ones.

All of these parameters could help the clinician on SSc diagnosis.

Further studies are needed in order to make a correlation between clinical data and skin alteration phases.

## P.357

## EOSINOPHILIC FASCIITIS, A MONOCENTRIC RETROSEPCTIVE STUDY OF 30 PATIENTS

M. Thiebaut^1^, B. Chaigne^1^, N. Dupin^1^, O. Mangin^1^, P. Cohen^1^, J. London^1^, A. Regent^1^, N. Costedoat-Chalumeau^1^, C. Lejeunne^1^, F.-J. Authier^1^, L. Mouthon^1^

*Department of Internal Medicine, Systemic Autoimmune Diseases Reference Center of Ile de France, Cochin University Hosp, Paris, France*

**Introduction:** To describe clinical, histological features, treatments and outcome of 30 patients with eosinophilic fasciitis (EF).

**Material and Methods:** We reviewed the files from 30 patients with EF followed in the Internal Medicine and/or Dermatology departments of Cochin hospital. Clinical and histological data, treatment regimens and outcome were collected.

**Results:** Thirty patients were included of age at diagnosis of 52 [37; 61] years old, median [IQR] and follow-up of 63[36; 107] months. At the time of diagnosis, 28 patients (93 %) had skin sclerosis, 20 (67 %) had edema and 19 (63 %) had weight loss and 6 (20%) had morphea associated. Hypereosinophilia was present in 22 (73 %) of patients. Fascia biopsy was performed in 26 (87 %) patients with inflammatory infiltrates in all of patients and interstitial myositis in 7/17 (41 %). Muscle MRI was performed in 19 (63 %) patients, showing fascia hypersignal in 14/19 (74 %). All patients except one received corticosteroids (CS) and 14 (47%) developed CS dependence. Twenty-two patients (73 %) received methotrexate (MTX), 7 patients (32 %) as first line treatment in combination with CS, 14 (64 %) patients after CS initiation and 6 (27 %) after failure of another treatment. Under MTX, 14 (64 %) patients had weaning of CS therapy, 3 (14 %) were in remission and 14 (64 %) improved.

**Conclusions:** This retrospective study highlights importance of early initiation of CS sparing treatment in patients with EF. MTX seems to be the most effective immunosuppressant in this indication.

**Figure:**
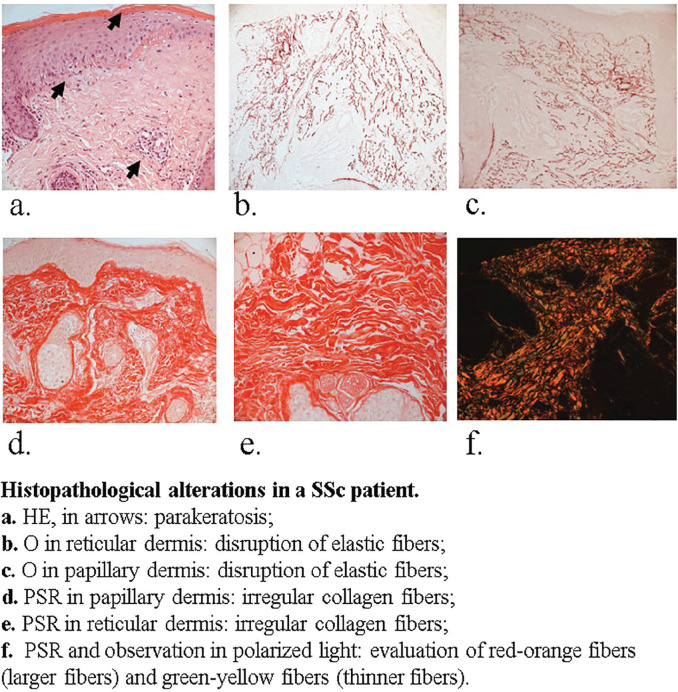


## P.358

## PROTEOMIC AND TRANSCRIPTOMIC ANALYSIS OF HUMAN EOSINOPHILIC FASCIITIS FIBROBLASTS

B. Chaigne, C. Fernandez, M. Le Gall, P. Chafey, B. Saintpierre, V. Salnot, F. Guillonneau, C. Le Jeunne, L. Mouthon

*INSERM U1016, Institut Cochin, Paris, France*

**Introduction:** Eosinophilic fasciitis (EF) is a rare scleroderma-like disorder with less than 200 cases reported. Due to the rarity of the disease, data regarding its pathophysiology are lacking. Herein we aimed at studying the transcriptome and the proteome of EF fibroblasts.

**Material and Methods:** Skin fibroblasts from EF patients (n=4), systemic sclerosis (SSc) patients (n=4), and healthy controls (HC) (n=4), were cultured and harvested for transcriptomic and proteomic analysis. Transcriptomic and proteomic analyses were performed next generation sequencing and using label-free quantification, respectfully. Functional analyses were performed using Ingenuity Pathway Analysis and Panther softwares.

**Results:** Five hundred and nine genes involved in 14 biological processes (BP) and 199 pathways were differentially expressed between EF and control groups. Among them, the circadian clock system (CCS) (p=1.70 10-9), the plasminogen-activating cascade (PAC) (p=3.82 10-5), the FGF signalling pathway (p=1.15 10-4), and the integrin signalling pathway (p=1.33 10-3) were overrepresented. When comparing EF to HC genes, Panther showed overrepresented down-regulated pathway including CCS (p=1.71 10-12), and overrepresented up-regulated pathways such as flavin biosynthesis, (p=9.22 10-4), PAC (p=1.19 10-6), de novo purine biosynthesis (p=1.63 10-3), FGF signalling pathway (p=1.47 10-3) and integrin signalling pathway (p=9.14 10-4). When comparing EF to SSc genes Panther showed overrepresented down-regulated pathway such as CCS (p=2.51 10-4).

Two hundred and twenty four proteins involved in 10 BP and 126 pathways were differentially expressed between EF and control groups. When comparing EF to HC proteins, Panther showed specific overrepresented up-regulated biological processes such as vesicle-mediated transport (p=4.66 10-5) and intracellular protein transport (p=1.24 10-4), and overrepresented up-regulated pathways such as 5-hydroxytryptamine degradation (5-HTD) (p=1.56 10-4) and overrepresented down-regulated pathways such as heterotrimeric G-protein signalling pathways-rod outer segment photo-transduction (HTGSP) (p=1.24 10-4). When comparing EF to SSc proteins, Panther showed specific overrepresented down-regulated biological processes such as 5-HTD (p=4.48 10-4), and overrepresented down-regulated pathways such as HTGSP (p=1.98 10-4), and ubiquitin proteasome pathway (p =1.05 10-3).

**Conclusions:** This work described the transcriptome and proteome of EF fibroblasts and highlighted significant specificities of EF fibroblasts when compared to HC and SSc.

## P.359

## CHARACTERIZATION OF SCLERODERMA PATIENTS ACCORDING TO RO52 AND KU ANTIBODIES

J.P. Caballero Castro^1^, A. Guillén Del Castillo^2^, E. Callejas Moraga^3^, D. Colunga Argüelles^4^, L. Sáez Comet^5^, M. Rubio Rivas^6^, A. Argibay^7^, J.A. Vargas Hitos^8^, J.A. Todolí Parra^9^, I. Perales Fraile^10^, C. Tolosa Vilella^3^, A. Marín Ballvé^11^, A.B. Madroñero Vuelta^12^, M.E. Sánchez García^13^, N. Ortego-Centeno^14^, G. Espinosa^15^, M. Rodríguez Carballeira^16^, V. Fonollosa Pla^2^, C.P. Simeón Aznar^2^, On Behalf of RESCLE investigators^17^

^1^*Department of Internal Medicine. Hospital Obispo Polanco, Teruel, Spain*, ^2^*Unit of Autoimmune Diseases, Department of Internal Medicine. Hospital Universitario Vall d’Hebron, Barcelona, Spain*, ^3^*Department of Internal Medicine. Corporación Sanitaria Universitaria Parc Taulí, Sabadell, Barcelona, Spain*, ^4^*Department of Internal Medicine. Hospital Universitario Central de Asturias, Oviedo, Asturias, Spain*, ^5^*Department of Internal Medicine. Hospital Universitario Miguel Servet, Zaragoza, Spain*, ^6^*Unit of Autoimmune Diseases, Department of Internal Medicine. Hospital Universitario de Bellvitge-IDIBELL, L’Hospitalet de Llobregat, Barcelona, Spain*, ^7^*Unit of Systemic Autoimmune Diseases and Thrombosis. Department of Internal Medicine. Complejo Hospitalario Universitari, Vigo, Pontevedra, Spain*, ^8^*Department of Internal Medicine. Hospital Universitario Virgen de las Nieves, Granada, Spain*, ^9^*Department of Internal Medicine. Hospital Universitario y Politécnico La Fe, Valencia, Spain*, ^10^*Department of Internal Medicine. Hospital Universitario Rey Juan Carlos, Móstoles, Madrid, Spain*, ^11^*Unit of Autoimmune Diseases, Department of Internal Medicine. Hospital Clínico Universitario Lozano Blesa, Zaragoza, Spain*, ^12^*Department of Internal Medicine. Hospital General San Jorge, Huesca, Spain*, ^13^*Department of Internal Medicine. Hospital Universitario Virgen de Valme, Sevilla, Spain*, ^14^*Inst Invest Biosanitaria Ibs Granada. Department of Internal Medicine, Unit of Systemic Autoimmune Diseases. Department, Granada, Spain*, ^15^*Department of Autoimmune Diseases. Hospital Clinic, Barcelona, Spain*, ^16^*Department of Internal Medicine. Hospital Universitario Mútua Terrassa, Terrassa, Barcelona, Spain*, ^17^*Autoinmune Diseases Study Group (GEAS), Spain*

**Introduction:** Systemic Sclerosis (SSc) is an autoimmune disease that is characterized by progressive and severe fibrosis with cutaneous and visceral involvement, fibroproliferative vasculopathy and alterations of cellular and humoral immunity, very heterogenous from the clinical and immunological point of view. There are many types of antibodies related to the disease that are used in the diagnosis and characterization.

Antibodies anti-Ku and anti-Ro52 have been found, although they are not specific to the disease, that could play an important role in the prognosis and clinical expression. To evaluate the clinical pattern and prognosis of SSc patients who carry antibodies Anti-Ku and Anti-Ro52.

**Material and Methods:** A retrospective, multicentric study of SSc patients included in the Spanish Registry of Scleroderma (RESCLE). Clinical, demographic, prevalence, serological, and survival data were analyzed.

**Results:** A total of 401 samples were analyzed for anti-Ku with 12 positive results (3%). For anti-R052, 1724 sampled were analyzed with 246 positive results (14%).

Anti-Ku positive patients presented a higher prevalence of myositis, but without differences in multivariant analysis.

Regarding anti-Ro52 positive patients, they had in the univariate analysis a higher frequency of ILD, sicca syndrome and coexistence with Rheumatoid factor, anti-Ro60, anti-La and anti-RNP antibodies. However, in the multivariant analysis only remained an association with sicca syndrome (OR 2.3, p<0.001) and its coexistence with Rheumatoid factor, anti-Ro60, anti-La and anti-RNP antibodies (OR 1.8; OR 44.8 and OR 3.7, respectively).

**Conclusions:** Patients with anti-Ku antibodies had a higher frequency of myositis, although this was not confirmed in multivariate analysis. Patients with anti-Ro52, had an association with ILD, although after multivariate analysis was only confirmed a correlation with sicca syndrome and Rheumatoid factor, anti-La and with anti-RNP antibodies.

**Figure:**
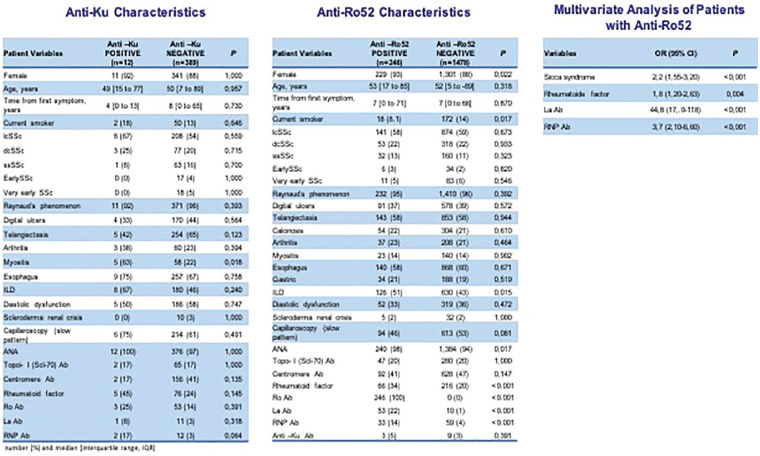


## P.360

## POTENTIAL BIOMARKERS OF SKIN CHANGES IN SYSTEMIC SCLEROSIS

R. Becvar, H. Storkanova, B. Sumova, M. Spiritovic, S. Oreska, L. Senolt, M. Tomcik

*Institute of Rheumatology, Prague, Czech Republic*

**Introduction:** Skin fibrosis is a hallmark of systemic sclerosis (SSc). There are no widely accepted biomarkers of skin involvement in this condition. Several serum or plasma markers have been studied in patients with SSc - monocyte chemoattractant protein-1 (MCP-1), chemokine (C-X-C motif) ligand 8 (CXCL8), interleukin-13 (IL-13), and some more recognized such as - platelet derived growth factor (PDGF), transforming growth factor-beta 1 (TGF-beta 1), epidermal growth factor (EGF) and basic fibroblast growth factor (bFGF).

The aim of this study was to assess several circulating biomarkers which may be relevant to the fibrosing process and further to correlate the obtained data with clinical indicators specific for SSc skin involvement.

**Material and Methods:** 59 SSc patients (M/F 9/50; mean age 52.1 years, mean disease duration 6.7 years, 36 patients with limited cutaneous SSc and 23 with diffuse cutaneous SSc. As a control group 36 healthy individuals matched to sex and age were examined. Serum concentrations of bFGF, granulocyte-colony stimulating factor (G-CSF), granulocyte-macrophage-colony stimulating factor (GM-CSF), MCP-1, PDGF, IL-8 and 13 were analysed using commercial multiplex kit. The following clinical examinations were performed: modified Rodnan skin score (mRSS), Hand Mobility in Scleroderma Test (assessing hand function) (HAMIS), Cochin Hand Function Scale (hand function) (CHFS), Delta Finger-to-Palm Distance (extension-flexion) (dFTP), Inter-lip Distance (inter-lip), Inter-incisor Distance (inter-incisor), and Mouth Handicap in Systemic Sclerosis Scale (mouth opening) (MHISS). For statistical evaluation Spearman’s correlation coefficient was used.

**Results:** When compared with healthy controls serum concentrations of bFGF (p<0.001), G-CSF (p<0.0001), GM-CSF (p<0.0001), MCP-1 (p<0.0001) IL-8 (p<0.0001), and IL-13 (p<0.001) were significantly elevated in SSc cohort . PDGF levels were increased in SSc patients with only a lower significance (p<0.01). bFGF, G-CSF, MCP-1 and IL-8 levels correlated significantly (p<0.05) with mRSS and HAMIS. GM-CSF levels correlated with mRSS and HAMIS and there was only a trend for negative correlation with inter-incisor. The was no correlation of IL-13 and PDGF levels with the evaluated clinical data.

**Conclusions:** Our results have shown that G-CSF, GM-CSF and IL-8 play a substantial role in SSc fibrosing process. Potential biomarkers as bFGF, G-CSF, MCP-1 and IL-8 correlated with a few clinical indices of SSc skin involvement.

**Acknowledgement:** This study was supported by research grants AZV 16-33574A and AZV 16-33542A.

## P.361

## PREVALENCE AND ASSOCIATES OF CANCER IN A COHORT OF PATIENTS WITH SYSTEMIC SCLEROSIS ATTENDING A SINGLE CENTRE

A. Agnihotri, J. Manning, S. Wilkinson, M. Samaranayaka, A.L. L Herrick

*University of Manchester, Manchester, United Kingdom*

**Introduction:** The link between cancer and systemic sclerosis (SSc) has recently attracted considerable interest, although the pathophysiological processes underpinning this link remain poorly understood. The aim of this study was to evaluate the prevalence (and types) of cancer in a cohort of patients with SSc attending a single centre, and to compare the demographic and clinical features, and autoantibody status (including anti-RNA polymerase), in those with and without a cancer diagnosis.

**Material and Methods:** This was a retrospective study of a cohort of 413 patients attending a single specialist centre for SSc. To be included, patients had to have had at least one visit to the SSc clinic in the last 10 years. Patients with SSc were identified from a dedicated clinical database. Demographic, clinical, serological and treatment-related data were recorded from the database and from the electronic patient record, and results compared between those with and without a cancer diagnosis.

**Results:** Thirty-four patients (8.2%) had a recorded diagnosis of cancer, with 39 malignancies within this group. Breast cancer (n = 10) was the most prevalent followed by melanoma (n = 8), lung (n = 5) and hematologic (n = 5) malignancies. The age of onset of Raynaud’s phenomenon was higher in patients with cancer compared to without: mean (standard deviation) 50 ± 16.4 years vs 44 ± 16.6 years (p = 0.044). Age of onset of first non-Raynaud’s clinical manifestation did not reach statistical significance: 56 ± 11.9 years in those with cancer vs 51 ± 14.5 years in those without (p = 0.051). Barrett’s oesophagus was over-represented in the cancer group: 5 (14.7%) with cancer vs 19 (5.1%) in those without (p = 0.022). Fewer patients in the cancer group (3 [8.8%]) had had treatment with mycophenolate mofetil than those in the non-cancer group (52 [13.8%]) (p = 0.003). No differences between groups were identified in age, gender, disease subtype (limited versus diffuse cutaneous), and autoantibody status.

**Conclusions:** In this retrospective study, the prevalence of reported cancer was 8.2% in patients with SSc. There was a significant association between the age of onset of RP and the diagnosis of cancer. Further studies are required to confirm the positive association with Barrett’s oesophagus and the negative association with current or previous treatment with mycophenolate mofetil.

## 15. Emerging therapies (pre-clinical)

## P.362

## AN ORALLY AVAILABLE HIGHLY SELECTIVE 5-HYDROXYTRYPTAMINE 2B (5-HT2B) RECEPTOR ANTAGONIST AMELIORATING PULMONARY AND DERMAL FIBROSIS IN PRECLINICAL MODELS OF SYSTEMIC SCLEROSIS

H. Arozenius, L. Pettersson, G. Ekström, C. Wenglén

*AnaMar AB, Lund, Sweden*

**Introduction:** Serotonin or 5-hydroxytryptamine (5-HT) is well known as a stimulator of tissue fibrosis and a significant role of peripheral 5-HT2B receptors in fibrosis has been suggested with the receptor being upregulated in fibrotic tissues. In addition, agonism of the 5-HT2B receptor has been implicated in human tissue fibrosis caused by drugs known to activate the receptor. Pharmacologic inhibition of 5-HT2B receptor signaling consequently represents a promising treatment strategy for fibrotic disorders including systemic sclerosis. The pro-fibrotic effects of 5-HT and the 5-HT2B receptor are believed to be mediated through activation of the TGF/Smad signaling pathway. The objektives of the current study were to investigate effects of a selective 5-HT2B receptor antagonist on fibrosis development and on potential biomarkers.

**Material and Methods:** The murine sclerodermatous chronic graft versus host disease (scGvHD) and the tight-skin-1 (Tsk-1) model were used to evaluate anti-fibrotic effects of the 5-HT2B receptor antagonist, AM1476. Oral, once and twice daily treatment with AM1476 was applied day 21 to 49 in the scGvHD model. Pulmonary fibrosis was evaluated using hydroxyproline content, Sirius Red staining and Ashcroft score. Dermal thickness, myofibroblast counts, collagen production and number of M2 macrophages were used to evaluate dermal fibrosis. In the Tsk-1 model AM1476 was orally administered once or twice daily from week 5 to week 10. Hypodermal thickening, myofibroblast counts and hydroxyproline content in skin biopsies were evaluated at the end of the treatment period. The number of phosphorylated Smad3 (pSmad3) positive cells in mice dermal tissue was used to evaluate inhibition of TGF signaling. To evaluate effects on collagen-production, human dermal fibroblasts were induced with 5-HT and TGF and treated with AM1476.

**Results:** The 5-HT2B receptor antagonist AM1476, significantly reduced all measured dermal and pulmonary fibrosis readouts in the scGvHD model using an oral therapeutic treatment approach. Therapeutic treatment of dermal fibrosis in the Tsk-1 model effectively and significantly reduced hypodermal thickening, number of myofibroblast and hydroxyproline content. The number of pSmad3 positive cells were significantly reduced in skin samples isolated from treated animals. Collagen production was significantly decreased in human cells

**Conclusions:** Inhibition of 5-HT2B receptor activity resulted in anti-fibrotic effects in pulmonary and dermal fibrotic tissues. Reduction of pSmad3 and collagen production was observed and will be evaluated as potential biomarkers in upcoming clinical trials. The highly selective 5-HT2B receptor antagonist AM1476, represents a promising drug candidate for treatment of fibrotic conditions and is currently in early development for systemic sclerosis.

## P.363

## HSP90 INHIBITOR 17-DMAG REDUCES PROGRESSION OF DERMAL FIBROSIS AND INDUCES REGRESSION OF ESTABLISHED EXPERIMENTAL DERMAL FIBROSIS IN MICE INDUCED BY BLEOMYCIN

H. Storkanova^1,2^, L. Storkanova^1^, S. Oreska^1,2^, M. Spiritovic^3^, B. Hermankova^3^, R. Becvar^1,2^, K. Pavelka^1,2^, J. Vencovsky^1,2^, J. Distler^4^, L. Senolt^1,2^, M. Tomcik^1,2^

^1^*Institute of Rheumatology, Prague, Czech Republic*, ^2^*Department of Rheumatology, 1st Faculty of Medicine, Charles University, Prague, Czech Republic*, ^3^*Faculty of Physical Education and Sport, Department of Physiotherapy, Charles University, Prague, Czech Republic*, ^4^*Department of Internal Medicine III and Institute for Clinical Immunology, University of Erlangen-Nuremberg, Erlangen, Germany*

**Introduction:** Our previous study demonstrated that Heat shock protein 90 (Hsp90) is overexpressed in the skin of patients with systemic sclerosis (SSc), in cultured SSc fibroblasts and preclinical models of SSc. HSP90 is a new regulator of canonical TGF-β signalling and its inhibition prevents the stimulatory effects of TGF-β on collagen synthesis and dermal fibrosis in three preclinical models of SSc. Herein, we aimed to evaluate the efficacy of Hsp90 inhibitor (17-DMAG) in the treatment of established experimental dermal fibrosis induced by bleomycin.

**Material and Methods:** Design consisted of three control groups, I (NaCl-s.c./6 weeks), II (bleomycin-s.c./3w and NaCl-s.c./3w), III (bleomycin-s.c./6w), and 2 treatment groups (bleomycin-s.c./6w). During the last 3 weeks, one group was treated with 17-DMAG 0.5mg/kg-i.p. every third day, whereas one group (with nintedanib 50mg/kg-p.o. twice daily) served as a comparator with already published efficacy in this setting. Total of 40 BL6 mice were examined weekly for weight, activity and fur texture. The effects of 17-DMAG were determined by assessment of dermal thickness (HE-staining), collagen content (hydroxyproline assay), myofibroblast counts (α-SMA staining) and of 23 serum inflammatory cytokines/chemokines (Mouse-Cytokine-23-plex, Bio-Rad-Laboratories).

**Results:** 17-DMAG decreased dermal thickening by 53±3% (p<0.001) (nintedanib by 46±2%,p<0.001), collagen content by 48±5% (p=0.004) (nintedanib by 50±4%,p=0.003), myofibroblast counts by 42±9% (p<0.001) (nintedanib by 44±7%,p<0.001), and levels of IL-1α, IL-6, IL-12(p40), CXCL1, MCP-1, MIP-1α/β, RANTES (in all: p<0.05) compared to vehicle-treated mice injected with bleomycin for 6w. Moreover, 17-DMAG also induced regression of pre-established fibrosis to below the levels of vehicle-treated mice injected with bleomycin for 3w and NaCl for 3w (dermal thickness by 14±3%, collagen content by 20±5%, myofibroblast counts by 13±9%; whereas in nintedanib by 10±3%, 21±4%, 17±7%, respectively; in all: p<0.05), and levels of IL-12(p40), CXCL1, MCP-1, MIP-1β, RANTES (in all: p<0.05). No significant weight loss, decrease in activity or changes in fur texture were observed upon 17-DMAG treatment.

**Conclusions:** This is the first study on effects of Hsp90 inhibitor 17-DMAG in the treatment of established dermal fibrosis. We demonstrate that 17-DMAG effectively prevents the progression and induces regression of established bleomycin-induced dermal fibrosis, in an extent that was comparable to nintedanib in this study (which was recently FDA approved for slowing the rate of decline in lung function in adults with SSc-ILD). 17-DMAG was well tolerated without obvious clinical signs of toxicity. These data suggest that Hsp90 could be a novel potential target in the treatment of SSc dermal fibrosis.

**Acknowledgement:** Supported by AZV-16-33542A, MHCR023728, SVV260373, Boehringer Ingelheim.

## P.364

## VITAMIN D DOWNREGULATES PRO-FIBROTIC MOLECULES ASSOCIATED WITH SYSTEMIC SCLEROSIS: ALPHA-SMA AND PSMAD2/3

A. Rodrigues De Almeida^1^, M.E. De Oliveira Gonçalves^1^, E. Gustavo Constantino Cunha^1^, J.V. De Melo Gomes^1^, A. Tavares Dantas^2^, R. Silva Guimarães Gonçalves^2^, A. Luzia Branco Pinto Duarte^2^, M.J. Barreto De Melo Rêgo^1^, M. Cristiny Pereira^1^, M. D. Lima de Oliveira^1^, M. Galdino Da Rocha Pitta^1^

^1^*Laboratório de Imunomodulação e Novas Abordagens Terapêuticas, Núcleo de Pesquisa em Inovação Terapêutica Suely Galdino, Recife-PE, Brazil*, ^2^*Hospital das Clínicas de Pernambuco, Federal University of Pernambuco, Recife-PE, Brazil*

**Introduction:** Systemic sclerosis (SSc) is a complex autoimmune connective tissue disease characterized by vasculopathy, immune dysregulation preceding tissue fibrosis in the skin and internal organs. Fibrosis follows microvascular/endothelial damage with fibroblast activation and differentiation into myofibroblasts, key effector cell in SSc, which express Alpha-Smooth Muscle Actin (Alpha-SMA) and increase the persistent ability to synthesize and accumulate extracellular matrix proteins. Smad2/3 are key players in canonical TGFb signaling which form a complex with co-Smad4 leading to transcriptional regulation of target genes, including those encoding extracellular matrix proteins such as collagen I and III. Studies have shown the immunomodulatory and antifibrotic properties of vitamin D, justified by the wide expression of the vitamin D receptor (VDR) in different cell types. In this context, the present study aimed to evaluate the antifibrotic activity of vitamin D in immortalized skin and lung fibroblasts.

**Material and Methods:** To evaluate this activity we used the HFF-1 (ATCC® SCRC-1041™; passage 43) and MRC-5 (ATCC® CCL-171™; passage 33) fibroblast cell lines derived from skin and lung, respectively. Cells were plated at a density of 1 x 104 cells on glass coverslips in 24-well plates and then stimulated with recombinant TGF-beta1 (10ng/mL) and treated or not treated with vitamin D (100nM). After 48 hours in a CO2 (37°C 5% CO2) incubator, expression of Alpha-SMA and pSmad2/3 markers were evaluated by immunofluorescence assay with monoclonal anti-Alpha-SMA conjugated to eFluor 570® (1:150; eBioscienceTM) and polyclonal anti-pSmad2/3 (1:100; eBioscience™) antibodies followed by IgG goat anti-rabbit antibody conjugated to Cyanine Cy™3 (1:300; Jackson Immunoresearch™).

**Results:** The outcomes show a reduction in both expressions of Alpha-SMA and pSmad2/3 in healthy skin (HFF-1) and pSmad2/3 in lung (MRC-5) fibroblasts by vitamin D (100nM).

**Conclusions:** Considering the important role played by Alpha-SMA and pSmad2/3 molecules in fibrotic process development, vitamin D might be a possible therapeutic agent for SSc.

## P.365

## CADHERIN 11 AND COLLAGEN I IN A SCLERODERMA FIBROTIC MODEL AND EFFECT OF MECHANOSENSING AND INFLAMMATORY INHIBITORS ON THEIR EXPRESSION

A. Mujkanovic, T. Lim, X. Shiwen, B. Ahmed Abdi, G. Martin, H. Lopez, D. Abraham, C. Denton, R. Stratton

*Universtiy College London, Royal Free Campus, London, United Kingdom*

**Introduction:** Introduction: In current understanding of pathogenesis of fibrosis a central role is taken by CD206+ M2 macrophages and myofibroblasts with their consequential crosstalk, this crosstalk being characterized through creation of adhesion junctions and subsequent increased synthesis of collagen. Adhesion junction between cells are partially achieved by cadherin 11. Cadherin-11 has been reported as increased in the skin and cells in SSc afflicted individuals, and it has been suggested as a possible fibrotic cell marker. The expression of cadherin-11 and collagen may be partly controlled by mechanosensing pathways MRTF-A and TGF-beta. Novel inhibitors of MRTF-A pathway CCG-257081, and a peptide inhibitor of CD206+, RP832 could hinder the expression of both, cadherin-11 and collagen and inhibit the crosstalk between these two cell types. The aim was to estimate the effect of culturing healthy/SSc macrophages and SSc-derived fibroblasts on 50kPa gels on collagen and cadherin-11 expression and test whether inhibitors of mechanosensing and inflammatory signaling hinder the expression of cadherin-11 and collagen in the co-culture model.

**Material and Methods:** Material and methods: 50 kPa 12-well plates were used for co-culture of SSc fibroblasts with healthy or SSc-derived macrophages and treated with RP832 and CCG inhibitors (both 10µM) separately as well as both together. The cell layer was processed for later qPCR or Western Blots analysis. Mice MRTF-A-/- macrophages were analyzed using NGS. Skin tissue sections of healthy and those affected with SSc were analyzed using IHC for CDH11.

**Results:** Results: Expression of cadherin and collagen was higher in co-culture model than in fibroblasts culture alone (P<0.001). The relative mean expression of CDH11 mRNA in the SSc group of co-cultures was smaller than in control healthy group (P<0.001). Both RP and CCG inhibitor suppressed CDH11 in the SSc co-culture. The RP832 inhibitor gave no statistically relevant effect on the mRNA expression in the healthy control group (P<0.034). Western blots analysis was mostly in line with findings obtained from qPCR, but no statistical significance was recorded; in addition, higher immunoreactivity confirmed qPCR and Western Blots results.

**Conclusions:** Conclusion: SSc Fibroblasts and healthy macrophages interact through CDH11 but less so in SSc macrophages. The higher relative expression of CDH11 seen in healthy individuals indicates that this factor may not be relevant as a marker of fibrosis in late SSc. The effect of RP832 on the macrophages derived from SSc group and lack of effect on macrophages derived from healthy individuals further support its potential use as a therapy in fibrosis.

## P.366

## VASCULARIZED HUMAN SKIN EQUIVALENTS AS A NOVEL IN VITRO MODEL OF SKIN FIBROSIS AND PLATFORM FOR TESTING OF ANTIFIBROTIC DRUGS

A.-E. Matei^1^, C.-W. Chen^1^, L. Kiesewetter^2^, A.-H. Györfi^1^, Y.-N. Li^1^, T. Trinh-Minh^1^, X. Xu^1^, T.M. Cuong^1^, T.H. van Kuppevelt^3^, J. Hansmann^2,4^, A. Jüngel^5^, G. Schett^1^, F. Groeber-Becker^2,6^, J.H. Distler^1^

^1^*Department of Internal Medicine 3Rheumatology and Immunology, Friedrich-Alexander-University Erlangen-Nuremberg, Erlangen, Germany*, ^2^*Fraunhofer Translational Center Regenerative Therapies, Fraunhofer Institute for Silicate Research ISC, Würzburg, Germany*, ^3^*Radboud University Medical Center, Radboud Institute for Molecular Life Sciences, Department of Biochemistry, Nijmegen, The Netherlands*, ^4^*University for Applied Sciences Würzburg-Schweinfurt, Würzburg, Germany*, ^5^*Department of Rheumatology, University Hospital Zurich, Zurich, Switzerland*, ^6^*Department for Tissue Engineering and Regenerative Medicine, Würzburg University Medical Center, Würzburg, Germany*

**Introduction:** Fibrotic diseases such as systemic sclerosis develop as a consequence of complex pathophysiological process involving interplay between multiple cell types. Experimental modelling of fibrosis is essential for understanding its pathogenesis and for testing candidate antifibrotic drugs. However, most current models employ either phylogenetically relatively distant species or rely on human cells cultured in an artificial two-dimensional environment, with forced, unphysiological cell polarization and attachment to a substrate much stiffer than the human skin. Here we evaluated the potential of vascularized in vitro human skin equivalents as a novel model of skin fibrosis and a platform for the evaluation of antifibrotic drugs.

**Material and Methods:** Skin equivalents were generated by seeding endothelial cells, fibroblasts and keratinocytes of human origin on a decellularized, three-dimensional extracellular matrix of porcine origin with intact, perfusable vessel structure. The skin models were cultured for one month in a system that ensured perfusion of the vascular network at physiological pressure. Fibrotic transformation by exposure to TGFbeta and response to treatment with nintedanib as an established antifibrotic agent was evaluated by qPCR, capillary Western immunoassays, immunostaining and histology.

**Results:** Skin equivalents formed a mature, polarized epidermis, a stratified dermis and a functional vessel system including perfused small capillaries. Exposure of the skin equivalents to TGFbeta lead to their fibrotic transformation with replication of key features of fibrotic skin: activation of TGFbeta downstream pathways, fibroblast-to-myofibroblast transition and excessive deposition of extracellular matrix. Treatment of TGFbeta-exposed models with nintedanib, a drug that previously showed antifibrotic effects in both preclinical and clinical settings, ameliorated the fibrotic transformation of skin equivalents with attenuation of TGFbeta signaling, reduced fibroblast-to-myofibroblast transition and decreased deposition of extracellular matrix.

**Conclusions:** We present an in vitro test system for skin fibrosis, generated by adapting a previously described vascularized skin equivalent. Our data provide evidence that vascularized skin equivalents can reproduce all functional skin layers affected by fibrosis, that by exposure to TGFbeta these models recapitulate key features of fibrotic skin and that they may serve as a platform for the evaluation of antifibrotic drugs in a setting relevant for human pathophysiology.

**Figure:**
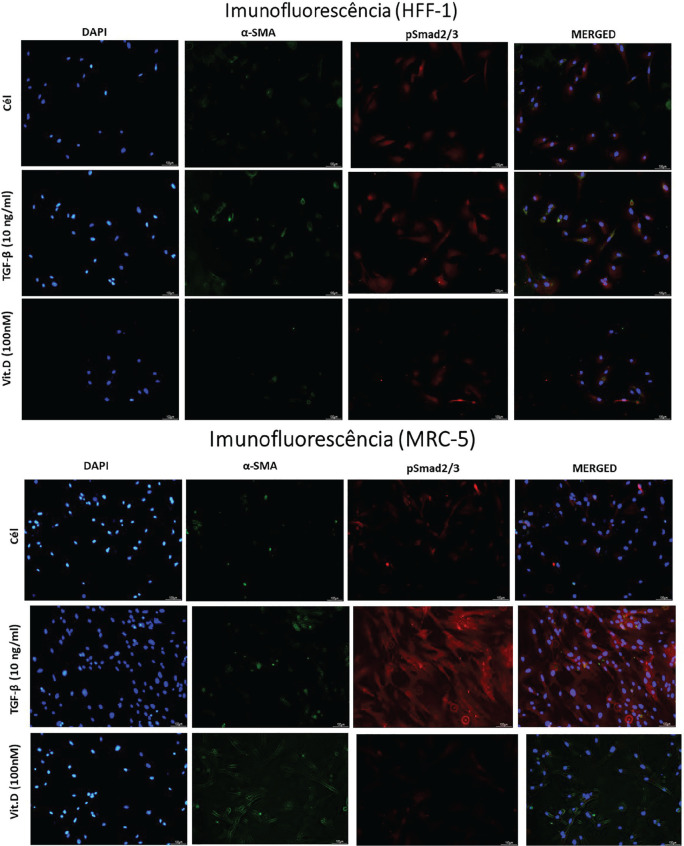


**Figure:**
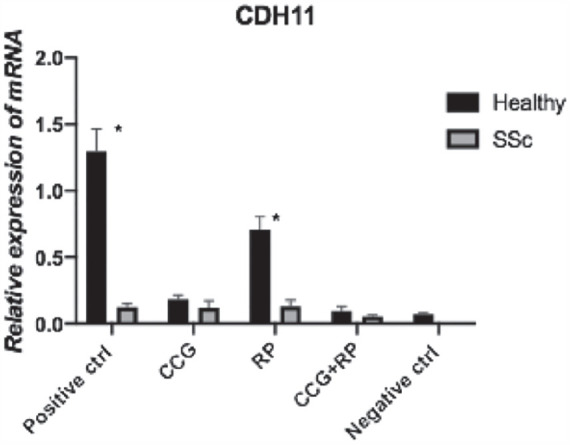


## P.367

## IMPACT OF JAK INHIBITORS ON MACROPHAGE POLARISATION: PERSPECTIVES FOR SYSTEMIC SCLEROSIS

A. Lescoat^1^, A. Ballerie^1^, M. Lelong^2^, C. Morzadec^2^, S. Jouneau^3^, P. Jego^1^, L. Vernhet^4^, O. Fardel^5^, V. Lecureur^4^

^1^*CHU Rennes, Internal medicine and clinical immunology and IRSET UMR 1085, Rennes, France*, ^2^*IRSET UMR 1085, Rennes, France*, ^3^*CHU Rennes, Pneumology and respiratory disorders and IRSET UMR 1085, Rennes, France*, ^4^*Rennes University and IRSET UMR 1085, Rennes, France*, ^5^*CHU-Rennes, Biology and IRSET UMR 1085, Rennes, France*

**Introduction:** Macrophage can adopt various phenotypes and activation states according to their surrounding microenvironment. M1 or inflammatory macrophages are generated under IFNg/LPS signalling and express the membrane marker CD86. Different subtypes of M2 macrophages are also described: M2a macrophages (generated under IL4/IL13 signalling) and characterized by a high expression of CD206 and pro-fibrotic properties and, M2c macrophages (generated under IL10 and/or glucocorticoid signalling), considered as anti-inflammatory resolving macrophages. There is a growing interest in the role of macrophages in the pathogenesis of Systemic Sclerosis (SSc). Recent studies highlight that macrophages from fibrotic tissues such as lung or skin from SSc patients have an M2 phenotype whereas, in blood-monocytes derived macrophages (MDM), SSc MDM have a mixed signature associating M1 and M2 characteristics. Jak inhibitors are treatments used in rheumatoid arthritis and that can variously target signals that could be involved both in M1 and in M2 polarization. This study evaluates in vitro the impact of three Jak inhibitors on the polarisation state of human MDM.

**Material and Methods:** Blood monocytes from healthy donors (HD) were differentiated with M-CSF (for 7 days) in MDM and pre-treated by ruxolitinib (Jak2-Jak1 inhibitor), tofacitinib (Jak3 inhibitor) or itacitinib (Jak1 inhibitor) (1µM for all). They were then polarised into M1i (IFNg, 20µg/mL), M1Li (IFNg+LPS, 20µg/mL), M2a (IL4+IL13; 20µg/ML), M2c (IL10, 20µg/mL) and M2c(dex) (IL10+dexamethasone, 10 nM). The impact of each Jak inhibitor on phenotype (flow cytometry), gene expression (qPCR) and cytokine secretion (ELISA) was evaluated in each polarization state.

**Results:** Concerning phenotypes, all Jak inhibitors reduced the expression of the M1i and M1Li marker CD86, but ruxolitinib had a higher effect. Only ruxolitinib reduced the expression of the M1i marker MHCII. All Jak inhibitors reduced the expression of CD206 in M2a. They had no impact on the expression of CD163, CD204 in any M2 conditions. Key M1 genes were repressed by all Jak inhibitors, such as CXCL10, IL6 or TNFa with a more significant effect of ruxolitinib. All Jak inhibitors reduced the gene expression of CXCL13 and SOCS3 in M2c. Secretion levels of IL6 and CCL18 were also repressed, with a more significant effect of ruxolitinib

**Conclusions:** Jak inhibitors can limit M1 and M2 polarisation state in vitro, with a more significant effect of the Jak2-Jak1 inhibitor ruxolitinib in vitro. The relevance of these results in MDM from SSc patients and in in vivo models of SSc are still to be determined.

## P.368

## SECUKINUMAB AND METFORMIN AMELIORATE BLEOMYCIN INDUCED DERMAL FIBROSIS

C. Celik^1^, A. Karatas^2^, B. Oz^2^, Z.A. Akar^2^, E.O. Etem^3^, A.F. Dagli^4^, S.S. Koca^2^

^1^*Department of Internal Medicine, Firat University, Faculty of Medicine, Elazig, Turkey*, ^2^*Department of Rheumatology, Firat University, Faculty of Medicine, Elazig, Turkey*, ^3^*Department of Medical Biology, Firat University, Faculty of Medicine, Elazig, Turkey*, ^4^*Department of Pathology, Firat University, Faculty of Medicine, Elazig, Turkey*

**Introduction:** Although the pathogenesis of systemic sclerosis is not exactly known, it is thought that immune activation has prominent roles in pathogenesis. Secukinumab is monoclonal antibody against interleukin (IL)-17A. Metformin, a widely used anti-diabetic medication, has anti-proliferative, immunomodulating and anti-fibrotic activities.

The purpose of our study is to determine the therapeutic efficacy of secukinumab and metformin on bleomycin (BLM) induced dermal fibrosis.

**Material and Methods:** 50 Balb/c female mice were divided into 5 groups: (group 1 [control], 2 [sham], 3 [secukinumab], 4 [metformin] and 5 [secukinumab+metformin]). The mice in the control group received 100 µl phosphate buffered saline (PBS), while the mice in other groups received 100 µl (100 µg) BLM in PBS subcutaneously (sc) every day for 4 weeks. In addition, mice in groups 3 and 5 received secukinumab at a dose of 10 mg/kg/week sc, and mice in the groups 4 and 5 received oral metformin (50 mg/kg/day) for 28 days. All groups of mice were sacrificed at the end of 4th week and tissue samples were taken for analysis. In addition to histopathological analysis, skin tissue mRNA expressions of IL-17 and collagen 3A were measured by real-time PCR.

**Results:** Repeated BLM injections had caused dermal fibrosis (Fig. 1, Table 1). In addition, mRNA expressions of IL-17 and collagen 3A were increased in the BLM group. Secukinumab and metformin ameliorated dermal fibrosis. They decreased dermal thickness and IL-17A and collagen 3A mRNA levels (Fig. 2).

**Conclusions:** Secukinumab and metformin exhibit anti-fibrotic effects in the BLM induced dermal fibrosis.

## P.369

## BOTULINUM TOXIN IN THE MANAGEMENT OF RAYNAUD’S PHENOMENON

D. Ennis^1-5^, Z. Ahmad^1,2,4^, K. Devakandan^1,2,3^, M. Anderson^6^, S. Johnson^1-4^

^1^*Toronto Scleroderma Program, Toronto, Canada*, ^2^*Mount Sinai Hospital, Department of Medicine, Division of Rheumatology, Toronto, Canada*, ^3^*Toronto Western Hospital, Department of Medicine, Division of Rheumatology, Toronto, Canada*, ^4^*University of Toronto, Toronto, Canada*, ^5^*University of British Columbia, Vancouver, Canada*, ^6^*University Health Network Library Services, Toronto General Hospital, Toronto, Canada*

**Introduction:** The objectives of this study were to evaluate the effectiveness and safety of botulinum toxin injection in Primary and systemic sclerosis (SSc) associated Raynaud’s phenomenon

**Material and Methods:** Medline, Embase, Cochrane Central Register of Controlled Trials, ClinicalTrials.gov and CINAHL databases were searched to identify eligible studies. Studies reporting use of botulinum toxin, effectiveness measures and safety outcomes were included.

**Results:** 421 patients with Raynaud’s were identified of which 202 (48%) had SSc. Botulinum toxin A doses ranged from 10-200 units, administered with similar frequency to the palm (n=272) and digits (n=253). The mean duration of benefit was 6.28 months (range: 1 month to 8.6 years). The most common adverse event was intrinsic muscle weakness (32 total events, frequency of 7.6%), and pain in 2 subjects. Only a single subject experienced persistent muscle weakness and atrophy. A single death was reported 3 days after botulinum toxin use, but was not attributed to the treatment. Outcome measures included the Raynaud’s Condition Score, QuickDASH, MCSS, LDI, patient acceptable symptom state, and duration of benefit.

**Conclusions:** The evidence for botulinum toxin in the treatment of primary and SSc-associated Raynaud’s phenomenon is promising. Consistency across patient populations, treatment options (botulinum serotype, dose, injection site) and outcome measures will be essential for further research.

**Fig 1.**
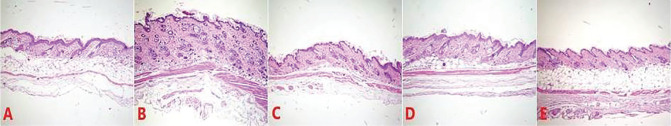
Histopathological sections of skin in the study groups (hematoxylin eosin, ×100) A: Control group, B: Bleomycin (Sham) group, C: Secukinumab group, D: Metformin group and E: Secukinumab+metformin group

**Figure:**
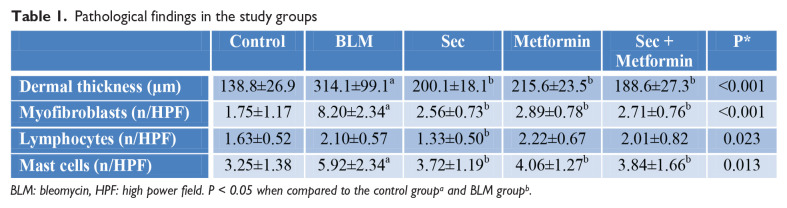


**Fig 2.**
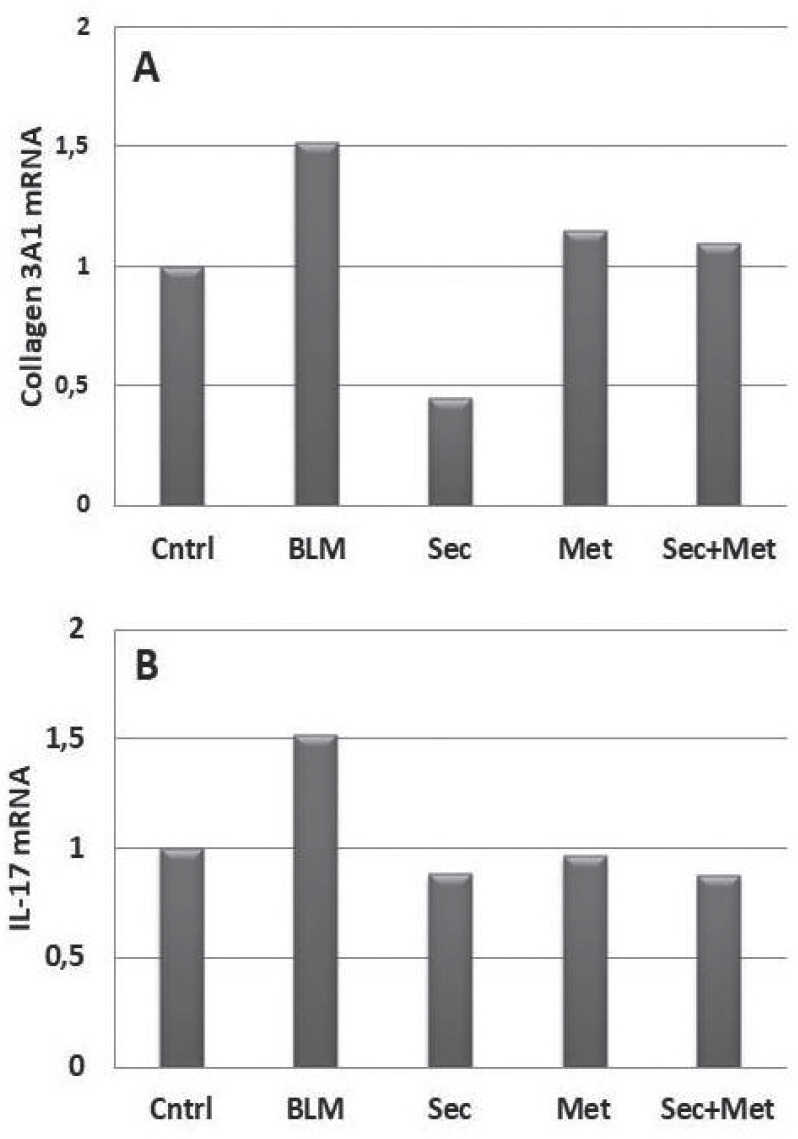
Tissue collagen 3A1 mRNA (A) and IL-17 mRNA (B) expressions in the study groups mRNA: messenger ribonucleic acid, IL: interleukin, Cntrl: Control group, BLM: bleomycin, Sec: secukinumab, Met: metformin

## P.370

## TRAINED IMMUNITY MODULATES INFLAMMATION-INDUCED FIBROSIS

M.M. Jeljeli^1,5^, L. Gama Coelho Riccio^2^, L. Doridot^1^, C. Chêne^1^, C. Nicco^1^, S. Chouzenoux^1^, Q. Deletang^1^, Y. Allanore^3^, N. Kavian^4^, F. Batteux^1,5^

^1^*3I Department, Institut Cochin, INSERM U1016, Université Paris Descartes. Sorbonne Paris Cité., Paris, France*, ^2^*Disciplina de Ginecologia, Departamento de Obstetrícia e Ginecologia, Universidade de Sao Paulo, Sao Paulo, Brazil*, ^3^*Assistance Publique Hôpitaux de Paris, Centre Hospitalier Universitaire (CHU) Cochin, Service de rhumatologie., Paris, France*, ^4^*Hong Kong University, Pasteur Research Pole, School of Public Health, The University of Hong Kong, Hong Kong, China., Hong Kong, Hong Kong*, ^5^*Assistance Publique Hôpitaux de Paris, Centre Hospitalier Universitaire (CHU) Cochin, Laboratoire d’immunologie., Paris, France*

**Introduction:** Chronic inflammation and fibrosis result from inappropriately activated immune system where macrophages play a key role in its pathogenesis by cytokine production and interaction with other immune cells and fibroblasts. Recently, it was advanced that macrophages can acquire memory-like characteristics following antigen exposure. The subsequent response can be either a heightened inflammatory response or a state of immune tolerance with immunosuppressive effects.

We propose to target trained immunity to modulate the development of chronic fibro-inflammatory pathology in order to demonstrate the involvement of trained immunity in its pathogenesis using a murine model that mimics the systemic sclerosis (SSc) opening the way to design novel therapeutic strategies.

**Material and Methods:** Balb/c Mice were challenged intraperitoneally by two doses of BCG (hyperinflammatory effects) or several low doses of LPS (immunetolerance) and SSc was induced by intradermal HOCl injections. Control groups of untrained SSc mice and untrained PBs mice were considered. Splenic and dermal macrophages were characterized by flow-cytometry analysis, cytokines secretion, epigenetic and metabolic modifications. Co-cultures of trained macrophages and skin fibroblasts from PBS and HOCl mice but also from human SSc patients were performed and fibrosis markers and cytokine production were evaluated. Trained macrophages in vitro were injected to SSc mice and therapeutic effects were evaluated.

**Results:** BCG-mice displayed a worsened clinical (thickened skinfold) and biological SSc features compared with untrained mice while LPSlow-mice had milder SSc manifestations as reflected by skin and lung collagen content, TGF-b, Collagen I, IL-6, alpha-SMA skin and lung expression, expression and anti-Scl70 antibody production. Splenic and dermal macrophages displayed elevated chemokine and inflammatory markers in BCG-mice in opposition to LPSlow-mice. An increase of inflammatory cytokine production in BCG-mice (IL-6, IL-1b, TNF-a) as well as Th2 cytokines (IL-4 and IL-13) contrasted with a shift toward IL-10 secretion in LPSlow-mice. In vitro murine but also human BCG-macrophages upregulated fibrosis markers in diseased fibroblasts and induced a pro-inflammatory cytokine profile in contrast to the effects of LPSow macrophages. Intraperitoneal injection of in vitro LPSlow-trained macrophages ameliorated clinical and biological features of SSc.

**Conclusions:** Our work provides new insight in the physiopathology of systemic sclerosis as inappropriate macrophage stimulation can maintain an aberrant inflammation status leading to an aggravated fibrosis. We also highlight the relevance of targeting trained immunity in human SSc and other auto-immune and inflammation-induced fibrotic disorders where efficient treatments are lacking.

## P.371

## 2-CARBA-CPA AMELIORATES EXPERIMENTAL DERMAL FIBROSIS BY SUPPRESSING THE ACTIVATION OF FIBROBLASTS AND IMMUNE CELLS

T. Higuchi^1^, K. Takagi^1^, A. Tochimoto^1^, T. Norose^1^, I. Masuda^1^, T. Sawada^2^, N. Shibata^2^, T. Morohoshi^3^, M. Harigai^1^, Y. Kawaguchi^1^

^1^*Department of Rheumatology, School of Medicine, Tokyo Women’s Medical University, Tokyo, Japan*, ^2^*Department of Pathology, Tokyo Women’s Medical University, Tokyo, Japan*, ^3^*SANSHO Co., Ltd, Tokyo, Japan*

**Introduction:** Systemic sclerosis (SSc) is a chronic connective tissue disorder that affects the skin and internal organs. The progressive fibrosis severely impairs prognosis and the quality of life of patients with SSc. Inflammation in the affected skin is driven by autoimmunity and activated skin fibroblasts, which have been the traditional relevant therapeutic targets. However, there is no established treatment for SSc. Cyclic phosphatidic acid (cPA) is a naturally occurring phosphatidic mediator. One of its pleiotropic properties is the inhibition of the autotaxin-lysophosphatidic acid (LPA) axis that has been implicated in the pathogenesis of fibrosis in SSc. The purpose of our present study was to evaluate the effects of 2-carba-cPA (2ccPA) against the fibrotic and inflammatory aspects of SSc using human skin fibroblasts and bleomycin-induced skin fibrosis in mice.

**Material and Methods:** Chemically stable 2ccPA was provided from SANSHO Co., Ltd. SSc dermal fibroblasts were obtained from patients with diffuse cutaneous SSc. Adult healthy dermal fibroblasts were commercially obtained. Dermal fibroblasts were incubated with 1-10 uM 2ccPA in the presence or absence of either LPA or TGF-b1. Expression of mRNA was quantified using real-time RT-PCR. Protein expression in cell lysates was analyzed by Western blotting. A mouse model of bleomycin-induced skin fibrosis was established by administering a subcutaneous injection of bleomycin on the back of BALB/c mice 5 times a week for 4 weeks. Littermate control mice were injected with PBS. Mice were peritoneally administered either PBS or 2ccPA (1 mg/kg or 10 mg/kg) 5 times a week for 4 weeks. After the treatment period, skin samples were obtained for histological analyses and type I collagen quantification.

**Results:** 2ccPA significantly suppressed the mRNA expression of fibrotic markers in SSc dermal fibroblasts. However, 2ccPA did not downregulate LPA-driven fibrotic markers in healthy dermal fibroblasts. 2ccPA also decreased the protein expression of type I collagen, CCN2 and aSMA in SSc dermal fibroblasts. In in vivo experiments using bleomycin-induced skin fibrosis mice model, 2ccPA ameliorated dermal thickness and hydroxyproline content. Immunohistological analyses revealed that 2ccPA treatment decreased the numbers of infiltrated F4/80+ cells (control, 7.1 cells/HPF; 2ccPA, 4.3 cells/HPF, p<0.05) in bleomycin-induced skin fibrosis.

**Conclusions:** Our present study suggests that 2ccPA can improve fibrotic properties of SSc by suppressing the activation of both fibroblasts and immune cells.

**Figure:**
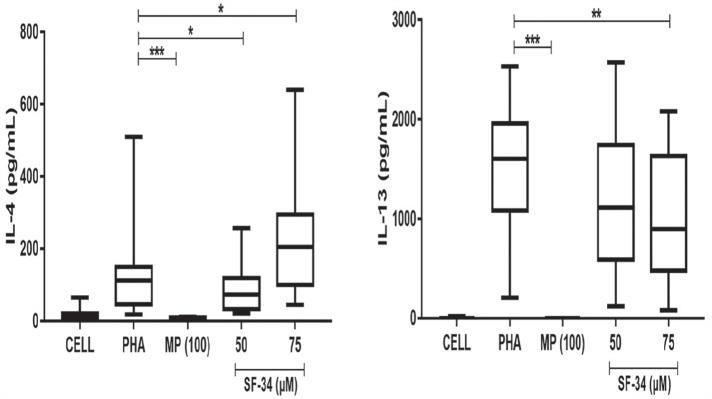


## P.372

## INHIBITION OF IL-4 AND IL-13 PRODUCTION BY A NEW THIAZOLIDINE DERIVATIVE (SF-34) IN PBMC FROM SYSTEMIC SCLEROSIS PATIENTS

M. Galdino Da Rocha Pitta^1^, A. Rodrigues De Almeida^1^, A. Tavares Dantas^2^, E. Gustavo Constantino Cunha^1^, M.A. Bezerra Correia^1^, L.D. De Azevedo Valadares^2^, M.E. De Oliveira Gonçalves^1^, J.V. De Melo Gomes^1^, M.J. Barreto De Melo Rêgo^1^, I. Da Rocha Pitta^3^, M. Galdino Da Rocha Pitta^3^, A. Luzia Branco Pinto Duarte^2^, D. Saes Parra Abdalla^4^

^1^*Laboratório de Imunomodulação e Novas Abordagens Terapêuticas, Núcleo de Pesquisa em Inovação Terapêutica Suely Galdino, Recife - PE, Brazil*, ^2^*Hospital das Clínicas de Pernambuco, Federal University of Pernambuco, Recife-PE, Brazil*, ^3^*Laboratório de Planejamento e Síntese de Fármacos, Núcleo de Pesquisa em Inovação Terapêutica Suely Galdino, Recife-PE, Brazil*, ^4^*Departamento de Análises Clínicas e Toxicológicas, Faculdade de Ciências Farmacêuticas, São Paulo-SP, Brazil*

**Introduction:** Systemic sclerosis (SSc) is an autoimmune disease characterized essentially by vascular changes, immune dysregulation and fibrosis. The pathophysiology of SSc is complex and incompletely understood, and T helper (Th) cells play important roles in this process. Th2 cytokines Interleukin (IL)-4 and IL-13 are implicated as key mediators of fibroproliferative disorders, including SSc. Thus, these cytokines may represent important therapeutic targets. The aim of the present study was to evaluate the immunomodulatory action of a thiazolidine derivative (SF-34) in the production of IL-4 and IL-13 in peripheral blood mononuclear cells (PBMC) of SSc patients.

**Material and Methods:** PBMC from twenty patients were purified and stimulated with PHA (1%) and treated with SF-34 at 50 and 75µM for 48 hours. Methylprednisolone (100µM) was used as a positive control. Cytokines levels were quantified by enzyme-linked immunosorbent assay (ELISA) or cytometric bead array (CBA) in culture supernatants of PBMC from SSc patients. Wilcoxon signed rank test was used to compare the results and p < 0.05 was considered statistically significant.

**Results:** In PBMC from SSc patients, IL-4 levels were significantly reduced after treatment with SF-34 at a concentration of 50µM (PHA = 131,5 pg/mL, PHA + SF-34 50µM= 93,0 pg/mL [p = 0,049]). However, IL-4 levels were increased after treatment with SF-34 at 75µM (PHA = 131,5 pg/mL, PHA + SF-34 75µM = 237,7 pg/mL [p= 0,044]). IL-13 levels were significantly reduced after treatment with SF-34 at a concentration of 75 µM (PHA = 1.515 pg/mL, PHA + SF-34 75µM = 1.070 pg/mL [p= 0,006]).

**Conclusions:** The thiazolidine derivative (SF-34) showed increased and decreased IL-4 production in PBMC supernatant from SSc patients, concentration-dependent. In addition, SF-34 significantly reduced IL-13 levels.

## P.373

## CXCL5 AMELIORATES SYSTEMIC SCLEROSIS VIA SUPPRESSING HELPER T CELL-MEDIATED IMMUNE RESPONSE

X. Fan^1^, D.Y. Guo^1^, C.T. Ng^1^, A. Law^1^, K.H. Lim^1^, J. Thumboo^1^, W. Hwang^2^, A. Low^1^

^1^*Singapore General Hospital, Singapore, Singapore*, ^2^*National Cancer Centre, Singapore, Singapore*

**Introduction:** Systemic sclerosis (SSc) is a multi-systemic disease characterized by immune dysregulation, vasculopathy and fibrosis. Discovery of novel effective therapies with less toxicity is an urgent need. We aim to elucidate the therapeutic potential and working mechanism of CXCL5 in experimental SSc.

**Material and Methods:** Skin fibrosis was induced in 6 to 8-week-old DBA/2J mice by subcutaneous injection of bleomycin (0.5 mg/ml) into a 1 square centimeter area on the lower back every other day for 3 weeks. This was followed by 3 weeks of treatment with either intravenous (i) CXCL5 (twice/week); (ii) dPBS twice/week (control); (iii) bone marrow mesenchymal stem cells [single dose BM-MSCs, 5-10 million cells/kg) or (iv) cyclophosphamide (single dose CP, 1g/square meter body surface area) (comparator arms). Skin thickness was measured from the bottom layer of epidermis to the top layer of fat on H&E staining. The number of infiltrated leukocytes was counted on five randomly-taken photographs with High Power Field (HPF, 400-fold magnification). Circulating immune cell subsets and cytokines were measured by flow cytometry and Luminex technology.

**Results:** Skin thickness was significantly reduced from 346.1 ± 37.9 micrometer (dPBS) to 253.5 ± 51.0 micrometer (CXCL5), which was superior to BM-MSCs (330.2 ± 84.2 micrometer) and CP (309.0 ± 95.3 micrometer) treatment. The leukocyte infiltration was significantly reduced from 6.5 ± 1.9 per HPF (dPBS) to 2.3 ± 2.0 per HPF (CXCL5), which was equivalent to BM-MSCs (2.9 ± 1.3 per HPF) and CP (3.5 ± 1.5 per HPF) treatment. In addition, after shaving, fur recovery was faster in CXCL5 treated mice than mice treated with BM-MSCs and CP. Further experiments showed that CXCL5 reduced the proliferation of TH1 and TH2 cells in in vitro functional assay. When administrated in vivo, CXCL5 reduced the proliferation of monocytes. Multiple proinflammatory cytokines (MCP-1/CCL2, MCP-3/CCL7, MIP-1β/CCL4 and RANTES/CCL5) were significantly reduced. Compared to CP, there were fewer adverse effects on haematopoiesis in mice treated with CXCL5 and BM-MSCs.

**Conclusions:** CXCL5 ameliorated SSc skin thickness by suppressing helper T cell-mediated immune response. Administering exogenous CXCL5 might be an attractive option to treat patients with SSc.

## P.374

## ADIPOSE-DERIVED MESENCHYMAL STEM CELLS CONDITIONED MEDIUM INHIBITS PRO-FIBROTIC ACTIVITY OF SSC FIBROBLASTS AND INDUCE A PRO-ANGIOGENIC PHENOTYPE

N. Del Papa^1^, M. Lorini^2^, V. Carbonelli^1^, F. Pignataro^1^, A. Minniti^1^, F. Campanaro^1^, C. Verrastro^1^, W. Maglione^1^

^1^*UOC DH Reumatologia, G. Pini Hospital, Milan, Italy*, ^2^*Dipartimento di Scienze Cliniche e di Comunità, Università di Milano, Milan, Italy*

**Introduction:** Adipose tissue (AT) is an attractive source of mesenchymal stromal cells (AD-MSCs), characterized by multipotent differentiation potential and regenerative effects. Recent studies have shown that autologous fat grafting may be effective in the treatment of fibrotic and vascular complications in Systemic Sclerosis (SSc), despite a pro-fibrotic signature. Aim of the study was to characterize the proliferative and secretive profile of AD-MSCs in normoxic and hypoxic conditions, and to evaluate mechanisms by which AD-MSCs exert their observed clinical effects.

**Material and Methods:** AT samples were obtained from 10 SSc patients and 8 healthy donors (HD). AD-MSCs were purified according to their adherence to the plastic and characterized to express specific MSC surface antigens by flow cytometry analysis. Proliferation of AD-MSCs from SSc patients and HD was evaluated in normoxic and hypoxic conditions. Fibroblasts and AD-MSCs derived from SSc patients were co-cultured in direct and indirect culture systems and compared to HD. Fibroblasts proliferation, mRNA expression and protein secretion of VEGF and fibrotic mediators including TGFβ-1, TGFR, CTGF, Collagen type 1(Col1) were analyzed in the same conditions

**Results:** Normoxic and hypoxic culture conditions do not modify the proliferation rate of both normal and SSc AD-MSCs. Hypoxia significantly increased mRNA expression of VEGF by normal and SSc AD-MSCs but had no effect on the mRNA expression of pro-fibrotic mediators, like TGFβ and TGFR. Normal and SSc fibroblast proliferation was significantly reduced in both co-culture systems (p<0.0001) and by treatment with normoxic and hypoxic conditioned medium (CM) (p=0.001 and p=0.003). In the same conditions, mRNA expression and protein secretion of TGFβ-1, CTGF and Col1 were significantly reduced (p=0.005). These results were confirmed when normal and SSc fibroblasts were cultured in the presence of AD-MSCs normoxic and hypoxic CM (p=0.02 and p=0.01). Furthermore, a significant increase in mRNA expression and production of VEGF was observed in SSc fibroblasts cultured in the presence of normoxic and hypoxic CM (p=0.003 and p<0.0001, respectively).

**Conclusions:** Treatment with the medium normoxic and hypoxic preconditioned AD-MSCs has an anti-fibrotic effect through the inhibition of fibroblast proliferation and key mediators of fibrosis. The increased expression of VEGF by SSc fibroblasts in the presence of normoxic and, even more, hypoxic AD-MSCs CM suggests that AD-MSCs can induce a pro-angiogenic phenotype. Altogether these data show that AD-MSCs may exert their anti-fibrotic and pro-angiogenetic effects on SSc fibroblasts by the secretion of paracrine factors, partly explaining the mechanisms underlining beneficial clinical results of AT graft in SSc patients.

## P.375

## PED5 INHIBITOR SILDENAFIL COUNTERACTS THE DNA DAMAGE INDUCED BY OXIDATIVE STRESS IN SCLERODERMA FIBROBLASTS

L. Di Luigi^1^, G. Duranti^2^, A. Antonioni^3^, P. Sgro’^1^, R. Ceci^2^, D. Caporossi^3^, S. Sabatini^2^, F. Del Galdo^4^, A. Lenzi^5^, I. Dimauro^3^, C. Antinozzi^1^

^1^*Unit of Endocrinology, Department of Movement, Human and Health Sciences, University of Rome Foro Italico, Rome, Ital, Rome, Italy*, ^2^*Unit of Biochermistry, Department of Movement, Human and Health Sciences, University of Rome Foro Italico, Rome, Ital, Rome, Italy*, ^3^*Unit of Biology, Department of Movement, Human and Health Sciences, University of Rome Foro Italico, Rome, Italy., Rome, Italy*, ^4^*Division of Rheumatic and Musculoskeletal Diseases, Leeds Institute of Molecular Medicine, University of Leeds, Leeds, Leeds, United Kingdom*, ^5^*Department of Experimental Medicine, Sapienza University of Rome, Italy, Rome, Italy*

**Introduction:** Systemic sclerosis (SSc) is a multi-system connective tissue disorder characterized by increased deposition of extracellular matrix proteins such as collagen and fibronectin. Although pathogenesis of SSc is not fully understood, a large number of studies suggest that free radicals could be the major contributors [Stein CM et al., 1996. DOI:10.1002/art.1780390711]. Indeed, different studies demonstrated as an excessive oxidative stress can contribute to the activation of fibrotic process at level of the skin and visceral organs [Denton CP et al., 2006. DOI:10.1038/ncprheum0115].

Emerging evidence highlights the beneficial effects of the phosphodiesterase type 5 (PDE5) inhibition with sildenafil on different cell lines, protecting from reactive oxygen species (ROS)-induced DNA damage [Bernardes FP et al., 2016. DOI:10.1186/s12944-016-0268-6]. These data make sildenafil a good candidate for therapeutic treatment aimed to reduce oxidative damage to biological macromolecules and thus preserve them from cell death.

The objective of this study was to evaluate the sensitivity of SSc dermal fibroblasts to exogenous oxidative stress evaluating the effect of genomic instability on cell cycle kinetics and determine the cellular effects of sildenafil in ROS-induced DNA damage.

**Material and Methods:** We analysed the cellular response of human fibroblasts isolated by SSc patients (HfSSc) and healthy controls to a pro-oxidant environment. Specifically, we assessed cell cycle dynamics and the expression of specific markers of DNA damage (gammaH2aX) and DNA repair (RAD51) following the exposure to exogenous ROS (1h at 50-100µM H2O2), in the presence or not of sildenafil (1µM).

**Results:** HfSSc showed an increased sensitivity to a pro-oxidant environment as assed by significant increase of nuclear gammaH2aX phosphorylation after 1h of 50µM and 100µM H2O2 (46.4%±17.9 and 82%±9.1 respectively) and proportion of cells blocked in G1 sub-phase (17% vs. 3.2% of healthy fibrobalsts) after 1h of 100µM H2O2.

In HfSSc sildenafil treatment reduced the nuclear levels of gammaH2aX phosphorylation by 50% and increased the number of RAD51 foci by 54% induced by 1h H2O2 100µM. Consistent with these findings, treatment with sildenafil reduced by 89% the proportion of cells blocked in G1 subphase growth arrest (17% vs. 2.4% after sildenafil treatment).

**Conclusions:** To our knowledge, this is the first report demonstrating that sildenafil administration prevents ROS-induced genomic instability and cell cycle arrest in human dermal fibroblasts isolated by SSc. These results expand the scope of PDE5 inhibitors as therapeutic agents in SSc by indicating a protective role in tissue damage induced by excessive ROS in vitro.

## P.376

## CONSTITUTIVE ACTIVATION OF TRANSFORMING GROWTH FACTOR BETA RECEPTOR 1 IN LUNG AND SKIN OF TRANSGENIC MOUSE - A NEW MODEL OF SCLERODERMA

G. Bogatkevich, I. Atanelishvili, T. Akter, A. Bogatkevich, R. Silver

*Medical University of South Carolina - Department of Medicine, Charleston, USA*

**Introduction:** Sustained activation of transforming growth factor β (TGFβ) pathways is linked to the development of many fibrotic diseases including scleroderma (systemic sclerosis, SSc). The aim of this study was to generate a novel transgenic mouse model of scleroderma in which over-activation of TGFβ signaling would lead to chronic fibrosis in lung and skin.

**Material and Methods:** Constitutively active (CA) TGF-β receptor 1 (TβR1CA) mouse on the C57Bl/6 genetic background was obtained from Dr. Laurent Bartholin (Lyon University, France) and crossed with BALB/c-Tg(FSP-Cre) mouse from the Jackson Laboratory to express CA TβR1 in fibroblasts and other cells expressing fibroblast specific protein (FSP). Mouse breeding and genotyping were performed in agreement with the Institutional Animal Care and Use Committee at the Medical University of South Carolina. Mice were euthanized at the age of 5, 6, 7, 8, 10, 12, 16, and 20 weeks; lung and skin tissues, isolated fibroblasts, bronchoalveolar lavage fluid (BALF), and plasma were investigated.

**Results:** Recombination of TβR1CA allele at the genomic DNA level was confirmed in 3-week old pups by genotyping. FSP-Cre-negative littermates were used as controls. The FSP-TβR1CA mice appear phenotypically normal but spontaneously develop chronic fibrosis in the lung and skin beginning at week 8. Importantly, lung fibrosis in the FSP-TβR1CA mice appears first in the subpleural portion of the lung showing diffuse involvement of alveolar walls with thickening and fusion closely resembling scleroderma-associated interstitial lung disease (SSc-ILD). We validated the expression of TβR1CA in lung and skin by immunoblotting with anti-HA antibody confirming activation of TβR1 signaling by over-expression of phospho-Smad2 in lung and skin of FSP-TβR1CA mice as compared to control littermates. Masson’s trichrome staining of skin biopsies from FSP-TβR1CA mice demonstrated accumulation of collagen and progressive changes in dermal architecture such as increased dermal thickness and decreased subcutaneous fat in the FSP-TβR1CA mice. Progressive increase of the total collagen in lung and skin of FSP-TβR1CA mice was confirmed by RT-PCR, immunoblotting, and by Sircol collagen assay.

**Conclusions:** Our FSP-TβR1CA mouse provides a novel chronic model of SSc that accurately mimics characteristic features of skin and lung fibrosis observed in the human disease. The model is easy to perform and reproduce, and should be appropriate to test the efficacy of novel antifibrotic therapeutics or to further delineate pathogenesis of SSc, in particular SSc-ILD.

## 16. Case reports

## P.377

## NEWLY DIAGNOSED SYSTEMIC SCLEROSIS IN AFRICAN PATIENT DURING VISIT IN SWEDEN – ETHICAL DILEMMAS OF MEDICAL CARE AND FOLLOW-UP

E. Thomaidi

*Karolinska University Hospital - Department of Rheumatology, Stockholm, Sweden*

**Introduction:** There are only few epidemiological studies about systemic sclerosis that until now support a higher occurrence in northern countries and lower occurrence in southern countries and Asian populations.

**Material and Methods:** We describe a case of a 26-year old woman from Gambia, who came to the emergency room last Februari with worsening joint pain in the extremities. The patient had arrived in Stockholm just a week before, to visit her mother. She was experiencing diffuse pains during the last year but had never met a doctor about it. Her fingertips were whiter and painful when cold and she felt breathless only after little effort. When arriving in Stockholm she felt immediately worse, probably because of the lower temperature. A consulting rheumatologist could confirm the presence of thickened skin up to forearms and knees and partly in the face and the abdomen. Pitting scars could be recognized in all fingertips. Knee and finger-joint arthritis were evident, as well as elbow contractures. The patient was directly placed in the rheumatology ward of the Karolinska hospital for further investigations. These revealed an scl-70-positive diffuse cutaneous systemic sclerosis with Skin Score 29/51 and associated interstitial lung disease. The patient was started with nifedipine, mycophenolate and low-dose prednisone.

There was then the issue of patient follow-up. She had a three-month visa which made the usual routines hard to apply. The patient’s mother was steadfast in her decision to keep her daughter in Sweden. With the help of a hospital-employed social worker she was granted a money benefit to cover some hospital expenses and an extended residence permit for continued medical care. The disease in the meantime progressed further, as confirmed during visit in August, with deteriorating Skin Score of 30/51 and the appearance of digital tip ulcers. Except for one occasion of iloprost-infusion when hospitalized, repeated sessions could not be arranged because of economic issues.

**Results:** The patient was surely at the right place in the right time when the diagnosis was made. However practical and economical obstacles complicate the application of optimal medical care for this case of serious, progressing systemic sclerosis in a young patient. In the meantime, she remains in a colder country, which could possibly aggravate her prognosis.

**Conclusions:** This case illustrates the need for increased awareness of scleroderma occurrence in other places around the world, not in the least in African countries were no epidemiological studies have been done.

## P.378

## A RARE PRESENTATION OF RENAL IMPAIRMENT IN DIFFUSE SYSTEMIC SCLEROSIS PATIENT

K.K. Solanki, A. Schollum, D. Lamont, D. White

*Waikato Hospital, Hamilton, New Zealand*

**Introduction:** RNA Polymerase is associated with diffuse cutaneous SSc (dcSSc) and with increased risk of Scleroderma renal crisis. However, we need to be vigilant regarding other causes of acute renal impairment in dcSSc.

**Material and Methods:** CASE:

A 57year Caucasian man with dcSSc with RNA Polymerase antibodies, was admitted to hospital with general unwellness, decreasing haemoglobin and rising serum creatinine. His C-reactive protein (CRP) was 24mg/L. His mRSS score was 38 out of 51.

**Results:** He had developed rapid progression of skin thickening, no telengiectasias but had early NFC changes and crepitations in lungs with increasing shortness of breath on exertion. His echocardiogram was fine. HRCT chest confirmed interstitial lung disease. He was commenced on mycophenolate mofetil.

5 months from the onset of SSc he was admitted to ward with rising serum creatinine and drop in haemoglobin from 131 to 91g/L and his blood films showed moderate fragmented red cells (consistent with microangiopathic haemolytic anaemia) and polychromasia.

His serum creatinine rose to 132 µmol/L (eGFR 51). He had mildly raised urine creatinine albumin ratio of 3.9 mg/mmol (normal <2.5). Though his blood pressure was 140/80 it was initially suspected to be a normotensive scleroderma renal crisis (SRC).

Re-examination revealed few discrete small vesicular-papular scattered lesions on limbs and torso. Skin swab was done was positive for varicella zoster virus (VZV) PCR.

Skin biopsy was processed for ZANCK test. The smear stained with H&E exhibited herpetic viral cytopathic effect including multinucleation and ground glass degeneration of nuclear chromatin. Cowdry Type A nuclear inclusions were identified. The picture was consistent with herpetic lesion.

He was treated with Variciclovir and his lesions, haemoglobin, renal function tests, urine albumin creatinine ratio all improved to baseline, and he felt better overall.

**Conclusions:** Chicken pox is caused by human herpes virus type 3 aka the varicella zoster virus. VZV related renal involvement is very rare. Usually it is self-limiting but severe complications like in our patient can occur mimicking SRC.

In a large series of more than 2500 patients only 3 (0.12%) developed clinical nephritis. As far as we know none has been reported in SSc patients presenting like SRC. VZV may present as acute proliferative glomerulonephritis (GN) or rarely as crescentic GN and present as microangiopathic haemolytic anaemia (MAHA).

## P.379

## EXTENSIVE CALCINOSIS CUTIS IN A PATIENT WITH SYSTEMIC SCLEROSIS

T. Simopoulou^1^, M. Vlychou^2^, D. Bogdanos^1^, L. Sakkas^1^

^1^*Department of Rheumatology and Clinical Immunology, University General Hospital of Larissa, Faculty of Medicine, Univer, Larissa, Greece*, ^2^*Department of Radiology, University General Hospital of Larissa, Faculty of Medicine, University of Thessaly, Larissa, Greece*

**Introduction:** Calcinosis, presenting as subcutaneous nodules of variable size, is a relatively common clinical feature in Systemic Sclerosis (SSc). However, extensive calcinosis cutis is an extremely rare and difficult to treat manifestation.

**Material and Methods:** We present a 55year old woman with SSc and extensive calcinosis cutis unresponsive to conventional treatment.

**Results:** In 2014, a Caucasian 49year-old woman, with no medical history, was diagnosed with SSc. She was first admitted to Rheumatology clinic for skin infection after surgical removal of a hard, subcutaneous nodule at the suprapatellar area of her right knee. She reported Raynaud’s for at least 5years and gastroesophageal reflux symptoms. Examination was remarkable for sclerodactyli and reduced grip strength. Antinuclear antibodies were positive at 1:160 titre with speckled pattern, without specificity. Pulmonary function tests and echocardiography were normal. The patient was initially treated with antibiotics, and then with methotrexate and diltiazem. Within few months, a significant worsening of calcinosis was noted. Methotrexate dose was increased, and colchicine and low-dose steroids were added. After 18 months the patient presented with fever, headache, blurred speech, clumsiness and mental deficits and immunosuppressants were stopped. Progressive multifocal encephalopathy (PML) was diagnosed with MRI brain lesion and positive PCR for JC virus in the cerebrospinal fluid. The patient showed gradual improvement of her CNS disease but calcinosis progressed. She currently has multiple calcified masses, affecting her buttocks, knees, soles, right hand, arms and shoulders, causing significant pain, recurrent episodes of local inflammation, ulcerations, and occasional infection, resulting in functional limitations, psychological distress and impaired quality of life. Multiple therapeutic agents, including colchicine, acenocoumarol, bisphosphonates, high dose diltiazem, have been used with only slight improvement.

**Conclusions:** Extensive calcinosis cutis is a rare feature of SSc with significant impact in quality of life. No effective therapy exists, making the management of this condition challenging.

**Figure:**
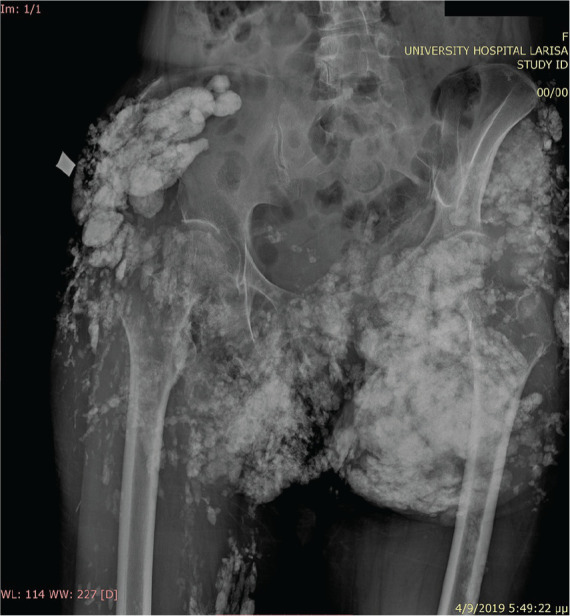


## P.380

## GAVE - AN OFTEN OVERLOOKED COMPLICATION IN SYSTEMIC SCLEROSIS

R. Shukla^1^, A. Herrick^1,2^

^1^*Department of Rheumatology, Salford Royal NHS Foundation Trust, Salford, United Kingdom*, ^2^*Division of Musculoskeletal and Dermatological Sciences, The University of Manchester, Manchester, United Kingdom*

**Introduction:** Gastric antral vascular ectasia (GAVE) is an insufficiently well recognised gastrointestinal feature of systemic sclerosis (SSc). We present two very different cases that demonstrate the need for early diagnosis of GAVE in SSc: local therapy can be successful and can avoid serious complications.

**Material and Methods:** Two cases of GAVE are described (case reports).

**Results:** Patient 1. A 59 year old female with known anti-centromere positive diffuse cutaneous SSc (disease duration of 24 years) attended a routine clinic appointment and reported coffee-ground vomiting that started one day before her appointment. She was known to have a past history of angiodysplasia demonstrated on previous gastroscopy. On examination she was pale and had generalised upper abdominal tenderness with normal bowel sounds. Her modified Rodnan skin score was 14. Haemoglobin was 60g/L (previously 110g/L eleven months ago), mean corpuscular volume (MCV) 83.9fL and urea 15.6mmol/L with normal creatinine (85 micromol/L) suggestive of an acute upper gastrointestinal bleed.

An urgent oesophago-gastro-duodenoscopy (OGD) revealed grade 3 oesophagitis and angioectasia in the duodenum treated with argon beam diathermy with good effect. She received a blood transfusion as an inpatient and her haemoglobin at discharge was 98g/L.

Patient 2. A 55 year old female was referred with Raynaud’s phenomenon, a recent left index finger ulcer and positive anti-centromere antibody. She had sclerodactyly and abnormal nailfold capillaroscopy, and a diagnosis of limited cutaneous SSc was made. She reported receiving 3 units of packed red cells for anaemia four months ago when her haemoglobin had dropped to 60g/L. Her haemoglobin when checked at the clinic was 146g/L and MCV was normal at 88.6fL. Her previous anaemia had not been investigated and an outpatient OGD was arranged.

This revealed classic ‘watermelon stomach’ or GAVE (Fig 1). In addition, it showed grade 1 oesophagitis. She was treated with Omeprazole 20mg daily and has not required any further blood transfusions. She has been taking oral iron supplements since the OGD and her haemoglobin has remained satisfactory.

**Conclusions:** The two case histories emphasise that GAVE should be suspected in patients with SSc presenting either as acute gastrointestinal bleeding (when it is a medical emergency) or chronic anaemia. GAVE remains an important infrequent complication of SSc which is treatable if recognised early.

**Fig 1.**
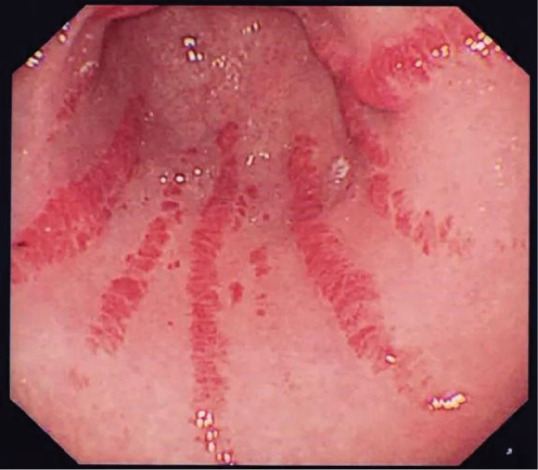
Oesophago-gastro-duodenoscopy examination of patient 2 depicting classic ‘watermelon stomach’.

## P.381

## THE RENAL CRISIS CONUNDRUM

S. Shoela, A. Beltagy

*Alexandria Faculty of Medicine, Alexandria, Egypt*

**Introduction:** Rheumatic diseases are systemic autoimmune diseases that affect joints & soft tissues, but the inflammatory process regularly involves solid organs, including the kidney. To some diseases, like Systemic lupus erythematosus; the kidney is the preferred organ, to Scleroderma, the kidney is a CRISIS.

**Material and Methods:** F is a 32 year old lady previously diagnosed with SLE based on + ANA, arthritis, malar rash & photo-sensitivity. She has 2 children & 1 spontaneous abortion in the first trimester. She also has history of Raynaud’s. She has been on steroids, hydroquine for 2 years, received methotrexate for a short while, & was recently prescribed cyclosporin.Few months ago, she noticed that her skin is getting harder, by examination it showed skin tightness of both hands extending beyond MCP joints but not beyond wrists with limitation of movement at the wrists & fingers, skin over the abdomen was slightly tight & small oral aperture.She presented this time with severe dyspnea, hemoptysis, oliguria, hypertension & was intubated due to severe respiratory distress & hypoxia. On admission she had anemia, thrombocytopenia, impaired renal functions. A CBC film was done revealing normocytic normochromic anemia, thrombocytopenia, red cell fragments, reticulocytes count 10% .24 hour urinary protein 800 mg. Urgent Echo showed dilated LV with reduced systolic function 34%, pulmonary HTN, fair RV systolic function, severe TR.US was done for renal assessment, it was unremarkable &Doppler on renal vessel to exclude any stenosis. CT chest came with bilateral ground glass opacities signifying pulmonary edema VS pulmonary hemorrhage.

**Results:** We are confronted here with a case that was diagnosed with SLE, presenting 2 years later with scleroderma features in hands, acute kidney injury,hypertension,hemolytic anemia with thrombocytopenia (TMA) &proteinuria.

So, we are facing a renal crisis conundrum:

Is it undiagnosed limited scleroderma with scleroderma renal crisis with thrombotic microangiopathy

OR

SLE with lupus nephritis & pneumonitis or pulmonary edema associated with TTP

OR

Cyclosporine induced HTN, renal impairment & TMA

OR

Overlap between systemic lupus & limited scleroderma

we decided that its a case of sclerodermal renal crisis with TMA therefore:She underwent 4 plasmapharesis sessions, extubated and the condition improved.Renal biopsy was done afterwards; it observed mesangiolysis which could be an early feature of a thrombotic microangiopathy.ACE inhibitors were used with calcium channel blockers to control hypertension. MMF was added to control scleroderma afterwards

**Conclusions:** Renal damage in scleroderma remains a challenge for clinicians with a dilemma of differential diagnoses

**Figure:**
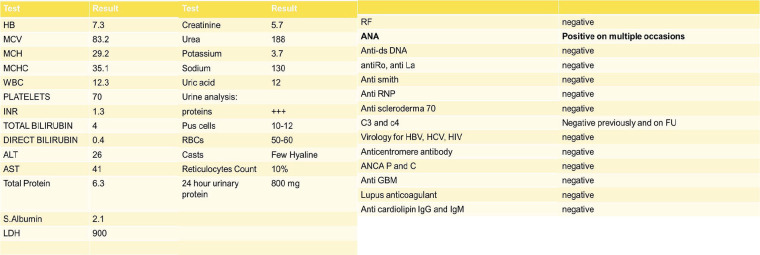


## P.382

## PERICARDIAL INVOLVEMENT AND CREST SYNDROME

O. Selmi, I. Saoud, M. Lajmi, I. Chaabene, R. Amri, H. Tounsi, W. Garbouj, B. Ben Ammou, J. Ouali, R. Jazi

*Mohamed Taher Maamouri, Nabeul, Tunisia*

**Introduction:** CREST syndrome is a limited cutaneous form of systemic sclerosis, an acronym refering to: calcinosis, Raynaud’s phenomenon, esophageal dysmotility, sclerodactyly and telangiectasia.

The prognosis of CREST syndrome is relatively good for a long-lasting disease.

Yet the transition to the systemic form is possible leading to more severe prognosis.

Among the organs affected by scleroderma is the pericardium.

Constrictive pericarditis is a rare complication of CREST syndrome.

**Material and Methods:** Case report of the follow-up of an internal medicine patient from 1996 - 2010.

**Results:** A 58-year-old male with medical history including; a heart rhythm disorder, a hypertension and mesenteric ischemia requiring surgical intervention.

He was diagnosed with CREST syndrome at the age of 45 years old with no need for treatment.

The patient presented acute chest pain and dyspnea and signs of cardiac tamponade requiring pericardiocentesis evacuating a sero-hematic fluid.

A renal insufficiency was noted with a creatinine clearance at 43.7ml / min, and anemia at 8 gr / dl.

The cause of the pericardial tamponade could be secondary to renal failure or related to scleroderma ; a biopsy of the pericardium was performed and concluded that histology is compatible with scleroderma.

**Conclusions:** Our patient has risk factors for poor prognosis such as the age at diagnosis> 30 years, male sex, pericardial involvement and renal failure.

The occurrence of systemic involvment of crest syndrome and its complications can determine its prognosis requiring a more strict follow up.

## P.383

## A CASE OF SYSTEMIC SCLEROSIS & NEUROSARCOIDOSIS

A. Schnell^1^, A. Gaffo^1,2^

^1^*University of Alabama at Birmingham, Birmingham, AL, USA*, ^2^*Birmingham Veterans Affairs Medical Center, Birmingham, AL, USA*

**Introduction:** Systemic sclerosis (SS) is frequently associated with other autoimmune disorders, but its association with sarcoidosis is rare. We report a case of neurosarcoidosis in a patient with SS.

**Material and Methods:** A 49-year-old African American woman with history of SS presented with several months of progressive right-sided weakness with acute worsening in addition to new miosis. Her SS was diagnosed 1.5 years prior to presentation and manifested by Raynauds, sclerodactyly, GERD, pericardial effusion, and an ANA of 1:1280 nucleolar. She complained of shortness of breath and pulmonary function testing showed a TLC of 70 % with DLCO of 58 %. However, high resolution computed tomography (CT) scan was negative for interstitial lung disease and right heart catheterization was negative for pulmonary hypertension. Skin disease was attempted to be treated with hydroxychlorquine (HCQ) and mycophenolate mofetil (MMF), but the patient could not tolerate MMF due to gastrointestinal (GI) side effects. Oral cyclophosphamide (CYC) was attempted, but again patient could not tolerate due to GI side effects. Intravenous (IV) CYC was started. Treatment course was complicated by cytopenias and IV CYC was discontinued after three months of use. She then was started on IV immunoglobulin and was given one dose for worsening skin symptoms three months prior to presentation.

**Results:** To work-up the new right-sided weakness, the patient underwent magnetic resonance imaging (MRI) of the brain and cervical-spine which showed focal area of enhancement near the cervical medullary junction within the right lateral dorsal cord with T2 hyperintensity of the gray matter of the cord to the level of C6. Findings were suspicious for a primary cord neoplasm. Additionally, the patient underwent a CT chest which showed multiple non-calcified right pulmonary nodules most consistent with metastatic disease. Right hilar and mediastinal adenopathy were also concerning for metastatic disease. Mediastinoscopy was performed and biopsy showed non-necrotizing granulomas, consistent with sarcoidosis. The patient was started on methotrexate (MTX) 10 mg/wk. The patient was found to have worsening of the neurosarcoidosis six months after diagnosis and was started on infliximab 3 mg/kg every six weeks and has been maintained for two years without worsening SS or neurosarcoidosis.

**Conclusions:** This case report demonstrates the rare, concomitant occurrence of both SS and neurosarcoidosis with stabilization of both conditions with treatment consisting of MTX, HCQ, and infliximab. This case highlights the importance of awareness of the occurrence of sarcoidosis with SS.

## P.384

## GRANULOMATOUS UVEITIS REVEALING SCLEROMYOSITIS

I. Saoud, W. Garbouj, O. Selmi, R. Amri, H. Tounsi, M. Lajmi, I. Chaabane, Z. Alaya, B. Ben Ammou, J. Ouali, R. Jazi

*Mohamed Tahar Maamouri Hospital, Nabeul, Tunisia*

**Introduction:** The scleromyositis is an overlap syndrome of scleroderma and polymyositis with specific antibodies: anti PM-Scl.This syndrome is rarely revealed by an ocular involvment especially a granulomatous uveitis.

**Material and Methods:** It is a case report about a patient with granulomatous uveitis revealing scleromyositis.

**Results:** A 55-year-old woman without antecedent was referred to our department to explore a bilateral granulomatous anterior uveitis. Systemic sarcoidosis was suspected in front of the ocular granuloma, dry eyes, an interstitial lung syndrome in chest X-ray and a negative intradermoreaction to tuberculin, but this dignosis was rejected in front of a normal level of angiotensin converting enzyme, a normal phosphocalcic balance and normal labial salivary gland biopsy.

At the interrogation, the patient reported myalgia during the four past monthes with Raynaud’s phenomenon. Clinical examination revealed cutaneous sclerosis with limited mouth opening, tapered nose, spontaneous and induced myalgia and proximal muscle weakness.

In blood tests, there were biological inflammatory syndrome and a hight level of muscle enzymes. The electromyogram showed a myopathic involvment. The antinuclear antibodies were present at a rate of 1/640 with speckled appearance. Anti PM-Scl1100 antibodies and anti PM-Scl175 antibodies were hightly positive.

The chest CT scan showed an infiltrative diffuse lung disease and a small pericardic effusion. The diagnosis of scleromyositis was made and the patient was treated with corticosteroid at the dose of 15 mg/day for the moderate muscle involvment. Local treatment with drops was sufficient to get a rapid and clear improvement of the uveitis.

**Conclusions:** The scleromyositis with anti PM-Scl antibodies has usually a good response to corticosteroids.

The severity of scleromyositis remains in scleroderma renal crisis especially with the pericardic effusion as a predictor sign, like for our patient which requires more strict follow-up.

Ocular involvment in scleroderma or myositis rarely occurs in the form of an uveitis and more rarely as agranulomatous one, which make this observation particular especially that is the sign revealing the disease.

## P.385

## SYSTEMIC SCLEROSIS – A POLYSEGMENTAL REFLEX DYSTROPHY – TREATED BY MATRIX RHYTHM THERAPY. CASE REPORT SEVEN YEARS AFTER DIAGNOSIS

U. Randoll^1^, Y. Largiader^2^

^1^*Dr-Randoll-Institut, Munich, Germany*, ^2^*Dental clinic, Grünwald, Germany*

**Introduction:** A lady, born 1970 was suffering in the beginnings of 2003 from Reynod Symptomatic in the area of her fingers on both hands. 2012 she became more and more exhausted gained weight more than 10 kg in a short time so that she consulted specialists at different Clinics who diagnosed in unison Systemic Sclerosis with beginning osteopenia and already affected esophagus. The short and long term prognoses she was given in those days where hard to accept. After standard treatment with regular infusions, she finished after her third cycle of Ilomedin 2013 and switched to 5mg of Cialis daily until end of 2014. Symptoms did not change sustainable and so she was still searching on for other solutions. It was the year 2014 where for the first time she got a whole body Matrix Rhythm Therapy especially along her sympathetic trunk. The question arose anamnestically if stress and emotional strain were the chief causes of systemic sclerosis in our case.

**Material and Methods:** Matrix Rhythm Therapy is a medical treatment based on the so-called entrainment effect. Vibrations according to the 8 – 12 Hz rhythm of alpha range of the CNS and according to the skeletal musculature are applied. The effects are e.g. optimizing the “logistics” around the cells and reduction of the sympathetic load.

Regularly she gets Matrix Rhythm Therapy once a week and stress management beside physical activity.

**Results:** Since 2015 till today the lady is nearly free of symptoms and free of any medication and works full time in her own clinic as dentist for orthodontics, needing her fingers every day.

**Conclusions:** We hypothesize that according to the structural design of “Segment Anatomy” of the human body, the nerves of every organ interact with the “Neurotome”. As a result of segmental sympathetic reflex dystrophies – analog to Sudeck Syndrome – silent inflammations of the interstitial tissue appear in the irritated tomes of the segments and cause successively systemic sclerosis in corresponding organs of Sclerotome, Myotome, Viscerotome, Dermatome.

## P.386

## SEVERE AORTIC ROOT DIALATATION IN PSS REVERSING WITH AGGRESSIVE MEDICAL THERAPY

C. Rajapakse Rajapakse

*Hutt Valley DHB, Lower Hutt, New Zealand*

**Introduction:** Severe Aortic Root Dialatation, potentially requiring aggressive surgery for correction Is rarely seen in PSS, least of all as a presenting feature.

We report such a case that reversed on aggressive medical therapy, and so was able to avoid surgical replacement of aortic root, valves and coronary ostea.

**Material and Methods:** A 67 year old male, presented to outpatients with recurrent atrial fibrillation, reversing with digoxin and metoprolol. On account chest pain, a CT scan of chest was done to exclude an aortic dissection.

Instead, this revealed a very dilated aortic root 55mm, moderate aortic stenosis and severe aortic regurgitation. along with bilateral pleural and pericardial effusions,pulmonary oedema. However he had a normal coronary angiogram.

Following a Rheumatology referral for diffuse MSK and shoulder pains & night sweats he was found to have features of PSS with raynauds ++, diffuse puffy hands and fore arms, reduced fist making, mild knee effusions, bi basal crepes ESR 106,CRP 102, ANA 1/1280, ScL 70 +ve..

Pending Rheumatology evaluation he was started on Prednisone 20mg that had already improved MSK symptoms significantly.

During the ensuing months his treatment was gradually escalated to include Silldenefil 100mg BD, IV Cyclophosphamide monthly for eventually 12 mos. followed by MMF 2 BD, a 5day course of Iloprost infusions which probably led to a rash, and oral pilocarpine for dry mouth.

Prednisone was gradually reduced to 10mg/d

**Results:** The aortic root dilatation on CT from 55mm on Aug ‘14, gradually improved to 52 by July ‘16, complemented by similar improvement on ECHO assessments of aortic root dilatation . AR improved from severe to trivial by Nov ‘15.

Consequently, he was taken off the surgical list, and committed to medical management of cardiac disease and PSS. The pleural and pericardial fluid improved too.So did his PFT’s.

These results and images will be shown in detail.

By June 2016 he was back to leading his previous active life

**Conclusions:** Severe aortic root dilatation with secondary systemic consequences can be a presenting feature of PSS Early recognition and aggressive medical treatment can reverse sequelae of PSS.

## P.387

## SYSTEMIC SCLEROSIS INDUCED BY CNS STIMULANTS FOR ADHD: A CASE SERIES AND REVIEW OF THE LITERATURE

K. Meridor^1^, Y. Levy^1,2^

^1^*Department of Internal Medicine, Meir Medical Center, Kfar Saba, Israel*, ^2^*Sackler Faculty of Medicine, Tel-Aviv university, Tel Aviv, Israel*

**Introduction:** Methylphenidate (Ritalin) is a CNS stimulant, and is a common treatment for children and adults with ADHD. It has been associated with Raynaud’s phenomenon (RP) but not with Systemic Sclerosis (SSc). We report a case series of patients pointing out the connection between Methylphenidate and SSc.

**Material and Methods:** We identified three patients in a single Rheumatology clinic in Israel, who developed SSc following treatment with CNS stimulants for ADHD.

**Results:** All three cases had Raynaud’s phenomenon, skin changes, pathological capillaroscopy and positive ANA. Symptoms appeared and worsened over months following the use of methylphenidate and subsided after its cessation.

**Conclusions:** This is the first report in the literature of a causative relation between methylphenidate and the development of SSc, a serious, life-threatening condition. Patients treated with CNS stimulants should be followed closely for side-effects such as RP and skin changes.

## P.388

## AN UNUSUAL EOSINOPHILIC FASCIITIS OCCURRING IN A FITNESS TRAINER: COINCIDENCE OR RELATIONSHIP?

E. Meleddu, F.L. Renzullo, P. Mascia, A. Cauli, A. Vacca

*Policlinico Universitario Duilio Casula, Cagliari, Italy*

**Introduction:** Eosinophilic Fasciitis (EF) is a rare connective-tissue disease, with characteristic cutaneous sclerosis, peripheral eosinophilia, hypergammaglobulinemia, and elevated erythrocite sedimentation rate (ERS). Onset is between the ages of 40 and 50 years, with a slightly higher prevalence in female sex. Etiology and pathophysiology are unknown. Many factors have been considered as triggers, such as infections, hemodialysis, radiotherapy, hematologic disorders, autoimmune diseases, drugs and even intense physical activity.

**Material and Methods:** A 47 year-old female fitness trainer, with no medical history, was referred to orthopedics in July 2018, because of nocturnal paresthesias of the hands, diffuse polyarthralgias, and myalgia of upper limbs. She was treated with corticosteroids, with partial response.

After two months, she presented to our Department, with oedema and skin tightening of the forearms, legs, and hands, with reduced joint range of motion. She also complained of fatigue and episodic gastroesophageal reflux, but not of Raynaud’s phenomenon nor digital ulcers.

**Results:** Laboratory findings showed slightly elevation of ESR and CRP, eosinophilia (1400/mm3) negative ANA, anti-Scl-70 antibodies and anti-centromere.

There was no sign of organ involvement, and capillaroscopy was normal. Photoplethysmography of the hands after cold pressor test demonstrated vasospasm, although not complained by the patient.

The forearm full skin to muscle biopsy demonstrated fibrotic thickness of the deep fascia,infiltrated with lymphocytes, plasmacells, histiocytes, and eosinophilis.

Diagnosis of EF was confirmed. The patient began treatment with methylprednisolone 16 mg every other day, and methotrexate 15 mg per week.

After three months, the patient reported improvement of symptoms and signs, including skin thickening and functional impairment, and she gradually resumed her career as a fitness trainer.

**Conclusions:** This case emphasizes the importance of strenuous exercise as a possible trigger associated with EF, since our patient’s career as a fitness trainer.

Differential diagnosis for EF includes systemic sclerosis and localized scleroderma, cancer, infections, toxics. In EF, skin tightening of distal digits is characteristically absent, as well as organ involvement.

The possibility of a complete or partial remission of the disease will be evaluated in the follow-up, especially after a complete return to physical activity.

## P.389

## SYSTEMIC SCLEROSIS AND NEPHROTIC SYNDROME: AN UNUSUAL ASSOCIATION

P. Martins^1^, E. Dourado^1^, F. Marques^3^, S. Jorge^3^, Ja. Lopes^3^, J. Guerra^3^, Je. Fonseca^1,2^, I. Cordeiro^1,2^, C. Resende^1^

^1^*Serviço de Reumatologia e Doenças Ósseas Metabólicas, Hospital de Santa Maria, CHULN, Lisboa, Portugal*, ^2^*Unidade de Investigação em Reumatologia, Instituto de Medicina Molecular, Faculdade de Medicina, Universidade de Lisboa, Lisboa, Portugal*, ^3^*Serviço de Nefrologia, Hospital de Santa Maria, Centro Hospitalar Universitário Lisboa Norte, CHULN, Centro Hospitalar U, Lisboa, Portugal*

**Introduction:** Renal complications are common in Systemic Sclerosis (SSc). The most common is scleroderma renal crisis (SRC), but several patterns are recognized. The appearance of nephrotic syndrome has been rarely described in SSc. We report a uncommon case of nephrotic syndrome in a patient with SSc.

**Material and Methods:** 39-year-old man diagnosed with limited cutaneous SSc at the age of 21, presenting with Raynaud’s phenomenon, digital ulcers, joint and limited distal skin involvement, under treatment with naftidrofuryl and aminaftone. He was admitted to the emergency department with a two-weeks history of morning, bilateral and malleolar edema with progression up to the knees. One week before admission, he noted the appearance of foamy urine. He denied any history of exposure to toxins. Upon hospitalization, the physical examination showed blood pressure of 140/50 mmHg, bilateral edema up to the knees, and digital ulcers in both hands. He had normal complete blood count; positive ANA >1/640 homogeneous pattern with negative anti-DS-DNA and ANCA, positive Scl-70 in a high titter (4.390 UQ), elevated ESR (68mm/h), total protein of 5.2g/dL (6.6-8.7), pCreat. of 1.03mg/dl; total cholesterol of 363mg/dL and LDL cholesterol of 274mg/dL. The 24-hour urine analysis showed: volume, 2850 mL; proteins, 23g/L.

**Results:** The kidney ultrasound showed a discrete hyperechoic parenchyma. On light microscopy, the renal biopsy showed a renal cortex with 12 glomeruli, with a slight increase in cellularity and mesangial matrix, without changes on the basement membranes of the glomerular capillaries. A small artery, with preserved morphology, was obtained. Immunofluorescence showed 14 glomeruli with mesangial deposits of IgA (+++) and IgM (+). Electron Microscopy showed podocytopathy. These histologic findings were compatible with IgA nephropaty, with an uncommon clinical presentation characterized by nephrotic syndrome, suggesting a superimposed podocytopathy, with characteristics of minimal change disease (MCD). The patient was treated with prednisolone 0.5mg/kg/day for 10 weeks and mycophenolate mofetil 500mg, twice a day and proteinuria decreased. He tapered prednisolone 5mg/week during 6 months and he is currently on complete remission (proteinuria 78.8 mg/24h), without edema and controlled blood pressure.

**Conclusions:** Few cases of nephrotic syndrome associated with SSc were described in the literature and most of them were associated with D-penicillamine. Our case represents an exceptional form of nephropathy in a SSc patient, unrelated with the drugs usually associated with nephrotic syndrome. The prognosis of IgA nephropathy usually follows a benign course and end-stage renal disease develops only in 20% of patients at 20 years of follow up.

## P.390

## A CASE REPORT OF SYSTEMIC SCLERODERMA WITH POSITIVE ANTI-KU AND ANTI-CYCLIC CITRULLINATED PEPTIDE ANTIBODIES

M. Lajmi, I. Saoud, O. Selmi, H. Tounsi, I. Chaabane, W. Garbouj, Z. Alaya, B. Ben Ammou, R. Jazi, O. Jihen, R. Amri

*Mohamed Taher El Maamouri Hospital, Nabeul, Tunisia*

**Introduction:** Systemic sclerosis (SSc) is a complex autoimmune connective tissue disorder characterized by increased vascular and fibrotic changes in skin and internal organs.

Common SSc-specific auto anti-bodies, such as anticentromere, antitopoisomerase I, and anti-RNA polymerase III antibodies, have been associated with specific clinical features. In recent years, less common SSc-associated autoantibodies such the anti-Ku and anti cyclic citrullinated peptide (anti-CCP) was increased and are more commonly seen in overlap syndromes.

**Material and Methods:** We report a case of systemic Scleroderma with positive anti-ku and anti-cyclic citrullinated peptide antibodies. We will try to show their clinical correlates characterized in our observation.

**Results:** A 39-year-old female, with a past medical history for hypertension consulted for persistent Raynaud’s phenomenon since five years.

She also complained of polyarthralgia involving shoulders, wrists, hands, finger sand ankles without joint swelling. Symptomatic acid reflux disease was present. The patient developed, in recent months, pain and weakness of her arms.

Physical examination revealed sclerodactyly, sclerosis of the face, limitation of mouth opening. No synovitis or tendon friction rubs were noted. Nailfold capillaroscopy finded microvascular abnormalities. Serum muscle enzyme levels were normal. Digestive endoscopy and biopsy showed chronic follicular gastritis. Chest computed tomography revealed Interstitial Lung Disease (ILD) predominantly in the bilateral lower lobes of the lung without restrictive impairment to the pulmonary function test.. Additional laboratory findings included an antinuclear antibody titer of 1/1600 dilutions with a speckled pattern. Further exploration by dot sclerosis revealed the high and single positivity of anti-Ku. The electromyogram was normal. In addition, the anti-CCP was positive contrasting with the negativity of the rheumatoid factor. X-rays as well as the ultrasound of the small joints of the hands were without abnormalities. The patient was diagnosed with Systemic scleroderma and was treated with calcium-channel blockers and proton-pump inhibitors.

**Conclusions:** Autoimmunity is one major factor in SSc pathogenesis. Anti-Ku and anti-CCP antibodies are rare in SSc and their presence does not lead to a diagnosis of overlap syndromes, as is the case with our patient. A statistically significant relationship was demonstrated between anti-CCP and erosions in addition to an increased risk pulmonary fibrosis. Other findings reported clinical associations between anti-Ku autoantibodies in SSc and ILD. In our study, the detection of anti-CCP and anti-ku has been correlated with the presence of ILD. These antibodies could be an interesting prognostic factor for pulmonary involvement in the course of SSc.

## P.391

## SYSTEMIC SCLEROSIS ASSOCIATED WITH ANTI-NEUTROPHIL CYTOPLASMIC ANTIBODY POSITIVE

J.H. Jung^1^, S.J. Choi^1,2^, G.G. Song^1,3^

^1^*Korea University College of Medicine, Seoul, South Korea*, ^2^*Korea University Ansan Hospital, Ansan-si, South Korea*, ^3^*Korea University Guro Hospital, Seoul, South Korea*

**Introduction:** Systemic sclerosis is commonly associated with anti-centromere or anti-topoisomerase antibodies. However, systemic sclerosis associated with anti-neutrophil cytoplasmic antibody (ANCA) positivity is often reported, with clinical symptoms similar to ANCA-associated vasculitis (AAV).

**Material and Methods:** We report a case of systemic sclerosis with ANCA positivity that was treated according to AAV.

**Results:** A 61-year woman presented with daily fever of 38°C or more, which started two month ago. When she had a fever, she had no febrile sensation or chilling. There was no symptoms such as cough and sputum, but there was crackle in respiratory sound, and usual interstitial pneumonia was diagnosed on plane radiography and high-resolution computed tomography.

She had Raynaud’s phenomenon for three years. She had puffy fingers, and sclerodactyly was observed on one finger. However, distal ulceration was not found. She was diagnosed as systemic sclerosis with a total of 9 points according to the 2013 American College of Rheumatology / European League Against Rheumatism classification criteria. She was prescribed nifedipine with empirical antibiotics.

However, the cause of the fever was not clear and persisted, and laboratory tests showed elevated erythrocyte sediment rate (ESR; >120 mm/hr) and C-reactive protein (CRP; 110 mg/L). Fluorescent antinuclear antibody results were positive (1:1,280, speckled type) and titers of anti-RNP and anti-Sm antibodies were elevated, but anti-centromere was negative and anti-topoisomerase antibodies was weakly positive. RF was elevated, and ANCA titer was also elevated to 1:2560. Both myeloperoxidase and proteinase-3 ANCA were highly measured.

After three days, she developed painful palpable purpura in her toes, fever did not improve, and ESR and CRP gradually elevated. Although symptoms such as renal involvement are not clearly correspond to AAV, we suggested that AAV was possible based on fever, interstitial lung disease, which is not common in AAV, and ANCA positivity. Prednisolone and cyclophosphamide were administered according to AAV with pulmonary fibrosis. After a month, body temperature returned to normal, skin symptoms improved, and ESR and CRP returned to normal levels.

**Conclusions:** The presence of ANCA in systemic sclerosis may be a clue in determining treatment options when accompanied by clinical symptoms related with AAV.

## P.392

## SEVERE LIMB ISCHEMIA AFTER VASOPRESSOR THERAPY DUE TO SEPTIC SHOCK FROM PERFORATED ACALCULOUS CHOLECYSTITIS IN A PATIENT WITH DIFFUSE CUTANEOUS SYSTEMIC SCLEROSIS

J.-B. Jun^1^, J. Kang^1^, Y.H. Kim^2^

^1^*Hanyang University Hospital for Rheumatic Diseases, Seoul, South Korea*, ^2^*Hanyang University Seoul Hospital, Seoul, South Korea*

**Introduction:** We report a rare case of amputation in both hands and feet due to severe gangrene, which was resulted from vasopressor therapy in severe vasodilatory shock, in a patient with diffuse cutaneous systemic sclerosis (dcSSc).

**Material and Methods:** She was a 71-year-old woman who was diagnosed with dcSSc and interstitial lung disease (ILD) in April 2013 and received a year of intravenous cyclophosphamide treatment. Since then, she has been treated with methotrexate and mycophenolate mofetil 2g/day for rapidly progressive cutaneous disease and ILD. In November 2018, she visited the emergency room due to high fever and was admitted under acute cholangitis suspicion. After about a week with antibiotic therapy, sudden high fever, right upper quadrant tenderness, and blood pressure decline occurred. Vasopressor therapy was introduced promptly in emergency condition. Moreover, intubation and continuous renal replacement therapy (CRRT) were performed due to respiratory failure and metabolic acidosis. Percutaneous transhepatic gallbladder drainage was inserted with suspicion of acute acalculous cholecystitis due to severe edematous wall thickening with increased vascularity of gallbladder on abdomen ultrasonography.

**Results:** Vasopressor therapy decreased as blood pressure stabilized, but severe ischemic changes occurred in both hands and feet. The vital sign became stable and the vasopressor therapy and CRRT were discontinued and extubated. However, gangrene occurred in both hands and feet with ischemic changes. A laparoscopic cholecystectomy was performed in December 2018. In computed tomographic angiography of the upper and lower extremities, small blood vessels of the hands and feet were damaged and could not be evaluated. In January 2019, amputation and flap operations were performed on both hands and feet.

**Conclusions:** In patients with poor peripheral circulation, such as patients with systemic sclerosis, no matter how critical an emergency is, vasopressor therapy requires more careful judgement and management.

## P.393

## SYSTEMIC SCLEROSIS SKIN CHANGES AND MULTI-ORGAN INVOLVEMENT AFTER TAXANE TREATMENT

N. Bakhsh^1,2^, K. Devakandan^1,2^, Z. Ahmad^1,3^

^1^*Toronto Scleroderma Program, Toronto, Canada*, ^2^*Mount Sinai Hospital, Toronto Western Hospital, Department of Medicine, Division of Rheumatology, Toronto, Canada*, ^3^*Mount Sinai Hospital, Department of Medicine, Division of Rheumatology, Toronto, Canada*

**Introduction:** There are several case reports which illustrate the well-established link between taxanes and scleroderma-like skin changes. Skin involvement can include both limited and diffuse cutaneous systemic sclerosis subsets, and can occur after the first dose or several years later. Few reports demonstrate an association with internal organ involvement, and the literature is further limited in multi-organ involvement.

**Material and Methods:** A 42-year-old female presented to Toronto Scleroderma Program following a recent diagnosis of systemic sclerosis manifesting as Raynaud’s phenomenon, sclerodactyly, inflammatory arthritis, and a Modified Rodnan Skin Score (MRSS) of 13. Her manifestations started after she was treated for uterine adenocarcinoma. She was treated with radical hysterectomy and bilateral salpingo-oophorectomy in October 2016, followed by chemotherapy which included taxanes and radiotherapy. 16 months after treatment, she developed inflammatory myositis, myocarditis, suspected renal crisis and severe gastrointestinal dysmotility with CT scan findings of interstitial lung disease and pneumatosis intestinalis. She was treated with captopril, prednisone, myfortic, digoxin, and metoprolol. Blood work revealed a positive ANA and anti-Ro-52 antibodies. She was negative for anti-Scl-70, anti-centromere, anti-RNA Pol III, anti-Smith, anti-Jo and anti-RNP antibodies.

**Results:** We believe this is the first report of scleroderma-like skin lesions and multiple internal organ involvement after taxane treatment for uterine adenocarcinoma. There are no reports that show organ involvement in taxane-associated scleroderma on this scale. Improvement of her inflammatory myositis, myocarditis and renal status was seen with immunosuppression and an ACE inhibitor. Although several reports have shown improvement after taxane withdrawal, there still may be a need for systemic steroid use with or without further immunosuppression, as is evident by this case.

**Conclusions:** Although the association between taxanes and scleroderma-like skin changes has been reported, literature is scarce with regard to multi-organ involvement in scleroderma following taxane use. Our understanding of the relationship between taxane exposure and development of scleroderma is also complicated by the known relationship between scleroderma and malignancy. This case suggests that a high burden of multi-organ involvement may occur months after taxane treatment has ended.

## P.394

## SUDDEN CARDIAC DEATH IN TWO PATIENTS WITH RAPIDLY PROGRESSING SYSTEMIC SCLEROSIS

R. Izzo, G. Pellegrino, G. Olivieri, C. Angelelli, K. Stefanantoni, C. Perricone, C. Alessandri, R. Scrivo, F. Conti, R. Priori, G. Valesini, V. Riccieri

*Dipartimento di Medicina Interna e Specialità Mediche, Reumatologia, Sapienza Università di Roma, Roma, Italy*

**Introduction:** Systemic Sclerosis (SSc) is an autoimmune disease, characterized by severe and often progressive organs involvement. Age at disease onset, male sex, diffuse cutaneous SSc (dcSSc), anti-Scl70 antibodies, cardiac, renal and lung features are associated with worse prognosis and high mortality risk1. Heart involvement is underestimated because it frequently occurs asymptomatic. The reported prevalence of cardiac rhythm disease in SSc patients varies from 25% to 75% and may result in sudden cardiac death2,3.We present two similar clinical histories both characterized by a dramatic end.

**Material and Methods:** Case 1. A 67-years-old female with a one-year history of Raynaud’s phenomenon, sclerodactily, digital ulcers, dysphagia and a capillaroscopic scleroderma pattern, with no lung or cardiac involvement, was treated with iloprost and mycophenolate mofetil. She was admitted to our Rheumatology Unit because of cachexia, diarrhea, worsening of swallowing and rapidly progressing dcSSc. The total body computed tomography (TB-CT) showed interstitial lung disease (ILD) and severe hypotonia of the intestinal loops; anti-SSA/Ro autoantibodies were the only immunological markers detected. On the 9th day of hospitalization the patient suddenly reported palpitations, atrial fibrillation was detected and she was promptly treated with b-blockers, but two days later a right bundle branch block appeared soon followed by cardiac arrest.

**Results:** Case 2. A 54-years-old man was diagnosed SSc in our Scleroderma Clinic due to a progressive and diffuse skin hardening, dysphagia and diarrhea, started six months before. A capillaroscopic scleroderma pattern was found, ANA and anti-ENA were absent, including anti-RNA-polymerase III, anti-Scl70 and anti-centromere. TB-CT showed distended gastric and small bowel loops as well as ILD; no cardiac rhythm abnormalities were reported. His symptoms improved with high dose methylprednisolone but two months later he was admitted to the Rheumatology Unit due to acute endocarditis, treated with antibiotics. During the hospitalization SSc rapidly worsened and at the 30th day of hospitalization the patient died due to a sudden cardiac arrest.

**Conclusions:** These two clinical cases show a similar disease course, with aggressive skin, lung and gastrointestinal involvements, without neoplasms and specific autoimmunity markers. In both cases fatal arrhythmias suddenly occurred. Serious cardiac complications happen more often in dcSSc, particularly during the first year after diagnosis2. Besides conduction heart disorders are not fully defined due to the variety of symptoms and the lack of wide and well-defined cohorts of patients, so it appears necessary to report all the available cases to better understand the early clinical signs and promptly treat severe heart involvement in SSc.

## P.395

## RARE PULMONARY MANIFESTATIONS OF SYSTEMIC SCLEROSIS – A UNIQUE CASE OF DIAPHRAGMATIC DYSFUNCTION

M.G. Guerra^1^, M. Silva^2^, S. Neves^2^, B. Samões^1^, D. Fonseca^1^, T. Videira^1^, R. Vieira^1^, J. Abelha-Aleixo^1^, P. Pinto^1^

^1^*Centro Hospitalar de Vila Nova de Gaia/Espinho, Department of Rheumatology, Vila Nova de Gaia, Portugal*, ^2^*Centro Hospitalar de Vila Nova de Gaia/Espinho, Department of Pneumology, Vila Nova de Gaia, Portugal*

**Introduction:** To date, the best recognized pulmonary manifestations in Systemic Sclerosis (SSc) are interstitial lung disease (ILD) and pulmonary hypertension (PH), which contribute greatly to disease morbimortality. Despite this, other impactful entities are described, such as thromboembolic disease, pleural effusion or airway disease. Isolated diaphragmatic dysfunction, however, is extremely rare, with only 2 cases described in literature.

**Material and Methods:** Description of a unique case of SSc-related diaphragmatic dysfunction.

**Results:** A 42-year-old female has been followed at our Rheumatology outpatient clinic for 11 years, with a diagnosis of scleromyositis, since the age of 24 years. During disease course, her clinical manifestations included limited cutaneous skin thickening, myositis, polyarthritis, Raynaud Phenomenon, digital ulcers/finger ischemia, oesophageal dysmotility and anorectal incontinence, treated with prednisolone, methotrexate (MTX), loperamide and iloprost cycles. She had positive anti-nuclear antibodies (titre 1/1280, nucleolar pattern, no specificities detected). At the age of 41 years, the patient presented with new-onset exertional dyspnoea (NYHA class II), with normal cardiopulmonary auscultation. Still suspecting cardiopulmonary involvement, MTX was suspended and further exams performed: high-resolution CT showed no parenchymal changes, transthoracic echocardiogram had no signs of PH or contractile dysfunction, electrocardiogram was normal and lung function tests (LFT) elicited a mild restrictive pattern (FVC 64%). Blood tests showed slightly raised C reactive protein (0.7mg/dL), raised myoglobin (117.1ng/mL) and normal aldolase/creatine kinase. During the following months, dyspnoea worsened progressively, as well as the LFT (deteriorating restrictive pattern with low DLCO) but with no clear cardiopulmonary involvement nor myositis. Furthermore, the chest x-ray depicted a left diaphragmatic elevation, which raised the suspicion of abnormal diaphragm contraction. Respiratory muscle function was assessed, that showed reduced maximal inspiratory pressure (MIP), maximal expiratory pressure (MEP) and sniff tests, respectively, -23 cmH2O, +14cmH2O and -17 mmHg. Supine spirometry was performed and presented a drop in FVC of 18%, compatible with diaphragm dysfunction. Diaphragm ultrasound did not find relevant findings. Based on this results, respiratory kinesiotherapy and treatment with oral azathioprine were started.

**Conclusions:** Diaphragmatic dysfunction is a heterogeneous entity related to a multitude of diseases. In SSc, it is an extremely rare manifestation and needs to be clearly distinguished from the usual causes of SSc-related dyspnoea (ILD and PH), for an adequate treatment, that includes respiratory rehabilitation.

## P.396

## REFRACTORY DIGITAL ULCERS WITH NECROSIS IN PATIENT WITH OVERLAP SYNDROME – EFFECT OF HYPERBARIC OXYGEN TREATMENT

K. Gheorghe^1^, A. Nordin^2^

^1^*Karolinska University Hospital, Rheumatology, Stockholm, Sweden*, ^2^*Karolinska Institute, Department of Medicine, Division of Rheumatology, Stockholm, Sweden*

**Introduction:** Digital ulcers are common manifestation in systemic sclerosis patients and represent a major cause of morbidity. The treatment is difficult and requires a multimodal therapeutic approach.

**Material and Methods:** Here we describe a 31-year patient with overlap syndrome consisting of Clinical and immunological features of systemic sclerosis, myositis and systemic lupus erythematosus who developed digital ulcers non-responding to combination therapy with vasodilator drugs.

**Results:** The disease was diagnosed at age 26 with clinical symptoms consisting of skin rash, Raynaud’s phenomenon, finger ulcerations, sclerodactyly, arthralgia/ arthritis, hair loss, fever, chest pain, difficulty in swallowing, palate ulcerations. Laboratory evaluation showed lymphopenia, anemia with positive direct antiglobulin test, complement activation, increased levels of muscle enzymes. Immunological profile revealed the presence of antinuclear antibodies with specked pattern and autoantibodies against Scl-70, ribosomal- P, SS-A, SS-B, Ku, as well as dsDNA with immunofluorescence technique. Radiological investigation showed pericardial effusion, esophageal dysmotility, muscle edema by MRI and later discrete interstitial parenchymal changes and ground glass. The initial immunosuppressive treatment with methotrexate, hydroxychloroquine and low dose prednisolone was changed over the years to rituximab and later to mycophenolate. For the digital ulcers, she was started on vasodilator therapy with calcium channel blocker nifedipine and iloprost infusions every month with good effect in the beginning and healing of ulcerations. Following a patient-based decision to discontinue the medication, her condition worsened with flares of muscle inflammation and relapsing pericarditis. New ulcers occured on extremities with necrosis and infection of skin and underlying bone. Longtime antibiotics, local wound treatment and addition of sildenafil, bosentan, acetyl salicylic acid and iloprost did not achieve stabilizing of ulcer progression. Injection with Botulinum toxin performed by hand surgeon was without effect. MRI angiography of the arterial tree revealed reduced blood filling of the superficial and deep palmar arcus in both hands. No endovascular nor open surgery was deemed possible by the vascular surgeon.

She was referred for treatment with hyperbaric oxygen as add-on therapy which she underwent for 40 sessions. She tolerated the treatment well and experienced relief of pain during the sessions. The wounds improved slowly and remained stable thereafter under iloprost infusions which she continued without interruption.

**Conclusions:** This case illustrates the difficulty in treating refractory systemic sclerosis related ulcers and the possible rescue therapy that hyperbaric oxygen therapy can achieve in selected cases.

## P.397

## DEPLETION OF B-CELLS IN PERIPHERAL BLOOD FOR 10 YEARS IN A PATIENT WITH SYSTEMIC SCLEROSIS, AFTER A SINGLE INFUSION OF RITUXIMAB

L. Garzanova, L. Ananyeva, O. Koneva

*V. A. Nasonova Research Institute of Rheumatology, Moscow, Russia*

**Introduction:** Currently, quite a lot has been written about the efficiency of rituximab (RTX) for the treatment of systemic sclerosis. However, there are not many long-term observations.

**Material and Methods:** We prospectively followed-up a course of the disease of a woman, 71-years-old, with diffuse SSc. Duration of the disease at the first visit was 2 years, debut with interstitial changes in the lungs, gastrointestinal symptoms, arthritis. SSc was diagnosed in 2008. At physical examination–Rodnan skin score (mRss)-25, dyspnea (NYHA-3), ANA hep-2–1/1280, anti-Scl-70 and ACA–negative, B-cells - 17,4%(0,649*109/l). ECG:sinus rhythm,65bpm. Echo: left ventricular ejection fraction-68%, pulmonary artery systolic pressure–38mmHg. HRCT-widespread bilateral diffuse interstitial changes in the lower parts of the lungs (“ground glass opacification” type). Forced vital capacity (FVC)-78,7% of predicted, diffusing capacity of the lungs for carbon monoxide (DLCO)-49,2% of predicted. 6 minute walk test(6-mwt)-360m. Activity score-4.HAQ–2,125.

**Results:** Rituximab 2000mg was administered in May 2008. There was complete depletion of B-cells after the infusion. Prednisolone 20mg/day, cyclophosphamide (CP) 800mg/month were prescribed at the same time. Total received dose of CP was 5g (stopped due to poor tolerance). The dose of prednisolone was reduced to 10mg/day by 2010. In subsequent years, the patient received prednisolone monotherapy (from 2014-5 mg/day). As a result of the therapy after 12 months - working capacity was improved, dyspnea decreased (NYHA-2), the mRss-5, B-cells – 0,033% (0,001*109/l); ANA hep-2-1/640 nucl, FVC-93,8%; DLCO-46,5%. HRCT-decrease in the degree of intense and size of zones of “ground glass opacification”. Activity score-0,5. 6-mwt-440m. HAQ–1,25. There was depletion of B-cells during 8years of observation and a low level of B-cells during next period. At examination in 2012: B-cells – 0,022%(0,001*109/l); activity score-0,5; mRss-4; HAQ–1,125; ANA hep-2–1/320. In 2014: B-cells – 0,8%(0,021*109/l); activity score-0,5; mRss-2; HAQ–1,125; ANA hep-2–1/320. In 2015: B-cells – 2,4%(0,028*109/l); activity score-0,5; mRss-1; HAQ–1; ANA hep-2–1/320. In 2016: B-cells – 4,4%(0,081*109/l); activity score-0,5; mRss-1; HAQ–0,375; ANA hep-2–1/320. In 2017: B-cells – 16,5%(0,259*109/l); activity score-0,5; mRss-0; HAQ–0,25; ANA hep-2–1/640. At the time of the last observation in August 2019 the patient was stable, the mRss-0, dyspnea (NYHA-2), HAQ–0,25.

**Conclusions:** This observation demonstrates prolonged B-cells depletion after a single infusion of RTX and subsequent treatment with a short course of CP and small doses of prednisolone. Low B-cells level was accompanied with clinical improvement of quality of life and working capacity, decreased disease activity and ANA. Early administration of RTX may be a good option for the initial treatment of diffuse SSc with lung involvement.

## P.398

## SYSTEMIC SCLEROSIS TRIGGERED BY CHIKUNGUNYA FEVER

S.M.D. Fontenele^1^, I. Barbosa^2^, B. Parente^2^, J. Monte^2^, M. Moura^2^

^1^*UECE, Fortaleza, Brazil*, ^2^*UNICHRISTUS, Fortaleza, Brazil*

**Introduction:** Chikungunya Fever is an arbovirusis caused by an arthritogenic Togaviridae virus, transmitted by mosquitoes Aedes, that may persist in target tissues, such as joint, and stimulate the formation of inflammatory mediators, leading to a chronic and uncertain course.

**Material and Methods:** To describe a Case report of a 30-year-old female patient that since on 2017 has presented chikungunya viruses with high fever, arthritis and continuous mild retroorbital headache and to realize a literature review about this theme.

**Results:** Even though she received symptomatic treatment, persistent neuromusculoskeletal involvement compromised her daily activities. Therefore, Methotrexate therapy was prescribed with maximum dose of 20mg/week, obtaining partial improvement of the symptoms. One year post acute fase, she sought for emergency due to edema of hands and feet, painful reduction of joint amplitude in metacarpophalangeal and proximal interphalangeal, which led to the suspicion of systemic lupus erythematosus or rheumatoid arthritis. On investigation with rheumatologist, patient referred photosensitivity, scaly xeroderma and diffuse cutaneous hyperpigmentation, Raynaud phenomenon and loss of skin elasticity. Physical examination revealed skin thickening and adherence to the deep planes of extremities and face, lip-sharpening, mild bilateral exophthalmia, and diffuse thyroid enlargement. Laboratory tests: Anti-Intrinsec Factor Antibody: 0,1; Hemoglobin:13.1 g/dL; Hematocrit:40.3%; MCV:85.7fL; MCH:27.9pg; MCHC:32.5g/dL; RDW:15.2%; Leukocytes:6750; Neutrophils:3726; Eosinophils: 351; Basophils:41; Lymphocytes:2072; Monocytes:560; Platelets: 328.000; PCR:>0.05mg/dL; Protein Electrophoresis: TP: 8,1g/dL; Albumin:52.4%; Alpha1 globulin:3.5%; Alpha2 globulin: 8.7%; Beta1 globulin: 5.4%; Beta2 globulin:3,5%; Gamma globulin:26.5% (polyclonal Hypergammaglobulinemia); VHS:10mm; Creatinin:0.65mg/dL; Urea: 32mg/dL; CPK: 307U/L; LDH:189UI/L; Latex RF:>9, 3UI/mL; Antithyroglobulin Antibody:435UI/mL; Thyroid Antithyreoperidase Antibody:>1300UI/mL; FAN:nuclear fine dotted 1/320; ACA: nonreactive; Anti-RNP:>5.0U/mL; Anti-SSA:>7.0U/mL; Anti-SSB:>7.0U/mL; Anti-Scl70: >7.0UmL; C3 Complement: 113mg/dL; C4 Complement: 16,9mg/dL. It was performed a capillaroscopy, that showed SD pattern, according to Maricq, in the active phase of capillary ectasia and vascular deletion (by Cuttolo). Thus, he was diagnosed as Systemic Sclerosis (SS) and began using azathioprine, 100 mg/day, in combination with levothyroxine for hypothyroidism. After three months, there was already some clinical improvement, even returning to exercise.

**Conclusions:** On chronic ChikF, some specific rheumatologic diseases have been described as triggered or worsened. However, there is no such citated relation about SS. The authors believe that the patient already had an organ-specific immune-mediated pathology and, after ChikF, might have underwent by a systemic immune dysregulation, with typical SS manifestations.

## P.399

## GROUP 3 OR COMBINED GROUP 1 AND GROUP 3 PULMONARY HYPERTENSION? THREE CASES WITH THREE SCENARIOS

S. Erol, Z.P. Onen, O. Ozdemir Kumbasar, T. Sayin, C. Tulunay Kaya

*Ankara University School of Medicine, Ankara, Turkey*

**Introduction:** In patients with systemic sclerosis (SSc), pulmonary hypertension (PH) may occur due to pulmonary arterial hypertension (group 1), left ventricular dysfunction (group 2) or systemic sclerosis-interstitial lung diseases (SSc-ILD) (group 3). In patients with both PH and ILD, it can be difficult to distinguish group 1 PH from group 3 or a combination of PH-ILD and PAH. According to recommendations of the 6th World Symposium, FVC<70% with extensive parenchymal changes on CT favors group 3 PH. However, if severe PH –defined as mPAP >35 mmHg- is present, individualized care is recommended.

**Material and Methods:** We present three cases with extensive parenchymal changes on computed tomography (CT) and high PH.

**Results:** Case 1 is 54 years old female with hypoxemia and extensive fibrosis on CT. Initial FVC and DLCO were 75% and 42% respectively. FVC decreased to 41% and DLCO to 30% despite similar CT findings in one year. Hypoxemia increased and she had a gradual increase in systolic PAP. She underwent right heart catheterization (RHC). Mean pulmonary arterial pressure (PAP), pulmonary vascular resistance (PVR) and pulmonary capillary wedge pressure (PCWP) were 32 mmHg, 6 woods/unit and 6 mmHg respectively.

Case 2 is 63 years old female patient with hypoxemia and extensive fibrosis on CT. Findings were similar between 2017 and 2019. However, sPAP increased to 60 mmHg from 30 mmHg and DLCO decreased to 19% from 26% despite normal FVC. RHC, mean PAP, PVR and PCWP were 40, 10 and 5 respectively.

Case 3 is a 41 years old male patient with hypoxemia and extensive emphysema on CT. His FEV1 and FVC were in normal limits during follow-up but DLCO decreased to 30% from 48%. Mean PAP, PVR and PCWP were 58, 6 and 5 respectively. The patient commenced on PAH specific treatment. Three months later walk distance and hypoxemia improved.

**Conclusions:** All patients have PH, hypoxemia and extensive CT findings. If CT findings are extensive (>20% of lungs involved ) and hypoxemia is present patients generally classified as group 3. However, as mentioned above if they have severe PH individualized care is necessary. As a conclusion, differentiation between group 1 PH and group 3 is not easy. Possibility of group 1 PH or combination of group1 and group 3 should be kept in mind even in patients with extensive parenchymal lung diseases. And as in case 3, they may see benefit from PAH specific treatment.

## P.400

## MORPHEA WITH ASSOCIATED SYSTEMIC MANIFESTATIONS: A CASE REPORT

A. Elsonbaty^1^, N. Adel Abbas^2^, N. Fathi^1^

^1^*Rheumatology and Rehabilitation unit, Assiut university hospital, Assiut, Egypt*, ^2^*Diagnostic Radiology unit, Assiut university hospital, Assiut, Egypt*

**Introduction:** Morphea (or Localized scleroderma) is an idiopathic disorder characterized by local inflammation followed by fibrotic changes in the skin and subcutaneous tissues. Despite a shared histologic appearance between systemic sclerosis and localized disease, the clinical distribution of lesions serves to distinguish these two disorders. Some literature reported systemic manifestations with morphea which resulted in changing concept for the nomenclature – using the term morphea in localized scleroderma and preference of this terminology.

**Material and Methods:** We present a 20 years old male patient with circumscribed markedly thickened skin lesion over neck, chest and part of the abdomen associated with systemic manifestations in the form of serositis, GIT involvement, cardiac affection and peripheral neuropathy.

**Results:** After investigations and after excluding other differential diagnosis. We propose using the term “Morphea with systemic manifestations.

**Conclusions:** We suggest reconsidering the former terminology Localized Scleroderma in new diagnostic and/or classification criteria.

**Figure:**
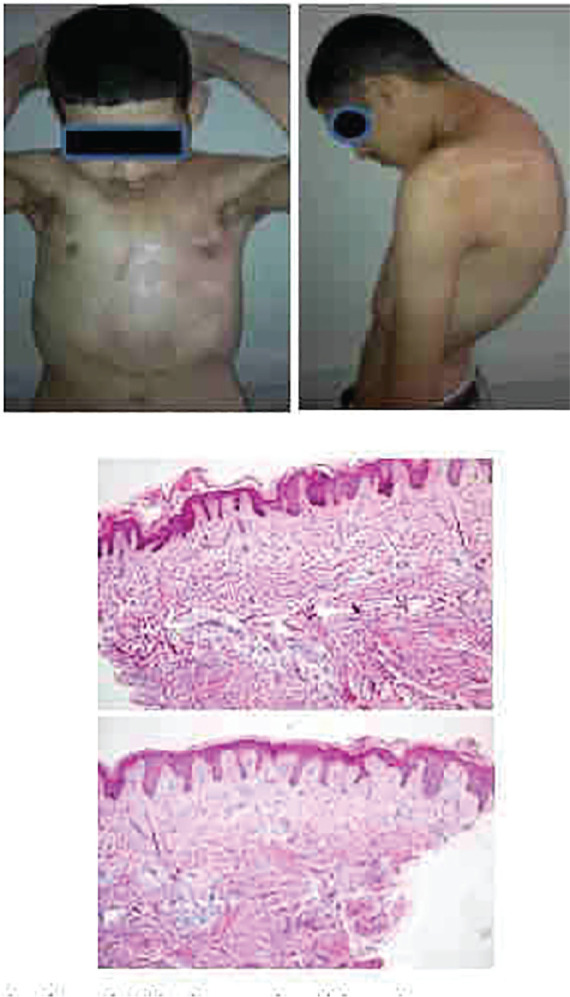


## P.401

## INDUCED SCLERODERMA

V. Babak, M. Starovoytova, O. Desinova, L. Ananieva

*V.A. Nassonova Research Rheumatology Institute, Moscow, Russia*

**Introduction:** Induced scleroderma (IS) is a representative of the scleroderma group of diseases. One of the causes of the development of the disease is the graft versus host disease (GVHD).

**Material and Methods:** Clinical case. A man, 31-years with IS.

**Results:** May 2014 - ESR 34 mm / h, fatigue, sweating, subfebrility for month, an increase in cervical, submandibular, axillary, inguinal lymph nodes, and tooth pain appeared. In the blood test - blast cells. The blast crisis of chronic myeloid leukemia was diagnosed. July 2014 - polychemotherapy. August 2014- the patient was administered imatinib 600-300 mg / day. 02/03/2015 transplantation of allogeneic hematopoietic cell from an unrelated donor. Cellcept 3gr / day and cyclosporin were added to the treatment. 10/26/2015 transfusion of leukoconcentrate. Mesenchymal cells were introduced on 11.16.2015. 03/21/2016 diagnosed with chronic GVHD with damage to the skin (induction), mucous membranes, liver. Prescribed prednisone 45 mg 3-4 months with a dose reduction to 13.75 / day. 10.2017 - rashes of bright red color and areas of hyperpigmentation on the back, neck, shoulders, dry skin. February 2018 - the spread of rash. July 2018 - restriction of movements in the joints of the hands, elbows, knee joints, tightness of the skin of the limbs, face, body. From April to September 2018 - extracorporeal photophoresis (13 sessions) - without a significant effect. August 2018- the dose of prednisone has been increased to 35 mg. September 2018- progressive contractures of the fingers, elbows, knees, ankles. October 2018 - gait disturbance, shortness of breath, feeling of incomplete breath. He was hospitalized with systemic sclerosis (SSc).

Objectively: Diffuse induction (cutaneous score 49 points) and hyperpigmentation of the skin with foci of hypopigmentation, masking of the face, oral aperture of 2.5 cm, sclerodactyly, contractures of the fingers, elbows, knees, ankles due to periarticular fibrosiss, hortness of breath, hepatosplenomegaly. anti-Ro SS-A, anti-La SS-B, anti-Scl -70, anti-RNP -70, ACA, anti-Jo1 - negative; ANF (hep-2) 1/640 cytopl. Lesions of the lungs and esophagus according to MSCT of the chest organs were not detected. Echocardiographia and lung function without clinically significant deviations. There are no capillaroscopic disorders. Sjogren’s syndrome is excluded.

**Conclusions:** The patient has IS developed as a result of GVHD during treatment of myeloid leukemia. Signs of skin lesions and periarticular tissues with the development of joint contractures are characteristic of the diffuse form of SSc. No visceral pathology characteristic of Ssc, immunological and capillaroscopic disorders were detected.

**Figure:**
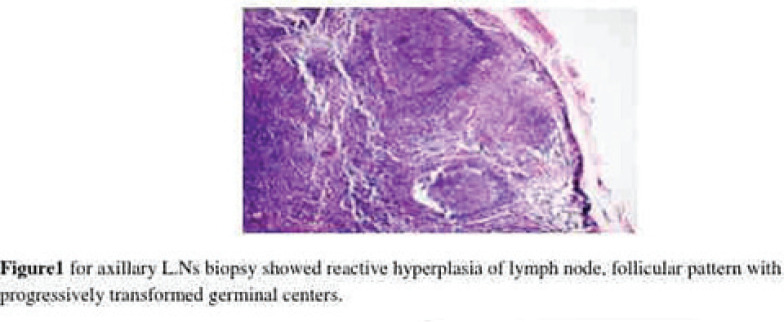


**Figure:**
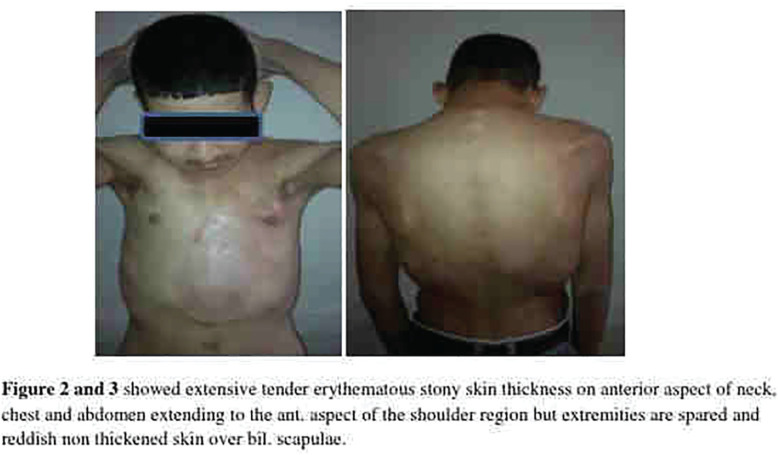


**Figure:**
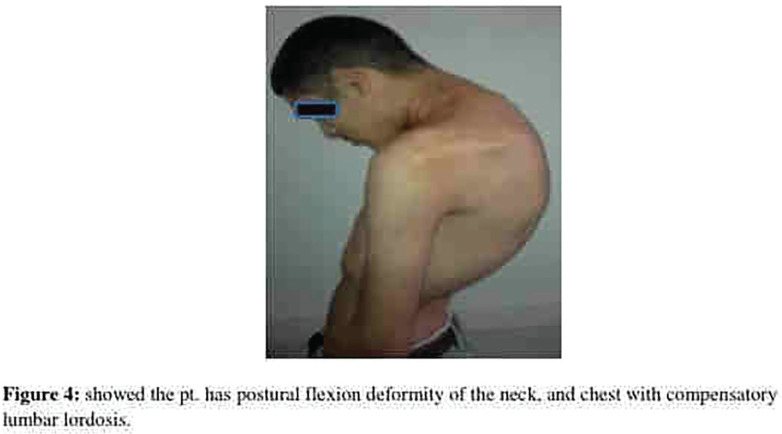


**Figure:**
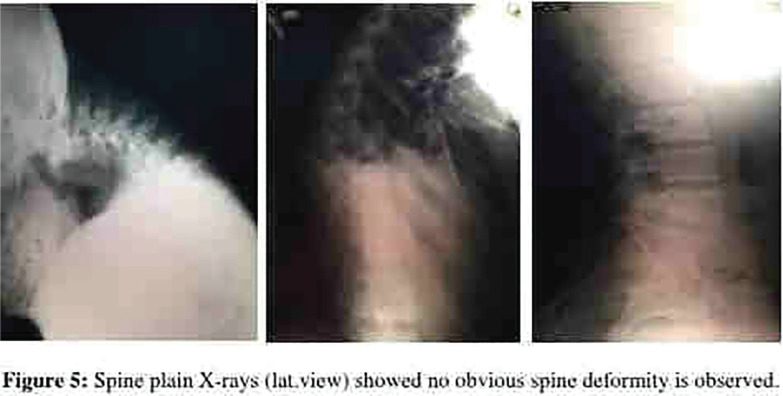


**Figure:**
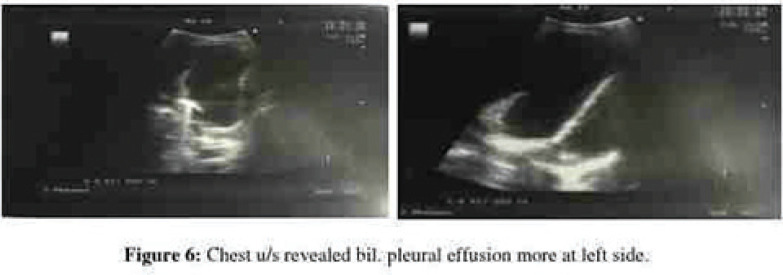


**Figure:**
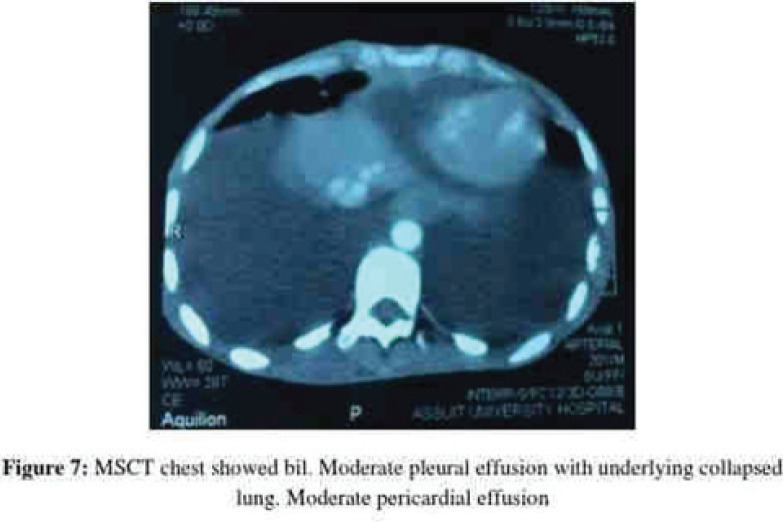


**Figure:**
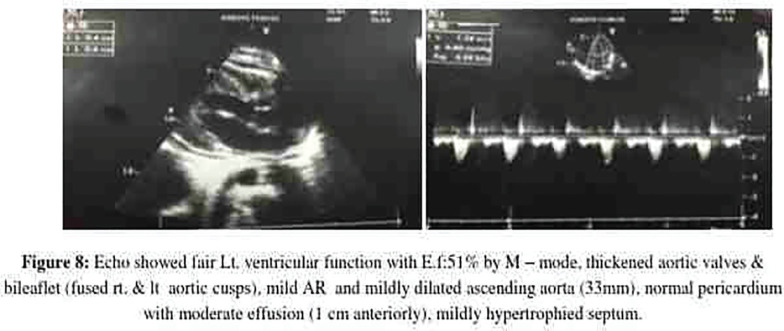


**Figure:**
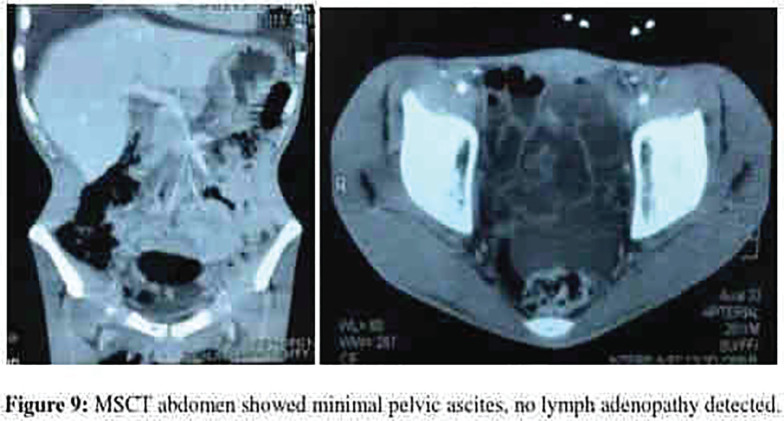


**Figure:**
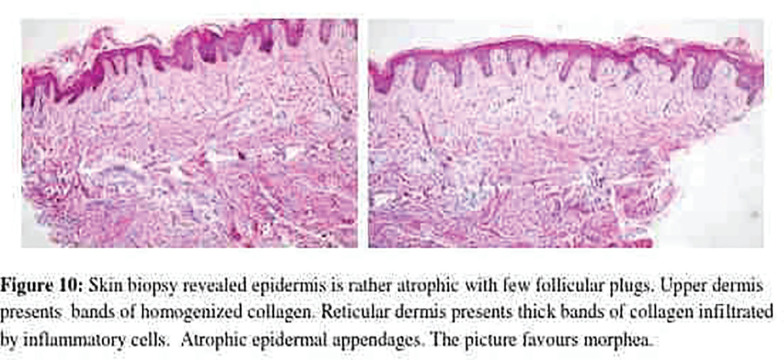


**Figure:**
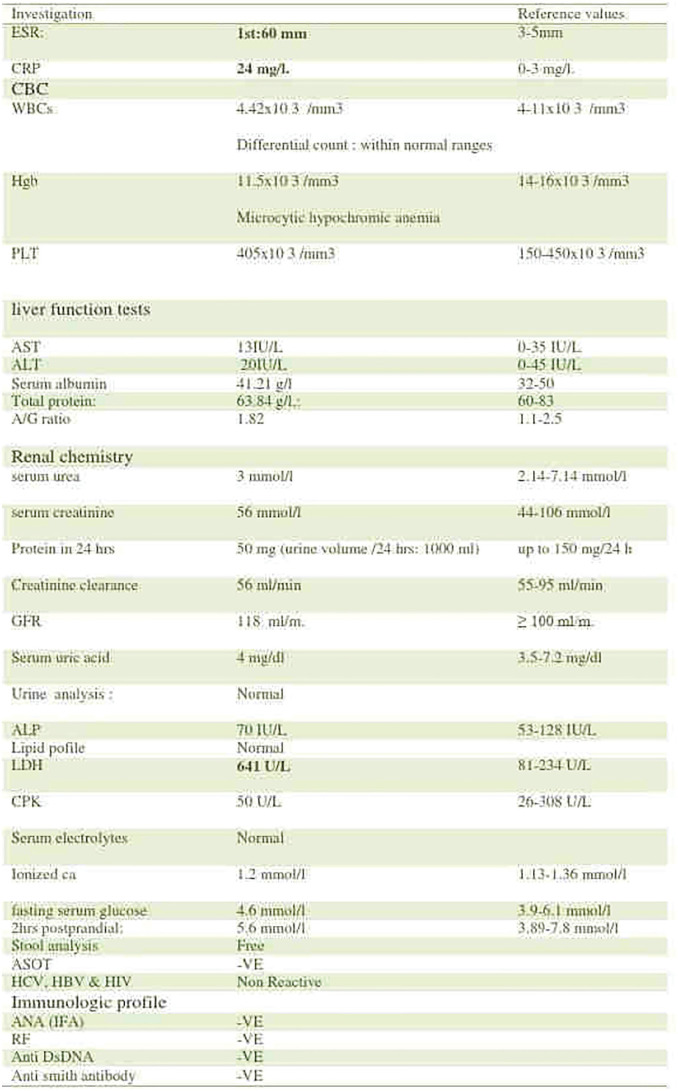


**Figure:**
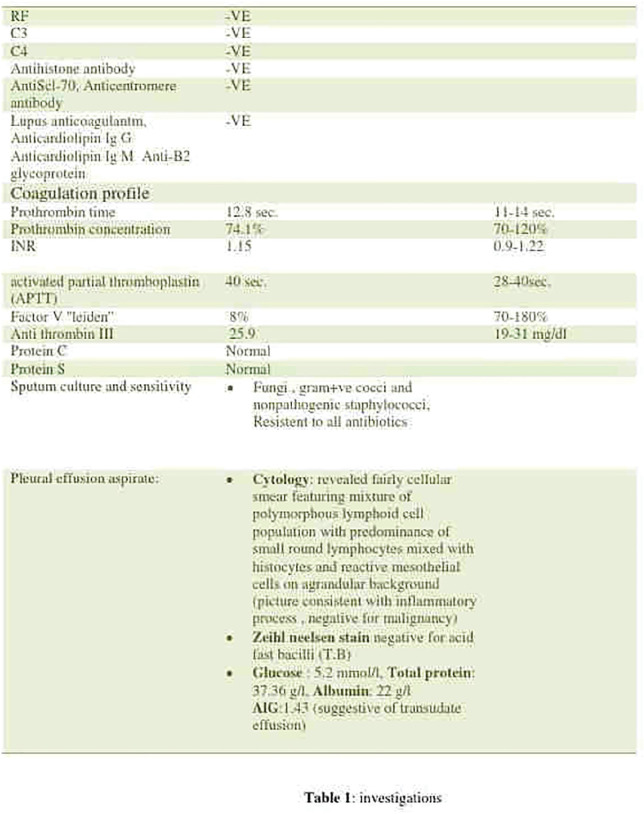


## P.402

## SCLEROMYXEDEMA WITH MULTIPLE SYSTEMIC INVOLVEMENT: INTRAVENOUS IMMUNOGLOBULIN AS STEROID-SPARING AGENT

W. CHOI^1^, S.Y. Kim^2^, K. Lee^1^, H.S. Kim^1^

^1^*Department of Rheumatology, Soonchunhyang University College of Medicine, Seoul, South Korea*, ^2^*Department of Dermatology, Soonchunhyang University College of Medicine, Seoul, South Korea*

**Introduction:** Scleromyxedmea is a rare connective tissue disorder characterized by a generalized lichenoid eruption and sclerodermoid induration with histologic features of dermal mucin deposition. The disease is usually associated with a monoclonal gammopathy and systemic involvements including restrictive lung disease, pulmonary hypertension, esophageal dysmotility and neurologic disorders as well as muscle weakness

**Material and Methods:** A 44-year-old man presented with 3 years history of generalized progressive skin thickening and sclerosis. He had diffuse skin-colored to erythematous firm papules coalescing into indurated plaques on whole body. There were characteristic multiple firm nodules on ears and retroauricular areas and exaggerated skin folds which made deep furrows on glabella (Leonine facies) and the back (Shar-Pei sign). Previously he was diagnosed as scleromyxedema from skin biopsy with monoclonal gammopathy of undetermined significance (MGUS) from the M-peak at serum protein electrophoresis and bone marrow biopsy result at the other tertiary hospital 3 years ago. He had been treated with systemic steroid but systemic symptoms such as dyspnea and dysphagia as well as skin swelling and induration got worsened from one year ago so that he visited our clinic seeking further evaluation and management. He had history of ophthalmologic surgery two months ago due to cataracts on both eyes and central serous chorioretinopathy on the left eye after long-term systemic steroid treatment. A 4mm punch biopsy was taken from a firm papule on the back. Histopathologic examination showed dense dermal collagen and focal separated by clear spaces which contain mucin proved by Alcian blue staining, and superficial and deep dermal perivascular lymphoplasmacytic infiltration, all of which findings were consistent with scleromyxedema. To determine mortality evaluation, he underwent a cardiac catheterization and a pulmonary function test, which revealed restrictive lung disease and constrictive pericarditis without pulmonary arterial hypertension. In serum and urine electrophoresis, there was no monoclonal gammopathy which having been observed before.

**Results:** The patient was received high dose intravenous immunoglobulin (IVIG) therapy once a month. After three courses of IVIG, his cutaneous symptoms, dyspnea and histologic change were improved dramatically.

**Conclusions:** Herein we report a rare case of scleromyxedema with systemic involvement who showed significant improvement after IVIG therapy with histologic change.

**Figure:**
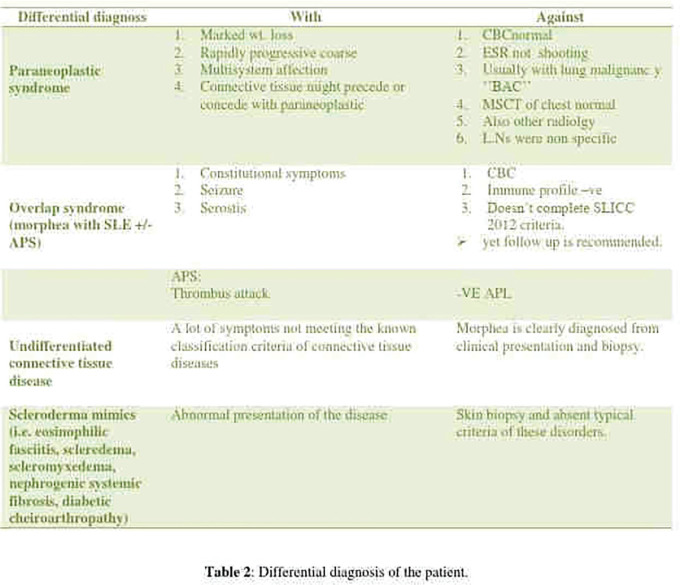


## P.403

## WHY THE SCLERODERMIFORM AFFECTIONS ARE CONSIDERED BORING DISEASES

*G. Chalhoub*

*Central Regional Hospital of Mercy, Ars Laquenexy, France*

**Introduction:** Shulman’s disease or Eosinophilic Fasciitis(EF) is a rare disease. It associates edema of the limbs with eosinophilia which is sometimes inconstantly present, or even absent when the cutaneous induration appears. We report a case of severe sclerdoermiform affection of the limbs.

**Material and Methods:** 36-year-old patient referred to our consultation for establishing a diagnosis in front of bilateral subcutaneous masses of the thighs and para-spinal muscles.

In 2017, the patient presented an inflammatory cellulite type and a fascial collection of the left lateral vast muscle.

thlinical examination showed myalgia of the thighs with bilateral tendinous retraction of the quadriceps and cutaneous sclerosis of the 2 upper limbs, lower limbs and an aspect of scleroderma (en sabre)of the lateral lateral surface of the 2 feet including left foot (brownish and sclerotic appearance).

**Results:** Biology: normal blood count with 118 / mm3eosinophils. Calcium was slightly increased 102mg / l. Normal CPK . CRP was negative. Hepatitis B, C, HTLV, enterovirus and HIV were negatives. Cicatricial EBV. IgM CMV positive with slight positive charge of CMV (may be reactivation).

Serum protein electrophoresis angiotensin converting enzyme were normal.

1 / 640th antinuclear antibodies without specificity of ENA screening.

Spinal and thighs scan: Infiltrations =calcifications of the lumbosacral subcutaneous soft tissues and the root of both thighs with thickening of the different fasciae + inguinal lymphadenopathy: chronic fasciitis.

Lumbar MRI : amyotrophy of the upper left gluteus maximus muscle with superior muscular edema of the gluteus maximus and thickening of the fascia with irregularity and subcutaneous infiltration, extended from L4-L5 to S5 without underlying collection.

Aspect in favor of myositis of gluteus maximus with chronic fasciitis that may correspond to a Shulman syndrome.

Cutaneous biopsy: normal skin architecture.

Calcification biopsy: SUBCUTANEOUS CALCINOSIS, WITHOUT SIGN OF SPECIFICITY.

Cardiac ultrasound wasnormal

PET FDG scan: normal .

We conclude to a Schulman’s disease .

HYDROXYCHLOROQUINE was started + low dose of steroides of 10mg/day,

The efficacy of the treatment, evaluated at 2 months, showed a partial response. An immunosuppressive treatment was started with METHOTREXATE.

**Conclusions:** The diagnosis of EF is based on the clinical exam+ MRI(showing a thickening of superficial+deep fascia) and confirmed by the histology. Steroids are effective in the majority of cases. Immunosuppressants must be combined in case of corticosteroids.

However, the presence of monoclonal gammopathy is frequently described in EF, the absence of eosinophilia and monoclonal gammopathy in our case makes the diagnosis difficult.

**Figure:**
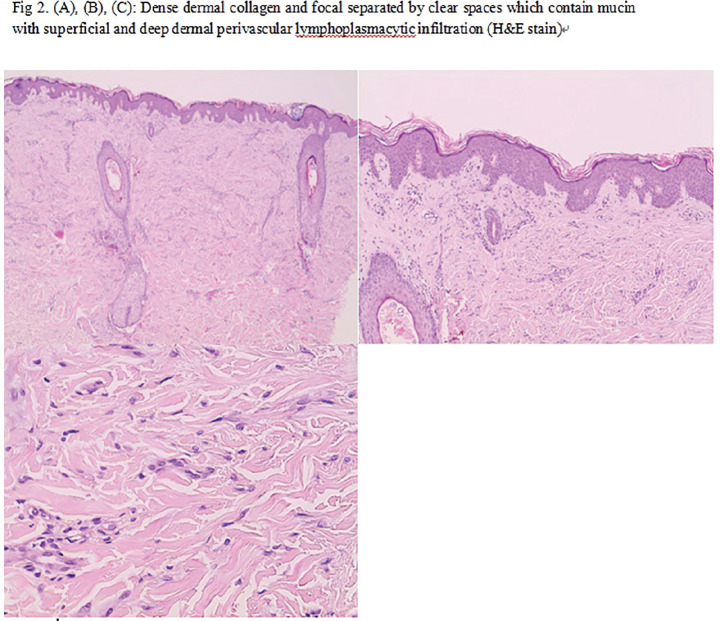


## P.404

## TOFACITINIB IS A THERAPEUTIC OPTION FOR REFRACTORY EOSINOPHILIC FASCIITIS

X. Cao, J. Zhao, Y. Hou, D. Xu, M. Li, Q. Wang, X. Zeng

*Department of Rheumatology and Clinical Immunology, Peking Union Medical College Hospital, Chinese Academy of Medical Sc, Beijing, China*

**Introduction:** We presented a case in which tofacitinib was effcetive for treating refractory eosinophilic fasciitis (EF).

**Material and Methods:** On July 2017, a 68-year-old man manifested as skin-pitting edema and erythema affecting bilateral forearms and calf. Gradually, the sclerosis of proximal limbs, neck, and trunk replaced the previous leisions. A peaud’ orange appearance and “groove sign” were prominent. The patient denied the Raynaud’s phenomenon. Laboratory examines revealed elevated erythrocyte sedimentation rate and hypersensitive C-reactive protein, and peripheral eosinophilia. The diagnosis of EF was made and oral prednisone 30 mg daily was prescribed and tapered gradually. Unfortunately, skin induration continued to progress. Numbness of extremities beyond the bilateral knee and elbow joint developed. The oral cyclophosphamide (CTX) 100 mg every other day was administered in March 2018. But the patient felt difficulty in dorsiflexion of the left foot, in May 2018. He was hospitalized in Peking Union Medical College Hospital on June 11, 2018. The patient agreed to off-label therapy involving tofacitinib and was given oral tofacitinib at a dose of 5 mg twice daily. The CTX was discontinued, and prednisone was tapered quickly.

**Results:** After five months of tofacitinib treatment, the clinical symptoms were improved remarkably.

**Conclusions:** The tofacitinib may be a potential therapeutic option for refractory EF.

## P.405

## ACR/EULAR 2013 CLASSIFICATION CRITERIA FOR SYSTEMIC SCLEROSIS: AN EXAMPLE OF FEMALE PATIENT WITH EARLY FORM OF SCLERODERMA DIAGNOSED AT CHILDHOOD

K. Bouchalova^1^, P. Horak^2^, D. Pospisilova^1^

^1^*Department of Pediatrics, Faculty of Medicine and Dentistry, Palacky University and University Hospital, Olomouc, Czech Republic*, ^2^*Dpt of Internal Medicine III-Nephrology, Rheumatology and Endocrinology, Faculty of Medicine and Dentistry, Palacky Univ, Olomouc, Czech Republic*

**Introduction:** Scleroderma represents a rare disease in childhood. Traditional classification criteria were based on presence of skin findings. Nevertheless, in the largest published series (Padua) 79/153 children patients lack these symptoms (Martini et al., 2006). Nowadays, ACR/EULAR 2013 Classification Criteria for Systemic Sclerosis, developed for adults are used also in pediatrics and enable us to diagnose disease at early stage.

**Material and Methods:** Case report of 17 year old female patient with prominent Raynaud phenomenon and pathological capillaroscopy. Positive autoantibodies associated with systemic sclerosis developed later.

**Results:** The patient was send by dermatologist due to pathological capillaroscopy, associated with Raynaud phenomenon. No skin or other symptoms of systemic sclerosis were present. Baseline autoantibodies were normal. Positive autoantibodies and capillaroscopy progression were found after one year of follow up. No other systemic involvement was detected. Surprisingly, right eye vasculitis was diagnosed. Markers of inflammation and urine tests were negative. Combination of vazodilatators (amlodipine, naftidrofuril hydrogenooxalas, Gingium) had no effect. Due to age, a consultation with adult rheumatologist was performed and led to consensus on methotrexate immunosuppression therapy initiation. Eye vasculitis and autoantibodies disappeared.

**Conclusions:** Systemic sclerosis without skin symptoms is found in a significant proportion of patients. Early diagnosis is crucial for therapy initiation.

## P.406

## DILTIAZEM-INDUCED MYOSITIS IN A PATIENT WITH SYSTEMIC SCLEROSIS

S. Berthier^1^, A. Grandvuillemin^2^, B. Nicolas^1^, A. Guilhem^1^, V. Leguy^1^, M. Samson^1^, S. Audia^1^, B. Bonnotte^1^

^1^*Department of internal medecine, CHU, Dijon, France*, ^2^*Department of pharmacology, CHU, Dijon, France*

**Introduction:** Calcium channel blockers are recommended in systemic sclerosis (SSc ) in order to improve or prevent the microvascular damage. Macrovascular disease also affects patients with SSc. Thus, statins can be recommended as increased prevalence of atherosclerosis in SSc has been reported. We describe the case of a patient who has developed myositis after diltiazem introduction.

**Material and Methods:** Caucasian male of sixty-seven years old was diagnosed with diffuse SSc. At diagnosis, he presented progressive thickening of the skin since nine months ago (Rodnan score: thirty), digital ulcers, puffy fingers, dysphagia, anti Scl seventy autoantibodies. Ten years ago, he had suffered an ischemic stroke with atrial fibrillation and beta-blocker, anti – vitamin K antagonist, ezetimibe and simvastatine were introduced. At the moment of the diagnosis of SSc, beta- blocker was switched with calcium channel blocker (diltiazem).

Six months after the switch, he developed myalgia, proximal muscle weakness and elevated muscle enzymes (CK: eight hundred forty- seven UI/L) without any inflammatory syndrome. Anti Scl seventy autoantibodies were positive. TSH was normal and HMGCoA auto antibodies were negative. No pulmonary damage was documented. Electromyography was abnormal and muscle biopsy showed HLA-I overexpression without any inflammatory infiltrate. Statin-induced myopathy was suspected and simvastatin was stopped. However, muscle weakness was unchanged eight weeks later. Then, methotrexate with low doses of prednisone was administered to the patient who improved in two months.

**Results:** Simvastatin, used as cholesterol – lowering agent, is metabolized by cytochrome P four hundred fifty- three A enzymes (CYPthreeA) whereas Diltiazem inhibits CYPthreeA enzymes. This therapeutic association increased the serum concentration and the area under the curve of simvastatin. So, their co-administration increases the risk of statin-induced myopathies. Interestingly, erythromycin has the same effect.

**Conclusions:** Statin should be used very carefully in patients suffering from SSc. When a statin is really needed to prevent atherosclerosis in SSc, pravastatin should be preferred because this statin, unlike other statins, does not increase the risk of induced myopathy when co-administered with diltiazem.

## P.407

## MALIGNANCY IN A SINGLE CENTER COHORT OF SYSTEMIC SCLEROSIS PATIENTS

A. Balbir Gurman^1^, V. Shataylo^2^, A. Mashiah^2^, N. Schultz^2^, E. Sabbah^2^, Y. Tavor^2^, M. Nahir^2^, K. Dolnikov^2^, N. Finkilstein^2^, Y. Braun-Moskovici^1^

^1^*Rheumatology Institute, Rambam Health Care Campus, Rappaport Faculty of Medicine, Technion, Haifa, Israel*, ^2^*Rheumatology Institute, Rambam Health Care Campus, Haifa, Israel*

**Introduction:** A link between scleroderma (SSc) and malignancy may be related to autoimmunity, inflammation, tissue ischemia, fibrosis, immunosuppression, and often x-ray exposure. We evaluated the incidence of malignancy in SSc patients at our center and correlation with clinical data.

**Material and Methods:** In a retrospective case-control study of 424 SSc patients (343 EUSTAR registry participants) 43 (10.1%) patients with malignancy (SSc-Ca) confirmed by biopsy or imaging were identified and compared to 86 SSc patients without cancer (cancer free, CF, N=86); CF patients were divided to age (Age-CF, N=43) and disease duration matching (DD-CF, N=43).

**Results:** SSc-Ca patients [7 males (16.3%); age (years, SD) 68.1 (12.7); age at SSc onset 52.9 (14.9) and at malignancy diagnosis 54.2 (12.7) and smoking 12 (27.9)] had malignancies: breast 9; lung 8; neck & head 8 (brain 3, thyroid 3, parotid 1, larynx 1); genital-urinary 8 (prostate 1, ovary 3, uteri 4; GIT 6 (stomach 2, pancreas 2, colon 2); hematologic 3 (lymphoma 2, myeloma 1); skin 3; pheochromocytoma 1, carcinoid 2; sarcoma 1. In 13 (30.2%) patients’ cancer appeared three years before SSc; 7 patients had cancer long before SSc; 6 patients had two cancers. There was not significant difference in term of SSc subset, antibodies status, digital ulcers, ILD, PAH, myositis, polyautoimmunity, GIT and heart involvement between SSc-Ca, Age-CF, DD-CF and all CF patient. Patients with solid tumor did not have SRC compared to 8 (9.3%) patients in CF patients. Among SSc-Ca patients there were 26 (60.5%) deaths compared to 16 (37.2%) in Age-CF (P<0.08), DD-CF (P<0.001), and all CF (P<0.001) patients. There was no difference between SSc related deaths in all-CF patients (22.1%) and SSc-Ca (16.3%); 19 SSc-Ca patients dead from cancer (73.1%). There were no differences between SSc-Ca and SSc-CF patients regarding treatment with methotrexate, mycofenolate, azathioprine, cyclophosphamide, calcium blockers and iloprost; 44.5% of SSc-CF patients were treated with bosentan compared to 24.3% SSc-Ca patients (P<0.015).

**Conclusions:** Malignancy could appear long before SSc, close to SSc appearance and in long-term SSc course. SSc-Ca patients had high mortality rate and mostly dead from cancer. Solid tumors were not associated with SRC; other SSc complications were reported similarly in SSc-Ca and SSc-CF patients. Treatment with bosentan was associated with less incidence of malignancy. SSc complication may mask underlying malignancy; awareness and early screening for cancer should be considered as a reasonable approach in caring SSc patients; early diagnosis and treatment of tumor may improve SSc patients’ morbidity and survival.

## P.408

## IGG-4 RELATED AORTITIS IN A PATIENT WITH RA-SSC OVERLAP, AN UNEXPECTED COMBINATION

A. Balbir Gurman^1^, Y. Tavor^2^, L. Guralnik^3^, Y. Braun-Moscovici^1^

^1^*Rheumatology Institute, Rambam Health Care Campus, Rappaport Faculty of Medicine, Technion, Haifa, Israel*, ^2^*Rheumatology Institute, Rambam Health Care Campus, Haifa, Israel*, ^3^*Radiology Department, Rambam Health Care Campus, Rappaport Faculty of Medicine, Technion, Haifa, Israel*

**Introduction:** Systemic sclerosis (SSc) is often a part of Overlap syndrome; in this regard myositis, rheumatoid arthritis (RA), Sjogren’s syndrome and systemic lupus erythematosus are listed. Vasculitis had been reported in patients with SSc.

**Material and Methods:** We present here a patient with SSc-RA overlap who developed severe IgG-4 related aortitis.

**Results:** A 59-year-old female was diagnosed with RA-SSc overlap 20 years ago; rheumatoid factor, ANA, p-ANCA and anti-topoisomerase antibodies were posotive. Her main problems were arthritis, sclerodactyly, Raynaud’s phenomenon, limited skin disease, and heartburnShe was treated with steroids for several years with gradually tapering after resolution of arthritis and softening of skin. In August 2018 the patient developed severe fatigue, high fever, sweating and weight loss for several weeks. On admission she had typical SSc face changes and mild sclerodactyly and telangiectasia; there was no rash, lymphadenopathy, arthritis or neurologic deficit. Auscultation of heart and lungs was unremarkable; there was mild abdominal tenderness without organomegaly. Blood tests: leucocytes 19.5X103/µl, hemoglobin 8.1 g/dl, platelets 846.0 X103/µl, ESR 76 mm/h, CRP 15.6 mg/dl, creatinine 1.3 mg/dl, ferritin 464 mg/ml, albumin 3.1 g/dl; liver enzymes, creatine kinase, TSH, and urinalysis were normal. Blood and urine cultures, tests for CMV, EBV, Parvovirus, Legionella, Mycoplasma, Q Fever, West Nile Fever were negative. Panendoscopy was unremarkable. Chest and abdominal CT showed esophageal widening, limited areas of bibasilar lung honeycombing; there were no lymphadenopathy or organomegaly; thoracic aorta and branches were normal but approximately 3.5cm of abdominal aorta showed significant wall thickening consistent with aortitis

There was a noticeable thickness of approximately 3.5 cm in the distal part of the abdominal aorta compatible with aortitis. Bone biopsy and bone marrow were normocellular with no evidence for hematologic malignancy but positive lymphocytes staining for IgG4; serum IgG4 was 200 ng/dl (upper limit 140 ng/dl). Treatment with methylprednisolone infusions 100 mg/day, followed with prednisone 60 mg/day and oral methotrexate 20 mg/week were started; after resolution of fever and normalization of blood tests doses of prednisone were gradually reduced to 5 mg/day during the following year. On her visit in August 2019, the patient is asymptomatic; blood count, creatinine, IgG4, and acute phase reactants are normal. Repeated MRI scans showed gradual and finally complete resolution of aortitis.

**Conclusions:** Overlap between SSc and vasculitis has been reported; large vessels vasculitis is extremely rare in SSc. Hitherto, there were no reports on IgG4 related diseases and particular on IgG4 related aortitis in patients with SSc.

## P.409

## DRAMATIC SCLERODERMA DEBUT WITH MALIGNANT HYPERTENSION AND RENAL CRISIS

A. Balbir Gurman^1^, G. Milo^2^, S. Assady^2^, R. Dragu^3^, K. Dolnikov^4^, Y. Braun-Moscovici^1^

^1^*Rheumatology Institute, Rambam Health Care Campus, Rappaport Faculty of Medicine, Technion, Haifa, Israel*, ^2^*Department of Nephrology and Hypertension, Rambam Health Care Campus, Rappaport Faculty of Medicine, Technion, Haifa, Israel*, ^3^*Internal Medicine C, Galilee Medical Center, Naharia, Israel*, ^4^*Rheumatology Institute, Rambam Health Care Campus, Haifa, Israel*

**Introduction:** Scleroderma renal crisis (SRC)] is a known complication of early diffuse systemic sclerosis (SSc). Arterial hypertension resistant to treatment (BP), rapidly progressive renal failure, anuria and hemolytic anemia are anchor signs of SRC. Uncontrolled hypertension may lead to myocardial infarction, cerebral ischemia, pulmonary edema, etc.

**Material and Methods:** We present two patients with malignant hypertension as a catastrophic debut of SSc.

**Results:** A 46-year-old previously healthy female suddenly developed pulmonary edema with BP 220/120 mm Hg and new onset renal failure. She was intubated and treated with diuretics, nitroprusside and beta-blockers with resolution of pulmonary edema. Transient discoloration of fingers suggested the possibility of Raynaud’s phenomenon (RP). Tests for ANA, ATA, ANCA, RNAPIII were negative. Kidney biopsy (KB) showed concentric intimal hyperplasia and hyalinization of the intima in small renal arteries without immune deposits. Angiotensin-converting enzyme inhibitors (ACE) and calcium channel blockers (CCB) were started with normalization of BP and renal function. During the following years, the patient developed SSc characteristic face changes, sclerodactily, digital ulcers, GAVE and moderate but stable renal impairment.

A 65-year-old-female obese patient, otherwise healthy, was admitted with new-onset progressive dyspnea and chest discomfort. She collapsed in the emergency room and underwent successful resuscitation for pulmonary edema and asystole. During the next several days she was treated with high doses of furosemide, normopressan and labetalol; her BP remained above 180/100 mm Hg. The patient complained of pain and discoloration of her fingers; the physical exam was positive for Raynaud phenomenon and tendon friction rubs; the skin was unremarkable. Blood tests revealed creatinine 1.6 ml/dL, hemoglobin 8.5 g/dL with no schistocytosis or bilirubin elevation; troponin and BNP were slightly elevated; tests for ANA and RNAPIII were highly positive. Endoscopy revealed GAVE . Typical ‘onion skin’ arterial lesions were demonstrated on KB. Switch to captopril and amlodipine led to rapid normalization of BP and creatinine. During the next two years the patient developed skin thickening up to elbows, sclerodactily and digital ulcers. Her condition is stable under treatment with cellcept, ramipril, amlodipine, iloprost infusions and bosentan.

**Conclusions:** SRC and malignant hypertension may be an early SSc feature. As approach for SRC-related malignant hypertension is principally different from other hypertension emergencies, proper diagnosis is crucial. Awareness of SRC is essential in cases of unexplained malignant hypertension; involvement of rheumatologists and nephrologists in such cases may change diagnostic and treatment paradigm with positive impact on patient outcome.

